# Associations Between Gastroenteropancreatic Neuroendocrine Neoplasms and Inflammatory Factors: Insights From a Two‐Sample Mendelian Randomization Analysis

**DOI:** 10.1155/cjgh/2591387

**Published:** 2025-12-26

**Authors:** Huimin Guo, Yu Li, Bowei Liu, Songtao Liu

**Affiliations:** ^1^ Department of Nuclear Medicine, Shandong Provincial Hospital Affiliated to Shandong First Medical University, Jinan, 250021, Shandong, China, sph.com.cn; ^2^ Department of Imaging, Laiwu People’s Hospital, Jinan, 271100, Shandong, China; ^3^ Coventry College, Communication University of China, Lingshui, 572400, Hainan, China, cuc.edu.cn; ^4^ Department of Nuclear Medicine, Cheeloo College of Medicine, Qilu Hospital (Qingdao), Shandong University, Qingdao, 266035, Shandong, China, sdu.edu.cn

**Keywords:** causal inference, gastrointestinal neuroendocrine neoplasms, immune cells, inflammatory proteins, Mendelian randomization

## Abstract

**Purpose:**

Inflammation is implicated in the pathogenesis of gastroenteropancreatic neuroendocrine neoplasms (GEP‐NENs); however, the causal nature of this association remains unclear. This study sought to evaluate the causal relationships between GEP‐NENs and inflammatory factors using a two‐sample Mendelian randomization (MR) approach.

**Methods:**

We performed a two‐sample MR analysis to investigate the causal associations between 91 inflammatory proteins and 731 immune cell traits as exposures and the five subtypes of GEP‐NENs as the outcomes. The analytical approach employed various methodologies, such as inverse variance weighting, MR‐Egger, weighted mode, weighted median, and simple mode. To evaluate the robustness of the results, sensitivity analyses were conducted, which encompassed MR Egger regression, MR multiple gene residual and outlier detection, leave‐one‐out analysis, and Cochran’s *Q* test. False discovery rate (FDR) correction was applied, and causal relationships at the gene level were deemed significant at *p* < 0.05 after FDR adjustment.

**Results:**

After FDR correction, the findings revealed robust causal associations between genetically predicted HLA DR++ monocyte %leukocyte (OR = 3.09, 95% CI: 1.76–5.44, *p* < 0.001, FDR = 0.022), HLA DR on CD14+ CD16‐ monocyte (OR = 1.72, 95% CI: 1.34–2.22, *p* < 0.001, FDR = 0.010), and HLA DR on CD14+ monocyte (OR = 1.76, 95% CI: 1.36–2.29, *p* < 0.001, FDR = 0.010) and genetically predicted stomach NENs. Reverse analysis revealed that GEP‐NENs had no major impact on inflammation.

**Conclusion:**

These findings reveal the immune mechanisms underlying GEP‐NENs and highlight potential therapeutic strategies targeting the immune microenvironment of GEP‐NENs.

## 1. Introduction

Neuroendocrine neoplasms (NENs) are a heterogeneous group of potentially malignant tumors that arise from the secretory cells of the neuroendocrine system. Gastroenteropancreatic NENs (GEP‐NENs), a subset of NENs, encompass tumors originating in the gastrointestinal tract and pancreas [1]. Although traditionally considered rare and slow‐growing, recent data have indicated a significant increase in the global incidence and prevalence of GEP‐NENs [2]. Despite improvements in prognosis, GEP‐NENs remain associated with substantial mortality [2]. These tumors are clinically heterogeneous, varying in hormonal activity and clinical presentation depending on their anatomical origin, which often leads to a delayed diagnosis, particularly for nonhormone‐producing tumors [3]. Given the clinical complexity and challenges of early detection, identifying reliable biomarkers for prognosis and therapy remains crucial for improving patient outcomes. Emerging evidence underscores the critical roles of inflammation in the initiation and progression of GEP‐NENs, including neutrophils [4], inflammatory cytokines [5, 6], and the neutrophil‐to‐lymphocyte ratio [7]. Chronic or acute inflammation may induce cellular transformation in susceptible cells, whereas intricate interactions between cancer cells and the tumor microenvironment (TME) significantly influence therapy resistance and prognosis in patients [8]. Sustained inflammatory cytokine production in the TME supports tumor growth and progression [9], including GEP‐NENs [10]. Neuroendocrine cells that are overstimulated by inflammation can undergo transformation [11]. However, the value of inflammatory predictive and prognostic biomarkers for GEP‐NENs remains controversial [11], possibly because the available epidemiological studies that revealed associations between inflammation and NENs [10–12] cannot establish causality [13]. Exploring the complex relationship between inflammatory mechanisms and GEP‐NETs may lead to the development of novel approaches for the early identification and implementation of targeted treatment strategies.

Mendelian randomization (MR) is a widely adopted analytical approach used to infer causal relationships between exposures and outcomes by leveraging genetic variants as instrumental variables (IVs) [14]. By utilizing genetic variants as IVs, MR mitigates the effects of confounding and reverse causation, which frequently challenge the validity of traditional epidemiological studies [15]. The objective of this research was to investigate the causal relationships among inflammatory cytokines, immune cell populations, and GEP‐NENs utilizing MR methodology. These findings may enhance our understanding of GEP‐NEN etiology and help identify novel biomarkers for early detection and targeted therapies.

## 2. Methods

### 2.1. Ethical Considerations and Study Design

This study utilized publicly available summary statistics from genomewide association studies (GWAS) that obtained all necessary ethical approvals, participant consent, and permissions in the original studies. As no new data were generated for this analysis, additional ethical approvals were not required.

**Figure 1:**
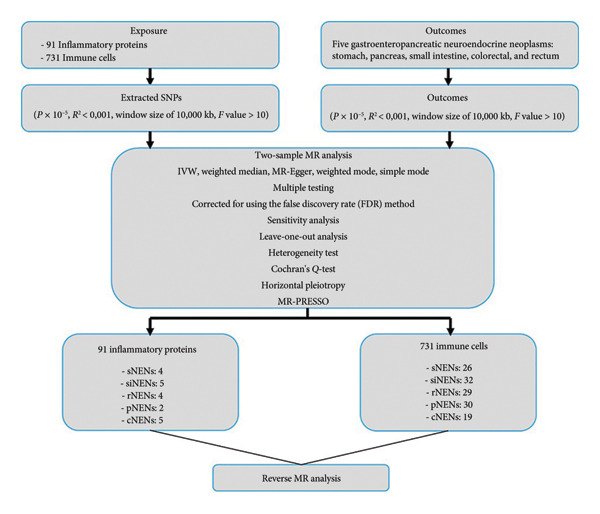
Flowchart for the Mendelian randomized analysis. Abbreviations: cNENs = colorectal neuroendocrine neoplasms, pNENs = pancreas neuroendocrine neoplasms, rNENs = rectum neuroendocrine neoplasms, siNENs = small intestine neuroendocrine neoplasms, and sNENs = stomach neuroendocrine neoplasms.

The study workflow is depicted in Figure 1, which outlines the two‐sample MR framework used to assess causal associations. In brief, peripheral immune cells and inflammatory proteins were used as exposure indicators, whereas GEP‐NENs, including stomach NENs (sNENs), pancreatic NENs (pNENs), small intestinal NENs (siNENs), colorectal NENs (cNENs), and rectal NENs (rNENs), were used as outcomes. Rigorous inclusion and exclusion criteria were implemented to identify single‐nucleotide polymorphisms (SNPs) that exhibited a strong association with circulating inflammatory proteins and immune cell characteristics, which were subsequently used as IVs. Causal associations were validated using multiple sensitivity analyses. In addition, reverse MR analysis was performed to evaluate the potential bidirectional effects and assess whether GEP‐NENs influence the levels of pathogenic inflammatory proteins and immune cell traits.

### 2.2. Data Sources

This investigation employed data classified into exposures and outcomes to conduct a two‐sample MR analysis. Outcome data concerning GEP‐NENs were acquired from the R10 release of the FinnGen project (accessible at https://www.finngen.fi/en/access_results), which is an extensive initiative involving a Finnish biobank that amalgamates genomic and health‐related information from the entire Finnish population, aimed at exploring disease etiology and improving public health outcomes [16]. The exposure data comprised two distinct elements: 91 inflammatory proteins and 731 immune cell characteristics’ data points. Information on the 91 inflammatory proteins was obtained from the EBI GWAS Catalog (accession numbers: GCST90274758–GCST90274848; available at https://www.ebi.ac.uk/gwas/downloads/summary-statistics) [17]. Data pertaining to the 731 immune cell characteristics were extracted from the GWAS Catalog database (http://ftp.ebi.ac.uk/pub/databases/gwas/summary_statistics) [18]. All GWAS datasets used in this MR analysis were derived from individuals of European descent. Further details of the datasets are provided in Supporting Table S1, respectively.

### 2.3. Genetic IV Selection

First, we established a stringent threshold (*p* < 1 × 10^−5^) as the genomewide significance cutoff to identify SNPs highly correlated with inflammation and GEP‐NENs, following previous research [19–25]. To reduce the impact of linkage disequilibrium (kb = 10,000, *R* ^2^ = 0.001), a clumping procedure was employed, which is a technique frequently used in MR studies [14]. *F*‐statistics were computed to evaluate the strength of the IVs using the equation *F* = ((*N* − *k* − 1)/*k*) × *R* ^2^/(1 − *R* ^2^), where IVs yielding an *F*‐statistic below the threshold of 10 were excluded, as they were deemed weak based on established guidelines [26, 27]. In addition, a harmonization step was conducted to guarantee the alignment of alleles between SNPs associated with the exposure and the outcome, which involved eliminating palindromic SNPs and those exhibiting incompatible alleles (Supporting Tables S2 and S3).

### 2.4. Primary Analysis and Sensitivity Analysis

MR analyses were performed using the TwoSampleMR and MR‐PRESSO packages in the R programming environment (Version 4.3.3) [28]. The main analysis employed the IVW approach to calculate the odds ratios (ORs) and their corresponding 95% confidence intervals (CIs), thereby assessing the causal influences of exposures on outcomes. Additional analyses were conducted using MR‐Egger regression, weighted median, simple mode, and weighted mode techniques to verify the robustness of the findings [27, 29, 30]. Sensitivity analyses were subsequently conducted, including leave‐one‐out analysis to assess the influence of individual SNPs, Cochran’s *Q* test to evaluate heterogeneity (*p* < 0.05, indicating significant heterogeneity among SNPs), and MR‐Egger regression along with MR‐PRESSO to detect horizontal pleiotropy and outliers.

The following criteria guided the identification of potentially suitable candidate inflammation IVs associated with GEP‐NENs: (i) consistency in amplitude and direction across the results of the five MR analyses and (ii) absence of pleiotropy and heterogeneity. To address the issue of multiple comparisons, *p* values were adjusted using the Benjamini–Hochberg false discovery rate (FDR) procedure. Importantly, FDR correction was performed separately for each exposure type (i.e., inflammatory proteins and immune cell parameters), rather than uniformly across all indicators, to ensure the validity of the association between each exposure and GEP‐NENs. Exposures with initial *p* < 0.05 were deemed to have a possible causal association with GEP‐NENs. A causal relationship at the gene level was considered if the *p* value was still < 0.05 after FDR.

### 2.5. Reverse Causality Analysis and Genetic Correlation

Reverse MR analysis was performed to rule out a potential bidirectional relationship between inflammation and GEP‐NENs. This study specifically targeted GEP‐NENs and identified their inflammatory biomarkers. The IVs used in this study were chosen according to a genomewide significance threshold set at *p* < 5 × 10^−5^ (kb = 10,000, *R* ^2^ = 0.001) (Supporting Table S4).

## 3. Results

### 3.1. Strength of the IVs

We examined the causal relationships between 91 inflammatory proteins, 731 circulating immune cell traits, and the risk of GEP‐NEN. For inflammatory proteins, the number of IVs ranged from 21 to 45 SNPs, with *F*‐statistics ranging from 19.51 to 1169.91 (mean = 40.36; median = 21.44). For immune cell traits, 4–37 SNPs were used as IVs, with *F*‐statistics ranging from 19.62 to 29.55 (mean = 21.60; median = 21.03). All IVs exceeded the threshold of *F* > 10, confirming sufficient instrument strength (Supporting Tables S2 and S3).

### 3.2. Overall Causal Associations

A total of 22 suggestive associations were initially detected using the IVW method (*p* < 0.05). After removing associations with evidence of pleiotropy or heterogeneity, four, two, five, five, and four robust associations remained for sNENs, pNENs, siNENs, cNENs, and rNENs, respectively (Supporting Table S5).

**Figure 2:**
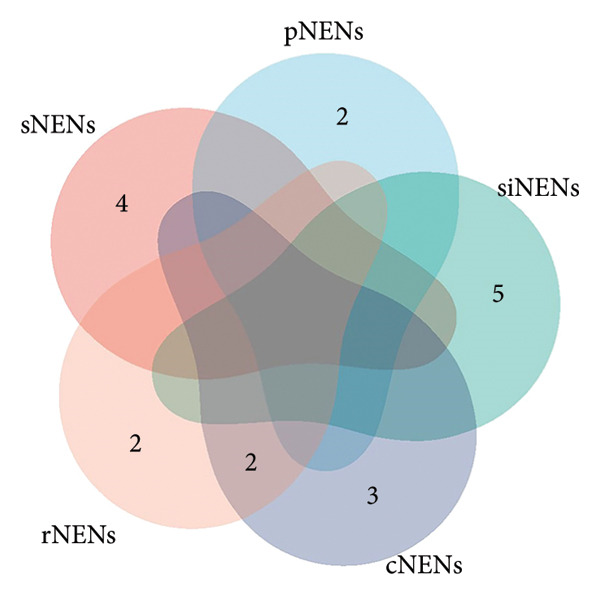
Venn diagram illustrating the number of 91 inflammatory proteins causally associated with various gastroenteropancreatic neuroendocrine neoplasms. Abbreviations: cNENs = colorectal neuroendocrine neoplasms, pNENs = pancreas neuroendocrine neoplasms, rNENs = rectum neuroendocrine neoplasms, siNENs = small intestine neuroendocrine neoplasms, and sNENs = stomach neuroendocrine neoplasms.

**Figure 3:**
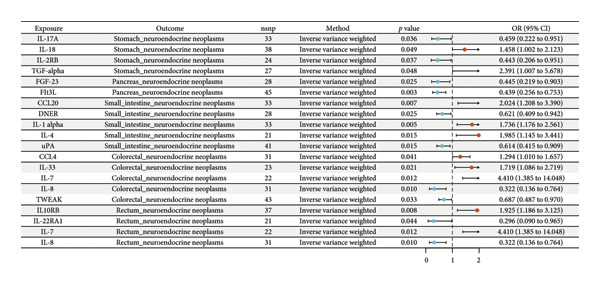
Forest plot for the causal effect of identified 91 inflammatory proteins on the risk of five types of gastroenteropancreatic neuroendocrine neoplasms derived from inverse variance weighted (lVW, *p* < 0.05). Abbreviations: Cl = confidence interval, OR = odds ratio, and SNP = single‐nucleotide polymorphism.

**Figure 4:**
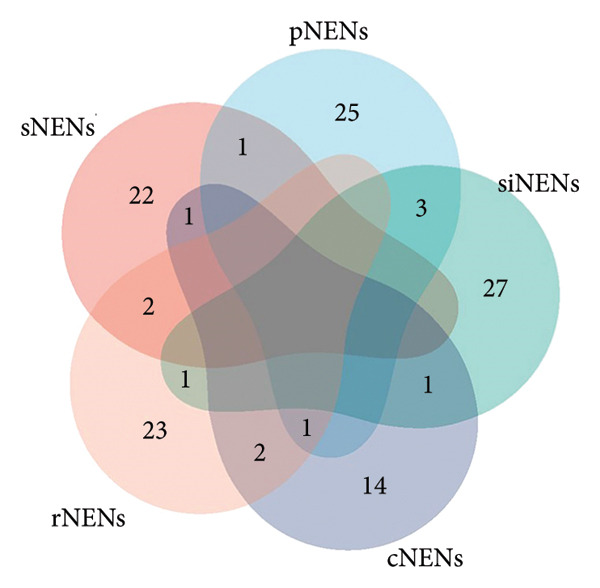
Venn diagram illustrating the number of 731 immune cells causally associated with various gastroenteropancreatic neuroendocrine neoplasms. Abbreviations: cNENs = colorectal neuroendocrine neoplasms, pNENs = pancreas neuroendocrine neoplasms, rNENs = rectum neuroendocrine neoplasms, siNENs = small intestine neuroendocrine neoplasms, and sNENs = stomach neuroendocrine neoplasms.

**Figure 5:**
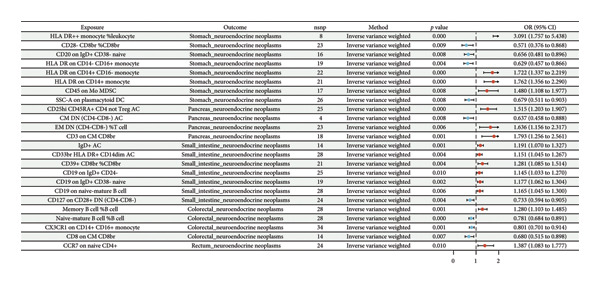
Forest plot for the causal effect of identified 731 immune cells on the risk of gastroenteropancreatic neuroendocrine neoplasms derived from inverse variance weighted (lVW, *p* < 0.01). Abbreviations: Cl = confidence interval, OR = odds ratio, and SNP = single‐nucleotide polymorphism.

Although no single inflammatory protein was linked to all tumor types, IL‐7 and IL‐8 consistently showed causal associations with both cNENs and rNENs with concordant effect directions (Figures 2 and 3). Among the immune traits, no marker was universally shared across all five subtypes, but 12 traits were associated with at least two tumor types (Table 1) (Supporting Table S6 and Figures 4 and 5), underscoring their potential biological relevance.

**Table 1:** Causal effect of immune cells on GEP‐NENs.

Immune cell exposure	Outcome	OR	95% CI	*P*
Resting Treg AC	pNENs	1.44	1.09–1.91	0.011
	siNENs	0.82	0.68–0.98	0.033

EM CD4+ %T cell (RC)	pNENs	0.72	0.52–0.99	0.048
	siNENs	1.26	1.05–1.53	0.014

IgD+ AC	pNENs	0.82	0.69–0.97	0.024
	siNENs	1.19	1.07–1.33	0.001

CD20 on IgD+ CD38− naive (MFI)	sNENs	0.67	0.48–0.90	0.008
	rNENs	0.65	0.43–0.95	0.026

CD25 on CD4+ (MFI)	sNENs	0.84	0.71–0.99	0.042
	rNENs	0.77	0.62–0.96	0.019

CD33dim HLA DR+ CD11b+ AC	sNENs	1.33	1.04–1.70	0.025
	cNENs	1.20	1.04–1.39	0.010

CM DN (CD4− CD8−) AC	sNENs	0.68	0.48–0.97	0.033
	pNENs	0.63	0.46–0.89	0.008

CD45RA on TD CD8br (MFI)	siNENs	0.73	0.55–0.99	0.048
	rNENs	1.87	1.04–3.34	0.035

CD8 on NKT (MFI)	siNENs	0.80	0.65–0.99	0.047
	cNENs	0.75	0.61–0.93	0.010

CD14+ CD16− monocyte AC	pNENs	1.22	1.00–1.48	0.045
	cNENs	1.13	1.00–1.27	0.038
	rNENs	1.33	1.05–1.67	0.018

### 3.3. Subtype‐Specific Causal Associations

#### 3.3.1. Causal Associations for sNENs

Key inflammatory proteins associated with sNENs included IL‐17A, IL‐18, IL‐2RB, and TGF‐α (Supporting Table S5 and Figure 3). Immune cell analysis highlighted strong positive associations with HLA‐DR expression on CD14^+^ monocytes, CD14^+^ CD16^−^ monocytes, and HLA‐DR^++^ monocytes (RC), whereas protective effects were observed for CD20 on IgD^+^ CD38^−^ naive B cells, CD4^−^ CD8^−^ double‐negative T cells, and plasmacytoid DC traits (Figure 5).

#### 3.3.2. Causal Associations for siNENs

The inflammatory proteins associated with siNENs included CCL20, DNER, IL‐1α, IL‐4, and uPA. Among the immune traits, IgD^+^ AC, CD19 expression on naive B‐cell subsets, and CD39^+^ CD8^+^ T cells were positively associated with siNENs, whereas CD127 expression on double‐negative T cells showed a protective effect (Supporting Table S6 and Figure 5).

#### 3.3.3. Causal Associations for cNENs

Causal associations were identified for CCL4, IL‐33, IL‐7, IL‐8, and TWEAK. On the immune side, memory and naive B‐cell subsets, CX3CR1 expression on CD14^+^ CD16^+^ monocytes, and CD8 expression on central memory T cells were implicated (Supporting Tables S5 and S6 and Figures 3 and 5).

#### 3.3.4. Causal Associations for pNENs

FGF‐23 and FLT3L are the primary inflammatory proteins associated with pNENs. For immune traits, CD25hi CD45RA^+^ CD4^+^ non‐Tregs, CD3 expression on central memory CD8^+^ T cells, and effector memory double‐negative T cells conferred an increased risk, whereas certain double‐negative T‐cell subsets showed protective associations (Supporting Table S6 and Figure 5).

#### 3.3.5. Causal Associations for rNENs

For rNENs, IL‐10RB, IL‐22RA1, IL‐7, and IL‐8 exhibited significant causal effects. Notably, CCR7 expression on naive CD4^+^ T cells was strongly associated with rNEN susceptibility (Supporting Table S6 and Figure 5).

### 3.4. Reverse MR Analysis

Supporting Table S4 shows the SNPs identified in GEP‐NENs upon exposure to inflammatory proteins and immune cells. In the reverse MR analysis, no causal relationship was observed between GEP‐NENs and the selected exposures (Supporting Tables S7 and S8). Specifically, GEP‐NENs did not significantly influence inflammation or immune cell parameters. This lack of association could be attributed to several factors, including the insufficient sample size, limited number of IVs, and the statistical power limitations. These findings emphasize the importance of carefully considering the statistical power of reverse MR analyses, especially when dealing with complex diseases such as neuroendocrine tumors. The negative results also highlight the need for further studies with larger sample sizes and more robust instruments to validate these findings.

### 3.5. Sensitivity Analyses

Sensitivity analyses, including leave‐one‐out and MR‐PRESSO, confirmed the robustness of our findings. The leave‐one‐out analysis showed no significant change in the causal estimates, indicating that our results were not driven by individual SNPs. In addition, MR‐PRESSO analysis revealed no evidence of horizontal pleiotropy, further supporting the validity of the identified causal relationships (Supporting Tables S5 and S6; Figures S1 and S2).

### 3.6. FDR Correction

After FDR correction, the findings revealed robust causal associations between genetically predicted HLA DR++ monocyte %leukocyte (OR = 3.09, 95% CI: 1.76–5.44, *p* < 0.001, FDR = 0.022), HLA DR on CD14+ CD16− monocyte (OR = 1.72, 95% CI: 1.34–2.22, *p* < 0.001, FDR = 0.010), and HLA DR on CD14+ monocyte (OR = 1.76, 95% CI: 1.36–2.29, *p* < 0.001, FDR = 0.010) and genetically predicted sNENs.

## 4. Discussion

This study systematically explored the causal relationships between inflammatory proteins, immune cell characteristics, and GEP‐NENs using a two‐sample MR analysis framework. Cross‐subtype comparisons revealed that IL‐7 and IL‐8 consistently emerged as pivotal inflammatory mediators associated with both cNENs and rNENs, highlighting their potential as early diagnostic and therapeutic targets for NENs. Although no single immune cell trait was universally shared across all five tumor subtypes, 12 traits were causally linked to at least two subtypes, mainly involving T cell subsets (e.g., resting Treg AC, effector memory CD4^+^ T cells, CD4^−^ CD8^−^ double‐negative T cells, CD45RA expression on TD CD8br T cells, and CD8 expression on NK/T cells), B‐cell parameters (such as IgD^+^ AC, CD20 expression on IgD^+^ CD38^−^ naive B cells, and CD25 expression on CD4^+^ T cells), and myeloid/monocyte populations (including CD33^dim^ HLA‐DR^+^ CD11b^+^ antigen–presenting cells and CD14^+^ CD16^−^ monocytes).

In addition to these shared associations, we identified subtype‐specific immune landscapes that highlighted the heterogeneity of GEP‐NENs. Elevated HLA‐DR expression on CD14^+^ monocytes remained significantly associated with sNENs after FDR correction, indicating a persistent proinflammatory state that may facilitate tumor proliferation and immune dysregulation [31–33].

We also observed context‐dependent effects of lymphocyte traits: resting Treg AC increased the risk of pNENs but was protective against siNENs, whereas effector memory CD4^+^ T cells and IgD^+^ B cells displayed opposite associations across subtypes [34, 35]. Other immune parameters, such as CD20 expression on IgD^+^ CD38^−^ naive B cells and CD25 on CD4^+^ T cells, conferred risks in sNENs and rNENs, and CD33^dim^ HLA‐DR^+^ CD11b^+^ myeloid cells were implicated in both sNENs and cNENs. Conversely, protective effects were observed for CD4^−^ CD8^−^ double‐negative T cells in sNENs and pNENs, suggesting an antitumor function [36]. In contrast, CD127 expression on CD4^+^ Tregs and CD14^+^ CD16^−^ monocytes consistently emerged as a risk factor across pNENs, cNENs, and rNENs, indicating their central role in disease pathogenesis [37–39].

Reverse MR analyses did not reveal significant feedback effects from GEP‐NENs to systemic inflammation, reinforcing a predominantly unidirectional causal role of inflammatory and immune traits in tumor development. This supports the notion that inflammation may function as a driving force rather than a secondary consequence of tumorigenesis and that local immune dysregulation within the tumor microenvironment, rather than systemic inflammation, may critically shape disease progression.

Several cytokines identified in this study have well‐established roles in tumor immunology. For example, IL‐18 enhances tumor‐infiltrating lymphocyte activation and promotes antitumor responses [40], IL‐1α exerts dual roles by driving cachexia [41] and priming antitumor immunity [42, 43], IL‐4 promotes epithelial tumor growth and immune evasion [44–46]; and both IL‐33 and IL‐7 exhibit pleiotropic effects on tumor immunity [47–50]. IL‐10RB, causally linked to GEP‐NENs in our analysis, is known to mediate dichotomous immunoregulatory functions [47], highlighting its potential as an immunotherapeutic modulator. Additional chemokines and growth factors, such as TGF‐α, CCL4, CCL20, IL‐17A, IL‐2RB, and FGF‐23, further underscore the multifaceted interplay between inflammation and tumor biology through pathways ranging from proliferation and angiogenesis to immune cell recruitment [51–56]. Other factors, including FLT3L/FLT3 [57], DNER [58–60], uPA [60], IL‐8 [61], TWEAK [62], and IL‐22R/IL‐22RA1 [63], may contribute to immune dysregulation or metastatic potential in GEP‐NENs and warrant further mechanistic investigation.

Taken together, these findings indicate that only a subset of inflammatory and immune markers, particularly IL‐7, IL‐8, HLA‐DR^+^ monocyte subsets, and lymphocyte traits, are likely to be clinically actionable. Their strong genetic associations and detectability in routine assays support their prioritization for early risk prediction and stratified management in patients with GEP‐NEN, whereas other factors may represent secondary or context‐dependent associations that require further validation.

From a clinical perspective, the identified markers, such as IL‐7, IL‐8, and HLA‐DR‐expressing monocyte subsets, are measurable in peripheral blood using routine assays, which supports their integration into risk prediction models. Incorporating these immune signatures into diagnostic panels may enable the earlier detection of individuals at an elevated risk of GEP‐NENs, while subtype‐specific associations (e.g., resting Treg AC in pNENs vs. siNENs) provide opportunities for stratified management strategies. Patients with high‐risk immune profiles should be prioritized for closer surveillance or early intervention, whereas protective traits may guide de‐escalated monitoring. These markers can also be combined with existing radiological and pathological indicators to construct composite prognostic scores, thereby enhancing individualized treatment planning and clinical decision‐making in GEP‐NENs.

This study had some limitations. First, two‐sample MR provides statistical evidence of causality but does not elucidate the molecular mechanisms, and experimental validation is required. Second, all GWAS datasets used were derived from individuals of primarily European ancestry, which may limit the generalizability of our findings to other populations. Future multiancestry studies are essential to assess the global relevance and ethnic specificity of these immunological markers. Collectively, our findings provide a rationale for prioritizing genetically anchored inflammatory and immune traits as candidate biomarkers or therapeutic targets in GEP‐NENs, while emphasizing the need for functional validation in population‐diverse cohorts.

## 5. Conclusions

In conclusion, our findings suggest that inflammatory proteins and immune‐related traits may play important roles in the development of GEP‐NEN. By revealing subtype‐specific immune patterns and potential immune‐related biomarkers, this study provides new perspectives on the immunological underpinnings of these tumors and may inform future therapeutic strategies for their treatment. Nevertheless, further experimental and clinical studies are warranted to validate these observations and evaluate their translational potential in precision oncology research.

## Ethics Statement

The authors have nothing to report.

## Consent

The authors have nothing to report.

## Disclosure

All authors agreed on the journal to which the article would be submitted, gave final approval of the version to be published, and agreed to be accountable for all aspects of the work.

## Conflicts of Interest

The authors declare no conflicts of interest.

## Author Contributions

Huimin Guo and Yu Li contributed equally to this work. Huimin Guo drafted the manuscript. Yu Li designed the study and revised the manuscript. Bowei Liu analyzed the data. Songtao Liu was responsible for giving constructive suggestions for revising the article. All authors contributed to data analysis and drafting or revising the article (and its Supporting Information).

## Funding

No funding was received for this study.

## Supporting Information

Additional supporting information can be found online in the Supporting Information section.

## Supporting information


**Supporting Information 1** Supporting Figure S1: MR analysis of 91 inflammatory proteins and the risk of five GEP‐NEN subtypes. Four plots are presented (from left to right): Forest plot, showing SNP‐specific effect estimates (Wald ratio) with the IVW estimate indicated by a red line; a rightward shift suggests that genetically predicted inflammatory protein levels increase GEP‐NEN risk. Funnel plot, displaying SNP effects against their standard errors to evaluate symmetry around the IVW estimate; symmetry supports the absence of directional pleiotropy and robust MR results. Leave‐one‐out analysis, assessing the influence of individual SNPs on the overall IVW estimate; stability of the “All” estimate indicates robustness, whereas large deviations upon excluding one SNP suggest outlier effects. Scatter plot, plotting SNP effects on inflammatory proteins (*x*‐axis) against GEP‐NEN risk (*y*‐axis), with fitted regression lines from multiple MR methods; an upward slope indicates a positive causal relationship. Together, these complementary analyses confirm the consistency and robustness of the MR findings.

**Supporting Information 2** Supporting Figure S2: MR analysis of 731 immune cell traits and the risk of five GEP‐NEN subtypes. Four types of plots are shown (from left to right): Forest plot, displaying the SNP‐specific effect estimates (Wald ratio) with the IVW estimate indicated by a red line; a rightward shift of the IVW line suggests that genetically predicted immune cell traits increase GEP‐NEN risk. Funnel plot, plotting SNP effects against their standard errors to evaluate symmetry around the IVW estimate, where symmetry supports the absence of directional pleiotropy and robust MR results. Leave‐one‐out analysis, assessing the influence of individual SNPs on the overall IVW estimate; the stability of the “All” estimate indicates robustness, while large deviations upon excluding one SNP suggest potential outlier effects. Scatter plot, showing SNP effects on immune cell traits (*x*‐axis) versus GEP‐NEN risk (*y*‐axis), with fitted regression lines from multiple MR methods; an upward trend indicates that increased immune cell traits are associated with higher GEP‐NEN risk. Together, these complementary analyses demonstrate the consistency and robustness of the MR findings.

**Supporting Information 3** Supporting Table S1: Details of the data sources used in this study. Supporting Table S2: IVs used in MR analysis of the association between 91 inflammatory proteins and GEP‐NENs. Supporting Table S3: IVs used in MR analysis of the association between 731 immune cells and GEP‐NENs. Supporting Table S4: SNPs of GEP‐NENs used as exposure in reverse MR. Supporting Table S5: MR approaches were employed to investigate the association between 91 inflammatory and immune‐related proteins and GEP‐NENs. Supporting Table S6: MR approaches were employed to investigate the association between 731 immune cells and GEP‐NENs. Supporting Table S7: The reverse MR results between 91 inflammatory proteins and GEP‐NENs. Supporting Table S8: The reverse MR results between 731 immune cells and GEP‐NENs.

## Data Availability

All data utilized in this investigation were sourced from the summary statistics of genomewide association studies that have been made publicly available by various genetic consortia. Specifically, the data were retrieved from OpenGWAS (https://gwas.mrcieu.ac.uk/) and the Finnish database, which comprises the R10 version of FinnGen (https://www.finngen.fi/en/access_results). The data supporting the findings of this study are available within the article (and its Supporting Information) and are available upon request from the corresponding authors.
