# Supplementary material for: Associations Between Gastroenteropancreatic Neuroendocrine Neoplasms and Inflammatory Factors: Insights From a Two‐Sample Mendelian Randomization Analysis
Source: Can J Gastroenterol Hepatol. 2025 Dec 26;2025:2591387. doi: 10.1155/cjgh/2591387 (PMC12741578; doi:10.1155/cjgh/2591387)
Supplement: Supplementary file 1 — Supporting Information 1 Supporting Figure S1: MR analysis of 91 inflammatory proteins and the risk of five GEP‐NEN subtypes. Four plots are presented (from left to right): Forest plot, showing SNP‐specific effect estimates (Wald ratio) with the IVW estimate indicated by a red line; a rightward shift suggests that genetically predicted inflammatory protein levels increase GEP‐NEN risk. Funnel plot, displaying SNP effects against their standard errors to evaluate symmetry around the IVW estimate; symmetry supports the absence of directional pleiotropy and robust MR results. Leave‐one‐out analysis, assessing the influence of individual SNPs on the overall IVW estimate; stability of the “All” estimate indicates robustness, whereas large deviations upon excluding one SNP suggest outlier effects. Scatter plot, plotting SNP effects on inflammatory proteins (x‐axis) against GEP‐NEN risk (y‐axis), with fitted regression lines from multiple MR methods; an upward slope indicates a positive causal relationship. Together, these complementary analyses confirm the consistency and robustness of the MR findings. [file CJGH-2025-2591387-s002.pdf]

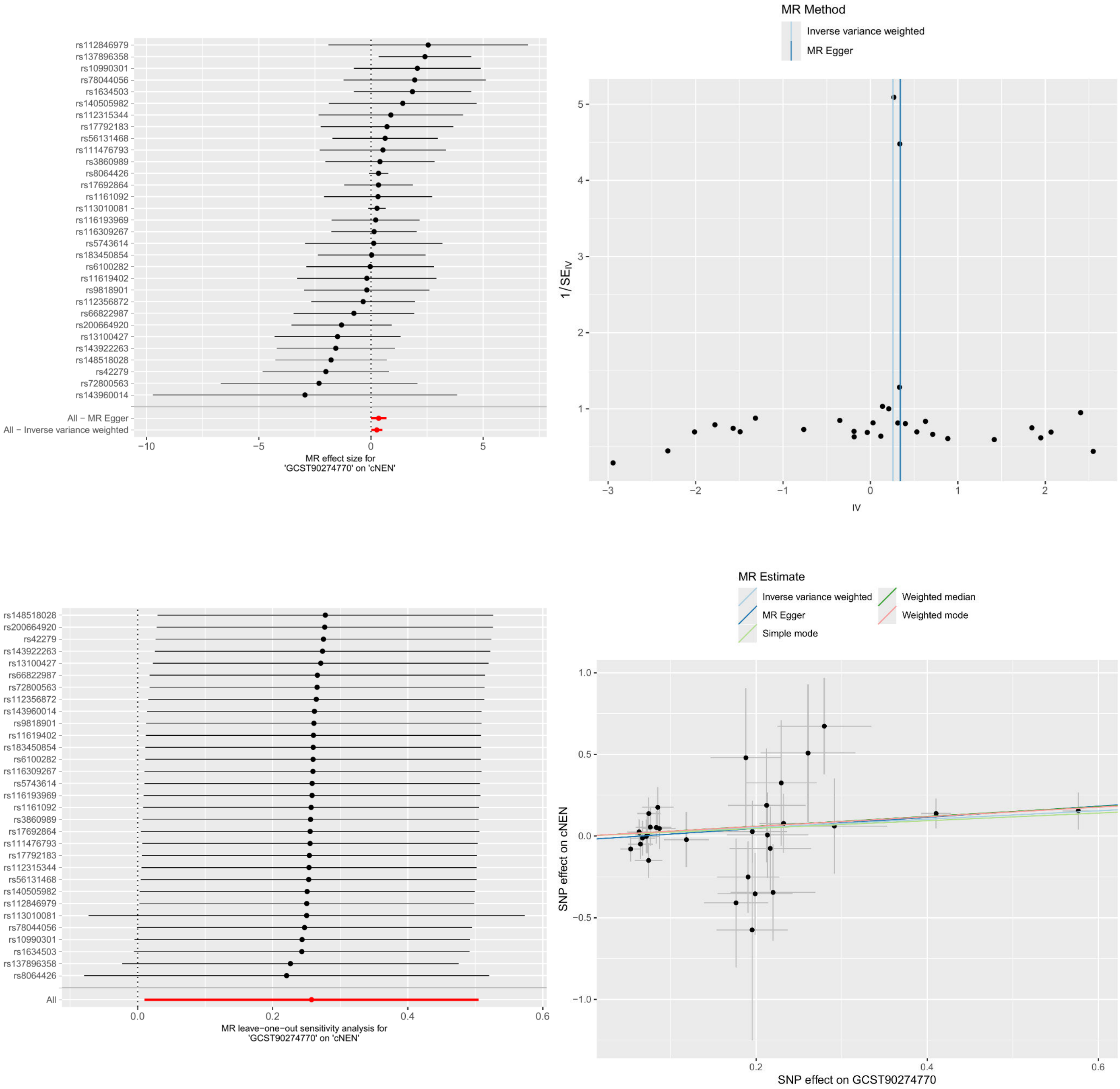

rs112846979
rs137896358
rs10990301
rs78044056
rs1634503
rs140505982
rs112315344
rs17792183
rs56131468
rs111476793
rs3860989
rs8064426
rs17692864
rs1161092
rs113010081
rs116193969
rs116309267
rs5743614
rs183450854
rs6100282
rs11619402
rs9818901
rs112356872
rs66822987
rs200664920
rs13100427
rs143922263
rs148518028
rs42279
rs72800563
rs143960014
All − MR Egger
All − Inverse variance weighted
−10
−5
0
5
MR effect size for
'GCST90274770' on 'cNEN'
MR Method
Inverse variance weighted
MR Egger
1/SE_IV
5
4
3
2
1
−3
−2
−1
0
1
2
IV
rs148518028
rs200664920
rs42279
rs143922263
rs13100427
rs66822987
rs72800563
rs112356872
rs143960014
rs9818901
rs11619402
rs183450854
rs6100282
rs116309267
rs5743614
rs116193969
rs1161092
rs3860989
rs17692864
rs111476793
rs17792183
rs112315344
rs56131468
rs140505982
rs112846979
rs113010081
rs78044056
rs10990301
rs1634503
rs137896358
rs8064426
All
0.0
0.2
0.4
0.6
MR leave−one−out sensitivity analysis for
'GCST90274770' on 'cNEN'
MR Estimate
Inverse variance weighted
MR Egger
Simple mode
Weighted median
Weighted mode
1.0
0.5
0.0
−0.5
−1.0
SNP effect on cNEN
0.2
0.4
0.6
SNP effect on GCST90274770

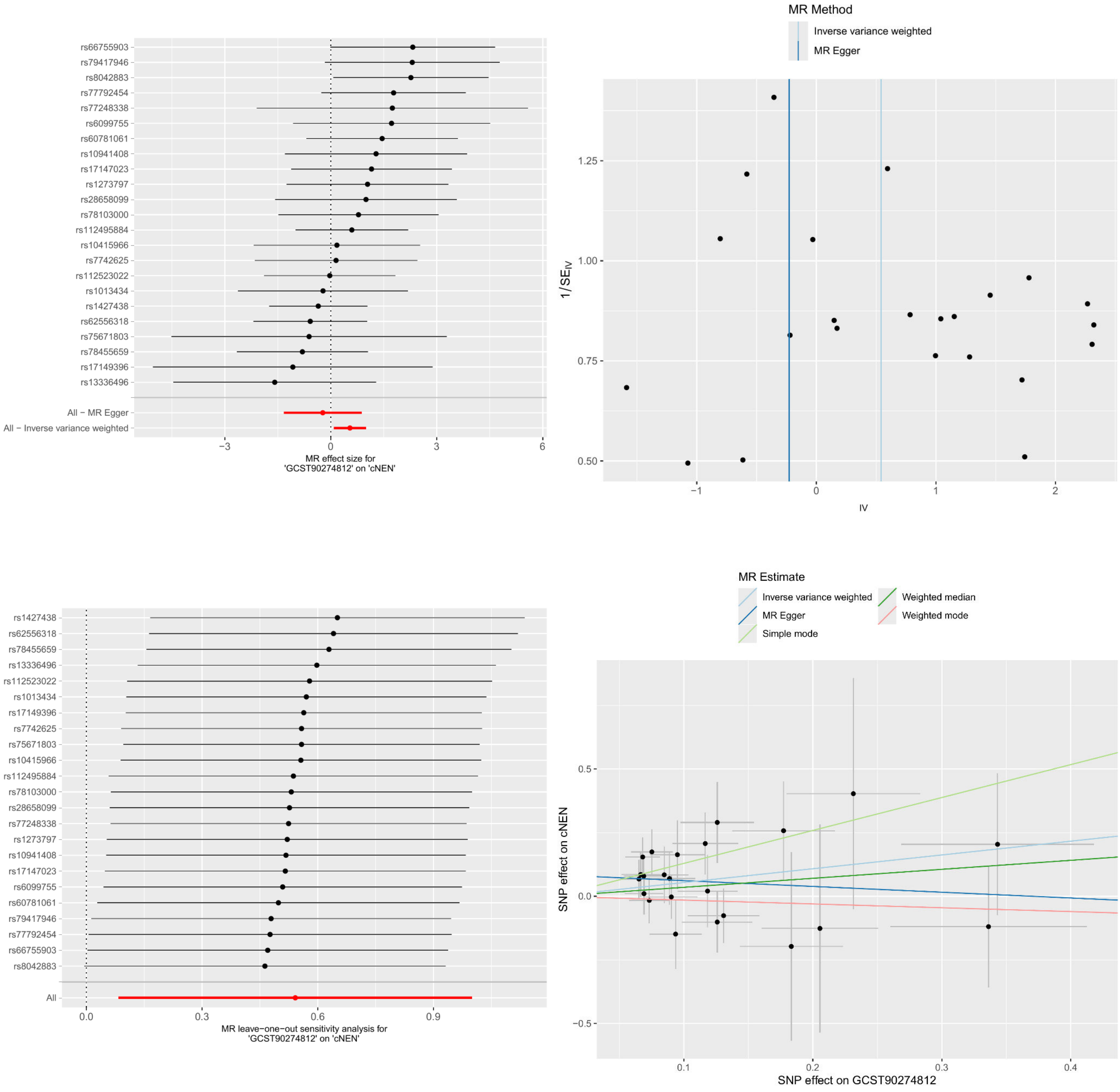
rs66755903
rs79417946
rs8042883
rs77792454
rs77248338
rs6099755
rs60781061
rs10941408
rs17147023
rs1273797
rs28658099
rs78103000
rs112495884
rs10415966
rs7742625
rs112523022
rs1013434
rs1427438
rs62556318
rs75671803
rs78455659
rs17149396
rs13336496
All − MR Egger
All − Inverse variance weighted
−3
0
3
6
MR effect size for
'GCST90274812' on 'cNEN'
MR Method
Inverse variance weighted
MR Egger
1/SE$_{IV}$
1.25
1.00
0.75
0.50
−1
0
1
2
IV
rs1427438
rs62556318
rs78455659
rs13336496
rs112523022
rs1013434
rs17149396
rs7742625
rs75671803
rs10415966
rs112495884
rs78103000
rs28658099
rs77248338
rs1273797
rs10941408
rs17147023
rs6099755
rs60781061
rs79417946
rs77792454
rs66755903
rs8042883
All
0.0
0.3
0.6
0.9
MR leave−one−out sensitivity analysis for
'GCST90274812' on 'cNEN'
MR Estimate
Inverse variance weighted
MR Egger
Simple mode
Weighted median
Weighted mode
SNP effect on cNEN
0.5
0.0
−0.5
0.1
0.2
0.3
0.4
SNP effect on GCST90274812

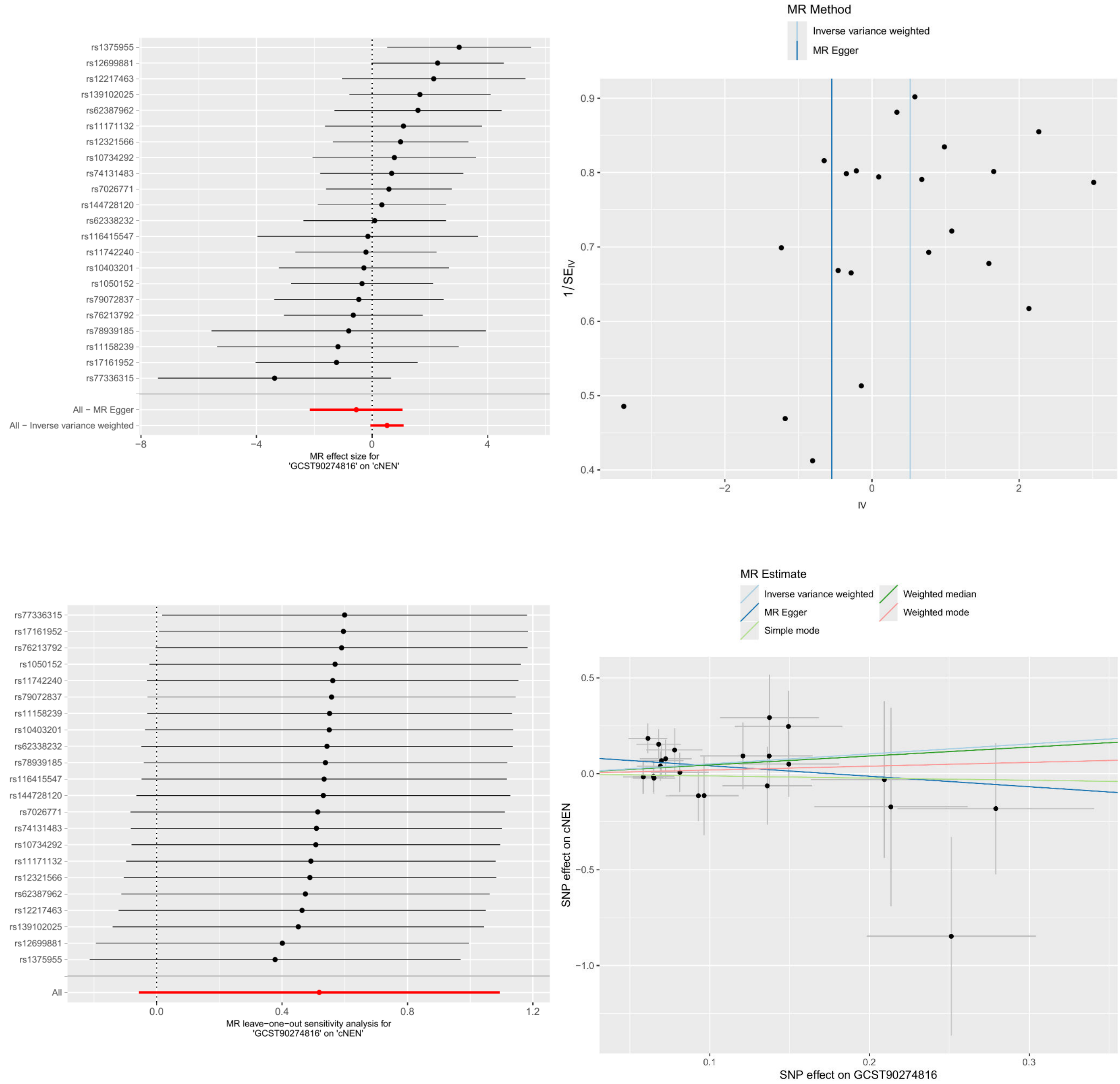
rs1375955
rs12699881
rs12217463
rs139102025
rs62387962
rs11171132
rs12321566
rs10734292
rs74131483
rs7026771
rs144728120
rs62338232
rs116415547
rs11742240
rs10403201
rs1050152
rs79072837
rs76213792
rs78939185
rs11158239
rs17161952
rs77336315
All − MR Egger
All − Inverse variance weighted
−8
−4
0
4
MR effect size for
'GCST90274816' on 'cNEN'
MR Method
Inverse variance weighted
MR Egger
1/SE_IV
0.9
0.8
0.7
0.6
0.5
0.4
−2
0
2
IV
rs77336315
rs17161952
rs76213792
rs1050152
rs11742240
rs79072837
rs11158239
rs10403201
rs62338232
rs78939185
rs116415547
rs144728120
rs7026771
rs74131483
rs10734292
rs11171132
rs12321566
rs62387962
rs12217463
rs139102025
rs12699881
rs1375955
All
0.0
0.4
0.8
1.2
MR leave−one−out sensitivity analysis for
'GCST90274816' on 'cNEN'
MR Estimate
Inverse variance weighted
MR Egger
Simple mode
Weighted median
Weighted mode
0.5
0.0
−0.5
−1.0
SNP effect on cNEN
0.1
0.2
0.3
SNP effect on GCST90274816

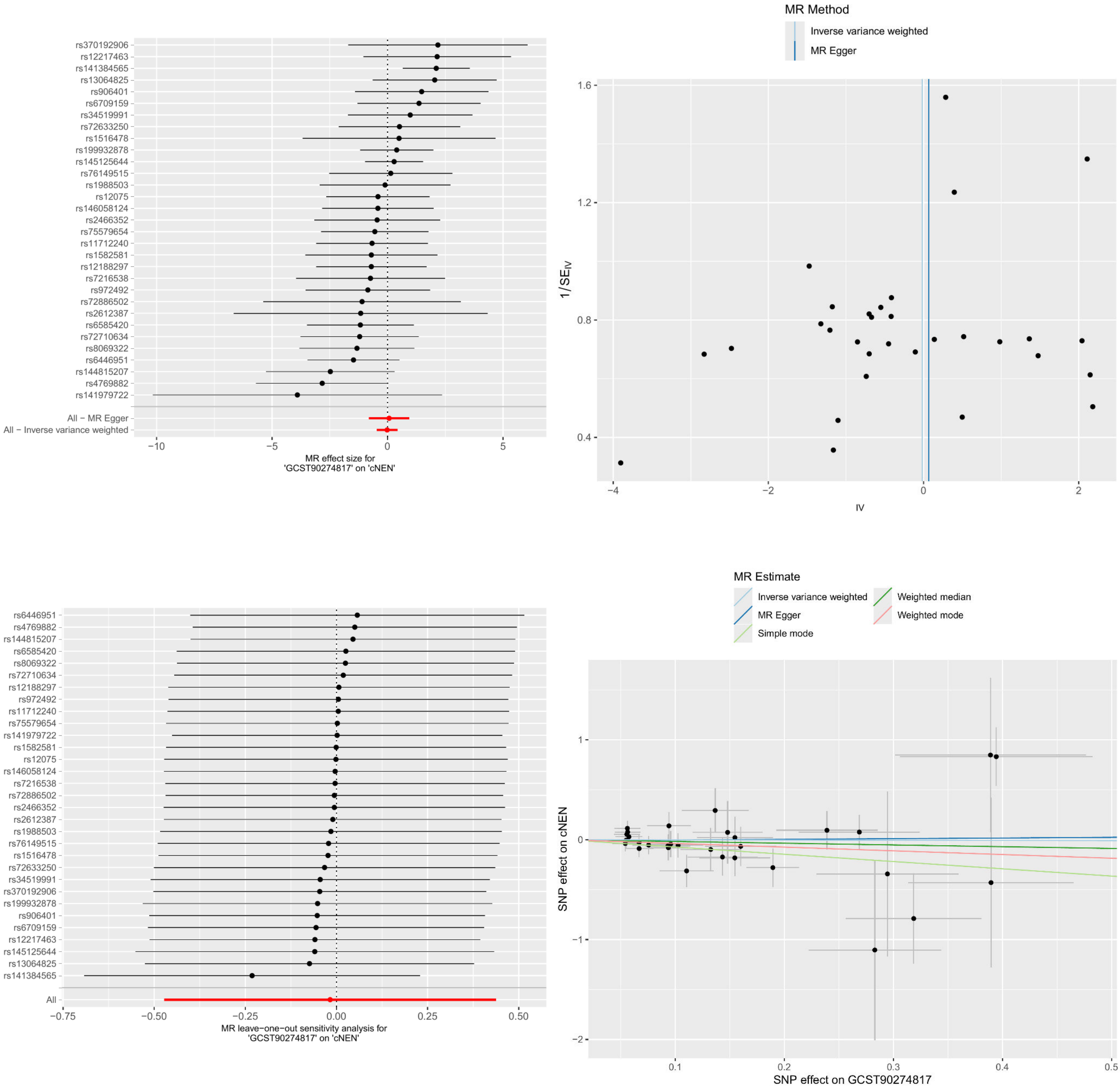
rs370192906
rs12217463
rs141384565
rs13064825
rs906401
rs6709159
rs34519991
rs72633250
rs1516478
rs199932878
rs145125644
rs76149515
rs1988503
rs12075
rs146058124
rs2466352
rs75579654
rs11712240
rs1582581
rs12188297
rs7216538
rs972492
rs72886502
rs2612387
rs6585420
rs72710634
rs8069322
rs6446951
rs144815207
rs4769882
rs141979722
All − MR Egger
All − Inverse variance weighted
−10
−5
0
5
MR effect size for
'GCST90274817' on 'cNEN'
MR Method
Inverse variance weighted
MR Egger
1.6
1.2
0.8
0.4
1/SE$_{IV}$
−4
−2
0
2
IV
rs6446951
rs4769882
rs144815207
rs6585420
rs8069322
rs72710634
rs12188297
rs972492
rs11712240
rs75579654
rs141979722
rs1582581
rs12075
rs146058124
rs7216538
rs72886502
rs2466352
rs2612387
rs1988503
rs76149515
rs1516478
rs72633250
rs34519991
rs370192906
rs199932878
rs906401
rs6709159
rs12217463
rs145125644
rs13064825
rs141384565
All
−0.75
−0.50
−0.25
0.00
0.25
0.50
MR leave-one-out sensitivity analysis for
'GCST90274817' on 'cNEN'
MR Estimate
Inverse variance weighted
MR Egger
Simple mode
Weighted median
Weighted mode
1
0
−1
−2
SNP effect on cNEN
0.1
0.2
0.3
0.4
0.5
SNP effect on GCST90274817

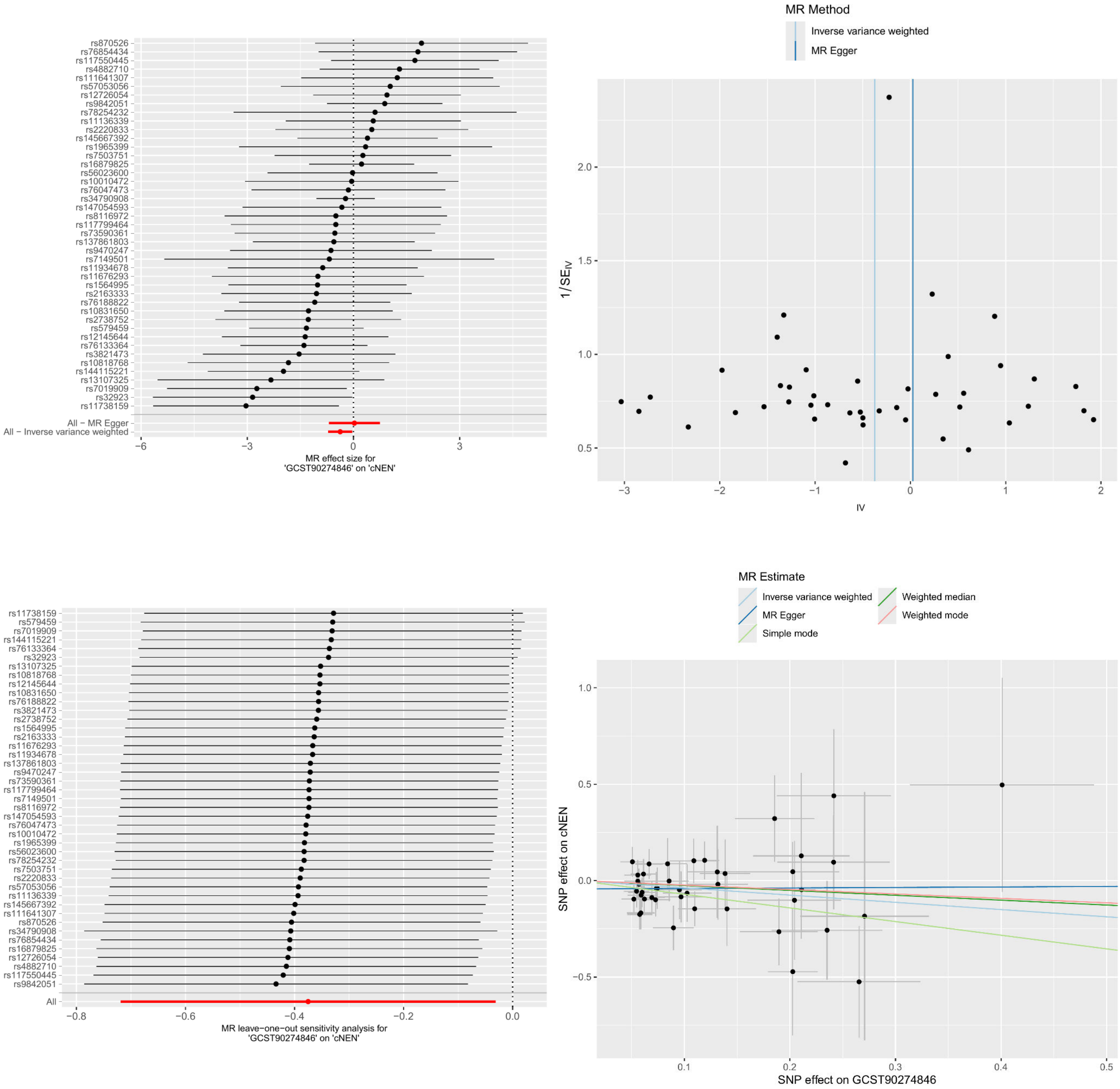
rs870526
rs76854434
rs117550445
rs4882710
rs111641307
rs57053056
rs12726054
rs9842051
rs78254232
rs11136339
rs2220833
rs145667392
rs1965399
rs7503751
rs16879825
rs56023600
rs10010472
rs76047473
rs34790908
rs147054593
rs8116972
rs117799464
rs73590361
rs137861803
rs9470247
rs7149501
rs11934678
rs11676293
rs1564995
rs2163333
rs76188822
rs10831650
rs2738752
rs579459
rs12145644
rs76133364
rs3821473
rs10818768
rs144115221
rs13107325
rs7019909
rs32923
rs11738159
All − MR Egger
All − Inverse variance weighted
−6
−3
0
3
MR effect size for
'GCST90274846' on 'cNEN'
MR Method
Inverse variance weighted
MR Egger
1/SE$_{IV}$
0.5
1.0
1.5
2.0
−3
−2
−1
0
1
2
IV
rs11738159
rs579459
rs7019909
rs144115221
rs76133364
rs32923
rs13107325
rs10818768
rs12145644
rs10831650
rs76188822
rs3821473
rs2738752
rs1564995
rs2163333
rs11676293
rs11934678
rs137861803
rs9470247
rs73590361
rs117799464
rs7149501
rs8116972
rs147054593
rs76047473
rs10010472
rs1965399
rs56023600
rs78254232
rs7503751
rs2220833
rs57053056
rs11136339
rs145667392
rs111641307
rs870526
rs34790908
rs76854434
rs16879825
rs12726054
rs4882710
rs117550445
rs9842051
All
−0.8
−0.6
−0.4
−0.2
0.0
MR leave−one−out sensitivity analysis for
'GCST90274846' on 'cNEN'
MR Estimate
Inverse variance weighted
MR Egger
Simple mode
Weighted median
Weighted mode
SNP effect on cNEN
−0.5
0.0
0.5
1.0
0.1
0.2
0.3
0.4
0.5
SNP effect on GCST90274846

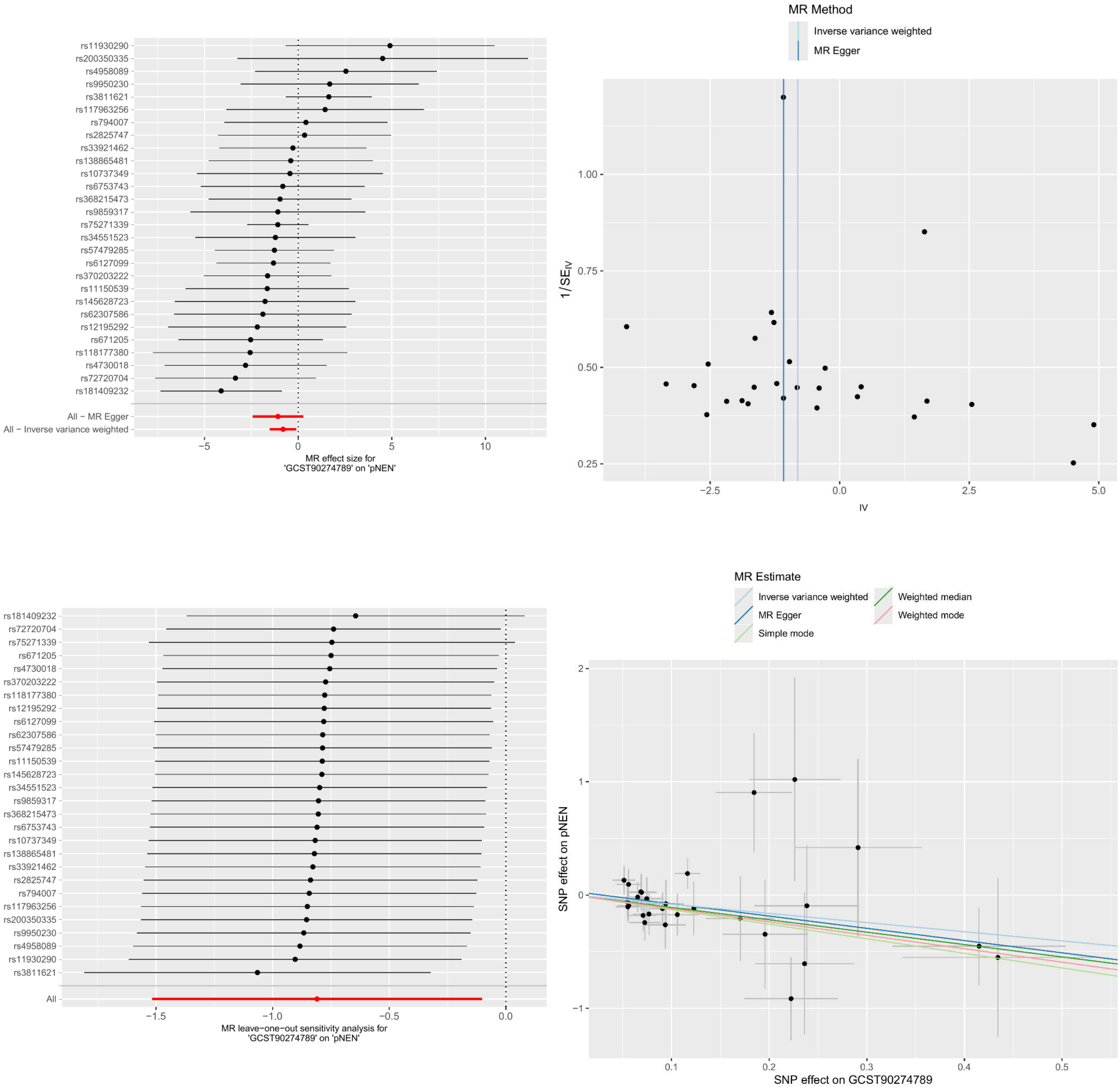
rs11930290
rs200350335
rs4958089
rs9950230
rs3811621
rs117963256
rs794007
rs2825747
rs33921462
rs138865481
rs10737349
rs6753743
rs368215473
rs9859317
rs75271339
rs34551523
rs57479285
rs6127099
rs370203222
rs11150539
rs145628723
rs62307586
rs12195292
rs671205
rs118177380
rs4730018
rs72720704
rs181409232
All − MR Egger
All − Inverse variance weighted
−5
0
5
10
MR effect size for
'GCST90274789' on 'pNEN'
MR Method
Inverse variance weighted
MR Egger
1/SE$_{IV}$
1.00
0.75
0.50
0.25
−2.5
0.0
2.5
5.0
IV
rs181409232
rs72720704
rs75271339
rs671205
rs4730018
rs370203222
rs118177380
rs12195292
rs6127099
rs62307586
rs57479285
rs11150539
rs145628723
rs34551523
rs9859317
rs368215473
rs6753743
rs10737349
rs138865481
rs33921462
rs2825747
rs794007
rs117963256
rs200350335
rs9950230
rs4958089
rs11930290
rs3811621
All
−1.5
−1.0
−0.5
0.0
MR leave−one−out sensitivity analysis for
'GCST90274789' on 'pNEN'
MR Estimate
Inverse variance weighted
MR Egger
Simple mode
Weighted median
Weighted mode
SNP effect on pNEN
2
1
0
−1
0.1
0.2
0.3
0.4
0.5
SNP effect on GCST90274789

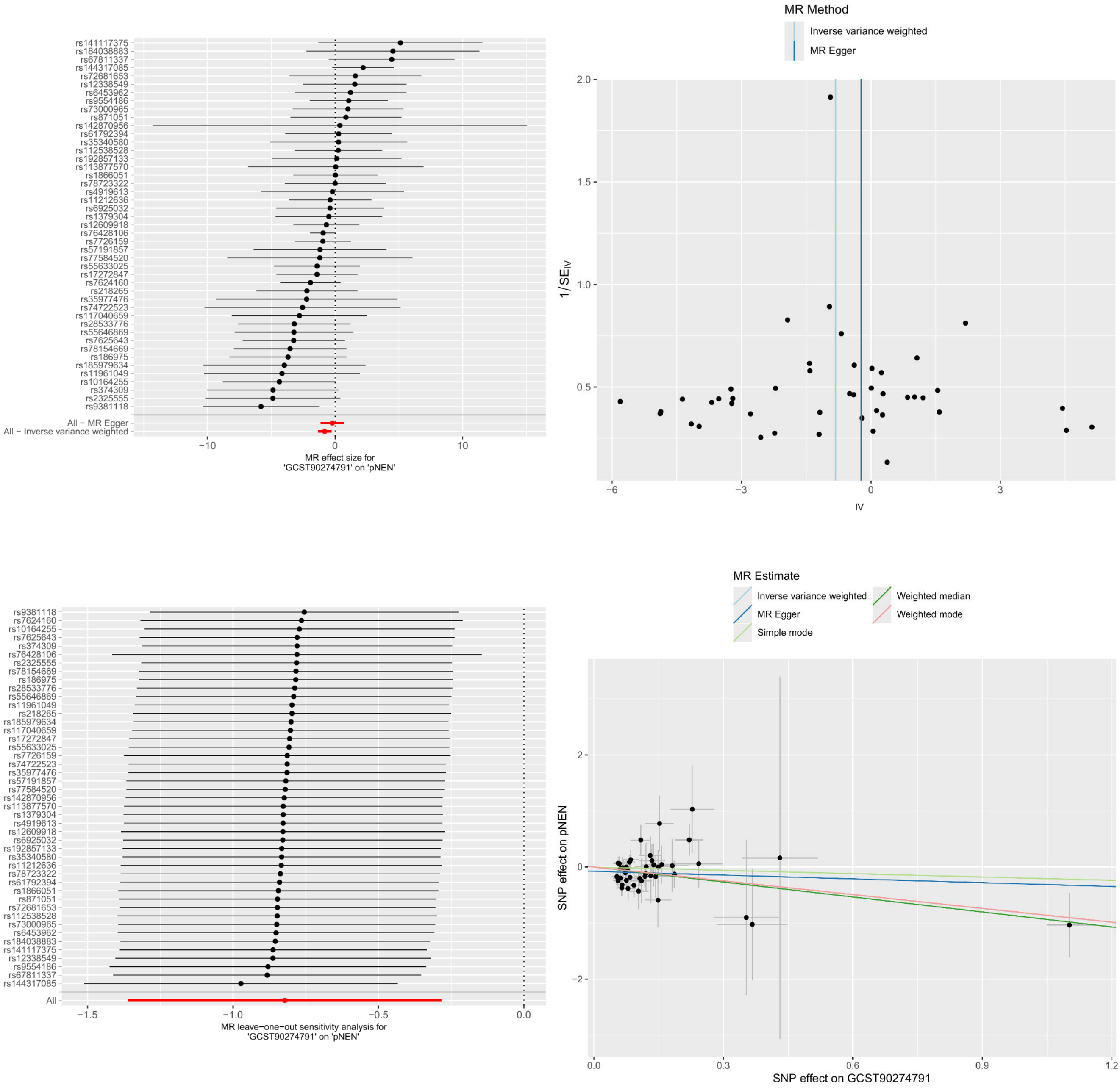
rs141117375
rs184038883
rs67811337
rs144317085
rs72681653
rs12338549
rs6453962
rs9554186
rs73000965
rs871051
rs142870956
rs61792394
rs35340580
rs112538528
rs192857133
rs113877570
rs1866051
rs78723322
rs4919613
rs11212636
rs6925032
rs1379304
rs12609918
rs76428106
rs7726159
rs57191857
rs77584520
rs55633025
rs17272847
rs7624160
rs218265
rs35977476
rs74722523
rs117040659
rs28533776
rs55646869
rs7625643
rs78154669
rs186975
rs185979634
rs11961049
rs10164255
rs374309
rs2325555
rs9381118
All − MR Egger
All − Inverse variance weighted
−10
0
10
MR effect size for
'GCST90274791' on 'pNEN'
MR Method
Inverse variance weighted
MR Egger
2.0
1.5
1.0
0.5
$1/SE_{IV}$
−6
−3
0
3
IV
rs9381118
rs7624160
rs10164255
rs7625643
rs374309
rs76428106
rs2325555
rs78154669
rs186975
rs28533776
rs55646869
rs11961049
rs218265
rs185979634
rs117040659
rs17272847
rs55633025
rs7726159
rs74722523
rs35977476
rs57191857
rs77584520
rs142870956
rs113877570
rs1379304
rs4919613
rs12609918
rs6925032
rs192857133
rs35340580
rs11212636
rs78723322
rs61792394
rs1866051
rs871051
rs72681653
rs112538528
rs73000965
rs6453962
rs184038883
rs141117375
rs12338549
rs9554186
rs67811337
rs144317085
All
−1.5
−1.0
−0.5
0.0
MR leave−one−out sensitivity analysis for
'GCST90274791' on 'pNEN'
MR Estimate
Inverse variance weighted
MR Egger
Simple mode
Weighted median
Weighted mode
2
0
−2
SNP effect on pNEN
0.0
0.3
0.6
0.9
1.2
SNP effect on GCST90274791

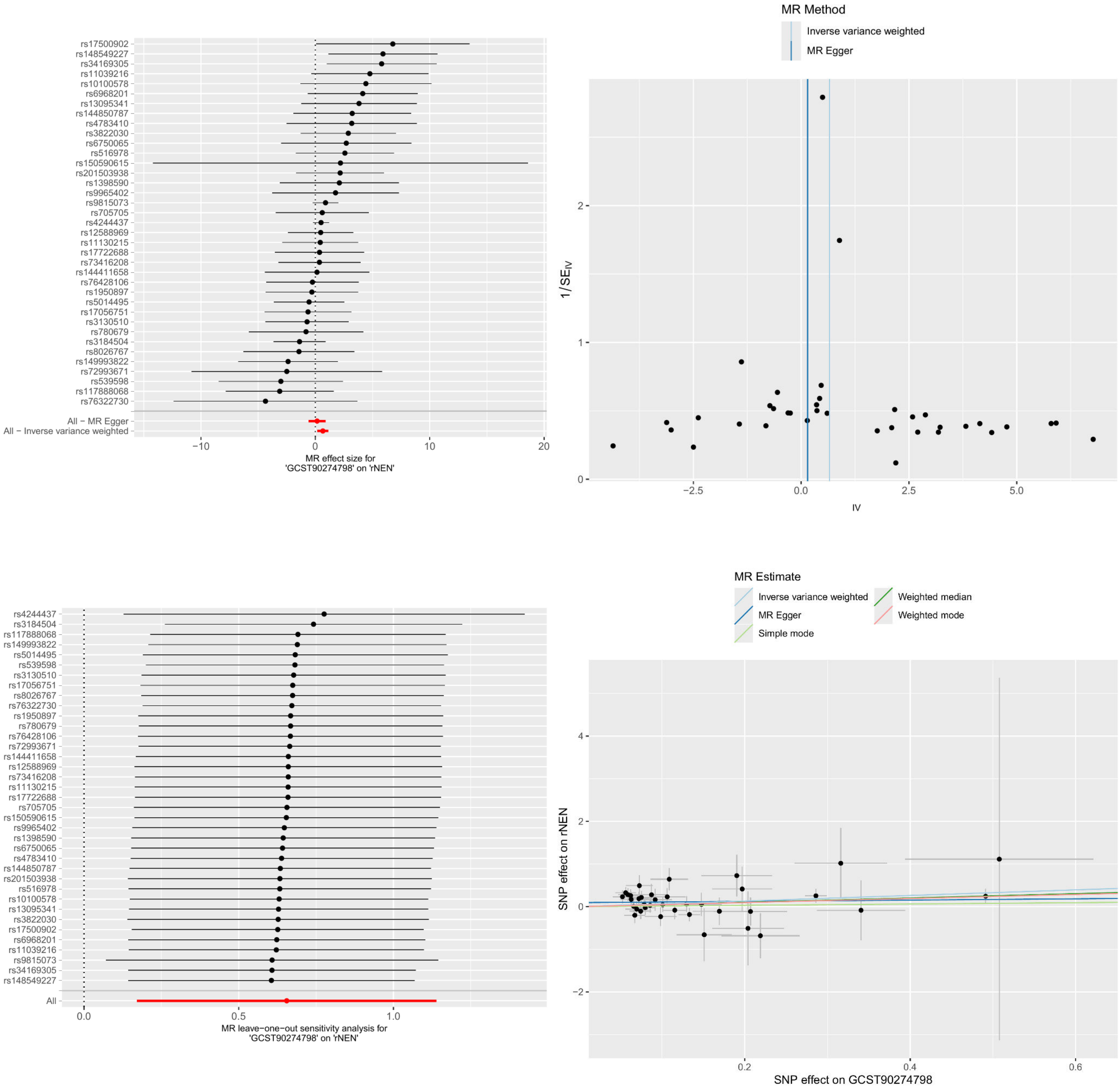
rs17500902
rs148549227
rs34169305
rs11039216
rs10100578
rs6968201
rs13095341
rs144850787
rs4783410
rs3822030
rs6750065
rs516978
rs150590615
rs201503938
rs1398590
rs9965402
rs9815073
rs705705
rs4244437
rs12588969
rs11130215
rs17722688
rs73416208
rs144411658
rs76428106
rs1950897
rs5014495
rs17056751
rs3130510
rs780679
rs3184504
rs8026767
rs149993822
rs72993671
rs539598
rs117888068
rs76322730
All − MR Egger
All − Inverse variance weighted
−10
0
10
20
MR effect size for
'GCST90274798' on 'rNEN'
MR Method
Inverse variance weighted
MR Egger
1/SE$_{IV}$
0
1
2
−2.5
0.0
2.5
5.0
IV
rs4244437
rs3184504
rs117888068
rs149993822
rs5014495
rs539598
rs3130510
rs17056751
rs8026767
rs76322730
rs1950897
rs780679
rs76428106
rs72993671
rs144411658
rs12588969
rs73416208
rs11130215
rs17722688
rs705705
rs150590615
rs9965402
rs1398590
rs6750065
rs4783410
rs144850787
rs201503938
rs516978
rs10100578
rs13095341
rs3822030
rs17500902
rs6968201
rs11039216
rs9815073
rs34169305
rs148549227
All
0.0
0.5
1.0
MR leave−one−out sensitivity analysis for
'GCST90274798' on 'rNEN'
MR Estimate
Inverse variance weighted
MR Egger
Simple mode
Weighted median
Weighted mode
SNP effect on rNEN
−2
0
2
4
0.2
0.4
0.6
SNP effect on GCST90274798

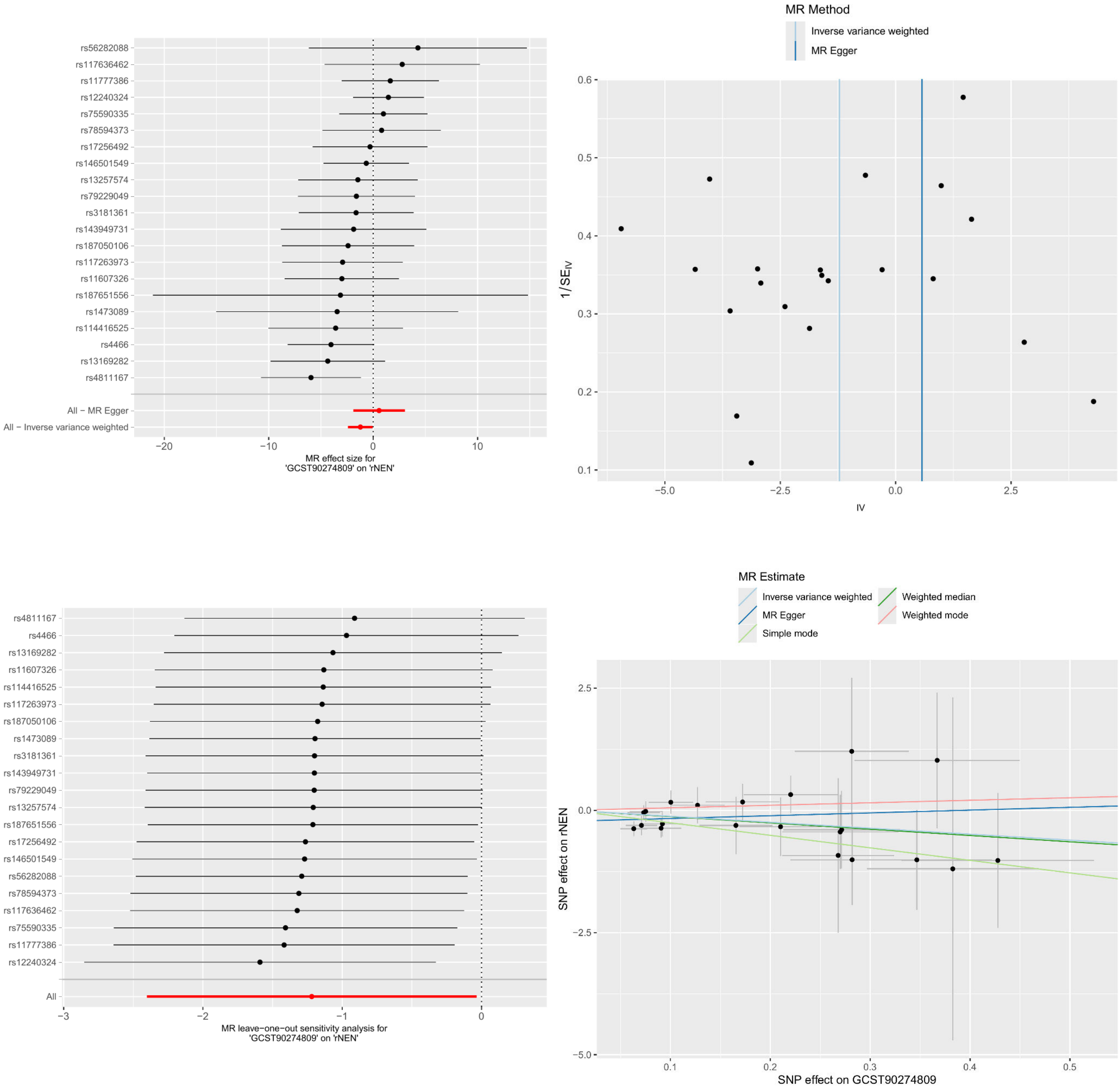
rs56282088
rs117636462
rs11777386
rs12240324
rs75590335
rs78594373
rs17256492
rs146501549
rs13257574
rs79229049
rs3181361
rs143949731
rs187050106
rs117263973
rs11607326
rs187651556
rs1473089
rs114416525
rs4466
rs13169282
rs4811167
All − MR Egger
All − Inverse variance weighted
−20
−10
0
10
MR effect size for
'GCST90274809' on 'rNEN'
MR Method
Inverse variance weighted
MR Egger
0.6
0.5
0.4
0.3
0.2
0.1
1/SE$_{IV}$
−5.0
−2.5
0.0
2.5
IV
rs4811167
rs4466
rs13169282
rs11607326
rs114416525
rs117263973
rs187050106
rs1473089
rs3181361
rs143949731
rs79229049
rs13257574
rs187651556
rs17256492
rs146501549
rs56282088
rs78594373
rs117636462
rs75590335
rs11777386
rs12240324
All
−3
−2
−1
0
MR leave−one−out sensitivity analysis for
'GCST90274809' on 'rNEN'
MR Estimate
Inverse variance weighted
MR Egger
Simple mode
Weighted median
Weighted mode
2.5
0.0
−2.5
−5.0
SNP effect on rNEN
0.1
0.2
0.3
0.4
0.5
SNP effect on GCST90274809

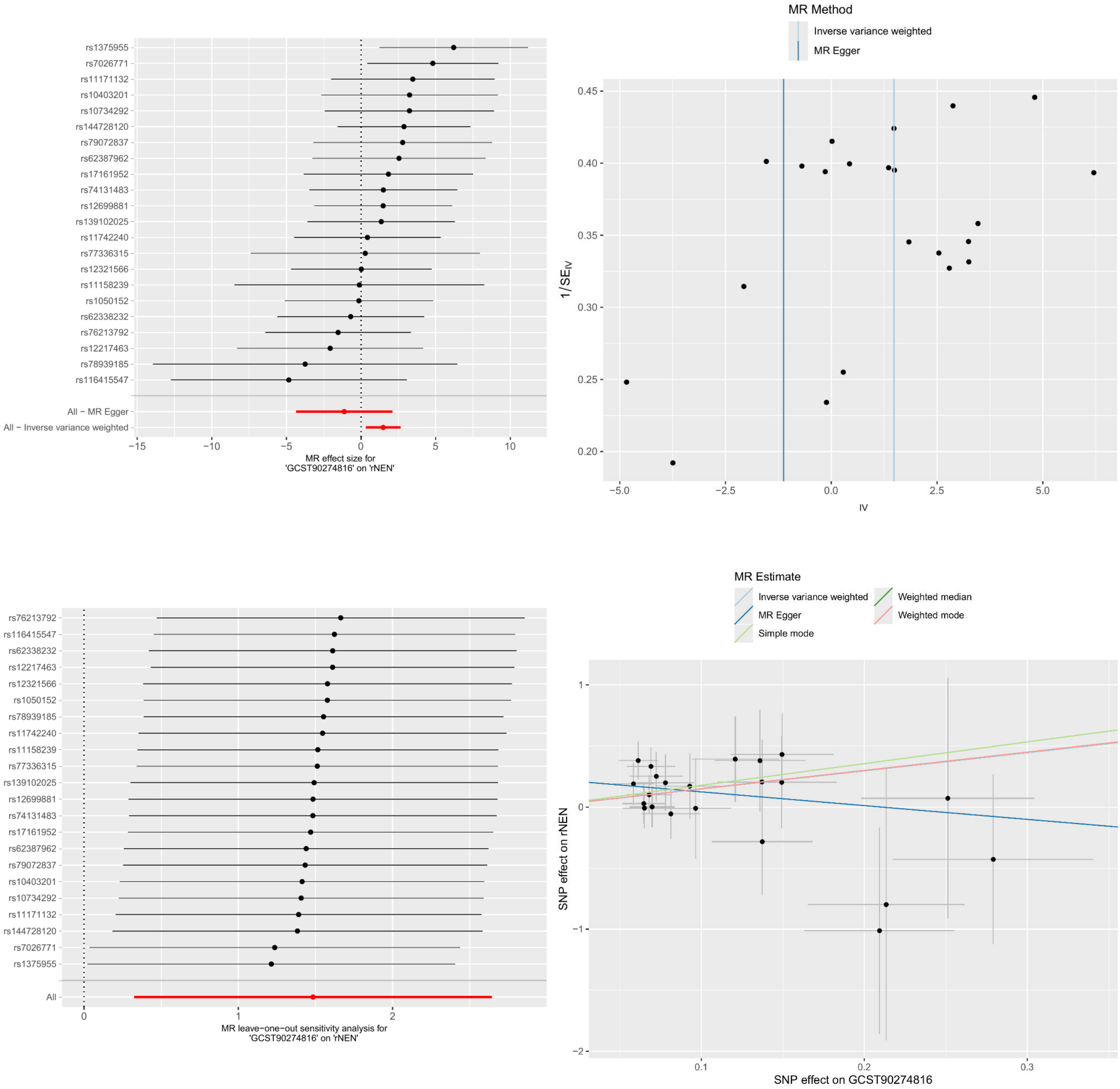

rs1375955
rs7026771
rs11171132
rs10403201
rs10734292
rs144728120
rs79072837
rs62387962
rs17161952
rs74131483
rs12699881
rs139102025
rs11742240
rs77336315
rs12321566
rs11158239
rs1050152
rs62338232
rs76213792
rs12217463
rs78939185
rs116415547
All − MR Egger
All − Inverse variance weighted
−15
−10
−5
0
5
10
MR effect size for
'GCST90274816' on 'rNEN'
MR Method
Inverse variance weighted
MR Egger
0.45
0.40
0.35
0.30
0.25
0.20
1/SE$_{IV}$
−5.0
−2.5
0.0
2.5
5.0
IV
rs76213792
rs116415547
rs62338232
rs12217463
rs12321566
rs1050152
rs78939185
rs11742240
rs11158239
rs77336315
rs139102025
rs12699881
rs74131483
rs17161952
rs62387962
rs79072837
rs10403201
rs10734292
rs11171132
rs144728120
rs7026771
rs1375955
All
0
1
2
MR leave−one−out sensitivity analysis for
'GCST90274816' on 'rNEN'
MR Estimate
Inverse variance weighted
MR Egger
Simple mode
Weighted median
Weighted mode
1
0
−1
−2
SNP effect on rNEN
0.1
0.2
0.3
SNP effect on GCST90274816

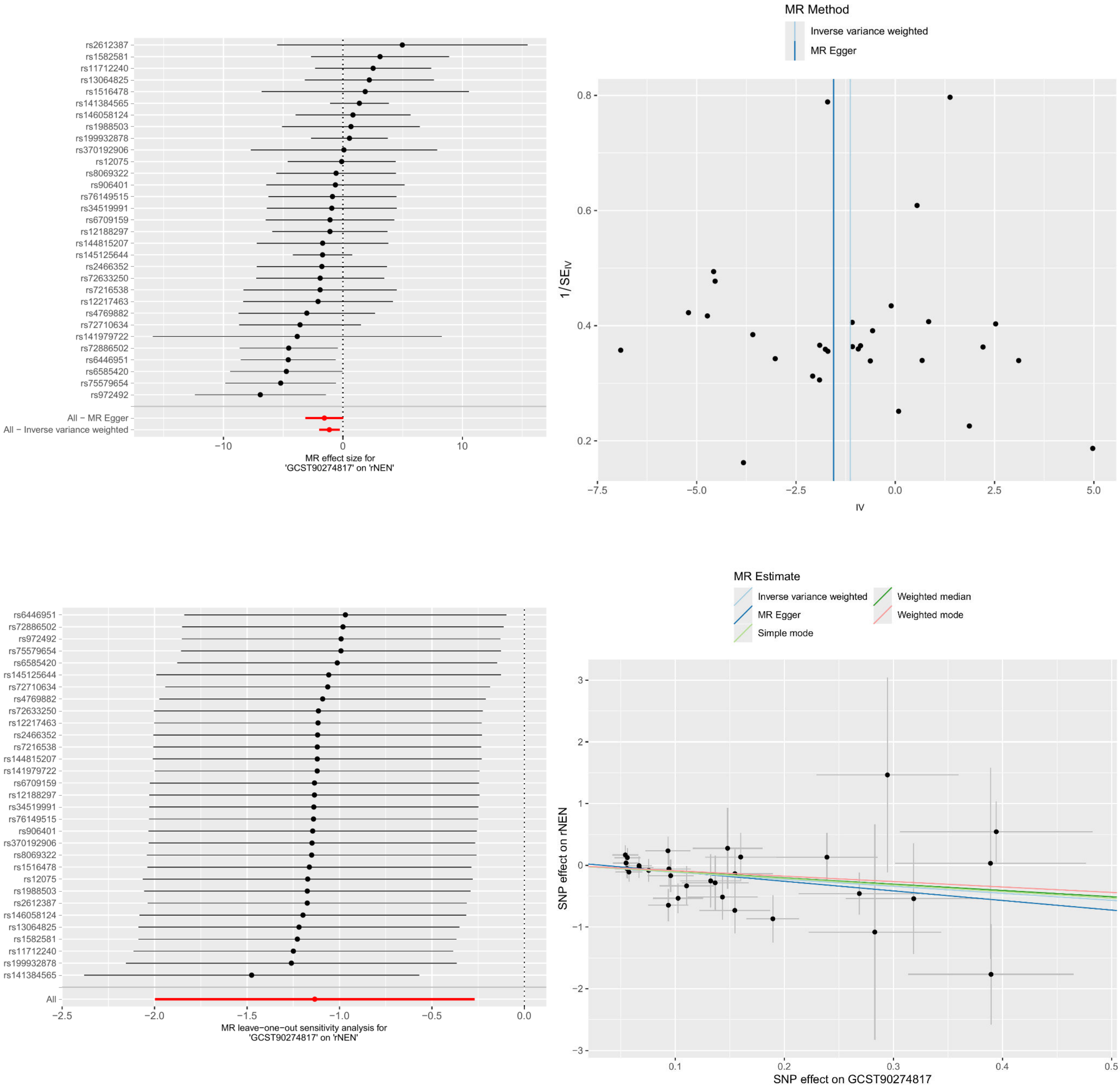

rs2612387
rs1582581
rs11712240
rs13064825
rs1516478
rs141384565
rs146058124
rs1988503
rs199932878
rs370192906
rs12075
rs8069322
rs906401
rs76149515
rs34519991
rs6709159
rs12188297
rs144815207
rs145125644
rs2466352
rs72633250
rs7216538
rs12217463
rs4769882
rs72710634
rs141979722
rs72886502
rs6446951
rs6585420
rs75579654
rs972492
All − MR Egger
All − Inverse variance weighted
−10
0
10
MR effect size for
'GCST90274817' on 'rNEN'
MR Method
Inverse variance weighted
MR Egger
1/SE$_{IV}$
0.8
0.6
0.4
0.2
−7.5
−5.0
−2.5
0.0
2.5
5.0
IV
rs6446951
rs72886502
rs972492
rs75579654
rs6585420
rs145125644
rs72710634
rs4769882
rs72633250
rs12217463
rs2466352
rs7216538
rs144815207
rs141979722
rs6709159
rs12188297
rs34519991
rs76149515
rs906401
rs370192906
rs8069322
rs1516478
rs12075
rs1988503
rs2612387
rs146058124
rs13064825
rs1582581
rs11712240
rs199932878
rs141384565
All
−2.5
−2.0
−1.5
−1.0
−0.5
0.0
MR leave−one−out sensitivity analysis for
'GCST90274817' on 'rNEN'
MR Estimate
Inverse variance weighted
MR Egger
Simple mode
Weighted median
Weighted mode
SNP effect on rNEN
3
2
1
0
−1
−2
−3
0.1
0.2
0.3
0.4
0.5
SNP effect on GCST90274817

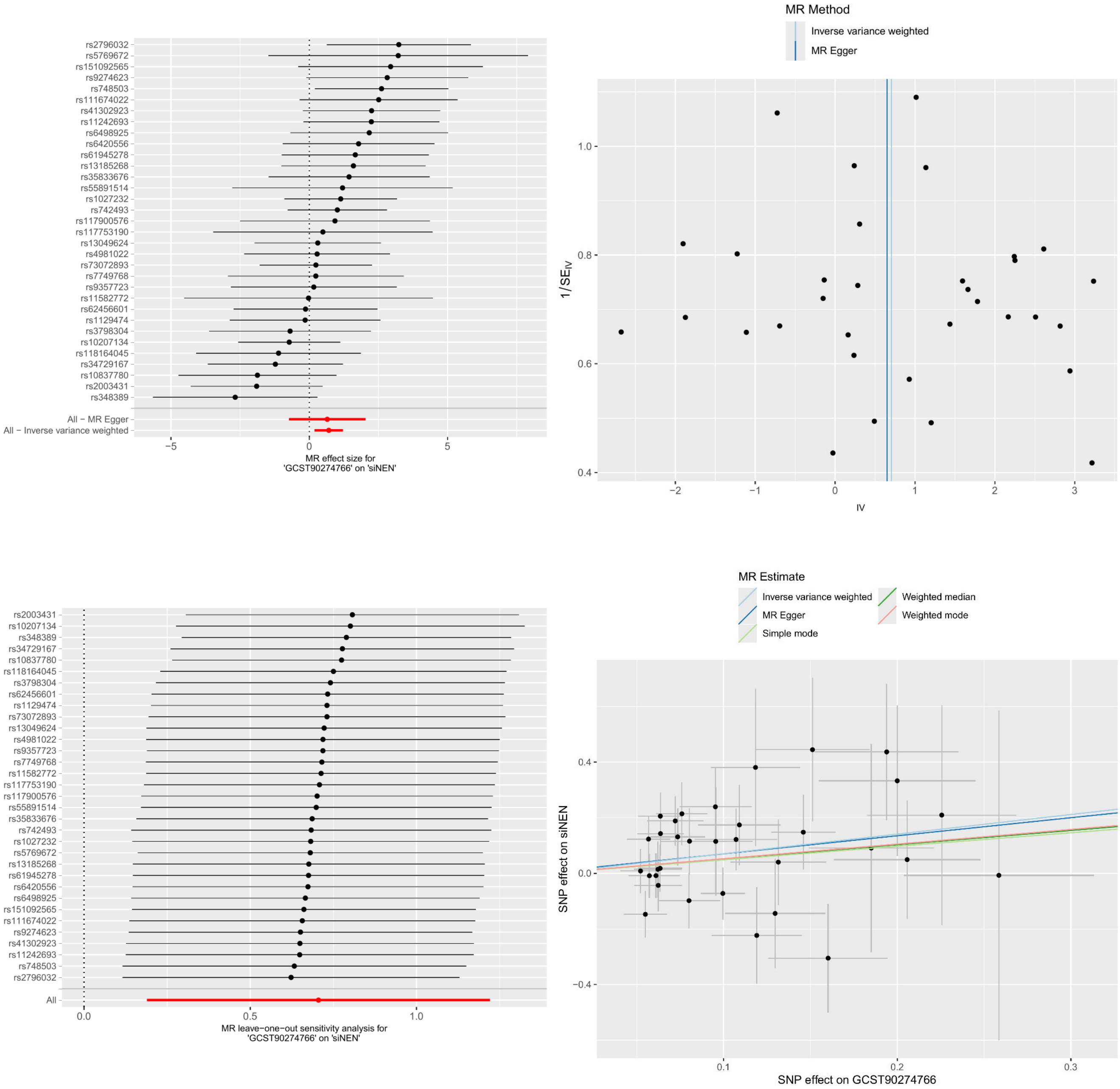
rs2796032
rs5769672
rs151092565
rs9274623
rs748503
rs111674022
rs41302923
rs11242693
rs6498925
rs6420556
rs61945278
rs13185268
rs35833676
rs55891514
rs1027232
rs742493
rs117900576
rs117753190
rs13049624
rs4981022
rs73072893
rs7749768
rs9357723
rs11582772
rs62456601
rs1129474
rs3798304
rs10207134
rs118164045
rs34729167
rs10837780
rs2003431
rs348389
All − MR Egger
All − Inverse variance weighted
−5
0
5
MR effect size for
'GCST90274766' on 'siNEN'
MR Method
Inverse variance weighted
MR Egger
1/SE$_{IV}$
1.0
0.8
0.6
0.4
−2
−1
0
1
2
3
IV
rs2003431
rs10207134
rs348389
rs34729167
rs10837780
rs118164045
rs3798304
rs62456601
rs1129474
rs73072893
rs13049624
rs4981022
rs9357723
rs7749768
rs11582772
rs117753190
rs117900576
rs55891514
rs35833676
rs742493
rs1027232
rs5769672
rs13185268
rs61945278
rs6420556
rs6498925
rs151092565
rs111674022
rs9274623
rs41302923
rs11242693
rs748503
rs2796032
All
0.0
0.5
1.0
MR leave−one−out sensitivity analysis for
'GCST90274766' on 'siNEN'
MR Estimate
Inverse variance weighted
MR Egger
Simple mode
Weighted median
Weighted mode
SNP effect on siNEN
0.4
0.0
−0.4
0.1
0.2
0.3
SNP effect on GCST90274766

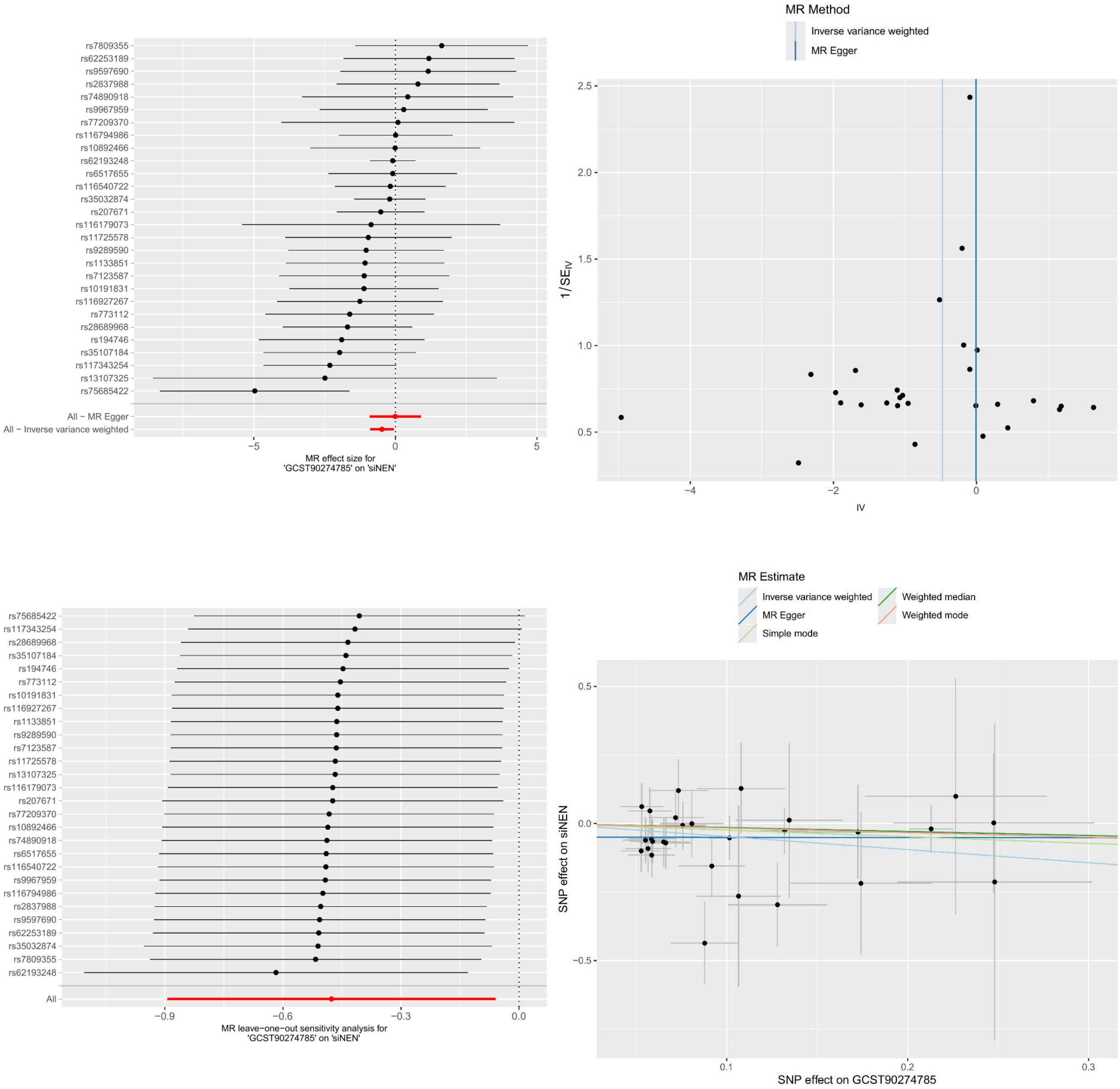
rs7809355
rs62253189
rs9597690
rs2837988
rs74890918
rs9967959
rs77209370
rs116794986
rs10892466
rs62193248
rs6517655
rs116540722
rs35032874
rs207671
rs116179073
rs11725578
rs9289590
rs1133851
rs7123587
rs10191831
rs116927267
rs773112
rs28689968
rs194746
rs35107184
rs117343254
rs13107325
rs75685422
All − MR Egger
All − Inverse variance weighted
−5
0
5
MR effect size for
'GCST90274785' on 'siNEN'
MR Method
Inverse variance weighted
MR Egger
1/SE_IV
2.5
2.0
1.5
1.0
0.5
−4
−2
0
IV
rs75685422
rs117343254
rs28689968
rs35107184
rs194746
rs773112
rs10191831
rs116927267
rs1133851
rs9289590
rs7123587
rs11725578
rs13107325
rs116179073
rs207671
rs77209370
rs10892466
rs74890918
rs6517655
rs116540722
rs9967959
rs116794986
rs2837988
rs9597690
rs62253189
rs35032874
rs7809355
rs62193248
All
−0.9
−0.6
−0.3
0.0
MR leave−one−out sensitivity analysis for
'GCST90274785' on 'siNEN'
MR Estimate
Inverse variance weighted
MR Egger
Simple mode
Weighted median
Weighted mode
0.5
0.0
−0.5
SNP effect on siNEN
0.1
0.2
0.3
SNP effect on GCST90274785

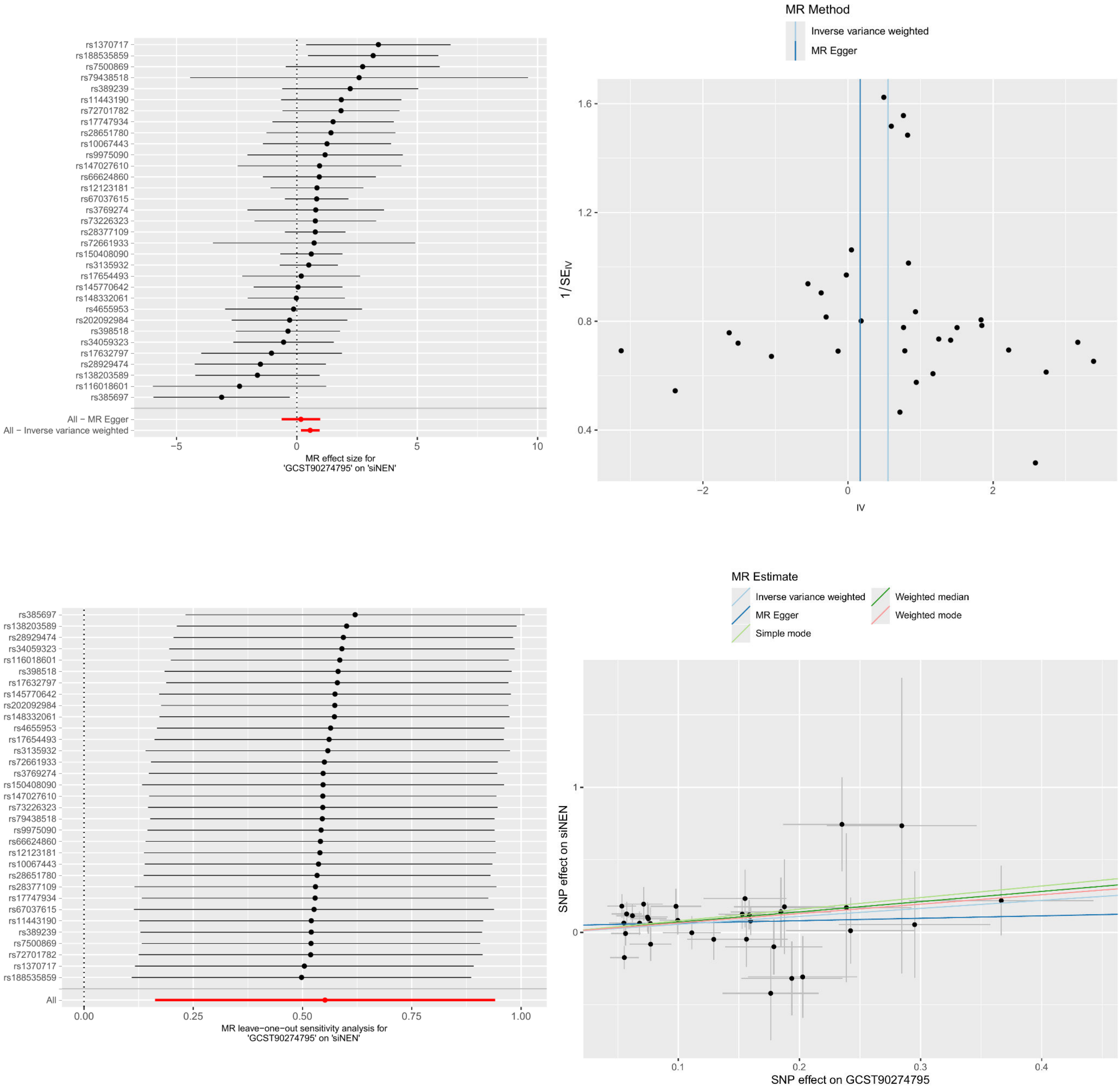

rs1370717
rs188535859
rs7500869
rs79438518
rs389239
rs11443190
rs72701782
rs17747934
rs28651780
rs10067443
rs9975090
rs147027610
rs66624860
rs12123181
rs67037615
rs3769274
rs73226323
rs28377109
rs72661933
rs150408090
rs3135932
rs17654493
rs145770642
rs148332061
rs4655953
rs202092984
rs398518
rs34059323
rs17632797
rs28929474
rs138203589
rs116018601
rs385697
All − MR Egger
All − Inverse variance weighted
−5
0
5
10
MR effect size for
'GCST90274795' on 'siNEN'
MR Method
Inverse variance weighted
MR Egger
1.6
1.2
0.8
0.4
$1/SE_{IV}$
−2
0
2
IV
rs385697
rs138203589
rs28929474
rs34059323
rs116018601
rs398518
rs17632797
rs145770642
rs202092984
rs148332061
rs4655953
rs17654493
rs3135932
rs72661933
rs3769274
rs150408090
rs147027610
rs73226323
rs79438518
rs9975090
rs66624860
rs12123181
rs10067443
rs28651780
rs28377109
rs17747934
rs67037615
rs11443190
rs389239
rs7500869
rs72701782
rs1370717
rs188535859
All
0.00
0.25
0.50
0.75
1.00
MR leave−one−out sensitivity analysis for
'GCST90274795' on 'siNEN'
MR Estimate
Inverse variance weighted
MR Egger
Simple mode
Weighted median
Weighted mode
1
0
SNP effect on siNEN
0.1
0.2
0.3
0.4
SNP effect on GCST90274795

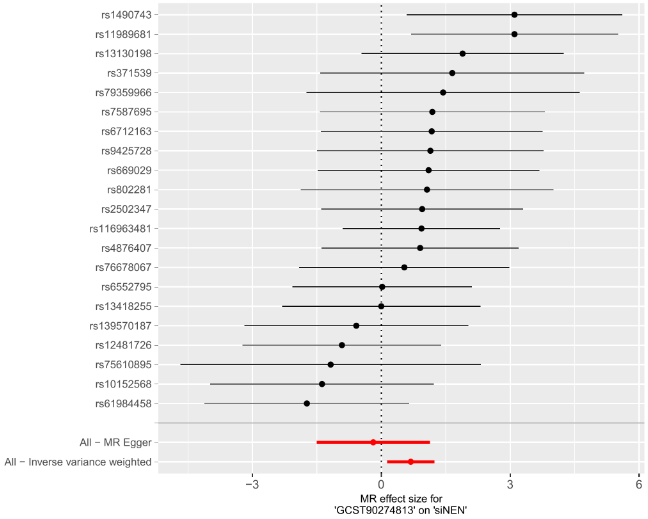
rs1490743
rs11989681
rs13130198
rs371539
rs79359966
rs7587695
rs6712163
rs9425728
rs669029
rs802281
rs2502347
rs116963481
rs4876407
rs76678067
rs6552795
rs13418255
rs139570187
rs12481726
rs75610895
rs10152568
rs61984458
All − MR Egger
All − Inverse variance weighted
−3
0
3
6
MR effect size for
'GCST90274813' on 'siNEN'

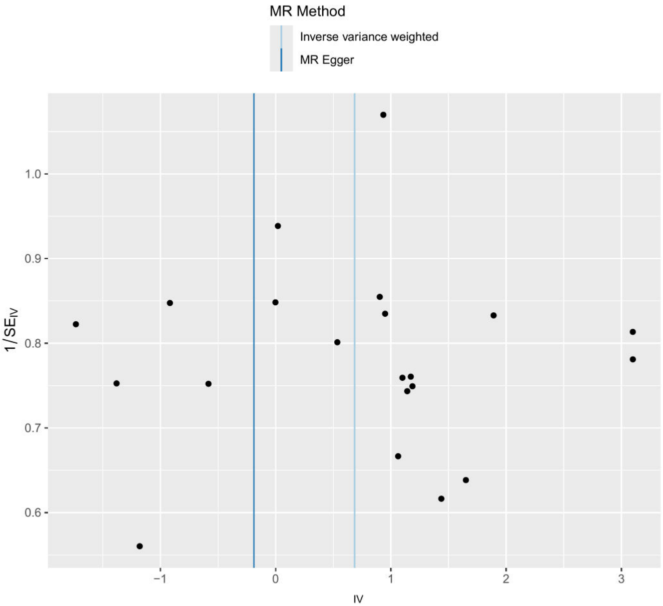
MR Method
Inverse variance weighted
MR Egger
1.0
0.9
0.8
0.7
0.6
1/SE$_{IV}$
−1
0
1
2
3
IV

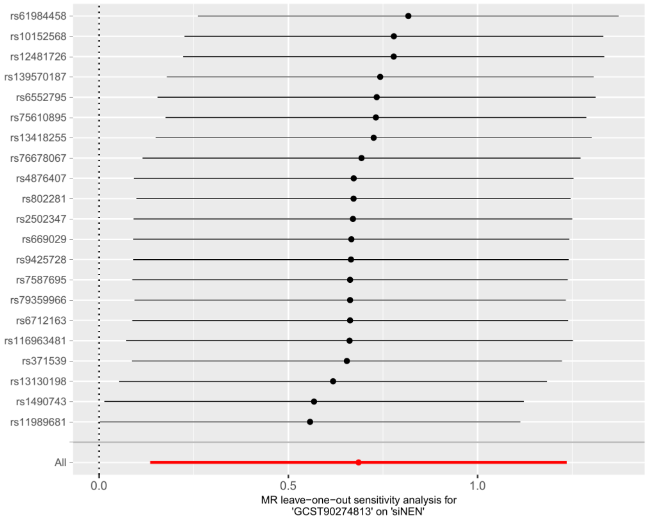
rs61984458
rs10152568
rs12481726
rs139570187
rs6552795
rs75610895
rs13418255
rs76678067
rs4876407
rs802281
rs2502347
rs669029
rs9425728
rs7587695
rs79359966
rs6712163
rs116963481
rs371539
rs13130198
rs1490743
rs11989681
All
0.0
0.5
1.0
MR leave−one−out sensitivity analysis for
'GCST90274813' on 'siNEN'

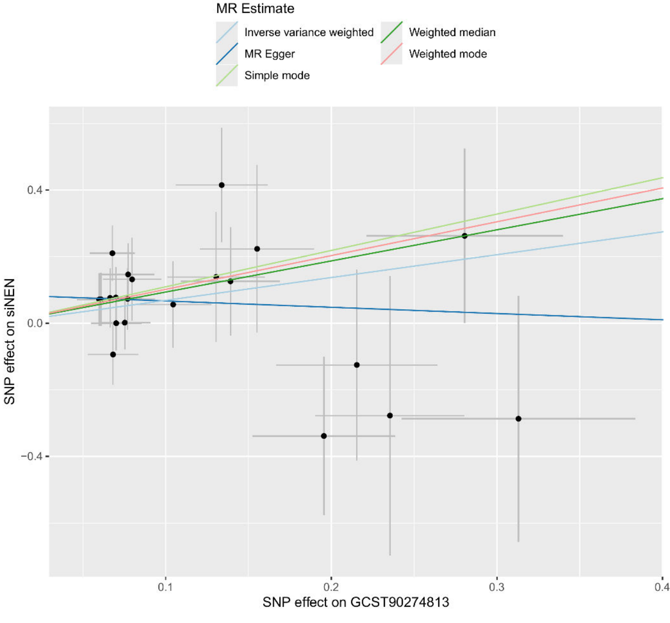
MR Estimate
Inverse variance weighted
MR Egger
Simple mode
Weighted median
Weighted mode
0.4
0.0
−0.4
SNP effect on siNEN
0.1
0.2
0.3
0.4
SNP effect on GCST90274813

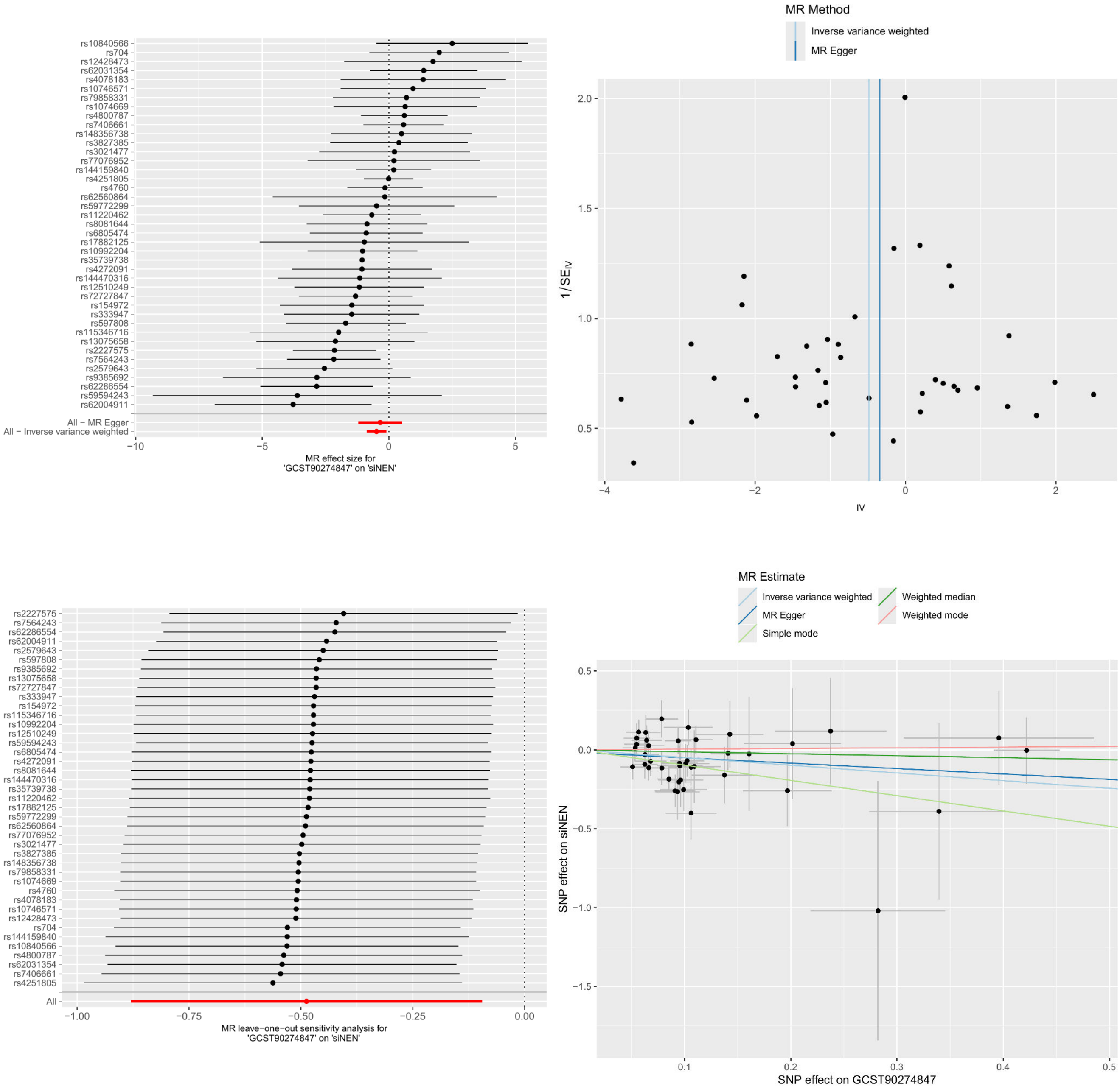
rs10840566
rs704
rs12428473
rs62031354
rs4078183
rs10746571
rs79858331
rs1074669
rs4800787
rs7406661
rs148356738
rs3827385
rs3021477
rs77076952
rs144159840
rs4251805
rs4760
rs62560864
rs59772299
rs11220462
rs8081644
rs6805474
rs17882125
rs10992204
rs35739738
rs4272091
rs144470316
rs12510249
rs72727847
rs154972
rs333947
rs597808
rs115346716
rs13075658
rs2227575
rs7564243
rs2579643
rs9385692
rs62286554
rs59594243
rs62004911
All − MR Egger
All − Inverse variance weighted
−10
−5
0
5
MR effect size for
'GCST90274847' on 'siNEN'
MR Method
Inverse variance weighted
MR Egger
$1/SE_{IV}$
2.0
1.5
1.0
0.5
−4
−2
0
2
IV
rs2227575
rs7564243
rs62286554
rs62004911
rs2579643
rs597808
rs9385692
rs13075658
rs72727847
rs333947
rs154972
rs115346716
rs10992204
rs12510249
rs59594243
rs6805474
rs4272091
rs8081644
rs144470316
rs35739738
rs11220462
rs17882125
rs59772299
rs62560864
rs77076952
rs3021477
rs3827385
rs148356738
rs79858331
rs1074669
rs4760
rs4078183
rs10746571
rs12428473
rs704
rs144159840
rs10840566
rs4800787
rs62031354
rs7406661
rs4251805
All
−1.00
−0.75
−0.50
−0.25
0.00
MR leave−one−out sensitivity analysis for
'GCST90274847' on 'siNEN'
MR Estimate
Inverse variance weighted
MR Egger
Simple mode
Weighted median
Weighted mode
0.5
0.0
−0.5
−1.0
−1.5
SNP effect on siNEN
0.1
0.2
0.3
0.4
0.5
SNP effect on GCST90274847

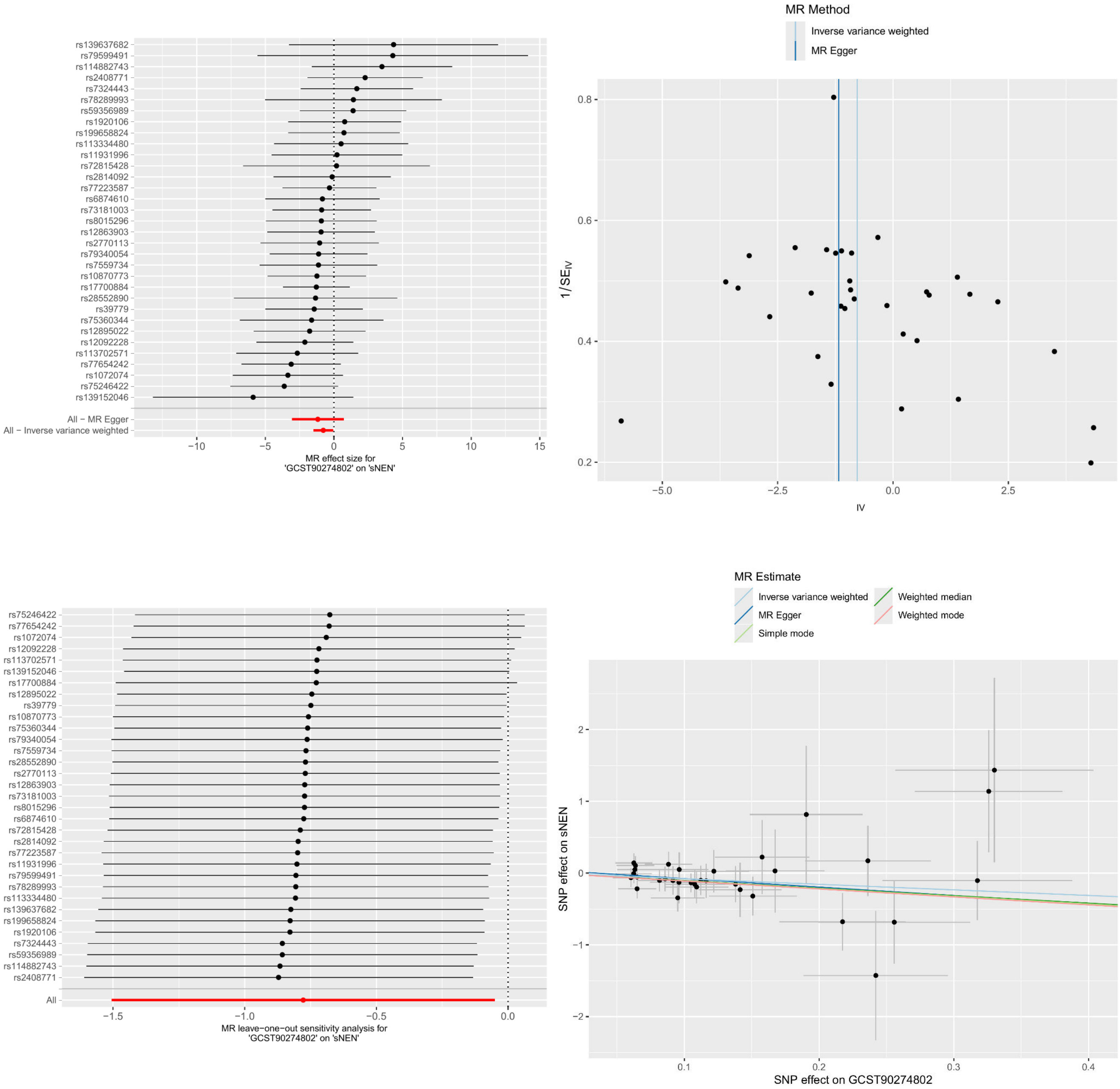

rs139637682
rs79599491
rs114882743
rs2408771
rs7324443
rs78289993
rs59356989
rs1920106
rs199658824
rs113334480
rs11931996
rs72815428
rs2814092
rs77223587
rs6874610
rs73181003
rs8015296
rs12863903
rs2770113
rs79340054
rs7559734
rs10870773
rs17700884
rs28552890
rs39779
rs75360344
rs12895022
rs12092228
rs113702571
rs77654242
rs1072074
rs75246422
rs139152046
All − MR Egger
All − Inverse variance weighted
−10
−5
0
5
10
15
MR effect size for
'GCST90274802' on 'sNEN'
MR Method
Inverse variance weighted
MR Egger
0.8
0.6
0.4
0.2
1/SE_IV
−5.0
−2.5
0.0
2.5
IV
rs75246422
rs77654242
rs1072074
rs12092228
rs113702571
rs139152046
rs17700884
rs12895022
rs39779
rs10870773
rs75360344
rs79340054
rs7559734
rs28552890
rs2770113
rs12863903
rs73181003
rs8015296
rs6874610
rs72815428
rs2814092
rs77223587
rs11931996
rs79599491
rs78289993
rs113334480
rs139637682
rs199658824
rs1920106
rs7324443
rs59356989
rs114882743
rs2408771
All
−1.5
−1.0
−0.5
0.0
MR leave−one−out sensitivity analysis for
'GCST90274802' on 'sNEN'
MR Estimate
Inverse variance weighted
MR Egger
Simple mode
Weighted median
Weighted mode
2
1
0
−1
−2
SNP effect on sNEN
0.1
0.2
0.3
0.4
SNP effect on GCST90274802

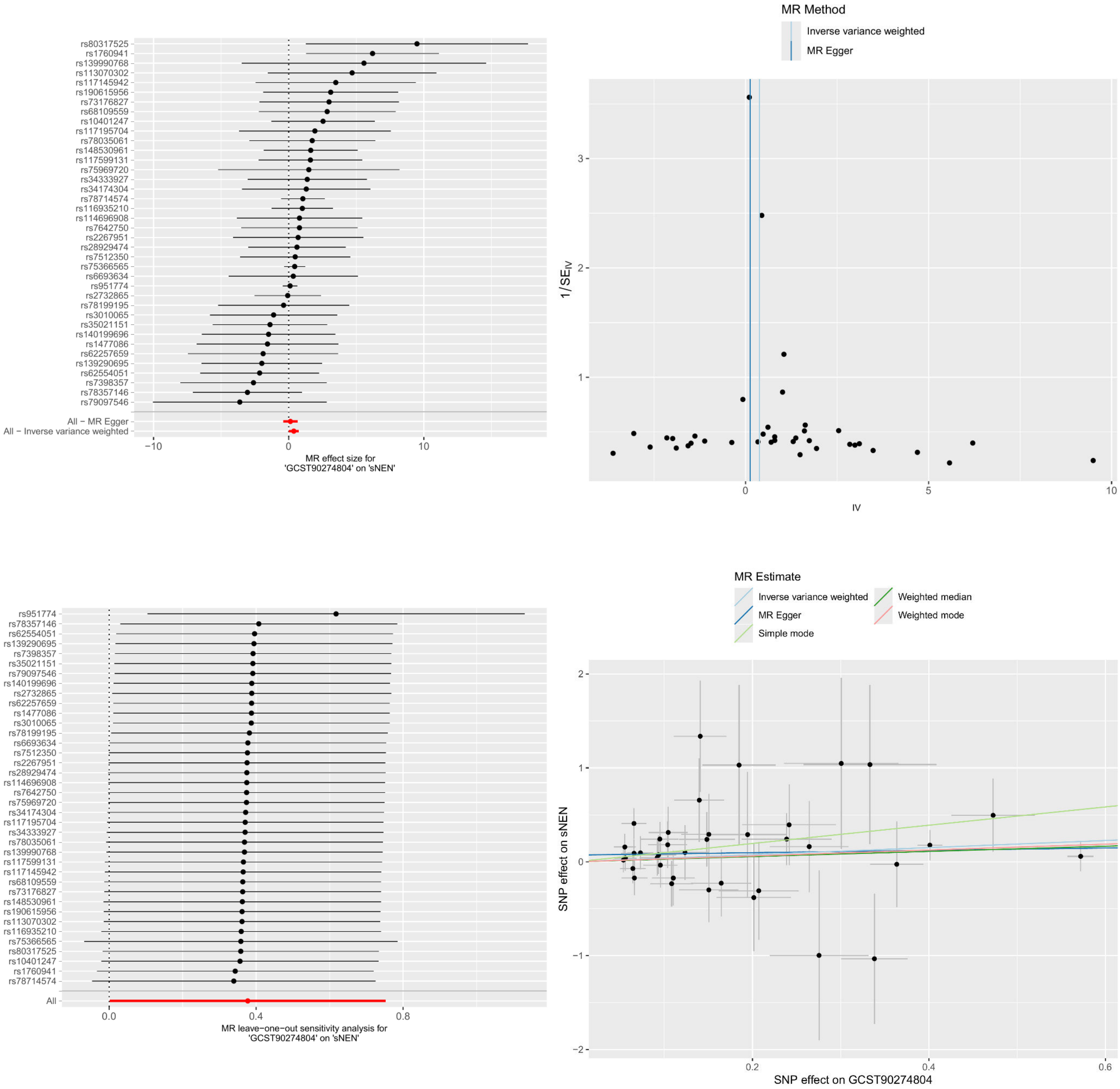

rs80317525
rs1760941
rs139990768
rs113070302
rs117145942
rs190615956
rs73176827
rs68109559
rs10401247
rs117195704
rs78035061
rs148530961
rs117599131
rs75969720
rs34333927
rs34174304
rs78714574
rs116935210
rs114696908
rs7642750
rs2267951
rs28929474
rs7512350
rs75366565
rs6693634
rs951774
rs2732865
rs78199195
rs3010065
rs35021151
rs140199696
rs1477086
rs62257659
rs139290695
rs62554051
rs7398357
rs78357146
rs79097546
All − MR Egger
All − Inverse variance weighted
−10
0
10
MR effect size for
'GCST90274804' on 'sNEN'
MR Method
Inverse variance weighted
MR Egger
1/SE$_{IV}$
IV
rs951774
rs78357146
rs62554051
rs139290695
rs7398357
rs35021151
rs79097546
rs140199696
rs2732865
rs62257659
rs1477086
rs3010065
rs78199195
rs6693634
rs7512350
rs2267951
rs28929474
rs114696908
rs7642750
rs75969720
rs34174304
rs117195704
rs34333927
rs78035061
rs139990768
rs117599131
rs117145942
rs68109559
rs73176827
rs148530961
rs190615956
rs113070302
rs116935210
rs75366565
rs80317525
rs10401247
rs1760941
rs78714574
All
0.0
0.4
0.8
MR leave−one−out sensitivity analysis for
'GCST90274804' on 'sNEN'
MR Estimate
Inverse variance weighted
MR Egger
Simple mode
Weighted median
Weighted mode
SNP effect on sNEN
SNP effect on GCST90274804

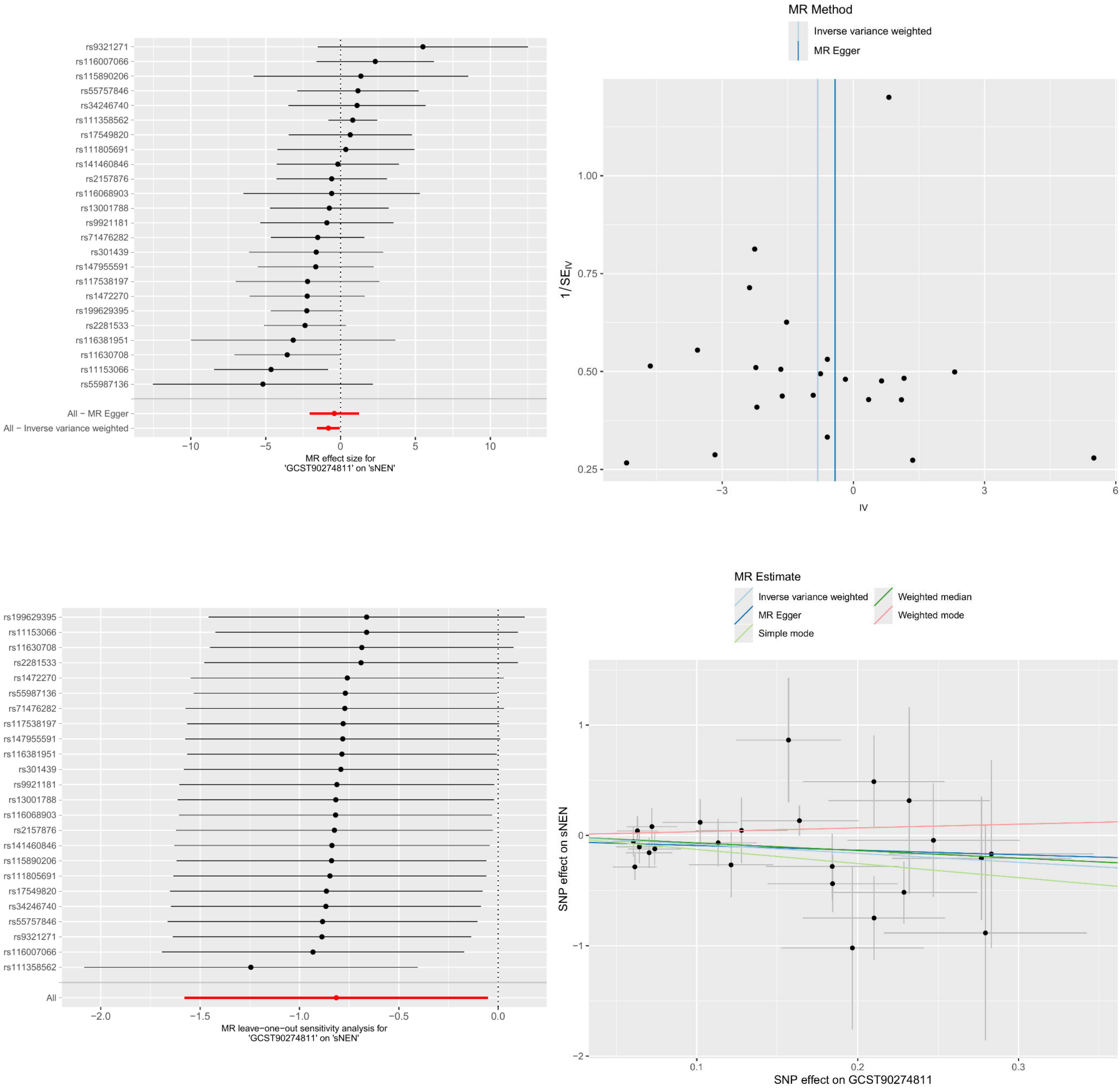

rs9321271
rs116007066
rs115890206
rs55757846
rs34246740
rs111358562
rs17549820
rs111805691
rs141460846
rs2157876
rs116068903
rs13001788
rs9921181
rs71476282
rs301439
rs147955591
rs117538197
rs1472270
rs199629395
rs2281533
rs116381951
rs11630708
rs11153066
rs55987136
All − MR Egger
All − Inverse variance weighted
−10
−5
0
5
10
MR effect size for
'GCST90274811' on 'sNEN'
MR Method
Inverse variance weighted
MR Egger
1/SEIV
1.00
0.75
0.50
0.25
−3
0
3
6
IV
rs199629395
rs11153066
rs11630708
rs2281533
rs1472270
rs55987136
rs71476282
rs117538197
rs147955591
rs116381951
rs301439
rs9921181
rs13001788
rs116068903
rs2157876
rs141460846
rs115890206
rs111805691
rs17549820
rs34246740
rs55757846
rs9321271
rs116007066
rs111358562
All
−2.0
−1.5
−1.0
−0.5
0.0
MR leave−one−out sensitivity analysis for
'GCST90274811' on 'sNEN'
MR Estimate
Inverse variance weighted
MR Egger
Simple mode
Weighted median
Weighted mode
SNP effect on sNEN
1
0
−1
−2
0.1
0.2
0.3
SNP effect on GCST90274811

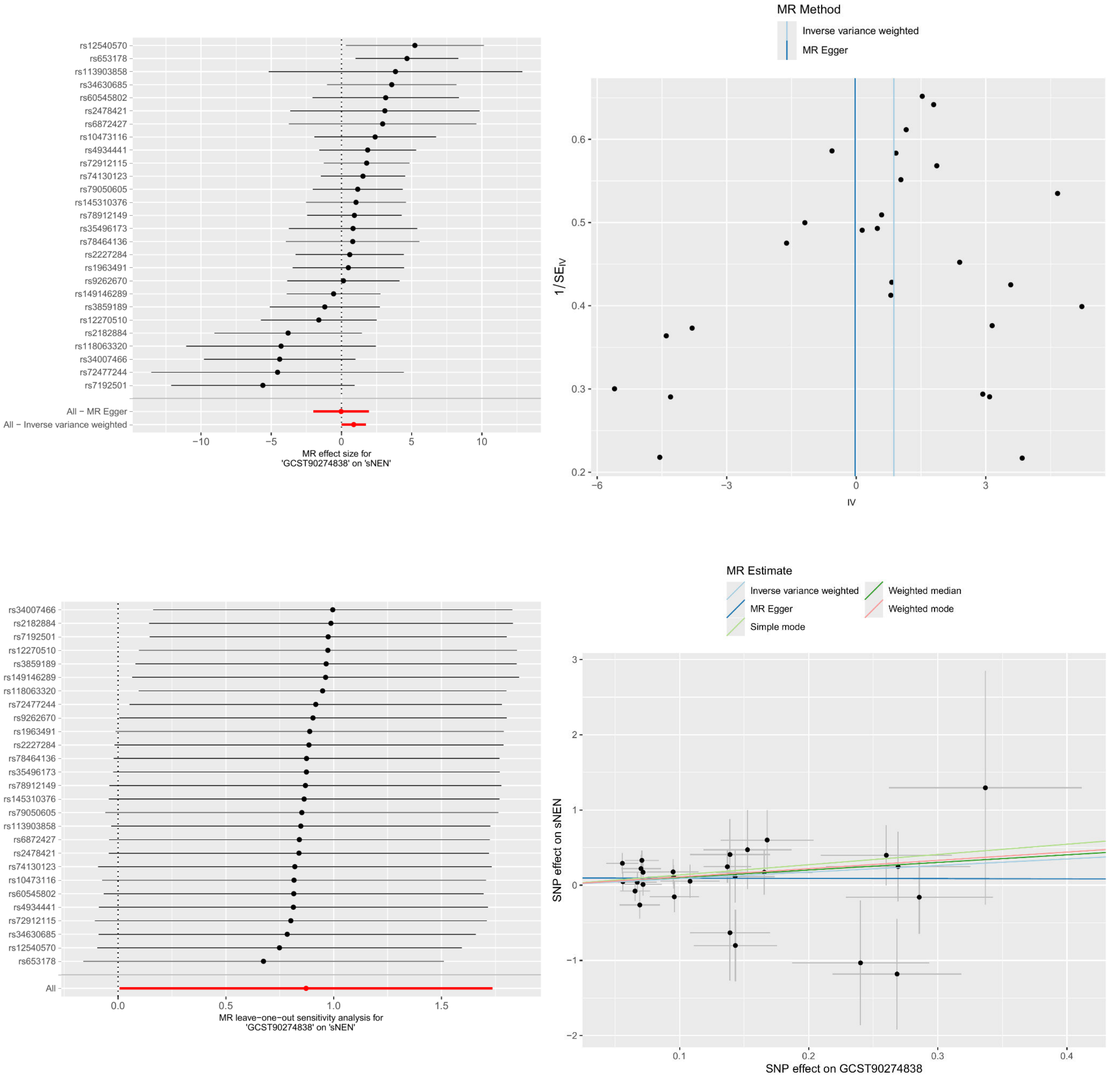

rs12540570
rs653178
rs113903858
rs34630685
rs60545802
rs2478421
rs6872427
rs10473116
rs4934441
rs72912115
rs74130123
rs79050605
rs145310376
rs78912149
rs35496173
rs78464136
rs2227284
rs1963491
rs9262670
rs149146289
rs3859189
rs12270510
rs2182884
rs118063320
rs34007466
rs72477244
rs7192501
All − MR Egger
All − Inverse variance weighted
−10
−5
0
5
10
MR effect size for
'GCST90274838' on 'sNEN'
MR Method
Inverse variance weighted
MR Egger
1/SE$_{IV}$
IV
0.2
0.3
0.4
0.5
0.6
−6
−3
0
3
rs34007466
rs2182884
rs7192501
rs12270510
rs3859189
rs149146289
rs118063320
rs72477244
rs9262670
rs1963491
rs2227284
rs78464136
rs35496173
rs78912149
rs145310376
rs79050605
rs113903858
rs6872427
rs2478421
rs74130123
rs10473116
rs60545802
rs4934441
rs72912115
rs34630685
rs12540570
rs653178
All
0.0
0.5
1.0
1.5
MR leave−one−out sensitivity analysis for
'GCST90274838' on 'sNEN'
MR Estimate
Inverse variance weighted
MR Egger
Simple mode
Weighted median
Weighted mode
SNP effect on sNEN
SNP effect on GCST90274838
−2
−1
0
1
2
3
0.1
0.2
0.3
0.4
