# Supplementary material for: Associations Between Gastroenteropancreatic Neuroendocrine Neoplasms and Inflammatory Factors: Insights From a Two‐Sample Mendelian Randomization Analysis
Source: Can J Gastroenterol Hepatol. 2025 Dec 26;2025:2591387. doi: 10.1155/cjgh/2591387 (PMC12741578; doi:10.1155/cjgh/2591387)
Supplement: Supplementary file 2 — Supporting Information 2 Supporting Figure S2: MR analysis of 731 immune cell traits and the risk of five GEP‐NEN subtypes. Four types of plots are shown (from left to right): Forest plot, displaying the SNP‐specific effect estimates (Wald ratio) with the IVW estimate indicated by a red line; a rightward shift of the IVW line suggests that genetically predicted immune cell traits increase GEP‐NEN risk. Funnel plot, plotting SNP effects against their standard errors to evaluate symmetry around the IVW estimate, where symmetry supports the absence of directional pleiotropy and robust MR results. Leave‐one‐out analysis, assessing the influence of individual SNPs on the overall IVW estimate; the stability of the “All” estimate indicates robustness, while large deviations upon excluding one SNP suggest potential outlier effects. Scatter plot, showing SNP effects on immune cell traits (x‐axis) versus GEP‐NEN risk (y‐axis), with fitted regression lines from multiple MR methods; an upward trend indicates that increased immune cell traits are associated with higher GEP‐NEN risk. Together, these complementary analyses demonstrate the consistency and robustness of the MR findings. [file CJGH-2025-2591387-s001.pdf]

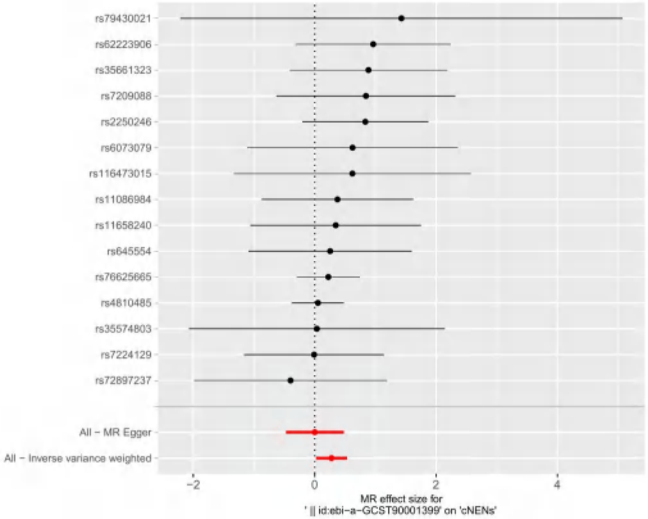
rs79430021
rs62223906
rs35661323
rs7209088
rs2250246
rs6073079
rs116473015
rs11086984
rs11658240
rs645554
rs76625665
rs4810485
rs35574803
rs7224129
rs72897237
All − MR Egger
All − Inverse variance weighted
−2
0
2
4
MR effect size for
' || id:ebi−a−GCST90001399' on 'cNENs'

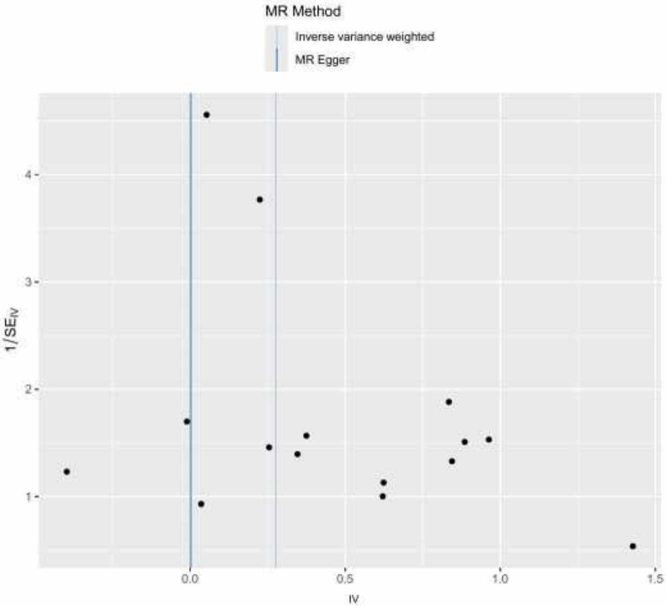
MR Method
Inverse variance weighted
MR Egger
1/SE$_{IV}$
1
2
3
4
0.0
0.5
1.0
1.5
IV

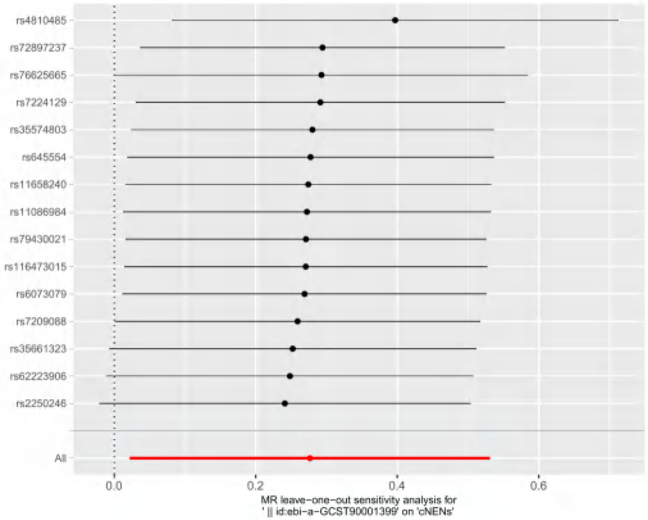
rs4810485
rs72897237
rs76625665
rs7224129
rs35574803
rs645554
rs11658240
rs11086984
rs79430021
rs116473015
rs6073079
rs7209088
rs35661323
rs62223906
rs2250246
All
0.0
0.2
0.4
0.6
MR leave−one−out sensitivity analysis for
' || id:ebi−a−GCST90001399' on 'cNENs'

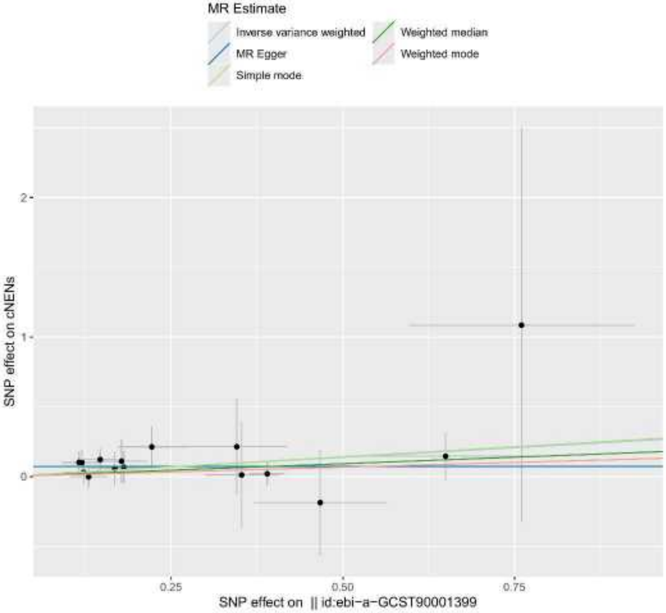
MR Estimate
Inverse variance weighted
MR Egger
Simple mode
Weighted median
Weighted mode
SNP effect on cNENs
0
1
2
0.25
0.50
0.75
SNP effect on || id:ebi−a−GCST90001399

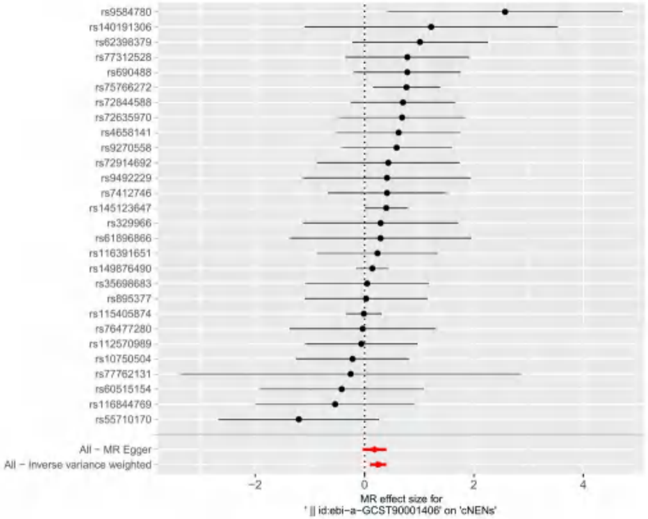

rs9584780
rs140191306
rs62398379
rs77312528
rs690488
rs75766272
rs72844588
rs72635970
rs4658141
rs9270558
rs72914692
rs9492229
rs7412746
rs145123647
rs329966
rs61896866
rs116391651
rs149876490
rs35698683
rs895377
rs115405874
rs76477280
rs112570989
rs10750504
rs77762131
rs60515154
rs116844769
rs55710170
All − MR Egger
All − Inverse variance weighted
−2
0
2
4
MR effect size for
' || id:ebi−a−GCST90001406' on 'cNENs'

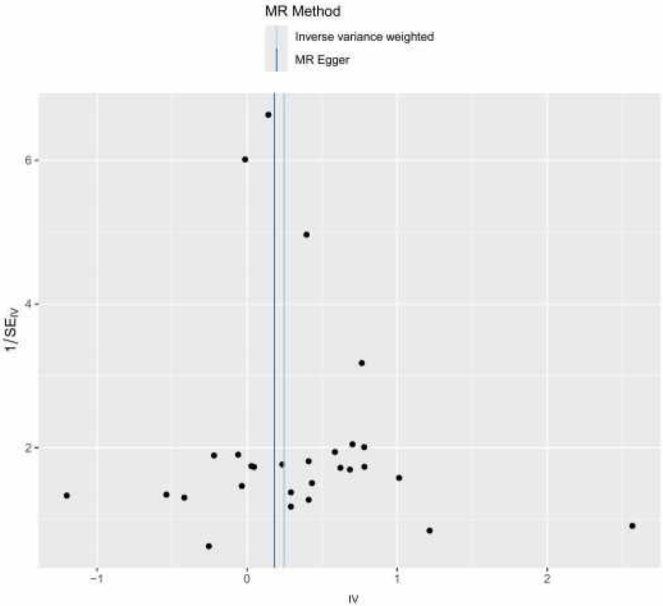

MR Method
Inverse variance weighted
MR Egger
6
4
2
1/SE$_{IV}$
−1
0
1
2
IV

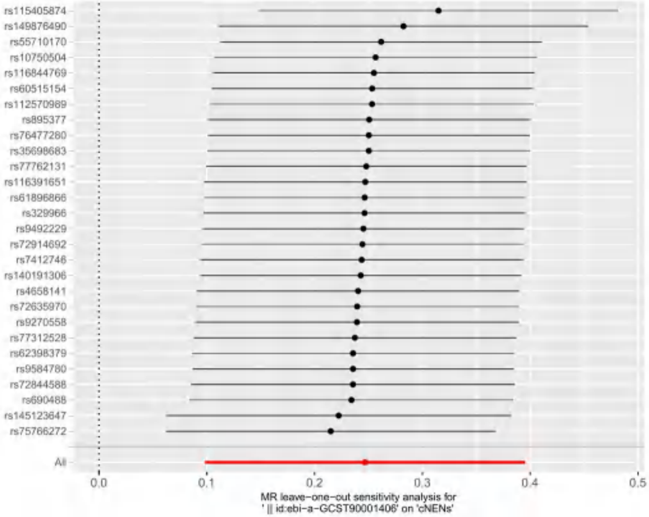

rs115405874
rs149876490
rs55710170
rs10750504
rs116844769
rs60515154
rs112570989
rs895377
rs76477280
rs35698683
rs77762131
rs116391651
rs61896866
rs329966
rs9492229
rs72914692
rs7412746
rs140191306
rs4658141
rs72635970
rs9270558
rs77312528
rs62398379
rs9584780
rs72844588
rs690488
rs145123647
rs75766272
All
0.0
0.1
0.2
0.3
0.4
0.5
MR leave−one−out sensitivity analysis for
' || id:ebi−a−GCST90001406' on 'cNENs'

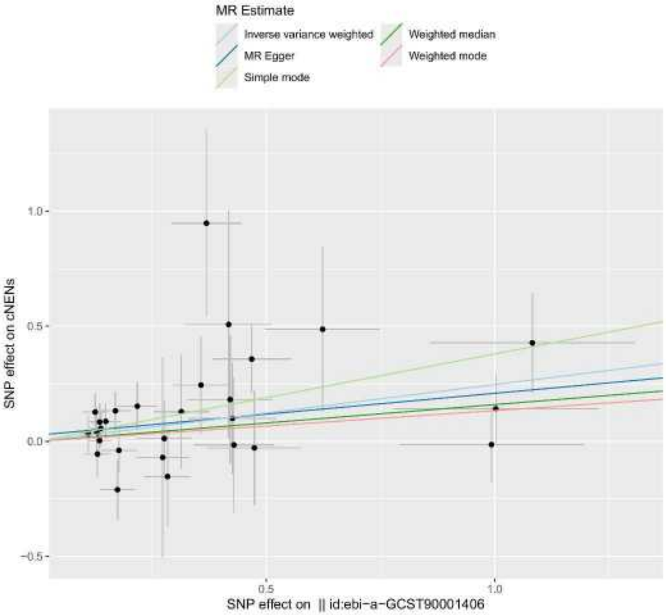

MR Estimate
Inverse variance weighted
MR Egger
Simple mode
Weighted median
Weighted mode
1.0
0.5
0.0
−0.5
SNP effect on cNENs
0.5
1.0
SNP effect on || id:ebi−a−GCST90001406

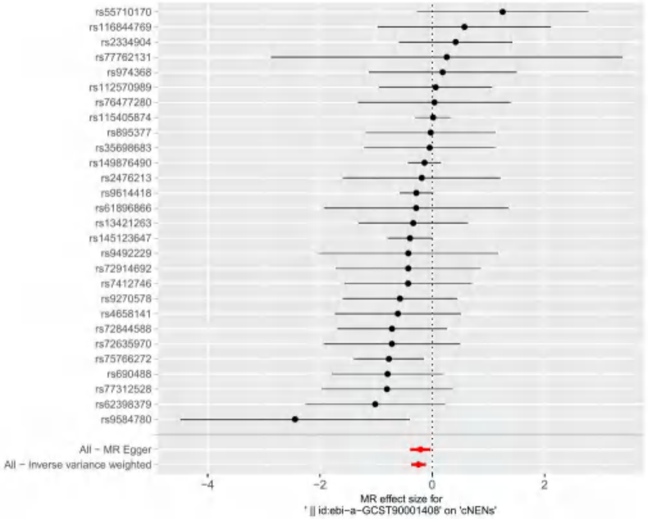
rs55710170
rs116844769
rs2334904
rs77762131
rs974368
rs112570989
rs76477280
rs115405874
rs895377
rs35698683
rs149876490
rs2476213
rs9614418
rs61896866
rs13421263
rs145123647
rs9492229
rs72914692
rs7412746
rs9270578
rs4658141
rs72844588
rs72635970
rs75766272
rs690488
rs77312528
rs62398379
rs9584780
All − MR Egger
All − Inverse variance weighted
−4
−2
0
2
MR effect size for
' || id:ebi−a−GCST90001408' on 'cNENs'

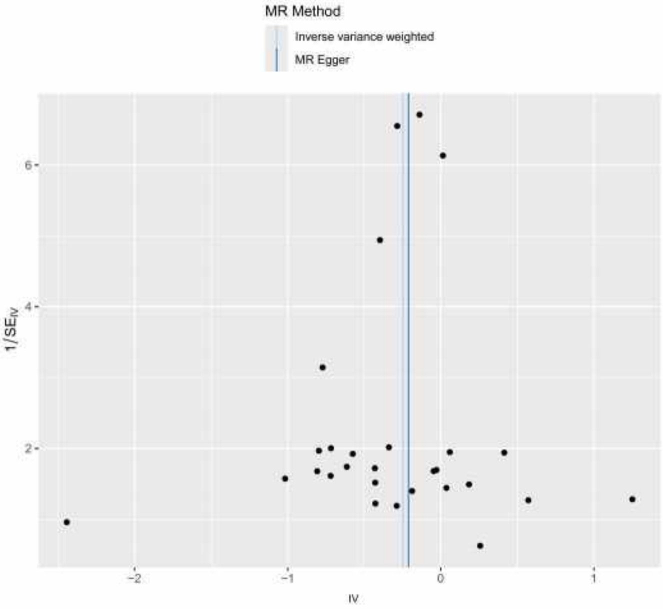
MR Method
Inverse variance weighted
MR Egger
1/SE_IV
6
4
2
−2
−1
0
1
IV

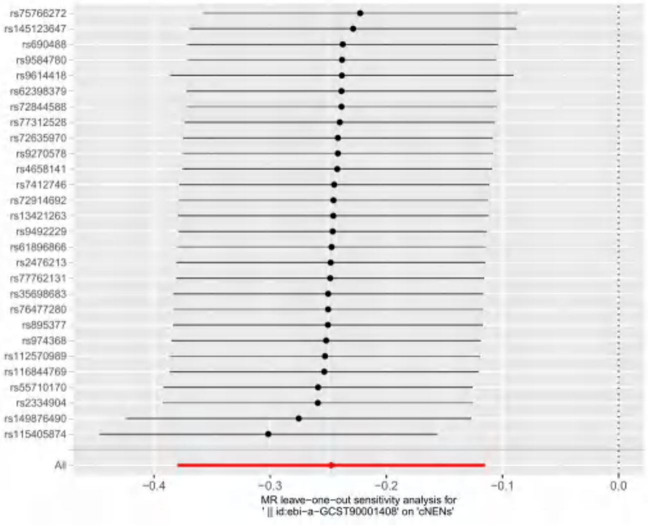
rs75766272
rs145123647
rs690488
rs9584780
rs9614418
rs62398379
rs72844588
rs77312528
rs72635970
rs9270578
rs4658141
rs7412746
rs72914692
rs13421263
rs9492229
rs61896866
rs2476213
rs77762131
rs35698683
rs76477280
rs895377
rs974368
rs112570989
rs116844769
rs55710170
rs2334904
rs149876490
rs115405874
All
−0.4
−0.3
−0.2
−0.1
0.0
MR leave−one−out sensitivity analysis for
' || id:ebi−a−GCST90001408' on 'cNENs'

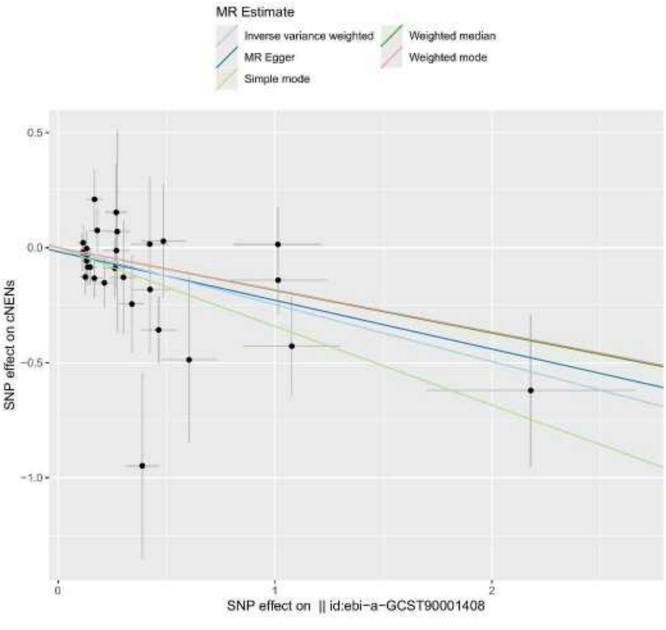
MR Estimate
Inverse variance weighted
MR Egger
Simple mode
Weighted median
Weighted mode
0.5
0.0
−0.5
−1.0
SNP effect on cNENs
0
1
2
SNP effect on || id:ebi−a−GCST90001408

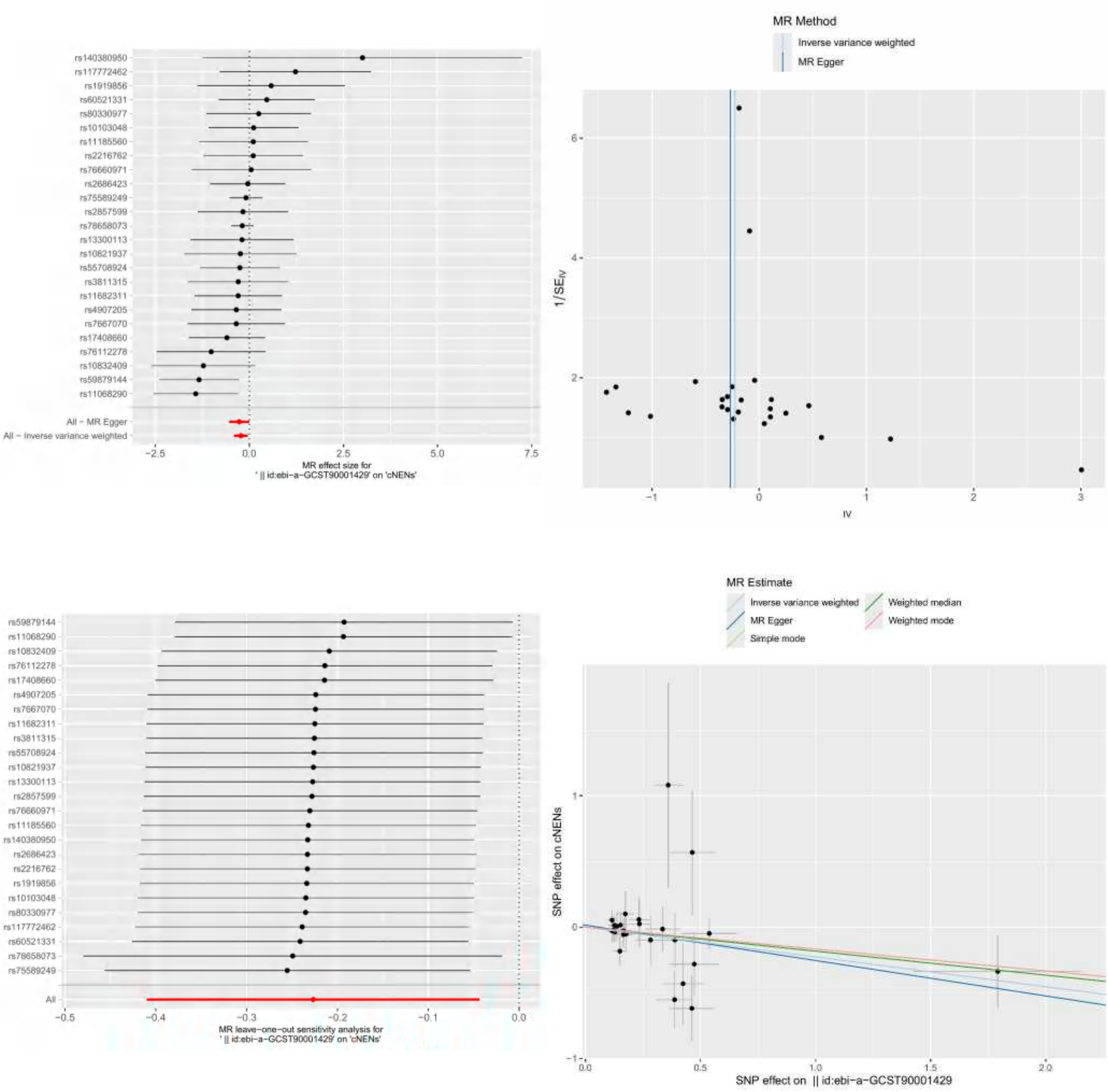

rs140380950
rs117772462
rs1919856
rs60521331
rs80330977
rs10103048
rs11185560
rs2216762
rs76660971
rs2686423
rs75589249
rs2857599
rs78658073
rs13300113
rs10821937
rs55708924
rs3811315
rs11682311
rs4907205
rs7667070
rs17408660
rs76112278
rs10832409
rs59879144
rs11068290
All − MR Egger
All − Inverse variance weighted
MR effect size for
' || id:ebi−a−GCST90001429' on 'cNENs'
MR Method
Inverse variance weighted
MR Egger
1/SE_IV
IV
rs59879144
rs11068290
rs10832409
rs76112278
rs17408660
rs4907205
rs7667070
rs11682311
rs3811315
rs55708924
rs10821937
rs13300113
rs2857599
rs76660971
rs11185560
rs140380950
rs2686423
rs2216762
rs1919856
rs10103048
rs80330977
rs117772462
rs60521331
rs78658073
rs75589249
All
MR leave−one−out sensitivity analysis for
' || id:ebi−a−GCST90001429' on 'cNENs'
MR Estimate
Inverse variance weighted
MR Egger
Simple mode
Weighted median
Weighted mode
SNP effect on cNENs
SNP effect on || id:ebi−a−GCST90001429

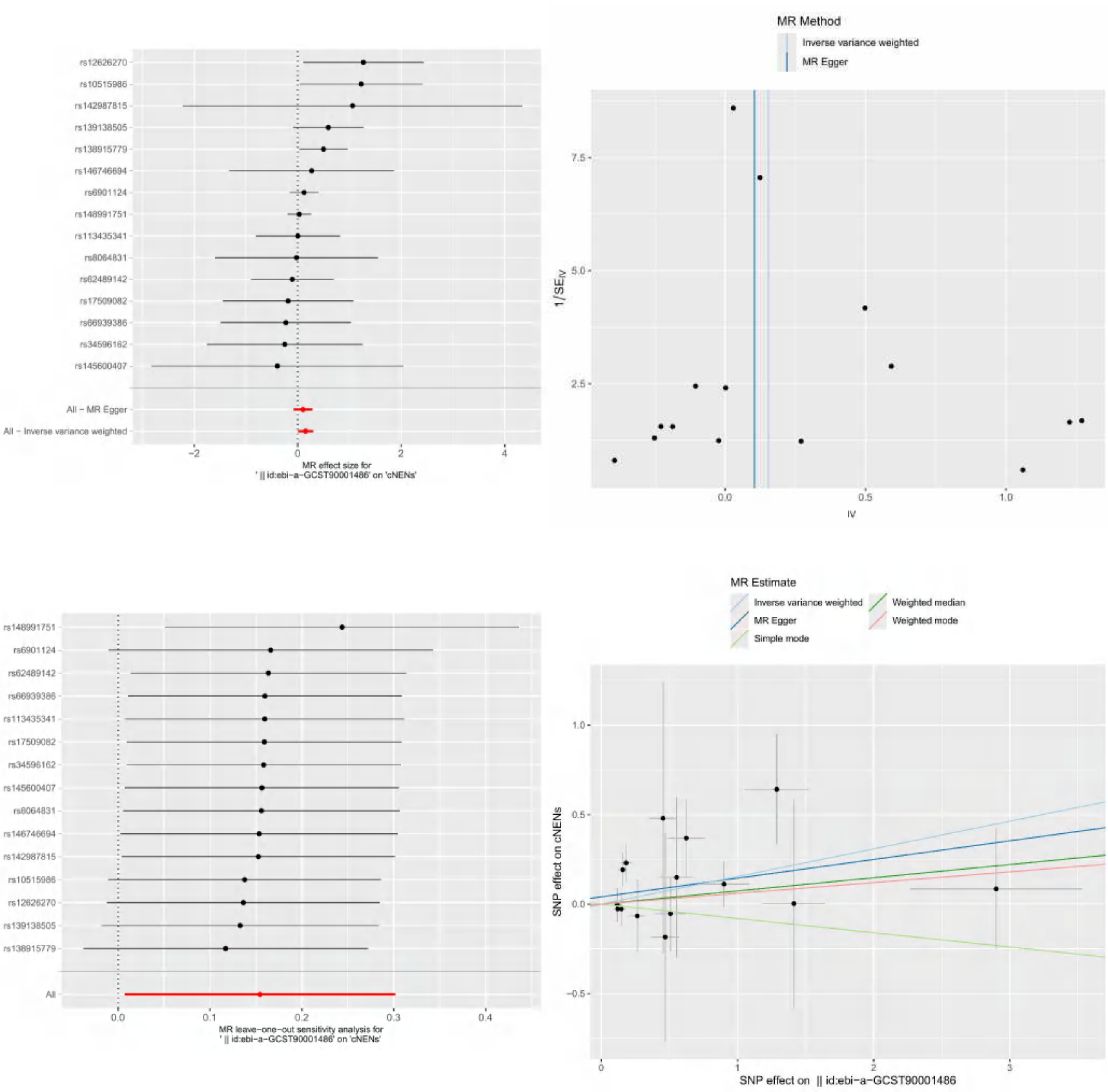

rs12626270
rs10515986
rs142987815
rs139138505
rs138915779
rs146746694
rs6901124
rs148991751
rs113435341
rs8064831
rs62489142
rs17509082
rs66939386
rs34596162
rs145600407
All − MR Egger
All − Inverse variance weighted
MR effect size for
' || id:ebi−a−GCST90001486' on 'cNENs'
MR Method
Inverse variance weighted
MR Egger
1/SE$_{IV}$
IV
MR leave−one−out sensitivity analysis for
' || id:ebi−a−GCST90001486' on 'cNENs'
All
MR Estimate
Inverse variance weighted
MR Egger
Simple mode
Weighted median
Weighted mode
SNP effect on cNENs
SNP effect on || id:ebi−a−GCST90001486

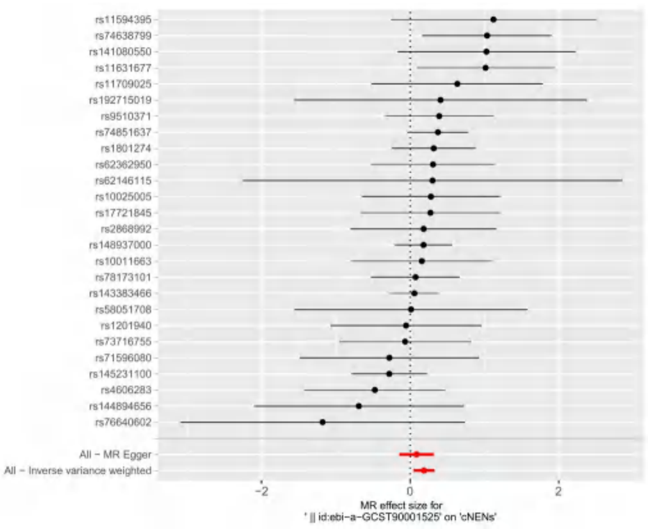
rs11594395
rs74638799
rs141080550
rs11631677
rs11709025
rs192715019
rs9510371
rs74851637
rs1801274
rs62362950
rs62146115
rs10025005
rs17721845
rs2868992
rs148937000
rs10011663
rs78173101
rs143383466
rs58051708
rs1201940
rs73716755
rs71596080
rs145231100
rs4606283
rs144894656
rs76640602
All − MR Egger
All − Inverse variance weighted
−2
0
2
MR effect size for
' || id:ebi−a−GCST90001525' on 'cNENs'

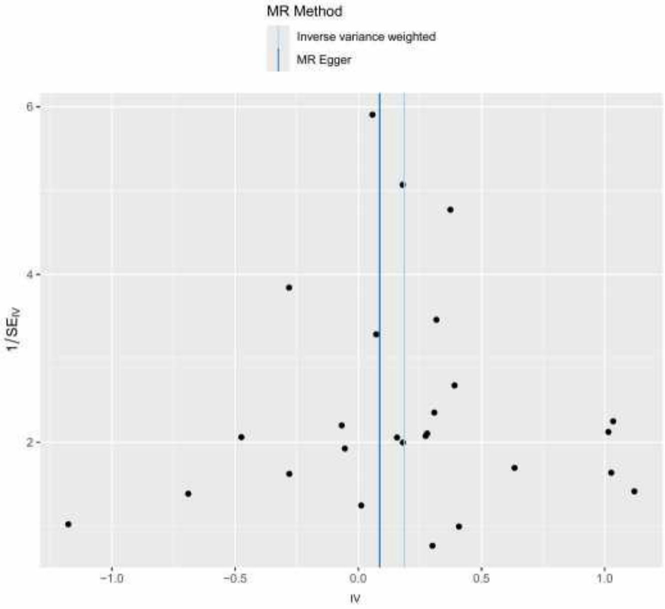
MR Method
Inverse variance weighted
MR Egger
1/SE$_{IV}$
6
4
2
−1.0
−0.5
0.0
0.5
1.0
IV

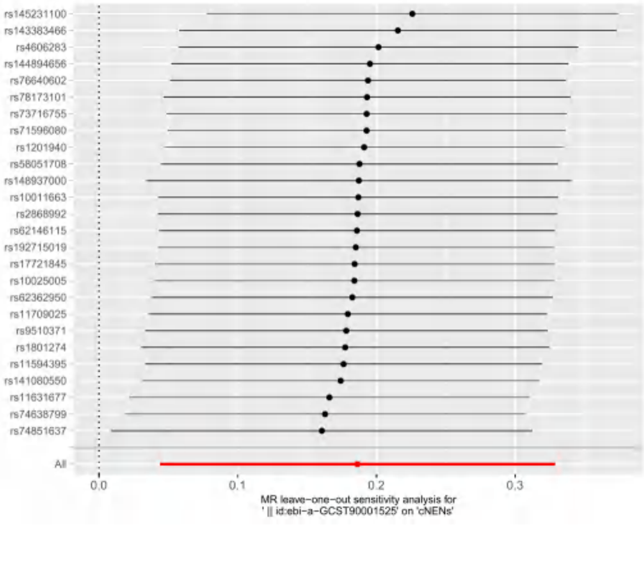
rs145231100
rs143383466
rs4606283
rs144894656
rs76640602
rs78173101
rs73716755
rs71596080
rs1201940
rs58051708
rs148937000
rs10011663
rs2868992
rs62146115
rs192715019
rs17721845
rs10025005
rs62362950
rs11709025
rs9510371
rs1801274
rs11594395
rs141080550
rs11631677
rs74638799
rs74851637
All
0.0
0.1
0.2
0.3
MR leave−one−out sensitivity analysis for
' || id:ebi−a−GCST90001525' on 'cNENs'

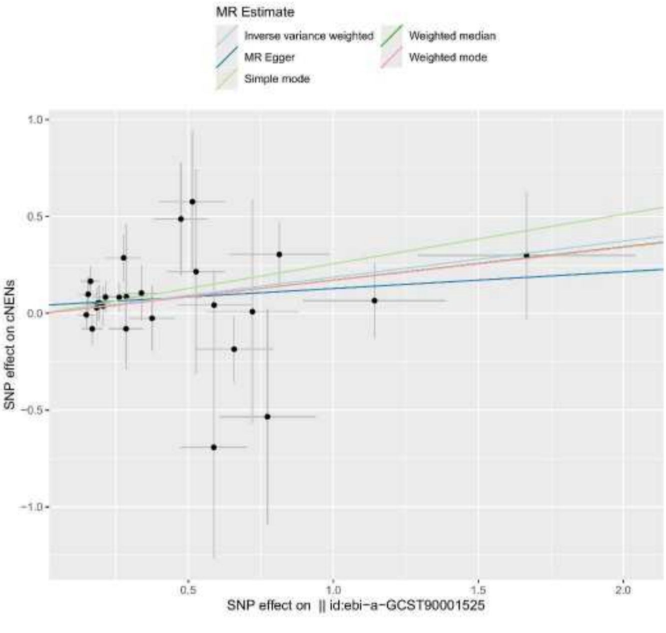
MR Estimate
Inverse variance weighted
MR Egger
Simple mode
Weighted median
Weighted mode
SNP effect on cNENs
1.0
0.5
0.0
−0.5
−1.0
0.5
1.0
1.5
2.0
SNP effect on || id:ebi−a−GCST90001525

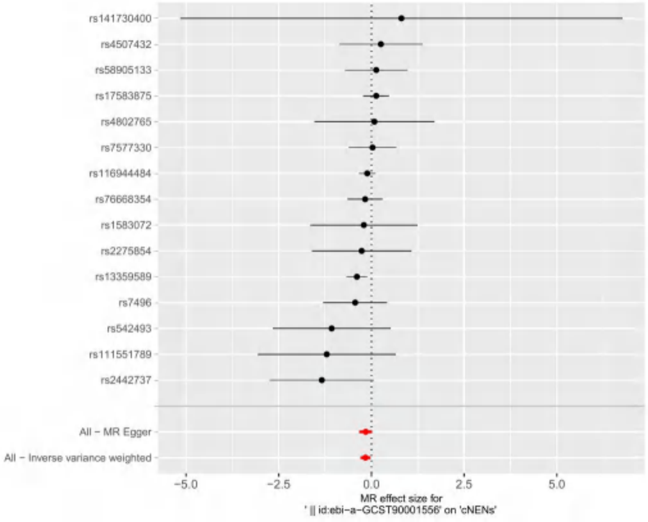
rs141730400
rs4507432
rs58905133
rs17583875
rs4802765
rs7577330
rs116944484
rs76668354
rs1583072
rs2275854
rs13359589
rs7496
rs542493
rs111551789
rs2442737
All − MR Egger
All − Inverse variance weighted
−5.0
−2.5
0.0
2.5
5.0
MR effect size for
' || id:ebi−a−GCST90001556' on 'cNENs'

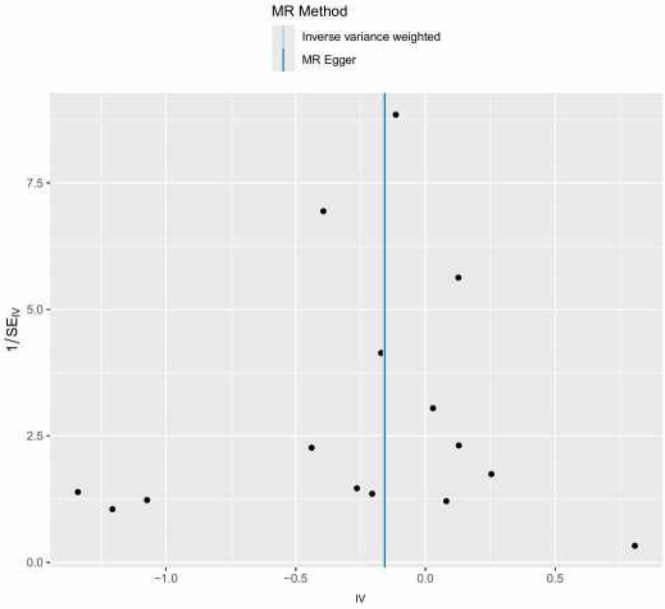
MR Method
Inverse variance weighted
MR Egger
1/SE_IV
7.5
5.0
2.5
0.0
−1.0
−0.5
0.0
0.5
IV

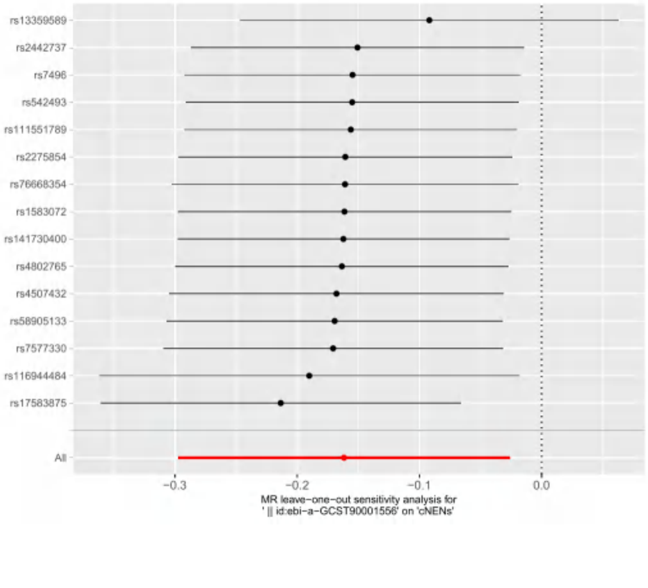
rs13359589
rs2442737
rs7496
rs542493
rs111551789
rs2275854
rs76668354
rs1583072
rs141730400
rs4802765
rs4507432
rs58905133
rs7577330
rs116944484
rs17583875
All
−0.3
−0.2
−0.1
0.0
MR leave−one−out sensitivity analysis for
' || id:ebi−a−GCST90001556' on 'cNENs'

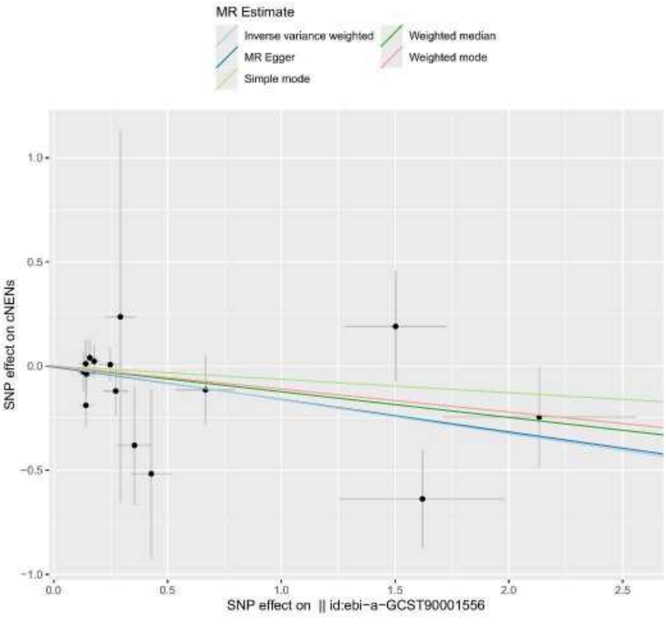
MR Estimate
Inverse variance weighted
MR Egger
Simple mode
Weighted median
Weighted mode
SNP effect on cNENs
1.0
0.5
0.0
−0.5
−1.0
0.0
0.5
1.0
1.5
2.0
2.5
SNP effect on || id:ebi−a−GCST90001556

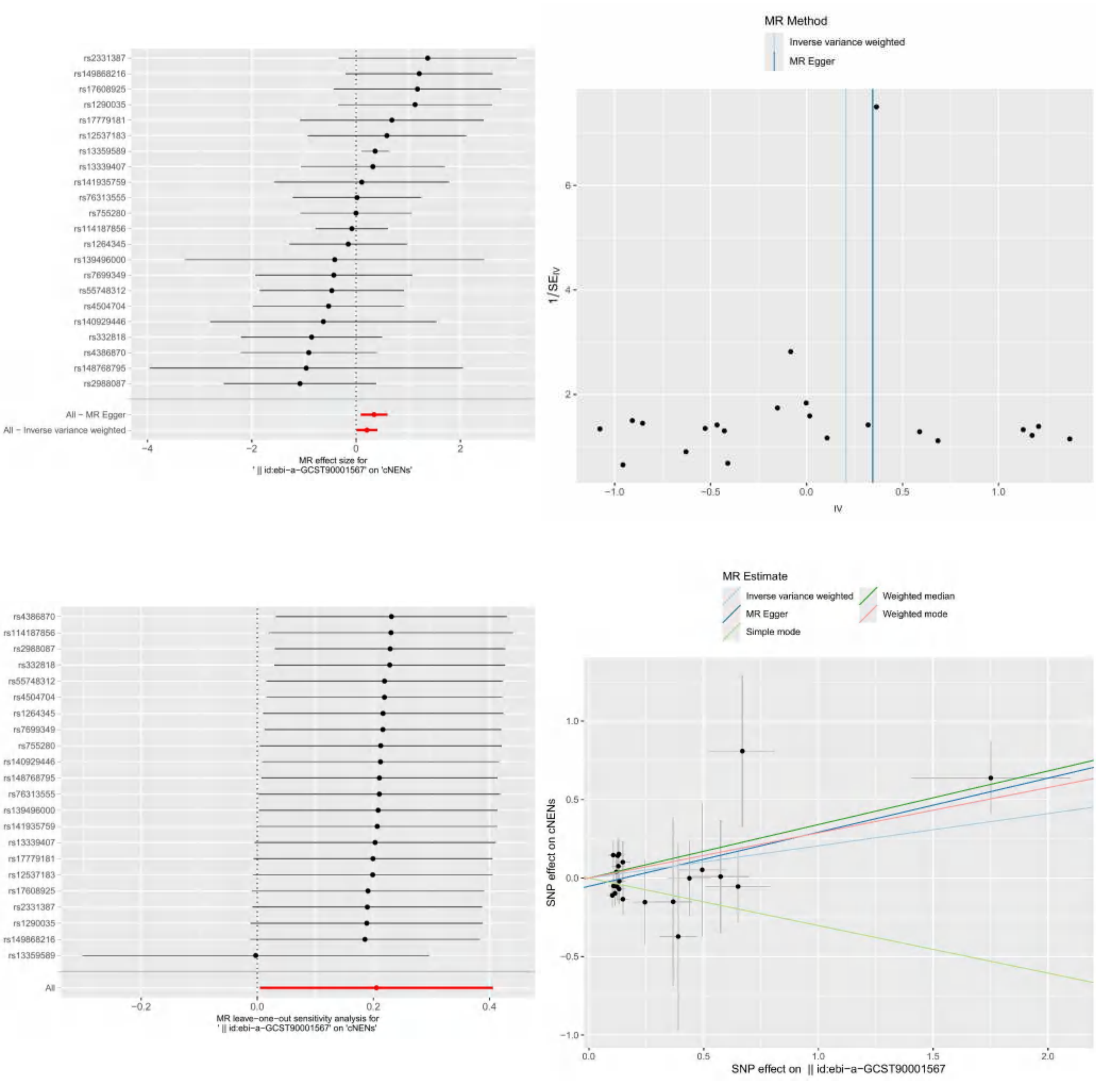
rs2331387
rs149868216
rs17608925
rs1290035
rs17779181
rs12537183
rs13359589
rs13339407
rs141935759
rs76313555
rs755280
rs114187856
rs1264345
rs139496000
rs7699349
rs55748312
rs4504704
rs140929446
rs332818
rs4386870
rs148768795
rs2988087
All − MR Egger
All − Inverse variance weighted
MR effect size for
' || id:ebi−a−GCST90001567' on 'cNENs'
MR Method
Inverse variance weighted
MR Egger
1/SE_IV
IV
rs4386870
rs114187856
rs2988087
rs332818
rs55748312
rs4504704
rs1264345
rs7699349
rs755280
rs140929446
rs148768795
rs76313555
rs139496000
rs141935759
rs13339407
rs17779181
rs12537183
rs17608925
rs2331387
rs1290035
rs149868216
rs13359589
All
MR leave−one−out sensitivity analysis for
' || id:ebi−a−GCST90001567' on 'cNENs'
MR Estimate
Inverse variance weighted
MR Egger
Simple mode
Weighted median
Weighted mode
SNP effect on cNENs
SNP effect on || id:ebi−a−GCST90001567

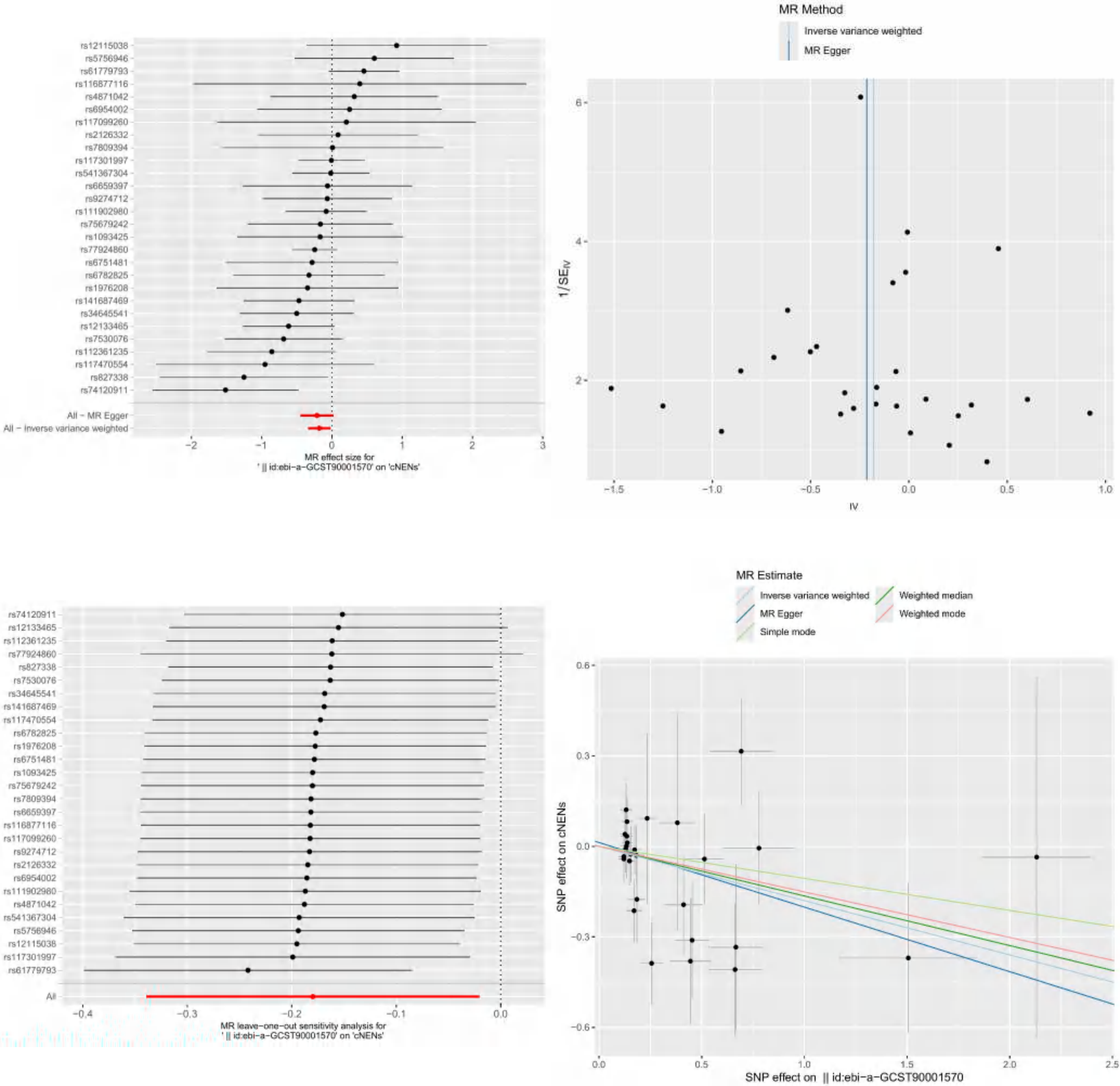
rs12115038
rs5756946
rs61779793
rs116877116
rs4871042
rs6954002
rs117099260
rs2126332
rs7809394
rs117301997
rs541367304
rs6659397
rs9274712
rs111902980
rs75679242
rs1093425
rs77924860
rs6751481
rs6782825
rs1976208
rs141687469
rs34645541
rs12133465
rs7530076
rs112361235
rs117470554
rs827338
rs74120911
All − MR Egger
All − Inverse variance weighted
−2
−1
0
1
2
3
MR effect size for
' || id:ebi−a−GCST90001570' on 'cNENs'
MR Method
Inverse variance weighted
MR Egger
1/SE$_{IV}$
6
4
2
IV
−1.5
−1.0
−0.5
0.0
0.5
1.0
rs74120911
rs12133465
rs112361235
rs77924860
rs827338
rs7530076
rs34645541
rs141687469
rs117470554
rs6782825
rs1976208
rs6751481
rs1093425
rs75679242
rs7809394
rs6659397
rs116877116
rs117099260
rs9274712
rs2126332
rs6954002
rs111902980
rs4871042
rs541367304
rs5756946
rs12115038
rs117301997
rs61779793
All
−0.4
−0.3
−0.2
−0.1
0.0
MR leave−one−out sensitivity analysis for
' || id:ebi−a−GCST90001570' on 'cNENs'
MR Estimate
Inverse variance weighted
MR Egger
Simple mode
Weighted median
Weighted mode
SNP effect on cNENs
0.6
0.3
0.0
−0.3
−0.6
0.0
0.5
1.0
1.5
2.0
2.5
SNP effect on || id:ebi−a−GCST90001570

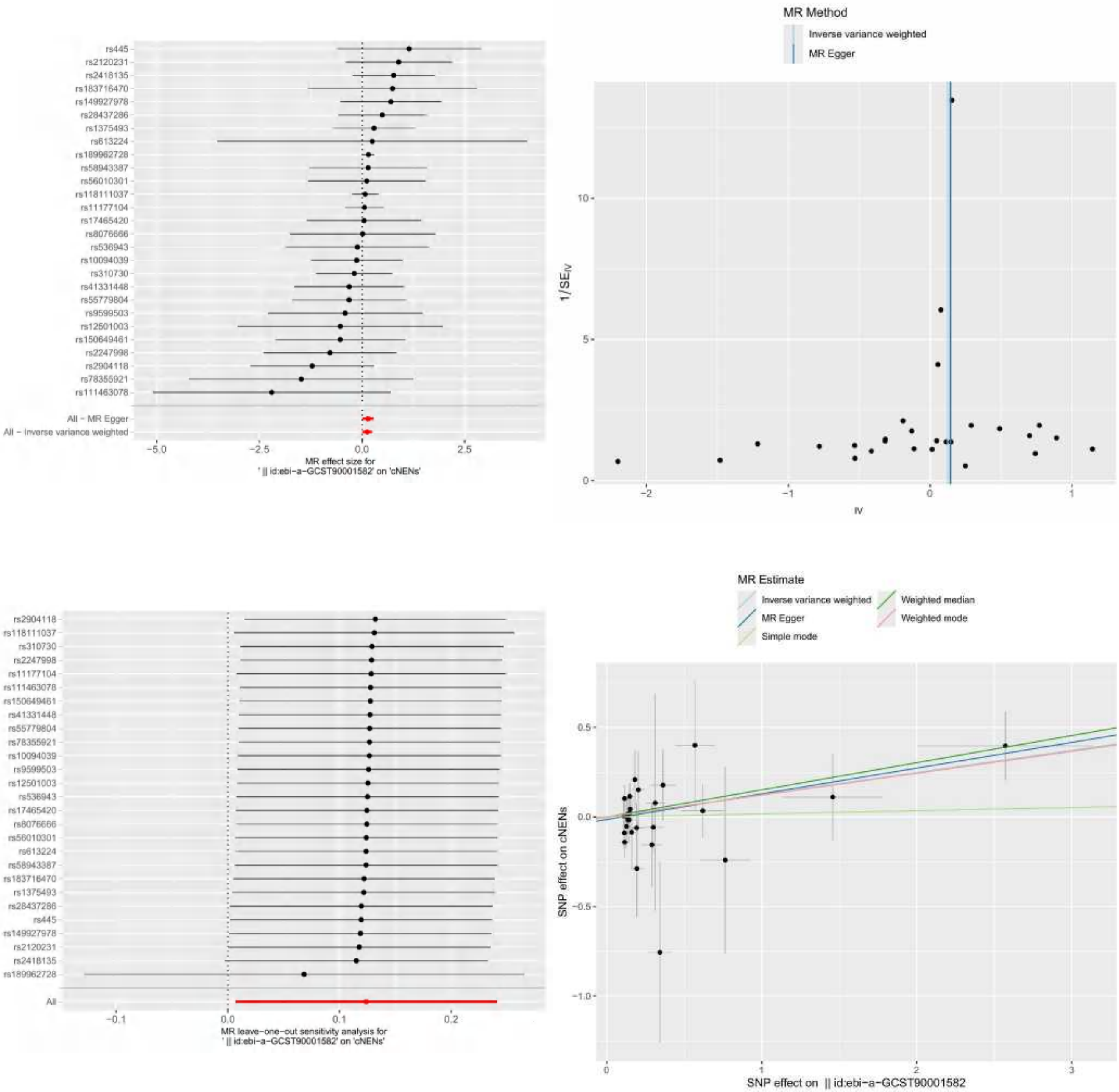

rs445
rs2120231
rs2418135
rs183716470
rs149927978
rs28437286
rs1375493
rs613224
rs189962728
rs58943387
rs56010301
rs118111037
rs11177104
rs17465420
rs8076666
rs536943
rs10094039
rs310730
rs41331448
rs55779804
rs9599503
rs12501003
rs150649461
rs2247998
rs2904118
rs78355921
rs111463078
All − MR Egger
All − Inverse variance weighted
−5.0
−2.5
0.0
2.5
MR effect size for
' || id:ebi−a−GCST90001582' on 'cNENs'
MR Method
Inverse variance weighted
MR Egger
1/SE$_{IV}$
IV
MR leave−one−out sensitivity analysis for
' || id:ebi−a−GCST90001582' on 'cNENs'
MR Estimate
Inverse variance weighted
MR Egger
Simple mode
Weighted median
Weighted mode
SNP effect on cNENs
SNP effect on || id:ebi−a−GCST90001582

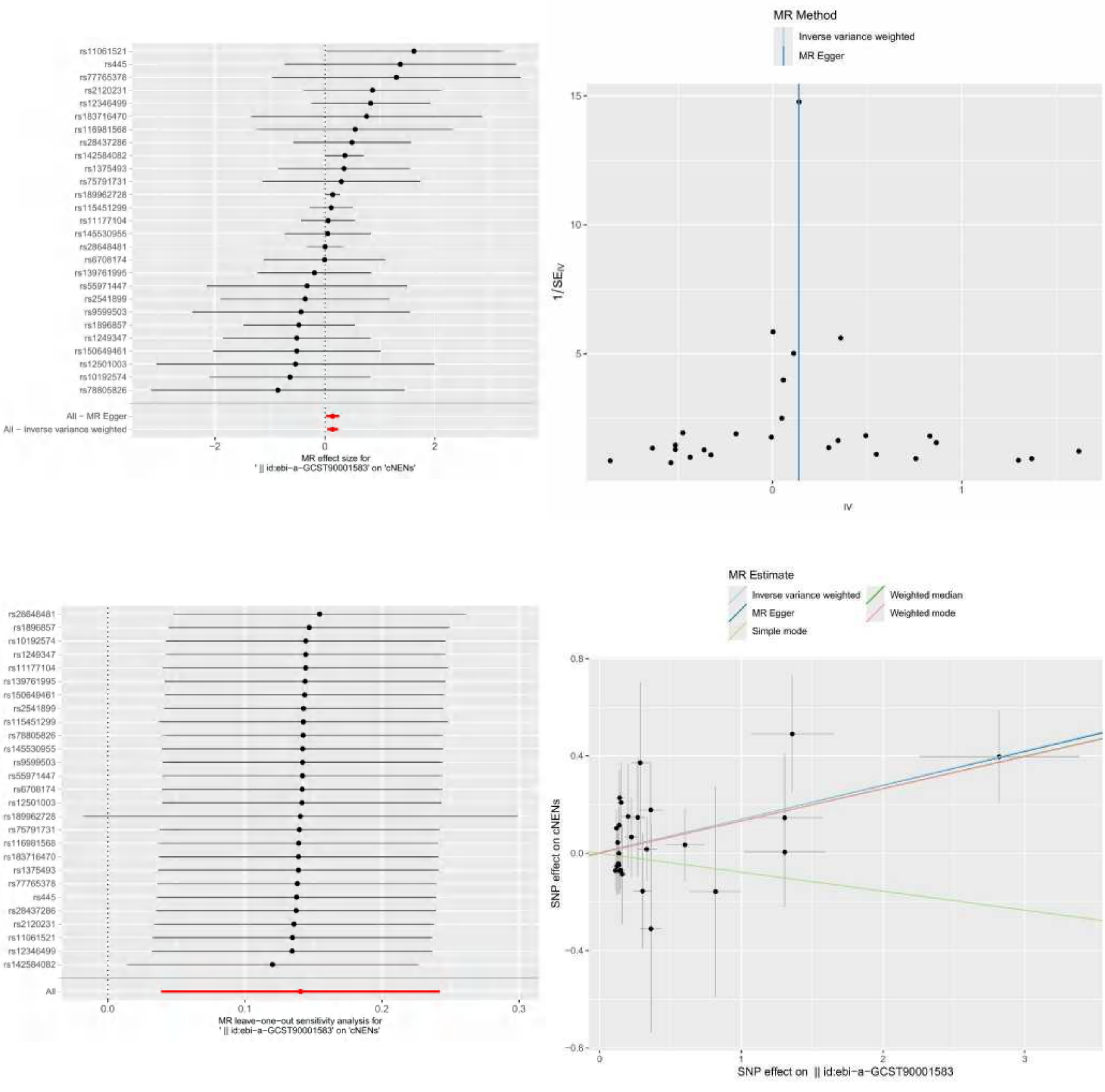
rs11061521
rs445
rs77765378
rs2120231
rs12346499
rs183716470
rs116981568
rs28437286
rs142584082
rs1375493
rs75791731
rs189962728
rs115451299
rs11177104
rs145530955
rs28648481
rs6708174
rs139761995
rs55971447
rs2541899
rs9599503
rs1896857
rs1249347
rs150649461
rs12501003
rs10192574
rs78805826
All − MR Egger
All − Inverse variance weighted
MR effect size for
' || id:ebi−a−GCST90001583' on 'cNENs'
MR Method
Inverse variance weighted
MR Egger
1/SE$_{IV}$
IV
MR leave−one−out sensitivity analysis for
' || id:ebi−a−GCST90001583' on 'cNENs'
All
MR Estimate
Inverse variance weighted
MR Egger
Simple mode
Weighted median
Weighted mode
SNP effect on cNENs
SNP effect on || id:ebi−a−GCST90001583

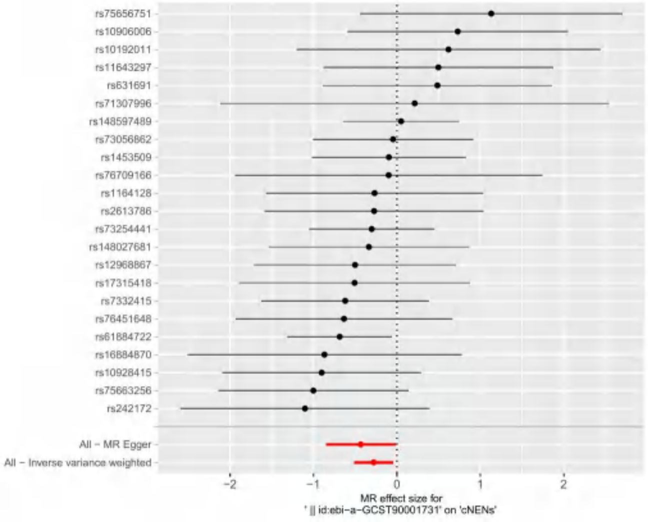
rs75656751
rs10906006
rs10192011
rs11643297
rs631691
rs71307996
rs148597489
rs73056862
rs1453509
rs76709166
rs1164128
rs2613786
rs73254441
rs148027681
rs12968867
rs17315418
rs7332415
rs76451648
rs61884722
rs16884870
rs10928415
rs75663256
rs242172
All − MR Egger
All − Inverse variance weighted
−2
−1
0
1
2
MR effect size for
' || id:ebi−a−GCST90001731' on 'cNENs'

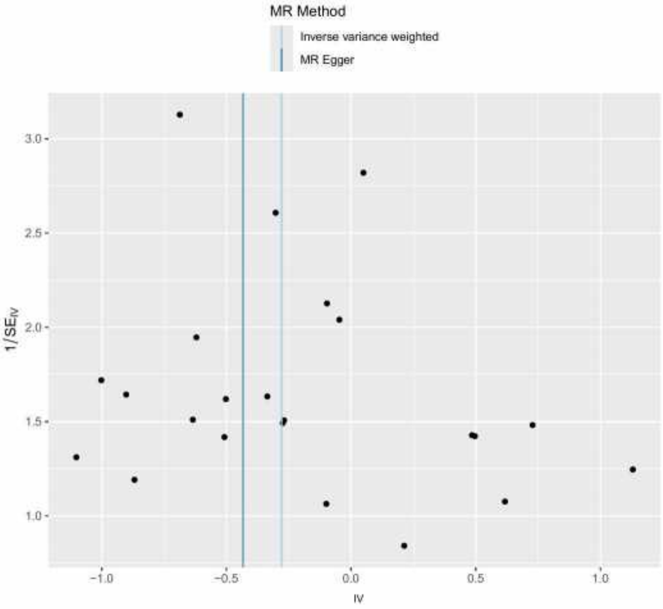
MR Method
Inverse variance weighted
MR Egger
$1/SE_{IV}$
3.0
2.5
2.0
1.5
1.0
−1.0
−0.5
0.0
0.5
1.0
IV

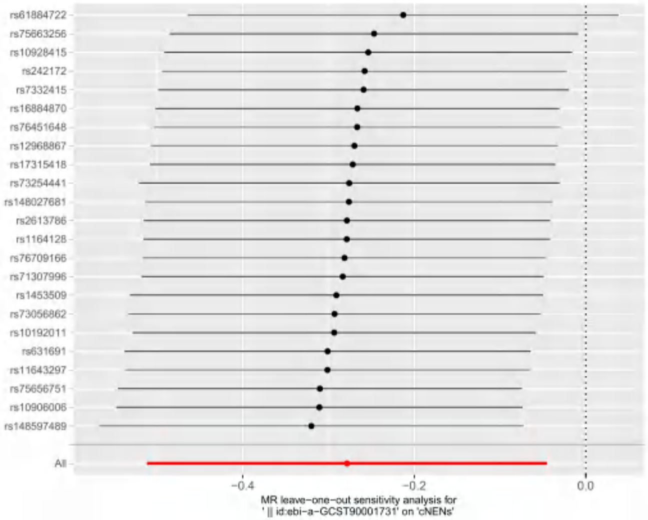
rs61884722
rs75663256
rs10928415
rs242172
rs7332415
rs16884870
rs76451648
rs12968867
rs17315418
rs73254441
rs148027681
rs2613786
rs1164128
rs76709166
rs71307996
rs1453509
rs73056862
rs10192011
rs631691
rs11643297
rs75656751
rs10906006
rs148597489
All
−0.4
−0.2
0.0
MR leave−one−out sensitivity analysis for
' || id:ebi−a−GCST90001731' on 'cNENs'

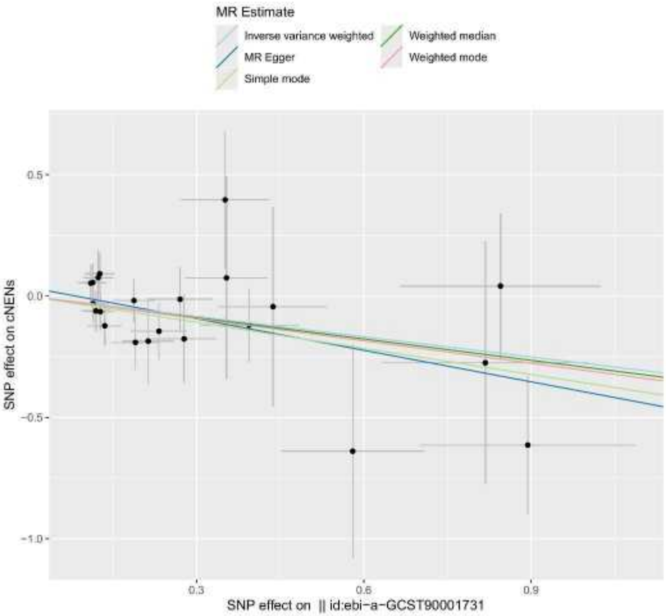
MR Estimate
Inverse variance weighted
MR Egger
Simple mode
Weighted median
Weighted mode
SNP effect on cNENs
0.5
0.0
−0.5
−1.0
0.3
0.6
0.9
SNP effect on || id:ebi−a−GCST90001731

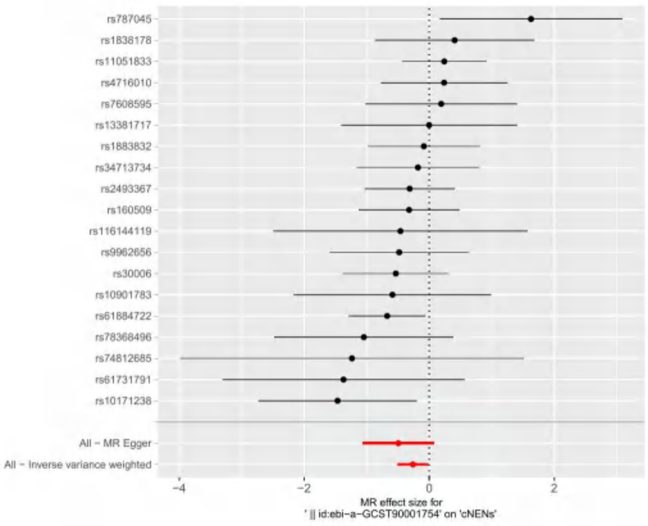
rs787045
rs1838178
rs11051833
rs4716010
rs7608595
rs13381717
rs1883832
rs34713734
rs2493367
rs160509
rs116144119
rs9962656
rs30006
rs10901783
rs61884722
rs78368496
rs74812685
rs61731791
rs10171238
All − MR Egger
All − Inverse variance weighted
−4
−2
0
2
MR effect size for
' || id:ebi−a−GCST90001754' on 'cNENs'

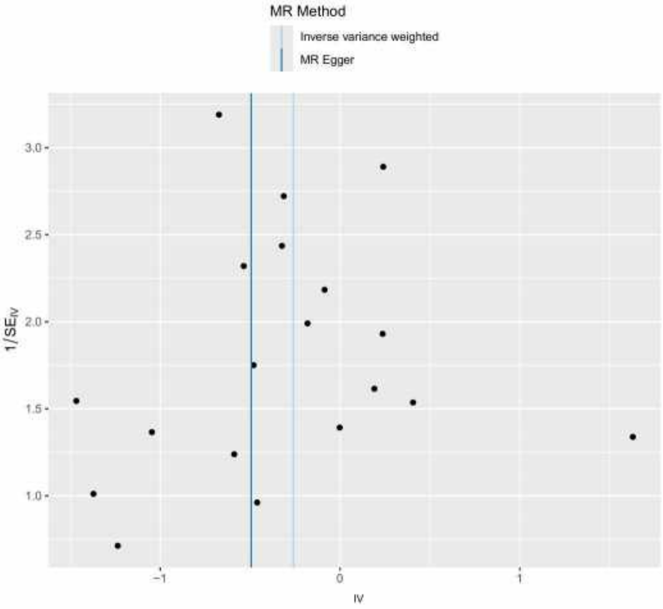
MR Method
Inverse variance weighted
MR Egger
$1/SE_{IV}$
3.0
2.5
2.0
1.5
1.0
−1
0
1
IV

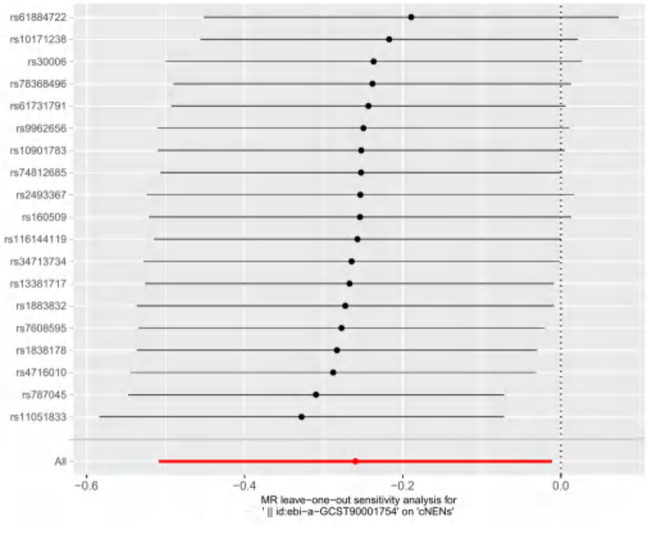
rs61884722
rs10171238
rs30006
rs78368496
rs61731791
rs9962656
rs10901783
rs74812685
rs2493367
rs160509
rs116144119
rs34713734
rs13381717
rs1883832
rs7608595
rs1838178
rs4716010
rs787045
rs11051833
All
−0.6
−0.4
−0.2
0.0
MR leave−one−out sensitivity analysis for
' || id:ebi−a−GCST90001754' on 'cNENs'

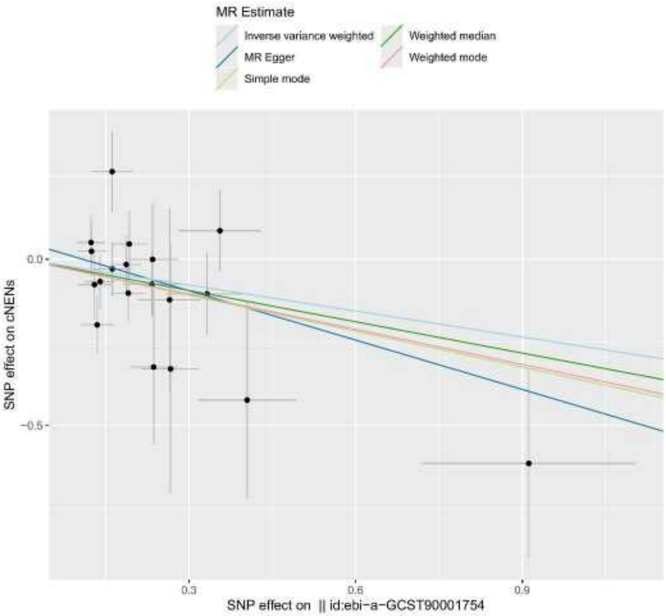
MR Estimate
Inverse variance weighted
MR Egger
Simple mode
Weighted median
Weighted mode
SNP effect on cNENs
0.0
−0.5
0.3
0.6
0.9
SNP effect on || id:ebi−a−GCST90001754

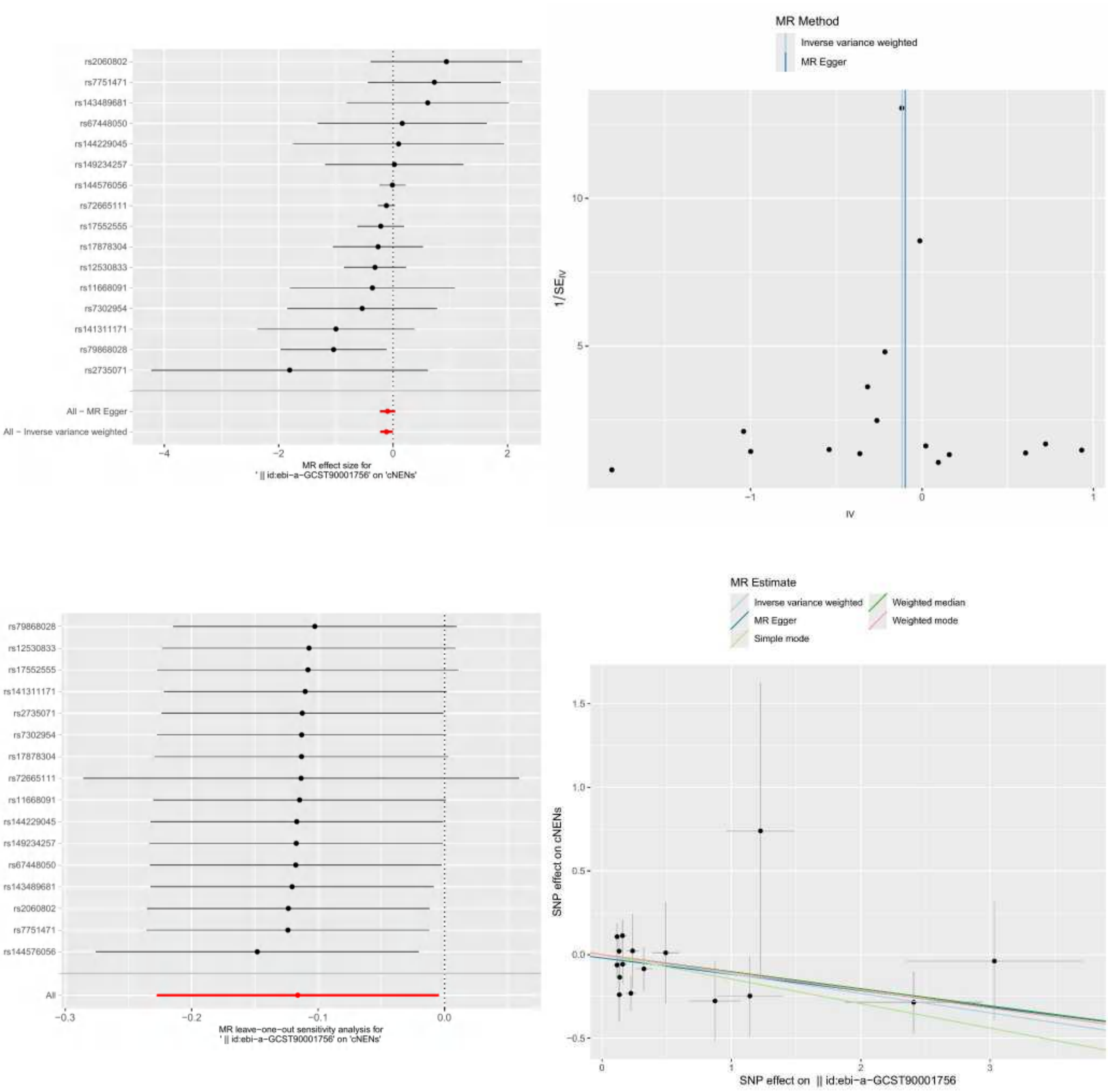

rs2060802
rs7751471
rs143489681
rs67448050
rs144229045
rs149234257
rs144576056
rs72665111
rs17552555
rs17878304
rs12530833
rs11668091
rs7302954
rs141311171
rs79868028
rs2735071
All − MR Egger
All − Inverse variance weighted
−4
−2
0
2
MR effect size for
' || id:ebi−a−GCST90001756' on 'cNENs'
MR Method
Inverse variance weighted
MR Egger
1/SE$_{IV}$
10
5
−1
0
1
IV
rs79868028
rs12530833
rs17552555
rs141311171
rs2735071
rs7302954
rs17878304
rs72665111
rs11668091
rs144229045
rs149234257
rs67448050
rs143489681
rs2060802
rs7751471
rs144576056
All
−0.3
−0.2
−0.1
0.0
MR leave−one−out sensitivity analysis for
' || id:ebi−a−GCST90001756' on 'cNENs'
MR Estimate
Inverse variance weighted
MR Egger
Simple mode
Weighted median
Weighted mode
SNP effect on cNENs
1.5
1.0
0.5
0.0
−0.5
0
1
2
3
SNP effect on || id:ebi−a−GCST90001756

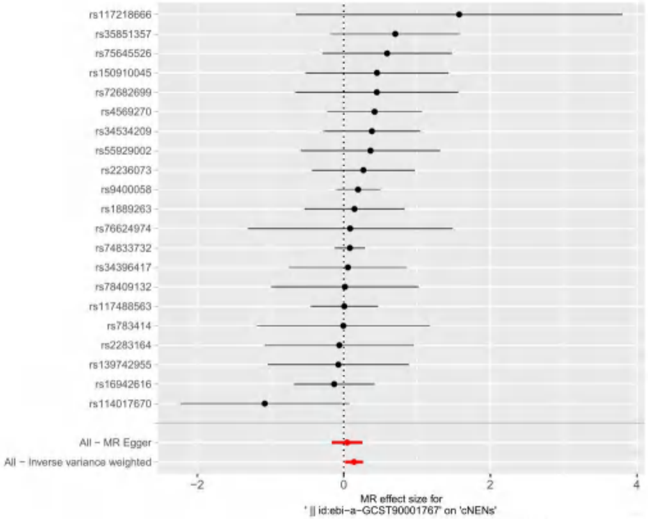

rs117218666
rs35851357
rs75645526
rs150910045
rs72682699
rs4569270
rs34534209
rs55929002
rs2236073
rs9400058
rs1889263
rs76624974
rs74833732
rs34396417
rs78409132
rs117488563
rs783414
rs2283164
rs139742955
rs16942616
rs114017670
All − MR Egger
All − Inverse variance weighted
−2
0
2
4
MR effect size for
' || id:ebi−a−GCST90001767' on 'cNENs'

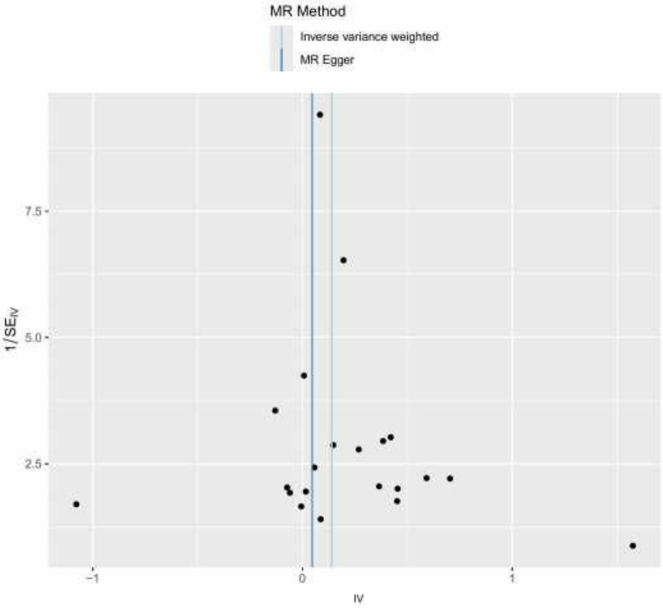

MR Method
Inverse variance weighted
MR Egger
7.5
5.0
2.5
1/SE_IV
−1
0
1
IV

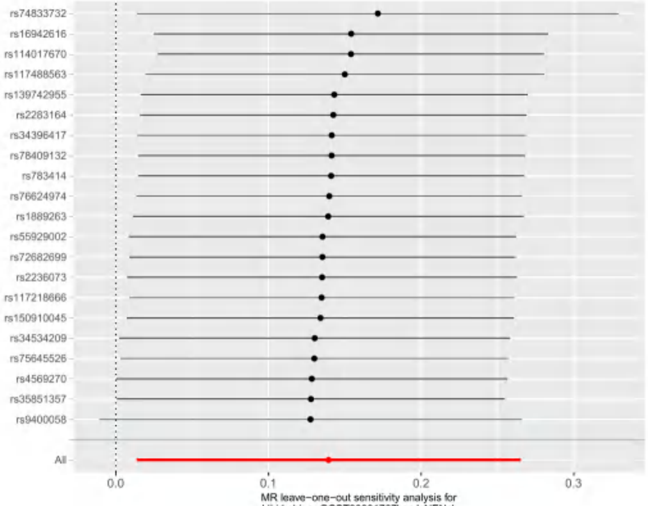

rs74833732
rs16942616
rs114017670
rs117488563
rs139742955
rs2283164
rs34396417
rs78409132
rs783414
rs76624974
rs1889263
rs55929002
rs72682699
rs2236073
rs117218666
rs150910045
rs34534209
rs75645526
rs4569270
rs35851357
rs9400058
All
0.0
0.1
0.2
0.3
MR leave−one−out sensitivity analysis for
' || id:ebi−a−GCST90001767' on 'cNENs'

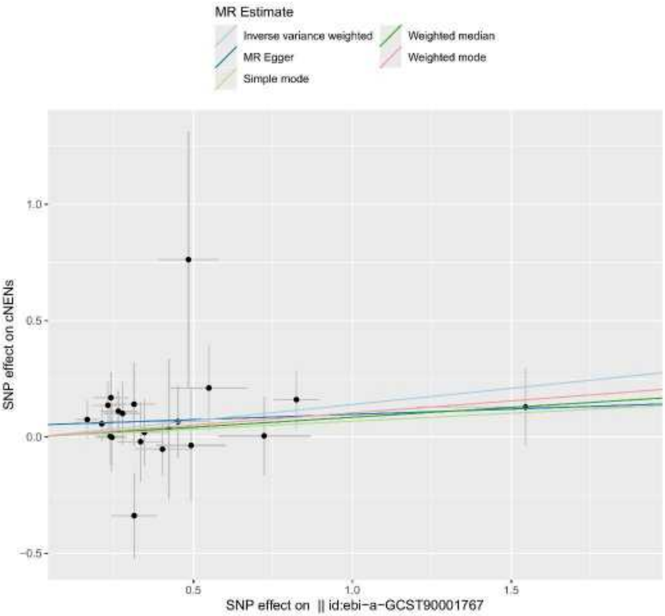

MR Estimate
Inverse variance weighted
MR Egger
Simple mode
Weighted median
Weighted mode
1.0
0.5
0.0
−0.5
SNP effect on cNENs
0.5
1.0
1.5
SNP effect on || id:ebi−a−GCST90001767

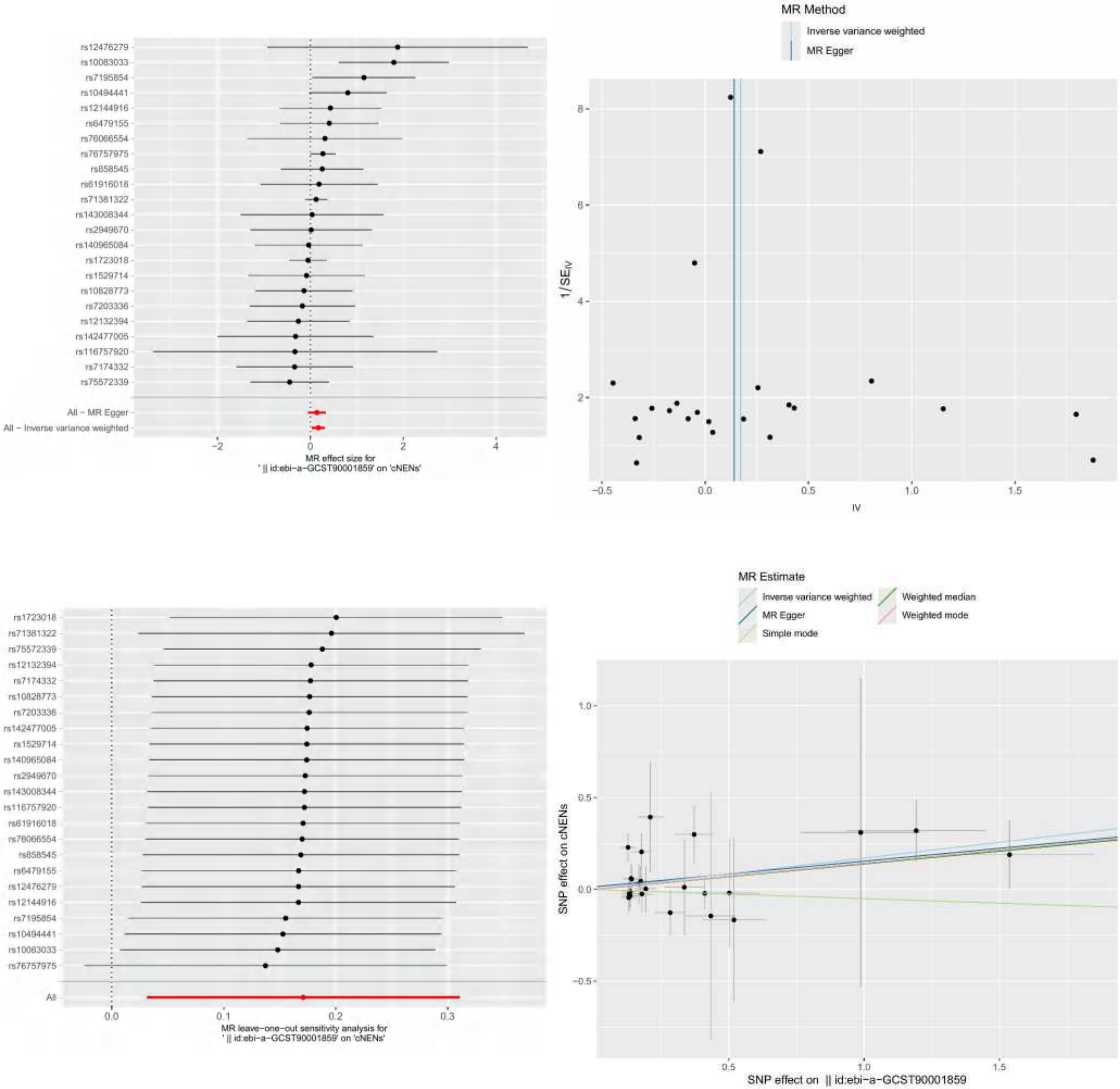
rs12476279
rs10083033
rs7195854
rs10494441
rs12144916
rs6479155
rs76066554
rs76757975
rs858545
rs61916018
rs71381322
rs143008344
rs2949670
rs140965084
rs1723018
rs1529714
rs10828773
rs7203336
rs12132394
rs142477005
rs116757920
rs7174332
rs75572339
All − MR Egger
All − Inverse variance weighted
MR effect size for
' || id:ebi−a−GCST90001859' on 'cNENs'
MR Method
Inverse variance weighted
MR Egger
1/SE$_{IV}$
IV
rs1723018
rs71381322
rs75572339
rs12132394
rs7174332
rs10828773
rs7203336
rs142477005
rs1529714
rs140965084
rs2949670
rs143008344
rs116757920
rs61916018
rs76066554
rs858545
rs6479155
rs12476279
rs12144916
rs7195854
rs10494441
rs10083033
rs76757975
All
MR leave−one−out sensitivity analysis for
' || id:ebi−a−GCST90001859' on 'cNENs'
MR Estimate
Inverse variance weighted
MR Egger
Simple mode
Weighted median
Weighted mode
SNP effect on cNENs
SNP effect on || id:ebi−a−GCST90001859

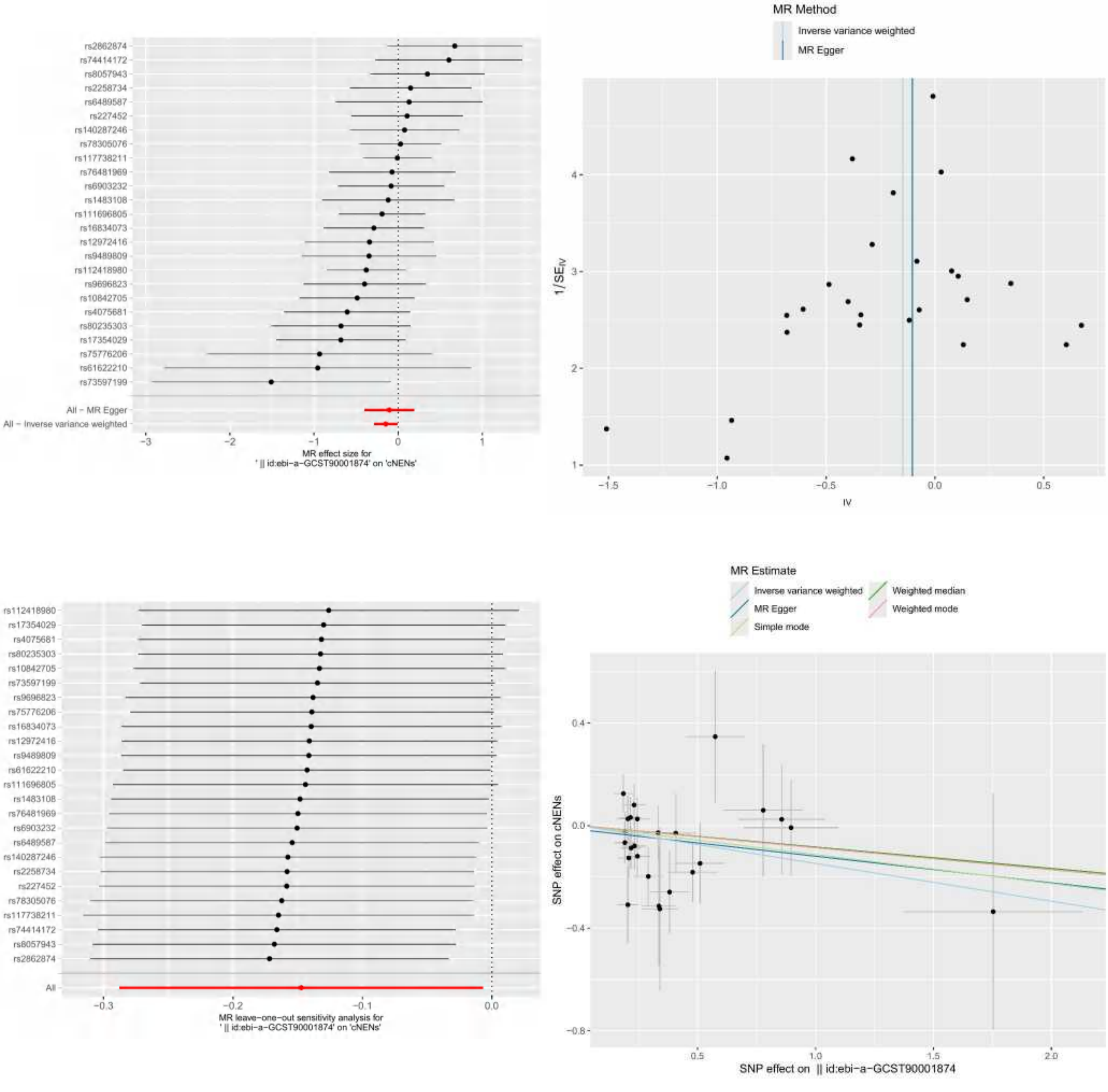

rs2862874
rs74414172
rs8057943
rs2258734
rs6489587
rs227452
rs140287246
rs78305076
rs117738211
rs76481969
rs6903232
rs1483108
rs111696805
rs16834073
rs12972416
rs9489809
rs112418980
rs9696823
rs10842705
rs4075681
rs80235303
rs17354029
rs75776206
rs61622210
rs73597199
All − MR Egger
All − Inverse variance weighted
MR effect size for
' || id:ebi−a−GCST90001874' on 'cNENs'
MR Method
Inverse variance weighted
MR Egger
1/SE$_{IV}$
IV
MR leave−one−out sensitivity analysis for
' || id:ebi−a−GCST90001874' on 'cNENs'
All
MR Estimate
Inverse variance weighted
MR Egger
Simple mode
Weighted median
Weighted mode
SNP effect on cNENs
SNP effect on || id:ebi−a−GCST90001874

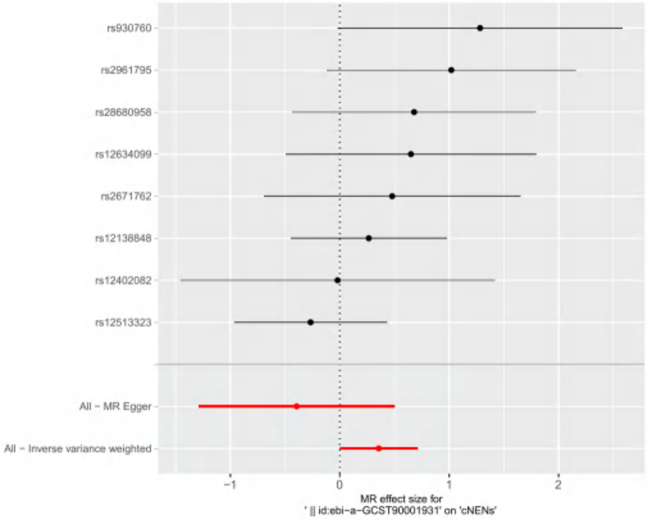
rs930760
rs2961795
rs28680958
rs12634099
rs2671762
rs12138848
rs12402082
rs12513323
All − MR Egger
All − Inverse variance weighted
−1
0
1
2
MR effect size for
' || id:ebi−a−GCST90001931' on 'cNENs'

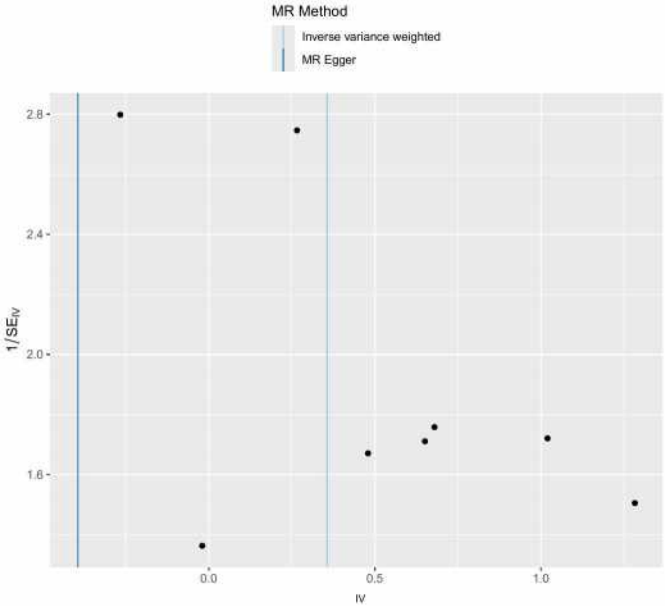
MR Method
Inverse variance weighted
MR Egger
2.8
2.4
2.0
1.6
$1/SE_{IV}$
0.0
0.5
1.0
IV

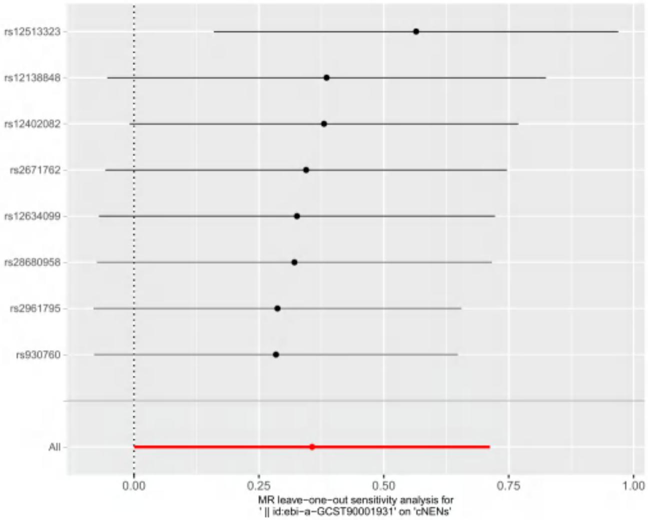
rs12513323
rs12138848
rs12402082
rs2671762
rs12634099
rs28680958
rs2961795
rs930760
All
0.00
0.25
0.50
0.75
1.00
MR leave−one−out sensitivity analysis for
' || id:ebi−a−GCST90001931' on 'cNENs'

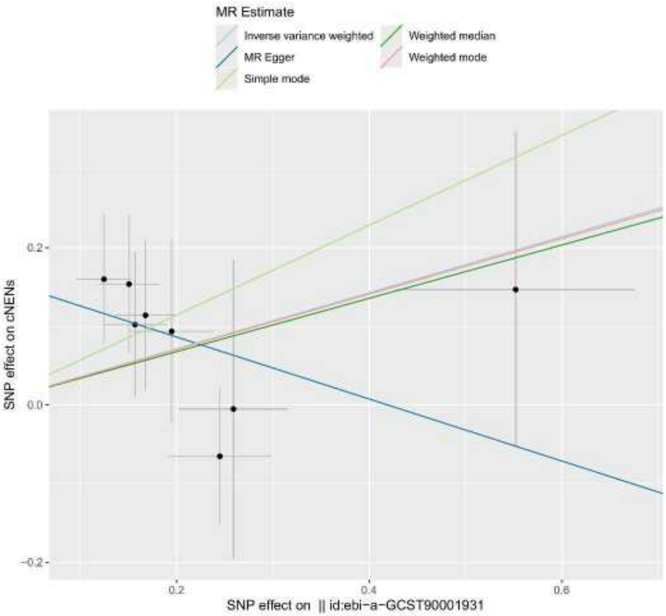
MR Estimate
Inverse variance weighted
MR Egger
Simple mode
Weighted median
Weighted mode
0.2
0.0
−0.2
SNP effect on cNENs
0.2
0.4
0.6
SNP effect on || id:ebi−a−GCST90001931

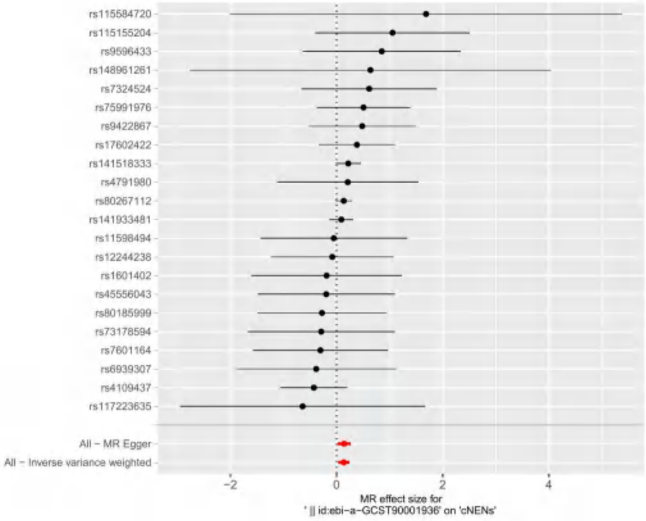
rs115584720
rs115155204
rs9596433
rs148961261
rs7324524
rs75991976
rs9422867
rs17602422
rs141518333
rs4791980
rs80267112
rs141933481
rs11598494
rs12244238
rs1601402
rs45556043
rs80185999
rs73178594
rs7601164
rs6939307
rs4109437
rs117223635
All – MR Egger
All – Inverse variance weighted
−2
0
2
4
MR effect size for
' || id:ebi−a−GCST90001936' on 'cNENs'

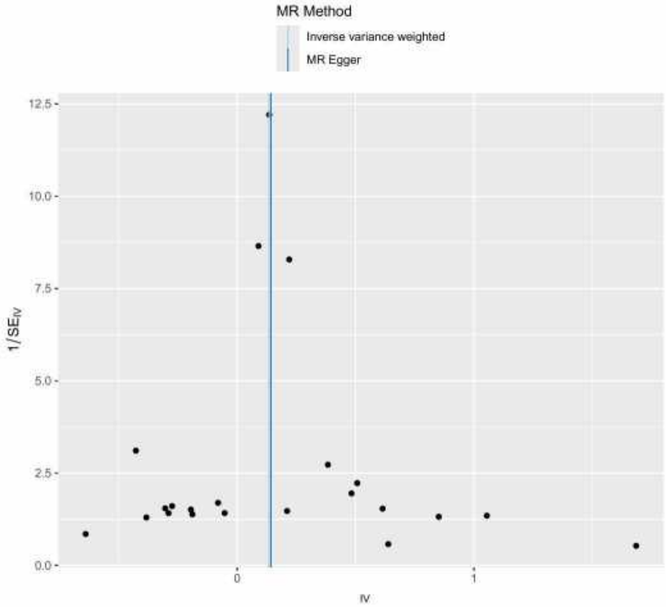
MR Method
Inverse variance weighted
MR Egger
12.5
10.0
7.5
5.0
2.5
0.0
$1/SE_{IV}$
0
1
IV

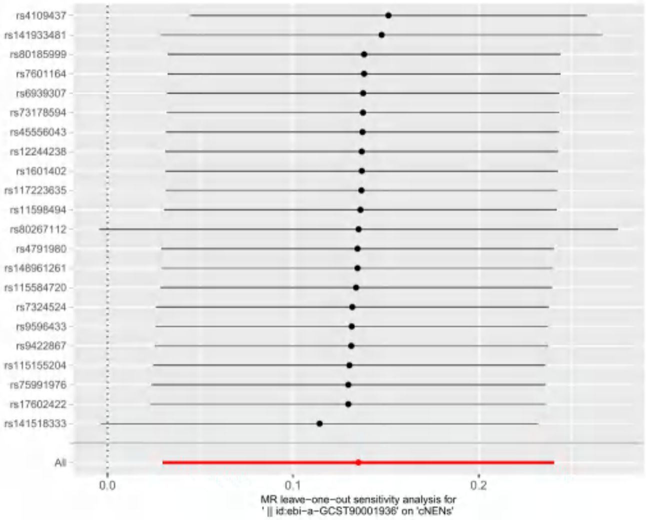
rs4109437
rs141933481
rs80185999
rs7601164
rs6939307
rs73178594
rs45556043
rs12244238
rs1601402
rs117223635
rs11598494
rs80267112
rs4791980
rs148961261
rs115584720
rs7324524
rs9596433
rs9422867
rs115155204
rs75991976
rs17602422
rs141518333
All
0.0
0.1
0.2
MR leave−one−out sensitivity analysis for
' || id:ebi−a−GCST90001936' on 'cNENs'

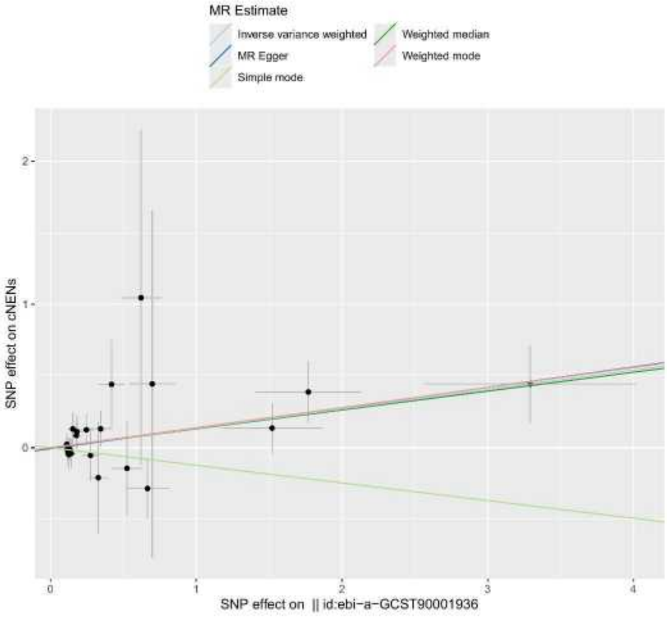
MR Estimate
Inverse variance weighted
MR Egger
Simple mode
Weighted median
Weighted mode
SNP effect on cNENs
2
1
0
0
1
2
3
4
SNP effect on || id:ebi−a−GCST90001936

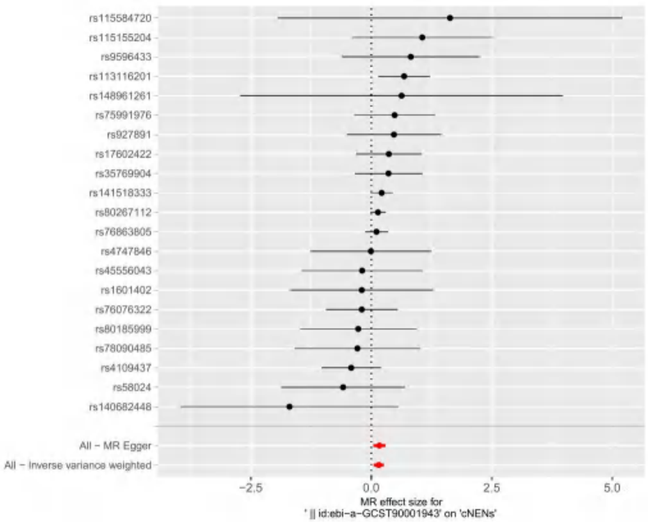
rs115584720
rs115155204
rs9596433
rs113116201
rs148961261
rs75991976
rs927891
rs17602422
rs35769904
rs141518333
rs80267112
rs76863805
rs4747846
rs45556043
rs1601402
rs76076322
rs80185999
rs78090485
rs4109437
rs58024
rs140682448
All − MR Egger
All − Inverse variance weighted
−2.5
0.0
2.5
5.0
MR effect size for
' || id:ebi−a−GCST90001943' on 'cNENs'

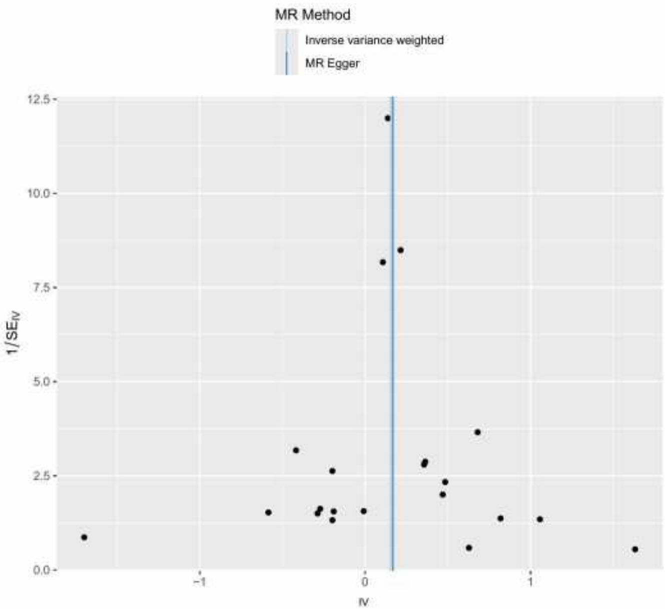
MR Method
Inverse variance weighted
MR Egger
12.5
10.0
7.5
5.0
2.5
0.0
$1/SE_{IV}$
−1
0
1
IV

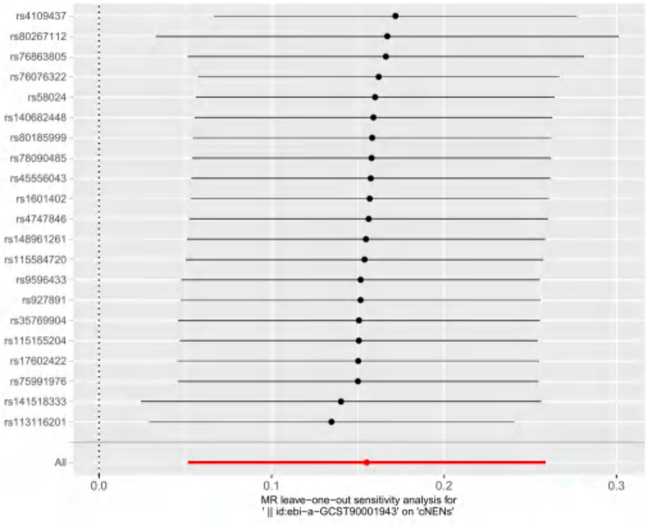
rs4109437
rs80267112
rs76863805
rs76076322
rs58024
rs140682448
rs80185999
rs78090485
rs45556043
rs1601402
rs4747846
rs148961261
rs115584720
rs9596433
rs927891
rs35769904
rs115155204
rs17602422
rs75991976
rs141518333
rs113116201
All
0.0
0.1
0.2
0.3
MR leave−one−out sensitivity analysis for
' || id:ebi−a−GCST90001943' on 'cNENs'

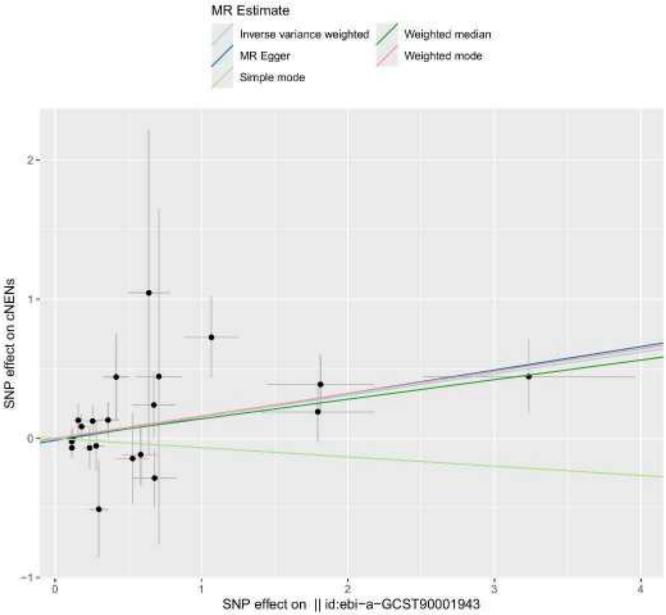
MR Estimate
Inverse variance weighted
MR Egger
Simple mode
Weighted median
Weighted mode
2
1
0
−1
SNP effect on cNENs
0
1
2
3
4
SNP effect on || id:ebi−a−GCST90001943

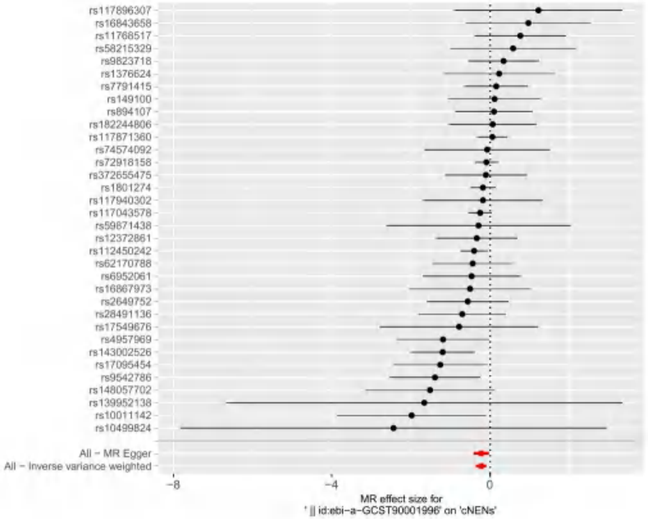
rs117896307
rs16843658
rs11768517
rs58215329
rs9823718
rs1376624
rs7791415
rs149100
rs894107
rs182244806
rs117871360
rs74574092
rs72918158
rs372655475
rs1801274
rs117940302
rs117043578
rs59871438
rs12372861
rs112450242
rs62170788
rs6952061
rs16867973
rs2649752
rs28491136
rs17549676
rs4957969
rs143002526
rs17095454
rs9542786
rs148057702
rs139952138
rs10011142
rs10499824
All − MR Egger
All − Inverse variance weighted
−8
−4
0
MR effect size for
' || id:ebi−a−GCST90001996' on 'cNENs'

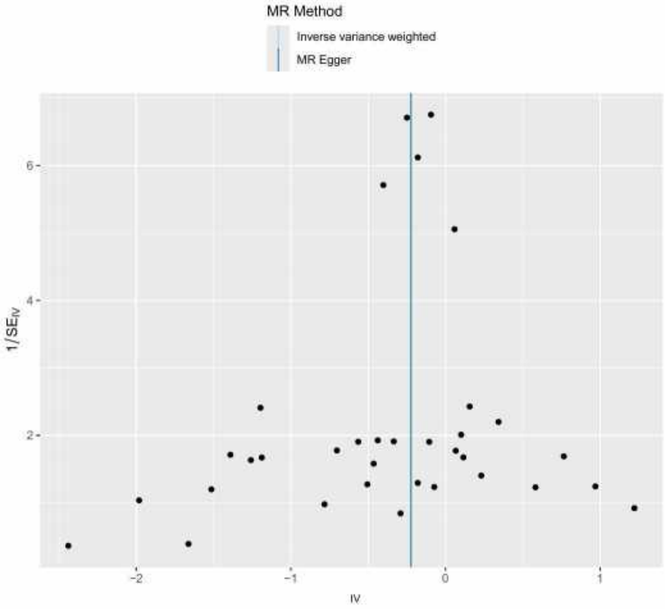
MR Method
Inverse variance weighted
MR Egger
$1/SE_{IV}$
6
4
2
−2
−1
0
1
IV

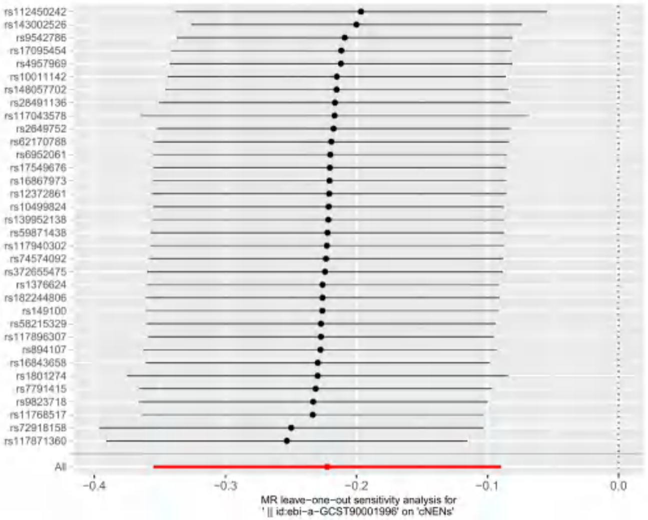
rs112450242
rs143002526
rs9542786
rs17095454
rs4957969
rs10011142
rs148057702
rs28491136
rs117043578
rs2649752
rs62170788
rs6952061
rs17549676
rs16867973
rs12372861
rs10499824
rs139952138
rs59871438
rs117940302
rs74574092
rs372655475
rs1376624
rs182244806
rs149100
rs58215329
rs117896307
rs894107
rs16843658
rs1801274
rs7791415
rs9823718
rs11768517
rs72918158
rs117871360
All
−0.4
−0.3
−0.2
−0.1
0.0
MR leave−one−out sensitivity analysis for
' || id:ebi−a−GCST90001996' on 'cNENs'

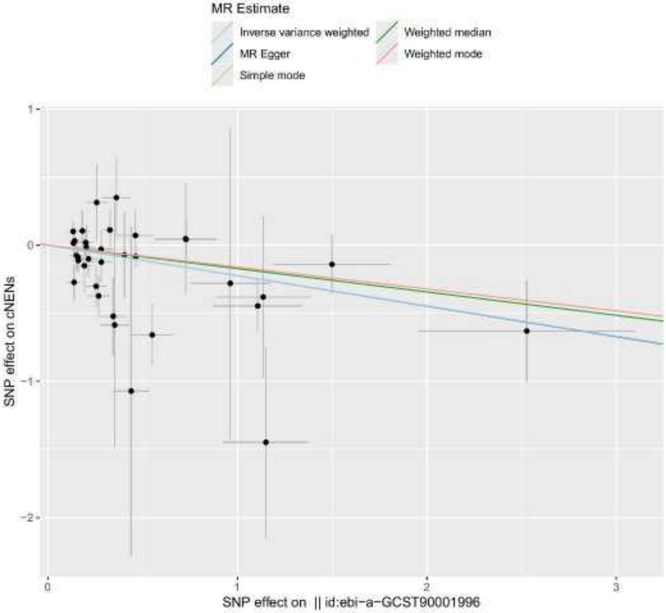
MR Estimate
Inverse variance weighted
MR Egger
Simple mode
Weighted median
Weighted mode
SNP effect on cNENs
1
0
−1
−2
0
1
2
3
SNP effect on || id:ebi−a−GCST90001996

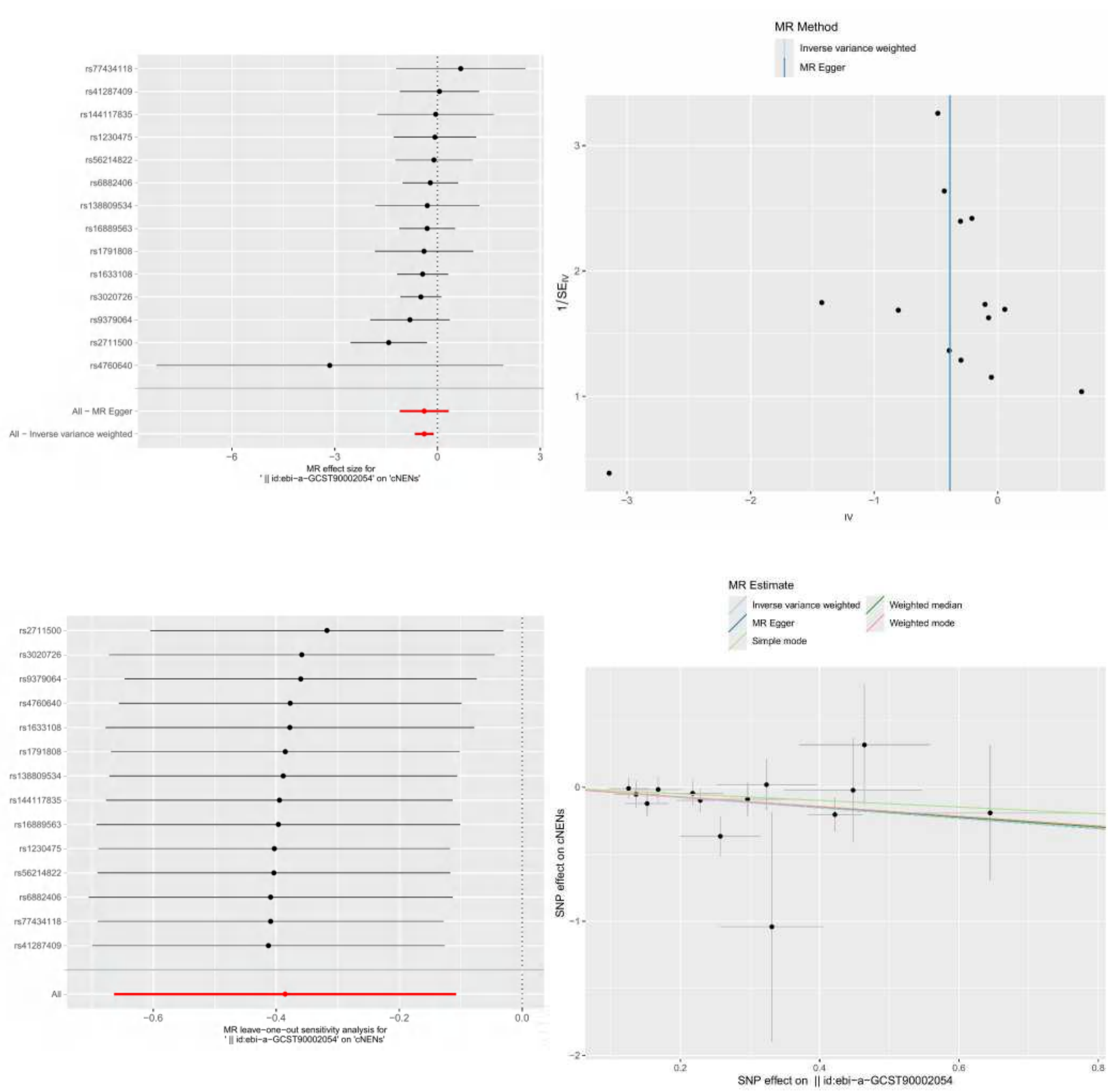

rs77434118
rs41287409
rs144117835
rs1230475
rs56214822
rs6882406
rs138809534
rs16889563
rs1791808
rs1633108
rs3020726
rs9379064
rs2711500
rs4760640
All − MR Egger
All − Inverse variance weighted
MR effect size for
' || id:ebi−a−GCST90002054' on 'cNENs'
MR Method
Inverse variance weighted
MR Egger
1/SE_IV
IV
rs2711500
rs3020726
rs9379064
rs4760640
rs1633108
rs1791808
rs138809534
rs144117835
rs16889563
rs1230475
rs56214822
rs6882406
rs77434118
rs41287409
All
MR leave−one−out sensitivity analysis for
' || id:ebi−a−GCST90002054' on 'cNENs'
MR Estimate
Inverse variance weighted
MR Egger
Simple mode
Weighted median
Weighted mode
SNP effect on cNENs
SNP effect on || id:ebi−a−GCST90002054

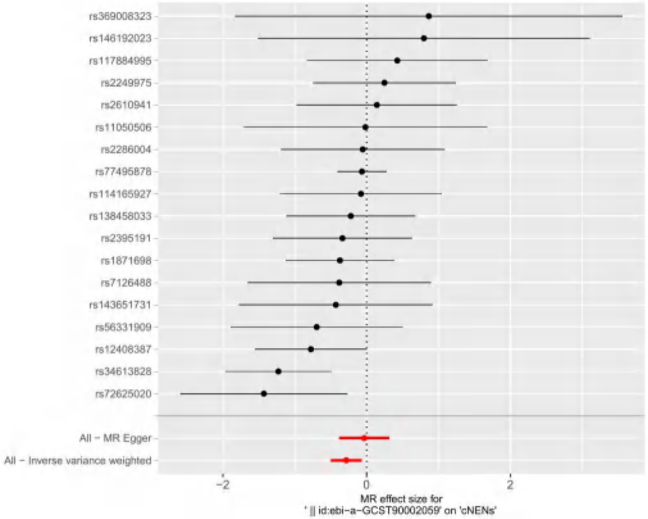
rs369008323
rs146192023
rs117884995
rs2249975
rs2610941
rs11050506
rs2286004
rs77495878
rs114165927
rs138458033
rs2395191
rs1871698
rs7126488
rs143651731
rs56331909
rs12408387
rs34613828
rs72625020
All − MR Egger
All − Inverse variance weighted
-2
0
2
MR effect size for
' || id:ebi-a-GCST90002059' on 'cNENs'

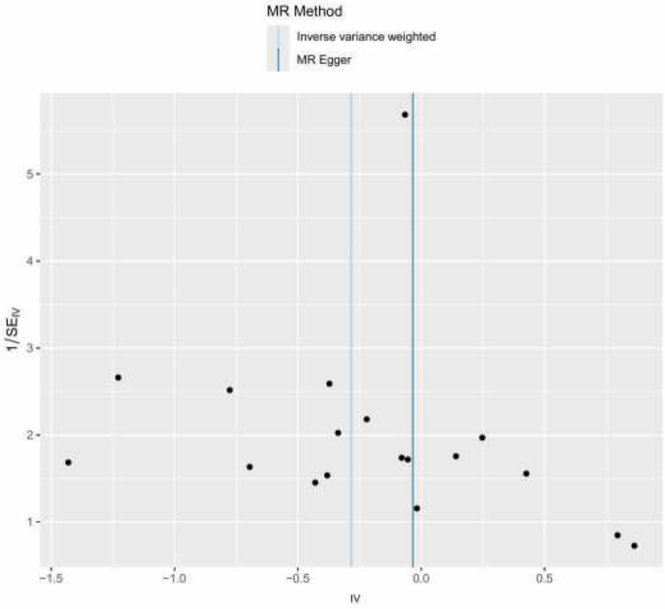
MR Method
Inverse variance weighted
MR Egger
1/SE_IV
5
4
3
2
1
-1.5
-1.0
-0.5
0.0
0.5
IV

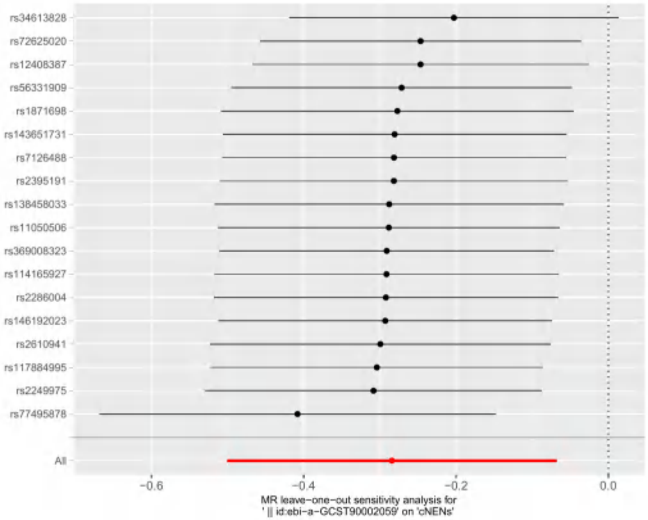
rs34613828
rs72625020
rs12408387
rs56331909
rs1871698
rs143651731
rs7126488
rs2395191
rs138458033
rs11050506
rs369008323
rs114165927
rs2286004
rs146192023
rs2610941
rs117884995
rs2249975
rs77495878
All
-0.6
-0.4
-0.2
0.0
MR leave−one−out sensitivity analysis for
' || id:ebi-a-GCST90002059' on 'cNENs'

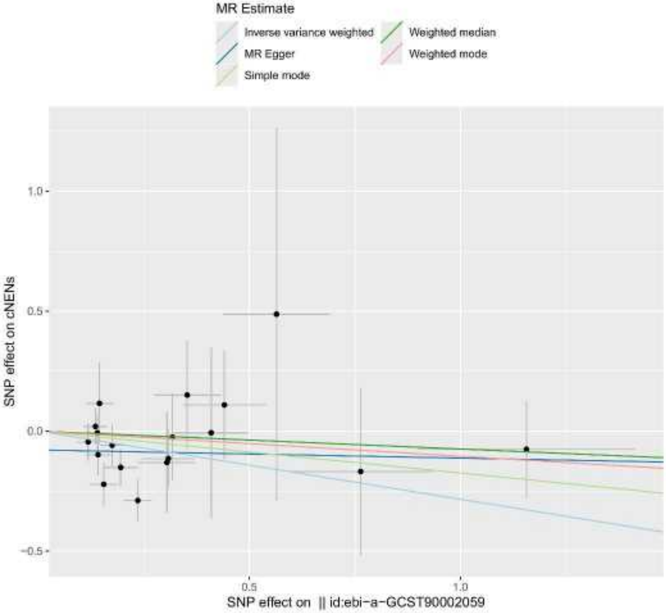
MR Estimate
Inverse variance weighted
MR Egger
Simple mode
Weighted median
Weighted mode
1.0
0.5
0.0
-0.5
SNP effect on cNENs
0.5
1.0
SNP effect on || id:ebi-a-GCST90002059

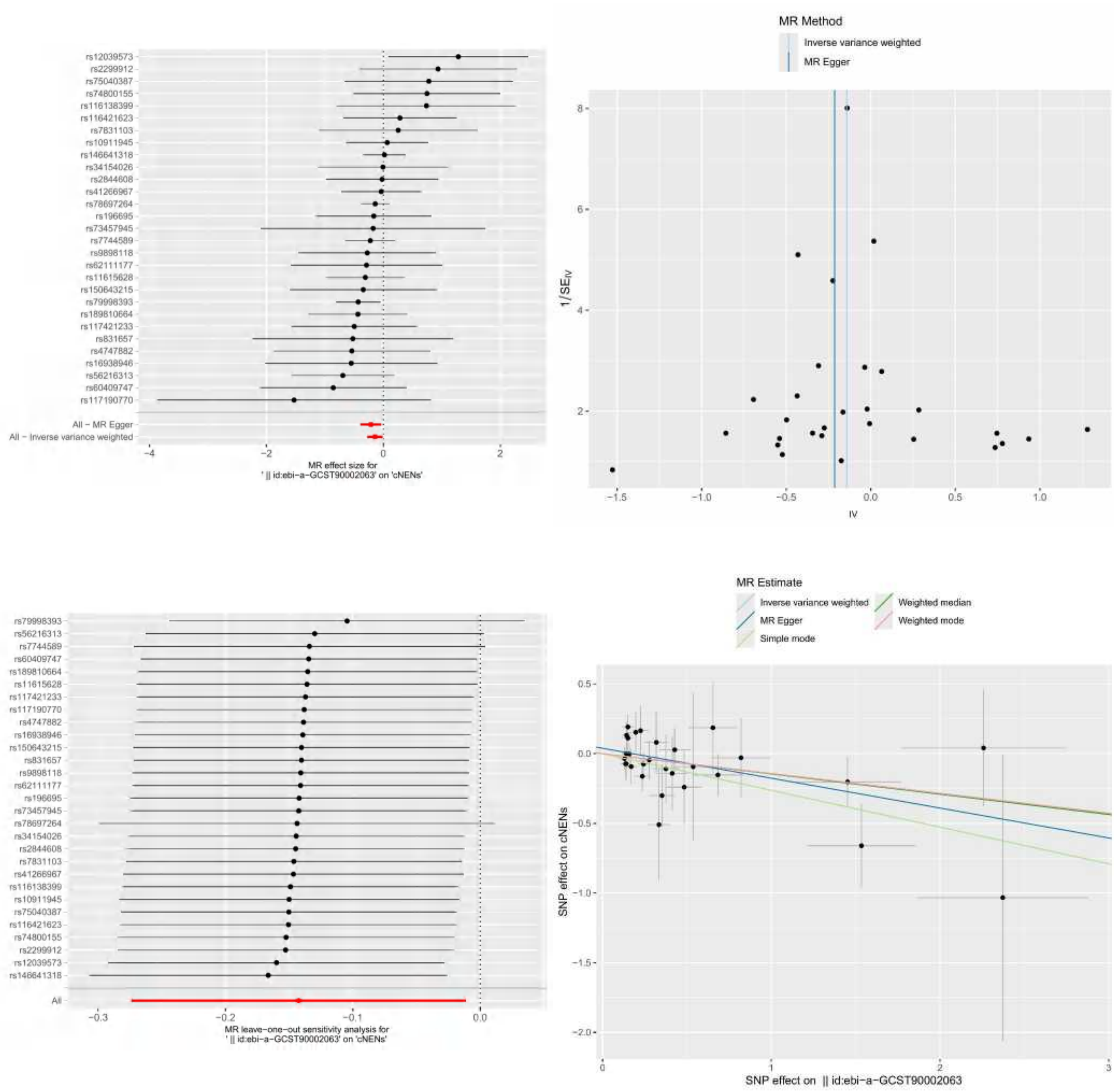

rs12039573
rs2299912
rs75040387
rs74800155
rs116138399
rs116421623
rs7831103
rs10911945
rs146641318
rs34154026
rs2844608
rs41266967
rs78697264
rs196695
rs73457945
rs7744589
rs9898118
rs62111177
rs11615628
rs150643215
rs79998393
rs189810664
rs117421233
rs831657
rs4747882
rs16938946
rs56216313
rs60409747
rs117190770
All − MR Egger
All − Inverse variance weighted
MR effect size for
' || id:ebi-a-GCST90002063' on 'cNENs'
MR Method
Inverse variance weighted
MR Egger
1/SE_IV
IV
rs79998393
rs56216313
rs7744589
rs60409747
rs189810664
rs11615628
rs117421233
rs117190770
rs4747882
rs16938946
rs150643215
rs831657
rs9898118
rs62111177
rs196695
rs73457945
rs78697264
rs34154026
rs2844608
rs7831103
rs41266967
rs116138399
rs10911945
rs75040387
rs116421623
rs74800155
rs2299912
rs12039573
rs146641318
All
MR leave−one−out sensitivity analysis for
' || id:ebi-a-GCST90002063' on 'cNENs'
MR Estimate
Inverse variance weighted
MR Egger
Simple mode
Weighted median
Weighted mode
SNP effect on cNENs
SNP effect on || id:ebi-a-GCST90002063

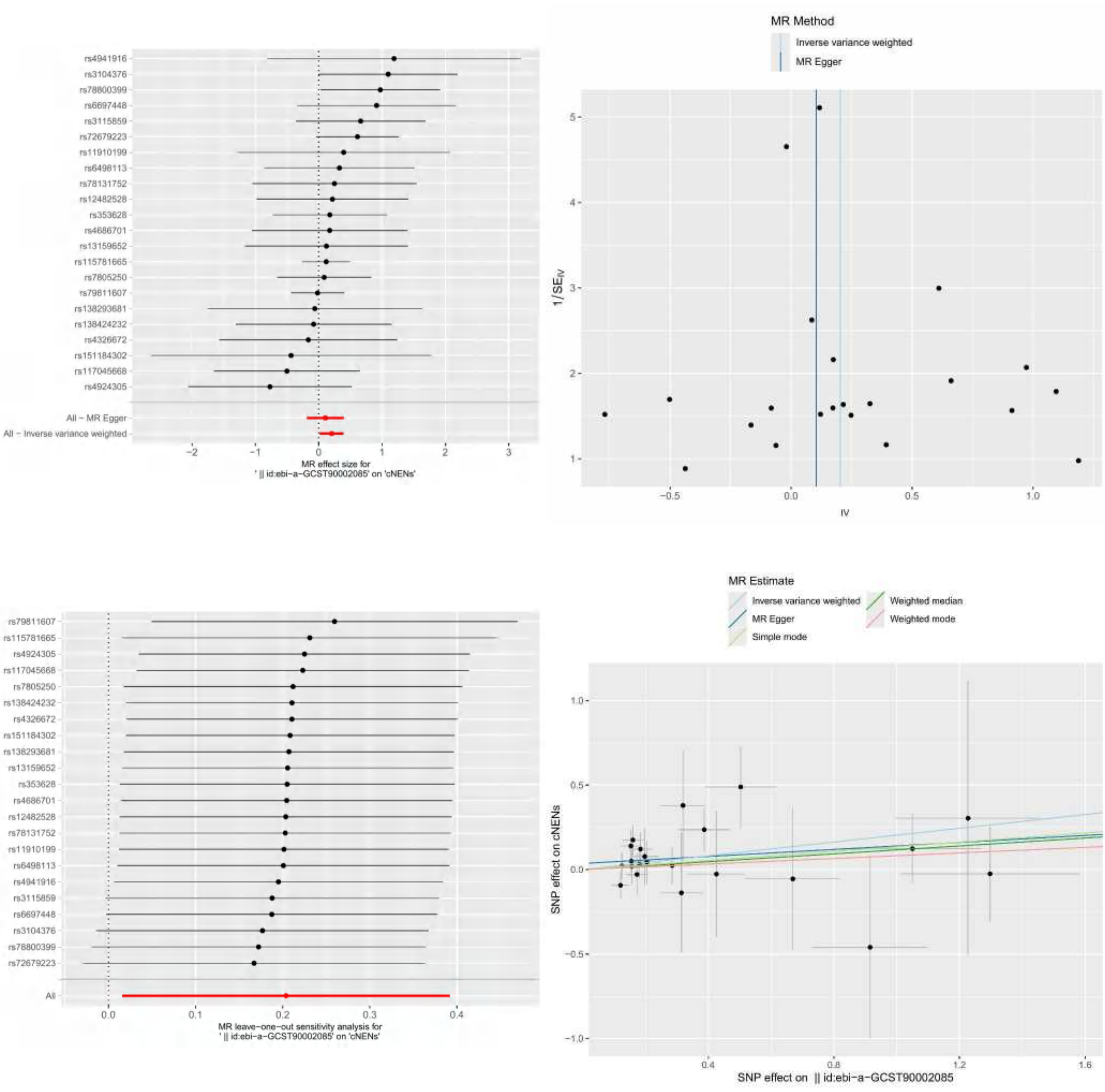
rs4941916
rs3104376
rs78800399
rs6697448
rs3115859
rs72679223
rs11910199
rs6498113
rs78131752
rs12482528
rs353628
rs4686701
rs13159652
rs115781665
rs7805250
rs79811607
rs138293681
rs138424232
rs4326672
rs151184302
rs117045668
rs4924305
All − MR Egger
All − Inverse variance weighted
−2
−1
0
1
2
3
MR effect size for
' || id:ebi−a−GCST90002085' on 'cNENs'
MR Method
Inverse variance weighted
MR Egger
1/SE_IV
IV
−0.5
0.0
0.5
1.0
rs79811607
rs115781665
rs4924305
rs117045668
rs7805250
rs138424232
rs4326672
rs151184302
rs138293681
rs13159652
rs353628
rs4686701
rs12482528
rs78131752
rs11910199
rs6498113
rs4941916
rs3115859
rs6697448
rs3104376
rs78800399
rs72679223
All
0.0
0.1
0.2
0.3
0.4
MR leave−one−out sensitivity analysis for
' || id:ebi−a−GCST90002085' on 'cNENs'
MR Estimate
Inverse variance weighted
MR Egger
Simple mode
Weighted median
Weighted mode
SNP effect on cNENs
SNP effect on || id:ebi−a−GCST90002085
1.0
0.5
0.0
−0.5
−1.0
0.4
0.8
1.2
1.6

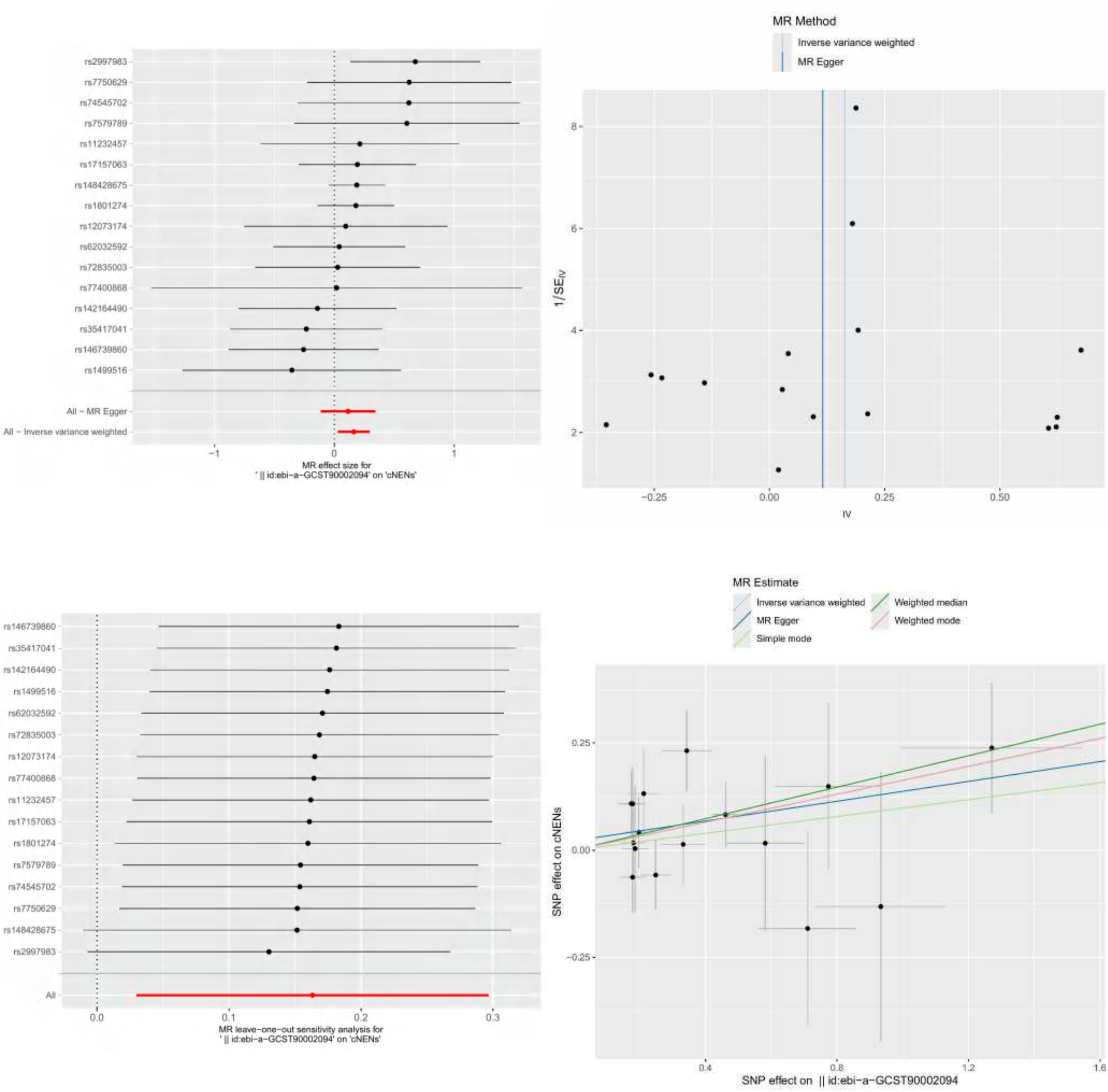

rs2997983
rs7750629
rs74545702
rs7579789
rs11232457
rs17157063
rs148428675
rs1801274
rs12073174
rs62032592
rs72835003
rs77400868
rs142164490
rs35417041
rs146739860
rs1499516
All − MR Egger
All − Inverse variance weighted
−1
0
1
MR effect size for
' || id:ebi−a−GCST90002094' on 'cNENs'
MR Method
Inverse variance weighted
MR Egger
1/SE_IV
8
6
4
2
−0.25
0.00
0.25
0.50
IV
rs146739860
rs35417041
rs142164490
rs1499516
rs62032592
rs72835003
rs12073174
rs77400868
rs11232457
rs17157063
rs1801274
rs7579789
rs74545702
rs7750629
rs148428675
rs2997983
All
0.0
0.1
0.2
0.3
MR leave−one−out sensitivity analysis for
' || id:ebi−a−GCST90002094' on 'cNENs'
MR Estimate
Inverse variance weighted
MR Egger
Simple mode
Weighted median
Weighted mode
0.25
0.00
−0.25
SNP effect on cNENs
0.4
0.8
1.2
1.6
SNP effect on || id:ebi−a−GCST90002094

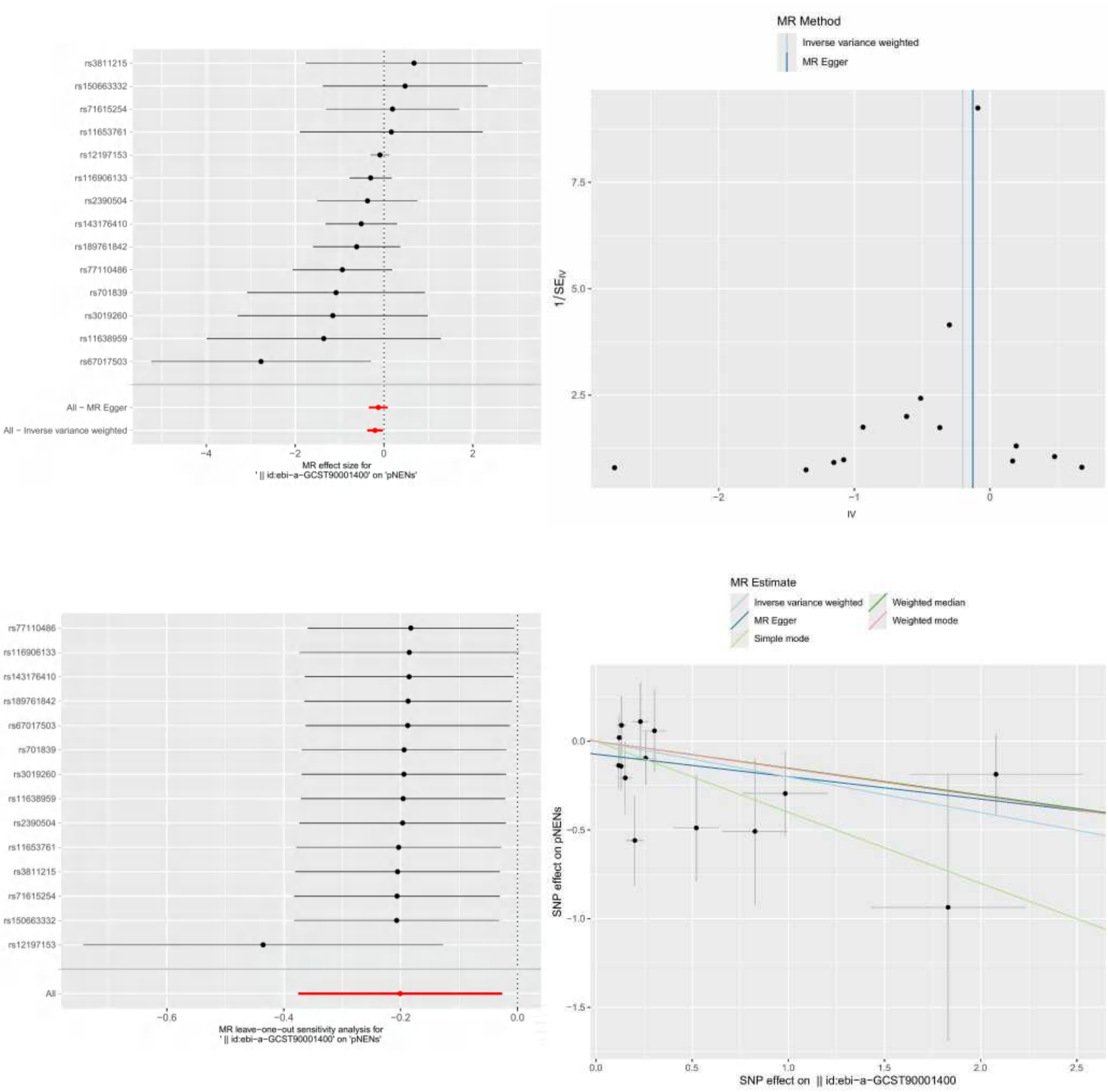
rs3811215
rs150663332
rs71615254
rs11653761
rs12197153
rs116906133
rs2390504
rs143176410
rs189761842
rs77110486
rs701839
rs3019260
rs11638959
rs67017503
All − MR Egger
All − Inverse variance weighted
−4
−2
0
2
MR effect size for
' || id:ebi−a−GCST90001400' on 'pNENs'
MR Method
Inverse variance weighted
MR Egger
1/SE_IV
7.5
5.0
2.5
−2
−1
0
IV
rs77110486
rs116906133
rs143176410
rs189761842
rs67017503
rs701839
rs3019260
rs11638959
rs2390504
rs11653761
rs3811215
rs71615254
rs150663332
rs12197153
All
−0.6
−0.4
−0.2
0.0
MR leave−one−out sensitivity analysis for
' || id:ebi−a−GCST90001400' on 'pNENs'
MR Estimate
Inverse variance weighted
MR Egger
Simple mode
Weighted median
Weighted mode
SNP effect on pNENs
0.0
−0.5
−1.0
−1.5
0.0
0.5
1.0
1.5
2.0
2.5
SNP effect on || id:ebi−a−GCST90001400

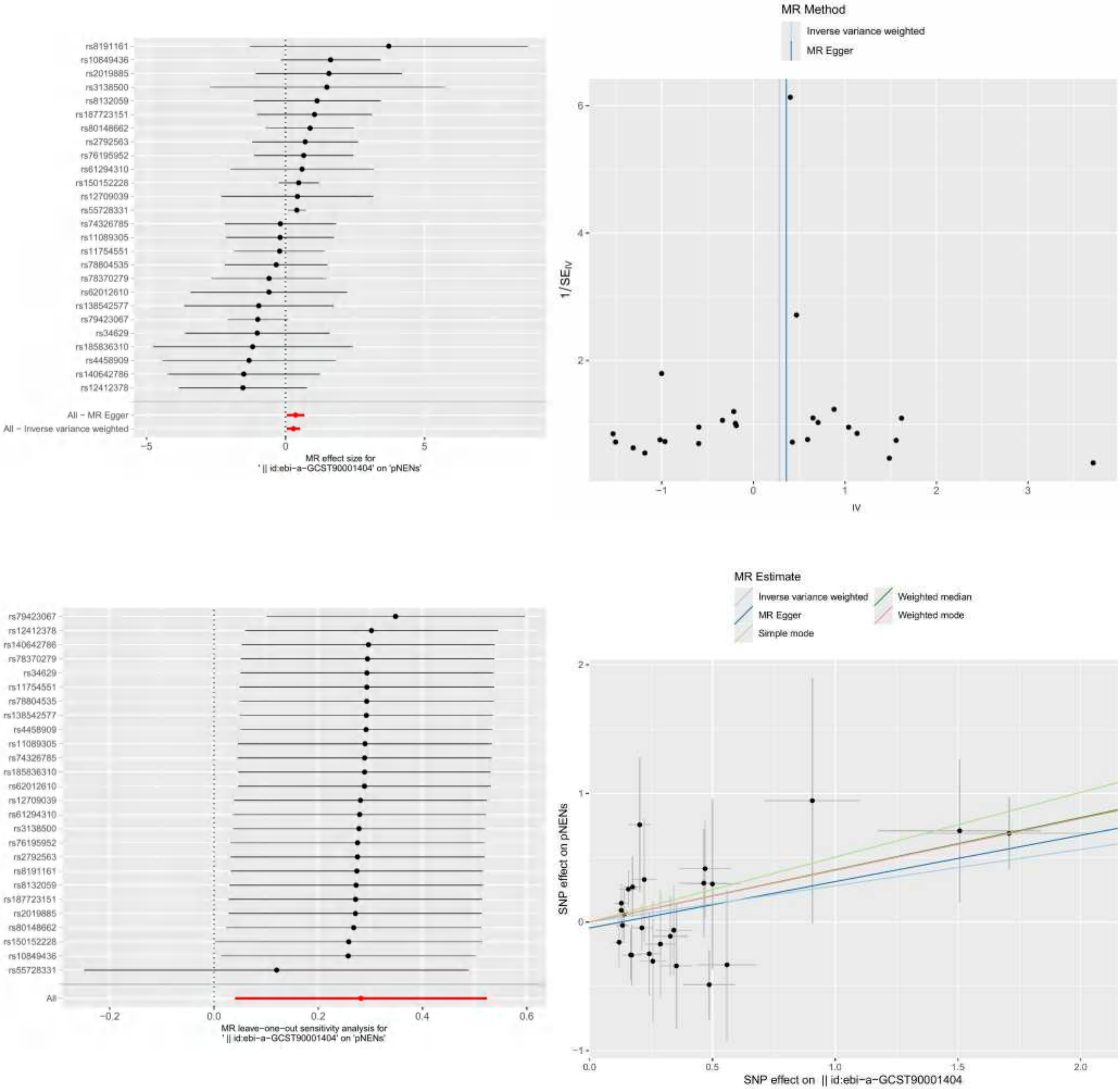

rs8191161
rs10849436
rs2019885
rs3138500
rs8132059
rs187723151
rs80148662
rs2792563
rs76195952
rs61294310
rs150152228
rs12709039
rs55728331
rs74326785
rs11089305
rs11754551
rs78804535
rs78370279
rs62012610
rs138542577
rs79423067
rs34629
rs185836310
rs4458909
rs140642786
rs12412378
All − MR Egger
All − Inverse variance weighted
−5
0
5
MR effect size for
' || id:ebi−a−GCST90001404' on 'pNENs'
MR Method
Inverse variance weighted
MR Egger
1/SE_IV
6
4
2
−1
0
1
2
3
IV
rs79423067
rs12412378
rs140642786
rs78370279
rs34629
rs11754551
rs78804535
rs138542577
rs4458909
rs11089305
rs74326785
rs185836310
rs62012610
rs12709039
rs61294310
rs3138500
rs76195952
rs2792563
rs8191161
rs8132059
rs187723151
rs2019885
rs80148662
rs150152228
rs10849436
rs55728331
All
−0.2
0.0
0.2
0.4
0.6
MR leave−one−out sensitivity analysis for
' || id:ebi−a−GCST90001404' on 'pNENs'
MR Estimate
Inverse variance weighted
MR Egger
Simple mode
Weighted median
Weighted mode
SNP effect on pNENs
2
1
0
−1
0.0
0.5
1.0
1.5
2.0
SNP effect on || id:ebi−a−GCST90001404

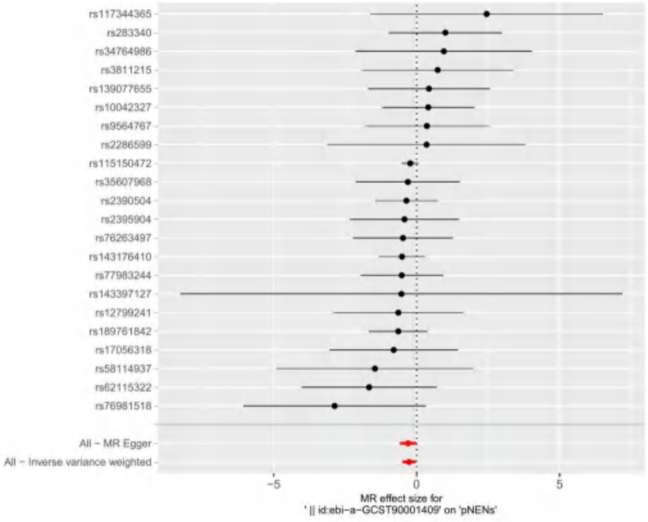
rs117344365
rs283340
rs34764986
rs3811215
rs139077655
rs10042327
rs9564767
rs2286599
rs115150472
rs35607968
rs2390504
rs2395904
rs76263497
rs143176410
rs77983244
rs143397127
rs12799241
rs189761842
rs17056318
rs58114937
rs62115322
rs76981518
All − MR Egger
All − Inverse variance weighted
−5
0
5
MR effect size for
' || id:ebi−a−GCST90001409' on 'pNENs'

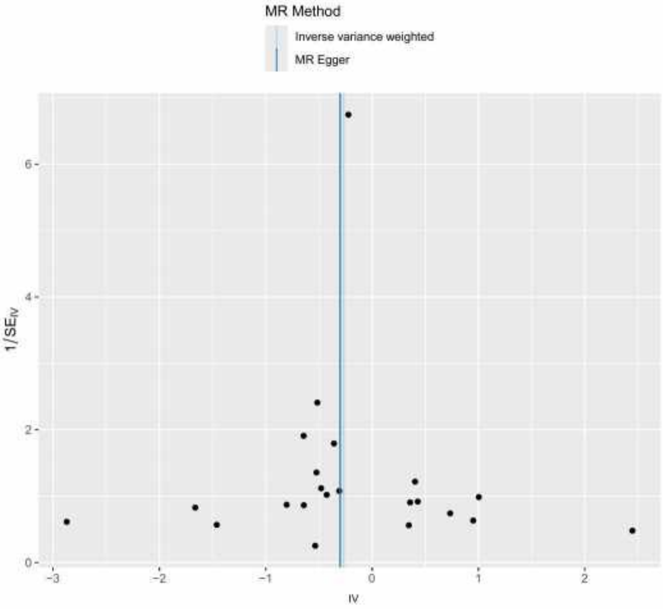
MR Method
Inverse variance weighted
MR Egger
$1/SE_{IV}$
0
2
4
6
−3
−2
−1
0
1
2
IV

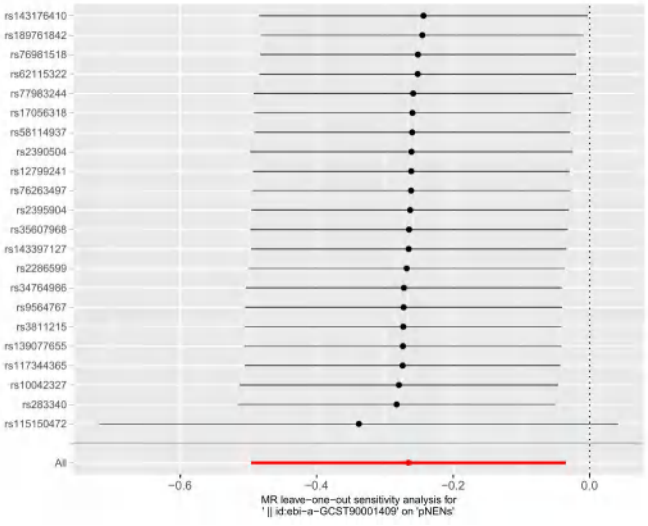
rs143176410
rs189761842
rs76981518
rs62115322
rs77983244
rs17056318
rs58114937
rs2390504
rs12799241
rs76263497
rs2395904
rs35607968
rs143397127
rs2286599
rs34764986
rs9564767
rs3811215
rs139077655
rs117344365
rs10042327
rs283340
rs115150472
All
−0.6
−0.4
−0.2
0.0
MR leave−one−out sensitivity analysis for
' || id:ebi−a−GCST90001409' on 'pNENs'

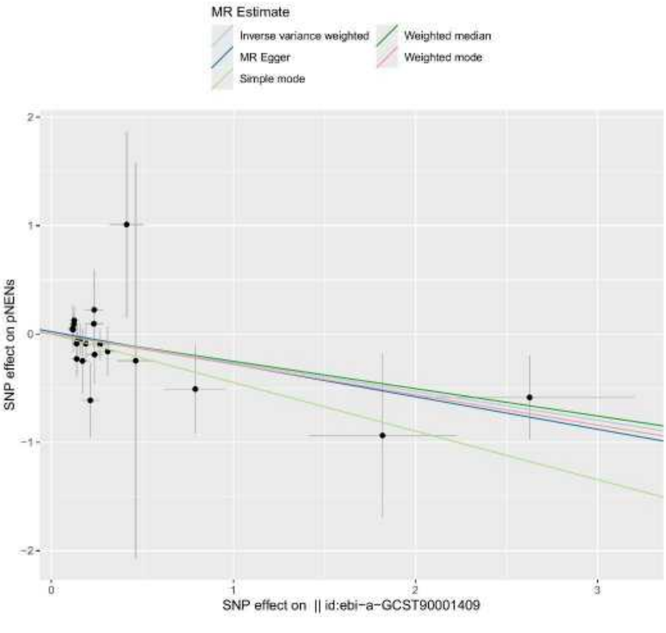
MR Estimate
Inverse variance weighted
MR Egger
Simple mode
Weighted median
Weighted mode
SNP effect on pNENs
2
1
0
−1
−2
0
1
2
3
SNP effect on || id:ebi−a−GCST90001409

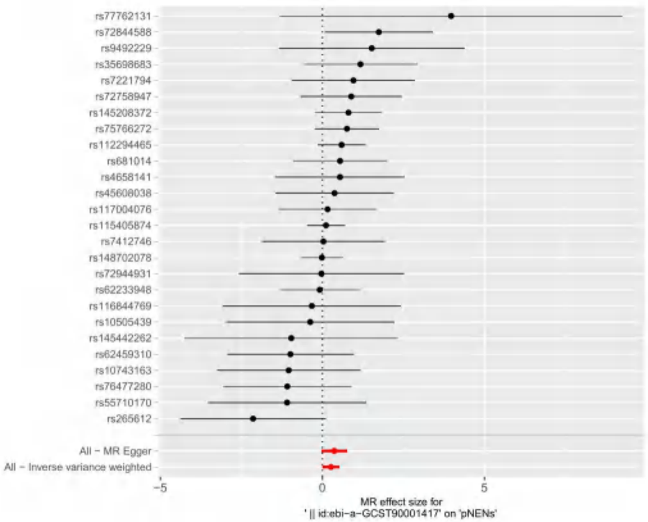
rs77762131
rs72844588
rs9492229
rs35698683
rs7221794
rs72758947
rs145208372
rs75766272
rs112294465
rs681014
rs4658141
rs45608038
rs117004076
rs115405874
rs7412746
rs148702078
rs72944931
rs62233948
rs116844769
rs10505439
rs145442262
rs62459310
rs10743163
rs76477280
rs55710170
rs265612
All − MR Egger
All − Inverse variance weighted
−5
0
5
MR effect size for
' || id:ebi−a−GCST90001417' on 'pNENs'

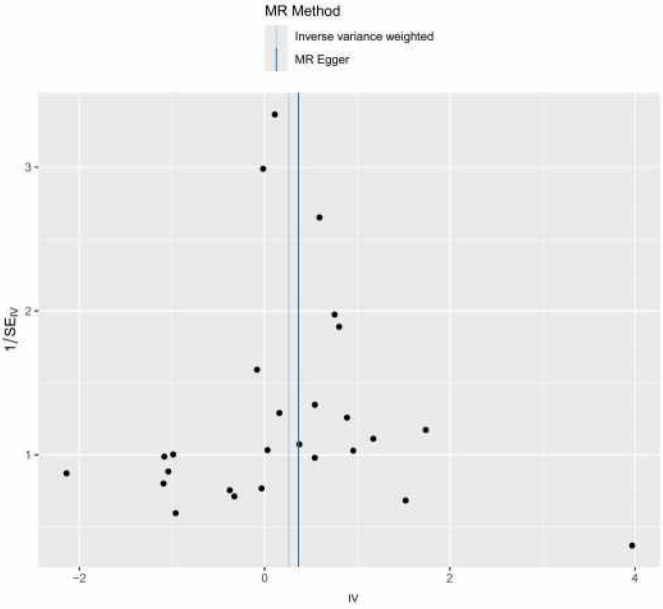
MR Method
Inverse variance weighted
MR Egger
$1/SE_{IV}$
3
2
1
−2
0
2
4
IV

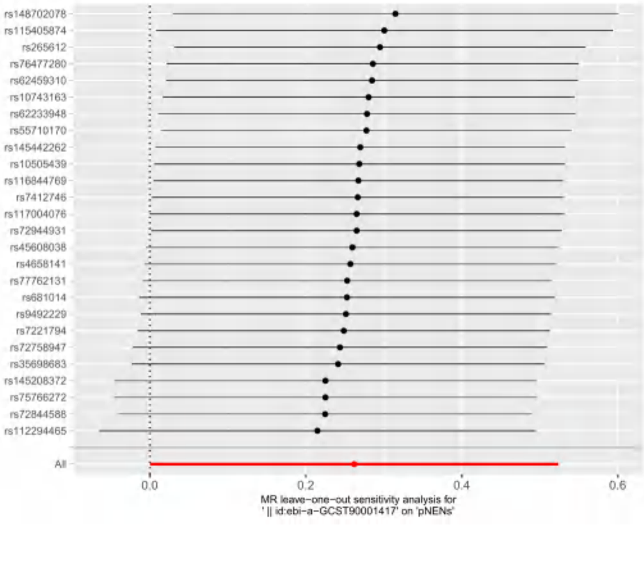
rs148702078
rs115405874
rs265612
rs76477280
rs62459310
rs10743163
rs62233948
rs55710170
rs145442262
rs10505439
rs116844769
rs7412746
rs117004076
rs72944931
rs45608038
rs4658141
rs77762131
rs681014
rs9492229
rs7221794
rs72758947
rs35698683
rs145208372
rs75766272
rs72844588
rs112294465
All
0.0
0.2
0.4
0.6
MR leave−one−out sensitivity analysis for
' || id:ebi−a−GCST90001417' on 'pNENs'

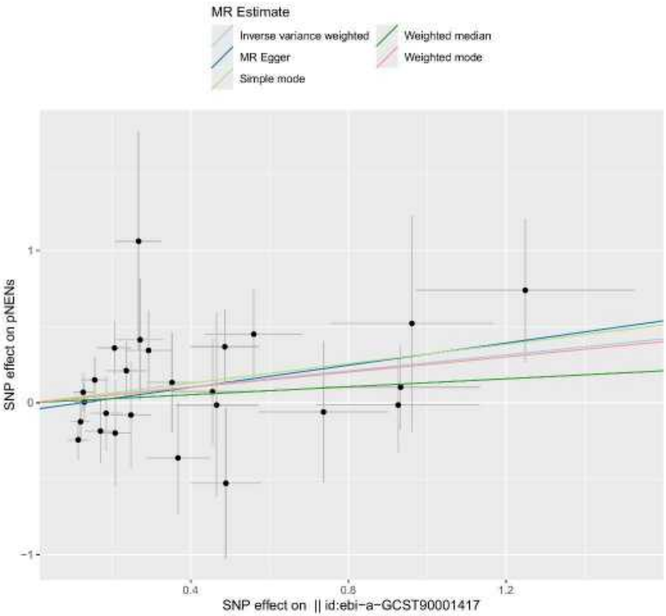
MR Estimate
Inverse variance weighted
MR Egger
Simple mode
Weighted median
Weighted mode
SNP effect on pNENs
1
0
−1
0.4
0.8
1.2
SNP effect on || id:ebi−a−GCST90001417

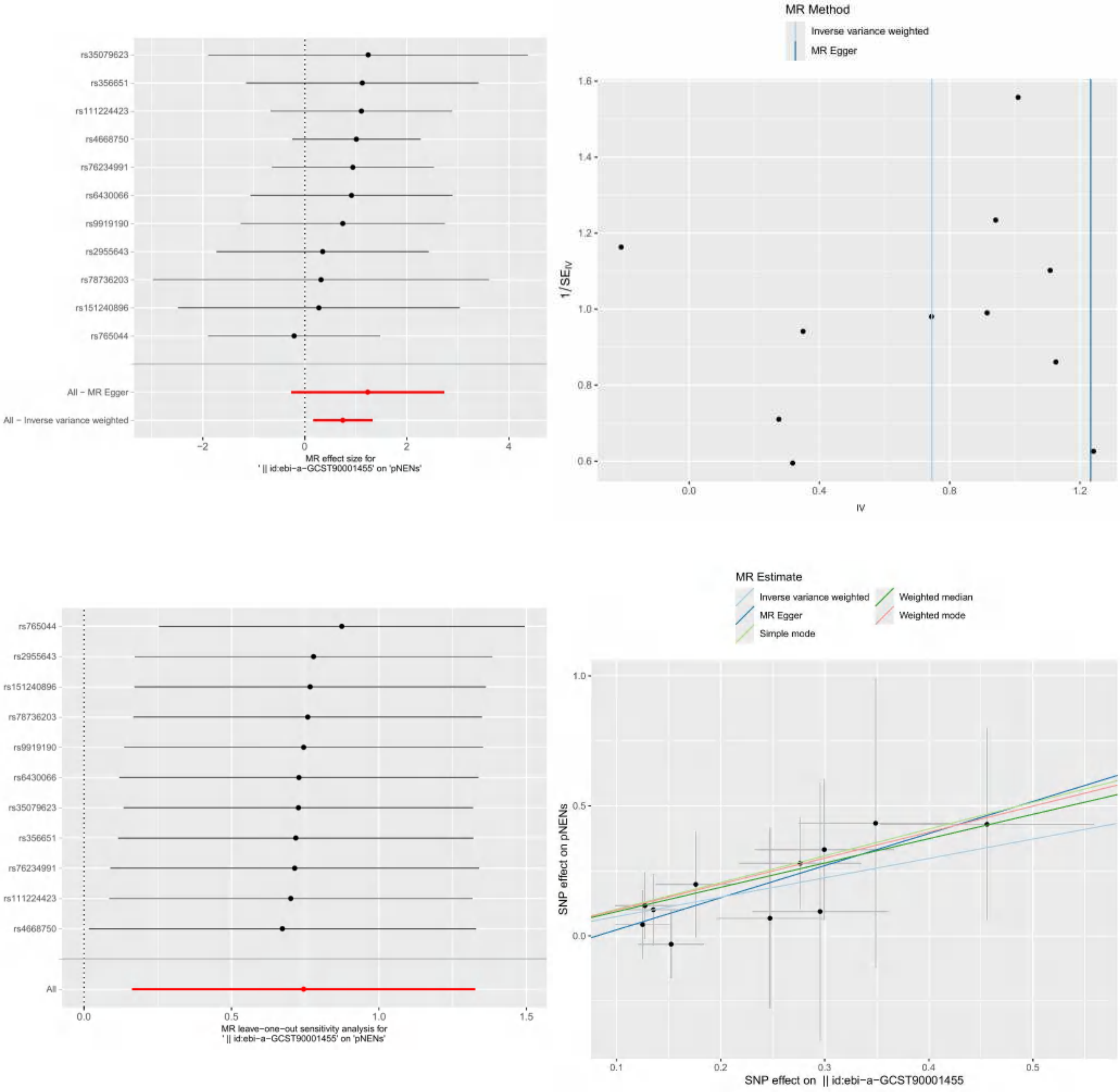
rs35079623
rs356651
rs111224423
rs4668750
rs76234991
rs6430066
rs9919190
rs2955643
rs78736203
rs151240896
rs765044
All − MR Egger
All − Inverse variance weighted
−2
0
2
4
MR effect size for
' || id:ebi−a−GCST90001455' on 'pNENs'
MR Method
Inverse variance weighted
MR Egger
1.6
1.4
1.2
1.0
0.8
0.6
$1/SE_{IV}$
0.0
0.4
0.8
1.2
IV
rs765044
rs2955643
rs151240896
rs78736203
rs9919190
rs6430066
rs35079623
rs356651
rs76234991
rs111224423
rs4668750
All
0.0
0.5
1.0
1.5
MR leave−one−out sensitivity analysis for
' || id:ebi−a−GCST90001455' on 'pNENs'
MR Estimate
Inverse variance weighted
MR Egger
Simple mode
Weighted median
Weighted mode
1.0
0.5
0.0
SNP effect on pNENs
0.1
0.2
0.3
0.4
0.5
SNP effect on || id:ebi−a−GCST90001455

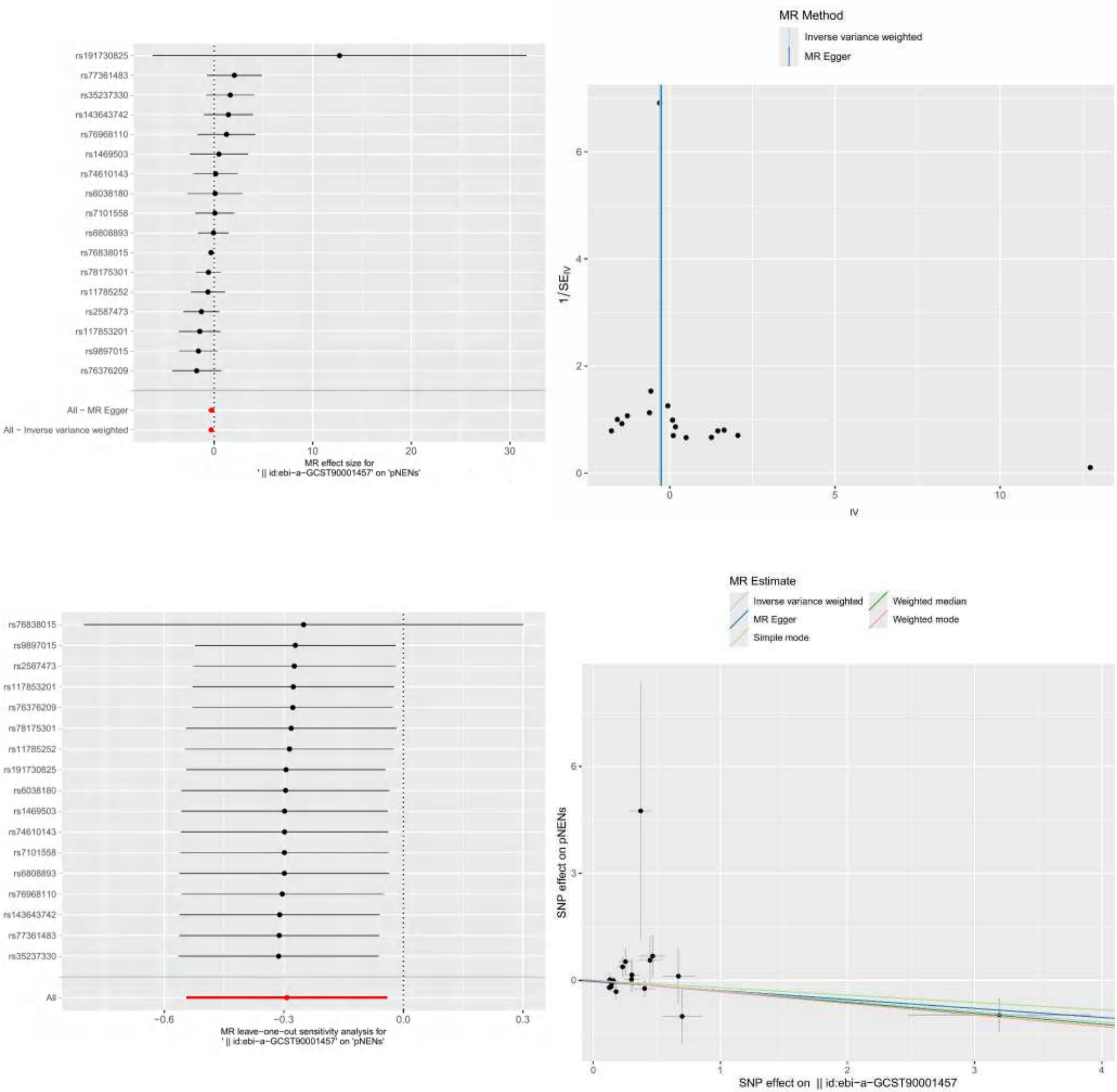

rs191730825
rs77361483
rs35237330
rs143643742
rs76968110
rs1469503
rs74610143
rs6038180
rs7101558
rs6808893
rs76838015
rs78175301
rs11785252
rs2587473
rs117853201
rs9897015
rs76376209
All − MR Egger
All − Inverse variance weighted
0
10
20
30
MR effect size for
' || id:ebi−a−GCST90001457' on 'pNENs'
MR Method
Inverse variance weighted
MR Egger
$1/SE_{IV}$
0
2
4
6
IV
0
5
10
rs76838015
rs9897015
rs2587473
rs117853201
rs76376209
rs78175301
rs11785252
rs191730825
rs6038180
rs1469503
rs74610143
rs7101558
rs6808893
rs76968110
rs143643742
rs77361483
rs35237330
All
−0.6
−0.3
0.0
0.3
MR leave−one−out sensitivity analysis for
' || id:ebi−a−GCST90001457' on 'pNENs'
MR Estimate
Inverse variance weighted
MR Egger
Simple mode
Weighted median
Weighted mode
SNP effect on pNENs
0
3
6
SNP effect on || id:ebi−a−GCST90001457
0
1
2
3
4

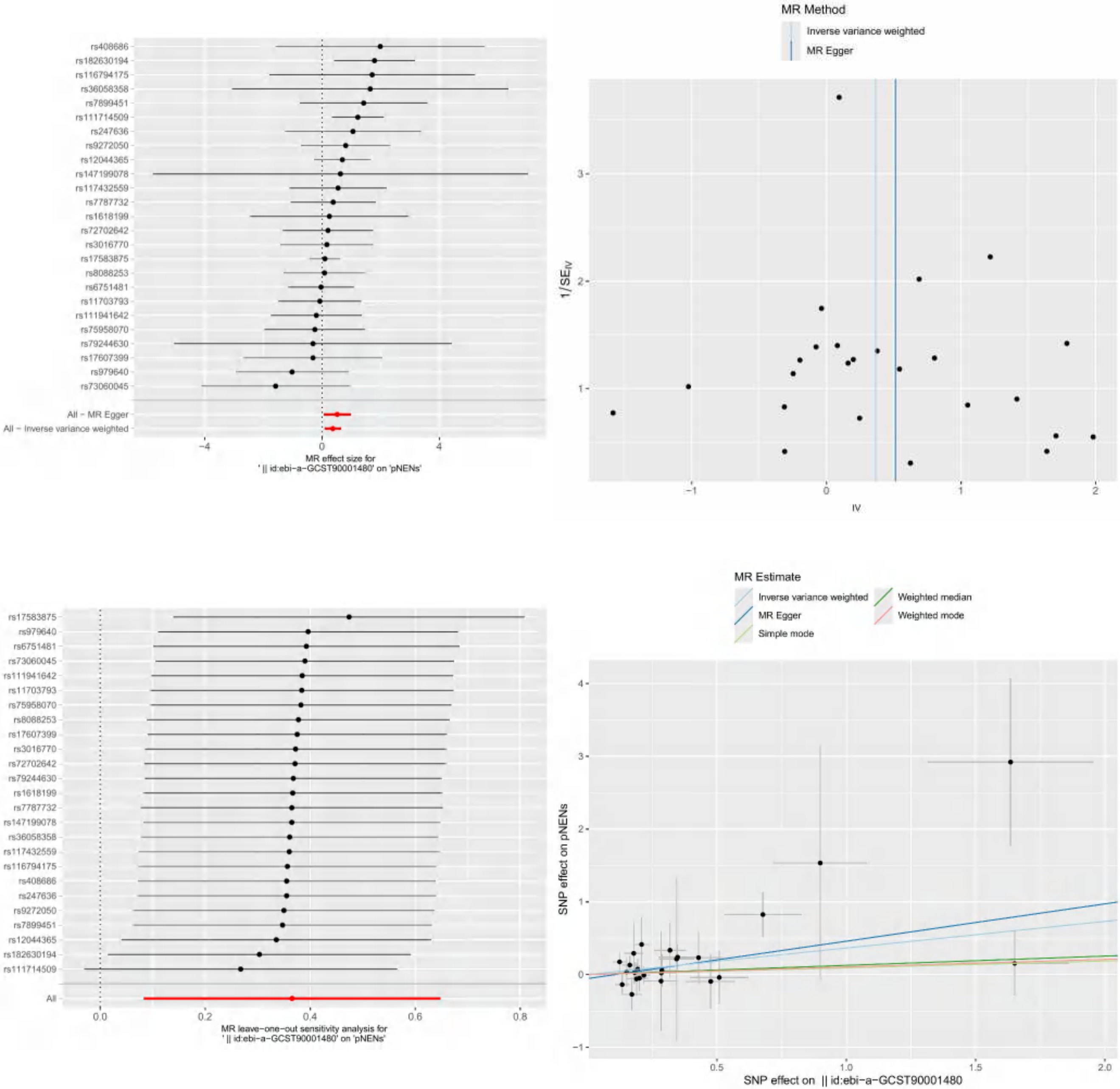

rs408686
rs182630194
rs116794175
rs36058358
rs7899451
rs111714509
rs247636
rs9272050
rs12044365
rs147199078
rs117432559
rs7787732
rs1618199
rs72702642
rs3016770
rs17583875
rs8088253
rs6751481
rs11703793
rs111941642
rs75958070
rs79244630
rs17607399
rs979640
rs73060045
All - MR Egger
All - Inverse variance weighted
MR effect size for
' || id:ebi-a-GCST90001480' on 'pNENs'
MR Method
Inverse variance weighted
MR Egger
1/SE_IV
IV
All
MR leave-one-out sensitivity analysis for
' || id:ebi-a-GCST90001480' on 'pNENs'
MR Estimate
Inverse variance weighted
MR Egger
Simple mode
Weighted median
Weighted mode
SNP effect on pNENs
SNP effect on || id:ebi-a-GCST90001480

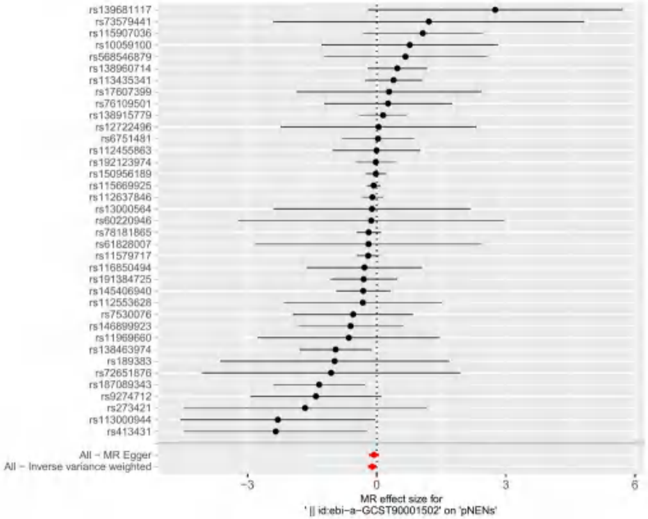
rs139681117
rs73579441
rs115907036
rs10059100
rs568546879
rs138960714
rs113435341
rs17607399
rs76109501
rs138915779
rs12722496
rs6751481
rs112455863
rs192123974
rs150956189
rs115669925
rs112637846
rs13000564
rs60220946
rs78181865
rs61828007
rs11579717
rs116850494
rs191384725
rs145406940
rs112553628
rs7530076
rs146899923
rs11969660
rs138463974
rs189383
rs72651876
rs187089343
rs9274712
rs273421
rs113000944
rs413431
All - MR Egger
All - Inverse variance weighted
-3
0
3
6
MR effect size for
' || id:ebi-a-GCST90001502' on 'pNENs'

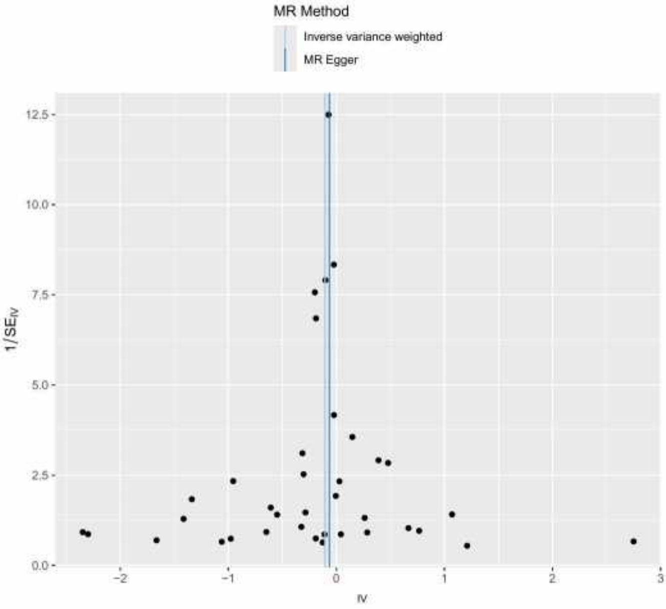
MR Method
Inverse variance weighted
MR Egger
12.5
10.0
7.5
5.0
2.5
0.0
1/SE$_{IV}$
-2
-1
0
1
2
3
IV

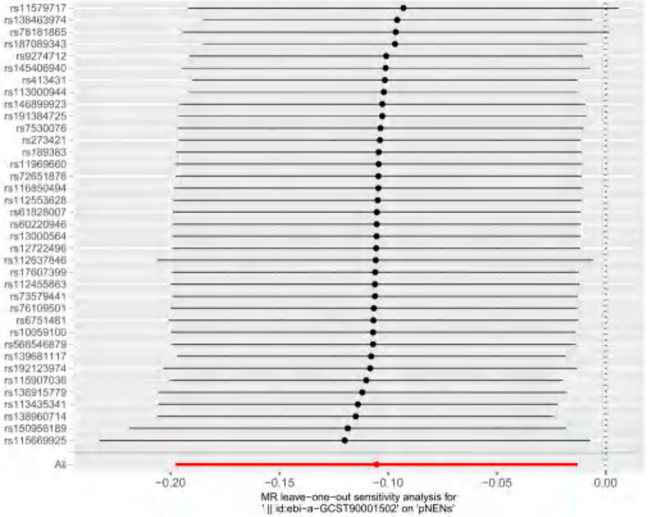
rs11579717
rs138463974
rs78181865
rs187089343
rs9274712
rs145406940
rs413431
rs113000944
rs146899923
rs191384725
rs7530076
rs273421
rs189383
rs11969660
rs72651876
rs116850494
rs112553628
rs61828007
rs60220946
rs13000564
rs12722496
rs112637846
rs17607399
rs112455863
rs73579441
rs76109501
rs6751481
rs10059100
rs568546879
rs139681117
rs192123974
rs115907036
rs138915779
rs113435341
rs138960714
rs150956189
rs115669925
All
-0.20
-0.15
-0.10
-0.05
0.00
MR leave-one-out sensitivity analysis for
' || id:ebi-a-GCST90001502' on 'pNENs'

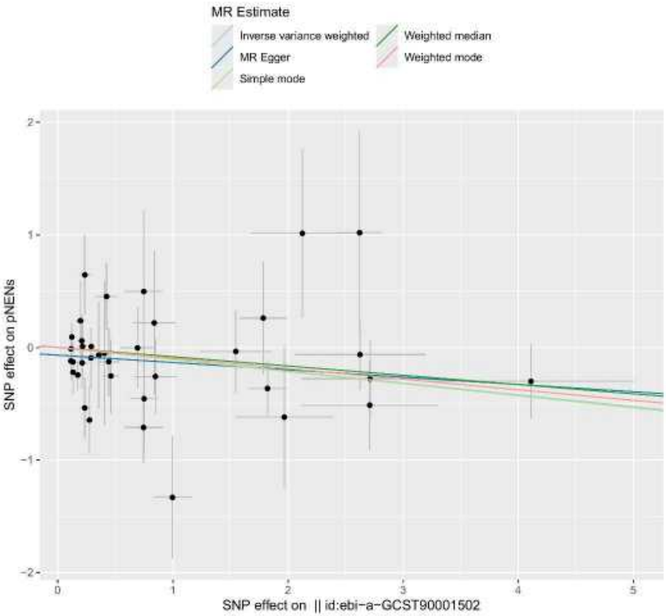
MR Estimate
Inverse variance weighted
MR Egger
Simple mode
Weighted median
Weighted mode
2
1
0
-1
-2
SNP effect on pNENs
0
1
2
3
4
5
SNP effect on || id:ebi-a-GCST90001502

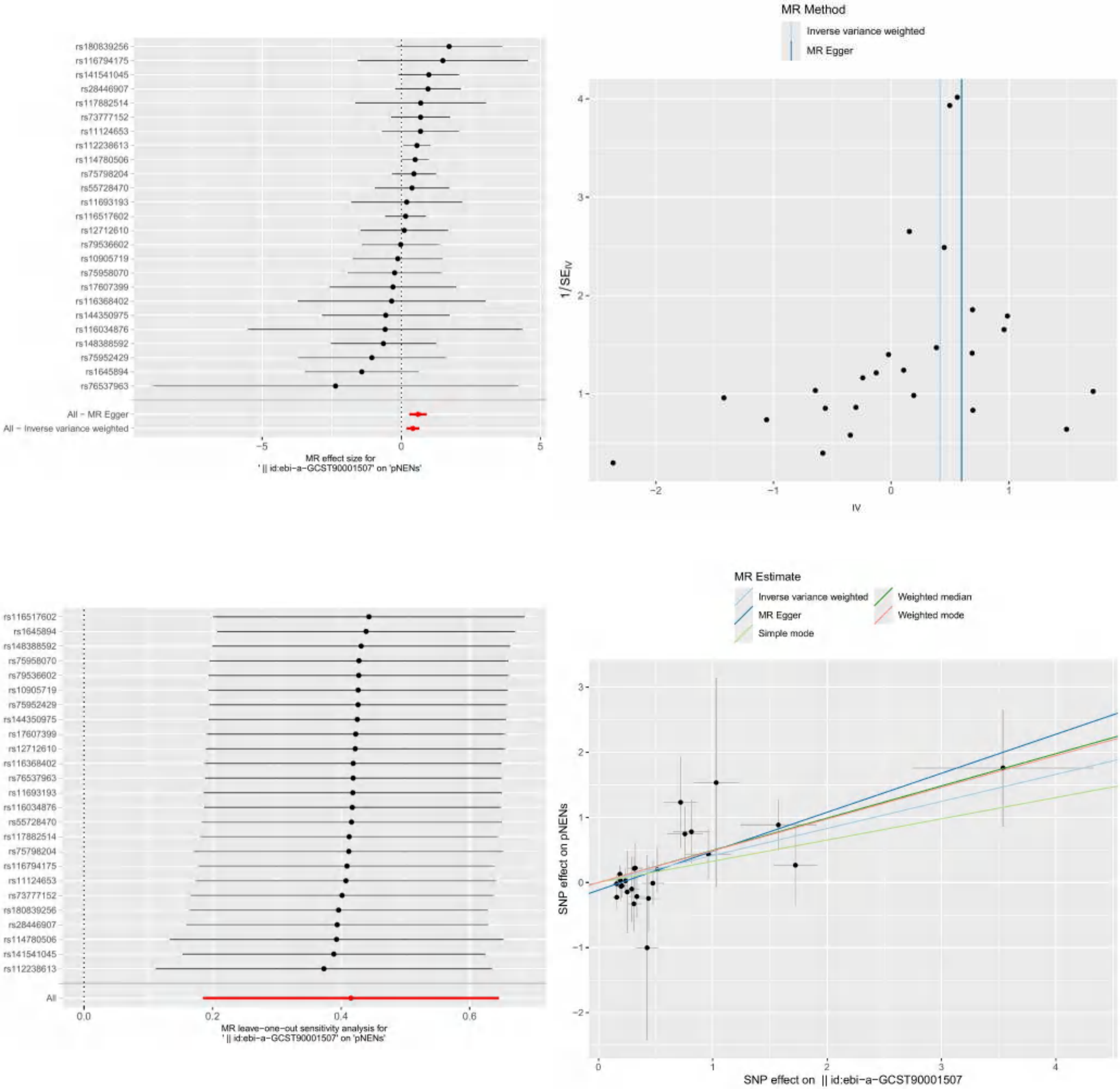

rs180839256
rs116794175
rs141541045
rs28446907
rs117882514
rs73777152
rs11124653
rs112238613
rs114780506
rs75798204
rs55728470
rs11693193
rs116517602
rs12712610
rs79536602
rs10905719
rs75958070
rs17607399
rs116368402
rs144350975
rs116034876
rs148388592
rs75952429
rs1645894
rs76537963
All − MR Egger
All − Inverse variance weighted
−5
0
5
MR effect size for
' || id:ebi−a−GCST90001507' on 'pNENs'
MR Method
Inverse variance weighted
MR Egger
1/SE$_{IV}$
IV
rs116517602
rs1645894
rs148388592
rs75958070
rs79536602
rs10905719
rs75952429
rs144350975
rs17607399
rs12712610
rs116368402
rs76537963
rs11693193
rs116034876
rs55728470
rs117882514
rs75798204
rs116794175
rs11124653
rs73777152
rs180839256
rs28446907
rs114780506
rs141541045
rs112238613
All
0.0
0.2
0.4
0.6
MR leave−one−out sensitivity analysis for
' || id:ebi−a−GCST90001507' on 'pNENs'
MR Estimate
Inverse variance weighted
MR Egger
Simple mode
Weighted median
Weighted mode
SNP effect on pNENs
SNP effect on || id:ebi−a−GCST90001507

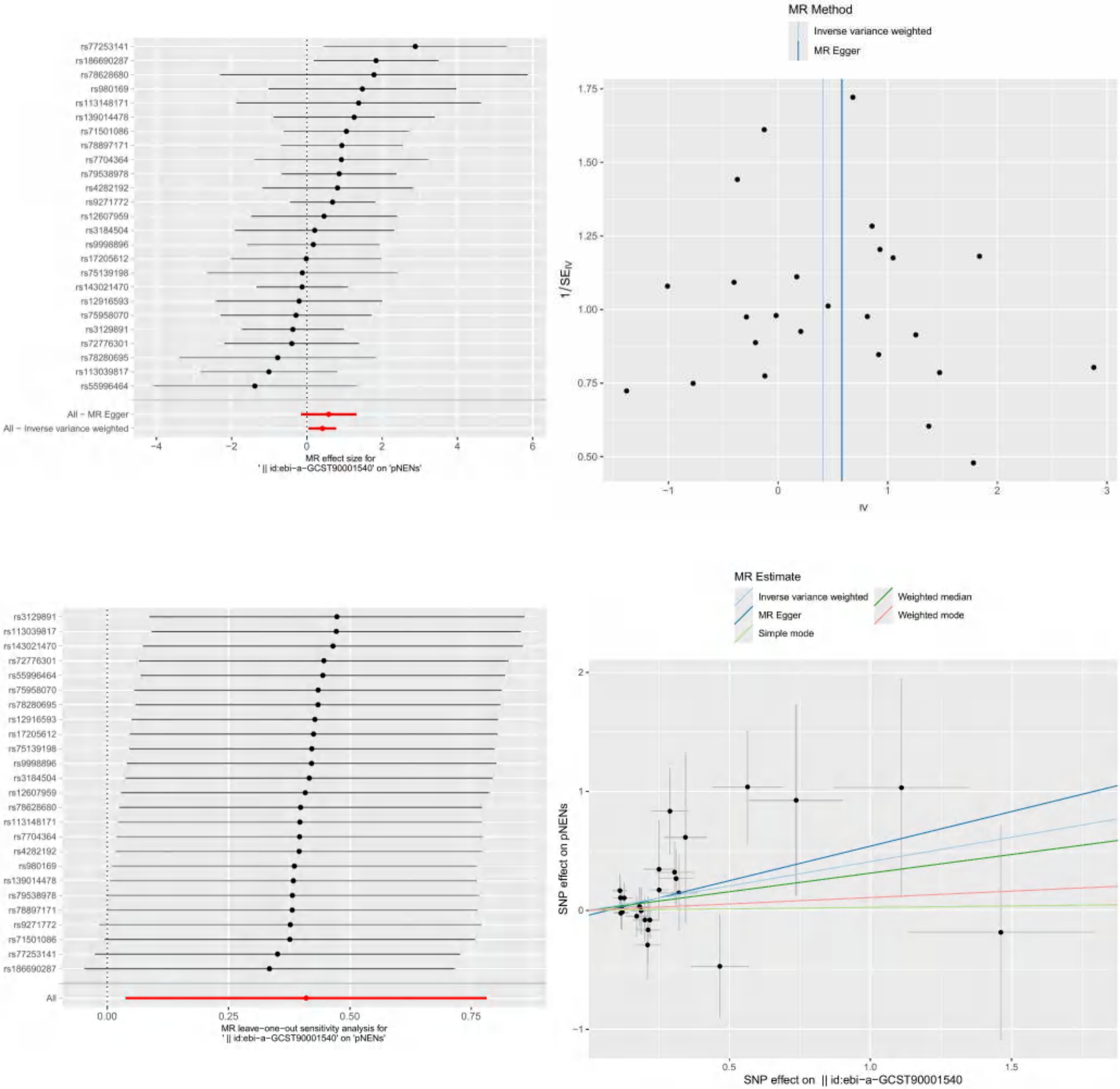

rs77253141
rs186690287
rs78628680
rs980169
rs113148171
rs139014478
rs71501086
rs78897171
rs7704364
rs79538978
rs4282192
rs9271772
rs12607959
rs3184504
rs9998896
rs17205612
rs75139198
rs143021470
rs12916593
rs75958070
rs3129891
rs72776301
rs78280695
rs113039817
rs55996464
All − MR Egger
All − Inverse variance weighted
MR effect size for
' || id:ebi−a−GCST90001540' on 'pNENs'
MR Method
Inverse variance weighted
MR Egger
1/SE$_{IV}$
IV
MR leave−one−out sensitivity analysis for
' || id:ebi−a−GCST90001540' on 'pNENs'
All
MR Estimate
Inverse variance weighted
MR Egger
Simple mode
Weighted median
Weighted mode
SNP effect on pNENs
SNP effect on || id:ebi−a−GCST90001540

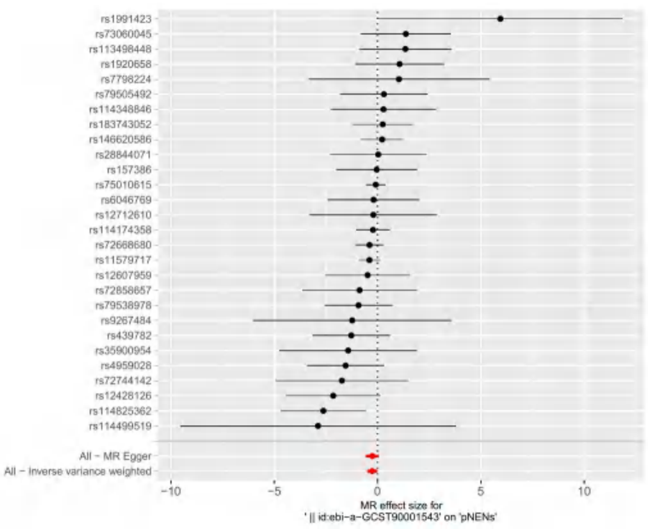
rs1991423
rs73060045
rs113498448
rs1920658
rs7798224
rs79505492
rs114348846
rs183743052
rs146620586
rs28844071
rs157386
rs75010615
rs6046769
rs12712610
rs114174358
rs72668680
rs11579717
rs12607959
rs72858657
rs79538978
rs9267484
rs439782
rs35900954
rs4959028
rs72744142
rs12428126
rs114825362
rs114499519
All − MR Egger
All − Inverse variance weighted
−10
−5
0
5
10
MR effect size for
' || id:ebi−a−GCST90001543' on 'pNENs'

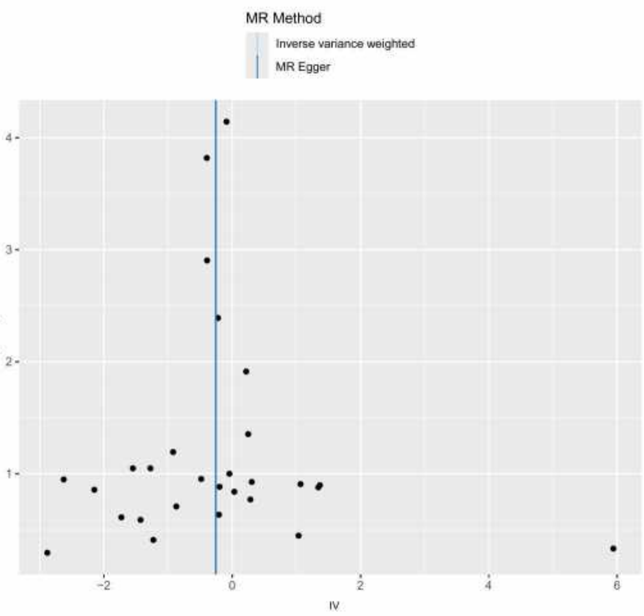
MR Method
Inverse variance weighted
MR Egger
$1/SE_{IV}$
4
3
2
1
−2
0
2
4
6
IV

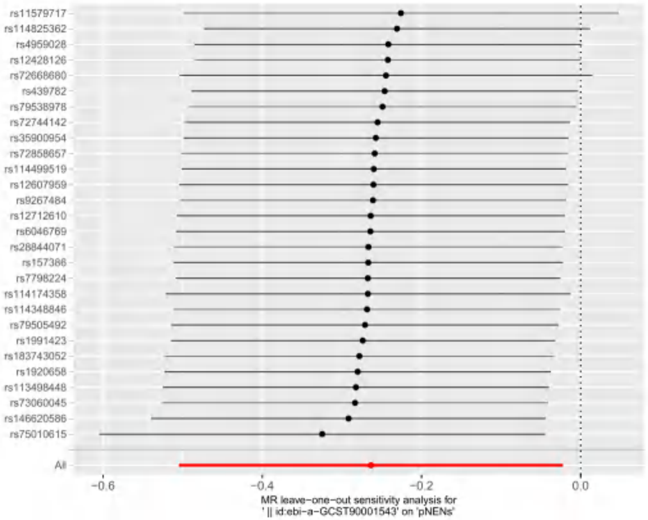
rs11579717
rs114825362
rs4959028
rs12428126
rs72668680
rs439782
rs79538978
rs72744142
rs35900954
rs72858657
rs114499519
rs12607959
rs9267484
rs12712610
rs6046769
rs28844071
rs157386
rs7798224
rs114174358
rs114348846
rs79505492
rs1991423
rs183743052
rs1920658
rs113498448
rs73060045
rs146620586
rs75010615
All
−0.6
−0.4
−0.2
0.0
MR leave−one−out sensitivity analysis for
' || id:ebi−a−GCST90001543' on 'pNENs'

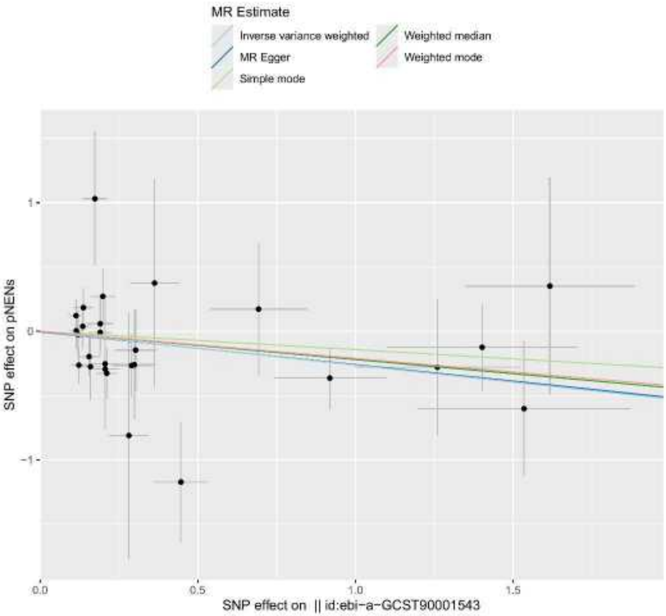
MR Estimate
Inverse variance weighted
MR Egger
Simple mode
Weighted median
Weighted mode
SNP effect on pNENs
1
0
−1
0.0
0.5
1.0
1.5
SNP effect on || id:ebi−a−GCST90001543

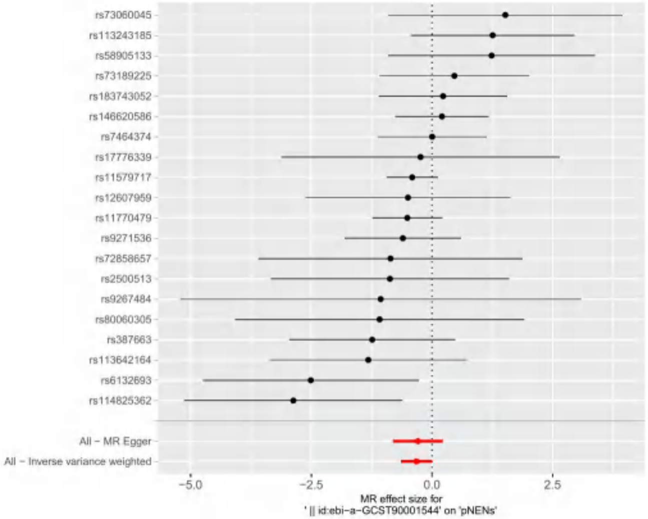
rs73060045
rs113243185
rs58905133
rs73189225
rs183743052
rs146620586
rs7464374
rs17776339
rs11579717
rs12607959
rs11770479
rs9271536
rs72858657
rs2500513
rs9267484
rs80060305
rs387663
rs113642164
rs6132693
rs114825362
All − MR Egger
All − Inverse variance weighted
−5.0
−2.5
0.0
2.5
MR effect size for
' || id:ebi−a−GCST90001544' on 'pNENs'
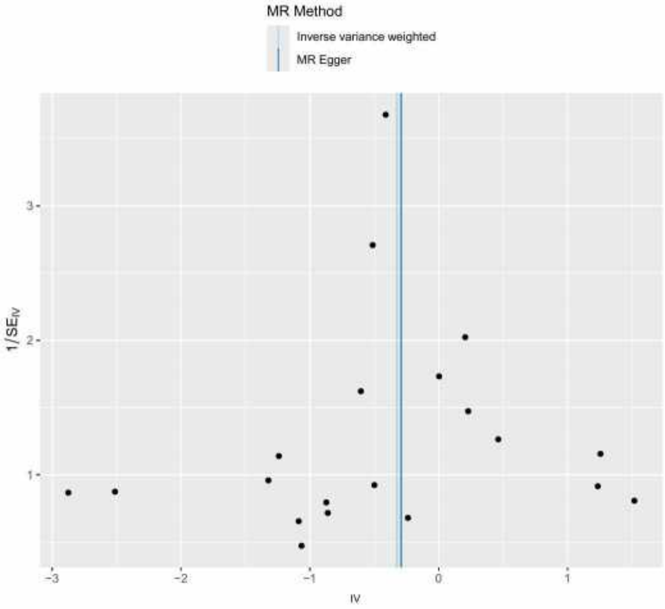
MR Method
Inverse variance weighted
MR Egger
1/SE$_{IV}$
3
2
1
−3
−2
−1
0
1
IV
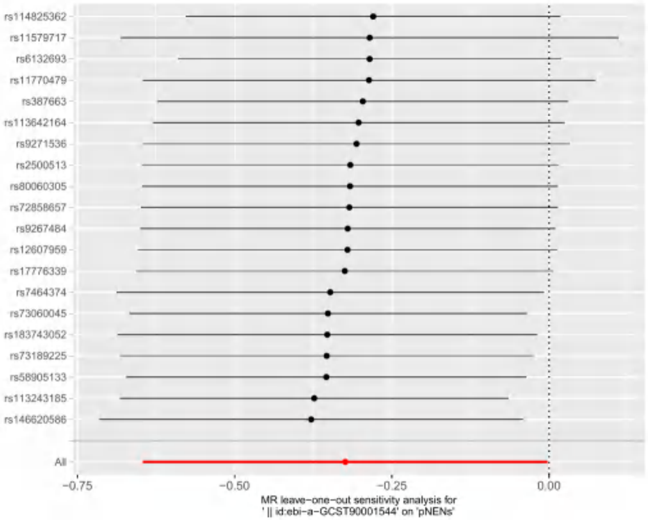
rs114825362
rs11579717
rs6132693
rs11770479
rs387663
rs113642164
rs9271536
rs2500513
rs80060305
rs72858657
rs9267484
rs12607959
rs17776339
rs7464374
rs73060045
rs183743052
rs73189225
rs58905133
rs113243185
rs146620586
All
−0.75
−0.50
−0.25
0.00
MR leave−one−out sensitivity analysis for
' || id:ebi−a−GCST90001544' on 'pNENs'
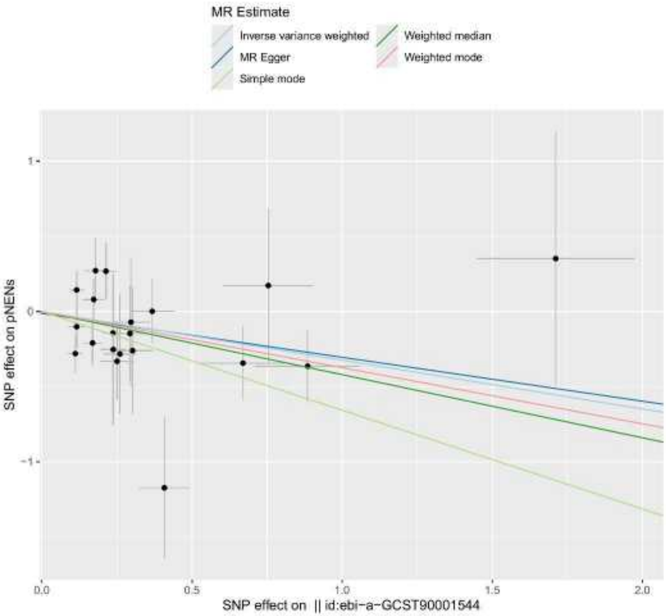
MR Estimate
Inverse variance weighted
MR Egger
Simple mode
Weighted median
Weighted mode
SNP effect on pNENs
1
0
−1
0.0
0.5
1.0
1.5
2.0
SNP effect on || id:ebi−a−GCST90001544

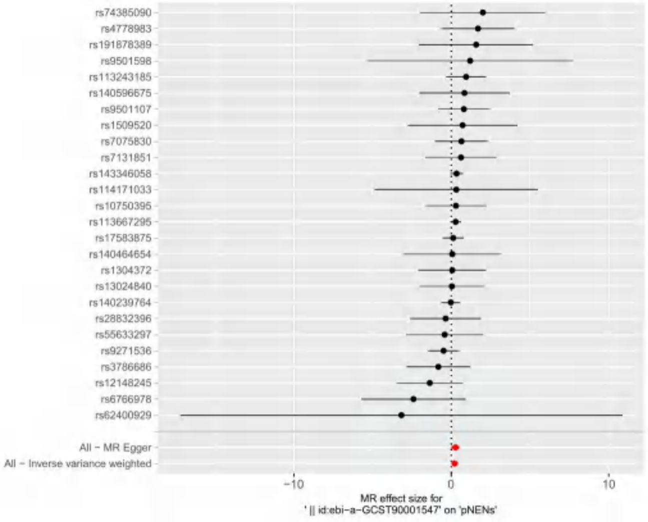
rs74385090
rs4778983
rs191878389
rs9501598
rs113243185
rs140596675
rs9501107
rs1509520
rs7075830
rs7131851
rs143346058
rs114171033
rs10750395
rs113667295
rs17583875
rs140464654
rs1304372
rs13024840
rs140239764
rs28832396
rs55633297
rs9271536
rs3786686
rs12148245
rs6766978
rs62400929
All − MR Egger
All − Inverse variance weighted
−10
0
10
MR effect size for
' || id:ebi−a−GCST90001547' on 'pNENs'

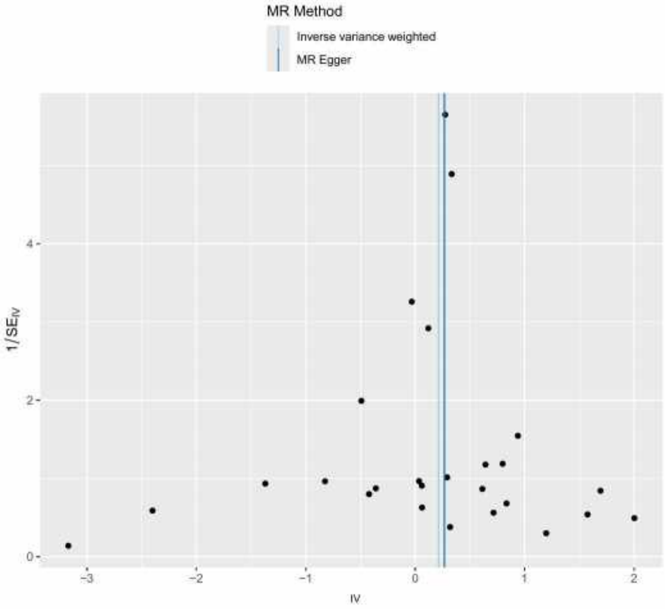
MR Method
Inverse variance weighted
MR Egger
1/SE$_{IV}$
0
2
4
−3
−2
−1
0
1
2
IV

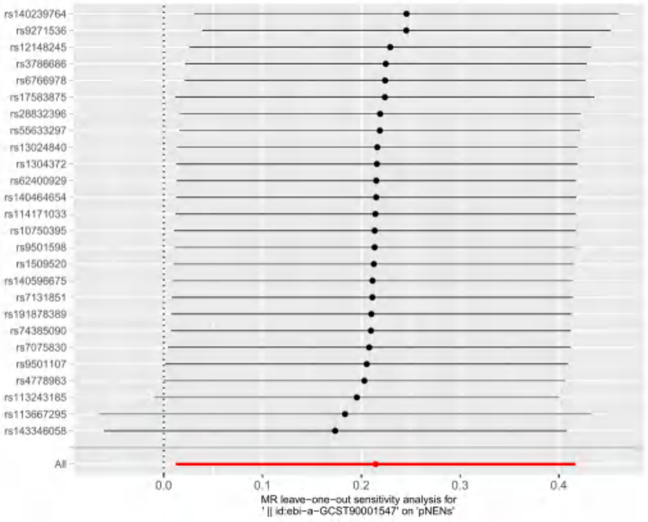
rs140239764
rs9271536
rs12148245
rs3786686
rs6766978
rs17583875
rs28832396
rs55633297
rs13024840
rs1304372
rs62400929
rs140464654
rs114171033
rs10750395
rs9501598
rs1509520
rs140596675
rs7131851
rs191878389
rs74385090
rs7075830
rs9501107
rs4778983
rs113243185
rs113667295
rs143346058
All
0.0
0.1
0.2
0.3
0.4
MR leave−one−out sensitivity analysis for
' || id:ebi−a−GCST90001547' on 'pNENs'

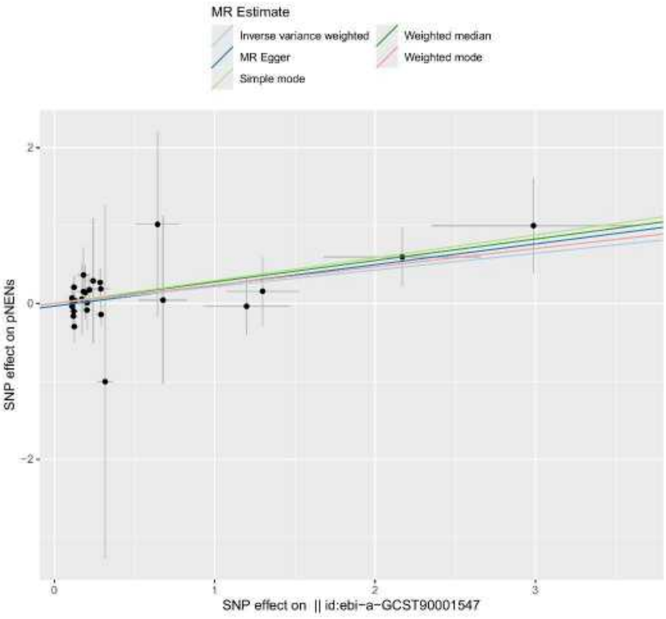
MR Estimate
Inverse variance weighted
MR Egger
Simple mode
Weighted median
Weighted mode
SNP effect on pNENs
2
0
−2
0
1
2
3
SNP effect on || id:ebi−a−GCST90001547

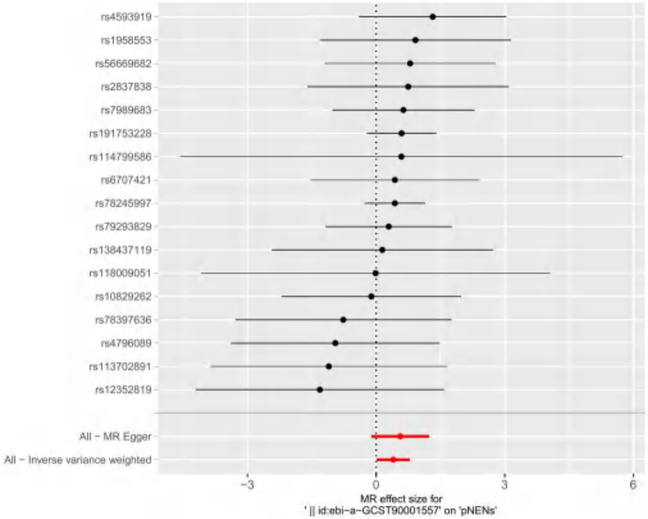
rs4593919
rs1958553
rs56669682
rs2837838
rs7989683
rs191753228
rs114799586
rs6707421
rs78245997
rs79293829
rs138437119
rs118009051
rs10829262
rs78397636
rs4796089
rs113702891
rs12352819
All − MR Egger
All − Inverse variance weighted
−3
0
3
6
MR effect size for
' || id:ebi−a−GCST90001557' on 'pNENs'

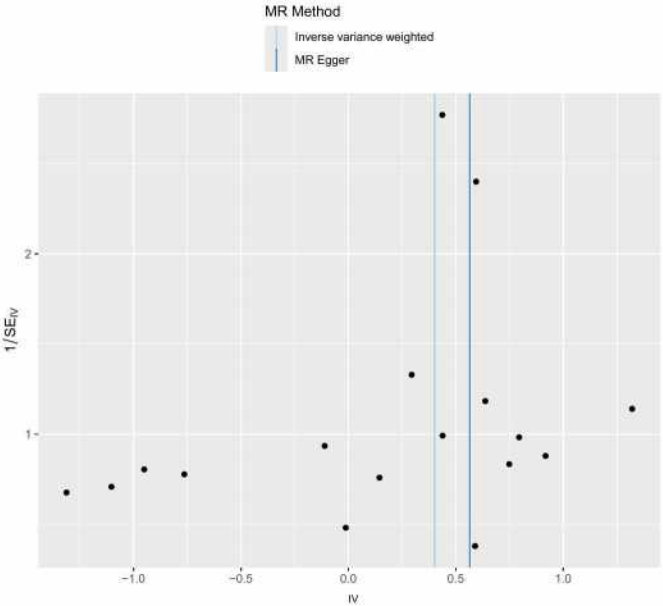
MR Method
Inverse variance weighted
MR Egger
1/SE_IV
2
1
−1.0
−0.5
0.0
0.5
1.0
IV

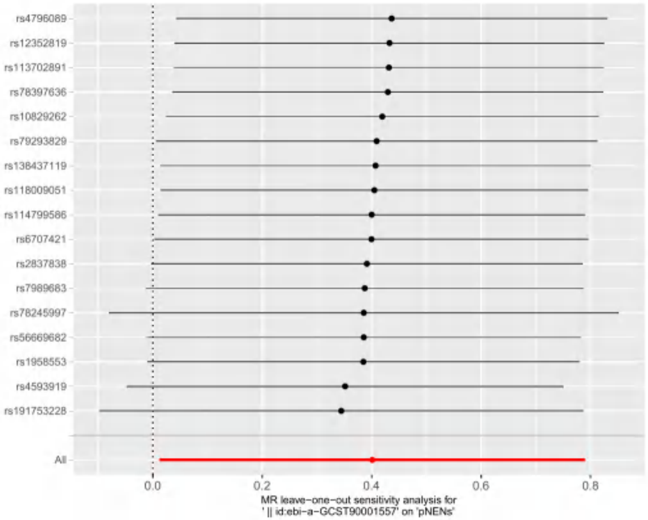
rs4796089
rs12352819
rs113702891
rs78397636
rs10829262
rs79293829
rs138437119
rs118009051
rs114799586
rs6707421
rs2837838
rs7989683
rs78245997
rs56669682
rs1958553
rs4593919
rs191753228
All
0.0
0.2
0.4
0.6
0.8
MR leave−one−out sensitivity analysis for
' || id:ebi−a−GCST90001557' on 'pNENs'

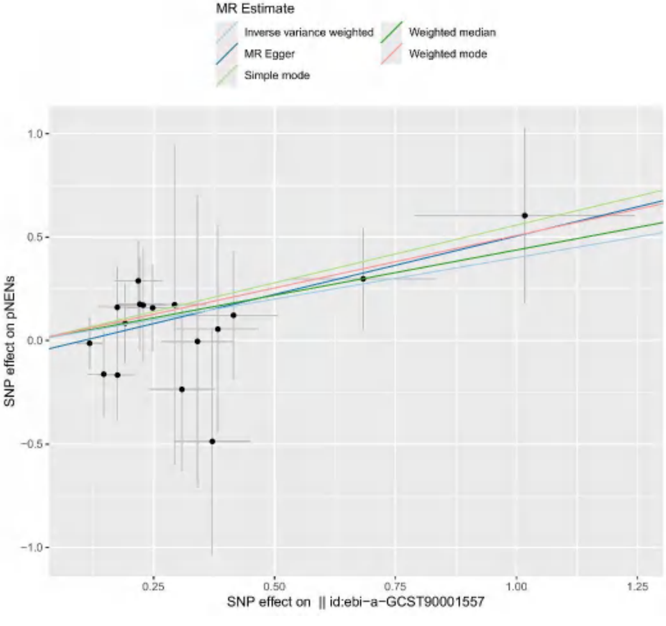
MR Estimate
Inverse variance weighted
MR Egger
Simple mode
Weighted median
Weighted mode
SNP effect on pNENs
1.0
0.5
0.0
−0.5
−1.0
0.25
0.50
0.75
1.00
1.25
SNP effect on || id:ebi−a−GCST90001557

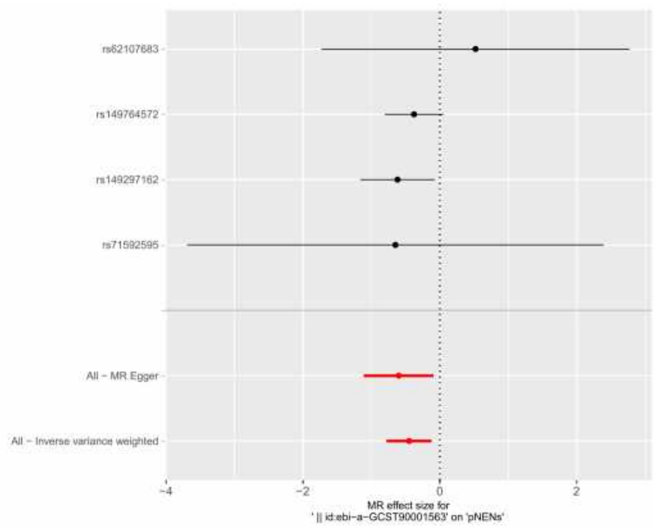
rs62107683
rs149764572
rs149297162
rs71592595
All − MR Egger
All − Inverse variance weighted
−4
−2
0
2
MR effect size for
' || id:ebi−a−GCST90001563' on 'pNENs'

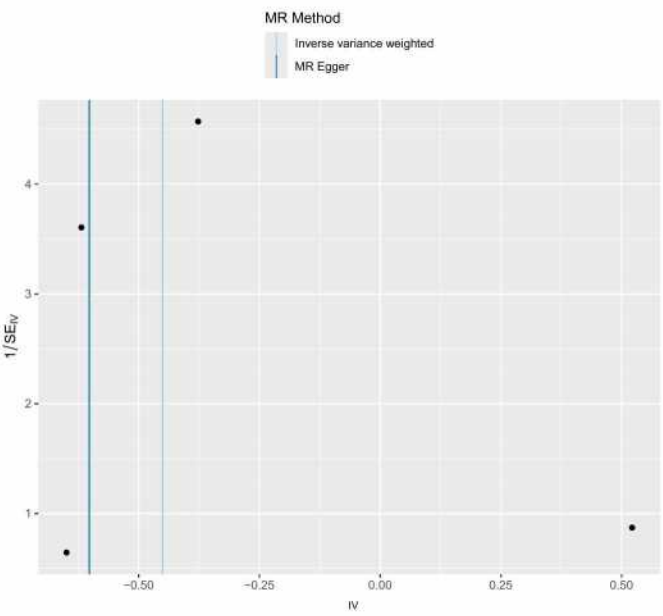
MR Method
Inverse variance weighted
MR Egger
1/SE_IV
4
3
2
1
−0.50
−0.25
0.00
0.25
0.50
IV

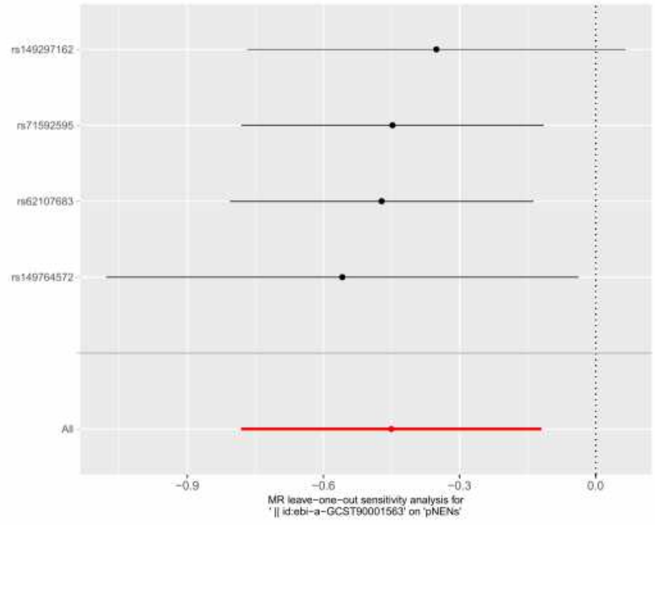
rs149297162
rs71592595
rs62107683
rs149764572
All
−0.9
−0.6
−0.3
0.0
MR leave−one−out sensitivity analysis for
' || id:ebi−a−GCST90001563' on 'pNENs'

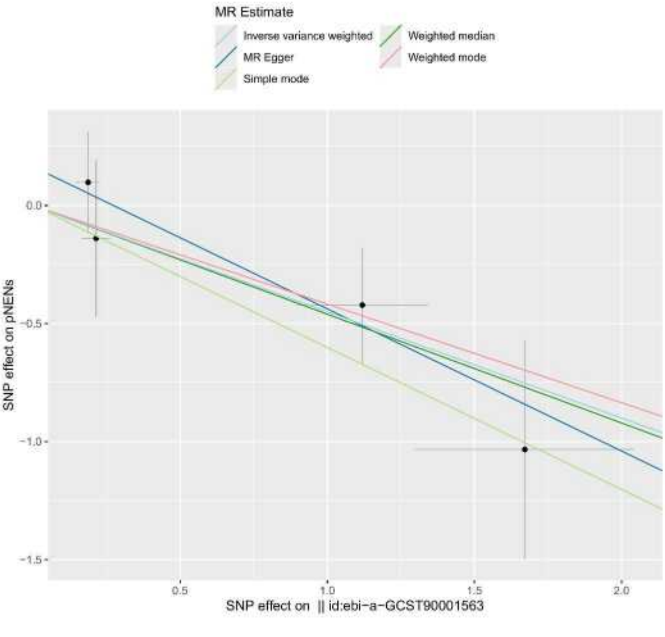
MR Estimate
Inverse variance weighted
MR Egger
Simple mode
Weighted median
Weighted mode
SNP effect on pNENs
0.0
−0.5
−1.0
−1.5
0.5
1.0
1.5
2.0
SNP effect on || id:ebi−a−GCST90001563

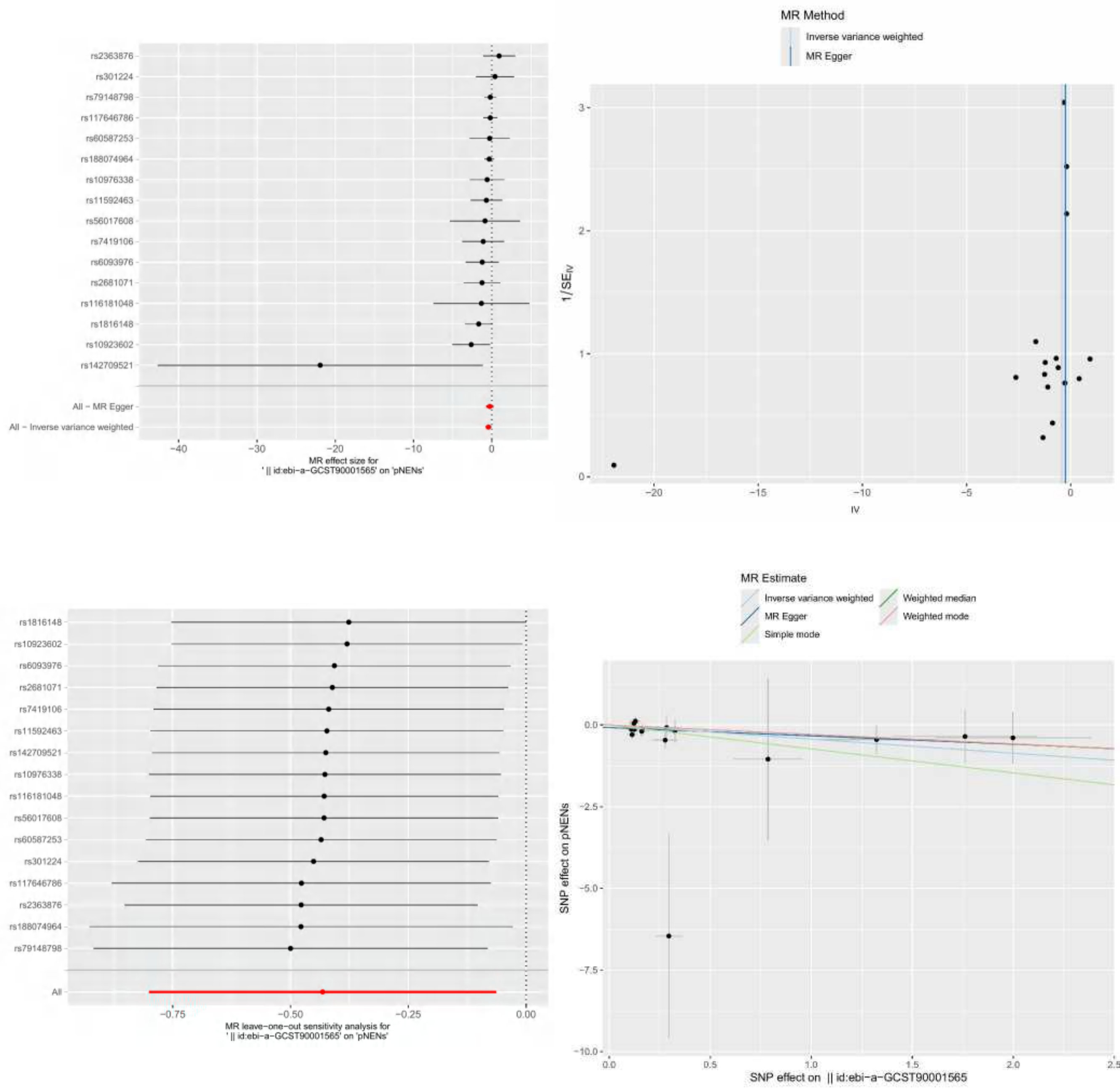

rs2363876
rs301224
rs79148798
rs117646786
rs60587253
rs188074964
rs10976338
rs11592463
rs56017608
rs7419106
rs6093976
rs2681071
rs116181048
rs1816148
rs10923602
rs142709521
All − MR Egger
All − Inverse variance weighted
−40
−30
−20
−10
0
MR effect size for
' || id:ebi−a−GCST90001565' on 'pNENs'
MR Method
Inverse variance weighted
MR Egger
1/SE_IV
IV
−20
−15
−10
−5
0
rs1816148
rs10923602
rs6093976
rs2681071
rs7419106
rs11592463
rs142709521
rs10976338
rs116181048
rs56017608
rs60587253
rs301224
rs117646786
rs2363876
rs188074964
rs79148798
All
−0.75
−0.50
−0.25
0.00
MR leave−one−out sensitivity analysis for
' || id:ebi−a−GCST90001565' on 'pNENs'
MR Estimate
Inverse variance weighted
MR Egger
Simple mode
Weighted median
Weighted mode
SNP effect on pNENs
SNP effect on || id:ebi−a−GCST90001565
0.0
−2.5
−5.0
−7.5
−10.0
0.5
1.0
1.5
2.0
2.5

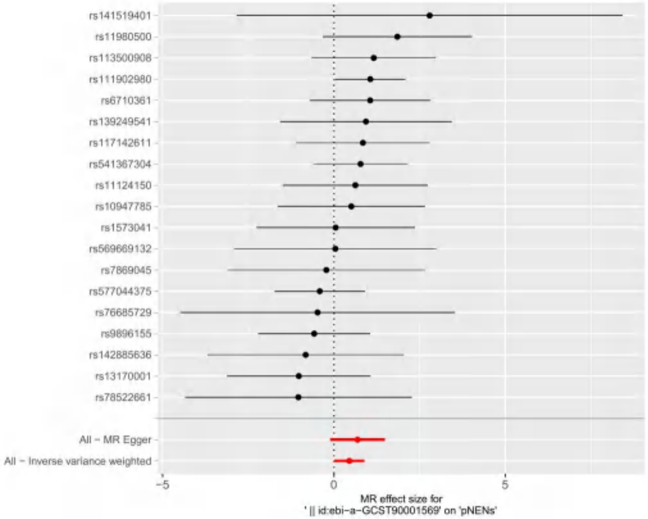
rs141519401
rs11980500
rs113500908
rs111902980
rs6710361
rs139249541
rs117142611
rs541367304
rs11124150
rs10947785
rs1573041
rs569669132
rs7869045
rs577044375
rs76685729
rs9896155
rs142885636
rs13170001
rs78522661
All − MR Egger
All − Inverse variance weighted
−5
0
5
MR effect size for
' || id:ebi−a−GCST90001569' on 'pNENs'

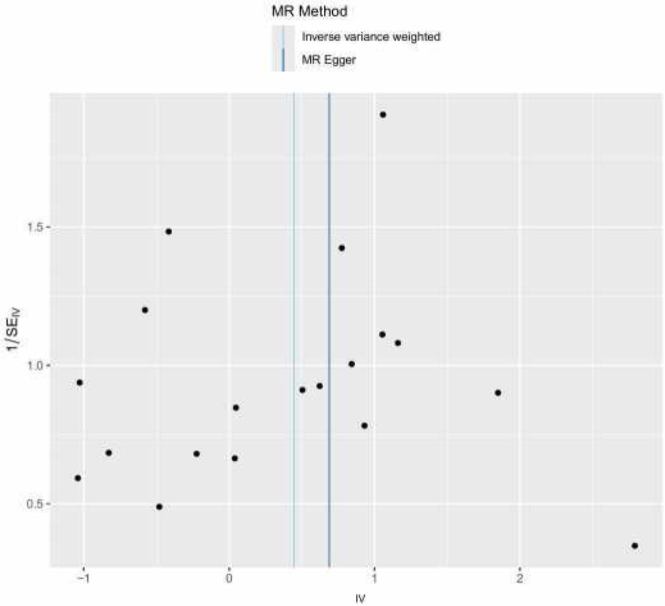
MR Method
Inverse variance weighted
MR Egger
1/SE$_{IV}$
1.5
1.0
0.5
−1
0
1
2
IV

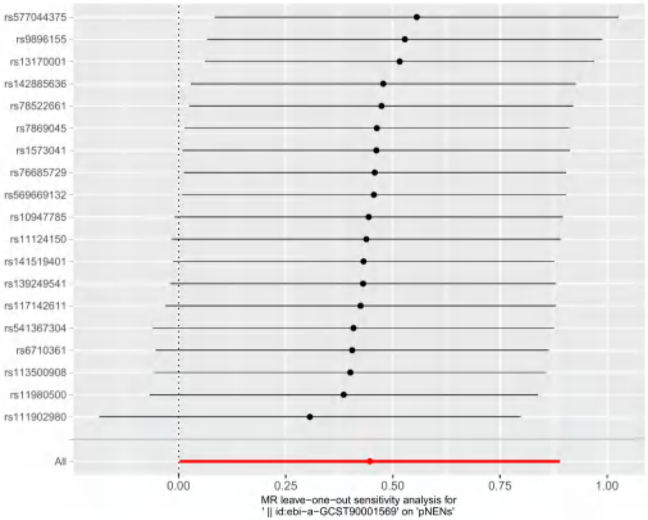
rs577044375
rs9896155
rs13170001
rs142885636
rs78522661
rs7869045
rs1573041
rs76685729
rs569669132
rs10947785
rs11124150
rs141519401
rs139249541
rs117142611
rs541367304
rs6710361
rs113500908
rs11980500
rs111902980
All
0.00
0.25
0.50
0.75
1.00
MR leave−one−out sensitivity analysis for
' || id:ebi−a−GCST90001569' on 'pNENs'

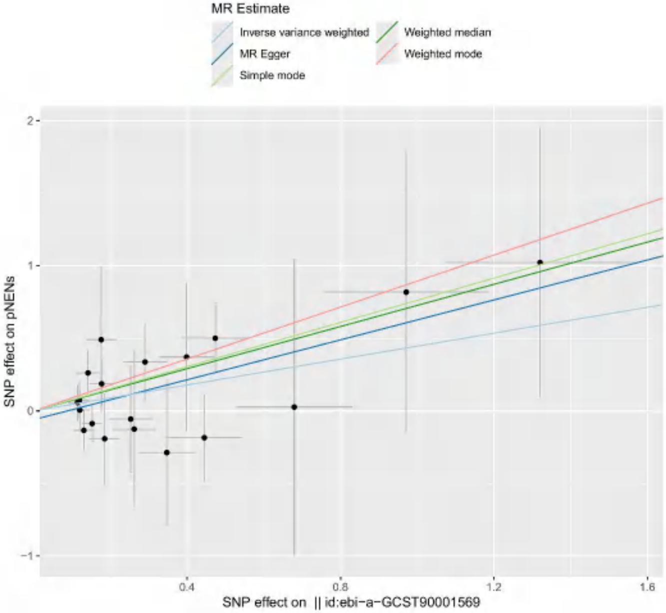
MR Estimate
Inverse variance weighted
MR Egger
Simple mode
Weighted median
Weighted mode
2
1
0
−1
SNP effect on pNENs
0.4
0.8
1.2
1.6
SNP effect on || id:ebi−a−GCST90001569

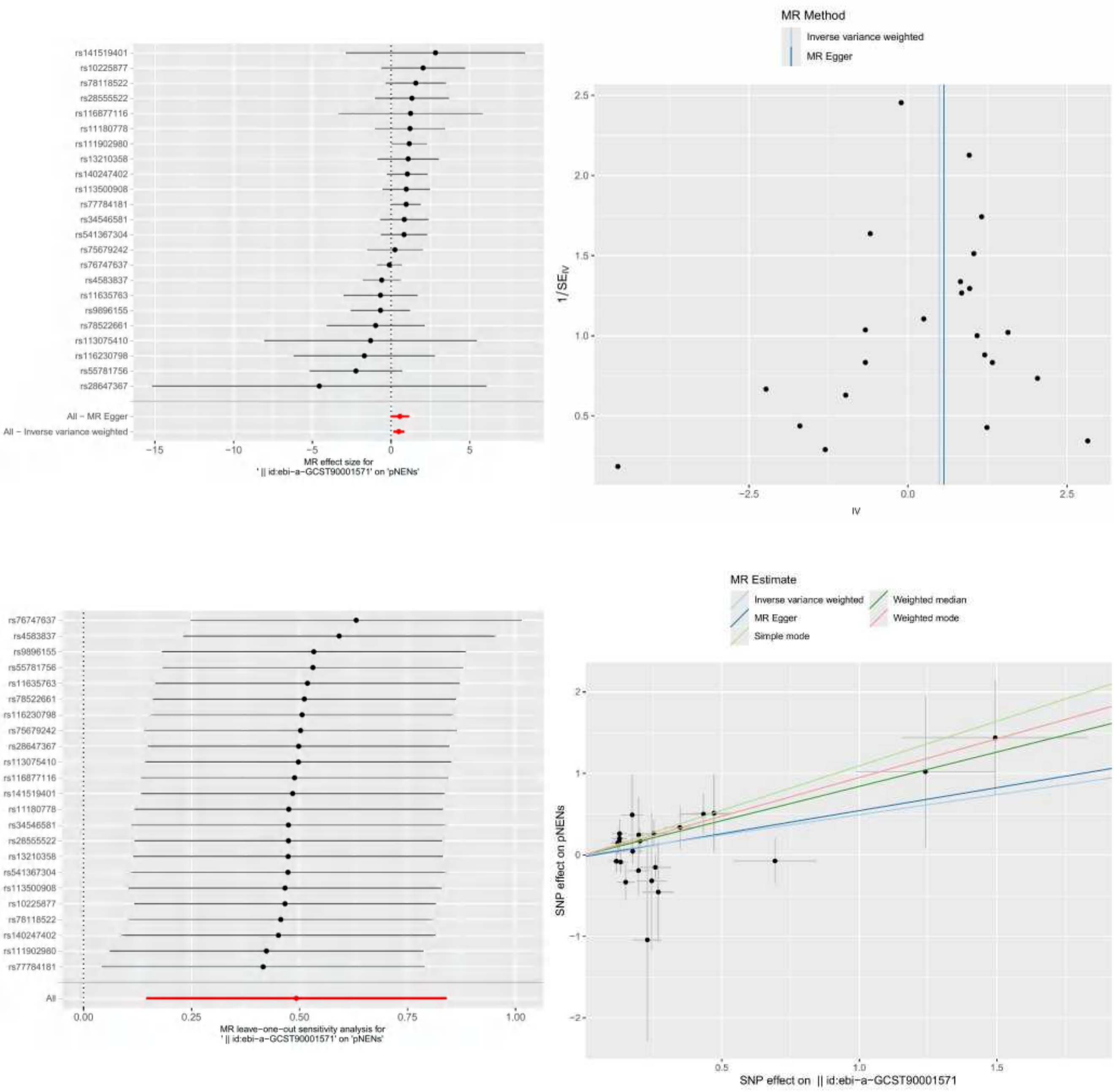

rs141519401
rs10225877
rs78118522
rs28555522
rs116877116
rs11180778
rs111902980
rs13210358
rs140247402
rs113500908
rs77784181
rs34546581
rs541367304
rs75679242
rs76747637
rs4583837
rs11635763
rs9896155
rs78522661
rs113075410
rs116230798
rs55781756
rs28647367
All − MR Egger
All − Inverse variance weighted
−15
−10
−5
0
5
MR effect size for
' || id:ebi−a−GCST90001571' on 'pNENs'
MR Method
Inverse variance weighted
MR Egger
1/SE_{IV}
2.5
2.0
1.5
1.0
0.5
−2.5
0.0
2.5
IV
rs76747637
rs4583837
rs9896155
rs55781756
rs11635763
rs78522661
rs116230798
rs75679242
rs28647367
rs113075410
rs116877116
rs141519401
rs11180778
rs34546581
rs28555522
rs13210358
rs541367304
rs113500908
rs10225877
rs78118522
rs140247402
rs111902980
rs77784181
All
0.00
0.25
0.50
0.75
1.00
MR leave−one−out sensitivity analysis for
' || id:ebi−a−GCST90001571' on 'pNENs'
MR Estimate
Inverse variance weighted
MR Egger
Simple mode
Weighted median
Weighted mode
SNP effect on pNENs
2
1
0
−1
−2
0.5
1.0
1.5
SNP effect on || id:ebi−a−GCST90001571

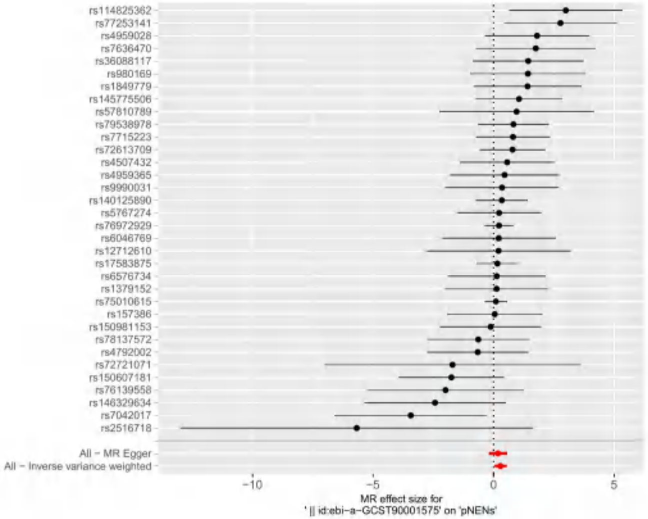
rs114825362
rs77253141
rs4959028
rs7636470
rs36088117
rs980169
rs1849779
rs145775506
rs57810789
rs79538978
rs7715223
rs72613709
rs4507432
rs4959365
rs9990031
rs140125890
rs5767274
rs76972929
rs6046769
rs12712610
rs17583875
rs6576734
rs1379152
rs75010615
rs157386
rs150981153
rs78137572
rs4792002
rs72721071
rs150607181
rs76139558
rs146329634
rs7042017
rs2516718
All − MR Egger
All − Inverse variance weighted
−10
−5
0
5
MR effect size for
' || id:ebi−a−GCST90001575' on 'pNENs'

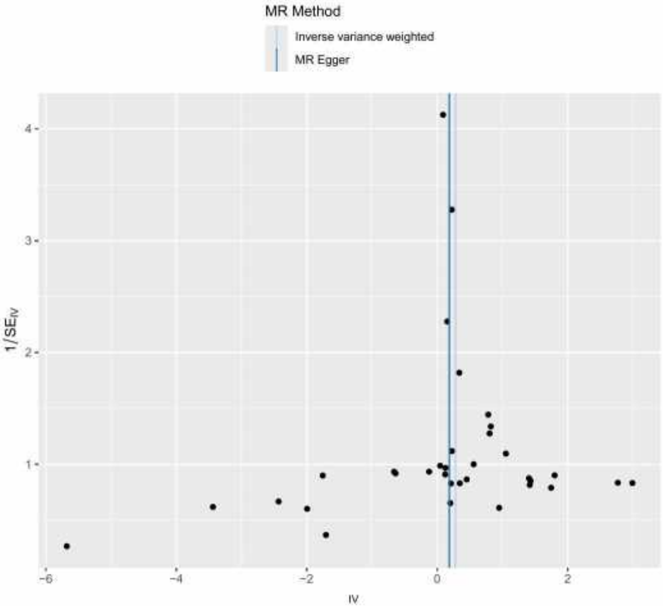
MR Method
Inverse variance weighted
MR Egger
1/SE$_{IV}$
4
3
2
1
−6
−4
−2
0
2
IV

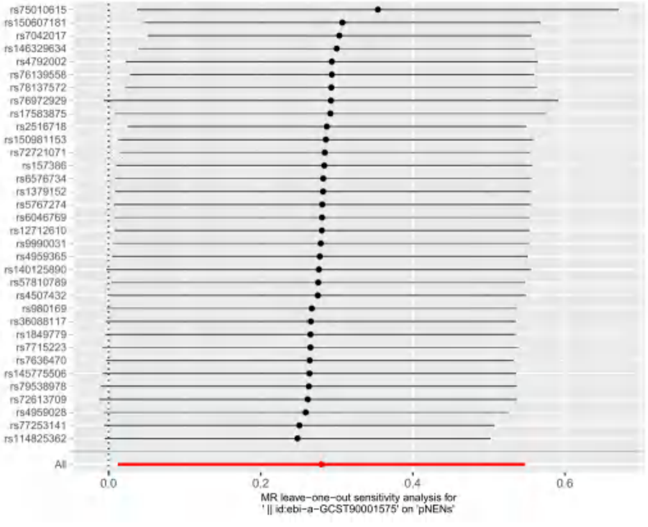
rs75010615
rs150607181
rs7042017
rs146329634
rs4792002
rs76139558
rs78137572
rs76972929
rs17583875
rs2516718
rs150981153
rs72721071
rs157386
rs6576734
rs1379152
rs5767274
rs6046769
rs12712610
rs9990031
rs4959365
rs140125890
rs57810789
rs4507432
rs980169
rs36088117
rs1849779
rs7715223
rs7636470
rs145775506
rs79538978
rs72613709
rs4959028
rs77253141
rs114825362
All
0.0
0.2
0.4
0.6
MR leave−one−out sensitivity analysis for
' || id:ebi−a−GCST90001575' on 'pNENs'

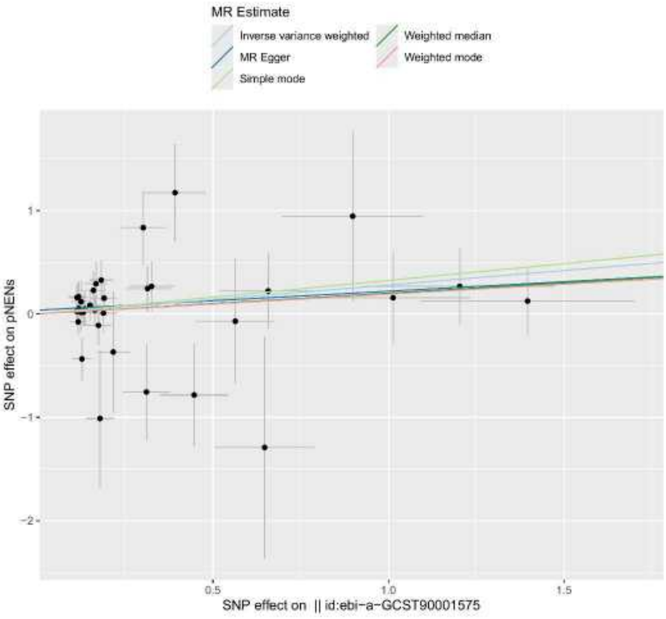
MR Estimate
Inverse variance weighted
MR Egger
Simple mode
Weighted median
Weighted mode
SNP effect on pNENs
1
0
−1
−2
0.5
1.0
1.5
SNP effect on || id:ebi−a−GCST90001575

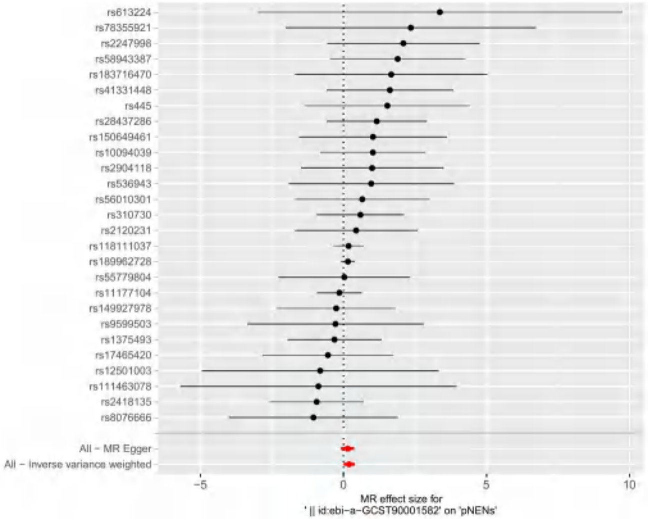
rs613224
rs78355921
rs2247998
rs58943387
rs183716470
rs41331448
rs445
rs28437286
rs150649461
rs10094039
rs2904118
rs536943
rs56010301
rs310730
rs2120231
rs118111037
rs189962728
rs55779804
rs11177104
rs149927978
rs9599503
rs1375493
rs17465420
rs12501003
rs111463078
rs2418135
rs8076666
All − MR Egger
All − Inverse variance weighted
-5
0
5
10
MR effect size for
' || id:ebi−a−GCST90001582' on 'pNENs'

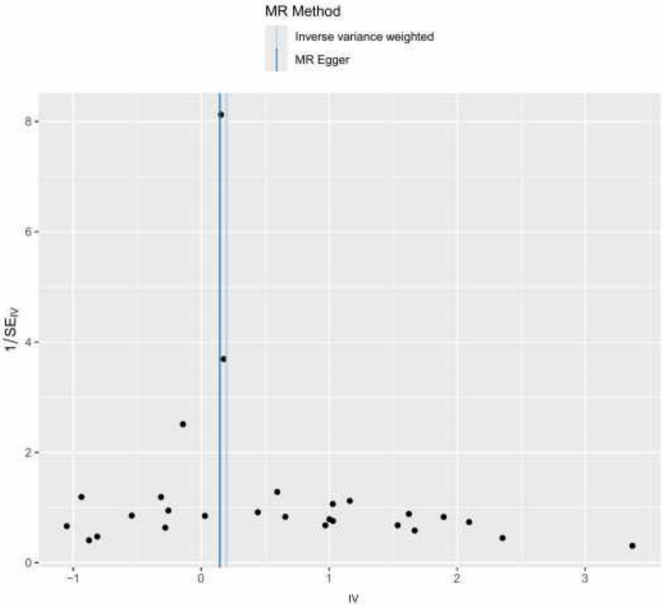
MR Method
Inverse variance weighted
MR Egger
1/SE$_{IV}$
IV
0
2
4
6
8
-1
1
3

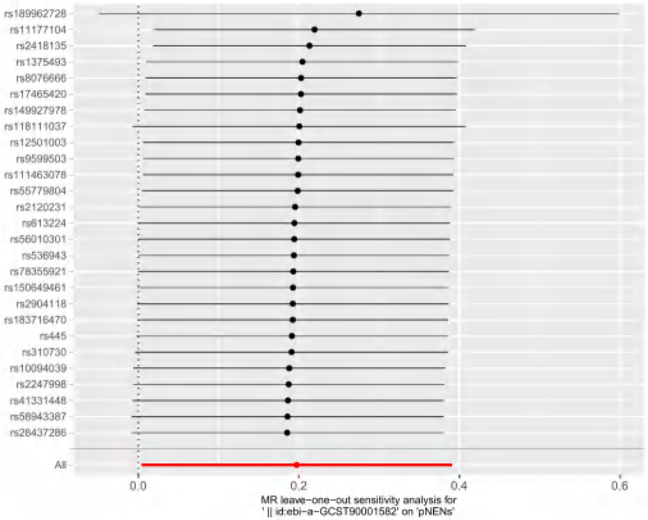
rs189962728
rs11177104
rs2418135
rs1375493
rs8076666
rs17465420
rs149927978
rs118111037
rs12501003
rs9599503
rs111463078
rs55779804
rs2120231
rs613224
rs56010301
rs536943
rs78355921
rs150649461
rs2904118
rs183716470
rs445
rs310730
rs10094039
rs2247998
rs41331448
rs58943387
rs28437286
All
0.0
0.2
0.4
0.6
MR leave−one−out sensitivity analysis for
' || id:ebi−a−GCST90001582' on 'pNENs'

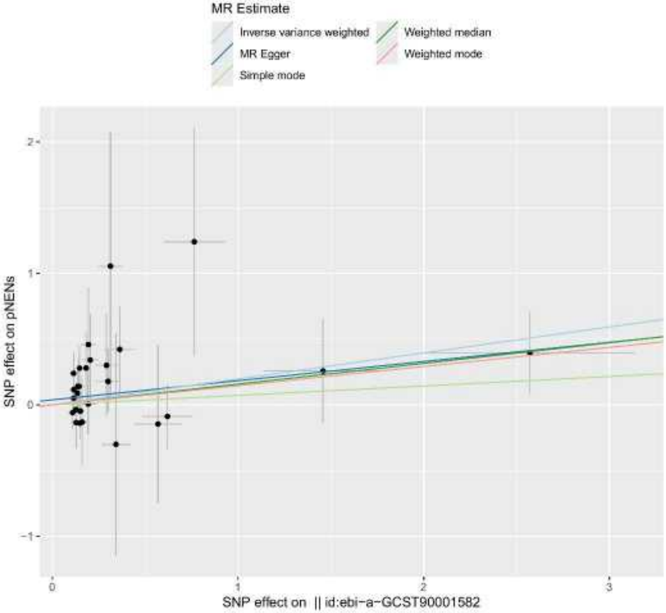
MR Estimate
Inverse variance weighted
MR Egger
Simple mode
Weighted median
Weighted mode
SNP effect on pNENs
SNP effect on || id:ebi−a−GCST90001582
-1
0
1
2
3

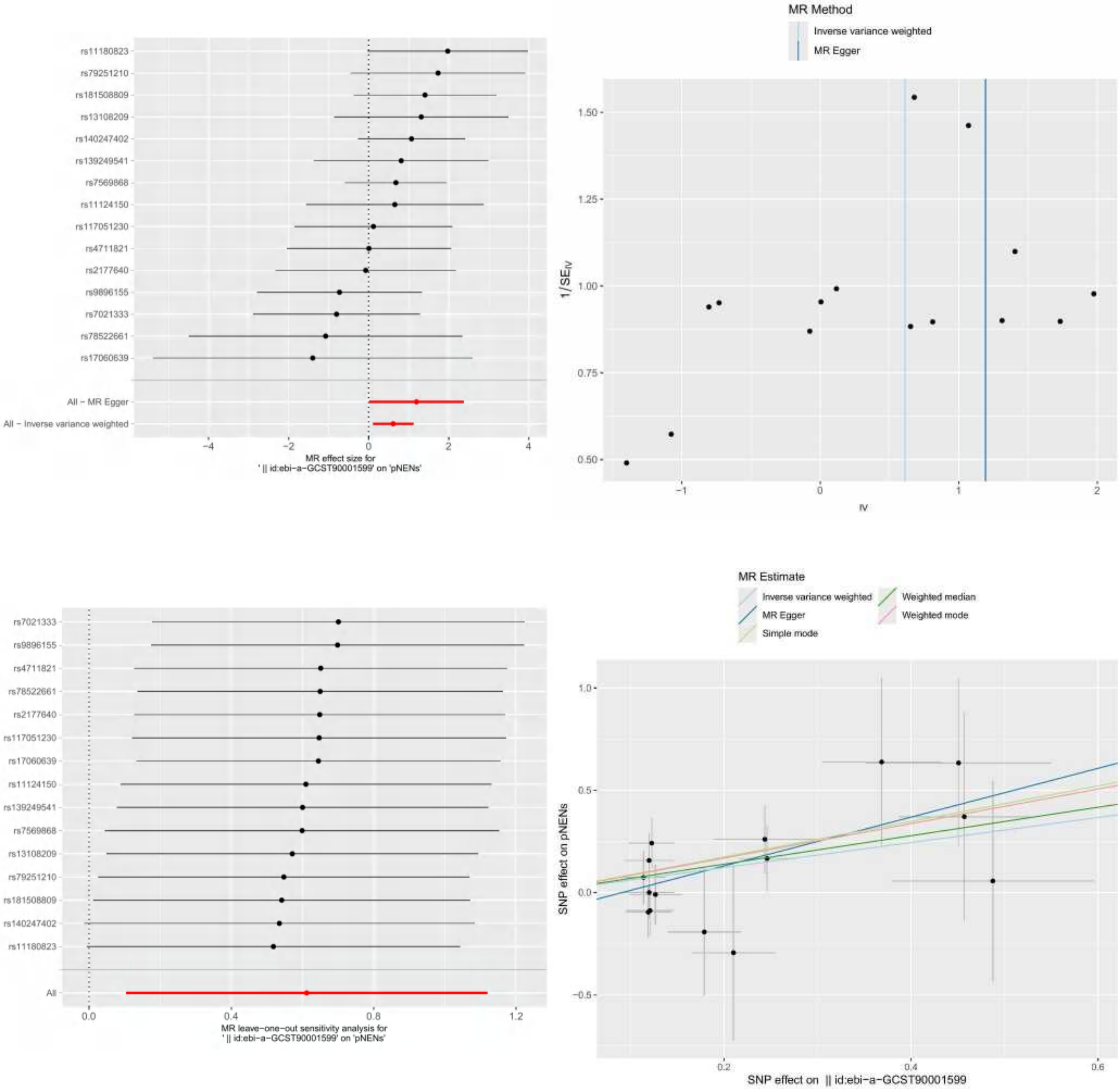
rs11180823
rs79251210
rs181508809
rs13108209
rs140247402
rs139249541
rs7569868
rs11124150
rs117051230
rs4711821
rs2177640
rs9896155
rs7021333
rs78522661
rs17060639
All − MR Egger
All − Inverse variance weighted
MR effect size for
' || id:ebi−a−GCST90001599' on 'pNENs'
MR Method
Inverse variance weighted
MR Egger
1/SE$_{IV}$
IV
rs7021333
rs9896155
rs4711821
rs78522661
rs2177640
rs117051230
rs17060639
rs11124150
rs139249541
rs7569868
rs13108209
rs79251210
rs181508809
rs140247402
rs11180823
All
MR leave−one−out sensitivity analysis for
' || id:ebi−a−GCST90001599' on 'pNENs'
MR Estimate
Inverse variance weighted
MR Egger
Simple mode
Weighted median
Weighted mode
SNP effect on pNENs
SNP effect on || id:ebi−a−GCST90001599

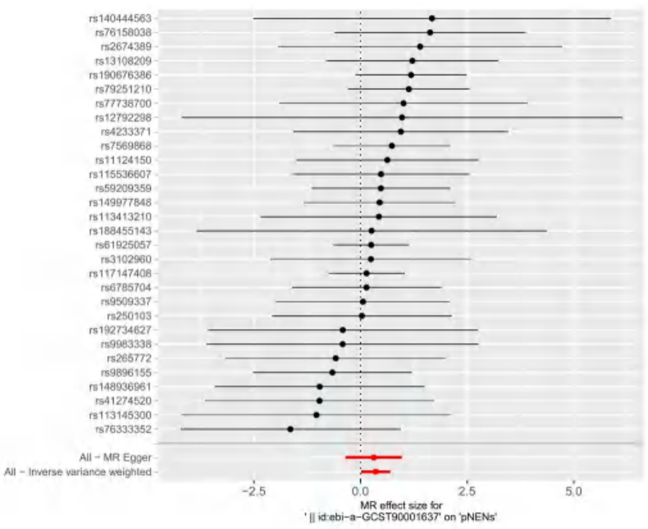
rs140444563
rs76158038
rs2674389
rs13108209
rs190676386
rs79251210
rs77738700
rs12792298
rs4233371
rs7569868
rs11124150
rs115536607
rs59209359
rs149977848
rs113413210
rs188455143
rs61925057
rs3102960
rs117147408
rs6785704
rs9509337
rs250103
rs192734627
rs9983338
rs265772
rs9896155
rs148936961
rs41274520
rs113145300
rs76333352
All − MR Egger
All − Inverse variance weighted
-2.5
0.0
2.5
5.0
MR effect size for
' || id:ebi-a-GCST90001637' on 'pNENs'

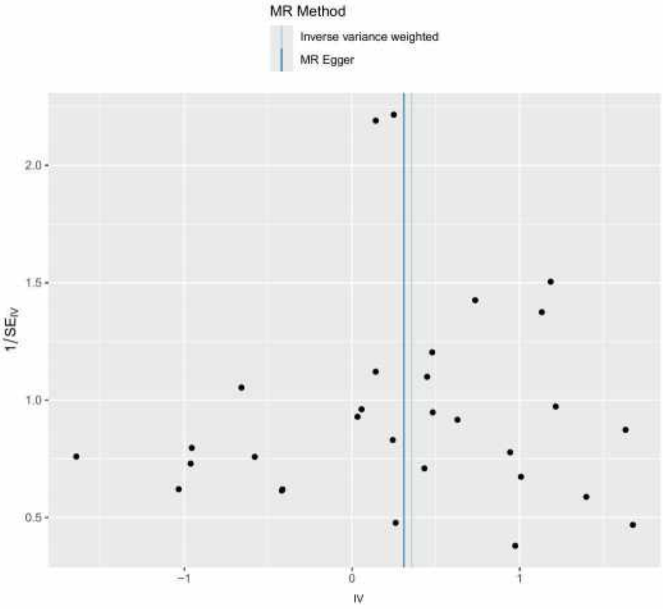
MR Method
Inverse variance weighted
MR Egger
2.0
1.5
1.0
0.5
$1/SE_{IV}$
−1
0
1
IV

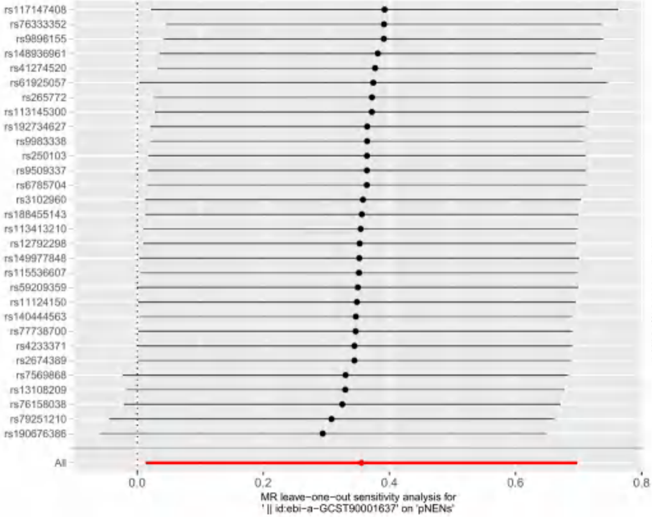
rs117147408
rs76333352
rs9896155
rs148936961
rs41274520
rs61925057
rs265772
rs113145300
rs192734627
rs9983338
rs250103
rs9509337
rs6785704
rs3102960
rs188455143
rs113413210
rs12792298
rs149977848
rs115536607
rs59209359
rs11124150
rs140444563
rs77738700
rs4233371
rs2674389
rs7569868
rs13108209
rs76158038
rs79251210
rs190676386
All
0.0
0.2
0.4
0.6
0.8
MR leave-one-out sensitivity analysis for
' || id:ebi-a-GCST90001637' on 'pNENs'

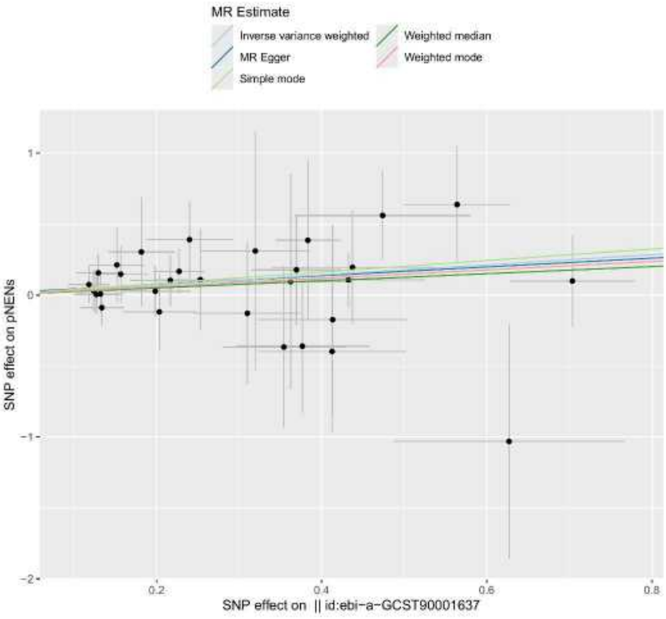
MR Estimate
Inverse variance weighted
MR Egger
Simple mode
Weighted median
Weighted mode
1
0
−1
−2
SNP effect on pNENs
0.2
0.4
0.6
0.8
SNP effect on || id:ebi-a-GCST90001637

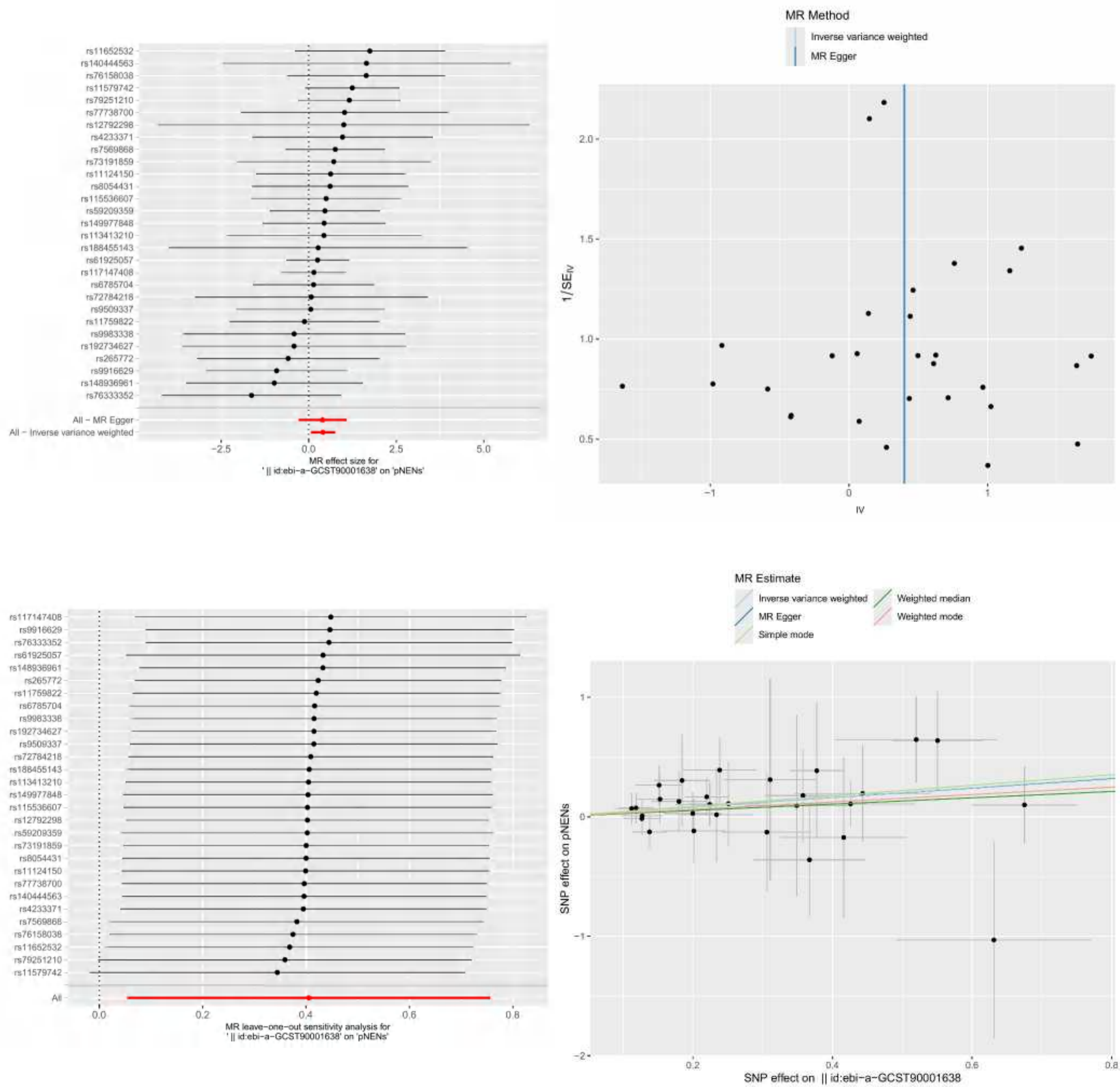
rs11652532
rs140444563
rs76158038
rs11579742
rs79251210
rs77738700
rs12792298
rs4233371
rs7569868
rs73191859
rs11124150
rs8054431
rs115536607
rs59209359
rs149977848
rs113413210
rs188455143
rs61925057
rs117147408
rs6785704
rs72784218
rs9509337
rs11759822
rs9983338
rs192734627
rs265772
rs9916629
rs148936961
rs76333352
All − MR Egger
All − Inverse variance weighted
-2.5
0.0
2.5
5.0
MR effect size for
' || id:ebi-a-GCST90001638' on 'pNENs'
MR Method
Inverse variance weighted
MR Egger
1/SE$_{IV}$
IV
MR leave-one-out sensitivity analysis for
' || id:ebi-a-GCST90001638' on 'pNENs'
All
MR Estimate
Inverse variance weighted
MR Egger
Simple mode
Weighted median
Weighted mode
SNP effect on pNENs
SNP effect on || id:ebi-a-GCST90001638

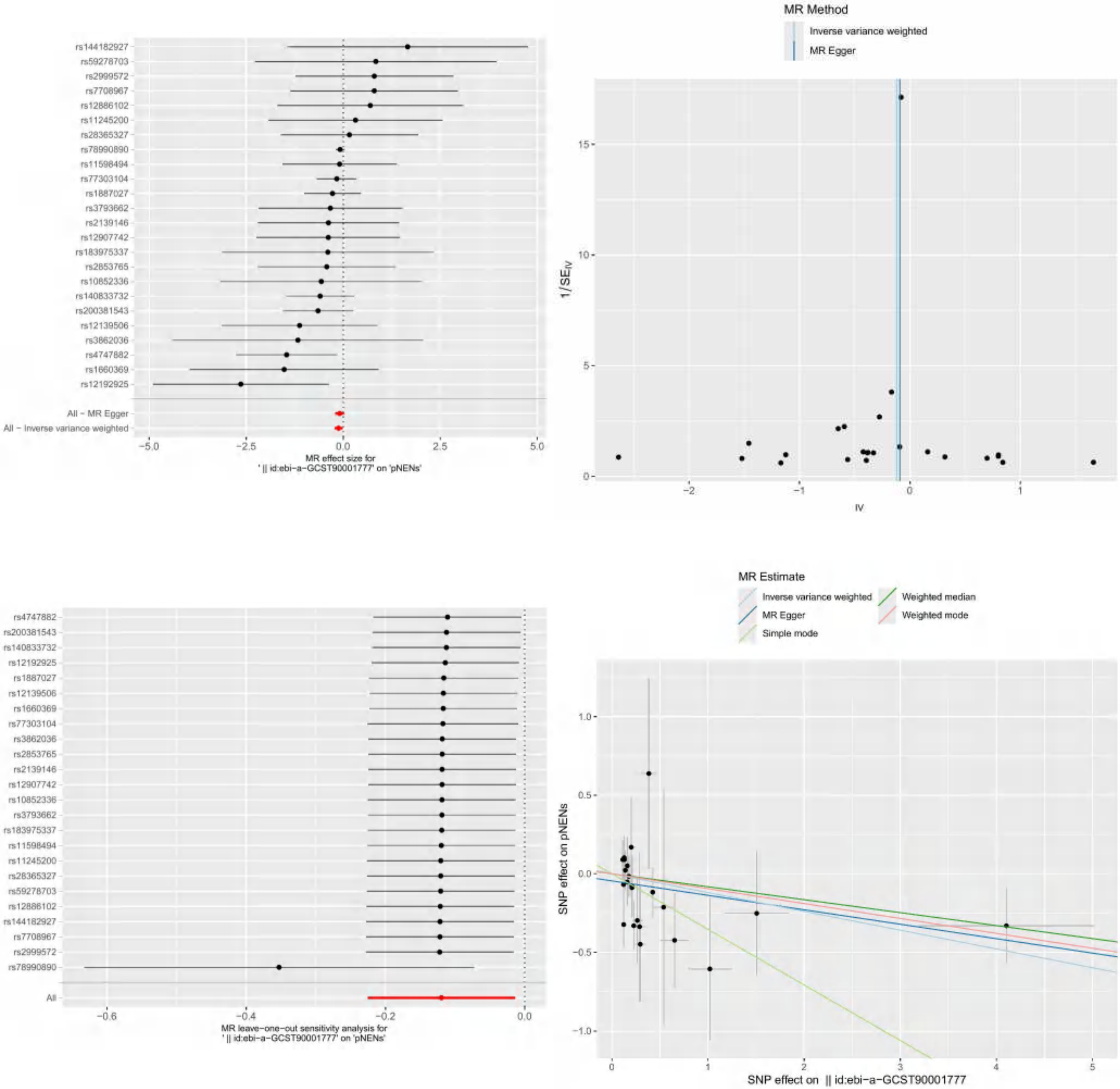
rs144182927
rs59278703
rs2999572
rs7708967
rs12886102
rs11245200
rs28365327
rs78990890
rs11598494
rs77303104
rs1887027
rs3793662
rs2139146
rs12907742
rs183975337
rs2853765
rs10852336
rs140833732
rs200381543
rs12139506
rs3862036
rs4747882
rs1660369
rs12192925
All − MR Egger
All − Inverse variance weighted
−5.0
−2.5
0.0
2.5
5.0
MR effect size for
' || id:ebi−a−GCST90001777' on 'pNENs'
MR Method
Inverse variance weighted
MR Egger
1/SE_IV
15
10
5
0
−2
−1
0
1
IV
rs4747882
rs200381543
rs140833732
rs12192925
rs1887027
rs12139506
rs1660369
rs77303104
rs3862036
rs2853765
rs2139146
rs12907742
rs10852336
rs3793662
rs183975337
rs11598494
rs11245200
rs28365327
rs59278703
rs12886102
rs144182927
rs7708967
rs2999572
rs78990890
All
−0.6
−0.4
−0.2
0.0
MR leave−one−out sensitivity analysis for
' || id:ebi−a−GCST90001777' on 'pNENs'
MR Estimate
Inverse variance weighted
MR Egger
Simple mode
Weighted median
Weighted mode
SNP effect on pNENs
1.0
0.5
0.0
−0.5
−1.0
0
1
2
3
4
5
SNP effect on || id:ebi−a−GCST90001777

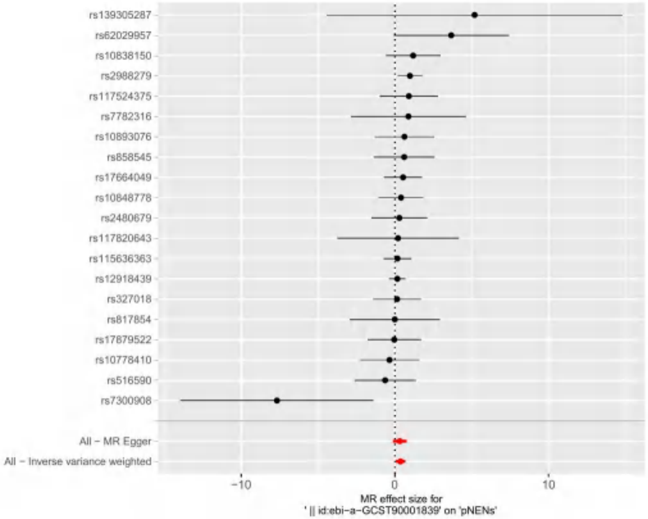
rs139305287
rs62029957
rs10838150
rs2988279
rs117524375
rs7782316
rs10893076
rs858545
rs17664049
rs10848778
rs2480679
rs117820643
rs115636363
rs12918439
rs327018
rs817854
rs17879522
rs10778410
rs516590
rs7300908
All − MR Egger
All − Inverse variance weighted
−10
0
10
MR effect size for
' || id:ebi−a−GCST90001839' on 'pNENs'

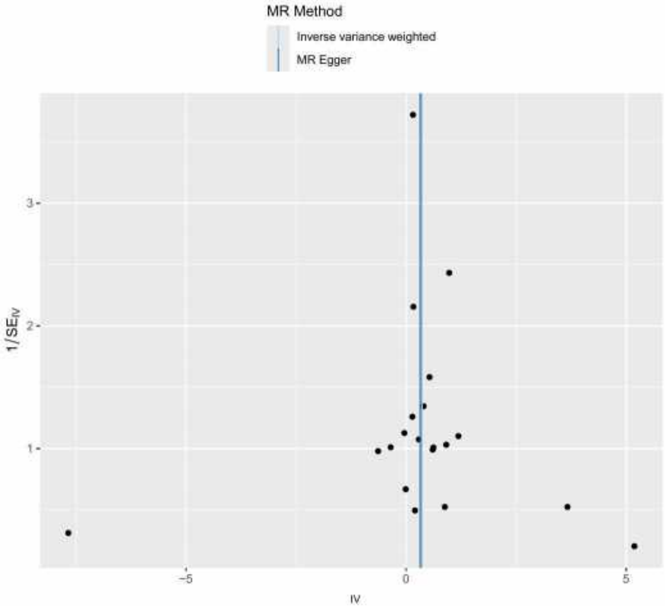
MR Method
Inverse variance weighted
MR Egger
1/SE$_{IV}$
3
2
1
−5
0
5
IV

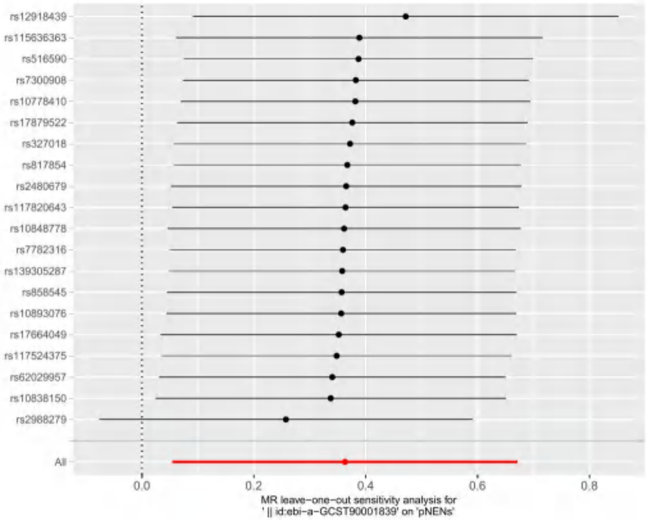
rs12918439
rs115636363
rs516590
rs7300908
rs10778410
rs17879522
rs327018
rs817854
rs2480679
rs117820643
rs10848778
rs7782316
rs139305287
rs858545
rs10893076
rs17664049
rs117524375
rs62029957
rs10838150
rs2988279
All
0.0
0.2
0.4
0.6
0.8
MR leave−one−out sensitivity analysis for
' || id:ebi−a−GCST90001839' on 'pNENs'

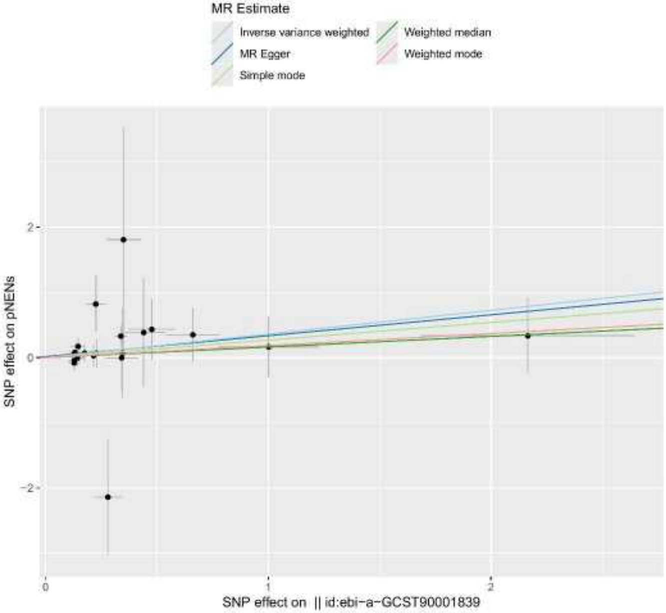
MR Estimate
Inverse variance weighted
MR Egger
Simple mode
Weighted median
Weighted mode
SNP effect on pNENs
2
0
−2
0
1
2
SNP effect on || id:ebi−a−GCST90001839

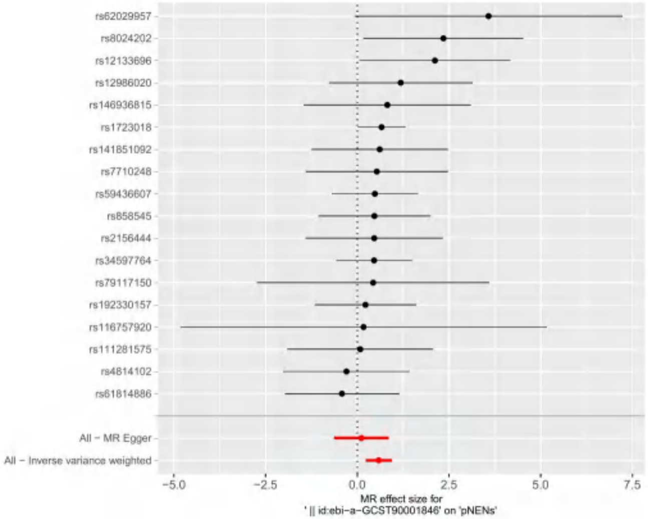
All − MR Egger
All − Inverse variance weighted
MR effect size for
' || id:ebi−a−GCST90001846' on 'pNENs'

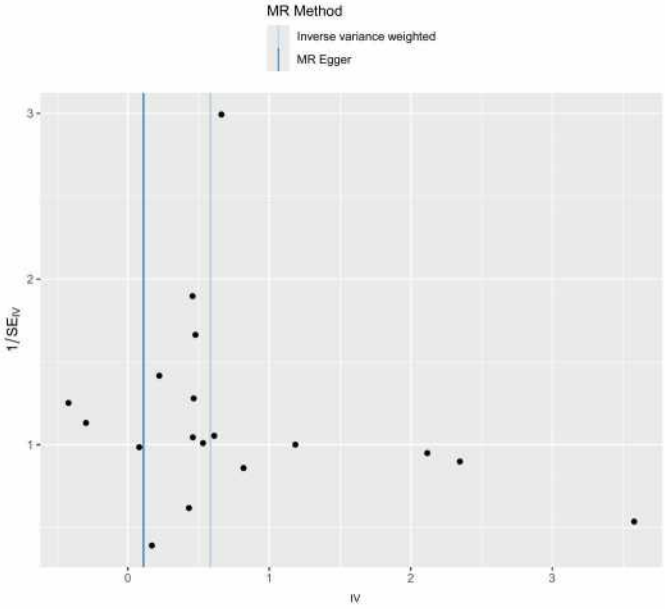
MR Method
Inverse variance weighted
MR Egger
1/SE_IV
IV

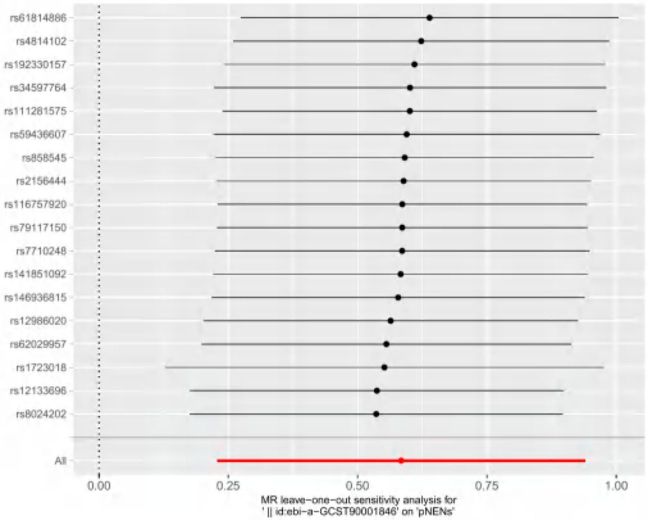
All
MR leave−one−out sensitivity analysis for
' || id:ebi−a−GCST90001846' on 'pNENs'

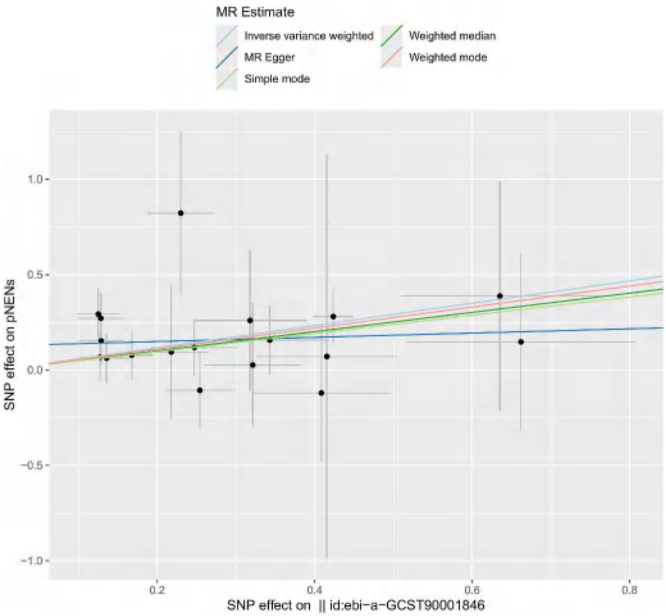
MR Estimate
Inverse variance weighted
MR Egger
Simple mode
Weighted median
Weighted mode
SNP effect on pNENs
SNP effect on || id:ebi−a−GCST90001846

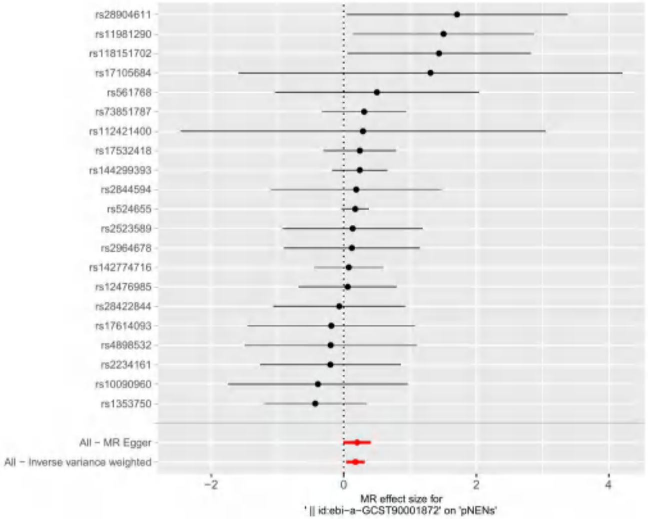

rs28904611
rs11981290
rs118151702
rs17105684
rs561768
rs73851787
rs112421400
rs17532418
rs144299393
rs2844594
rs524655
rs2523589
rs2964678
rs142774716
rs12476985
rs28422844
rs17614093
rs4898532
rs2234161
rs10090960
rs1353750
All − MR Egger
All − Inverse variance weighted
−2
0
2
4
MR effect size for
' || id:ebi−a−GCST90001872' on 'pNENs'

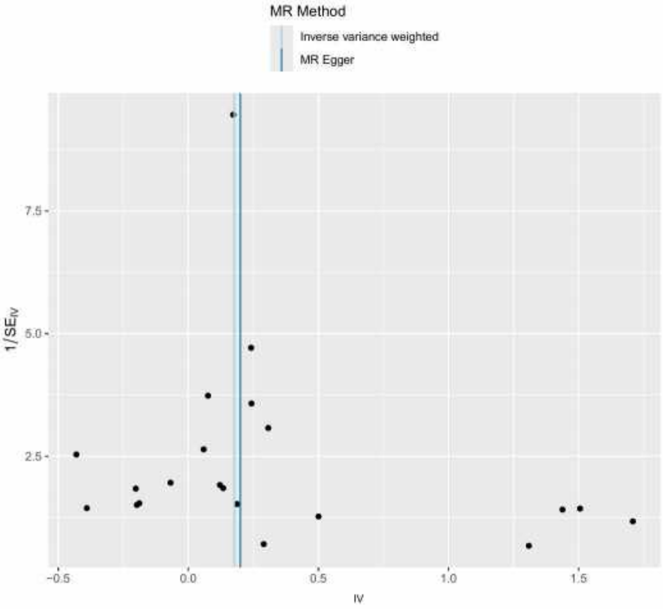

MR Method
Inverse variance weighted
MR Egger
7.5
5.0
2.5
$1/SE_{IV}$
−0.5
0.0
0.5
1.0
1.5
IV

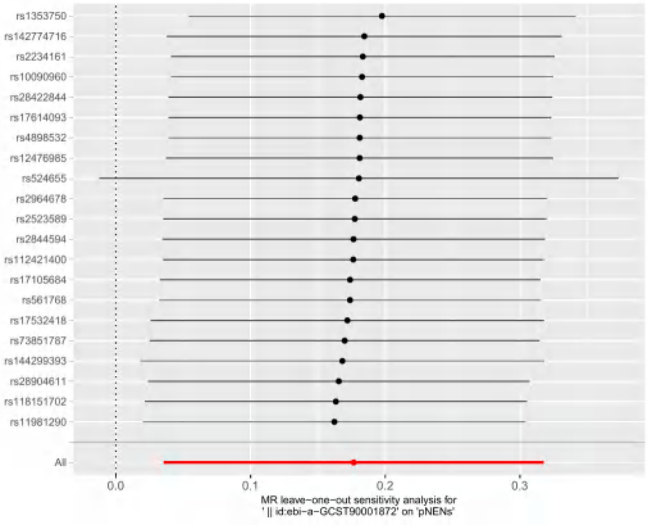

rs1353750
rs142774716
rs2234161
rs10090960
rs28422844
rs17614093
rs4898532
rs12476985
rs524655
rs2964678
rs2523589
rs2844594
rs112421400
rs17105684
rs561768
rs17532418
rs73851787
rs144299393
rs28904611
rs118151702
rs11981290
All
0.0
0.1
0.2
0.3
MR leave−one−out sensitivity analysis for
' || id:ebi−a−GCST90001872' on 'pNENs'

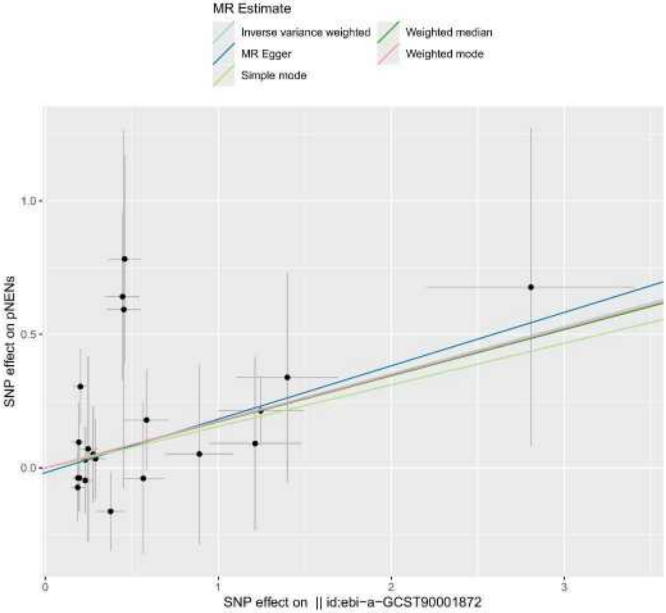

MR Estimate
Inverse variance weighted
MR Egger
Simple mode
Weighted median
Weighted mode
1.0
0.5
0.0
SNP effect on pNENs
0
1
2
3
SNP effect on || id:ebi−a−GCST90001872

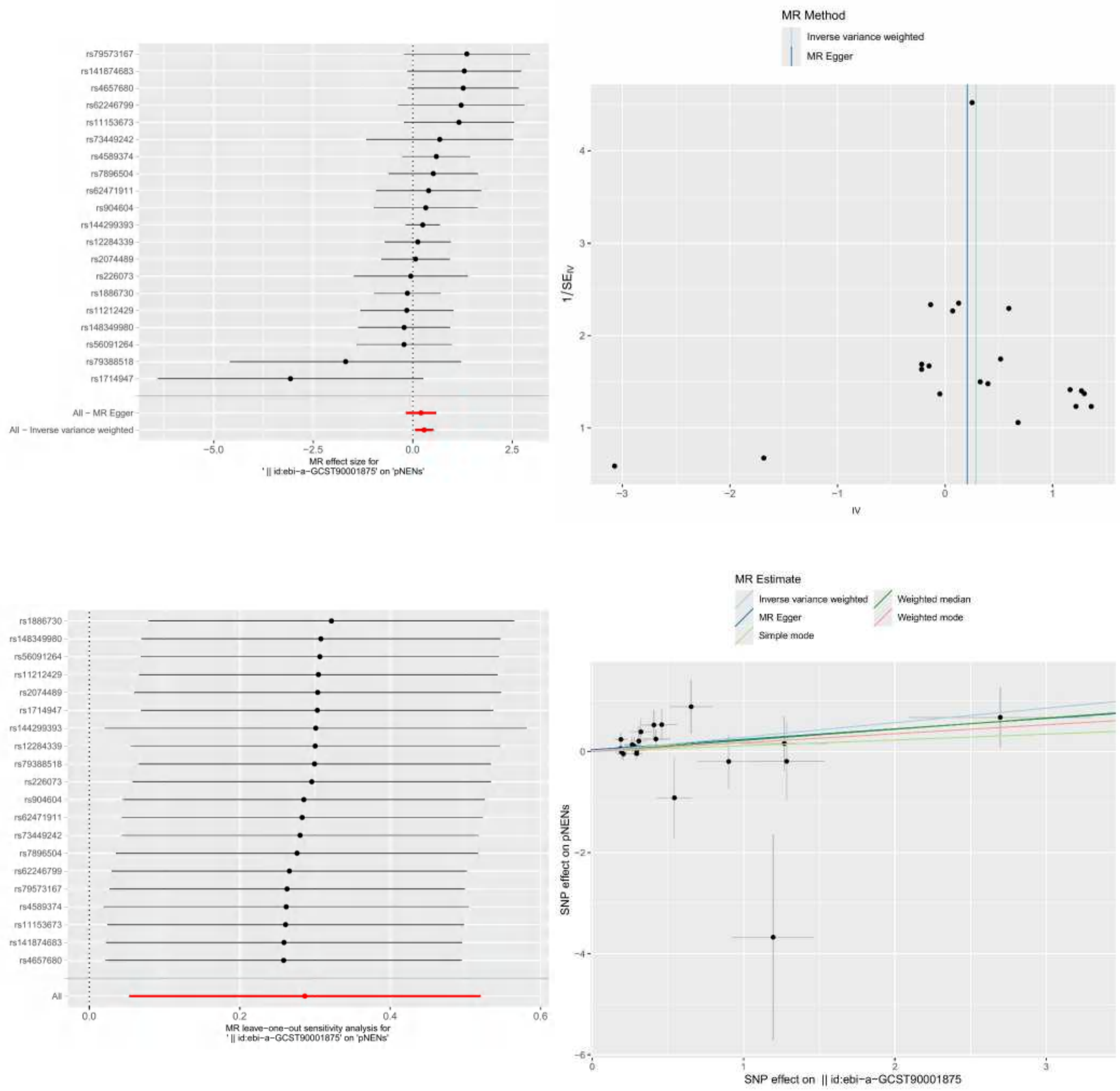

rs79573167
rs141874683
rs4657680
rs62246799
rs11153673
rs73449242
rs4589374
rs7896504
rs62471911
rs904604
rs144299393
rs12284339
rs2074489
rs226073
rs1886730
rs11212429
rs148349980
rs56091264
rs79388518
rs1714947
All − MR Egger
All − Inverse variance weighted
−5.0
−2.5
0.0
2.5
MR effect size for
' || id:ebi−a−GCST90001875' on 'pNENs'
MR Method
Inverse variance weighted
MR Egger
1/SE_IV
IV
rs1886730
rs148349980
rs56091264
rs11212429
rs2074489
rs1714947
rs144299393
rs12284339
rs79388518
rs226073
rs904604
rs62471911
rs73449242
rs7896504
rs62246799
rs79573167
rs4589374
rs11153673
rs141874683
rs4657680
All
0.0
0.2
0.4
0.6
MR leave−one−out sensitivity analysis for
' || id:ebi−a−GCST90001875' on 'pNENs'
MR Estimate
Inverse variance weighted
MR Egger
Simple mode
Weighted median
Weighted mode
SNP effect on pNENs
SNP effect on || id:ebi−a−GCST90001875

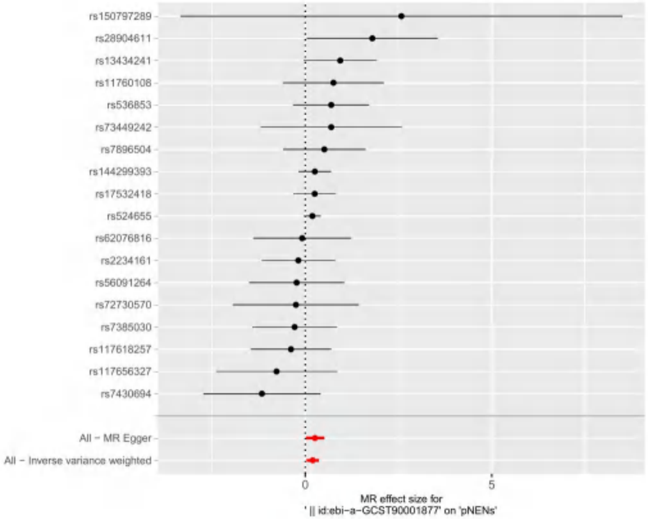
rs150797289
rs28904611
rs13434241
rs11760108
rs536853
rs73449242
rs7896504
rs144299393
rs17532418
rs524655
rs62076816
rs2234161
rs56091264
rs72730570
rs7385030
rs117618257
rs117656327
rs7430694
All − MR Egger
All − Inverse variance weighted
0
5
MR effect size for
' || id:ebi-a-GCST90001877' on 'pNENs'

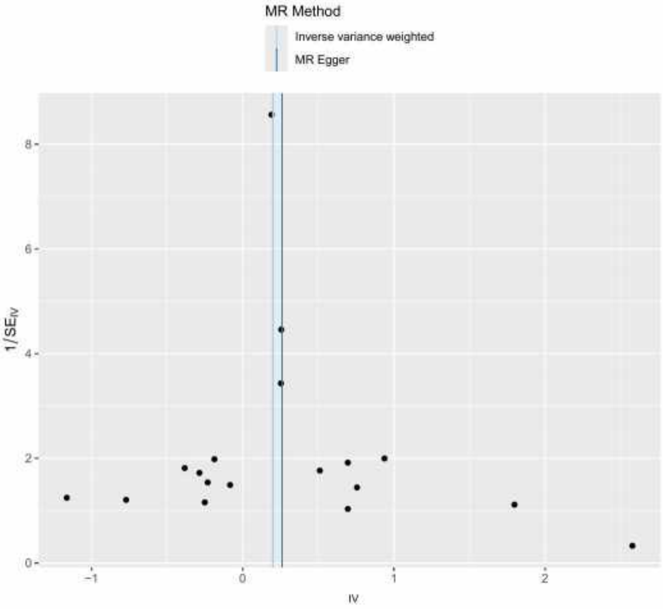
MR Method
Inverse variance weighted
MR Egger
1/SE_IV
0
2
4
6
8
−1
0
1
2
IV

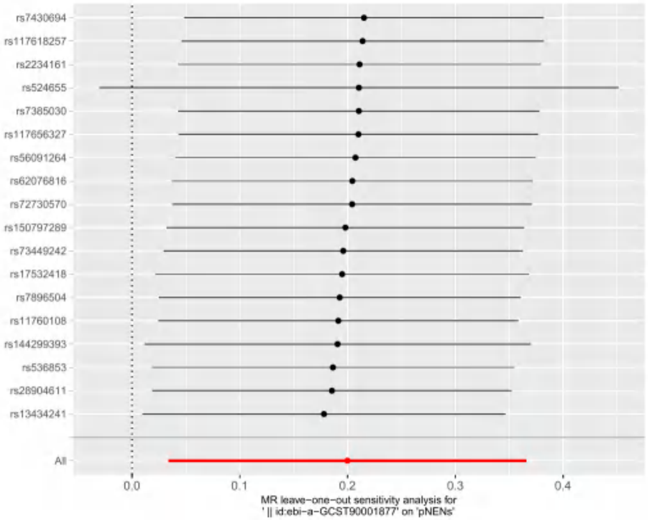
rs7430694
rs117618257
rs2234161
rs524655
rs7385030
rs117656327
rs56091264
rs62076816
rs72730570
rs150797289
rs73449242
rs17532418
rs7896504
rs11760108
rs144299393
rs536853
rs28904611
rs13434241
All
0.0
0.1
0.2
0.3
0.4
MR leave−one−out sensitivity analysis for
' || id:ebi-a-GCST90001877' on 'pNENs'

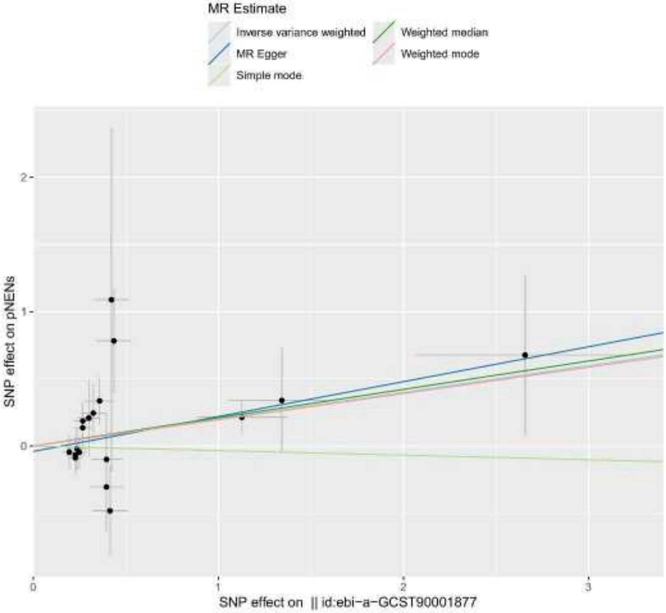
MR Estimate
Inverse variance weighted
MR Egger
Simple mode
Weighted median
Weighted mode
SNP effect on pNENs
0
1
2
0
1
2
3
SNP effect on || id:ebi-a-GCST90001877

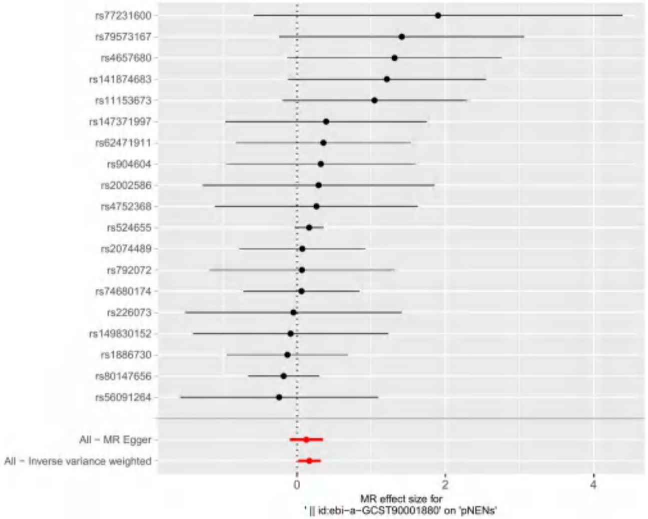
rs77231600
rs79573167
rs4657680
rs141874683
rs11153673
rs147371997
rs62471911
rs904604
rs2002586
rs4752368
rs524655
rs2074489
rs792072
rs74680174
rs226073
rs149830152
rs1886730
rs80147656
rs56091264
All − MR Egger
All − Inverse variance weighted
0
2
4
MR effect size for
' || id:ebi−a−GCST90001880' on 'pNENs'

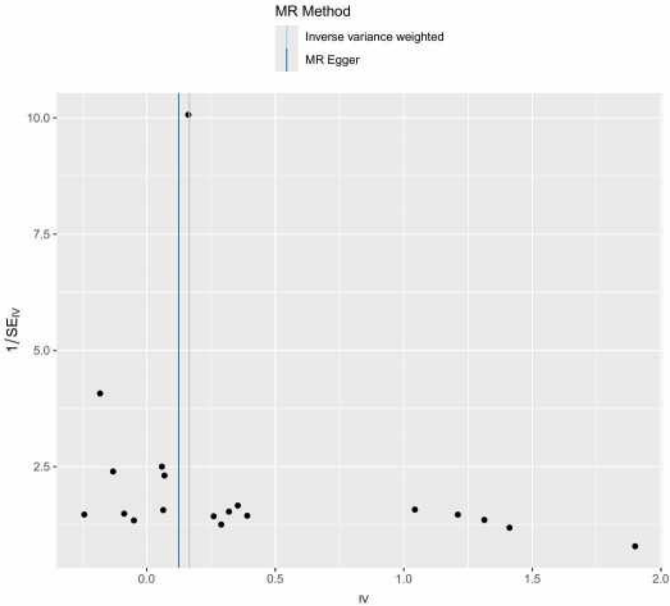
MR Method
Inverse variance weighted
MR Egger
10.0
7.5
5.0
2.5
1/SE$_{IV}$
0.0
0.5
1.0
1.5
2.0
IV

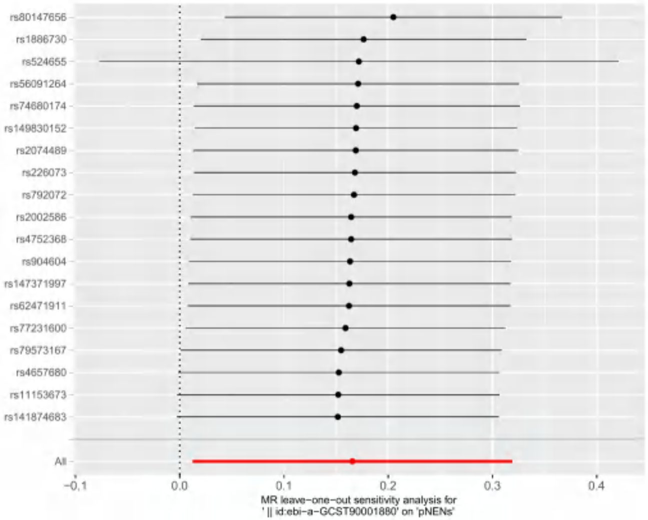
rs80147656
rs1886730
rs524655
rs56091264
rs74680174
rs149830152
rs2074489
rs226073
rs792072
rs2002586
rs4752368
rs904604
rs147371997
rs62471911
rs77231600
rs79573167
rs4657680
rs11153673
rs141874683
All
−0.1
0.0
0.1
0.2
0.3
0.4
MR leave−one−out sensitivity analysis for
' || id:ebi−a−GCST90001880' on 'pNENs'

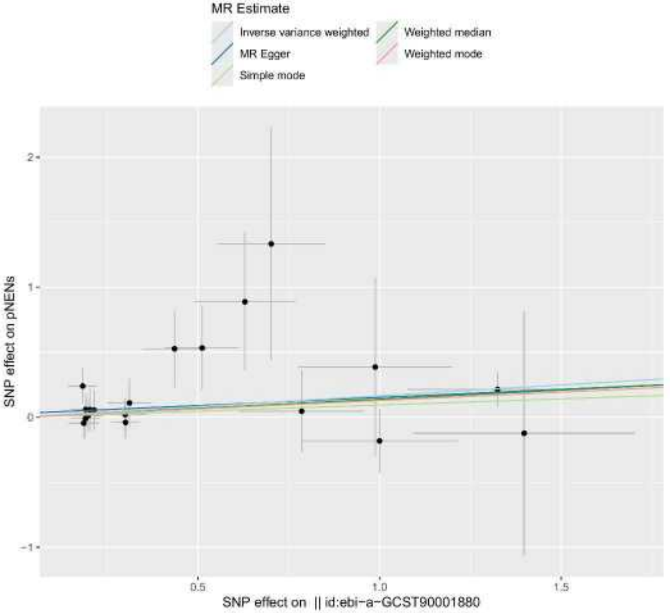
MR Estimate
Inverse variance weighted
MR Egger
Simple mode
Weighted median
Weighted mode
2
1
0
−1
SNP effect on pNENs
0.5
1.0
1.5
SNP effect on || id:ebi−a−GCST90001880

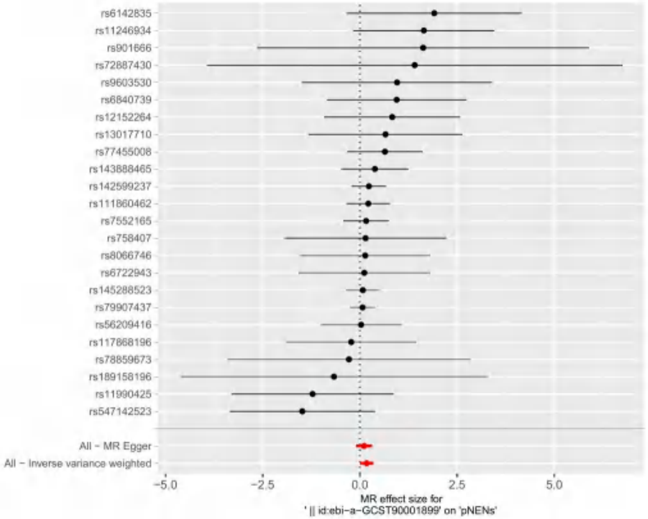
rs6142835
rs11246934
rs901666
rs72887430
rs9603530
rs6840739
rs12152264
rs13017710
rs77455008
rs143888465
rs142599237
rs111860462
rs7552165
rs758407
rs8066746
rs6722943
rs145288523
rs79907437
rs56209416
rs117868196
rs78859673
rs189158196
rs11990425
rs547142523
All − MR Egger
All − Inverse variance weighted
−5.0
−2.5
0.0
2.5
5.0
MR effect size for
' || id:ebi−a−GCST90001899' on 'pNENs'

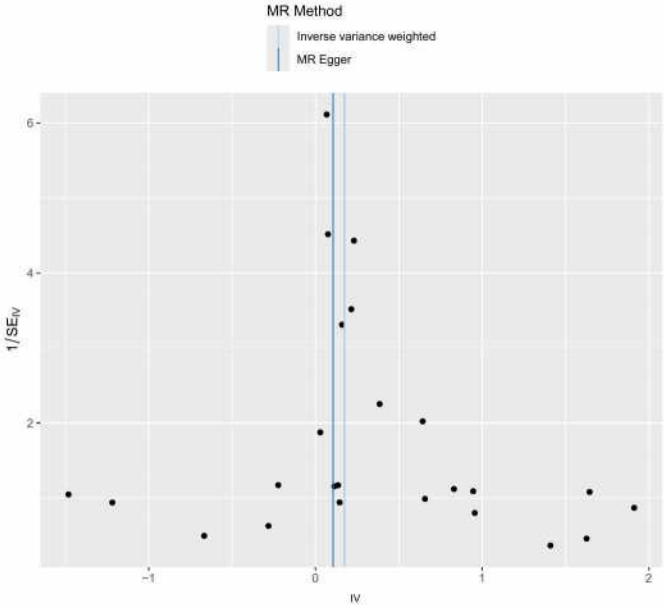
MR Method
Inverse variance weighted
MR Egger
1/SE$_{IV}$
6
4
2
−1
0
1
2
IV

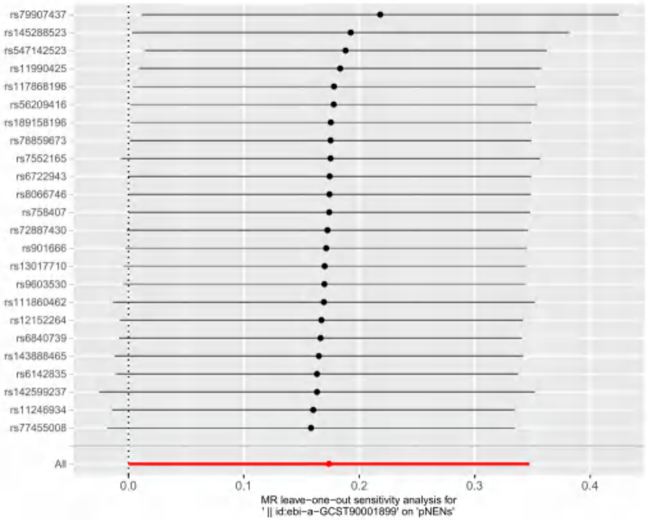
rs79907437
rs145288523
rs547142523
rs11990425
rs117868196
rs56209416
rs189158196
rs78859673
rs7552165
rs6722943
rs8066746
rs758407
rs72887430
rs901666
rs13017710
rs9603530
rs111860462
rs12152264
rs6840739
rs143888465
rs6142835
rs142599237
rs11246934
rs77455008
All
0.0
0.1
0.2
0.3
0.4
MR leave−one−out sensitivity analysis for
' || id:ebi−a−GCST90001899' on 'pNENs'

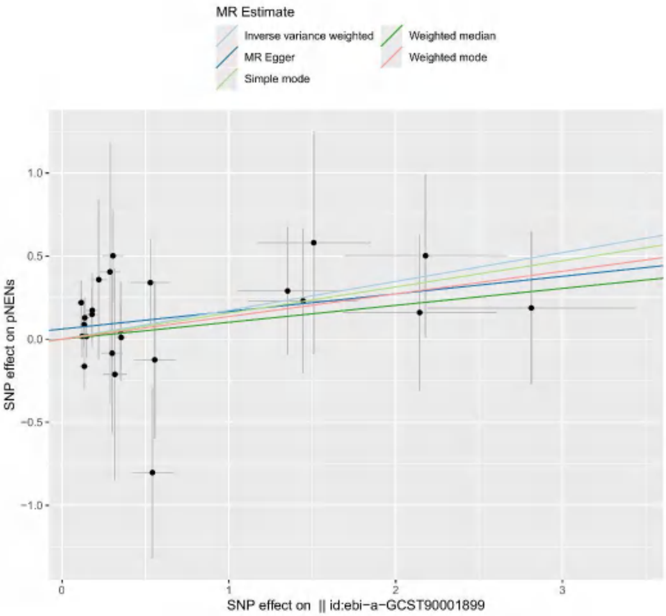
MR Estimate
Inverse variance weighted
MR Egger
Simple mode
Weighted median
Weighted mode
SNP effect on pNENs
1.0
0.5
0.0
−0.5
−1.0
0
1
2
3
SNP effect on || id:ebi−a−GCST90001899

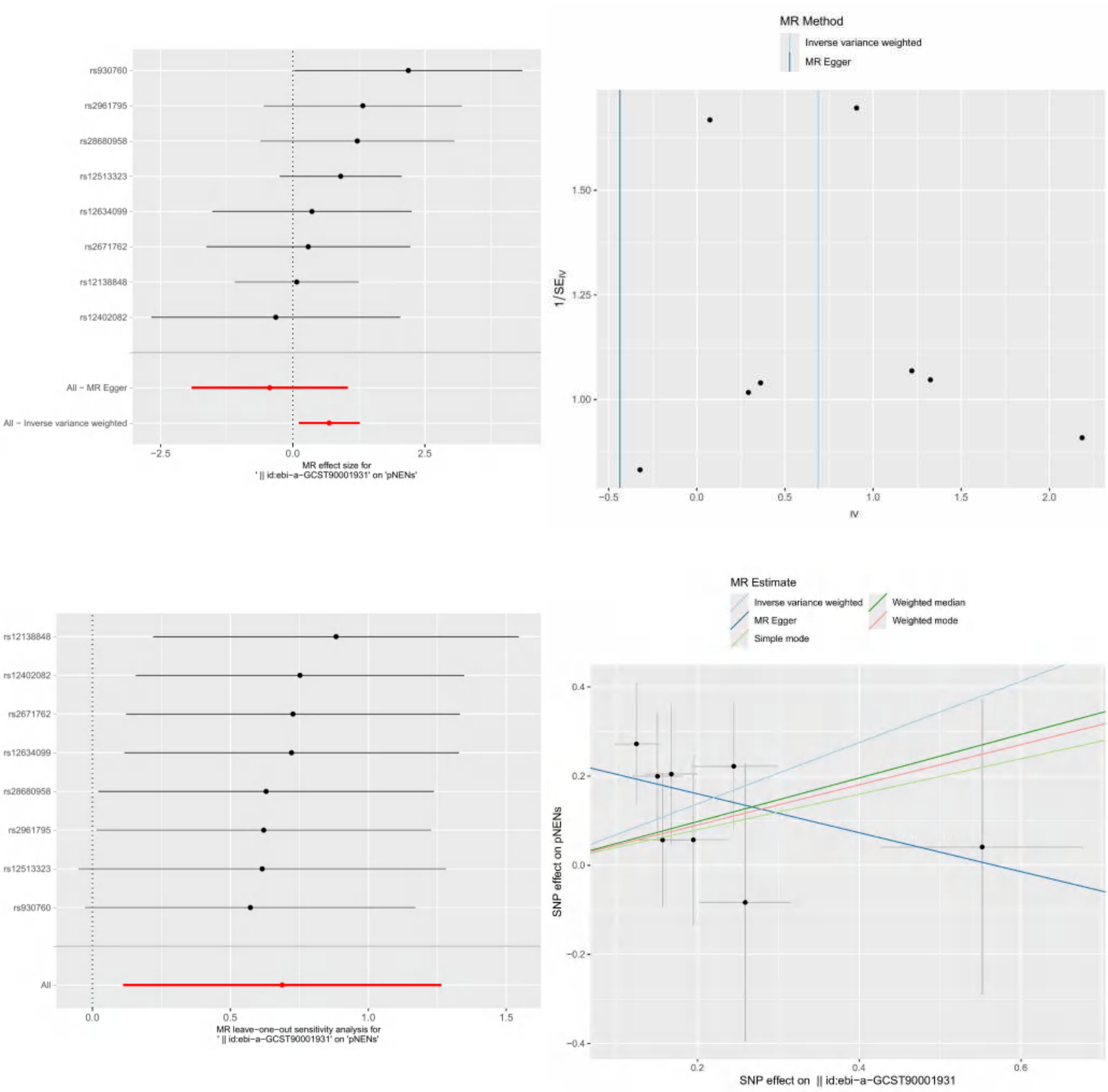

rs930760
rs2961795
rs28680958
rs12513323
rs12634099
rs2671762
rs12138848
rs12402082
All − MR Egger
All − Inverse variance weighted
-2.5
0.0
2.5
MR effect size for
' || id:ebi−a−GCST90001931' on 'pNENs'
MR Method
Inverse variance weighted
MR Egger
1.50
1.25
1.00
1/SE_IV
-0.5
0.0
0.5
1.0
1.5
2.0
IV
rs12138848
rs12402082
rs2671762
rs12634099
rs28680958
rs2961795
rs12513323
rs930760
All
0.0
0.5
1.0
1.5
MR leave−one−out sensitivity analysis for
' || id:ebi−a−GCST90001931' on 'pNENs'
MR Estimate
Inverse variance weighted
MR Egger
Simple mode
Weighted median
Weighted mode
0.4
0.2
0.0
-0.2
-0.4
SNP effect on pNENs
0.2
0.4
0.6
SNP effect on || id:ebi−a−GCST90001931

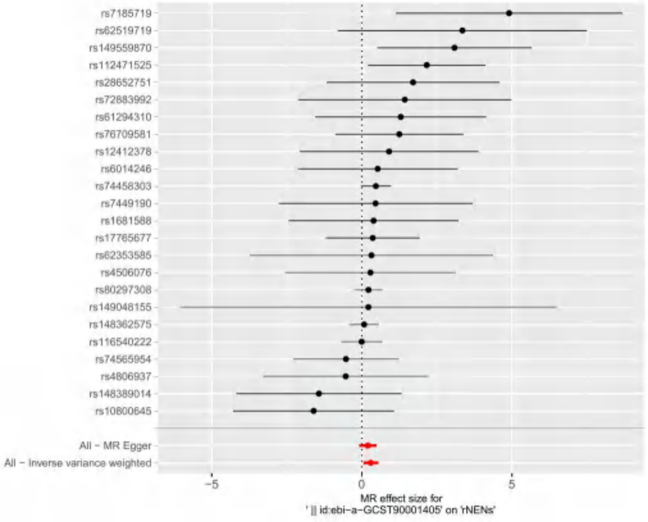
rs7185719
rs62519719
rs149559870
rs112471525
rs28652751
rs72883992
rs61294310
rs76709581
rs12412378
rs6014246
rs74458303
rs7449190
rs1681588
rs17765677
rs62353585
rs4506076
rs80297308
rs149048155
rs148362575
rs116540222
rs74565954
rs4806937
rs148389014
rs10800645
All − MR Egger
All − Inverse variance weighted
−5
0
5
MR effect size for
' || id:ebi−a−GCST90001405' on 'rNENs'

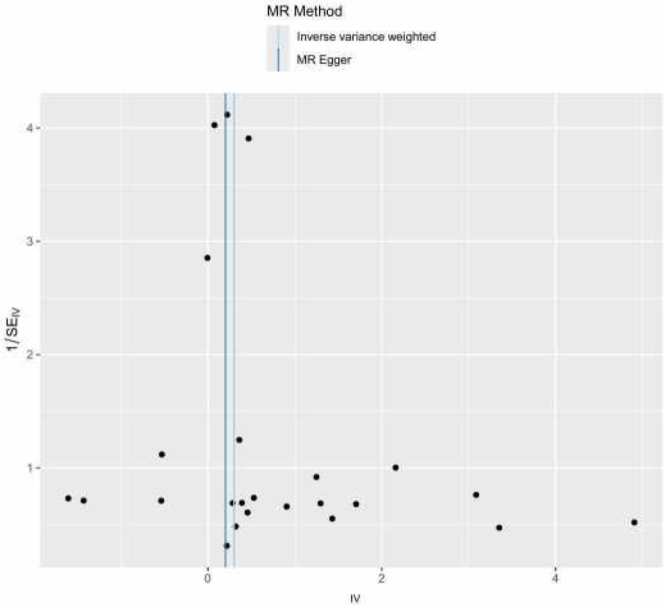
MR Method
Inverse variance weighted
MR Egger
1/SE$_{IV}$
IV
0
2
4
1
2
3
4

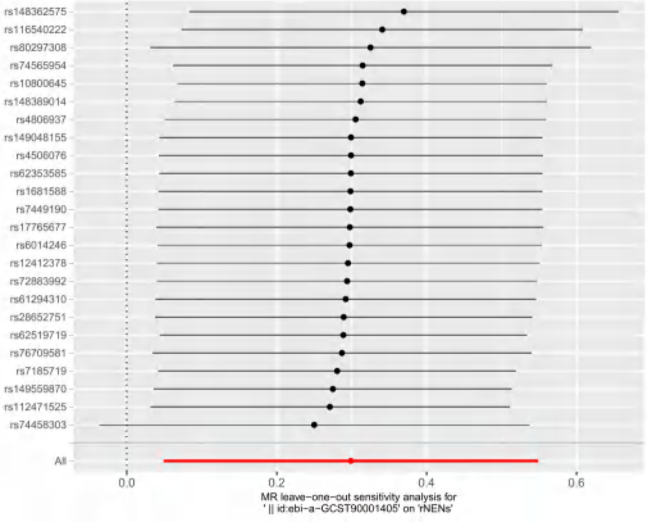
rs148362575
rs116540222
rs80297308
rs74565954
rs10800645
rs148389014
rs4806937
rs149048155
rs4506076
rs62353585
rs1681588
rs7449190
rs17765677
rs6014246
rs12412378
rs72883992
rs61294310
rs28652751
rs62519719
rs76709581
rs7185719
rs149559870
rs112471525
rs74458303
All
0.0
0.2
0.4
0.6
MR leave−one−out sensitivity analysis for
' || id:ebi−a−GCST90001405' on 'rNENs'

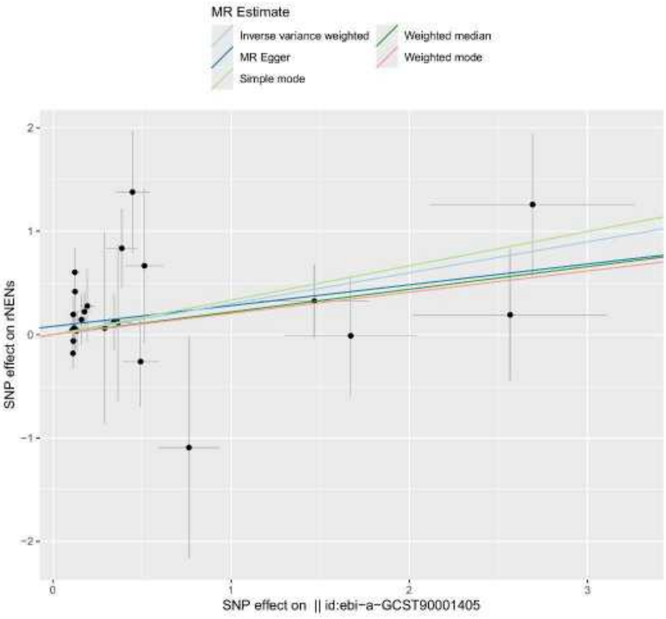
MR Estimate
Inverse variance weighted
MR Egger
Simple mode
Weighted median
Weighted mode
SNP effect on rNENs
SNP effect on || id:ebi−a−GCST90001405
−2
−1
0
1
2
0
1
2
3

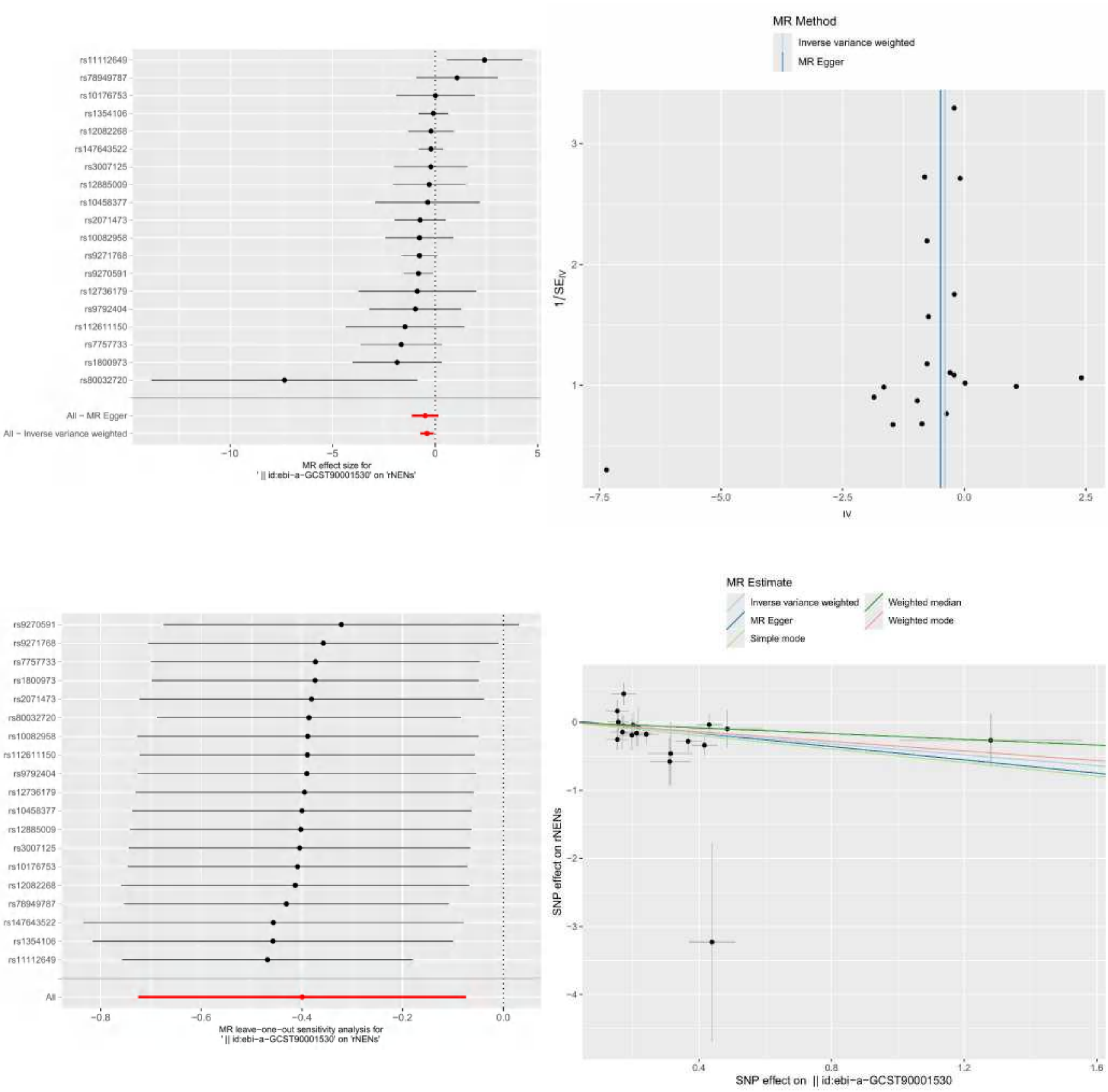
rs11112649
rs78949787
rs10176753
rs1354106
rs12082268
rs147643522
rs3007125
rs12885009
rs10458377
rs2071473
rs10082958
rs9271768
rs9270591
rs12736179
rs9792404
rs112611150
rs7757733
rs1800973
rs80032720
All − MR Egger
All − Inverse variance weighted
−10
−5
0
5
MR effect size for
' || id:ebi−a−GCST90001530' on 'rNENs'
MR Method
Inverse variance weighted
MR Egger
1/SE_IV
3
2
1
−7.5
−5.0
−2.5
0.0
2.5
IV
rs9270591
rs9271768
rs7757733
rs1800973
rs2071473
rs80032720
rs10082958
rs112611150
rs9792404
rs12736179
rs10458377
rs12885009
rs3007125
rs10176753
rs12082268
rs78949787
rs147643522
rs1354106
rs11112649
All
−0.8
−0.6
−0.4
−0.2
0.0
MR leave−one−out sensitivity analysis for
' || id:ebi−a−GCST90001530' on 'rNENs'
MR Estimate
Inverse variance weighted
MR Egger
Simple mode
Weighted median
Weighted mode
0
−1
−2
−3
−4
SNP effect on rNENs
0.4
0.8
1.2
1.6
SNP effect on || id:ebi−a−GCST90001530

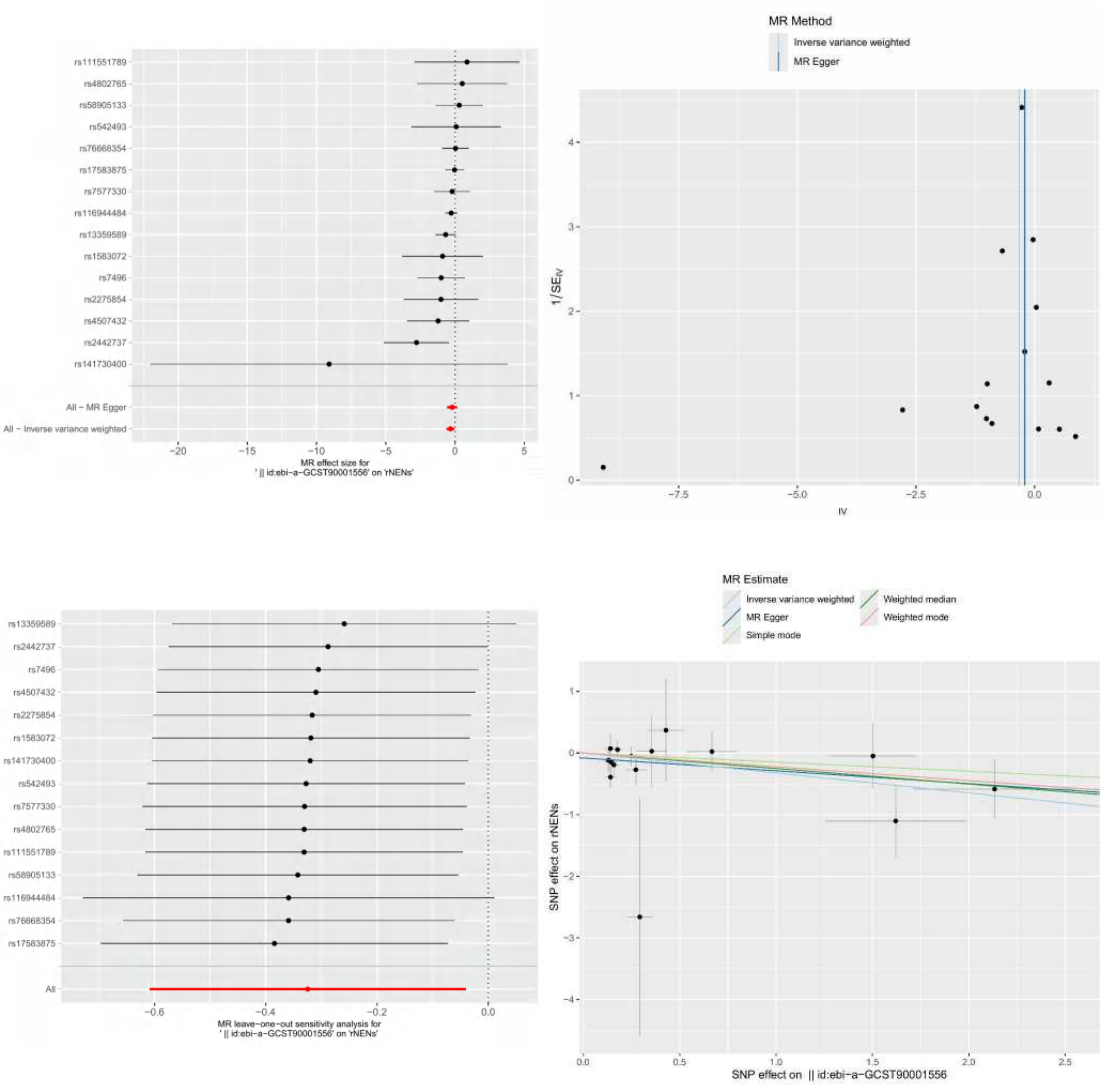
rs111551789
rs4802765
rs58905133
rs542493
rs76668354
rs17583875
rs7577330
rs116944484
rs13359589
rs1583072
rs7496
rs2275854
rs4507432
rs2442737
rs141730400
All − MR Egger
All − Inverse variance weighted
−20
−15
−10
−5
0
5
MR effect size for
' || id:ebi−a−GCST90001556' on 'rNENs'
MR Method
Inverse variance weighted
MR Egger
1/SE$_{IV}$
IV
−7.5
−5.0
−2.5
0.0
rs13359589
rs2442737
rs7496
rs4507432
rs2275854
rs1583072
rs141730400
rs542493
rs7577330
rs4802765
rs111551789
rs58905133
rs116944484
rs76668354
rs17583875
All
−0.6
−0.4
−0.2
0.0
MR leave−one−out sensitivity analysis for
' || id:ebi−a−GCST90001556' on 'rNENs'
MR Estimate
Inverse variance weighted
MR Egger
Simple mode
Weighted median
Weighted mode
SNP effect on rNENs
SNP effect on || id:ebi−a−GCST90001556
0.0
0.5
1.0
1.5
2.0
2.5

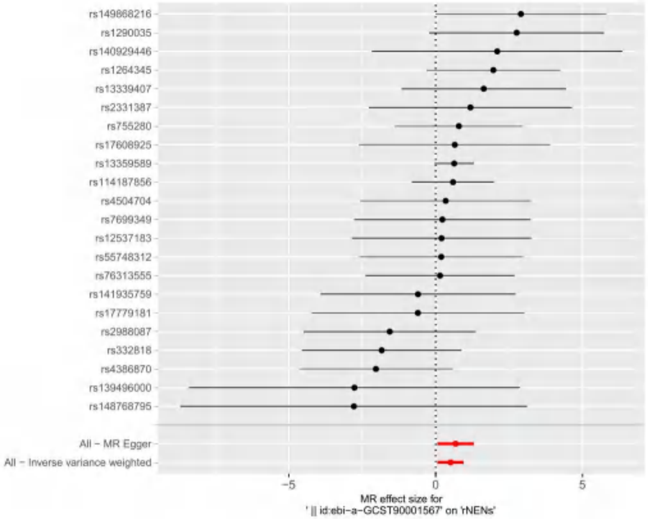
rs149868216
rs1290035
rs140929446
rs1264345
rs13339407
rs2331387
rs755280
rs17608925
rs13359589
rs114187856
rs4504704
rs7699349
rs12537183
rs55748312
rs76313555
rs141935759
rs17779181
rs2988087
rs332818
rs4386870
rs139496000
rs148768795
All − MR Egger
All − Inverse variance weighted
−5
0
5
MR effect size for
' || id:ebi−a−GCST90001567' on 'rNENs'
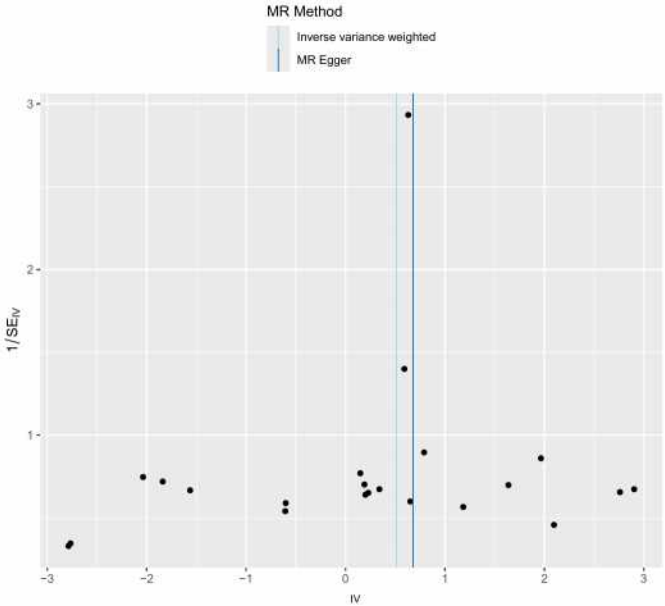
MR Method
Inverse variance weighted
MR Egger
$1/SE_{IV}$
IV
3
2
1
−3
−2
−1
0
1
2
3
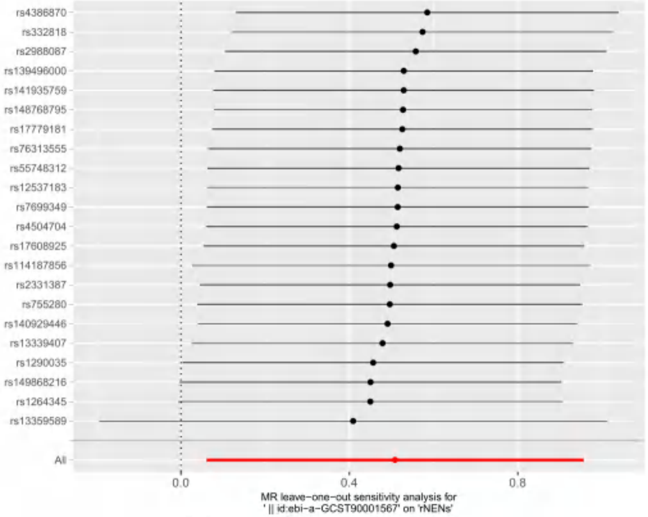
rs4386870
rs332818
rs2988087
rs139496000
rs141935759
rs148768795
rs17779181
rs76313555
rs55748312
rs12537183
rs7699349
rs4504704
rs17608925
rs114187856
rs2331387
rs755280
rs140929446
rs13339407
rs1290035
rs149868216
rs1264345
rs13359589
All
0.0
0.4
0.8
MR leave−one−out sensitivity analysis for
' || id:ebi−a−GCST90001567' on 'rNENs'
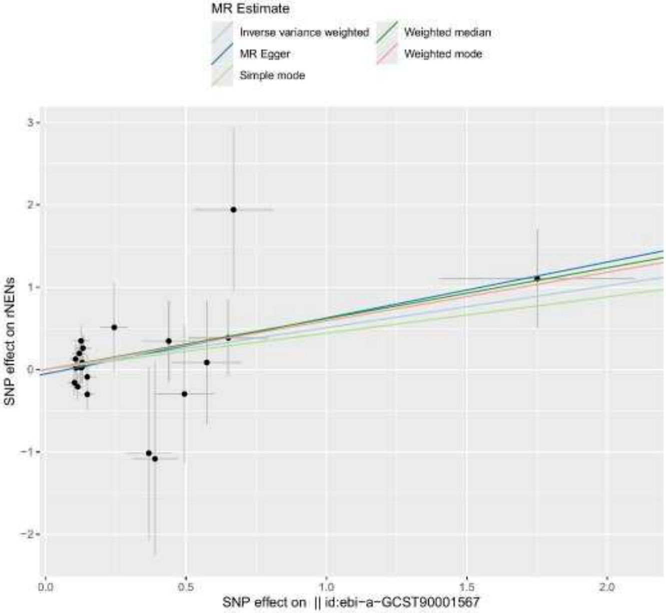
MR Estimate
Inverse variance weighted
MR Egger
Simple mode
Weighted median
Weighted mode
SNP effect on rNENs
SNP effect on || id:ebi−a−GCST90001567
3
2
1
0
−1
−2
0.0
0.5
1.0
1.5
2.0

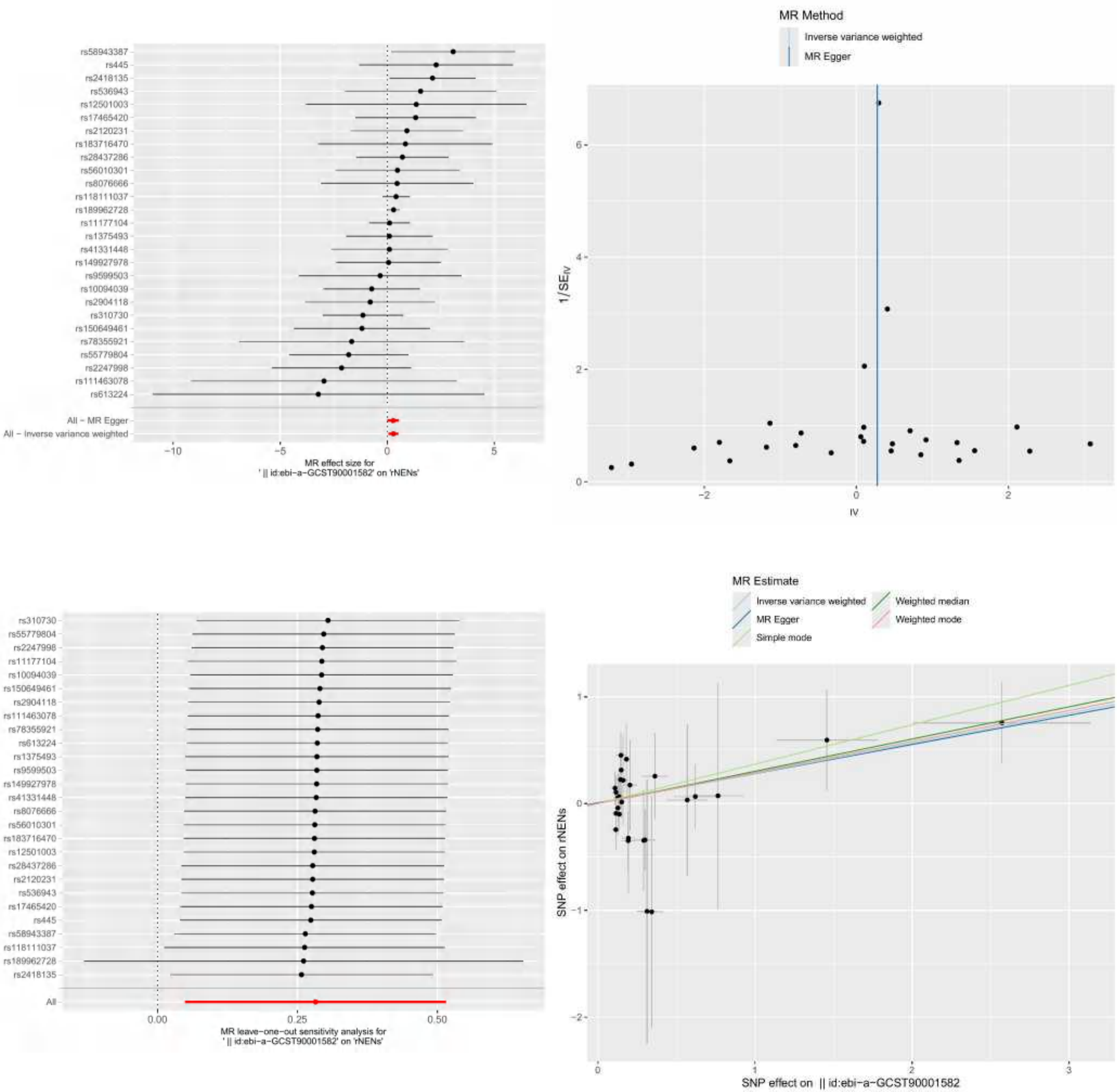
rs58943387
rs445
rs2418135
rs536943
rs12501003
rs17465420
rs2120231
rs183716470
rs28437286
rs56010301
rs8076666
rs118111037
rs189962728
rs11177104
rs1375493
rs41331448
rs149927978
rs9599503
rs10094039
rs2904118
rs310730
rs150649461
rs78355921
rs55779804
rs2247998
rs111463078
rs613224
All − MR Egger
All − Inverse variance weighted
−10
−5
0
5
MR effect size for
' || id:ebi−a−GCST90001582' on 'rNENs'
MR Method
Inverse variance weighted
MR Egger
1/SE$_{IV}$
0
2
4
6
−2
0
2
IV
rs310730
rs55779804
rs2247998
rs11177104
rs10094039
rs150649461
rs2904118
rs111463078
rs78355921
rs613224
rs1375493
rs9599503
rs149927978
rs41331448
rs8076666
rs56010301
rs183716470
rs12501003
rs28437286
rs2120231
rs536943
rs17465420
rs445
rs58943387
rs118111037
rs189962728
rs2418135
All
0.00
0.25
0.50
MR leave−one−out sensitivity analysis for
' || id:ebi−a−GCST90001582' on 'rNENs'
MR Estimate
Inverse variance weighted
MR Egger
Simple mode
Weighted median
Weighted mode
SNP effect on rNENs
1
0
−1
−2
0
1
2
3
SNP effect on || id:ebi−a−GCST90001582

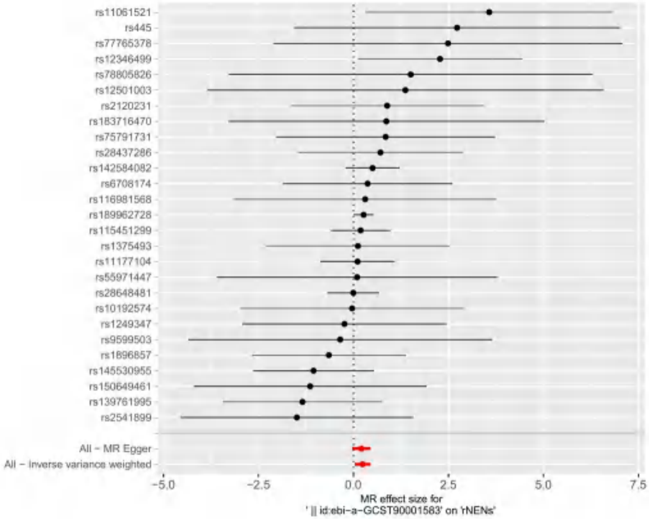
rs11061521
rs445
rs77765378
rs12346499
rs78805826
rs12501003
rs2120231
rs183716470
rs75791731
rs28437286
rs142584082
rs6708174
rs116981568
rs189962728
rs115451299
rs1375493
rs11177104
rs55971447
rs28648481
rs10192574
rs1249347
rs9599503
rs1896857
rs145530955
rs150649461
rs139761995
rs2541899
All − MR Egger
All − Inverse variance weighted
−5.0
−2.5
0.0
2.5
5.0
7.5
MR effect size for
' || id:ebi−a−GCST90001583' on 'rNENs'

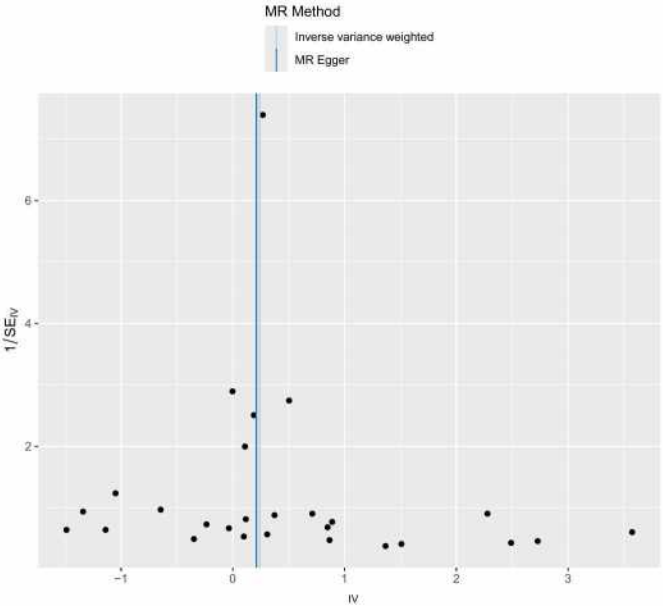
MR Method
Inverse variance weighted
MR Egger
1/SE$_{IV}$
6
4
2
−1
0
1
2
3
IV

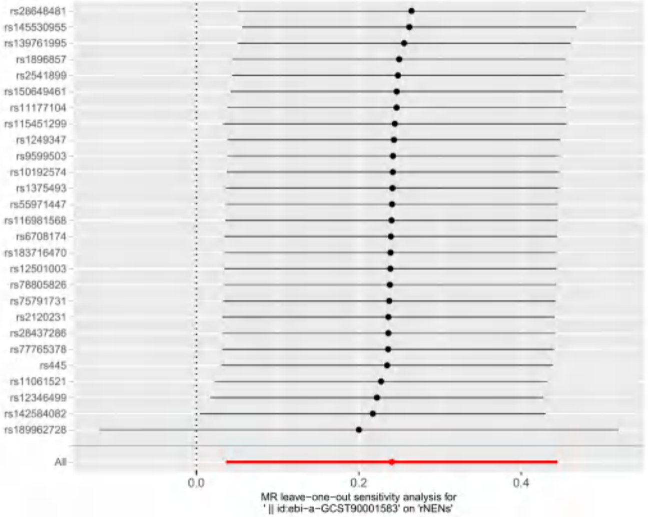
rs28648481
rs145530955
rs139761995
rs1896857
rs2541899
rs150649461
rs11177104
rs115451299
rs1249347
rs9599503
rs10192574
rs1375493
rs55971447
rs116981568
rs6708174
rs183716470
rs12501003
rs78805826
rs75791731
rs2120231
rs28437286
rs77765378
rs445
rs11061521
rs12346499
rs142584082
rs189962728
All
0.0
0.2
0.4
MR leave−one−out sensitivity analysis for
' || id:ebi−a−GCST90001583' on 'rNENs'

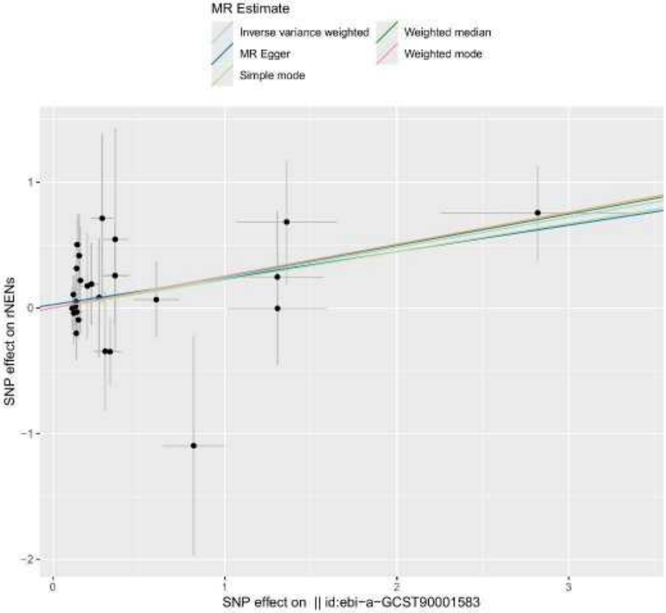
MR Estimate
Inverse variance weighted
MR Egger
Simple mode
Weighted median
Weighted mode
1
0
−1
−2
SNP effect on rNENs
0
1
2
3
SNP effect on || id:ebi−a−GCST90001583

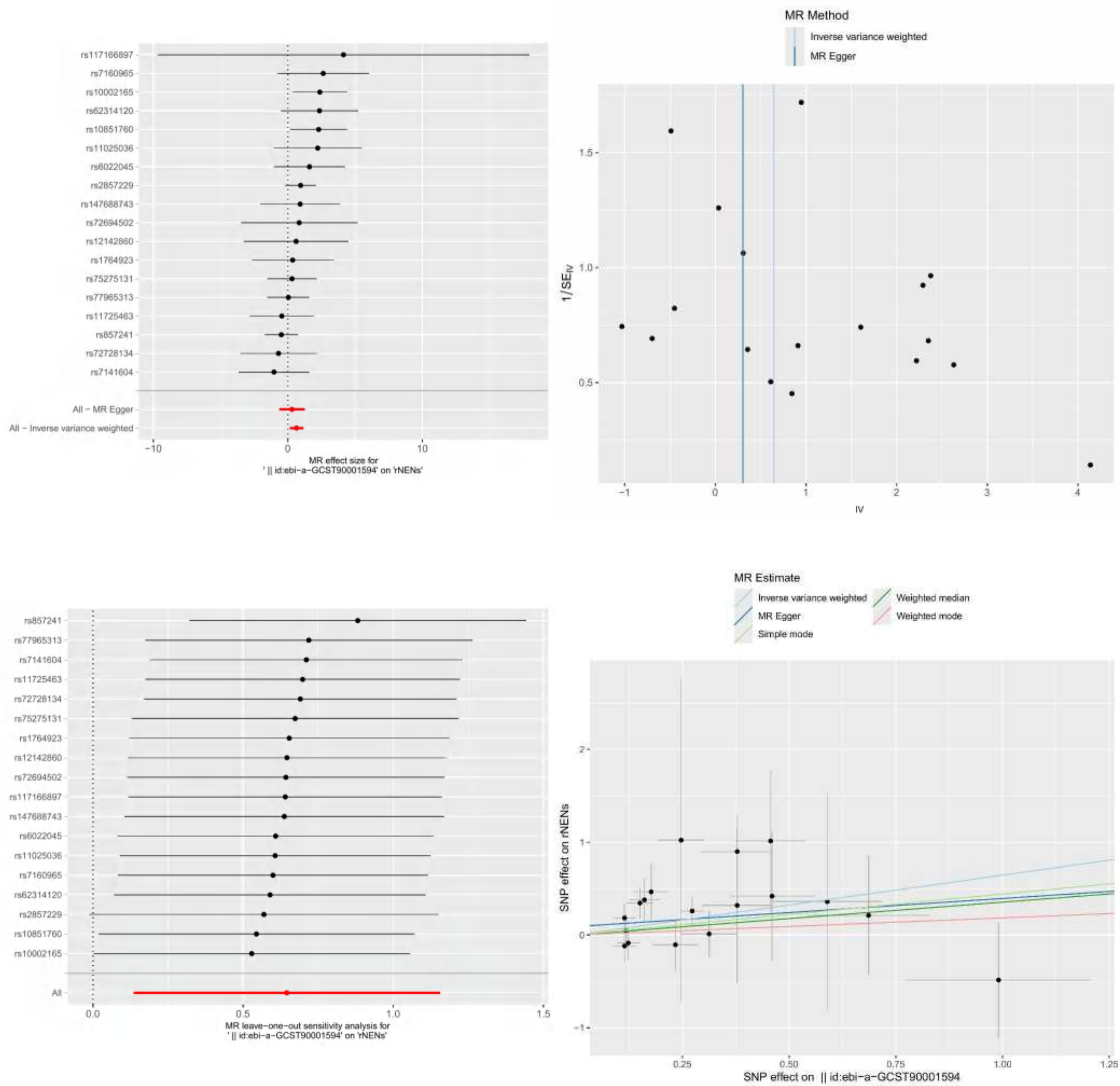
rs117166897
rs7160965
rs10002165
rs62314120
rs10851760
rs11025036
rs6022045
rs2857229
rs147688743
rs72694502
rs12142860
rs1764923
rs75275131
rs77965313
rs11725463
rs857241
rs72728134
rs7141604
All − MR Egger
All − Inverse variance weighted
-10
0
10
MR effect size for
' || id:ebi−a−GCST90001594' on 'rNENs'
MR Method
Inverse variance weighted
MR Egger
1/SE_IV
1.5
1.0
0.5
-1
0
1
2
3
4
IV
rs857241
rs77965313
rs7141604
rs11725463
rs72728134
rs75275131
rs1764923
rs12142860
rs72694502
rs117166897
rs147688743
rs6022045
rs11025036
rs7160965
rs62314120
rs2857229
rs10851760
rs10002165
All
0.0
0.5
1.0
1.5
MR leave−one−out sensitivity analysis for
' || id:ebi−a−GCST90001594' on 'rNENs'
MR Estimate
Inverse variance weighted
MR Egger
Simple mode
Weighted median
Weighted mode
SNP effect on rNENs
2
1
0
-1
0.25
0.50
0.75
1.00
1.25
SNP effect on || id:ebi−a−GCST90001594

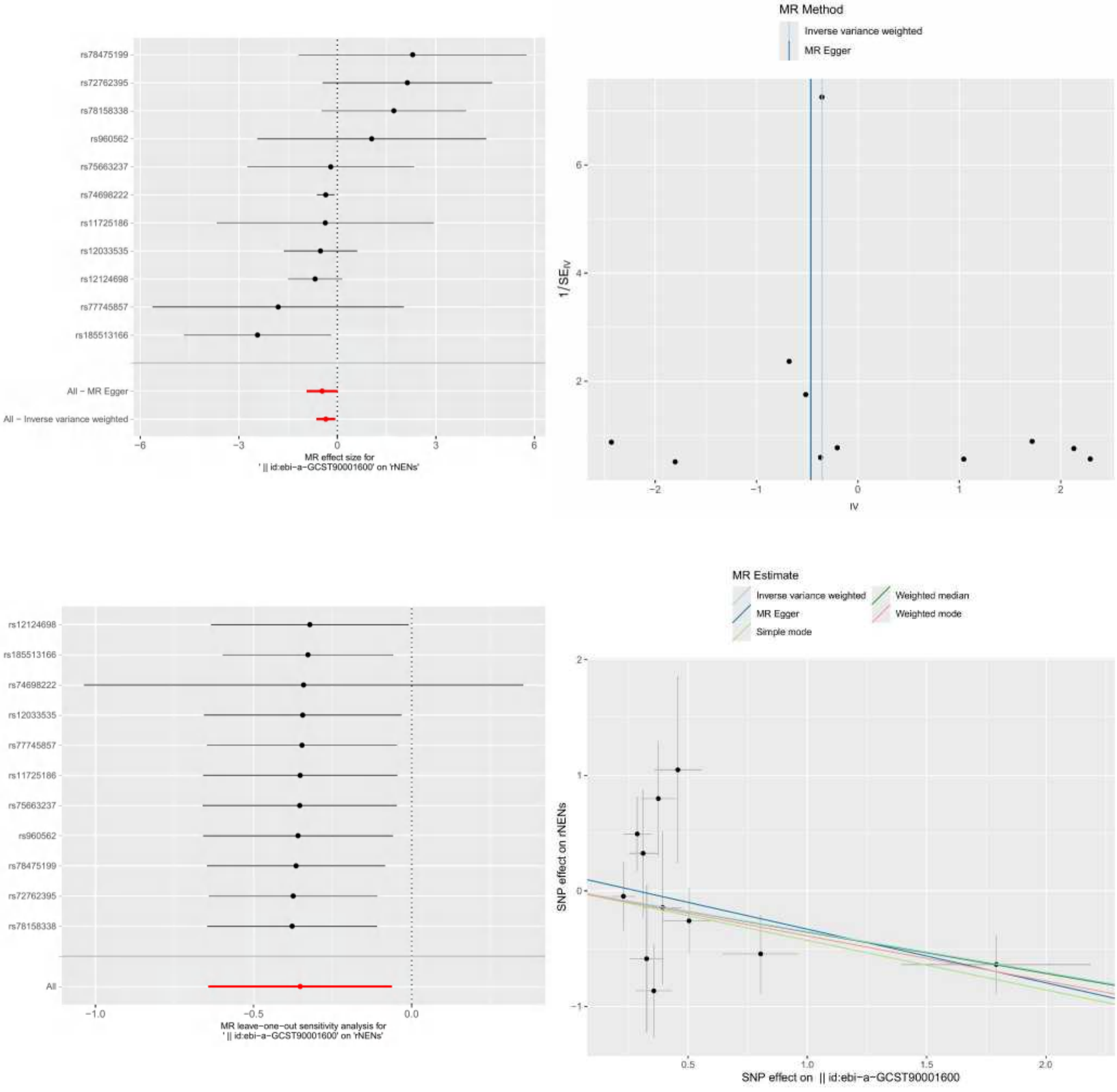

rs78475199
rs72762395
rs78158338
rs960562
rs75663237
rs74698222
rs11725186
rs12033535
rs12124698
rs77745857
rs185513166
All − MR Egger
All − Inverse variance weighted
−6
−3
0
3
6
MR effect size for
' || id:ebi−a−GCST90001600' on 'rNENs'
MR Method
Inverse variance weighted
MR Egger
1/SE_IV
6
4
2
−2
−1
0
1
2
IV
rs12124698
rs185513166
rs74698222
rs12033535
rs77745857
rs11725186
rs75663237
rs960562
rs78475199
rs72762395
rs78158338
All
−1.0
−0.5
0.0
MR leave−one−out sensitivity analysis for
' || id:ebi−a−GCST90001600' on 'rNENs'
MR Estimate
Inverse variance weighted
MR Egger
Simple mode
Weighted median
Weighted mode
SNP effect on rNENs
2
1
0
−1
0.5
1.0
1.5
2.0
SNP effect on || id:ebi−a−GCST90001600

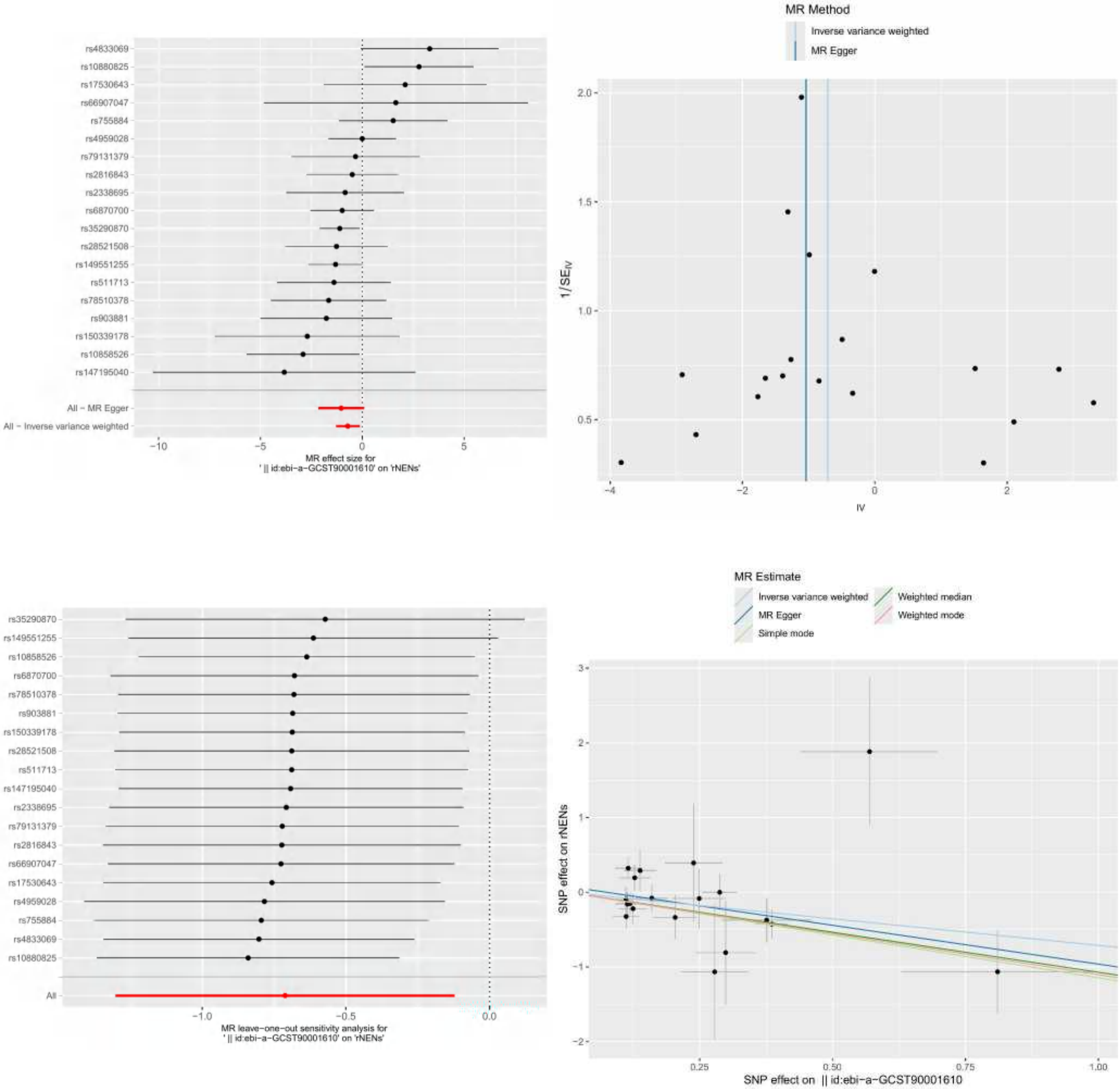
rs4833069
rs10880825
rs17530643
rs66907047
rs755884
rs4959028
rs79131379
rs2816843
rs2338695
rs6870700
rs35290870
rs28521508
rs149551255
rs511713
rs78510378
rs903881
rs150339178
rs10858526
rs147195040
All − MR Egger
All − Inverse variance weighted
−10
−5
0
5
MR effect size for
' || id:ebi−a−GCST90001610' on 'rNENs'
MR Method
Inverse variance weighted
MR Egger
2.0
1.5
1.0
0.5
1/SE$_{IV}$
−4
−2
0
2
IV
rs35290870
rs149551255
rs10858526
rs6870700
rs78510378
rs903881
rs150339178
rs28521508
rs511713
rs147195040
rs2338695
rs79131379
rs2816843
rs66907047
rs17530643
rs4959028
rs755884
rs4833069
rs10880825
All
−1.0
−0.5
0.0
MR leave−one−out sensitivity analysis for
' || id:ebi−a−GCST90001610' on 'rNENs'
MR Estimate
Inverse variance weighted
MR Egger
Simple mode
Weighted median
Weighted mode
3
2
1
0
−1
−2
SNP effect on rNENs
0.25
0.50
0.75
1.00
SNP effect on || id:ebi−a−GCST90001610

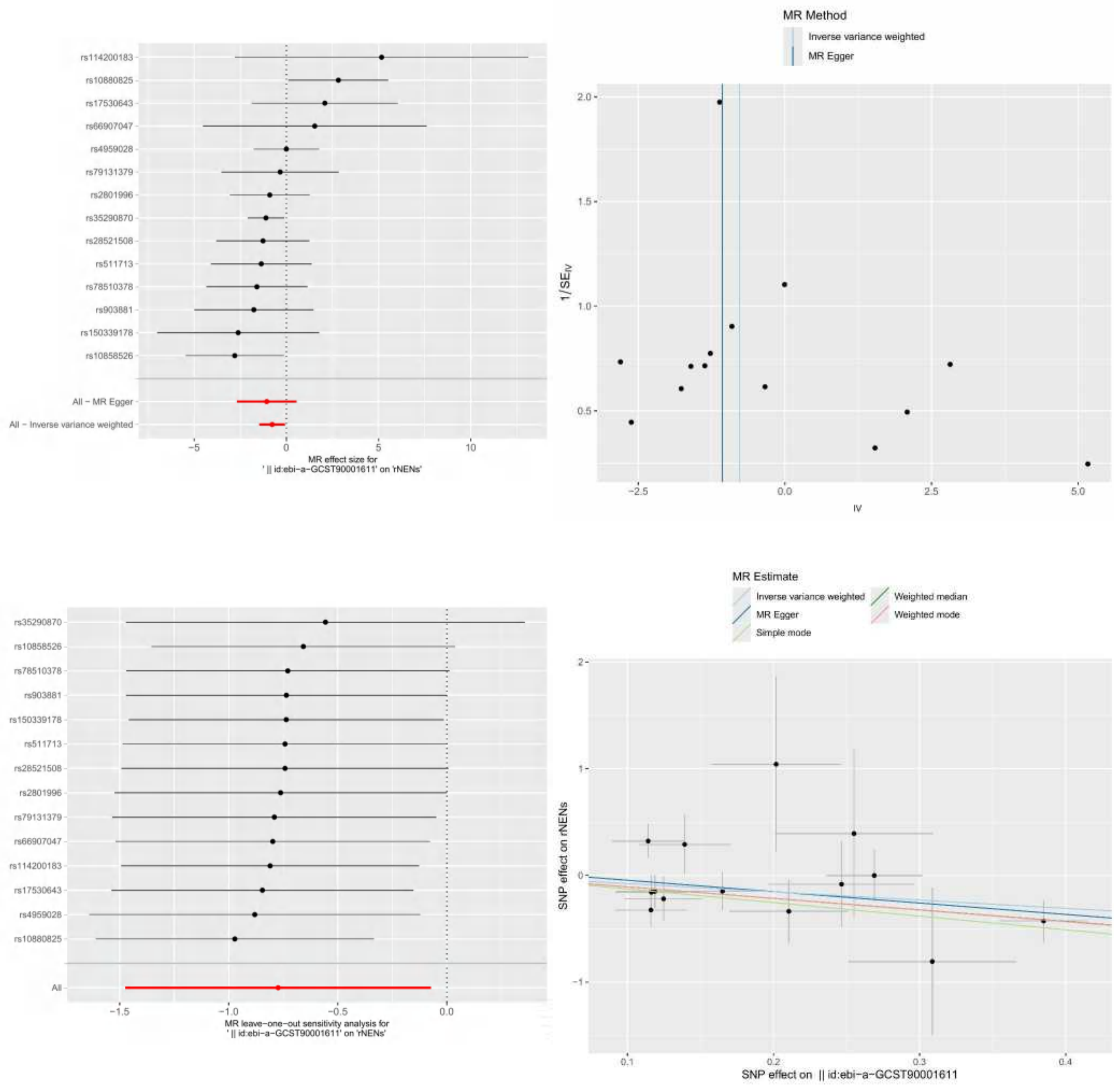

rs114200183
rs10880825
rs17530643
rs66907047
rs4959028
rs79131379
rs2801996
rs35290870
rs28521508
rs511713
rs78510378
rs903881
rs150339178
rs10858526
All − MR Egger
All − Inverse variance weighted
MR effect size for
' || id:ebi−a−GCST90001611' on 'rNENs'
MR Method
Inverse variance weighted
MR Egger
1/SE_{IV}
IV
All
MR leave−one−out sensitivity analysis for
' || id:ebi−a−GCST90001611' on 'rNENs'
MR Estimate
Inverse variance weighted
MR Egger
Simple mode
Weighted median
Weighted mode
SNP effect on rNENs
SNP effect on || id:ebi−a−GCST90001611

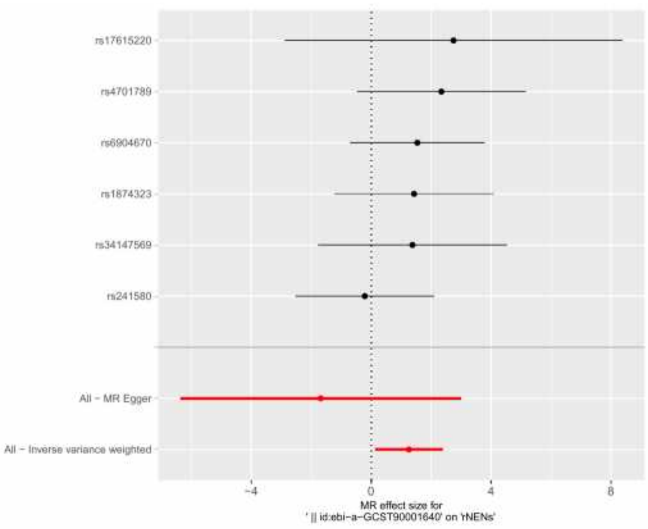
rs17615220
rs4701789
rs6904670
rs1874323
rs34147569
rs241580
All − MR Egger
All − Inverse variance weighted
−4
0
4
8
MR effect size for
' || id:ebi−a−GCST90001640' on 'rNENs'

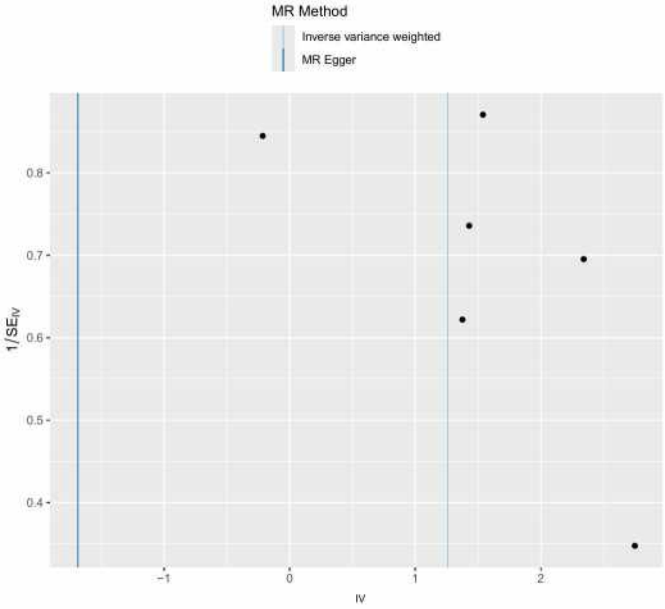
MR Method
Inverse variance weighted
MR Egger
1/SE_IV
0.8
0.7
0.6
0.5
0.4
−1
0
1
2
IV

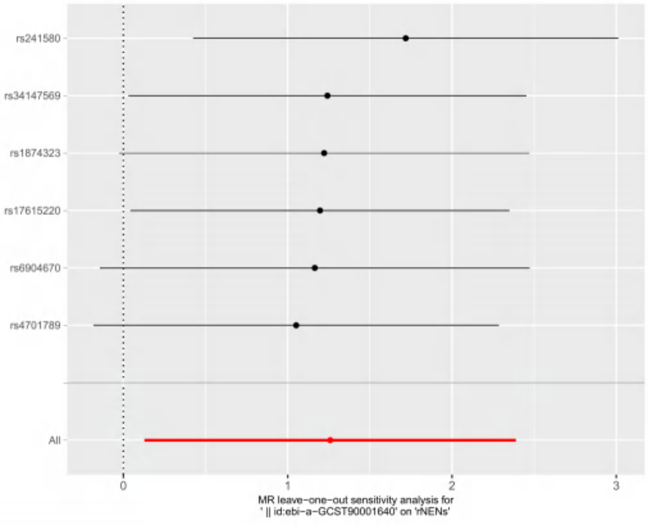
rs241580
rs34147569
rs1874323
rs17615220
rs6904670
rs4701789
All
0
1
2
3
MR leave−one−out sensitivity analysis for
' || id:ebi−a−GCST90001640' on 'rNENs'

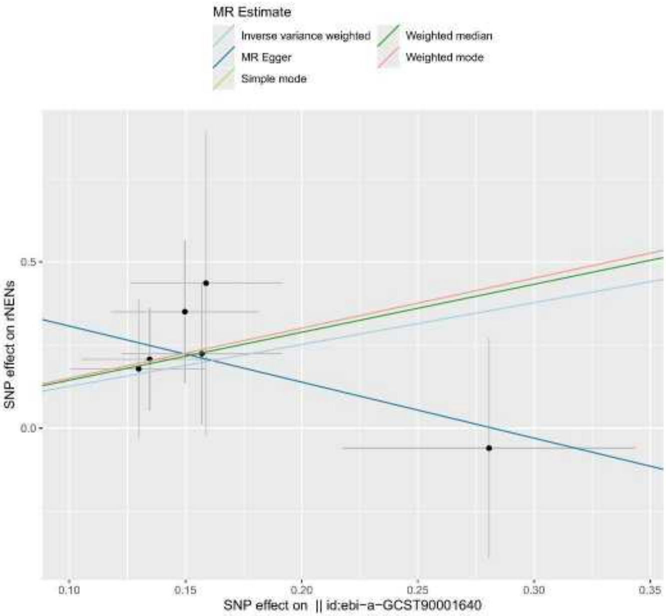
MR Estimate
Inverse variance weighted
MR Egger
Simple mode
Weighted median
Weighted mode
SNP effect on rNENs
0.5
0.0
0.10
0.15
0.20
0.25
0.30
0.35
SNP effect on || id:ebi−a−GCST90001640

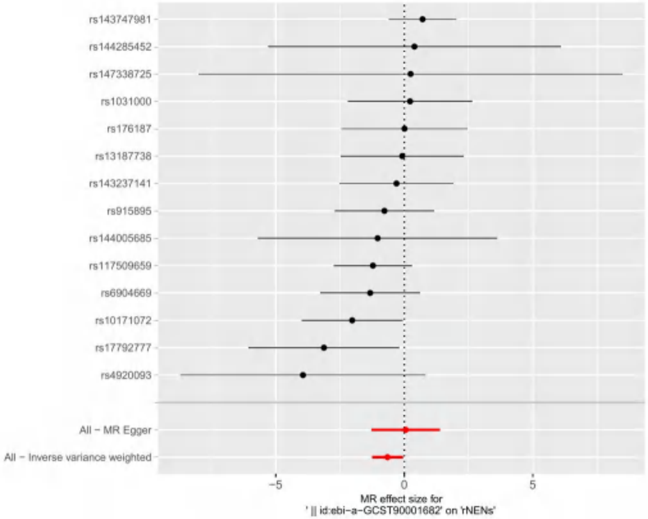
rs143747981
rs144285452
rs147338725
rs1031000
rs176187
rs13187738
rs143237141
rs915895
rs144005685
rs117509659
rs6904669
rs10171072
rs17792777
rs4920093
All − MR Egger
All − Inverse variance weighted
−5
0
5
MR effect size for
' || id:ebi−a−GCST90001682' on 'rNENs'

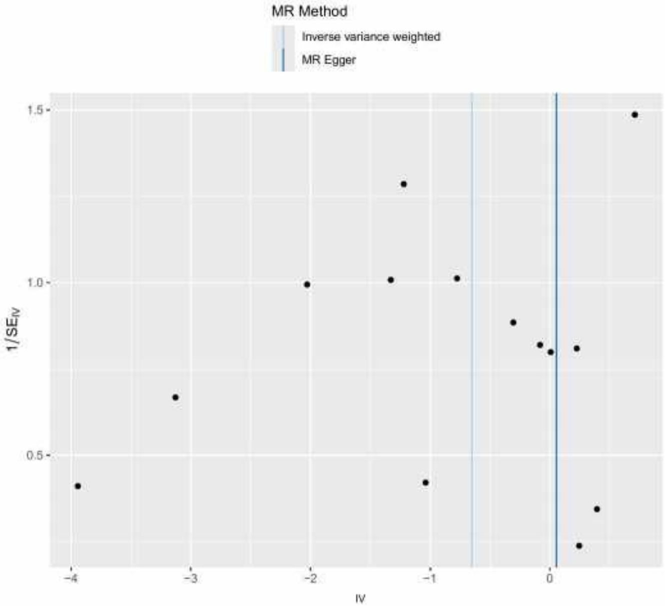
MR Method
Inverse variance weighted
MR Egger
1.5
1.0
0.5
1/SE$_{IV}$
−4
−3
−2
−1
0
IV

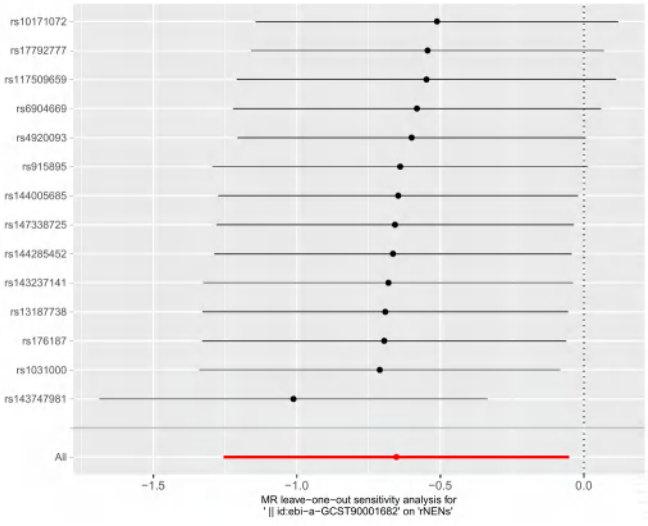
rs10171072
rs17792777
rs117509659
rs6904669
rs4920093
rs915895
rs144005685
rs147338725
rs144285452
rs143237141
rs13187738
rs176187
rs1031000
rs143747981
All
−1.5
−1.0
−0.5
0.0
MR leave−one−out sensitivity analysis for
' || id:ebi−a−GCST90001682' on 'rNENs'

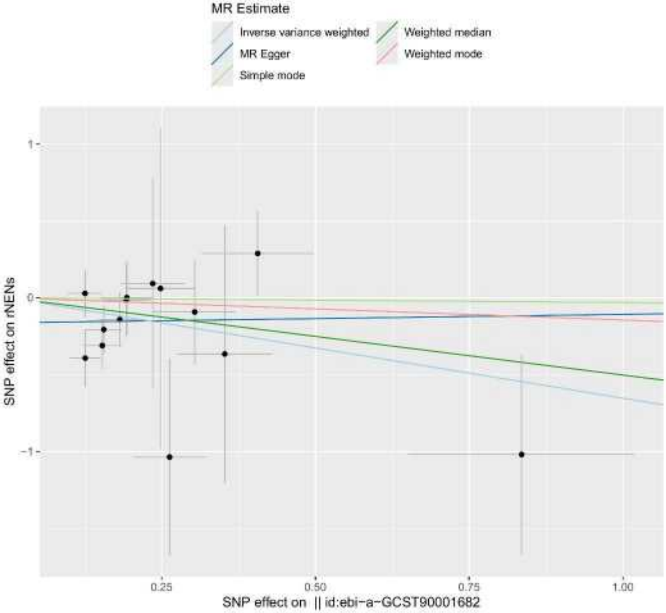
MR Estimate
Inverse variance weighted
MR Egger
Simple mode
Weighted median
Weighted mode
1
0
−1
SNP effect on rNENs
0.25
0.50
0.75
1.00
SNP effect on || id:ebi−a−GCST90001682

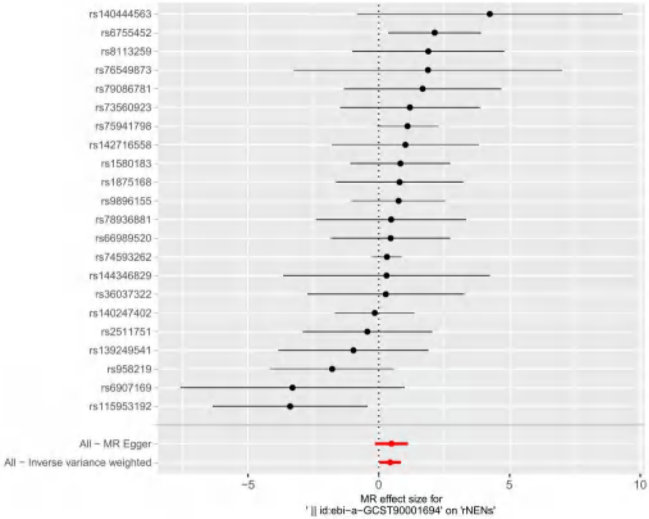
rs140444563
rs6755452
rs8113259
rs76549873
rs79086781
rs73560923
rs75941798
rs142716558
rs1580183
rs1875168
rs9896155
rs78936881
rs66989520
rs74593262
rs144346829
rs36037322
rs140247402
rs2511751
rs139249541
rs958219
rs6907169
rs115953192
All − MR Egger
All − Inverse variance weighted
−5
0
5
10
MR effect size for
' || id:ebi−a−GCST90001694' on 'rNENs'

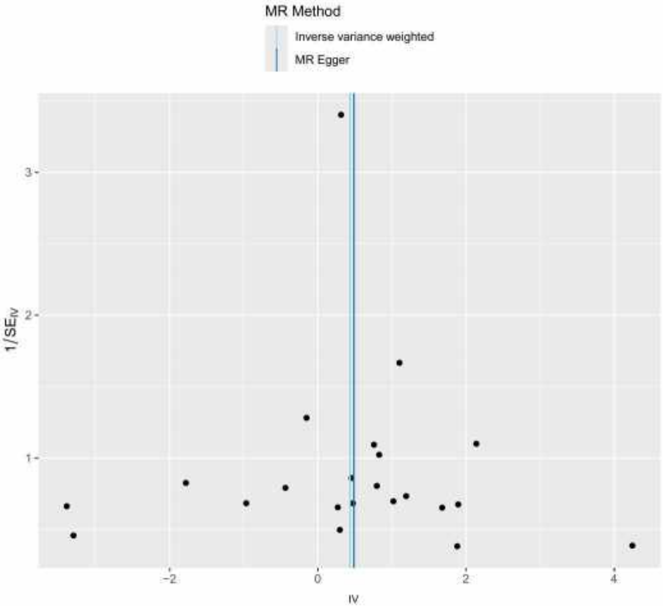
MR Method
Inverse variance weighted
MR Egger
1/SE$_{IV}$
3
2
1
−2
0
2
4
IV

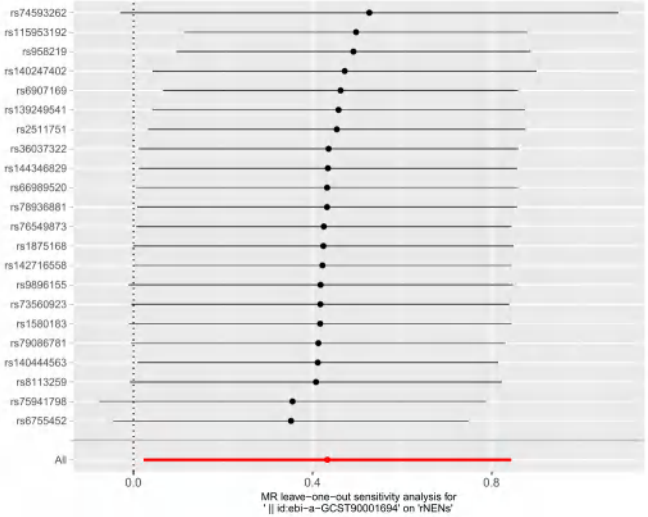
rs74593262
rs115953192
rs958219
rs140247402
rs6907169
rs139249541
rs2511751
rs36037322
rs144346829
rs66989520
rs78936881
rs76549873
rs1875168
rs142716558
rs9896155
rs73560923
rs1580183
rs79086781
rs140444563
rs8113259
rs75941798
rs6755452
All
0.0
0.4
0.8
MR leave−one−out sensitivity analysis for
' || id:ebi−a−GCST90001694' on 'rNENs'

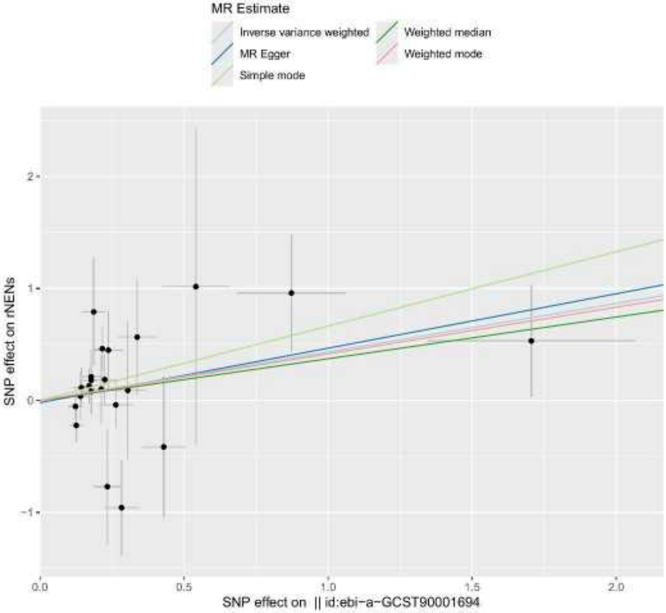
MR Estimate
Inverse variance weighted
MR Egger
Simple mode
Weighted median
Weighted mode
SNP effect on rNENs
2
1
0
−1
0.0
0.5
1.0
1.5
2.0
SNP effect on || id:ebi−a−GCST90001694

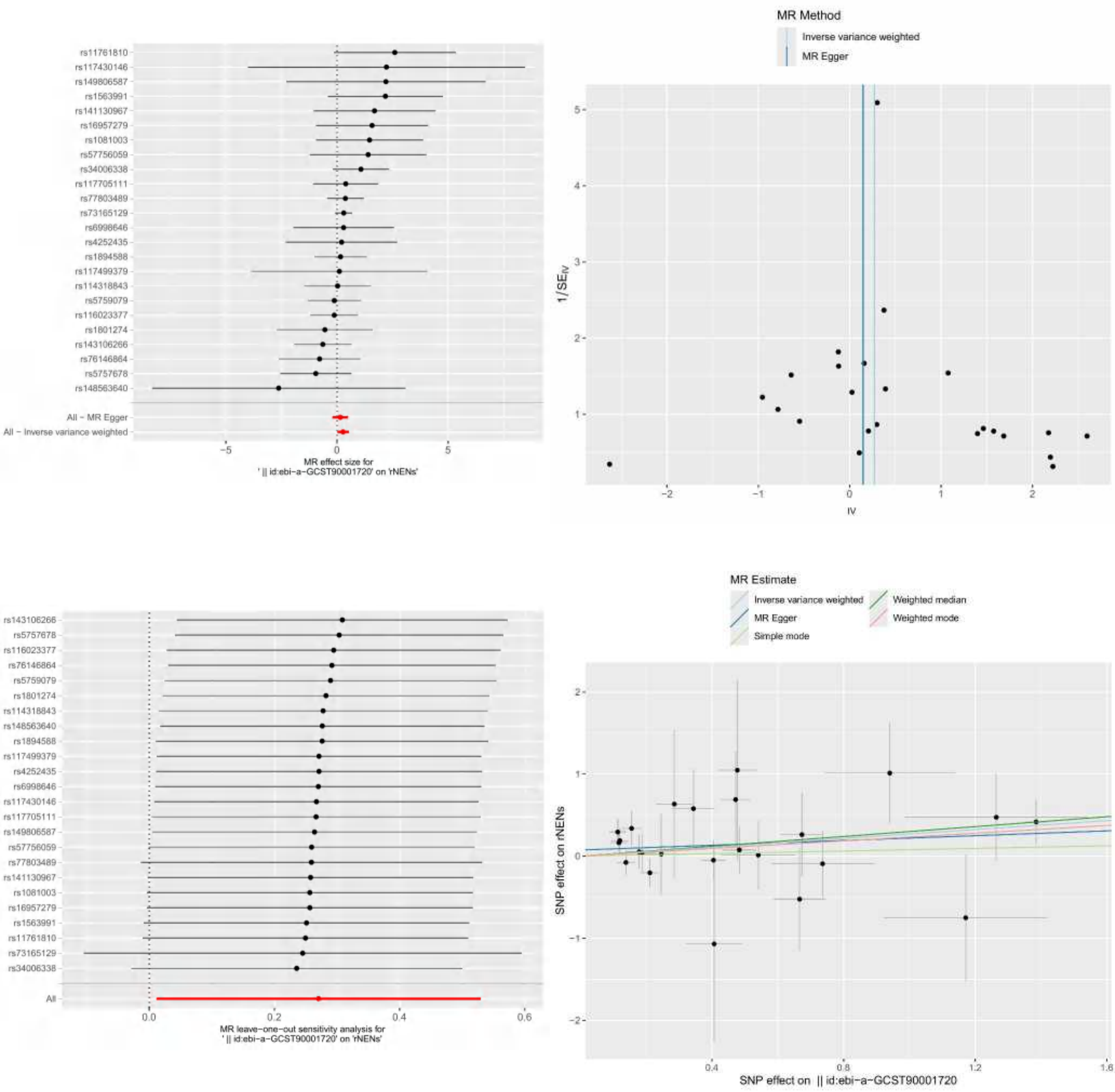
rs11761810
rs117430146
rs149806587
rs1563991
rs141130967
rs16957279
rs1081003
rs57756059
rs34006338
rs117705111
rs77803489
rs73165129
rs6998646
rs4252435
rs1894588
rs117499379
rs114318843
rs5759079
rs116023377
rs1801274
rs143106266
rs76146864
rs5757678
rs148563640
All - MR Egger
All - Inverse variance weighted
MR effect size for
' || id:ebi-a-GCST90001720' on 'rNENs'
MR Method
Inverse variance weighted
MR Egger
$1/SE_{IV}$
IV
rs143106266
rs5757678
rs116023377
rs76146864
rs5759079
rs1801274
rs114318843
rs148563640
rs1894588
rs117499379
rs4252435
rs6998646
rs117430146
rs117705111
rs149806587
rs57756059
rs77803489
rs141130967
rs1081003
rs16957279
rs1563991
rs11761810
rs73165129
rs34006338
All
MR leave-one-out sensitivity analysis for
' || id:ebi-a-GCST90001720' on 'rNENs'
MR Estimate
Inverse variance weighted
MR Egger
Simple mode
Weighted median
Weighted mode
SNP effect on rNENs
SNP effect on || id:ebi-a-GCST90001720

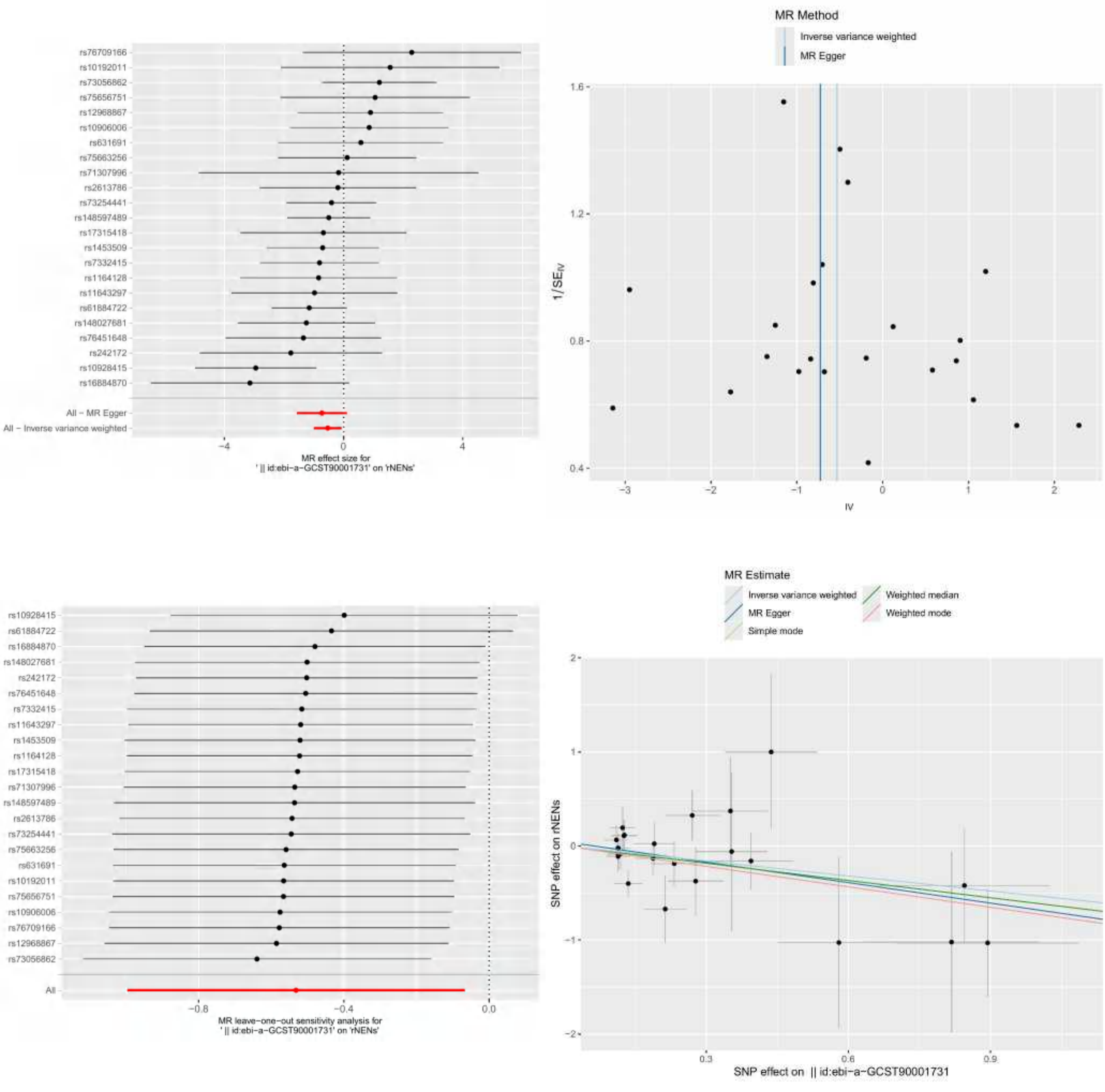

rs76709166
rs10192011
rs73056862
rs75656751
rs12968867
rs10906006
rs631691
rs75663256
rs71307996
rs2613786
rs73254441
rs148597489
rs17315418
rs1453509
rs7332415
rs1164128
rs11643297
rs61884722
rs148027681
rs76451648
rs242172
rs10928415
rs16884870
All - MR Egger
All - Inverse variance weighted
-4
0
4
MR effect size for
' || id:ebi-a-GCST90001731' on 'rNENs'
MR Method
Inverse variance weighted
MR Egger
1.6
1.2
0.8
0.4
1/SE$_{IV}$
-3
-2
-1
0
1
2
IV
rs10928415
rs61884722
rs16884870
rs148027681
rs242172
rs76451648
rs7332415
rs11643297
rs1453509
rs1164128
rs17315418
rs71307996
rs148597489
rs2613786
rs73254441
rs75663256
rs631691
rs10192011
rs75656751
rs10906006
rs76709166
rs12968867
rs73056862
All
-0.8
-0.4
0.0
MR leave-one-out sensitivity analysis for
' || id:ebi-a-GCST90001731' on 'rNENs'
MR Estimate
Inverse variance weighted
MR Egger
Simple mode
Weighted median
Weighted mode
2
1
0
-1
-2
SNP effect on rNENs
0.3
0.6
0.9
SNP effect on || id:ebi-a-GCST90001731

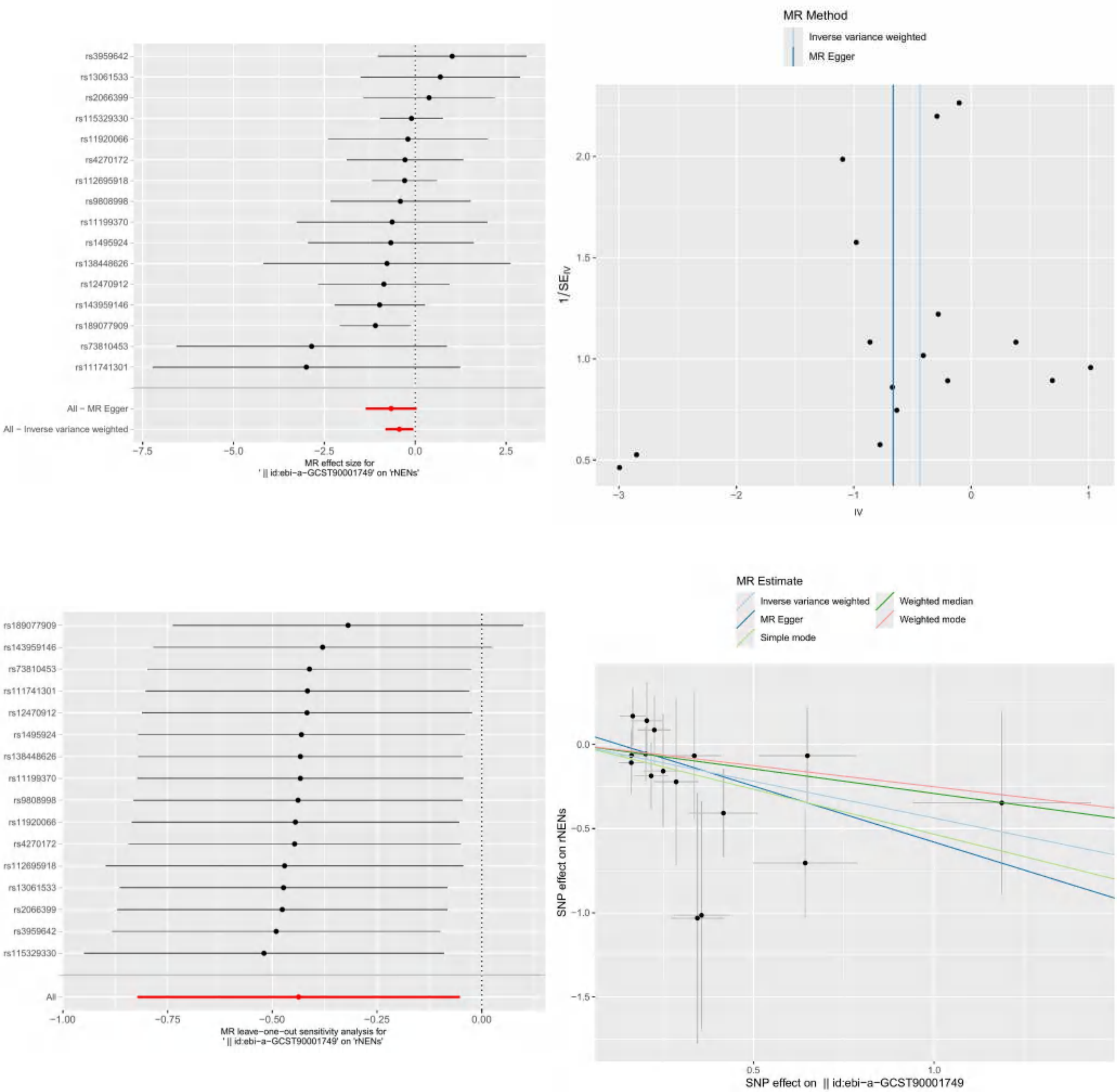

rs3959642
rs13061533
rs2066399
rs115329330
rs11920066
rs4270172
rs112695918
rs9808998
rs11199370
rs1495924
rs138448626
rs12470912
rs143959146
rs189077909
rs73810453
rs111741301
All − MR Egger
All − Inverse variance weighted
−7.5 −5.0 −2.5 0.0 2.5
MR effect size for
' || id:ebi−a−GCST90001749' on 'rNENs'
MR Method
Inverse variance weighted
MR Egger
1/SE_IV
0.5 1.0 1.5 2.0
−3 −2 −1 0 1
IV
rs189077909
rs143959146
rs73810453
rs111741301
rs12470912
rs1495924
rs138448626
rs11199370
rs9808998
rs11920066
rs4270172
rs112695918
rs13061533
rs2066399
rs3959642
rs115329330
All
−1.00 −0.75 −0.50 −0.25 0.00
MR leave−one−out sensitivity analysis for
' || id:ebi−a−GCST90001749' on 'rNENs'
MR Estimate
Inverse variance weighted
MR Egger
Simple mode
Weighted median
Weighted mode
SNP effect on rNENs
0.0 −0.5 −1.0 −1.5
0.5 1.0
SNP effect on || id:ebi−a−GCST90001749

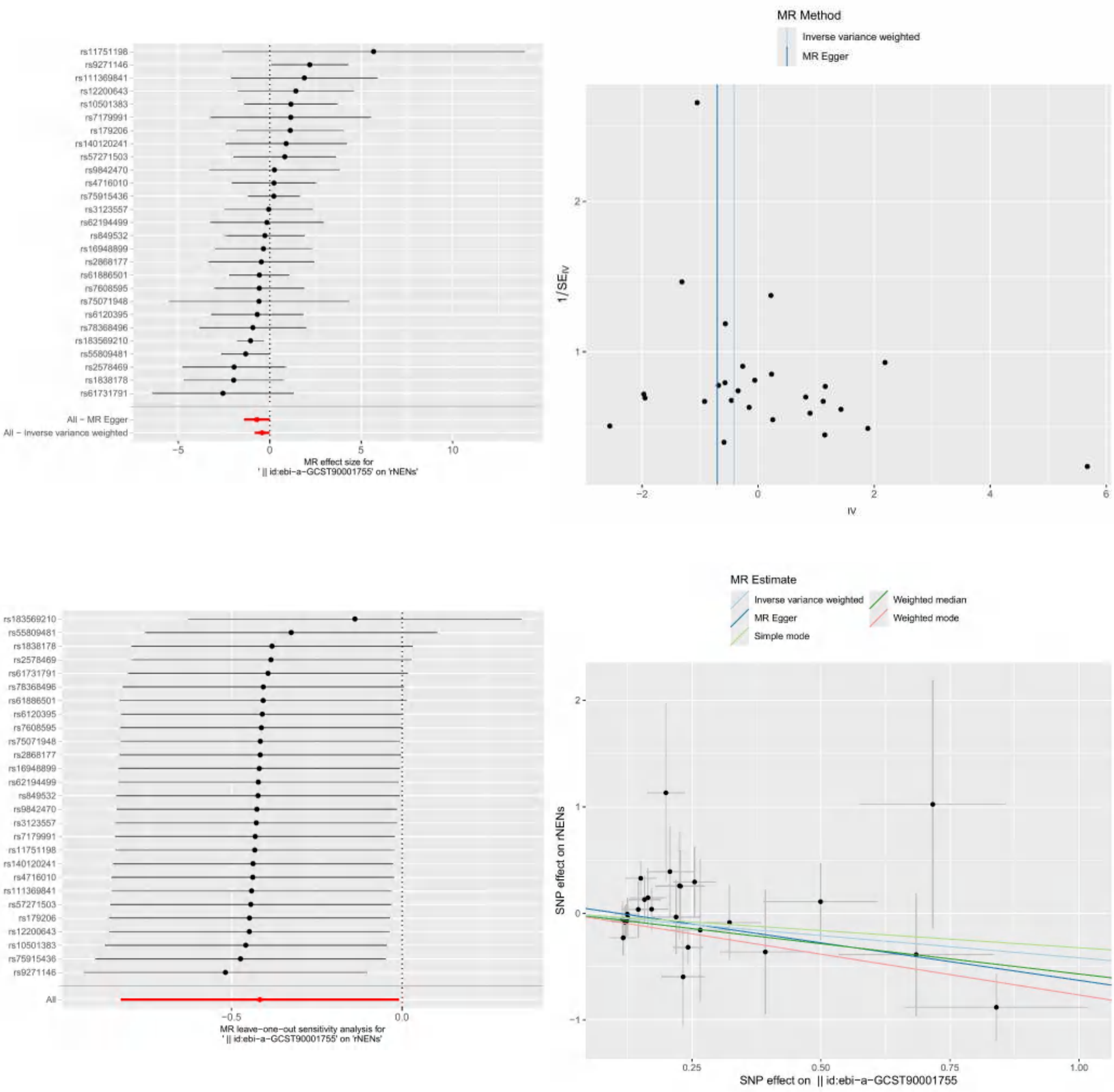

rs11751198
rs9271146
rs111369841
rs12200643
rs10501383
rs7179991
rs179206
rs140120241
rs57271503
rs9842470
rs4716010
rs75915436
rs3123557
rs62194499
rs849532
rs16948899
rs2868177
rs61886501
rs7608595
rs75071948
rs6120395
rs78368496
rs183569210
rs55809481
rs2578469
rs1838178
rs61731791
All − MR Egger
All − Inverse variance weighted
−5
0
5
10
MR effect size for
' || id:ebi−a−GCST90001755' on 'rNENs'
MR Method
Inverse variance weighted
MR Egger
1/SE$_{IV}$
IV
MR leave−one−out sensitivity analysis for
' || id:ebi−a−GCST90001755' on 'rNENs'
All
MR Estimate
Inverse variance weighted
MR Egger
Simple mode
Weighted median
Weighted mode
SNP effect on rNENs
SNP effect on || id:ebi−a−GCST90001755

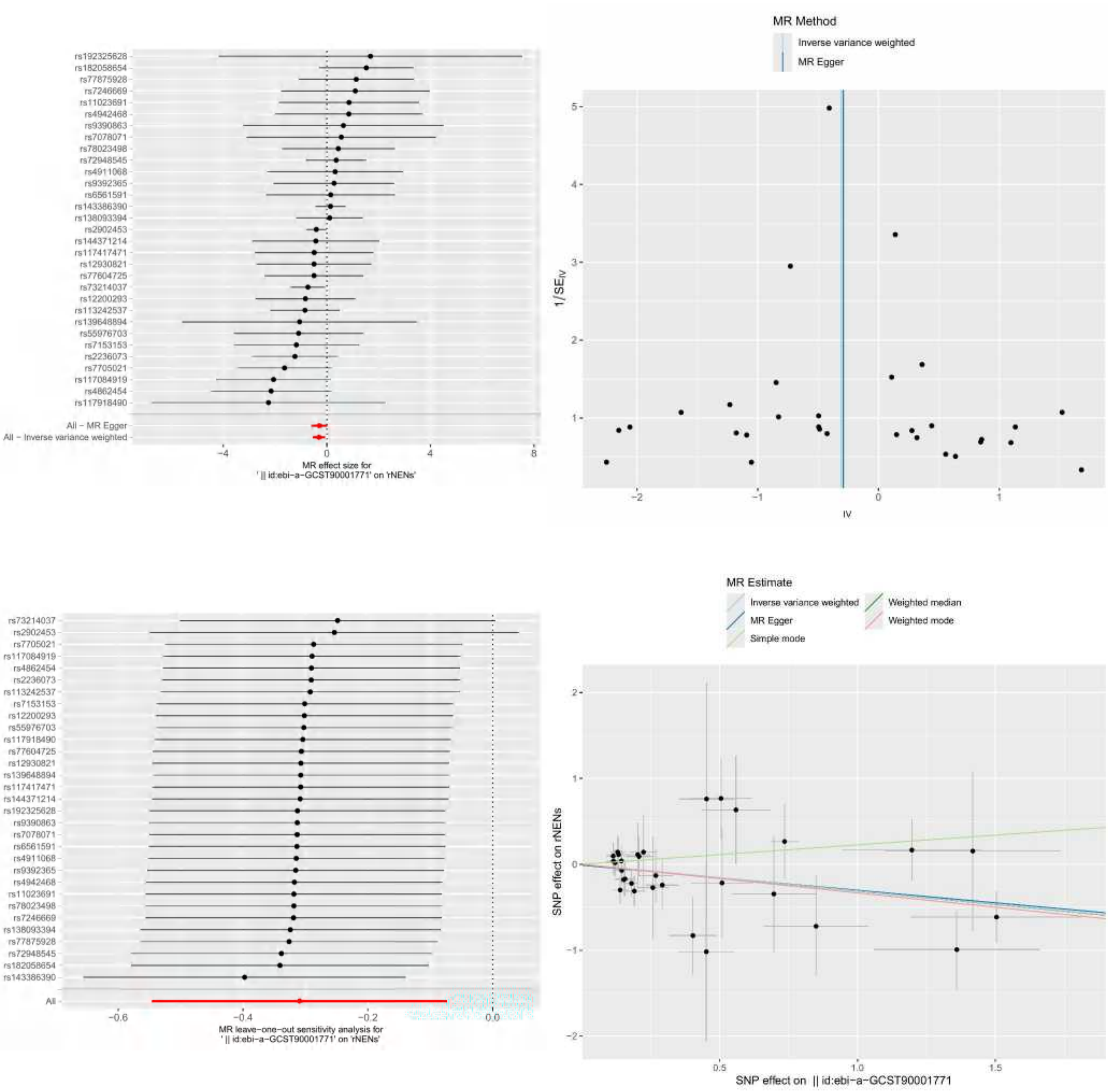
rs192325628
rs182058654
rs77875928
rs7246669
rs11023691
rs4942468
rs9390863
rs7078071
rs78023498
rs72948545
rs4911068
rs9392365
rs6561591
rs143386390
rs138093394
rs2902453
rs144371214
rs117417471
rs12930821
rs77604725
rs73214037
rs12200293
rs113242537
rs139648894
rs55976703
rs7153153
rs2236073
rs7705021
rs117084919
rs4862454
rs117918490
All - MR Egger
All - Inverse variance weighted
MR effect size for
' || id:ebi-a-GCST90001771' on 'rNENs'
MR Method
Inverse variance weighted
MR Egger
1/SE_IV
IV
rs73214037
rs2902453
rs7705021
rs117084919
rs4862454
rs2236073
rs113242537
rs7153153
rs12200293
rs55976703
rs117918490
rs77604725
rs12930821
rs139648894
rs117417471
rs144371214
rs192325628
rs9390863
rs7078071
rs6561591
rs4911068
rs9392365
rs4942468
rs11023691
rs78023498
rs7246669
rs138093394
rs77875928
rs72948545
rs182058654
rs143386390
All
MR leave-one-out sensitivity analysis for
' || id:ebi-a-GCST90001771' on 'rNENs'
MR Estimate
Inverse variance weighted
MR Egger
Simple mode
Weighted median
Weighted mode
SNP effect on rNENs
SNP effect on || id:ebi-a-GCST90001771

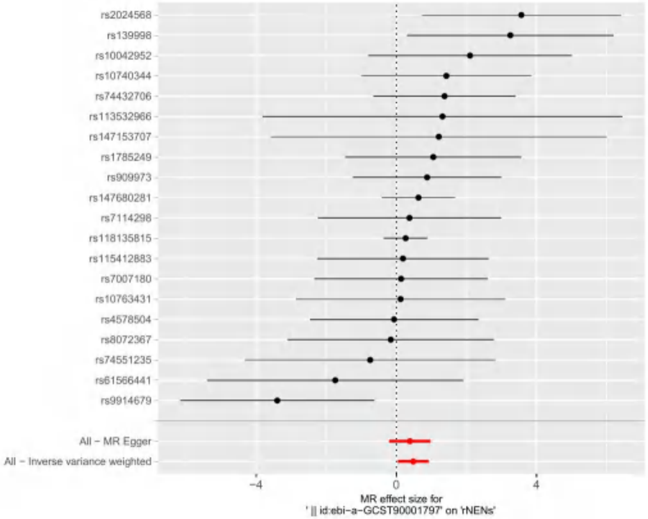

rs2024568
rs139998
rs10042952
rs10740344
rs74432706
rs113532966
rs147153707
rs1785249
rs909973
rs147680281
rs7114298
rs118135815
rs115412883
rs7007180
rs10763431
rs4578504
rs8072367
rs74551235
rs61566441
rs9914679
All − MR Egger
All − Inverse variance weighted
−4
0
4
MR effect size for
' || id:ebi−a−GCST90001797' on 'rNENs'

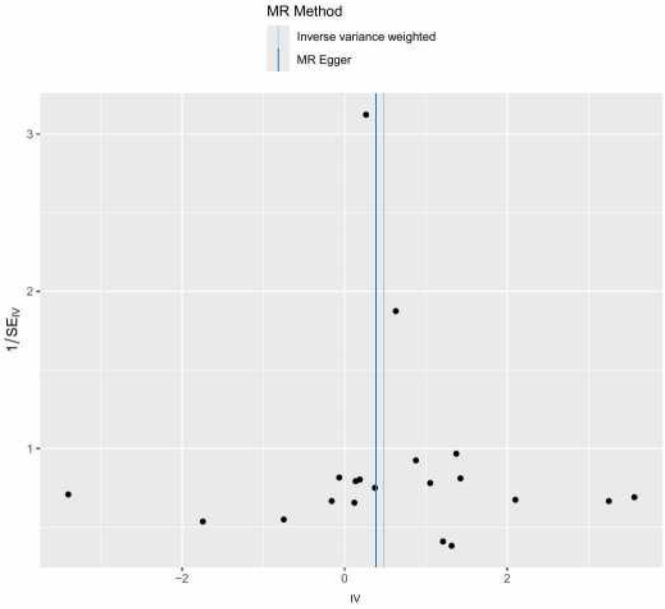

MR Method
Inverse variance weighted
MR Egger
1/SE_IV
3
2
1
−2
0
2
IV

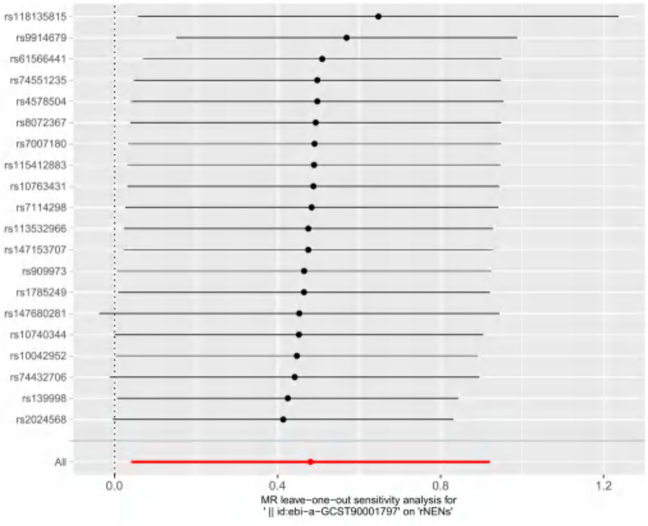

rs118135815
rs9914679
rs61566441
rs74551235
rs4578504
rs8072367
rs7007180
rs115412883
rs10763431
rs7114298
rs113532966
rs147153707
rs909973
rs1785249
rs147680281
rs10740344
rs10042952
rs74432706
rs139998
rs2024568
All
0.0
0.4
0.8
1.2
MR leave−one−out sensitivity analysis for
' || id:ebi−a−GCST90001797' on 'rNENs'

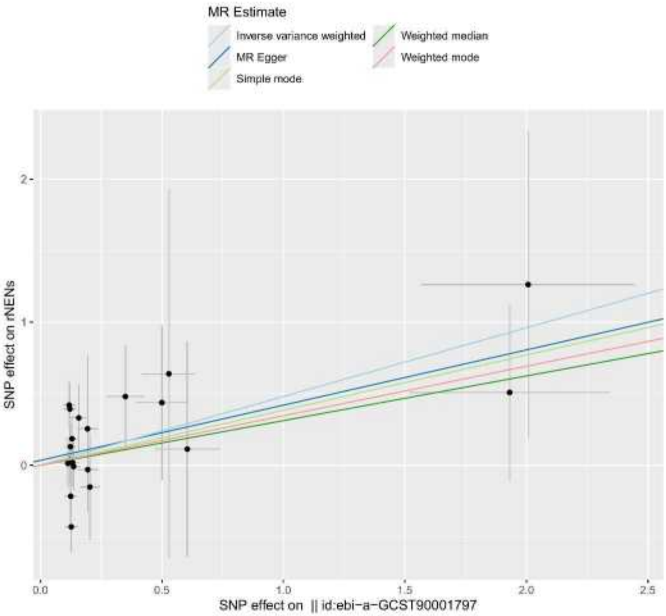

MR Estimate
Inverse variance weighted
MR Egger
Simple mode
Weighted median
Weighted mode
SNP effect on rNENs
2
1
0
0.0
0.5
1.0
1.5
2.0
2.5
SNP effect on || id:ebi−a−GCST90001797

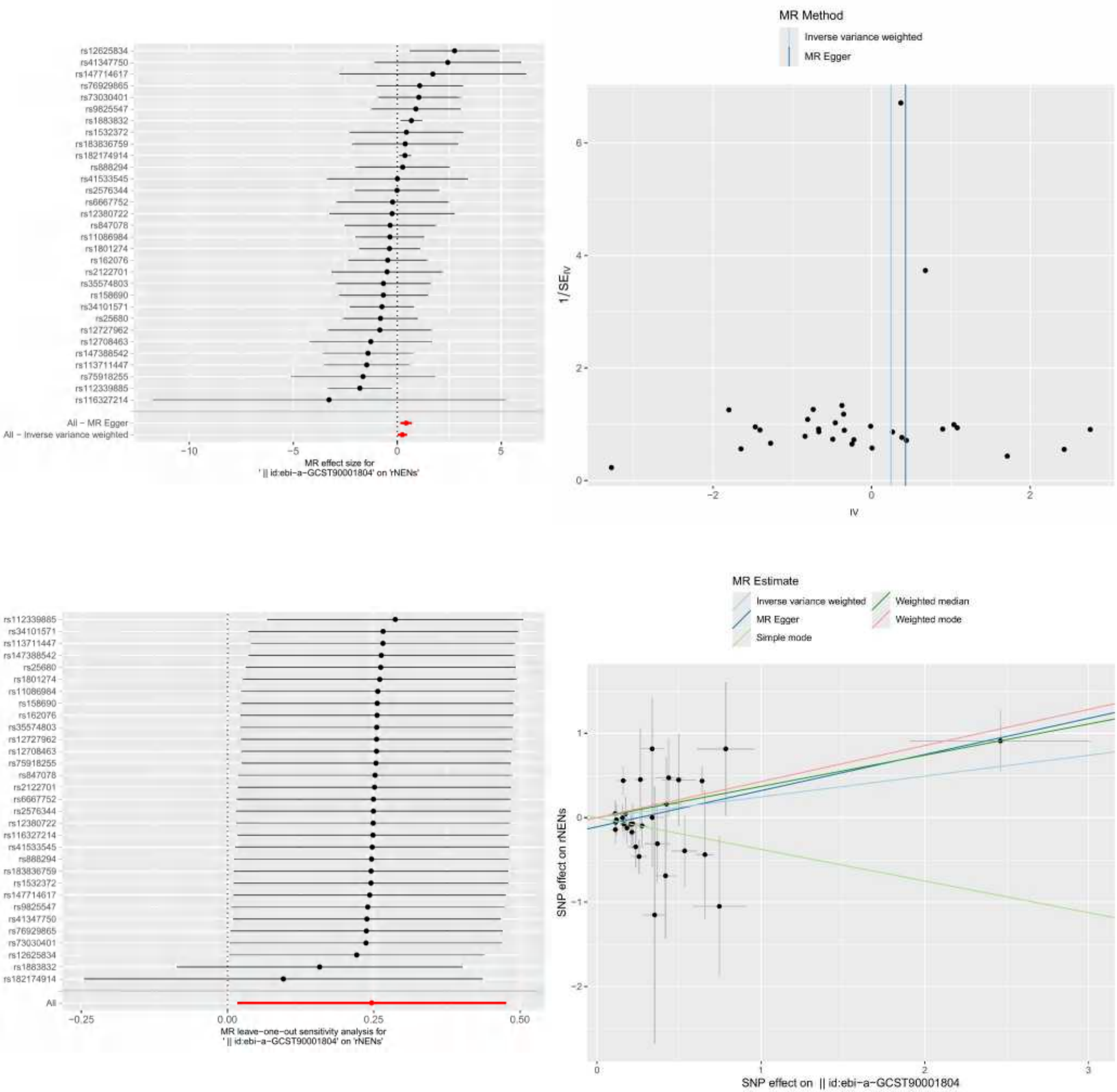

rs12625834
rs41347750
rs147714617
rs76929865
rs73030401
rs9825547
rs1883832
rs1532372
rs183836759
rs182174914
rs888294
rs41533545
rs2576344
rs6667752
rs12380722
rs847078
rs11086984
rs1801274
rs162076
rs2122701
rs35574803
rs158690
rs34101571
rs25680
rs12727962
rs12708463
rs147388542
rs113711447
rs75918255
rs112339885
rs116327214
All − MR Egger
All − Inverse variance weighted
−10
−5
0
5
MR effect size for
' || id:ebi−a−GCST90001804' on 'rNENs'
MR Method
Inverse variance weighted
MR Egger
1/SE$_{IV}$
IV
0
2
4
6
−2
0
2
rs112339885
rs34101571
rs113711447
rs147388542
rs25680
rs1801274
rs11086984
rs158690
rs162076
rs35574803
rs12727962
rs12708463
rs75918255
rs847078
rs2122701
rs6667752
rs2576344
rs12380722
rs116327214
rs41533545
rs888294
rs183836759
rs1532372
rs147714617
rs9825547
rs41347750
rs76929865
rs73030401
rs12625834
rs1883832
rs182174914
All
−0.25
0.00
0.25
0.50
MR leave−one−out sensitivity analysis for
' || id:ebi−a−GCST90001804' on 'rNENs'
MR Estimate
Inverse variance weighted
MR Egger
Simple mode
Weighted median
Weighted mode
SNP effect on rNENs
SNP effect on || id:ebi−a−GCST90001804
1
0
−1
−2
0
1
2
3

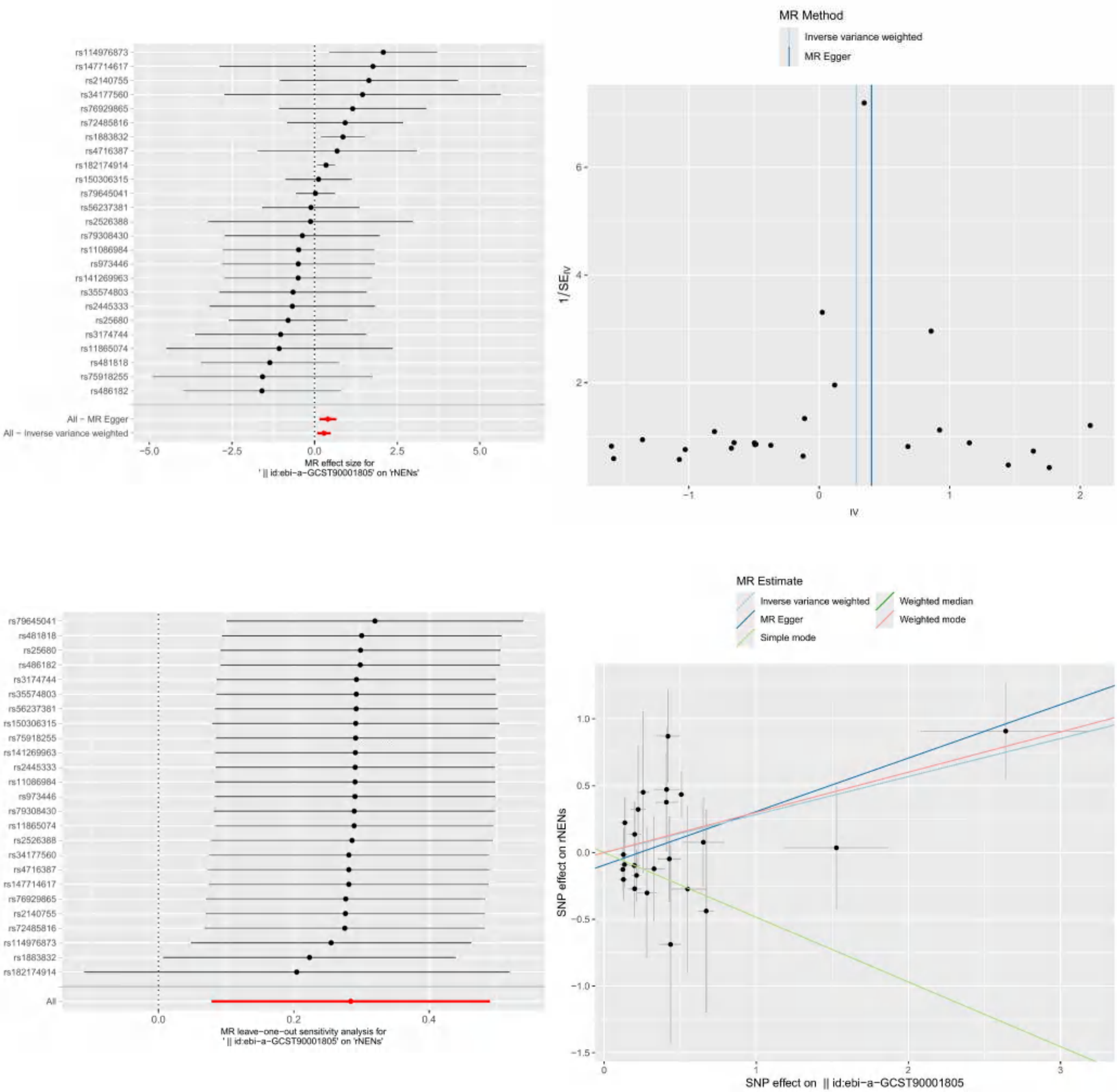

rs114976873
rs147714617
rs2140755
rs34177560
rs76929865
rs72485816
rs1883832
rs4716387
rs182174914
rs150306315
rs79645041
rs56237381
rs2526388
rs79308430
rs11086984
rs973446
rs141269963
rs35574803
rs2445333
rs25680
rs3174744
rs11865074
rs481818
rs75918255
rs486182
All − MR Egger
All − Inverse variance weighted
−5.0
−2.5
0.0
2.5
5.0
MR effect size for
' || id:ebi−a−GCST90001805' on 'rNENs'
MR Method
Inverse variance weighted
MR Egger
1/SE_IV
IV
MR leave−one−out sensitivity analysis for
' || id:ebi−a−GCST90001805' on 'rNENs'
All
MR Estimate
Inverse variance weighted
MR Egger
Simple mode
Weighted median
Weighted mode
SNP effect on rNENs
SNP effect on || id:ebi−a−GCST90001805

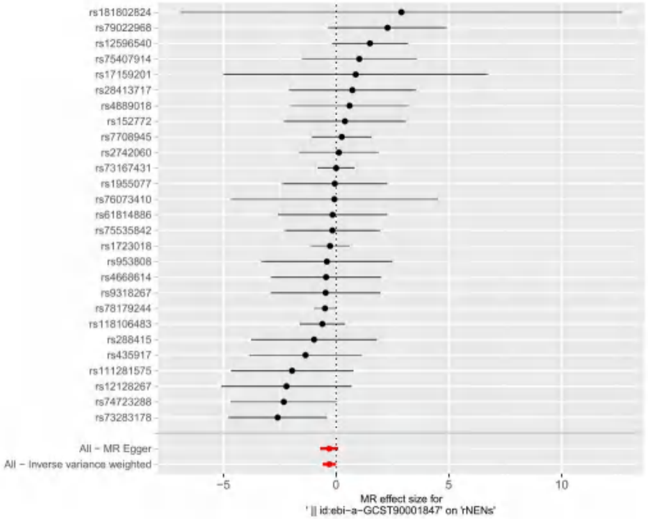
rs181802824
rs79022968
rs12596540
rs75407914
rs17159201
rs28413717
rs4889018
rs152772
rs7708945
rs2742060
rs73167431
rs1955077
rs76073410
rs61814886
rs75535842
rs1723018
rs953808
rs4668614
rs9318267
rs78179244
rs118106483
rs288415
rs435917
rs111281575
rs12128267
rs74723288
rs73283178
All − MR Egger
All − Inverse variance weighted
-5
0
5
10
MR effect size for
' || id:ebi-a-GCST90001847' on 'rNENs'

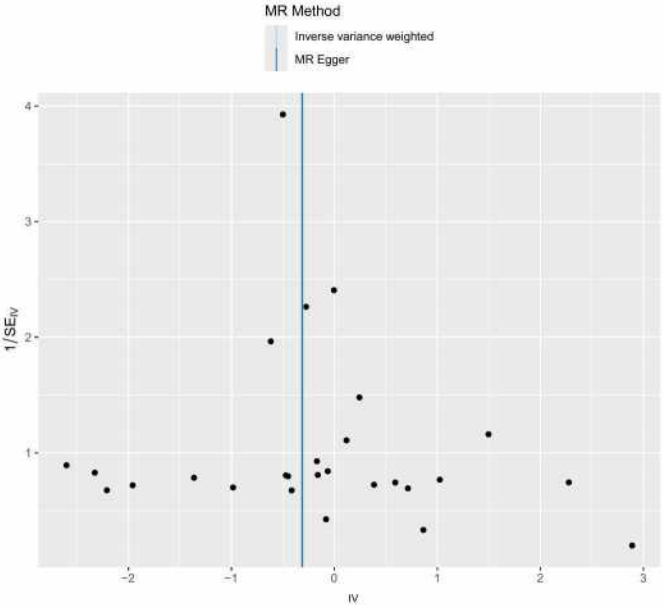
MR Method
Inverse variance weighted
MR Egger
1/SE_IV
4
3
2
1
-2
-1
0
1
2
3
IV

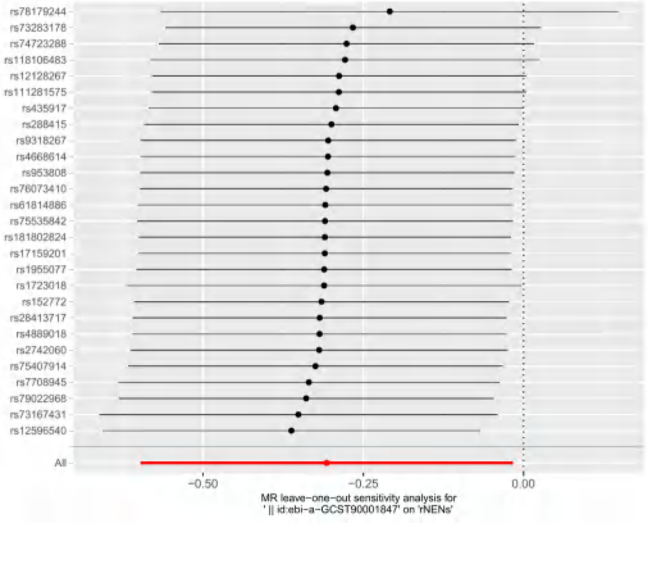
rs78179244
rs73283178
rs74723288
rs118106483
rs12128267
rs111281575
rs435917
rs288415
rs9318267
rs4668614
rs953808
rs76073410
rs61814886
rs75535842
rs181802824
rs17159201
rs1955077
rs1723018
rs152772
rs28413717
rs4889018
rs2742060
rs75407914
rs7708945
rs79022968
rs73167431
rs12596540
All
-0.50
-0.25
0.00
MR leave-one-out sensitivity analysis for
' || id:ebi-a-GCST90001847' on 'rNENs'

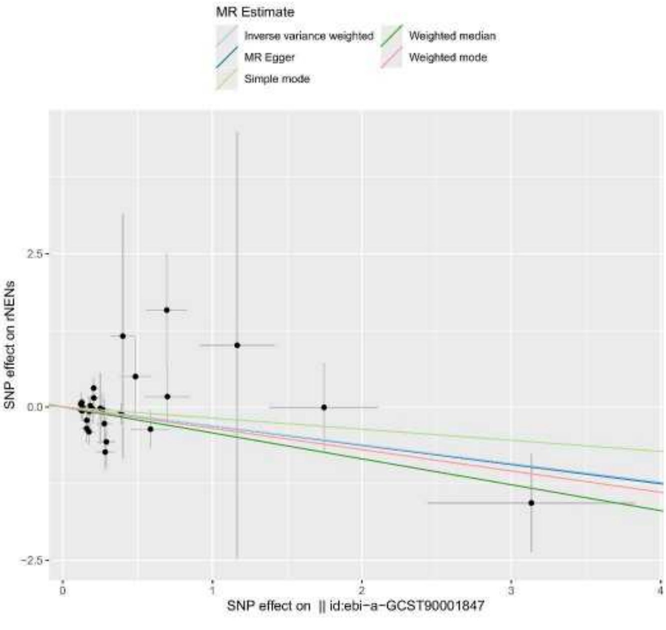
MR Estimate
Inverse variance weighted
MR Egger
Simple mode
Weighted median
Weighted mode
SNP effect on rNENs
2.5
0.0
-2.5
0
1
2
3
4
SNP effect on || id:ebi-a-GCST90001847

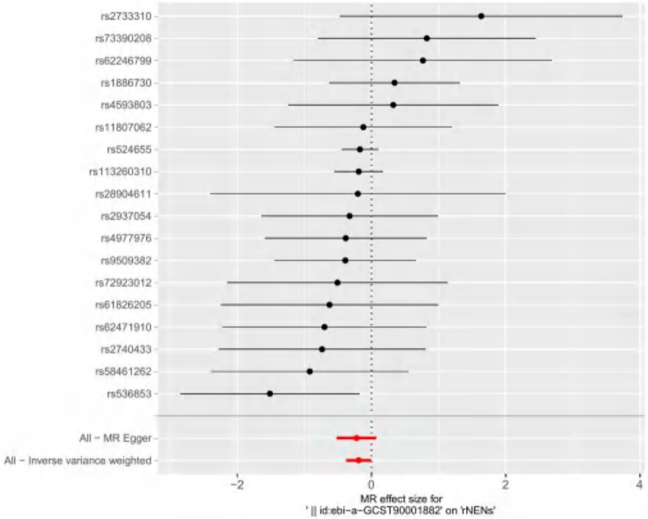
rs2733310
rs73390208
rs62246799
rs1886730
rs4593803
rs11807062
rs524655
rs113260310
rs28904611
rs2937054
rs4977976
rs9509382
rs72923012
rs61826205
rs62471910
rs2740433
rs58461262
rs536853
All − MR Egger
All − Inverse variance weighted
−2
0
2
4
MR effect size for
' || id:ebi−a−GCST90001882' on 'rNENs'

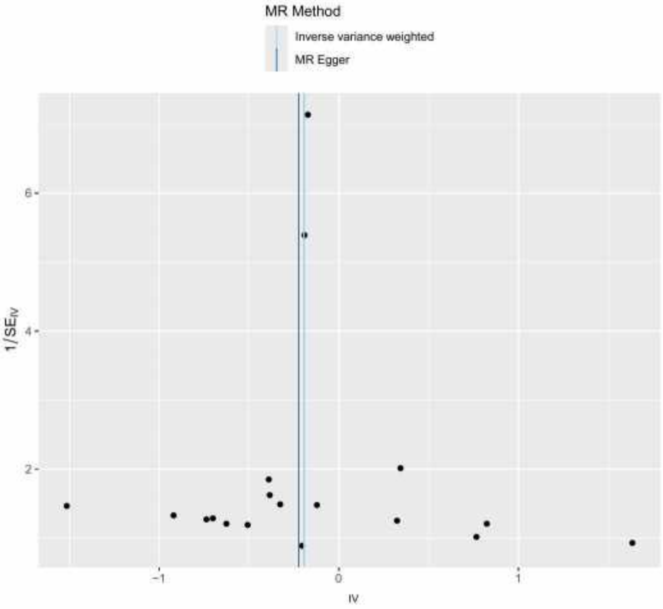
MR Method
Inverse variance weighted
MR Egger
1/SE_IV
6
4
2
−1
0
1
IV

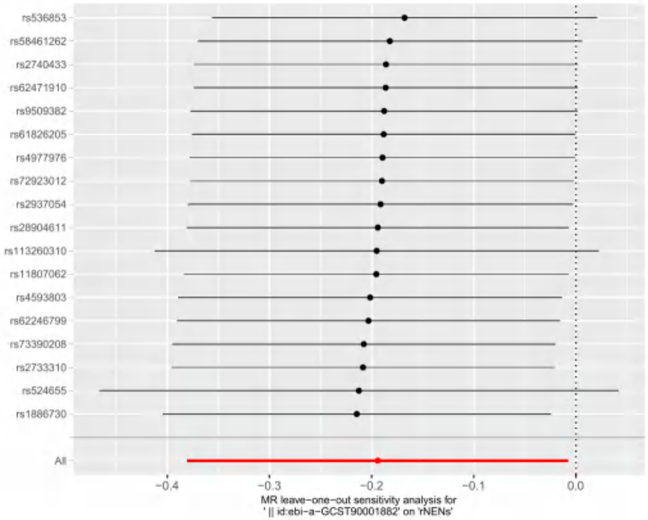
rs536853
rs58461262
rs2740433
rs62471910
rs9509382
rs61826205
rs4977976
rs72923012
rs2937054
rs28904611
rs113260310
rs11807062
rs4593803
rs62246799
rs73390208
rs2733310
rs524655
rs1886730
All
−0.4
−0.3
−0.2
−0.1
0.0
MR leave−one−out sensitivity analysis for
' || id:ebi−a−GCST90001882' on 'rNENs'

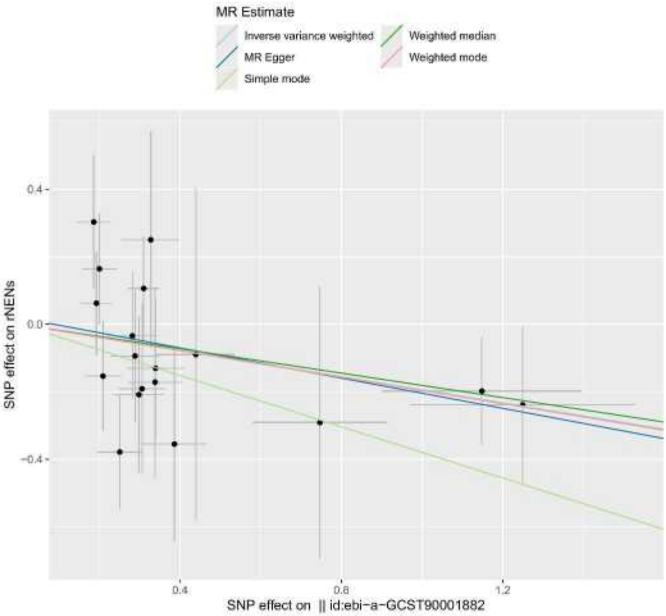
MR Estimate
Inverse variance weighted
MR Egger
Simple mode
Weighted median
Weighted mode
SNP effect on rNENs
0.4
0.0
−0.4
0.4
0.8
1.2
SNP effect on || id:ebi−a−GCST90001882

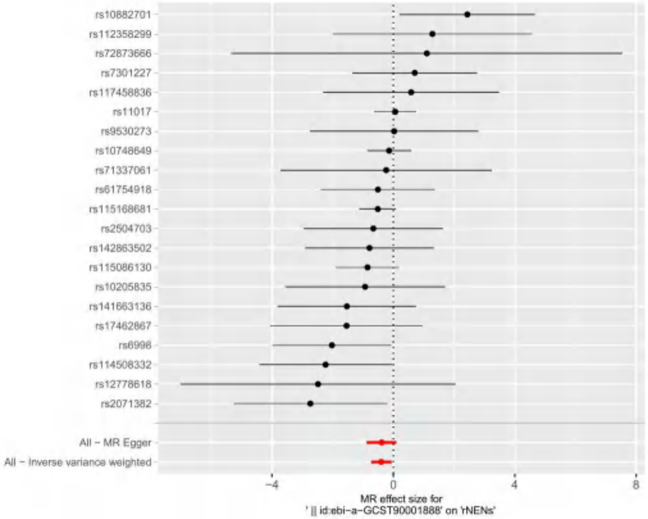
rs10882701
rs112358299
rs72873666
rs7301227
rs117458836
rs11017
rs9530273
rs10748649
rs71337061
rs61754918
rs115168681
rs2504703
rs142863502
rs115086130
rs10205835
rs141663136
rs17462867
rs6998
rs114508332
rs12778618
rs2071382
All − MR Egger
All − Inverse variance weighted
-4
0
4
8
MR effect size for
' || id:ebi−a−GCST90001888' on 'rNENs'

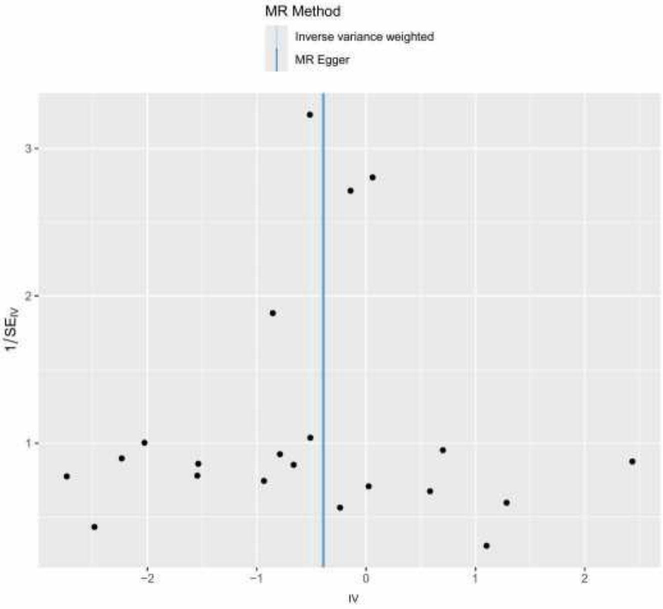
MR Method
Inverse variance weighted
MR Egger
1/SE$_{IV}$
3
2
1
−2
−1
0
1
2
IV

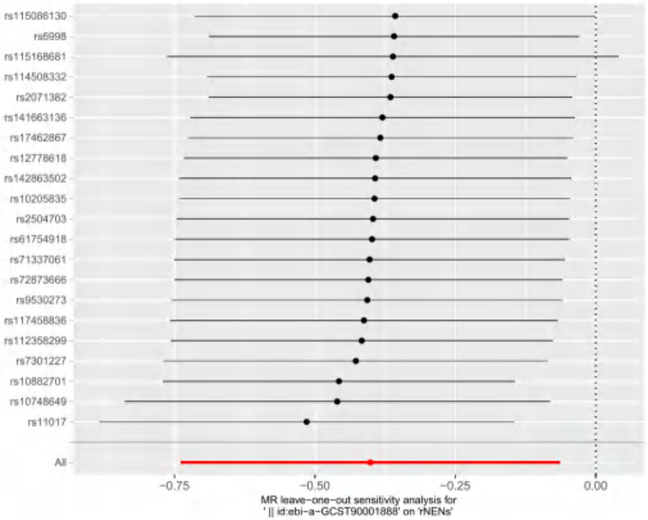
rs115086130
rs6998
rs115168681
rs114508332
rs2071382
rs141663136
rs17462867
rs12778618
rs142863502
rs10205835
rs2504703
rs61754918
rs71337061
rs72873666
rs9530273
rs117458836
rs112358299
rs7301227
rs10882701
rs10748649
rs11017
All
−0.75
−0.50
−0.25
0.00
MR leave−one−out sensitivity analysis for
' || id:ebi−a−GCST90001888' on 'rNENs'

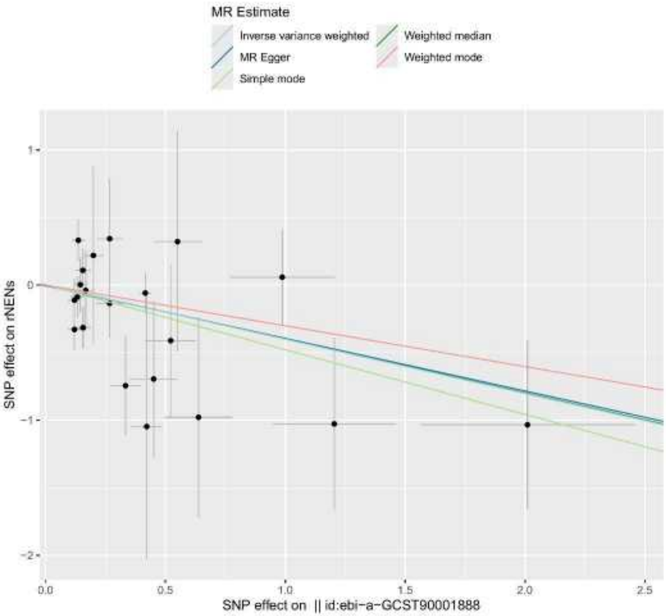
MR Estimate
Inverse variance weighted
MR Egger
Simple mode
Weighted median
Weighted mode
SNP effect on rNENs
1
0
−1
−2
0.0
0.5
1.0
1.5
2.0
2.5
SNP effect on || id:ebi−a−GCST90001888

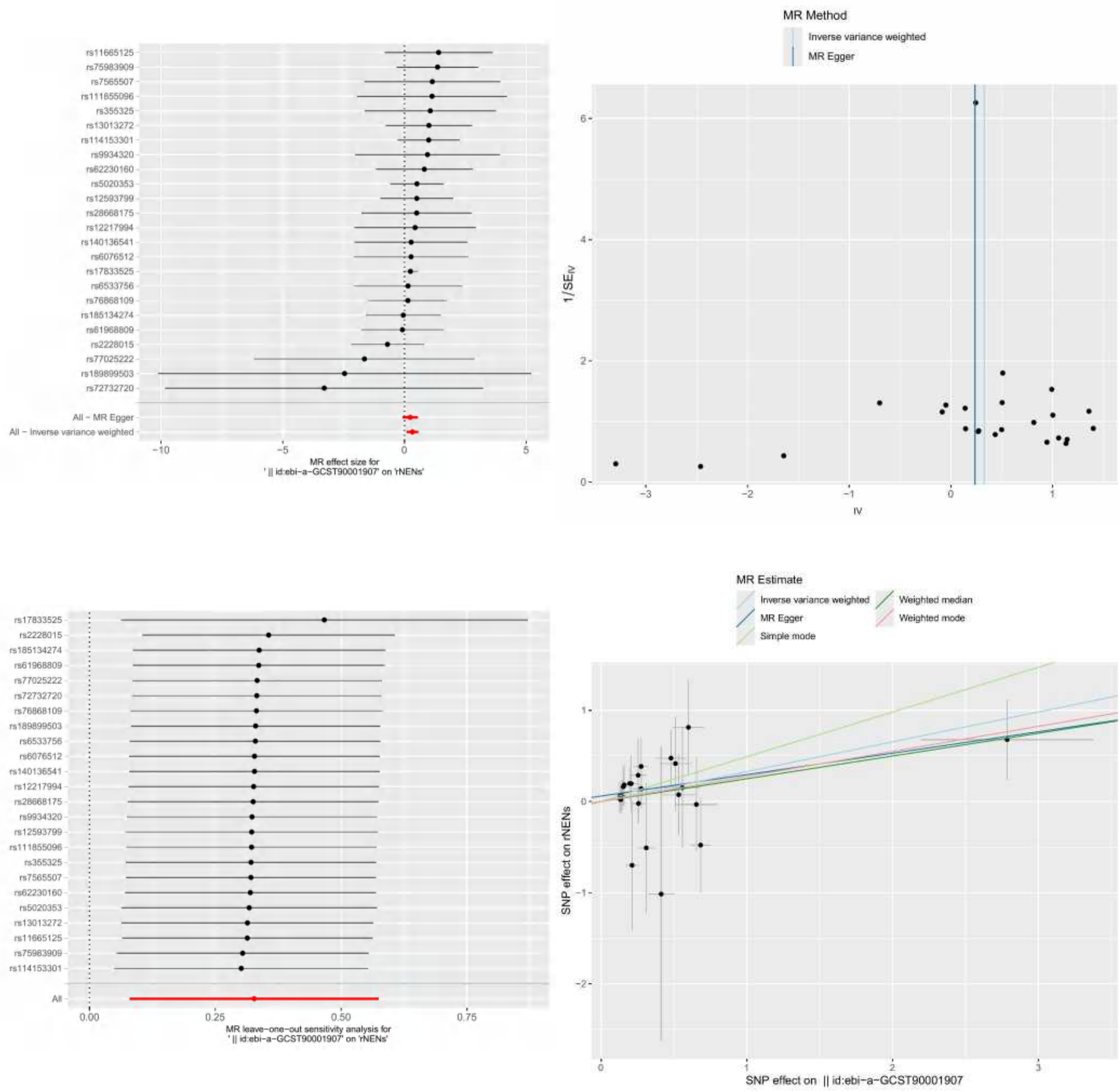

rs11665125
rs75983909
rs7565507
rs111855096
rs355325
rs13013272
rs114153301
rs9934320
rs62230160
rs5020353
rs12593799
rs28668175
rs12217994
rs140136541
rs6076512
rs17833525
rs6533756
rs76868109
rs185134274
rs61968809
rs2228015
rs77025222
rs189899503
rs72732720
All − MR Egger
All − Inverse variance weighted
−10
−5
0
5
MR effect size for
' || id:ebi−a−GCST90001907' on 'rNENs'
MR Method
Inverse variance weighted
MR Egger
1/SE_IV
0
2
4
6
−3
−2
−1
0
1
IV
rs17833525
rs2228015
rs185134274
rs61968809
rs77025222
rs72732720
rs76868109
rs189899503
rs6533756
rs6076512
rs140136541
rs12217994
rs28668175
rs9934320
rs12593799
rs111855096
rs355325
rs7565507
rs62230160
rs5020353
rs13013272
rs11665125
rs75983909
rs114153301
All
0.00
0.25
0.50
0.75
MR leave−one−out sensitivity analysis for
' || id:ebi−a−GCST90001907' on 'rNENs'
MR Estimate
Inverse variance weighted
MR Egger
Simple mode
Weighted median
Weighted mode
SNP effect on rNENs
1
0
−1
−2
0
1
2
3
SNP effect on || id:ebi−a−GCST90001907

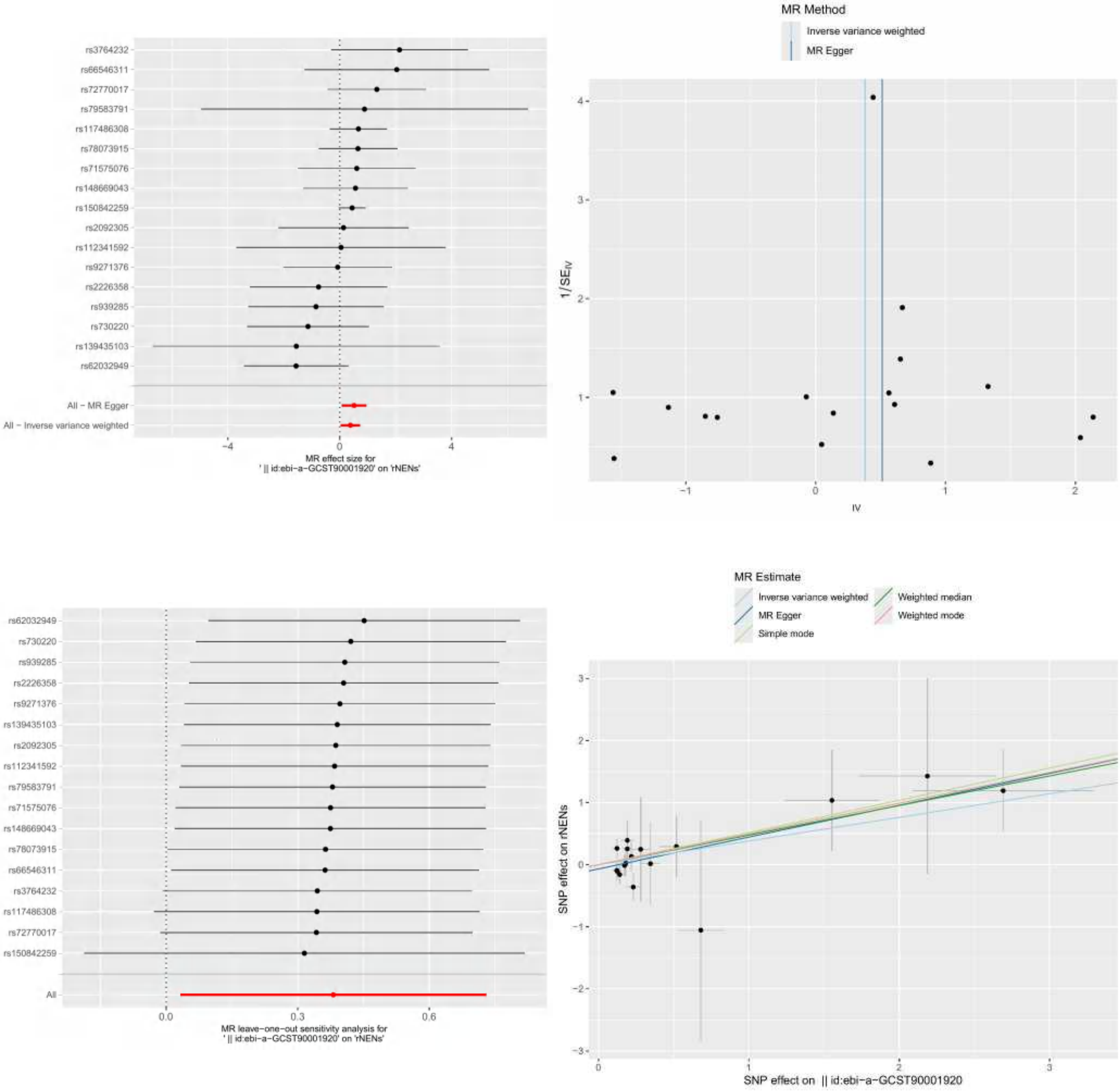
rs3764232
rs66546311
rs72770017
rs79583791
rs117486308
rs78073915
rs71575076
rs148669043
rs150842259
rs2092305
rs112341592
rs9271376
rs2226358
rs939285
rs730220
rs139435103
rs62032949
All − MR Egger
All − Inverse variance weighted
−4
0
4
MR effect size for
' || id:ebi−a−GCST90001920' on 'rNENs'
MR Method
Inverse variance weighted
MR Egger
1/SE_IV
IV
MR leave−one−out sensitivity analysis for
' || id:ebi−a−GCST90001920' on 'rNENs'
All
0.0
0.3
0.6
MR Estimate
Inverse variance weighted
MR Egger
Simple mode
Weighted median
Weighted mode
SNP effect on rNENs
SNP effect on || id:ebi−a−GCST90001920

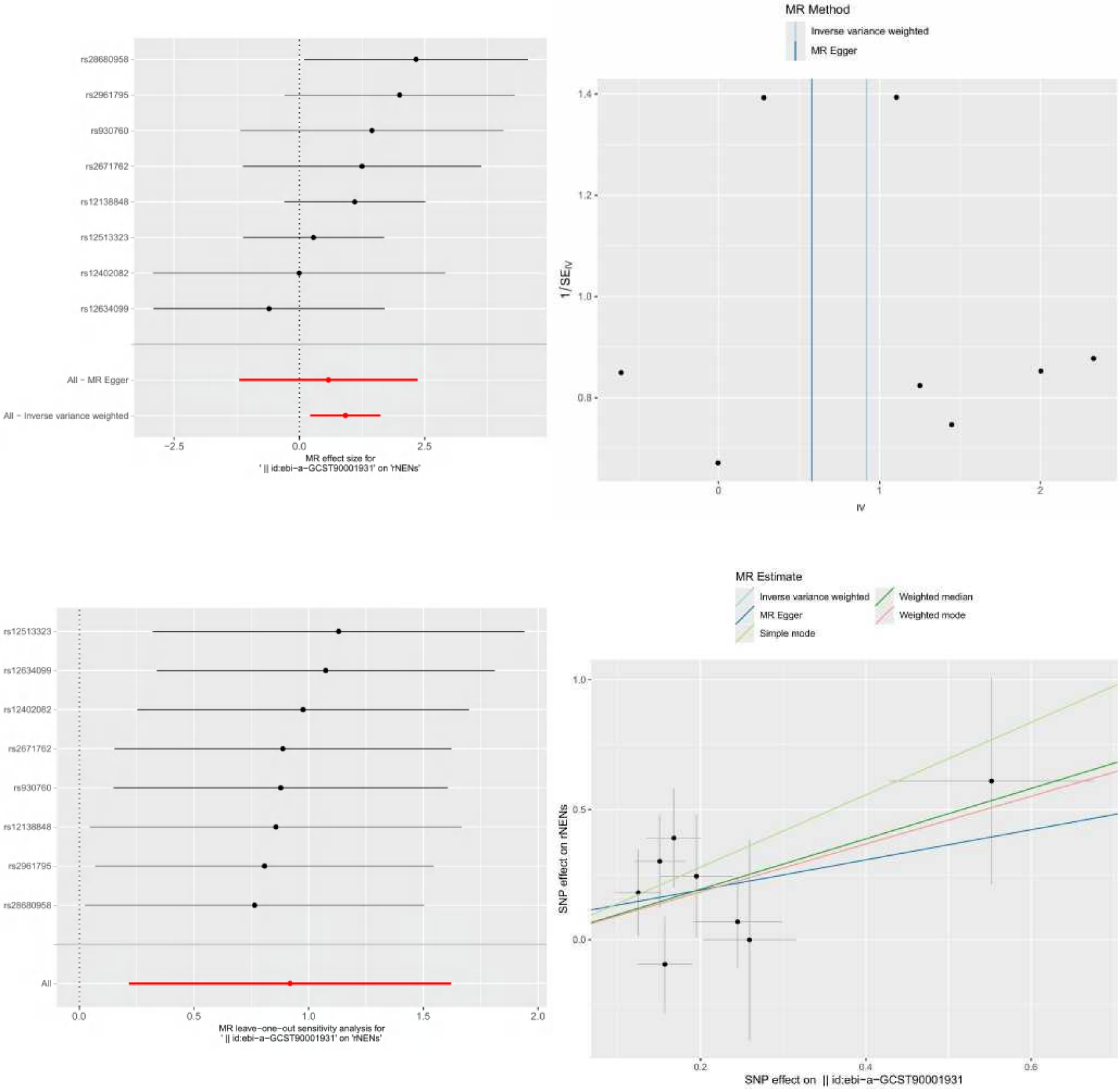

rs28680958
rs2961795
rs930760
rs2671762
rs12138848
rs12513323
rs12402082
rs12634099
All − MR Egger
All − Inverse variance weighted
-2.5
0.0
2.5
MR effect size for
' || id:ebi−a−GCST90001931' on 'rNENs'
MR Method
Inverse variance weighted
MR Egger
1.4
1.2
1.0
0.8
1/SE
IV
0
1
2
IV
rs12513323
rs12634099
rs12402082
rs2671762
rs930760
rs12138848
rs2961795
rs28680958
All
0.0
0.5
1.0
1.5
2.0
MR leave−one−out sensitivity analysis for
' || id:ebi−a−GCST90001931' on 'rNENs'
MR Estimate
Inverse variance weighted
MR Egger
Simple mode
Weighted median
Weighted mode
1.0
0.5
0.0
SNP effect on rNENs
0.2
0.4
0.6
SNP effect on || id:ebi−a−GCST90001931

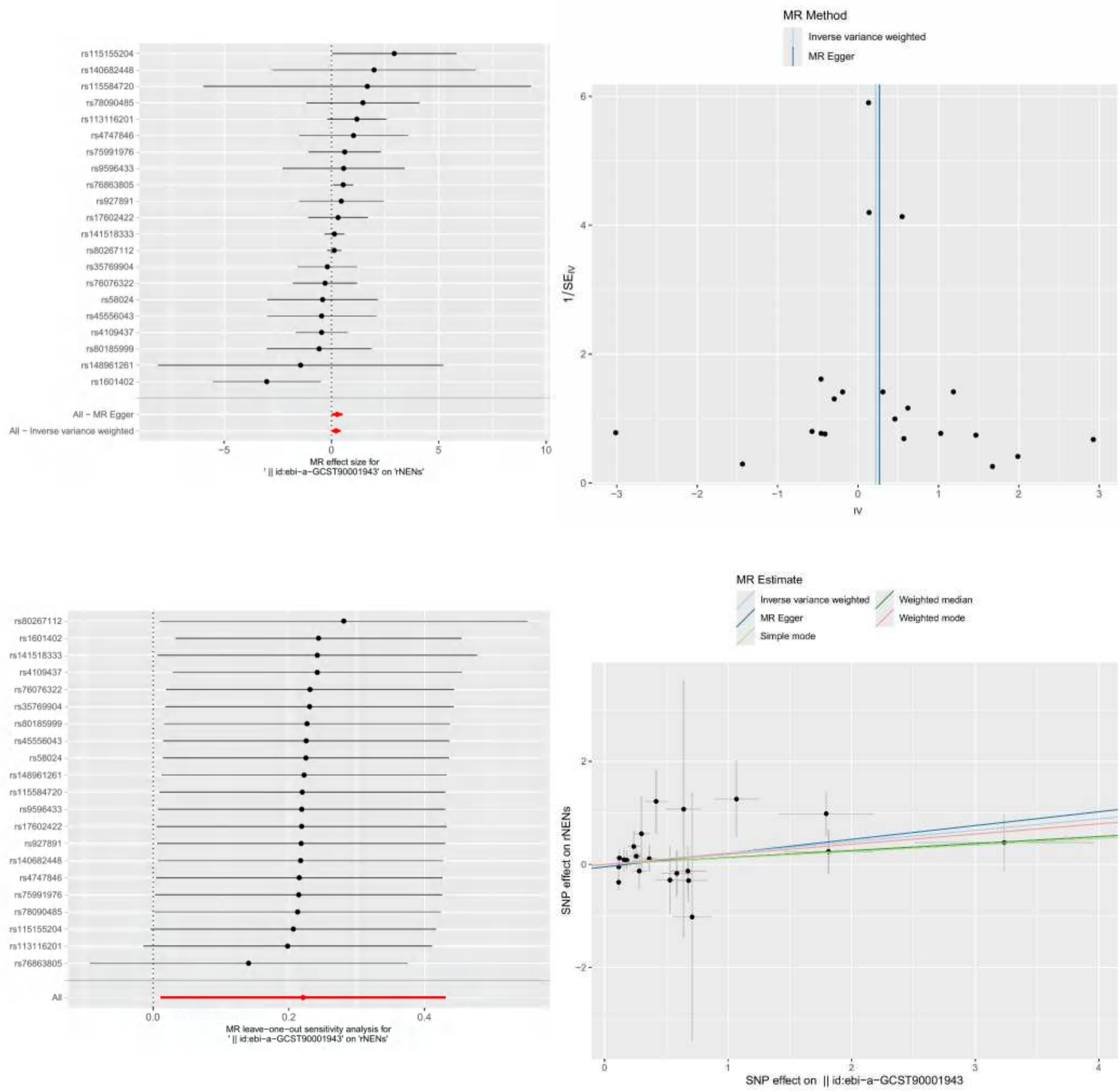

rs115155204
rs140682448
rs115584720
rs78090485
rs113116201
rs4747846
rs75991976
rs9596433
rs76863805
rs927891
rs17602422
rs141518333
rs80267112
rs35769904
rs76076322
rs58024
rs45556043
rs4109437
rs80185999
rs148961261
rs1601402
All - MR Egger
All - Inverse variance weighted
-5
0
5
10
MR effect size for
' || id:ebi-a-GCST90001943' on 'rNENs'
MR Method
Inverse variance weighted
MR Egger
$1/SE_{IV}$
IV
rs80267112
rs1601402
rs141518333
rs4109437
rs76076322
rs35769904
rs80185999
rs45556043
rs58024
rs148961261
rs115584720
rs9596433
rs17602422
rs927891
rs140682448
rs4747846
rs75991976
rs78090485
rs115155204
rs113116201
rs76863805
All
0.0
0.2
0.4
MR leave-one-out sensitivity analysis for
' || id:ebi-a-GCST90001943' on 'rNENs'
MR Estimate
Inverse variance weighted
MR Egger
Simple mode
Weighted median
Weighted mode
SNP effect on rNENs
SNP effect on || id:ebi-a-GCST90001943

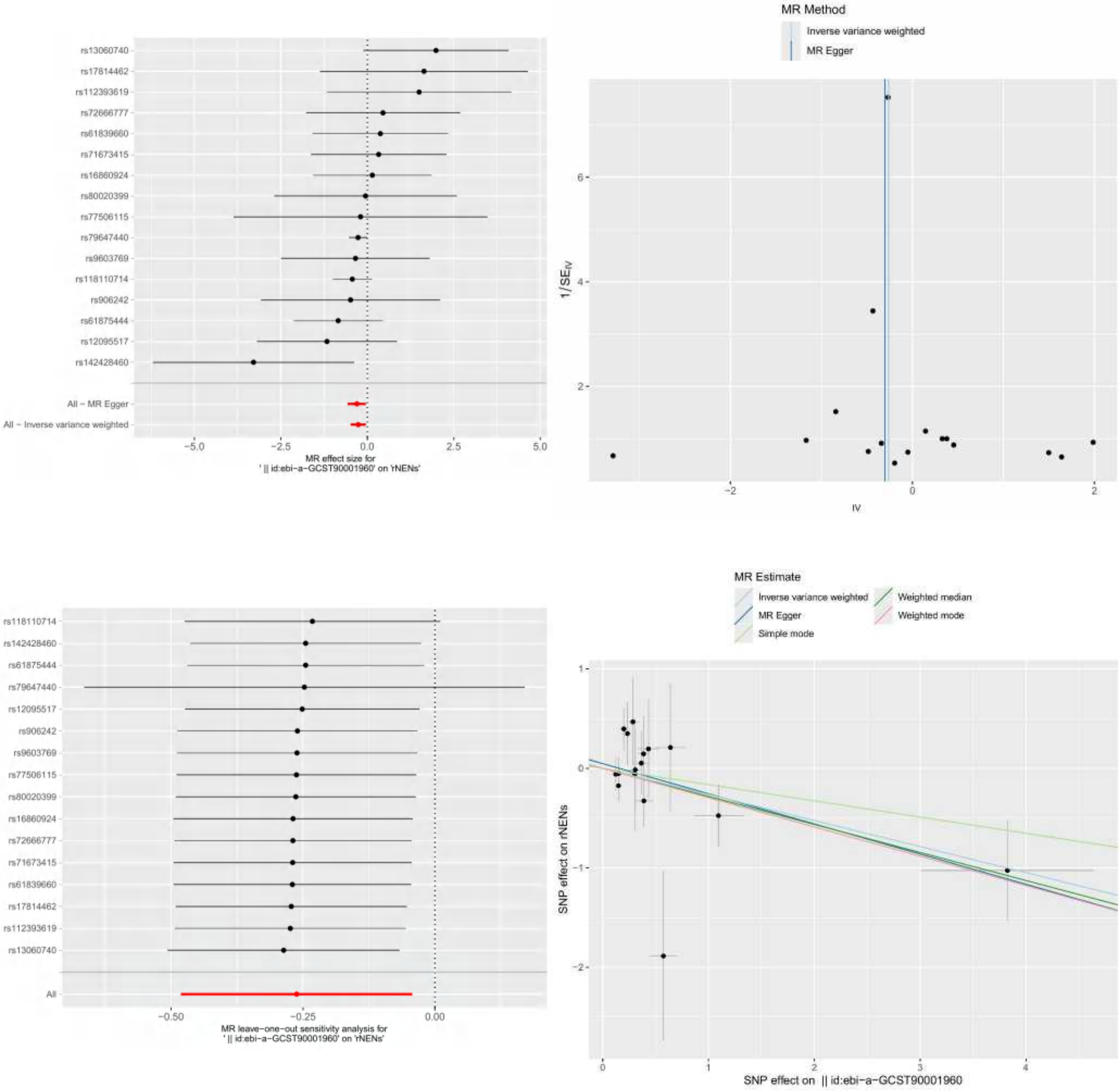

rs13060740
rs17814462
rs112393619
rs72666777
rs61839660
rs71673415
rs16860924
rs80020399
rs77506115
rs79647440
rs9603769
rs118110714
rs906242
rs61875444
rs12095517
rs142428460
All − MR Egger
All − Inverse variance weighted
−5.0
−2.5
0.0
2.5
5.0
MR effect size for
' || id:ebi−a−GCST90001960' on 'rNENs'
MR Method
Inverse variance weighted
MR Egger
1/SE_IV
IV
MR leave−one−out sensitivity analysis for
' || id:ebi−a−GCST90001960' on 'rNENs'
All
−0.50
−0.25
0.00
MR Estimate
Inverse variance weighted
MR Egger
Simple mode
Weighted median
Weighted mode
SNP effect on rNENs
SNP effect on || id:ebi−a−GCST90001960

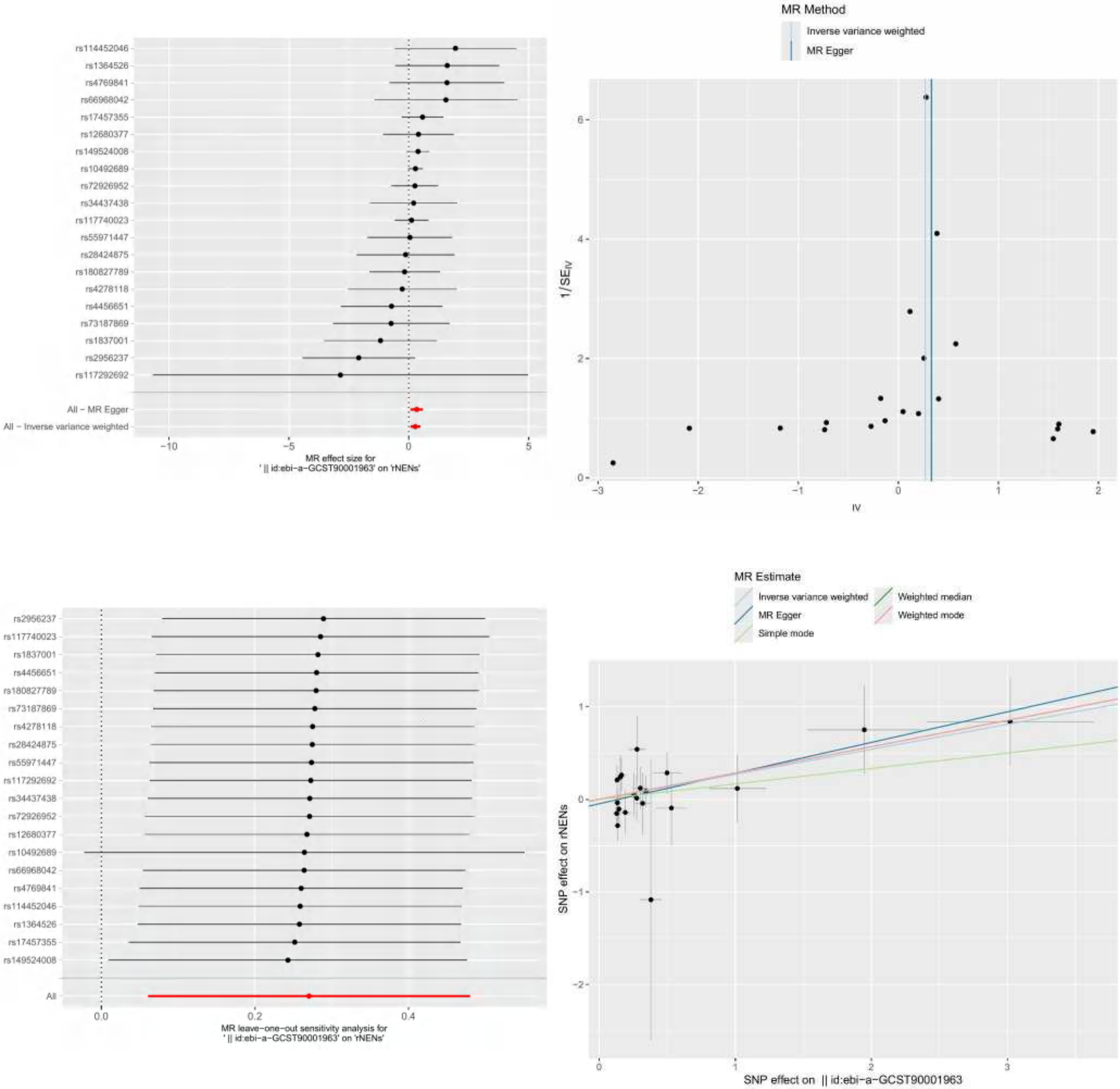

rs114452046
rs1364526
rs4769841
rs66968042
rs17457355
rs12680377
rs149524008
rs10492689
rs72926952
rs34437438
rs117740023
rs55971447
rs28424875
rs180827789
rs4278118
rs4456651
rs73187869
rs1837001
rs2956237
rs117292692
All − MR Egger
All − Inverse variance weighted
−10
−5
0
5
MR effect size for
' || id:ebi−a−GCST90001963' on 'rNENs'
MR Method
Inverse variance weighted
MR Egger
1/SE$_{IV}$
IV
rs2956237
rs117740023
rs1837001
rs4456651
rs180827789
rs73187869
rs4278118
rs28424875
rs55971447
rs117292692
rs34437438
rs72926952
rs12680377
rs10492689
rs66968042
rs4769841
rs114452046
rs1364526
rs17457355
rs149524008
All
0.0
0.2
0.4
MR leave−one−out sensitivity analysis for
' || id:ebi−a−GCST90001963' on 'rNENs'
MR Estimate
Inverse variance weighted
MR Egger
Simple mode
Weighted median
Weighted mode
SNP effect on rNENs
SNP effect on || id:ebi−a−GCST90001963

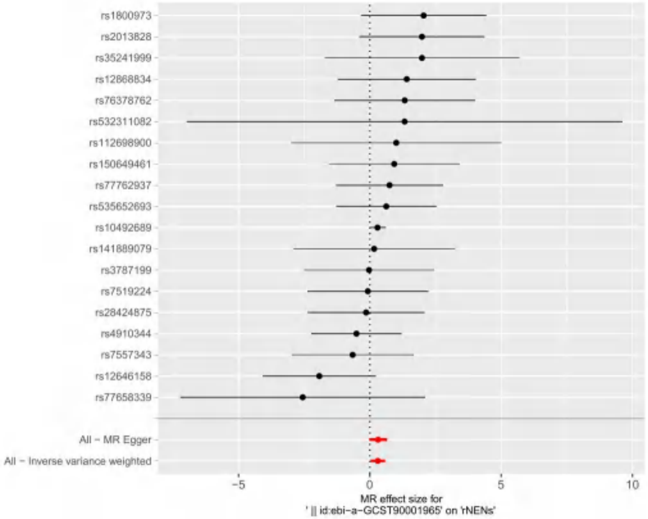
rs1800973
rs2013828
rs35241999
rs12868834
rs76378762
rs532311082
rs112698900
rs150649461
rs77762937
rs535652693
rs10492689
rs141889079
rs3787199
rs7519224
rs28424875
rs4910344
rs7557343
rs12646158
rs77658339
All − MR Egger
All − Inverse variance weighted
-5
0
5
10
MR effect size for
' || id:ebi−a−GCST90001965' on 'rNENs'

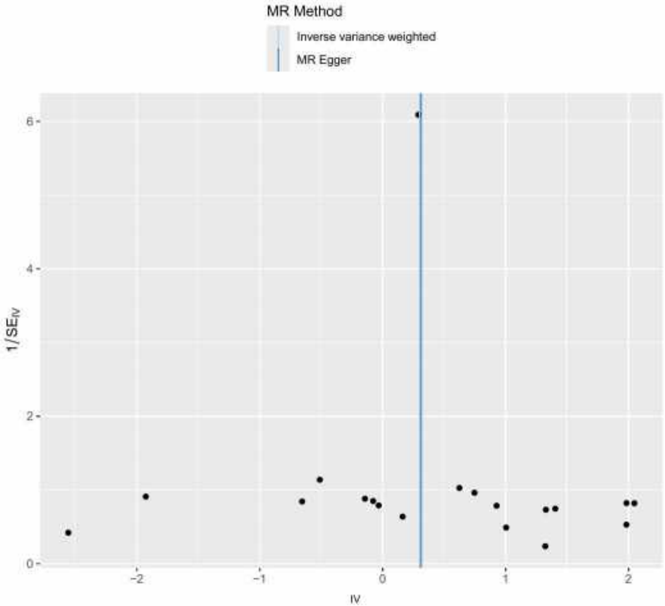
MR Method
Inverse variance weighted
MR Egger
1/SE$_{IV}$
IV

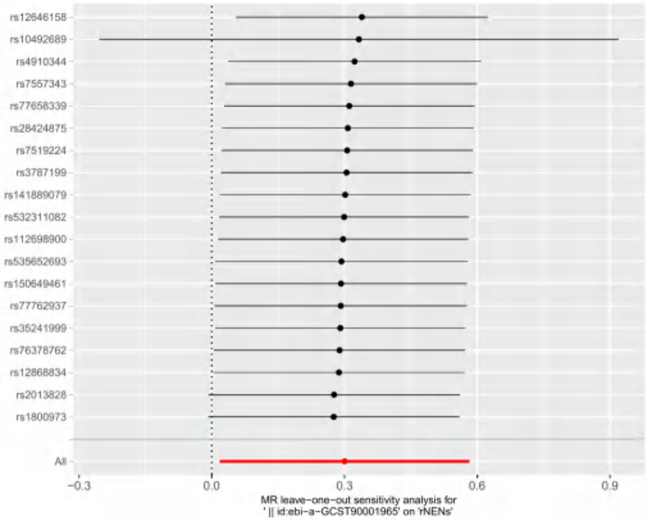
rs12646158
rs10492689
rs4910344
rs7557343
rs77658339
rs28424875
rs7519224
rs3787199
rs141889079
rs532311082
rs112698900
rs535652693
rs150649461
rs77762937
rs35241999
rs76378762
rs12868834
rs2013828
rs1800973
All
-0.3
0.0
0.3
0.6
0.9
MR leave−one−out sensitivity analysis for
' || id:ebi−a−GCST90001965' on 'rNENs'

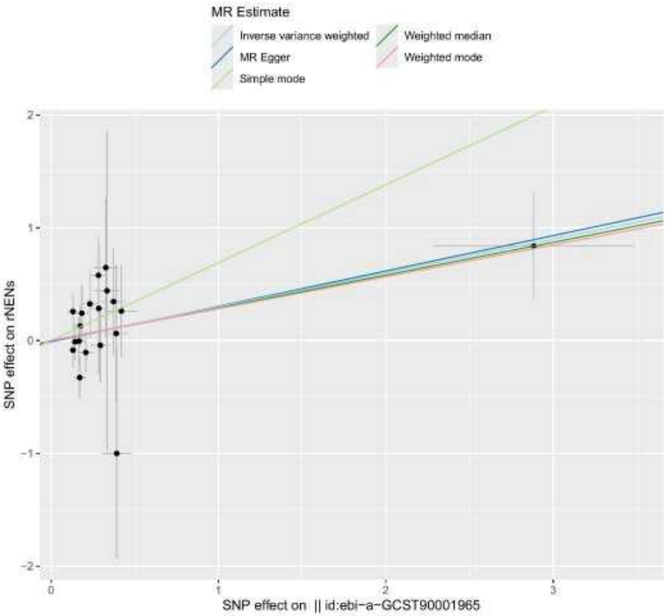
MR Estimate
Inverse variance weighted
MR Egger
Simple mode
Weighted median
Weighted mode
SNP effect on rNENs
SNP effect on || id:ebi−a−GCST90001965

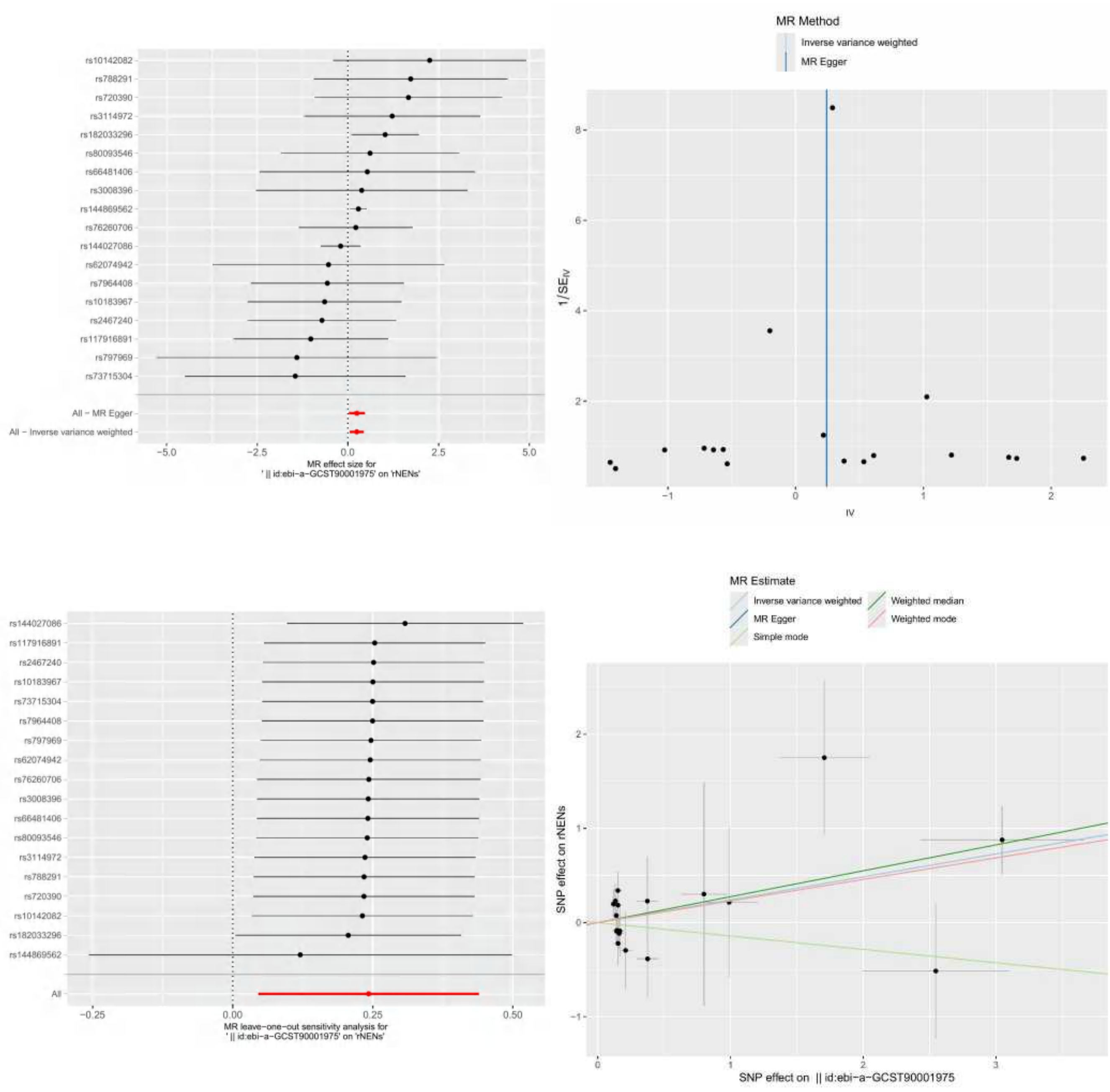
rs10142082
rs788291
rs720390
rs3114972
rs182033296
rs80093546
rs66481406
rs3008396
rs144869562
rs76260706
rs144027086
rs62074942
rs7964408
rs10183967
rs2467240
rs117916891
rs797969
rs73715304
All − MR Egger
All − Inverse variance weighted
−5.0
−2.5
0.0
2.5
5.0
MR effect size for
' || id:ebi−a−GCST90001975' on 'rNENs'
MR Method
Inverse variance weighted
MR Egger
1/SE$_{IV}$
IV
8
6
4
2
−1
0
1
2
rs144027086
rs117916891
rs2467240
rs10183967
rs73715304
rs7964408
rs797969
rs62074942
rs76260706
rs3008396
rs66481406
rs80093546
rs3114972
rs788291
rs720390
rs10142082
rs182033296
rs144869562
All
−0.25
0.00
0.25
0.50
MR leave−one−out sensitivity analysis for
' || id:ebi−a−GCST90001975' on 'rNENs'
MR Estimate
Inverse variance weighted
MR Egger
Simple mode
Weighted median
Weighted mode
SNP effect on rNENs
SNP effect on || id:ebi−a−GCST90001975
2
1
0
−1
0
1
2
3

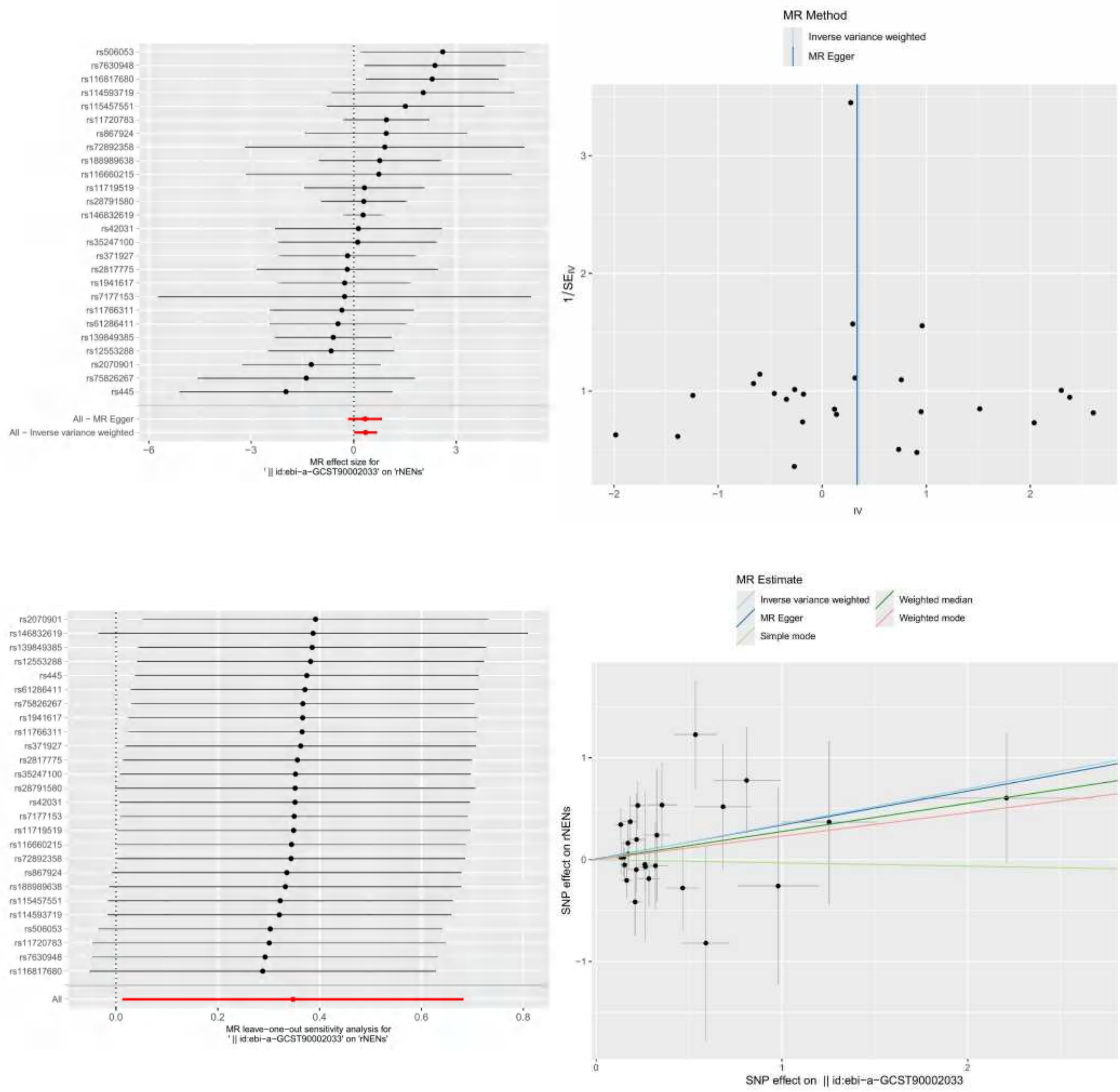

rs506053
rs7630948
rs116817680
rs114593719
rs115457551
rs11720783
rs867924
rs72892358
rs188989638
rs116660215
rs11719519
rs28791580
rs146832619
rs42031
rs35247100
rs371927
rs2817775
rs1941617
rs7177153
rs11766311
rs61286411
rs139849385
rs12553288
rs2070901
rs75826267
rs445
All - MR Egger
All - Inverse variance weighted
-6
-3
0
3
MR effect size for
' || id:ebi-a-GCST90002033' on 'rNENs'
MR Method
Inverse variance weighted
MR Egger
1/SE$_{IV}$
3
2
1
-2
-1
0
1
2
IV
rs2070901
rs146832619
rs139849385
rs12553288
rs445
rs61286411
rs75826267
rs1941617
rs11766311
rs371927
rs2817775
rs35247100
rs28791580
rs42031
rs7177153
rs11719519
rs116660215
rs72892358
rs867924
rs188989638
rs115457551
rs114593719
rs506053
rs11720783
rs7630948
rs116817680
All
0.0
0.2
0.4
0.6
0.8
MR leave-one-out sensitivity analysis for
' || id:ebi-a-GCST90002033' on 'rNENs'
MR Estimate
Inverse variance weighted
MR Egger
Simple mode
Weighted median
Weighted mode
SNP effect on rNENs
1
0
-1
0
1
2
SNP effect on || id:ebi-a-GCST90002033

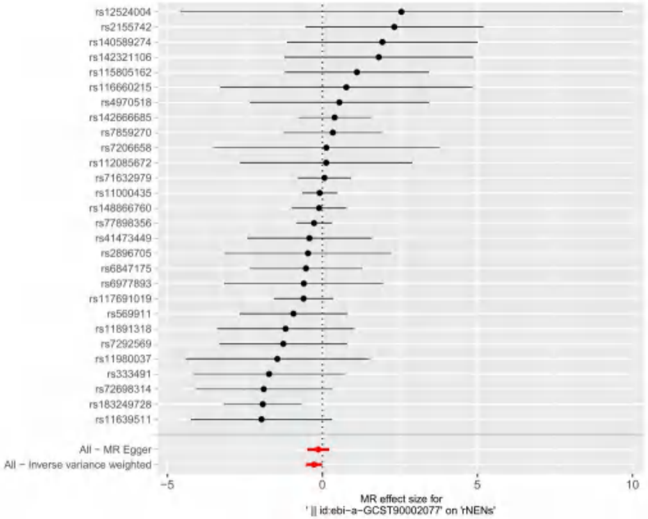
rs12524004
rs2155742
rs140589274
rs142321106
rs115805162
rs116660215
rs4970518
rs142666685
rs7859270
rs7206658
rs112085672
rs71632979
rs11000435
rs148866760
rs77898356
rs41473449
rs2896705
rs6847175
rs6977893
rs117691019
rs569911
rs11891318
rs7292569
rs11980037
rs333491
rs72698314
rs183249728
rs11639511
All – MR Egger
All – Inverse variance weighted
−5
0
5
10
MR effect size for
' || id:ebi−a−GCST90002077' on 'rNENs'

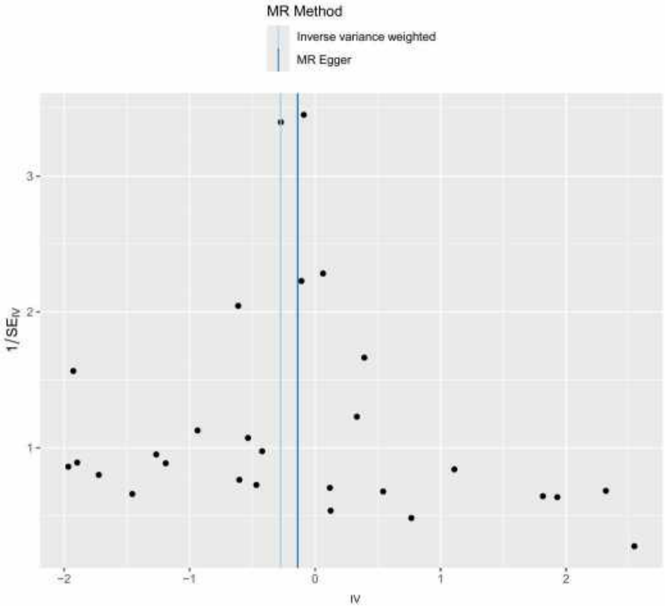
MR Method
Inverse variance weighted
MR Egger
1/SE_{IV}
1
2
3
−2
−1
0
1
2
IV

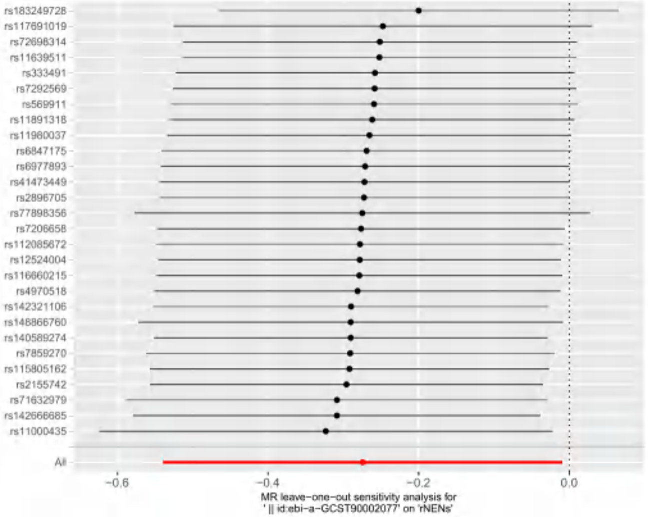
rs183249728
rs117691019
rs72698314
rs11639511
rs333491
rs7292569
rs569911
rs11891318
rs11980037
rs6847175
rs6977893
rs41473449
rs2896705
rs77898356
rs7206658
rs112085672
rs12524004
rs116660215
rs4970518
rs142321106
rs148866760
rs140589274
rs7859270
rs115805162
rs2155742
rs71632979
rs142666685
rs11000435
All
−0.6
−0.4
−0.2
0.0
MR leave−one−out sensitivity analysis for
' || id:ebi−a−GCST90002077' on 'rNENs'

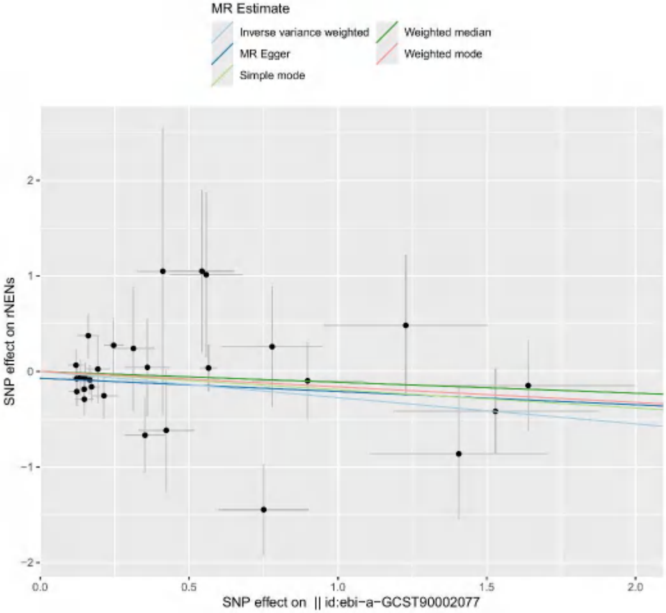
MR Estimate
Inverse variance weighted
MR Egger
Simple mode
Weighted median
Weighted mode
SNP effect on rNENs
−2
−1
0
1
2
0.0
0.5
1.0
1.5
2.0
SNP effect on || id:ebi−a−GCST90002077

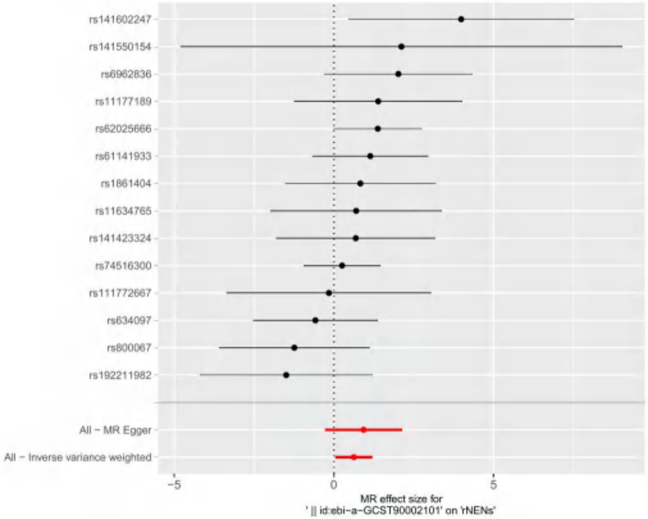
rs141602247
rs141550154
rs6962836
rs11177189
rs62025666
rs61141933
rs1861404
rs11634765
rs141423324
rs74516300
rs111772667
rs634097
rs800067
rs192211982
All − MR Egger
All − Inverse variance weighted
−5
0
5
MR effect size for
' || id:ebi−a−GCST90002101' on 'rNENs'

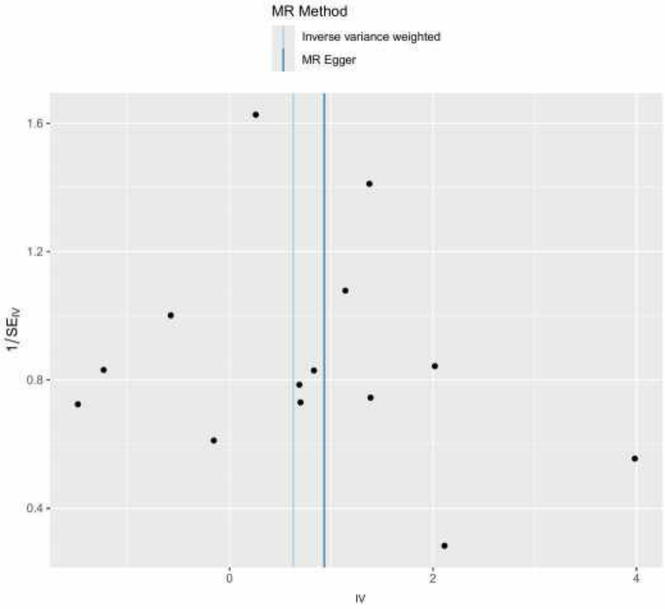
MR Method
Inverse variance weighted
MR Egger
1.6
1.2
0.8
0.4
1/SE_IV
0
2
4
IV

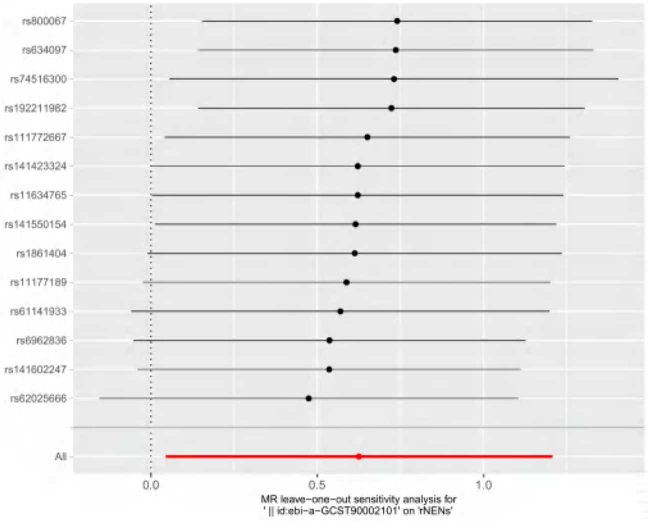
rs800067
rs634097
rs74516300
rs192211982
rs111772667
rs141423324
rs11634765
rs141550154
rs1861404
rs11177189
rs61141933
rs6962836
rs141602247
rs62025666
All
0.0
0.5
1.0
MR leave−one−out sensitivity analysis for
' || id:ebi−a−GCST90002101' on 'rNENs'

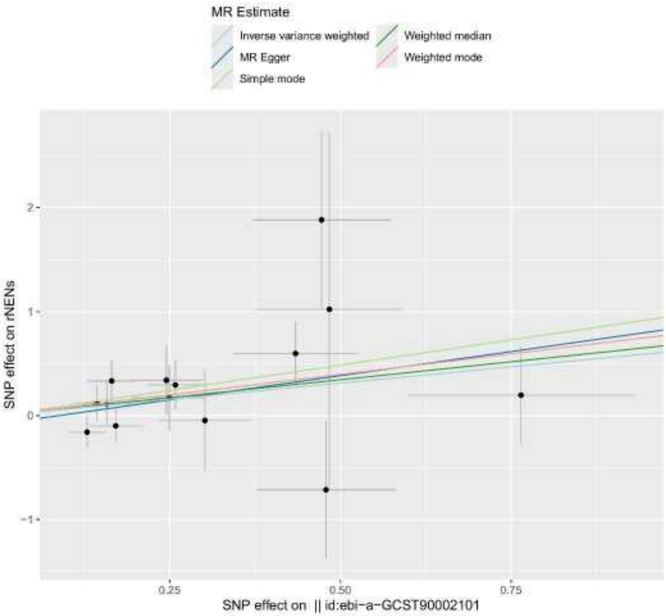
MR Estimate
Inverse variance weighted
MR Egger
Simple mode
Weighted median
Weighted mode
2
1
0
−1
SNP effect on rNENs
0.25
0.50
0.75
SNP effect on || id:ebi−a−GCST90002101

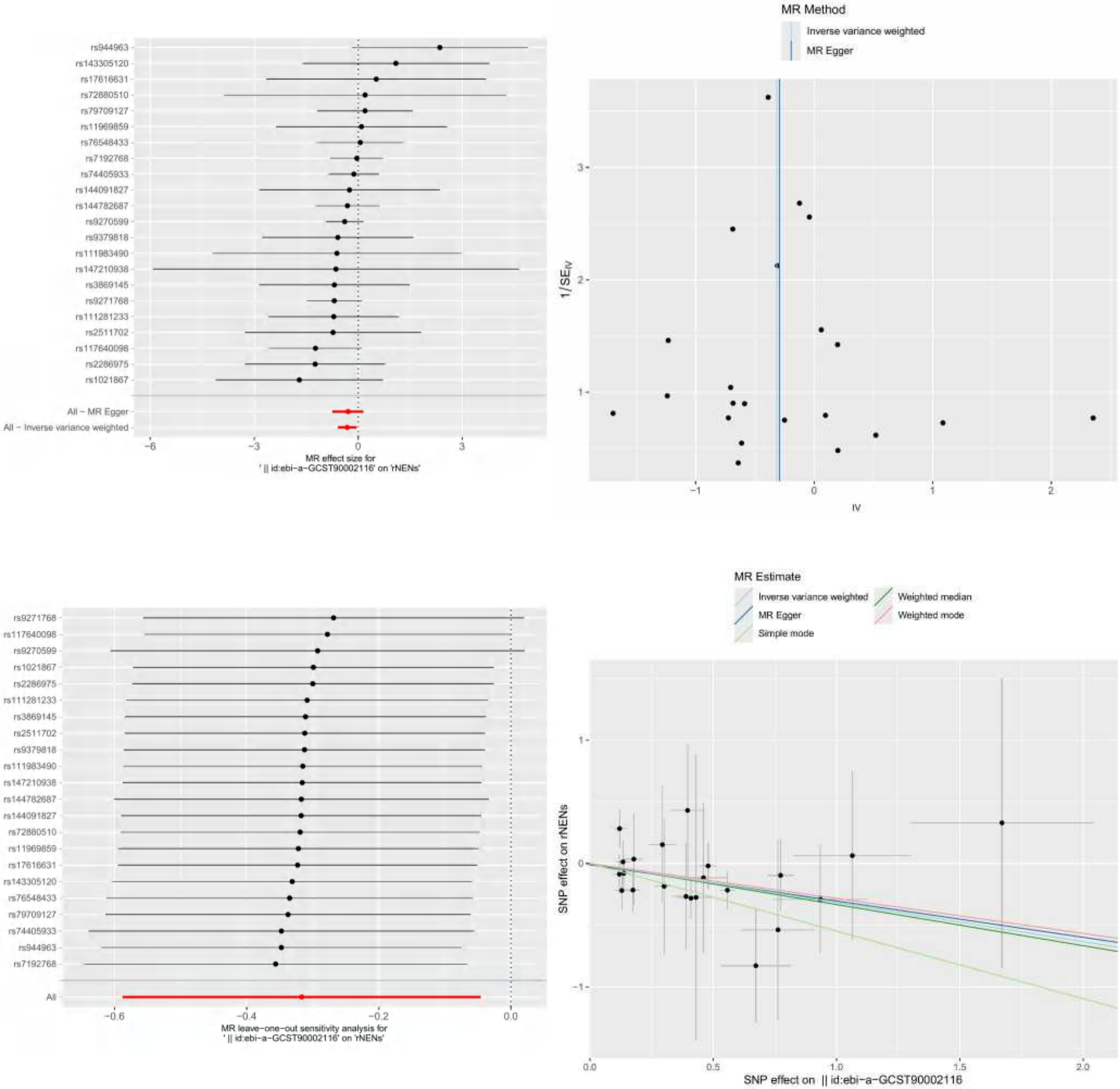

rs944963
rs143305120
rs17616631
rs72880510
rs79709127
rs11969859
rs76548433
rs7192768
rs74405933
rs144091827
rs144782687
rs9270599
rs9379818
rs111983490
rs147210938
rs3869145
rs9271768
rs111281233
rs2511702
rs117640098
rs2286975
rs1021867
All − MR Egger
All − Inverse variance weighted
MR effect size for
' || id:ebi−a−GCST90002116' on 'rNENs'
MR Method
Inverse variance weighted
MR Egger
1/SE_IV
IV
MR leave−one−out sensitivity analysis for
' || id:ebi−a−GCST90002116' on 'rNENs'
All
MR Estimate
Inverse variance weighted
MR Egger
Simple mode
Weighted median
Weighted mode
SNP effect on rNENs
SNP effect on || id:ebi−a−GCST90002116

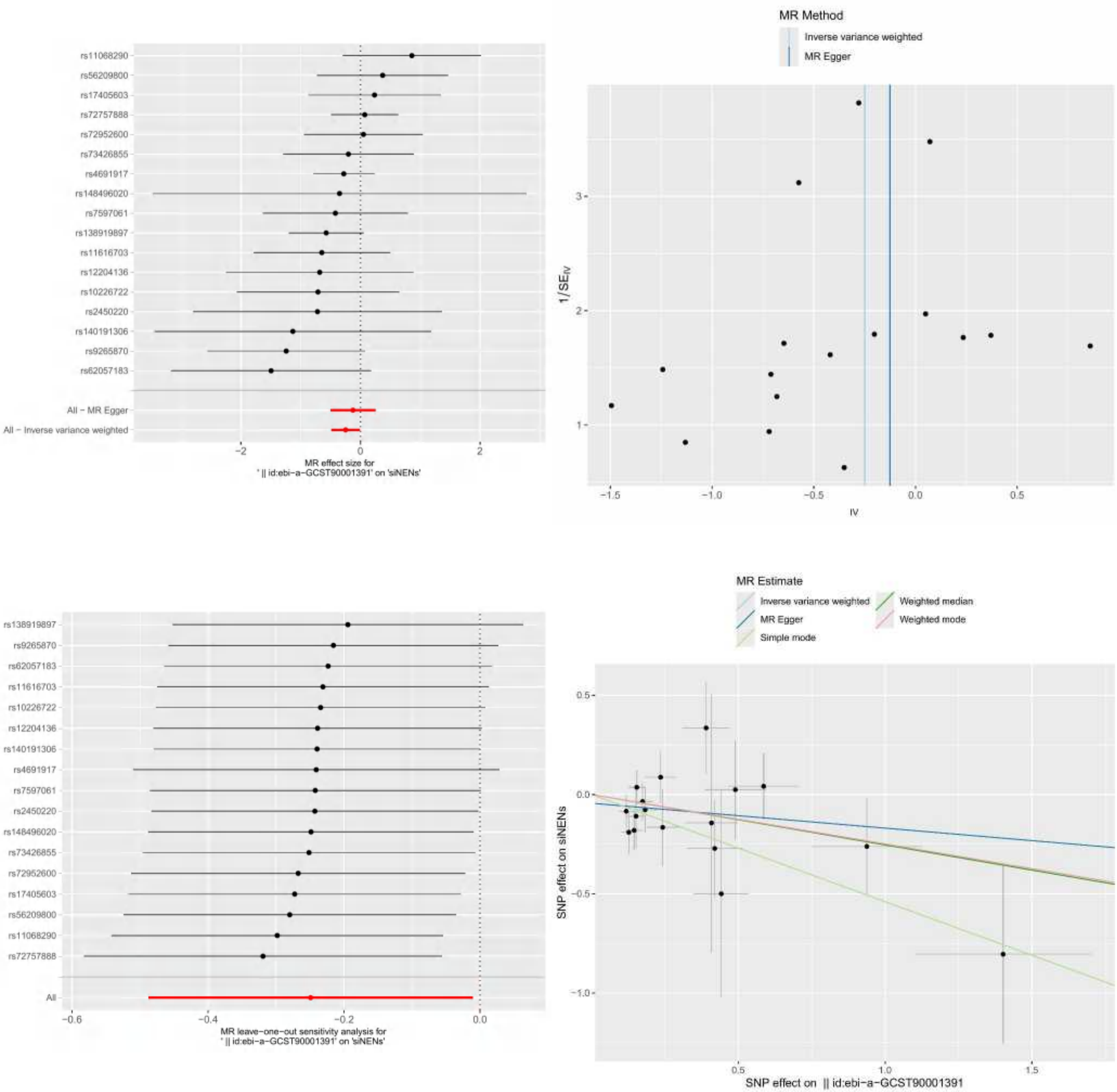

rs11068290
rs56209800
rs17405603
rs72757888
rs72952600
rs73426855
rs4691917
rs148496020
rs7597061
rs138919897
rs11616703
rs12204136
rs10226722
rs2450220
rs140191306
rs9265870
rs62057183
All − MR Egger
All − Inverse variance weighted
−2
0
2
MR effect size for
' || id:ebi−a−GCST90001391' on 'siNENs'
MR Method
Inverse variance weighted
MR Egger
1/SE$_{IV}$
3
2
1
−1.5
−1.0
−0.5
0.0
0.5
IV
rs138919897
rs9265870
rs62057183
rs11616703
rs10226722
rs12204136
rs140191306
rs4691917
rs7597061
rs2450220
rs148496020
rs73426855
rs72952600
rs17405603
rs56209800
rs11068290
rs72757888
All
−0.6
−0.4
−0.2
0.0
MR leave−one−out sensitivity analysis for
' || id:ebi−a−GCST90001391' on 'siNENs'
MR Estimate
Inverse variance weighted
MR Egger
Simple mode
Weighted median
Weighted mode
SNP effect on siNENs
0.5
0.0
−0.5
−1.0
0.5
1.0
1.5
SNP effect on || id:ebi−a−GCST90001391

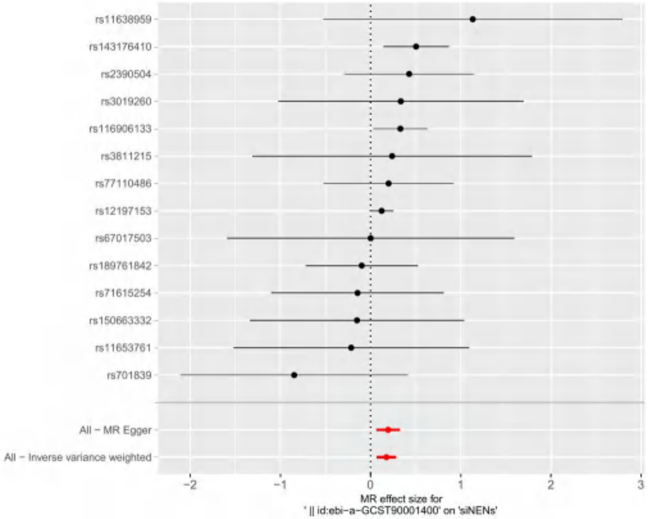
rs11638959
rs143176410
rs2390504
rs3019260
rs116906133
rs3811215
rs77110486
rs12197153
rs67017503
rs189761842
rs71615254
rs150663332
rs11653761
rs701839
All − MR Egger
All − Inverse variance weighted
−2 −1 0 1 2 3
MR effect size for
' || id:ebi−a−GCST90001400' on 'siNENs'

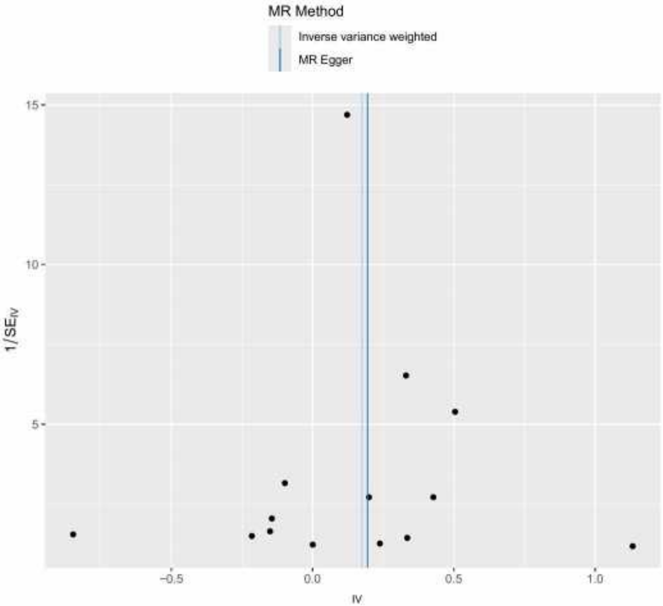
MR Method
Inverse variance weighted
MR Egger
15
10
5
1/SE_IV
−0.5 0.0 0.5 1.0
IV

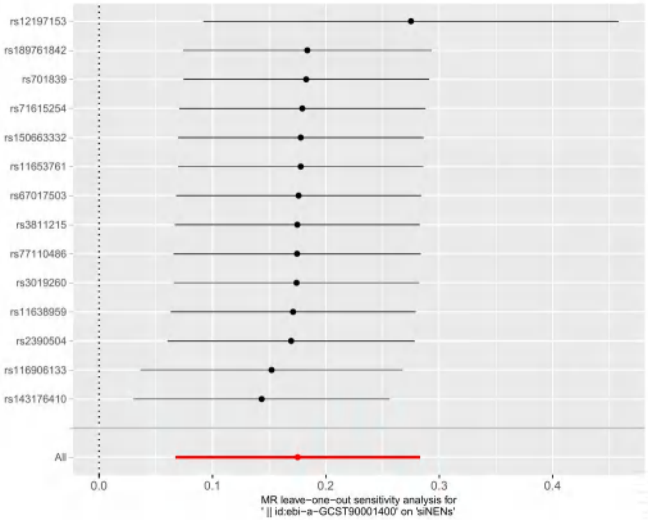
rs12197153
rs189761842
rs701839
rs71615254
rs150663332
rs11653761
rs67017503
rs3811215
rs77110486
rs3019260
rs11638959
rs2390504
rs116906133
rs143176410
All
0.0 0.1 0.2 0.3 0.4
MR leave−one−out sensitivity analysis for
' || id:ebi−a−GCST90001400' on 'siNENs'

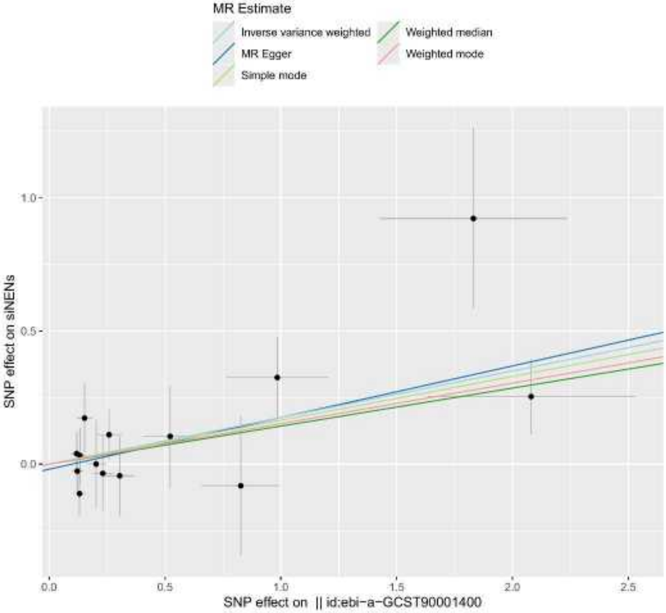
MR Estimate
Inverse variance weighted
MR Egger
Simple mode
Weighted median
Weighted mode
1.0
0.5
0.0
SNP effect on siNENs
0.0 0.5 1.0 1.5 2.0 2.5
SNP effect on || id:ebi−a−GCST90001400

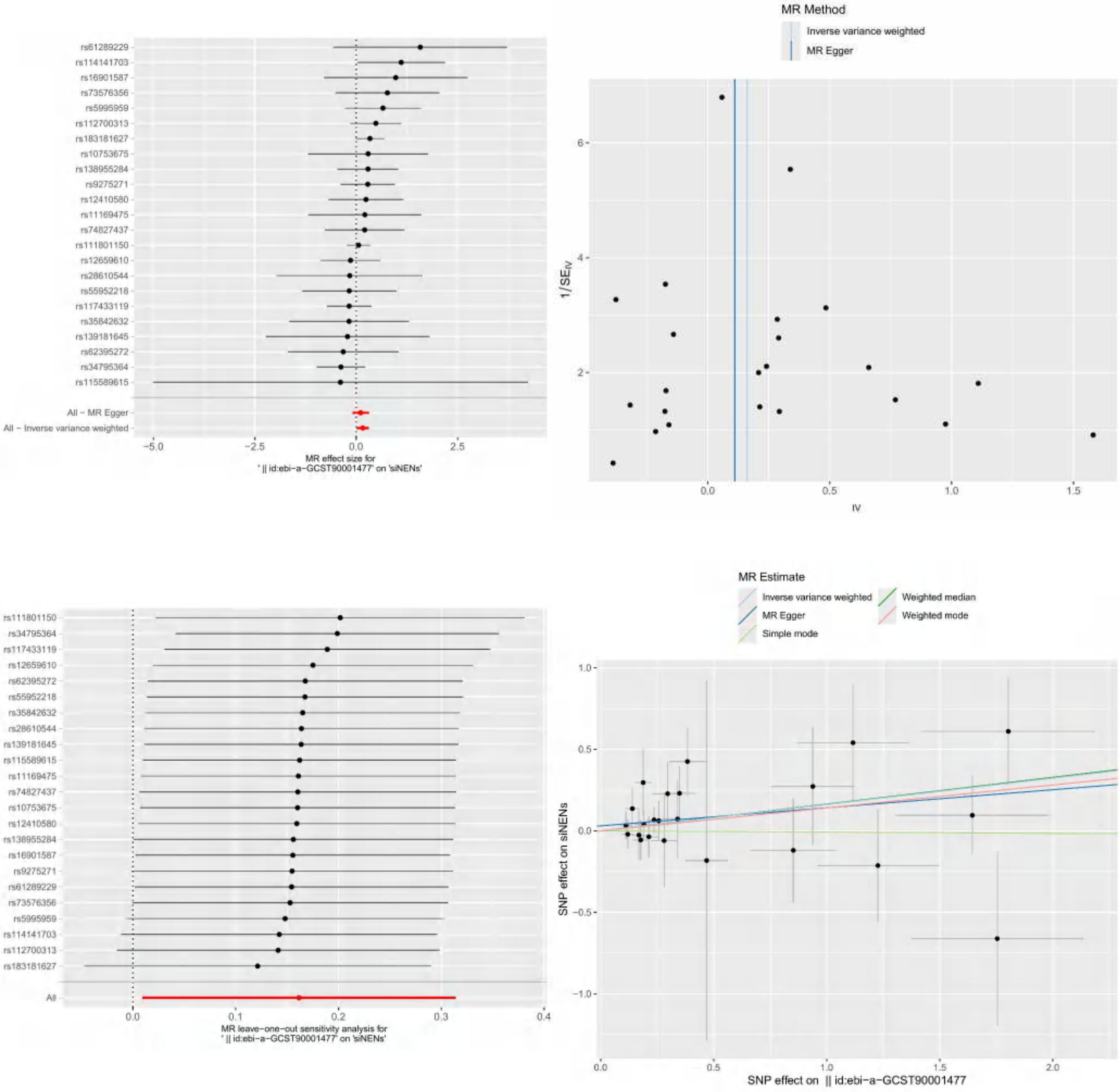
rs61289229
rs114141703
rs16901587
rs73576356
rs5995959
rs112700313
rs183181627
rs10753675
rs138955284
rs9275271
rs12410580
rs11169475
rs74827437
rs111801150
rs12659610
rs28610544
rs55952218
rs117433119
rs35842632
rs139181645
rs62396272
rs34795364
rs115589615
All − MR Egger
All − Inverse variance weighted
−5.0
−2.5
0.0
2.5
MR effect size for
' || id:ebi−a−GCST90001477' on 'siNENs'
MR Method
Inverse variance weighted
MR Egger
1/SE$_{IV}$
6
4
2
0.0
0.5
1.0
1.5
IV
rs111801150
rs34795364
rs117433119
rs12659610
rs62395272
rs55952218
rs35842632
rs28610544
rs139181645
rs115589615
rs11169475
rs74827437
rs10753675
rs12410580
rs138955284
rs16901587
rs9275271
rs61289229
rs73576356
rs5995959
rs114141703
rs112700313
rs183181627
All
0.0
0.1
0.2
0.3
0.4
MR leave−one−out sensitivity analysis for
' || id:ebi−a−GCST90001477' on 'siNENs'
MR Estimate
Inverse variance weighted
MR Egger
Simple mode
Weighted median
Weighted mode
1.0
0.5
0.0
−0.5
−1.0
SNP effect on siNENs
0.0
0.5
1.0
1.5
2.0
SNP effect on || id:ebi−a−GCST90001477

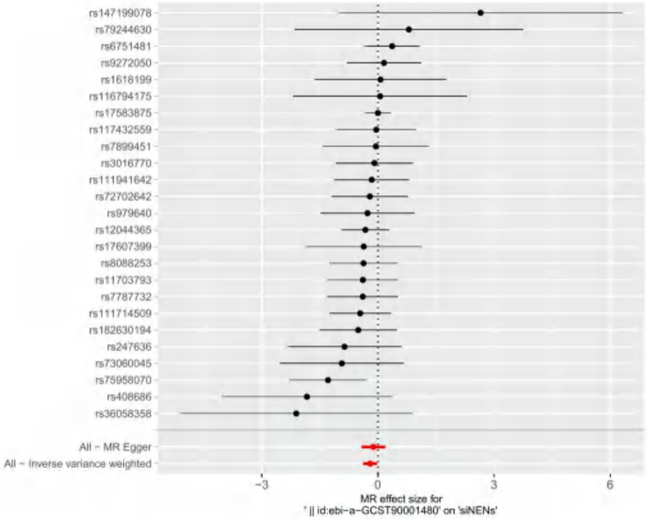
rs147199078
rs79244630
rs6751481
rs9272050
rs1618199
rs116794175
rs17583875
rs117432559
rs7899451
rs3016770
rs111941642
rs72702642
rs979640
rs12044365
rs17607399
rs8088253
rs11703793
rs7787732
rs111714509
rs182630194
rs247636
rs73060045
rs75958070
rs408686
rs36058358
All - MR Egger
All - Inverse variance weighted
-3
0
3
6
MR effect size for
' || id:ebi-a-GCST90001480' on 'siNENs'

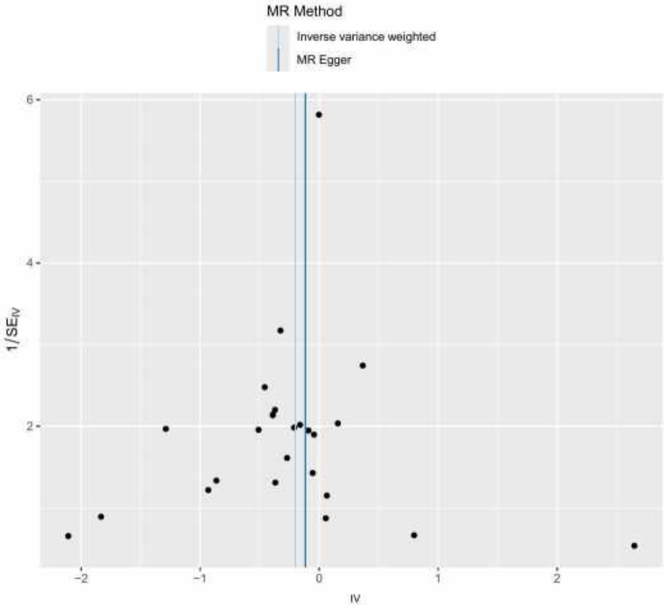
MR Method
Inverse variance weighted
MR Egger
6
4
2
1/SE_IV
-2
-1
0
1
2
IV

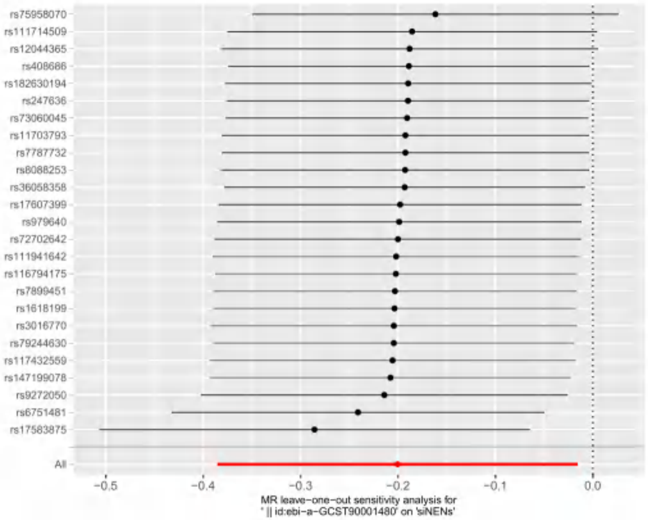
rs75958070
rs111714509
rs12044365
rs408686
rs182630194
rs247636
rs73060045
rs11703793
rs7787732
rs8088253
rs36058358
rs17607399
rs979640
rs72702642
rs111941642
rs116794175
rs7899451
rs1618199
rs3016770
rs79244630
rs117432559
rs147199078
rs9272050
rs6751481
rs17583875
All
-0.5
-0.4
-0.3
-0.2
-0.1
0.0
MR leave-one-out sensitivity analysis for
' || id:ebi-a-GCST90001480' on 'siNENs'

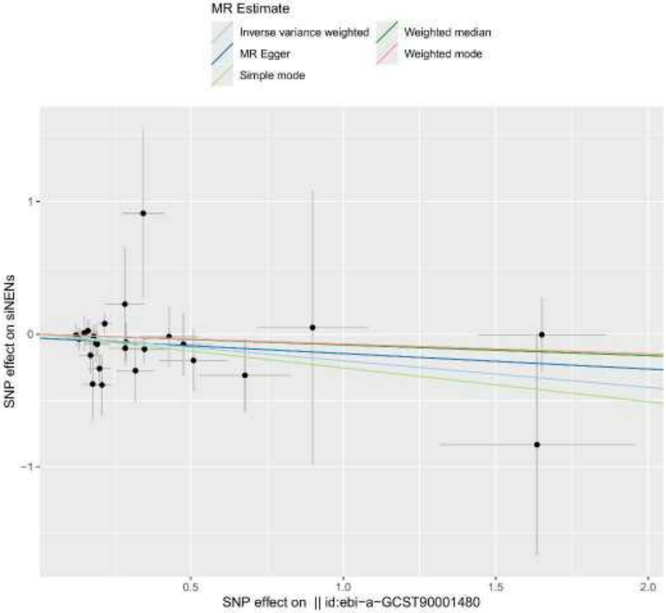
MR Estimate
Inverse variance weighted
MR Egger
Simple mode
Weighted median
Weighted mode
1
0
-1
SNP effect on siNENs
0.5
1.0
1.5
2.0
SNP effect on || id:ebi-a-GCST90001480

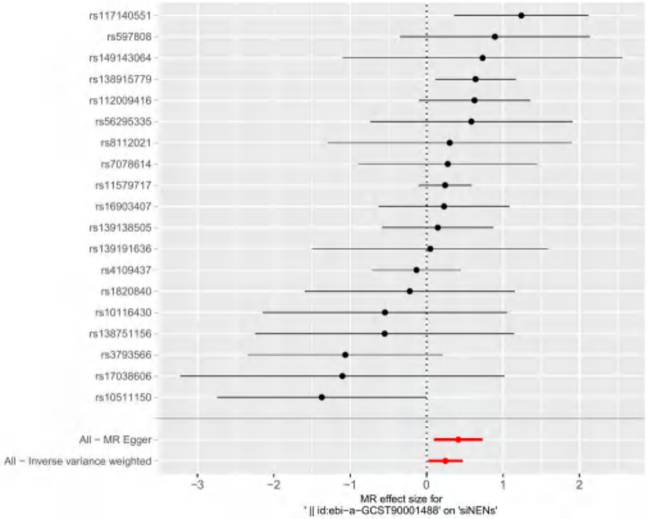
rs117140551
rs597808
rs149143064
rs138915779
rs112009416
rs56295335
rs8112021
rs7078614
rs11579717
rs16903407
rs139138505
rs139191636
rs4109437
rs1820840
rs10116430
rs138751156
rs3793566
rs17038606
rs10511150
All − MR Egger
All − Inverse variance weighted
−3
−2
−1
0
1
2
MR effect size for
' || id:ebi−a−GCST90001488' on 'siNENs'

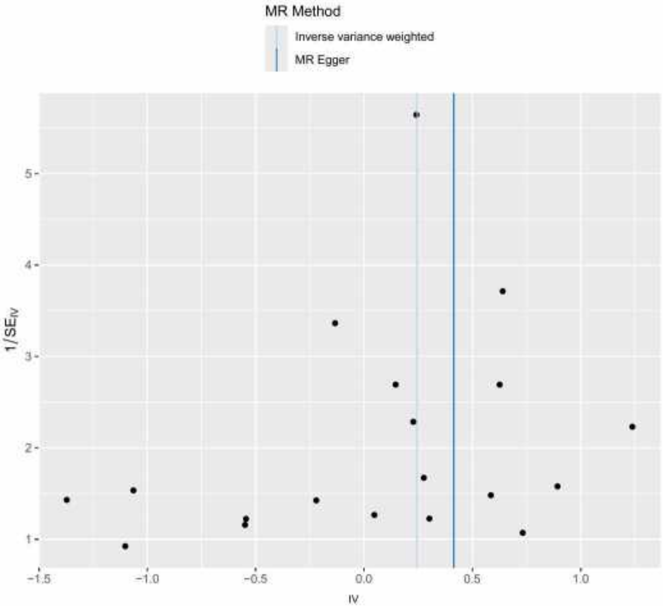
MR Method
Inverse variance weighted
MR Egger
1/SE$_{IV}$
5
4
3
2
1
−1.5
−1.0
−0.5
0.0
0.5
1.0
IV

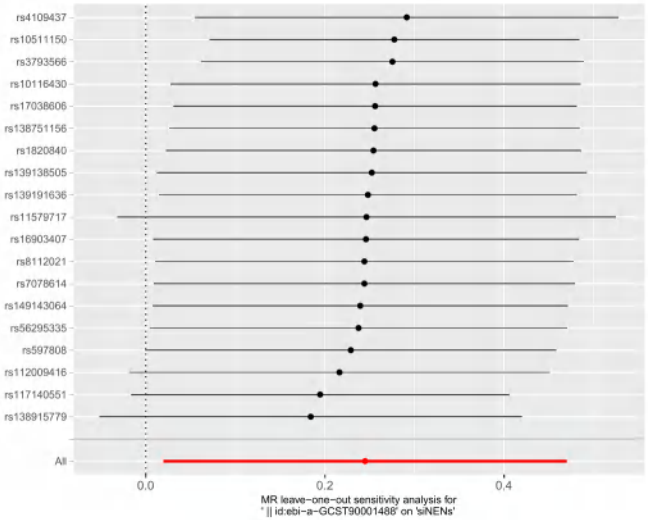
rs4109437
rs10511150
rs3793566
rs10116430
rs17038606
rs138751156
rs1820840
rs139138505
rs139191636
rs11579717
rs16903407
rs8112021
rs7078614
rs149143064
rs56295335
rs597808
rs112009416
rs117140551
rs138915779
All
0.0
0.2
0.4
MR leave−one−out sensitivity analysis for
' || id:ebi−a−GCST90001488' on 'siNENs'

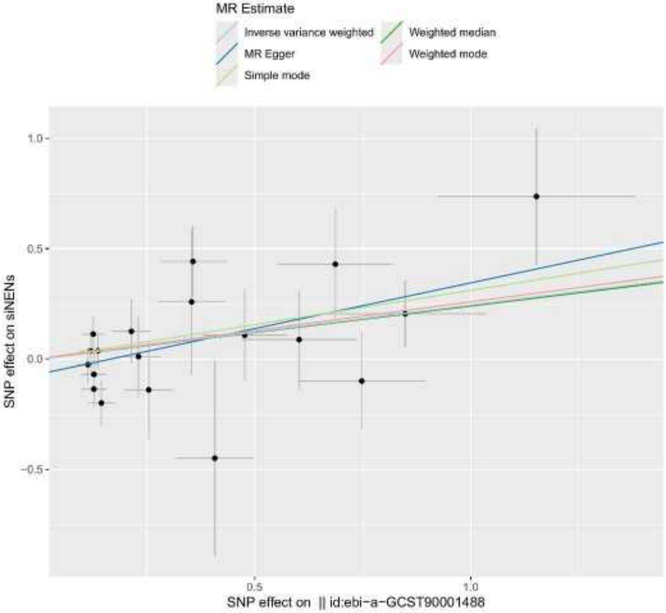
MR Estimate
Inverse variance weighted
MR Egger
Simple mode
Weighted median
Weighted mode
1.0
0.5
0.0
−0.5
SNP effect on siNENs
0.5
1.0
SNP effect on || id:ebi−a−GCST90001488

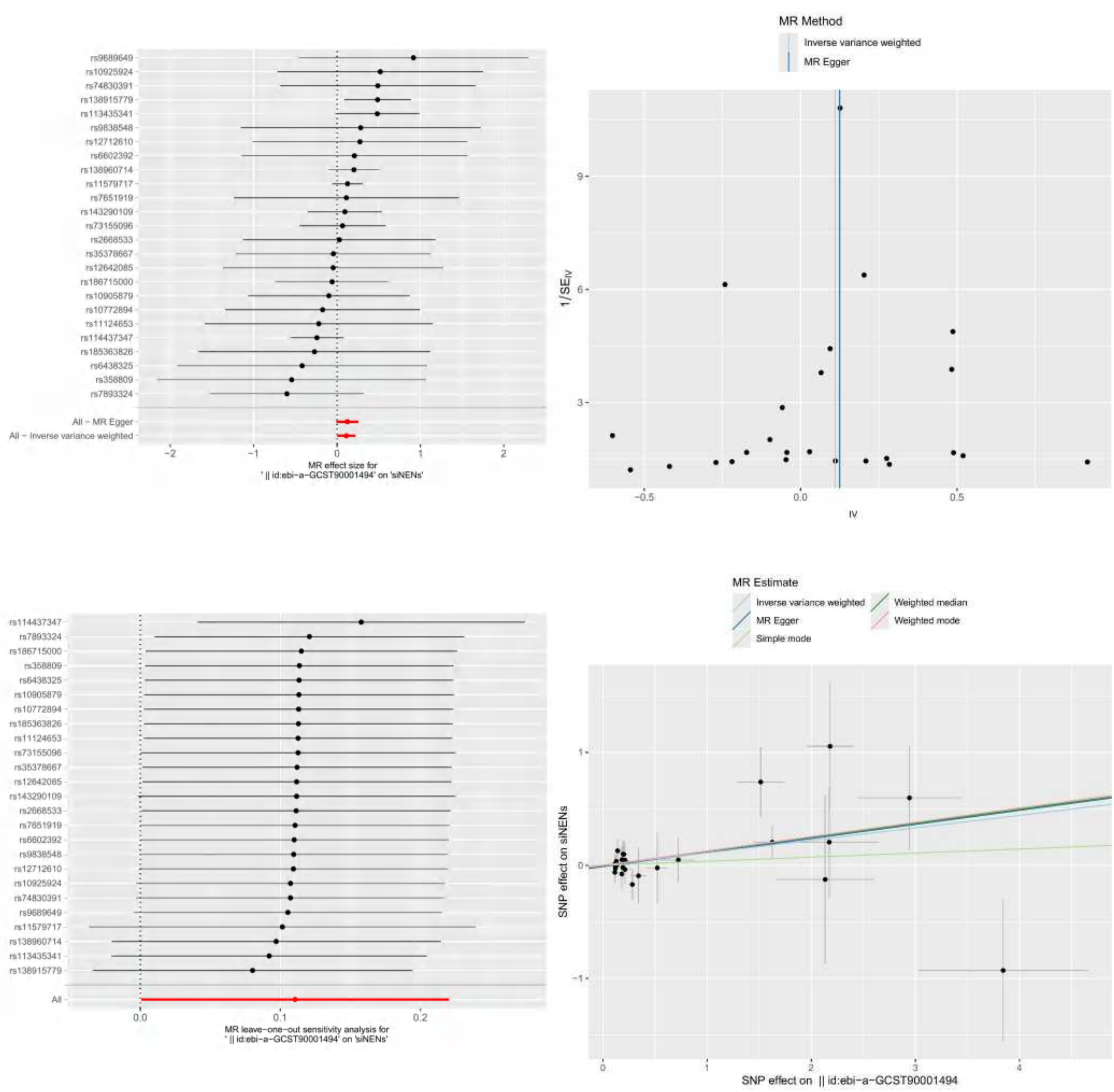
rs9689649
rs10925924
rs74830391
rs138915779
rs113435341
rs9838548
rs12712610
rs6602392
rs138960714
rs11579717
rs7651919
rs143290109
rs73155096
rs2668533
rs35378667
rs12642085
rs186715000
rs10905879
rs10772894
rs11124653
rs114437347
rs185363826
rs6438325
rs358809
rs7893324
All – MR Egger
All – Inverse variance weighted
MR effect size for
' || id:ebi-a-GCST90001494' on 'siNENs'
MR Method
Inverse variance weighted
MR Egger
1/SE_IV
IV
rs114437347
rs7893324
rs186715000
rs358809
rs6438325
rs10905879
rs10772894
rs185363826
rs11124653
rs73155096
rs35378667
rs12642085
rs143290109
rs2668533
rs7651919
rs6602392
rs9838548
rs12712610
rs10925924
rs74830391
rs9689649
rs11579717
rs138960714
rs113435341
rs138915779
All
MR leave-one-out sensitivity analysis for
' || id:ebi-a-GCST90001494' on 'siNENs'
MR Estimate
Inverse variance weighted
MR Egger
Simple mode
Weighted median
Weighted mode
SNP effect on siNENs
SNP effect on || id:ebi-a-GCST90001494

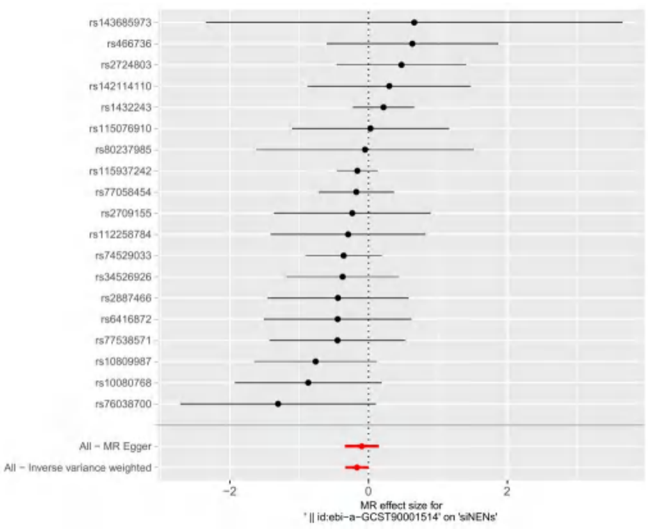
rs143685973
rs466736
rs2724803
rs142114110
rs1432243
rs115076910
rs80237985
rs115937242
rs77058454
rs2709155
rs112258784
rs74529033
rs34526926
rs2887466
rs6416872
rs77538571
rs10809987
rs10080768
rs76038700
All − MR Egger
All − Inverse variance weighted
−2
0
2
MR effect size for
' || id:ebi−a−GCST90001514' on 'siNENs'

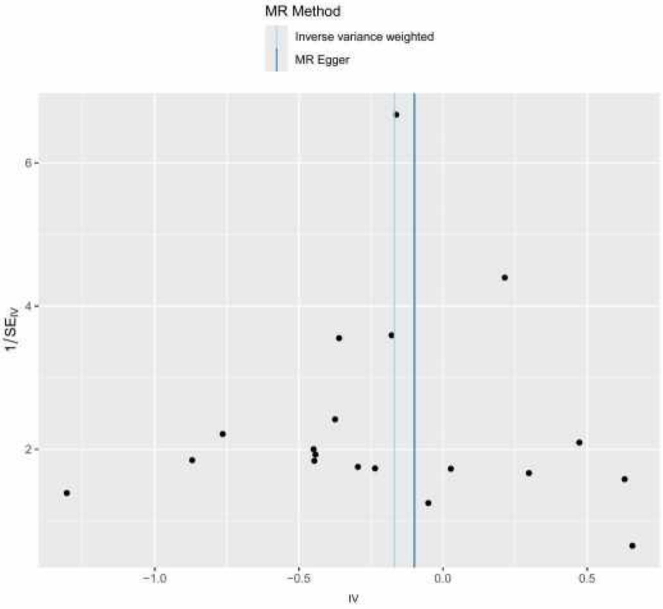
MR Method
Inverse variance weighted
MR Egger
1/SE_IV
6
4
2
−1.0
−0.5
0.0
0.5
IV

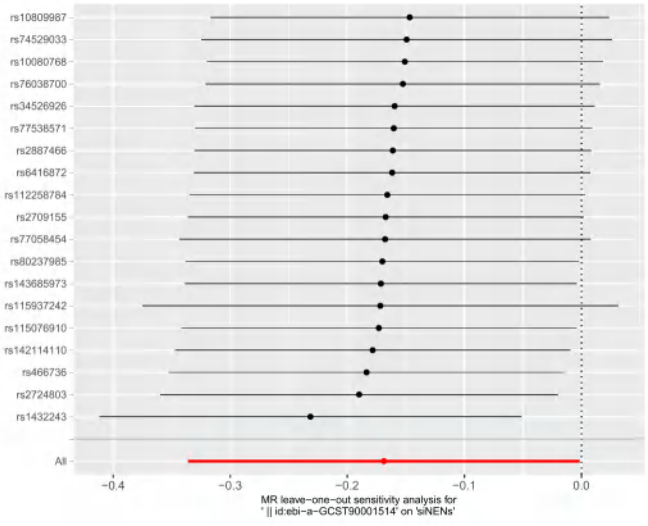
rs10809987
rs74529033
rs10080768
rs76038700
rs34526926
rs77538571
rs2887466
rs6416872
rs112258784
rs2709155
rs77058454
rs80237985
rs143685973
rs115937242
rs115076910
rs142114110
rs466736
rs2724803
rs1432243
All
−0.4
−0.3
−0.2
−0.1
0.0
MR leave−one−out sensitivity analysis for
' || id:ebi−a−GCST90001514' on 'siNENs'

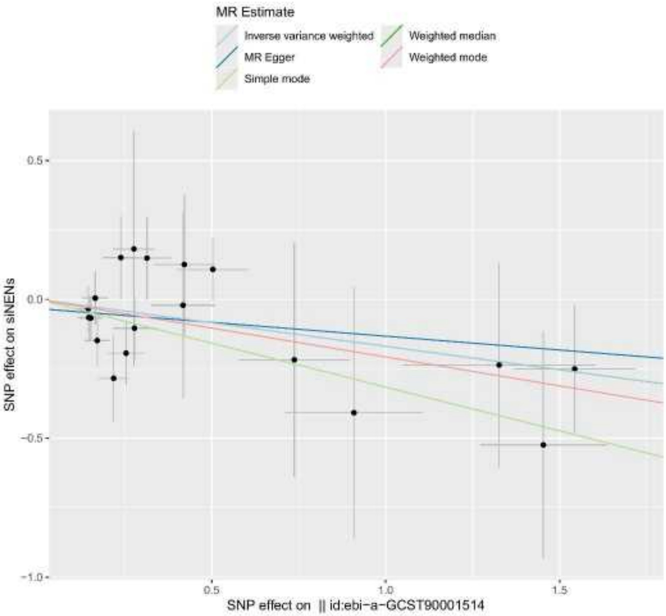
MR Estimate
Inverse variance weighted
MR Egger
Simple mode
Weighted median
Weighted mode
SNP effect on siNENs
0.5
0.0
−0.5
−1.0
0.5
1.0
1.5
SNP effect on || id:ebi−a−GCST90001514

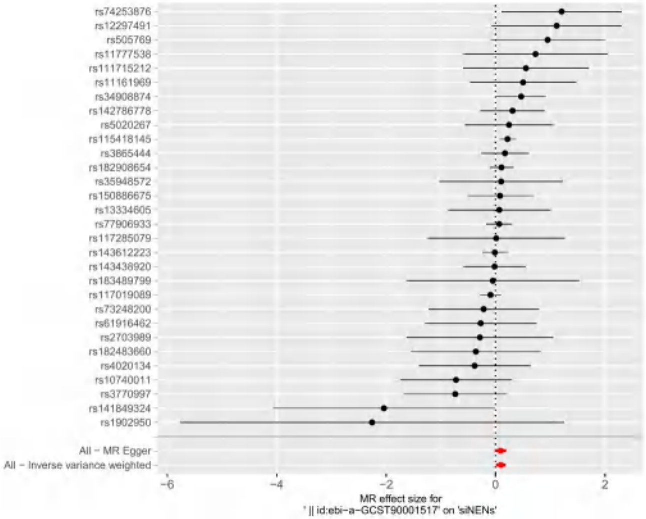
rs74253876
rs12297491
rs505769
rs11777538
rs111715212
rs11161969
rs34908874
rs142786778
rs5020267
rs115418145
rs3865444
rs182908654
rs35948572
rs150886675
rs13334605
rs77906933
rs117285079
rs143612223
rs143438920
rs183489799
rs117019089
rs73248200
rs61916462
rs2703989
rs182483660
rs4020134
rs10740011
rs3770997
rs141849324
rs1902950
All − MR Egger
All − Inverse variance weighted
−6
−4
−2
0
2
MR effect size for
' || id:ebi−a−GCST90001517' on 'siNENs'

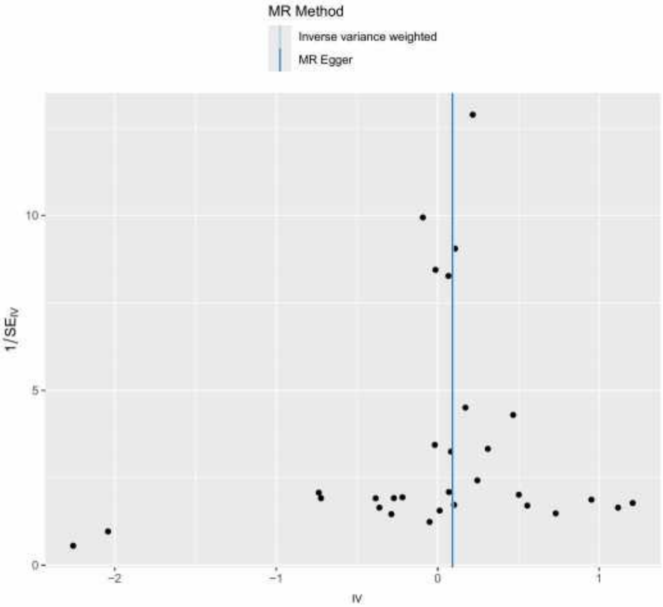
MR Method
Inverse variance weighted
MR Egger
1/SE_IV
10
5
0
−2
−1
0
1
IV

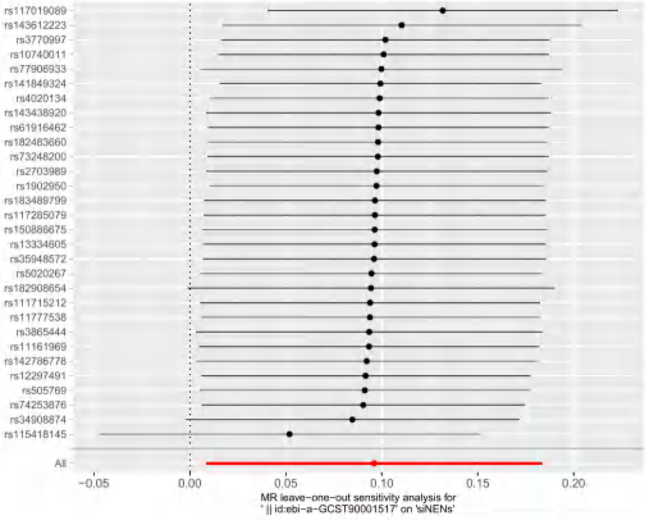
rs117019089
rs143612223
rs3770997
rs10740011
rs77906933
rs141849324
rs4020134
rs143438920
rs61916462
rs182483660
rs73248200
rs2703989
rs1902950
rs183489799
rs117285079
rs150886675
rs13334605
rs35948572
rs5020267
rs182908654
rs111715212
rs11777538
rs3865444
rs11161969
rs142786778
rs12297491
rs505769
rs74253876
rs34908874
rs115418145
All
−0.05
0.00
0.05
0.10
0.15
0.20
MR leave−one−out sensitivity analysis for
' || id:ebi−a−GCST90001517' on 'siNENs'

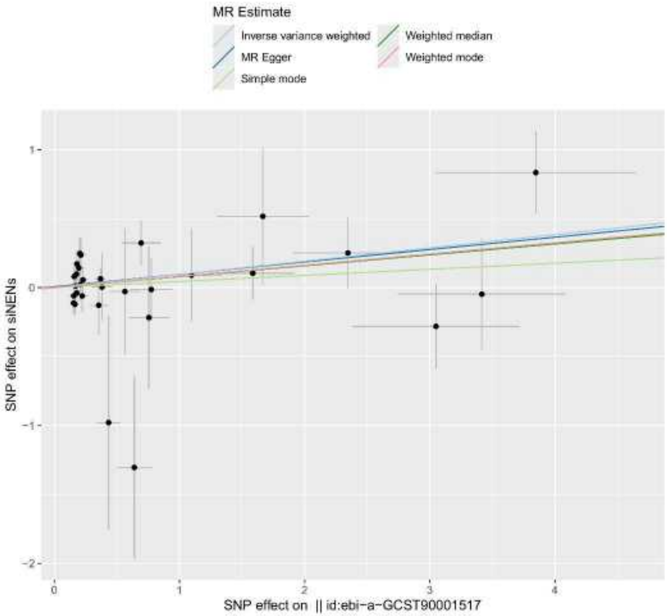
MR Estimate
Inverse variance weighted
MR Egger
Simple mode
Weighted median
Weighted mode
SNP effect on siNENs
1
0
−1
−2
0
1
2
3
4
SNP effect on || id:ebi−a−GCST90001517

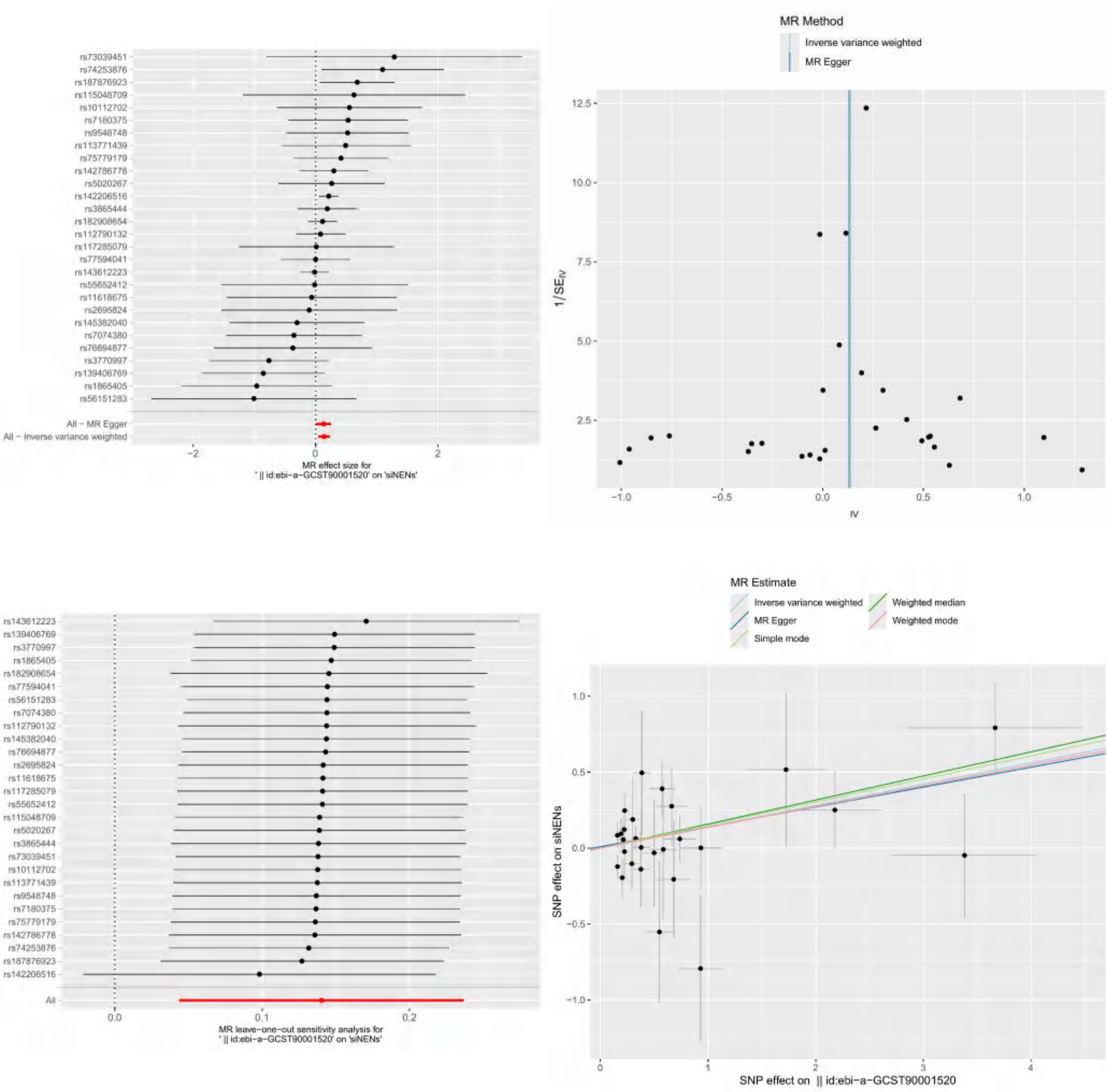
MR effect size for
' || id:ebi-a-GCST90001520' on 'siNENs'
All - MR Egger
All - inverse variance weighted
MR Method
Inverse variance weighted
MR Egger
1/SE_IV
IV
MR leave-one-out sensitivity analysis for
' || id:ebi-a-GCST90001520' on 'siNENs'
All
MR Estimate
Inverse variance weighted
MR Egger
Simple mode
Weighted median
Weighted mode
SNP effect on siNENs
SNP effect on || id:ebi-a-GCST90001520

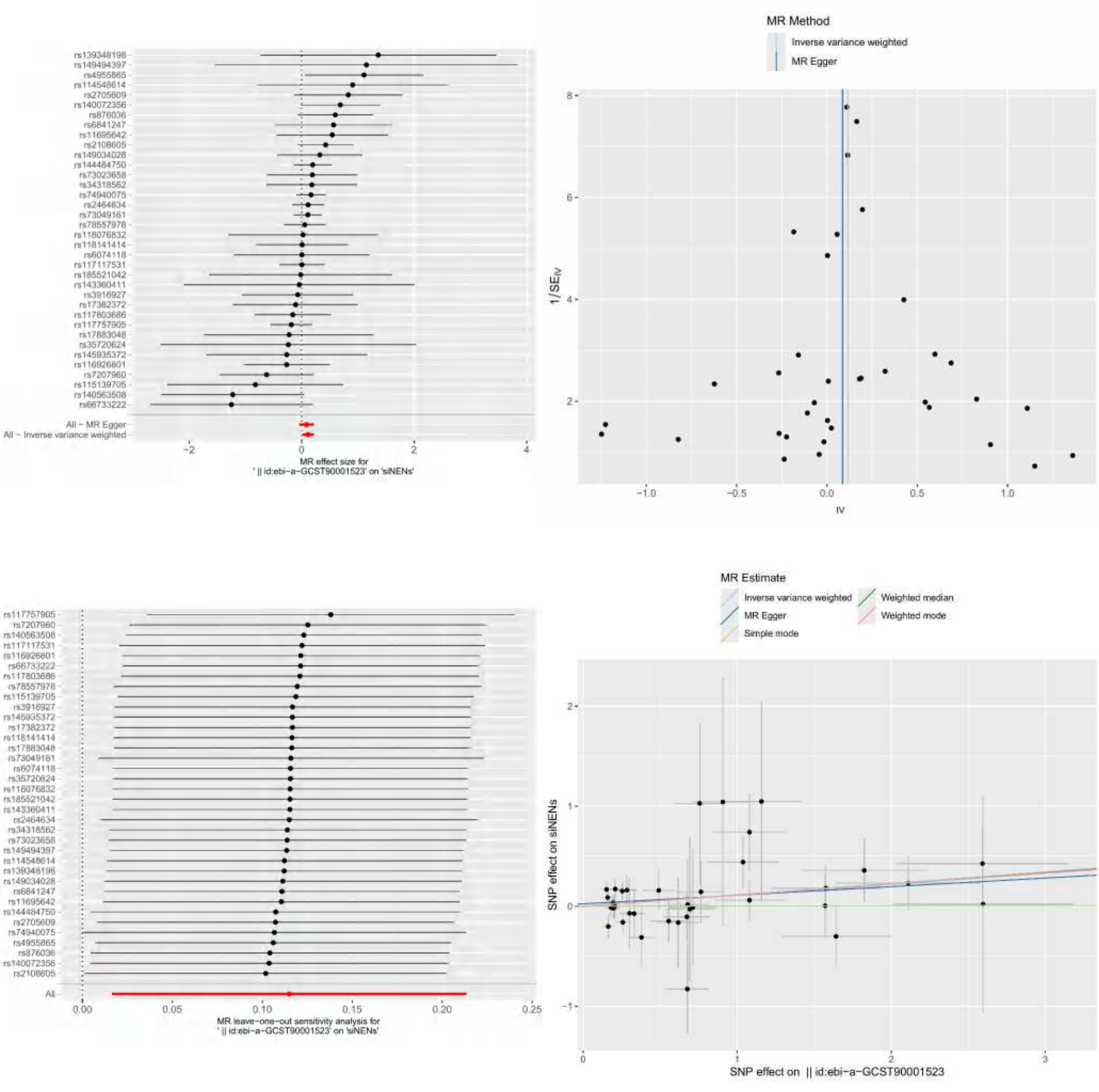

rs139348198
rs149494397
rs4955865
rs114548614
rs2705609
rs140072356
rs876036
rs6841247
rs11695642
rs2108605
rs149034028
rs144484750
rs73023658
rs34318562
rs74940075
rs2464634
rs73049161
rs78557978
rs118076832
rs118141414
rs6074118
rs117117531
rs185521042
rs143360411
rs3916927
rs17382372
rs117803686
rs117757905
rs17883048
rs35720624
rs145935372
rs116926801
rs7207960
rs115139705
rs140563508
rs66733222
All − MR Egger
All − Inverse variance weighted
MR effect size for
' || id:ebi-a-GCST90001523' on 'siNENs'
MR Method
Inverse variance weighted
MR Egger
1/SE_IV
IV
MR leave−one−out sensitivity analysis for
' || id:ebi-a-GCST90001523' on 'siNENs'
MR Estimate
Inverse variance weighted
MR Egger
Simple mode
Weighted median
Weighted mode
SNP effect on siNENs
SNP effect on || id:ebi-a-GCST90001523

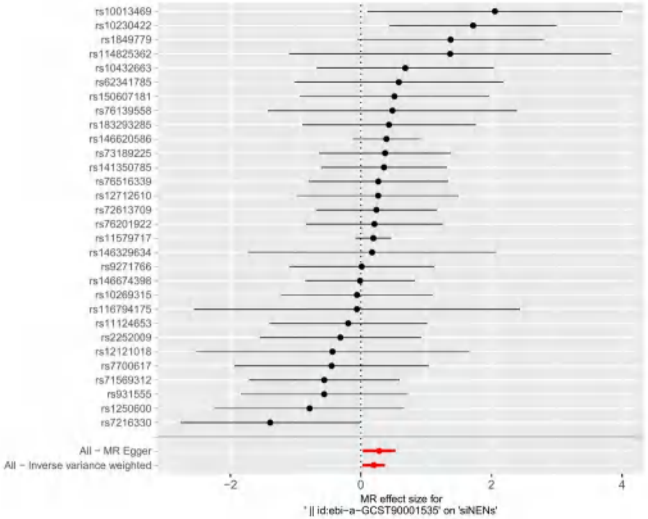

rs10013469
rs10230422
rs1849779
rs114825362
rs10432663
rs62341785
rs150607181
rs76139558
rs183293285
rs146620586
rs73189225
rs141350785
rs76516339
rs12712610
rs72613709
rs76201922
rs11579717
rs146329634
rs9271766
rs146674398
rs10269315
rs116794175
rs11124653
rs2252009
rs12121018
rs7700617
rs71569312
rs931555
rs1250600
rs7216330
All − MR Egger
All − Inverse variance weighted
−2
0
2
4
MR effect size for
' || id:ebi−a−GCST90001535' on 'siNENs'

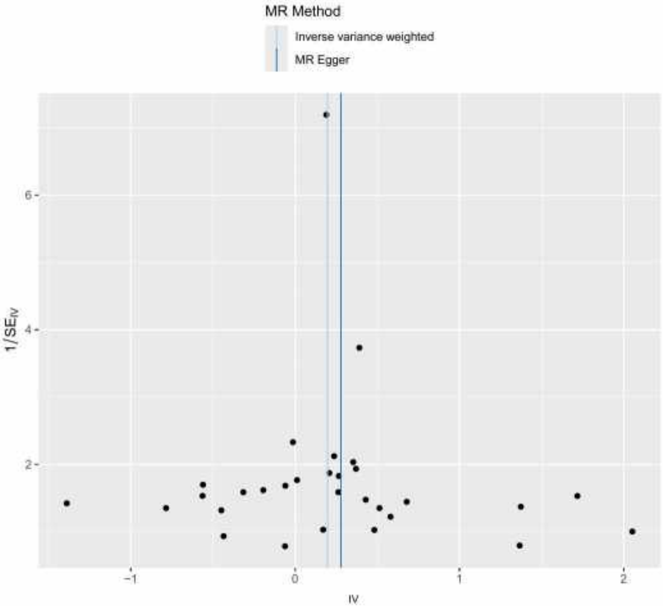

MR Method
Inverse variance weighted
MR Egger
1/SE$_{IV}$
6
4
2
−1
0
1
2
IV

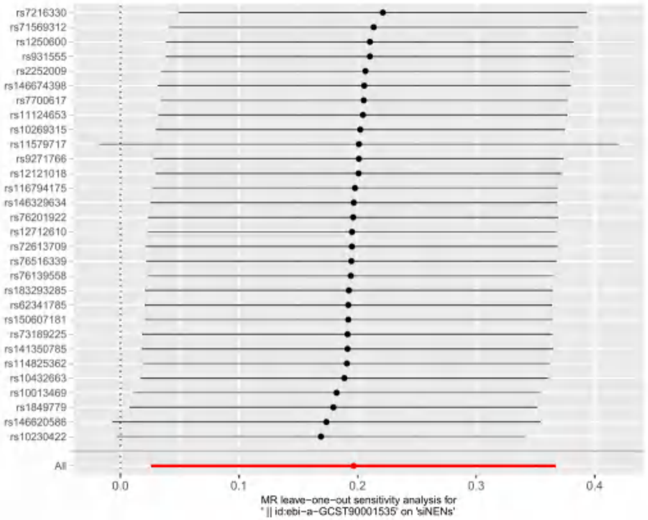

rs7216330
rs71569312
rs1250600
rs931555
rs2252009
rs146674398
rs7700617
rs11124653
rs10269315
rs11579717
rs9271766
rs12121018
rs116794175
rs146329634
rs76201922
rs12712610
rs72613709
rs76516339
rs76139558
rs183293285
rs62341785
rs150607181
rs73189225
rs141350785
rs114825362
rs10432663
rs10013469
rs1849779
rs146620586
rs10230422
All
0.0
0.1
0.2
0.3
0.4
MR leave−one−out sensitivity analysis for
' || id:ebi−a−GCST90001535' on 'siNENs'

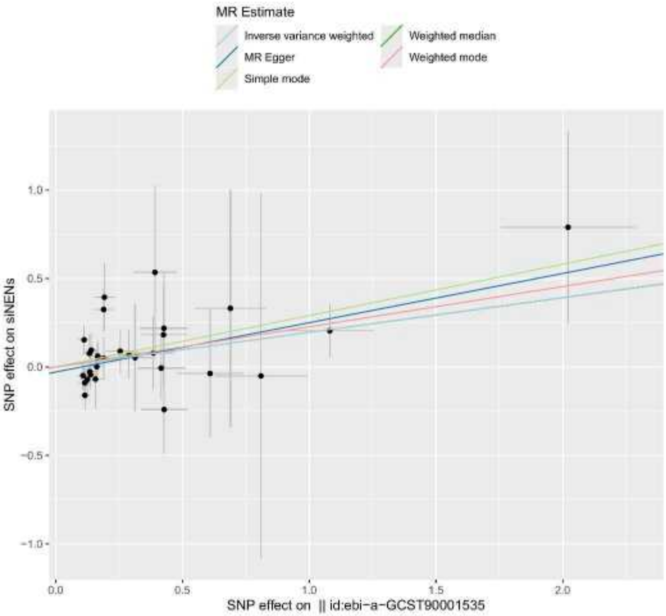

MR Estimate
Inverse variance weighted
MR Egger
Simple mode
Weighted median
Weighted mode
SNP effect on siNENs
1.0
0.5
0.0
−0.5
−1.0
0.0
0.5
1.0
1.5
2.0
SNP effect on || id:ebi−a−GCST90001535

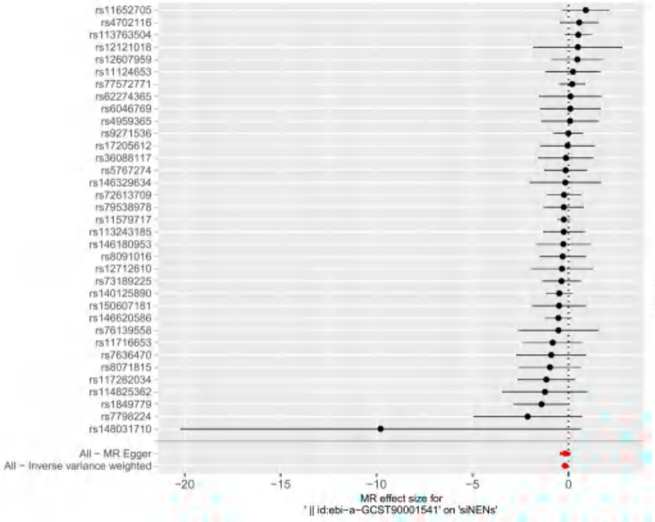
rs11652705
rs4702116
rs113763504
rs12121018
rs12607959
rs11124653
rs77572771
rs62274365
rs6046769
rs4959365
rs9271536
rs17205612
rs36088117
rs5767274
rs146329634
rs72613709
rs79538978
rs11579717
rs113243185
rs146180953
rs8091016
rs12712610
rs73189225
rs140125890
rs150607181
rs146620586
rs76139558
rs11716653
rs7636470
rs8071815
rs117262034
rs114825362
rs1849779
rs7798224
rs148031710
All − MR Egger
All − Inverse variance weighted
−20
−15
−10
−5
0
MR effect size for
' || id:ebi−a−GCST90001541' on 'siNENs'

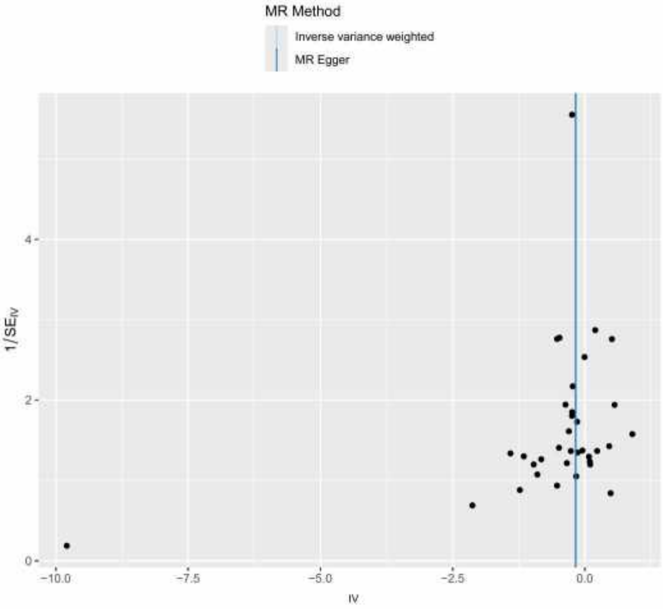
MR Method
Inverse variance weighted
MR Egger
$1/SE_{IV}$
0
2
4
−10.0
−7.5
−5.0
−2.5
0.0
IV

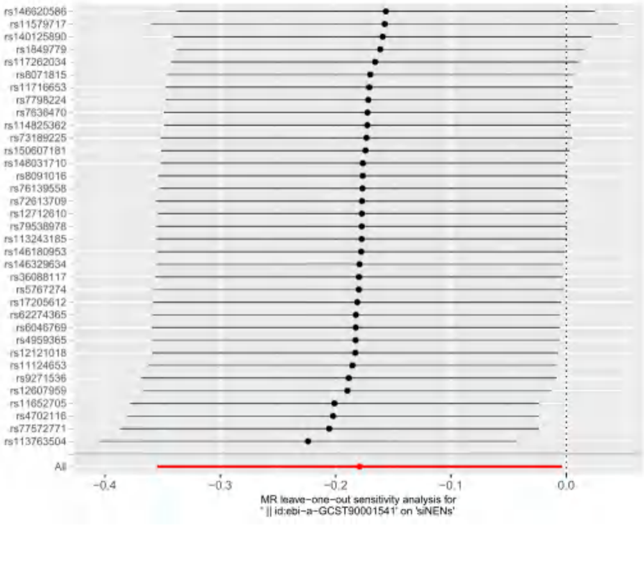
rs146620586
rs11579717
rs140125890
rs1849779
rs117262034
rs8071815
rs11716653
rs7798224
rs7636470
rs114825362
rs73189225
rs150607181
rs148031710
rs8091016
rs76139558
rs72613709
rs12712610
rs79538978
rs113243185
rs146180953
rs146329634
rs36088117
rs5767274
rs17205612
rs62274365
rs6046769
rs4959365
rs12121018
rs11124653
rs9271536
rs12607959
rs11652705
rs4702116
rs77572771
rs113763504
All
−0.4
−0.3
−0.2
−0.1
0.0
MR leave−one−out sensitivity analysis for
' || id:ebi−a−GCST90001541' on 'siNENs'

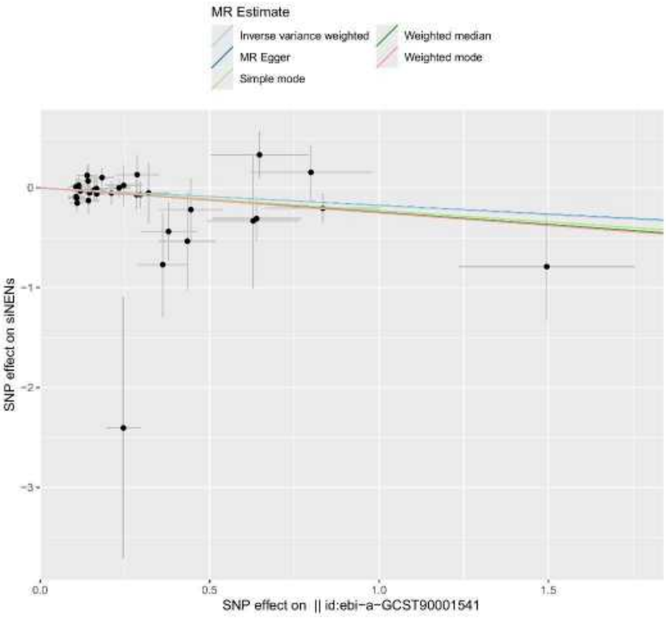
MR Estimate
Inverse variance weighted
MR Egger
Simple mode
Weighted median
Weighted mode
SNP effect on siNENs
0
−1
−2
−3
0.0
0.5
1.0
1.5
SNP effect on || id:ebi−a−GCST90001541

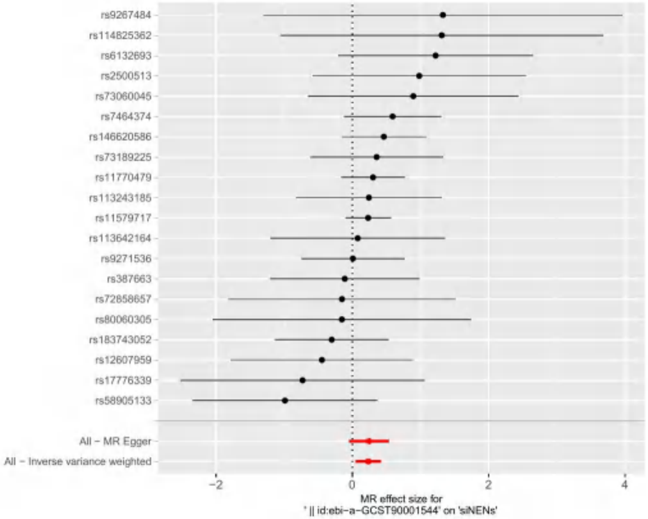

rs9267484
rs114825362
rs6132693
rs2500513
rs73060045
rs7464374
rs146620586
rs73189225
rs11770479
rs113243185
rs11579717
rs113642164
rs9271536
rs387663
rs72858657
rs80060305
rs183743052
rs12607959
rs17776339
rs58905133
All − MR Egger
All − Inverse variance weighted
−2
0
2
4
MR effect size for
' || id:ebi−a−GCST90001544' on 'siNENs'

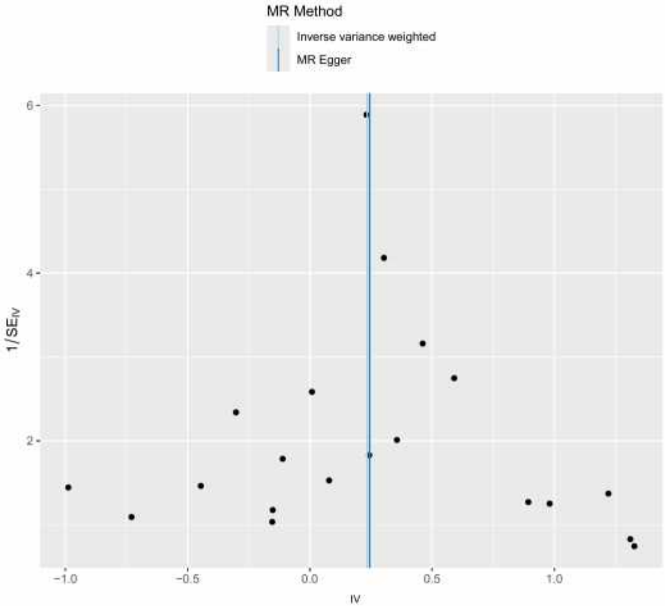

MR Method
Inverse variance weighted
MR Egger
6
4
2
$1/SE_{IV}$
−1.0
−0.5
0.0
0.5
1.0
IV

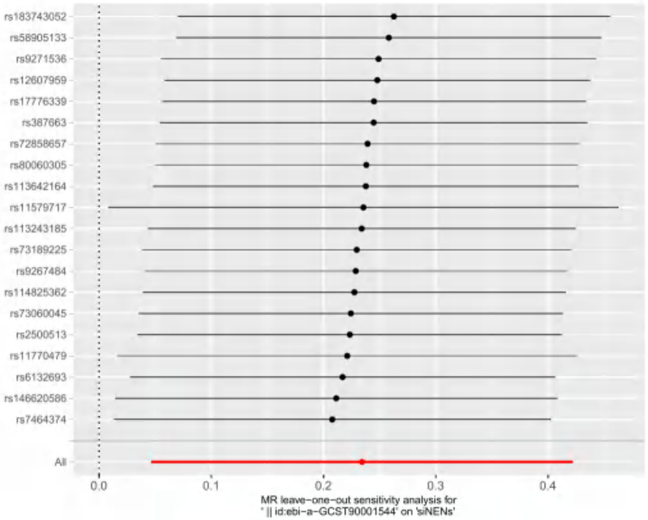

rs183743052
rs58905133
rs9271536
rs12607959
rs17776339
rs387663
rs72858657
rs80060305
rs113642164
rs11579717
rs113243185
rs73189225
rs9267484
rs114825362
rs73060045
rs2500513
rs11770479
rs6132693
rs146620586
rs7464374
All
0.0
0.1
0.2
0.3
0.4
MR leave−one−out sensitivity analysis for
' || id:ebi−a−GCST90001544' on 'siNENs'

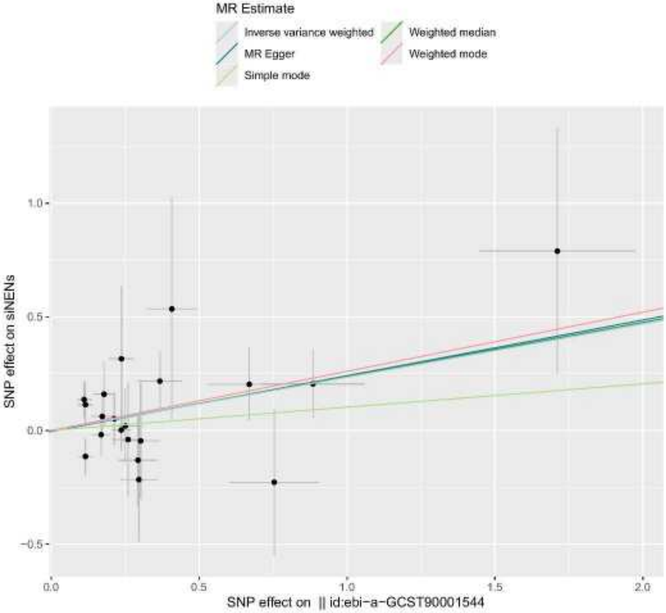

MR Estimate
Inverse variance weighted
MR Egger
Simple mode
Weighted median
Weighted mode
1.0
0.5
0.0
−0.5
SNP effect on siNENs
0.0
0.5
1.0
1.5
2.0
SNP effect on || id:ebi−a−GCST90001544

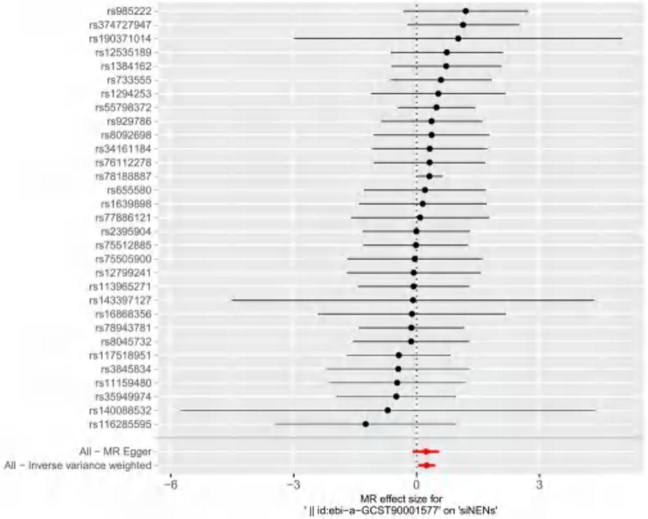
rs985222
rs374727947
rs190371014
rs12535189
rs1384162
rs733555
rs1294253
rs55798372
rs929786
rs8092698
rs34161184
rs76112278
rs78188887
rs655580
rs1639898
rs77886121
rs2395904
rs75512885
rs75505900
rs12799241
rs113965271
rs143397127
rs16868356
rs78943781
rs8045732
rs117518951
rs3845834
rs11159480
rs35949974
rs140088532
rs116285595
All − MR Egger
All − Inverse variance weighted
−6
−3
0
3
MR effect size for
' || id:ebi−a−GCST90001577' on 'siNENs'

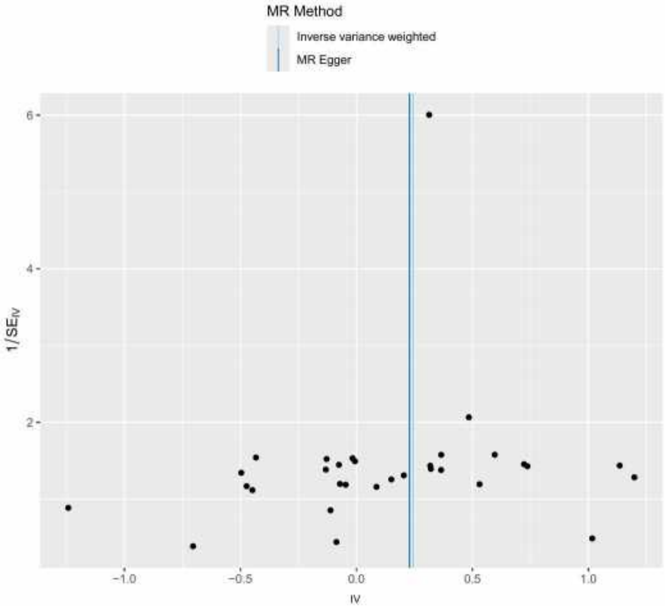
MR Method
Inverse variance weighted
MR Egger
1/SE$_{IV}$
6
4
2
−1.0
−0.5
0.0
0.5
1.0
IV

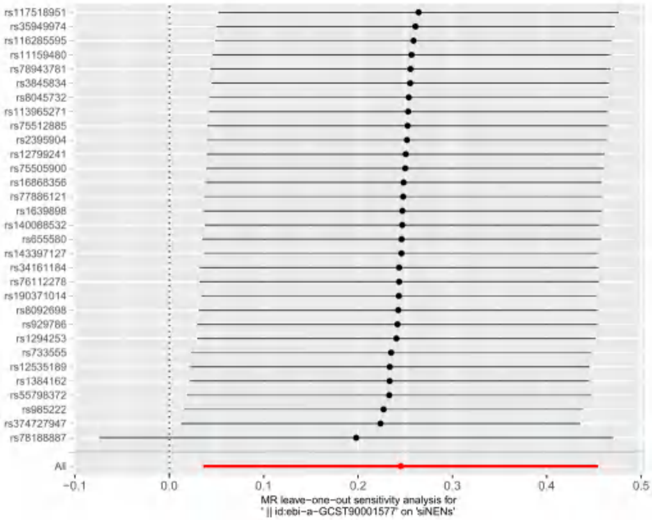
rs117518951
rs35949974
rs116285595
rs11159480
rs78943781
rs3845834
rs8045732
rs113965271
rs75512885
rs2395904
rs12799241
rs75505900
rs16868356
rs77886121
rs1639898
rs140088532
rs655580
rs143397127
rs34161184
rs76112278
rs190371014
rs8092698
rs929786
rs1294253
rs733555
rs12535189
rs1384162
rs55798372
rs985222
rs374727947
rs78188887
All
−0.1
0.0
0.1
0.2
0.3
0.4
0.5
MR leave−one−out sensitivity analysis for
' || id:ebi−a−GCST90001577' on 'siNENs'

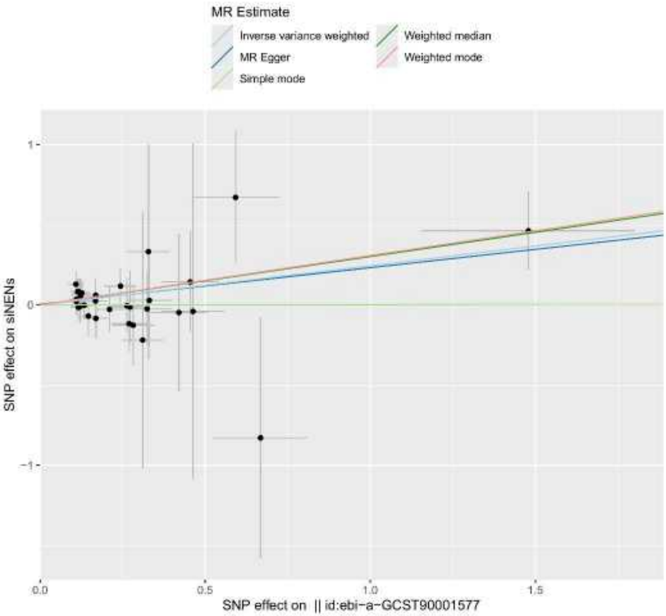
MR Estimate
Inverse variance weighted
MR Egger
Simple mode
Weighted median
Weighted mode
SNP effect on siNENs
1
0
−1
0.0
0.5
1.0
1.5
SNP effect on || id:ebi−a−GCST90001577

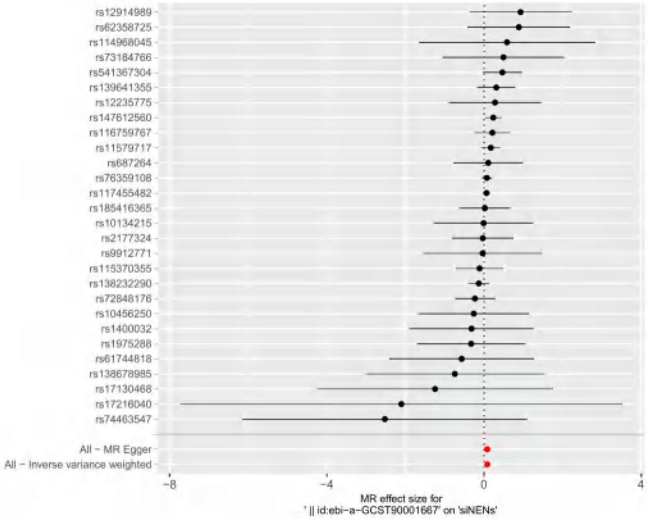
rs12914989
rs62358725
rs114968045
rs73184766
rs541367304
rs139641355
rs12235775
rs147612560
rs116759767
rs11579717
rs687264
rs76359108
rs117455482
rs185416365
rs10134215
rs2177324
rs9912771
rs115370355
rs138232290
rs72848176
rs10456250
rs1400032
rs1975288
rs61744818
rs138678985
rs17130468
rs17216040
rs74463547
All − MR Egger
All − Inverse variance weighted
-8
-4
0
4
MR effect size for
' || id:ebi-a-GCST90001667' on 'siNENs'

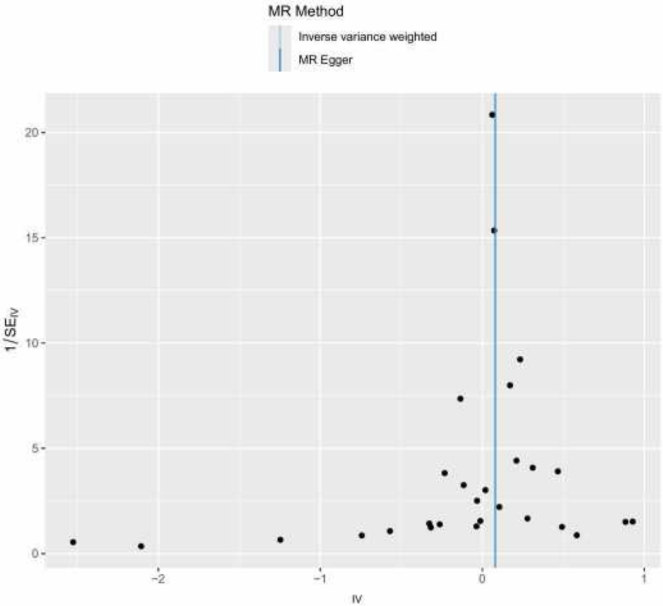
MR Method
Inverse variance weighted
MR Egger
1/SE$_{IV}$
0
5
10
15
20
-2
-1
0
1
IV

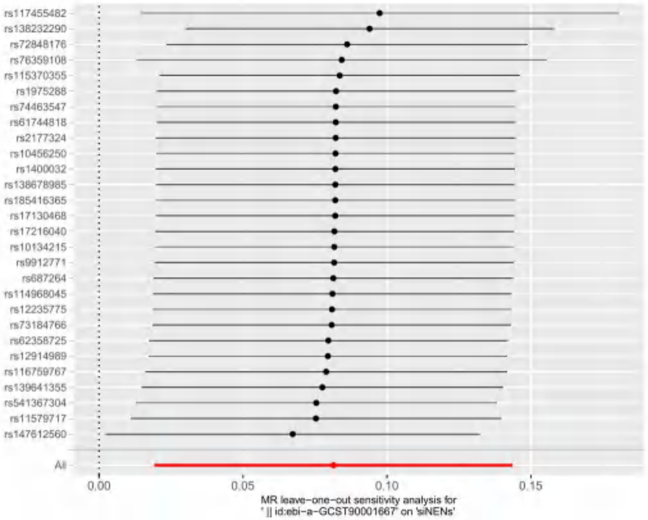
rs117455482
rs138232290
rs72848176
rs76359108
rs115370355
rs1975288
rs74463547
rs61744818
rs2177324
rs10456250
rs1400032
rs138678985
rs185416365
rs17130468
rs17216040
rs10134215
rs9912771
rs687264
rs114968045
rs12235775
rs73184766
rs62358725
rs12914989
rs116759767
rs139641355
rs541367304
rs11579717
rs147612560
All
0.00
0.05
0.10
0.15
MR leave-one-out sensitivity analysis for
' || id:ebi-a-GCST90001667' on 'siNENs'

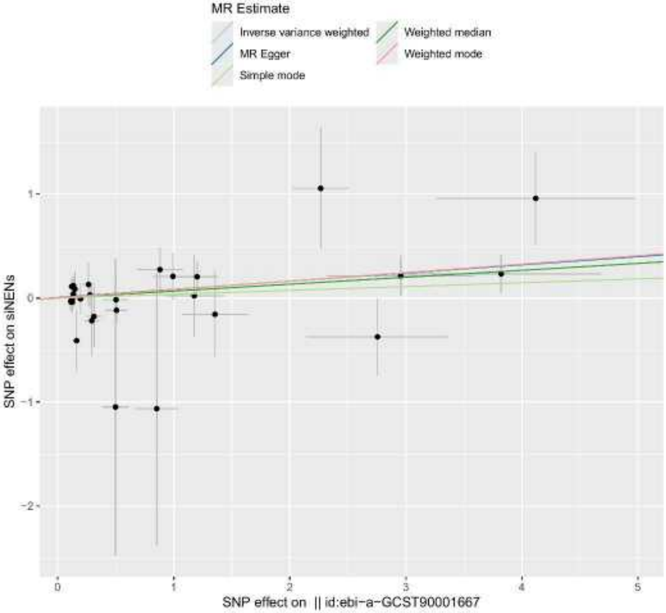
MR Estimate
Inverse variance weighted
MR Egger
Simple mode
Weighted median
Weighted mode
SNP effect on siNENs
1
0
-1
-2
0
1
2
3
4
5
SNP effect on || id:ebi-a-GCST90001667

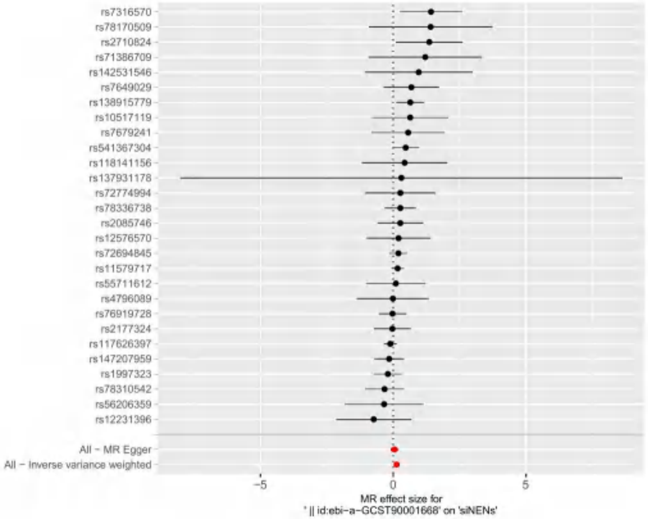
rs7316570
rs78170509
rs2710824
rs71386709
rs142531546
rs7649029
rs138915779
rs10517119
rs7679241
rs541367304
rs118141156
rs137931178
rs72774994
rs78336738
rs2085746
rs12576570
rs72694845
rs11579717
rs55711612
rs4796089
rs76919728
rs2177324
rs117626397
rs147207959
rs1997323
rs78310542
rs56206359
rs12231396
All − MR Egger
All − Inverse variance weighted
−5
0
5
MR effect size for
' || id:ebi−a−GCST90001668' on 'siNENs'

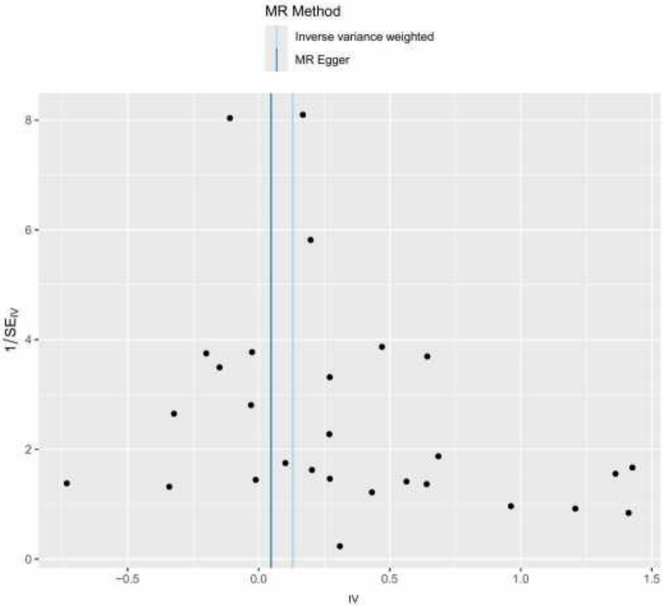
MR Method
Inverse variance weighted
MR Egger
1/SE_IV
0
2
4
6
8
−0.5
0.0
0.5
1.0
1.5
IV

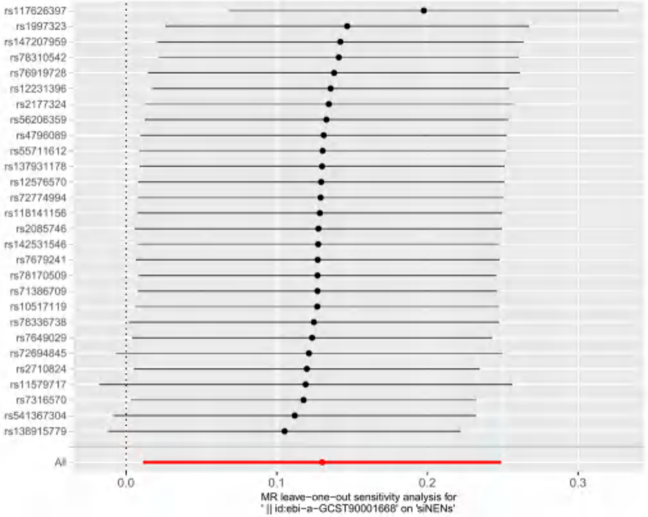
rs117626397
rs1997323
rs147207959
rs78310542
rs76919728
rs12231396
rs2177324
rs56206359
rs4796089
rs55711612
rs137931178
rs12576570
rs72774994
rs118141156
rs2085746
rs142531546
rs7679241
rs78170509
rs71386709
rs10517119
rs78336738
rs7649029
rs72694845
rs2710824
rs11579717
rs7316570
rs541367304
rs138915779
All
0.0
0.1
0.2
0.3
MR leave−one−out sensitivity analysis for
' || id:ebi−a−GCST90001668' on 'siNENs'

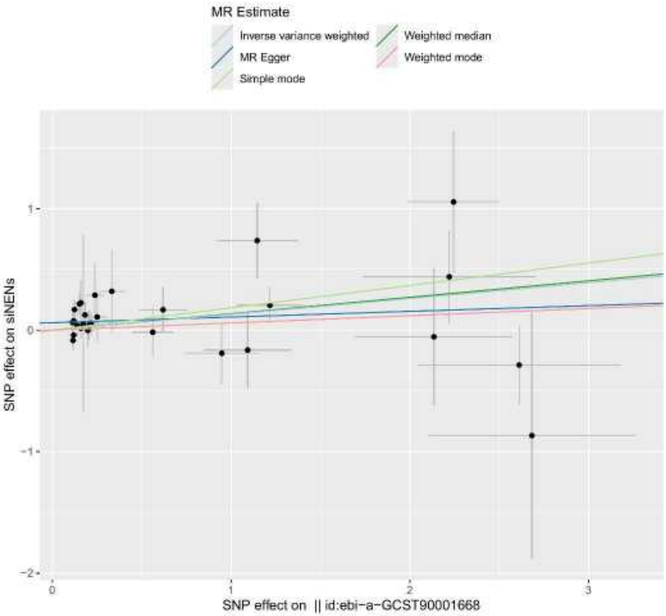
MR Estimate
Inverse variance weighted
MR Egger
Simple mode
Weighted median
Weighted mode
SNP effect on siNENs
1
0
−1
−2
0
1
2
3
SNP effect on || id:ebi−a−GCST90001668

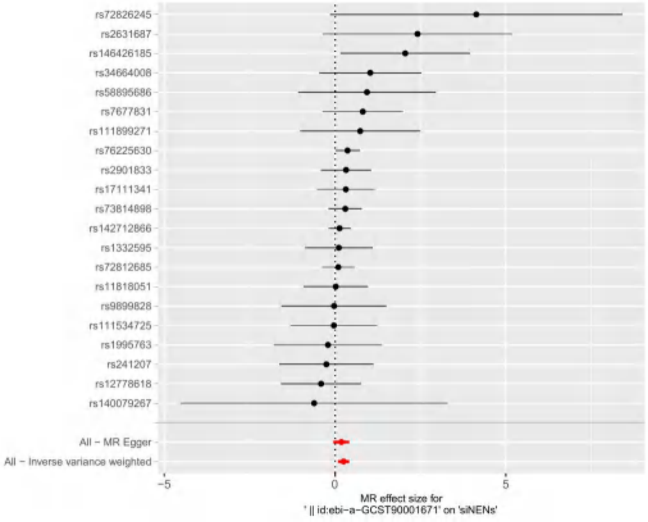
rs72826245
rs2631687
rs146426185
rs34664008
rs58895686
rs7677831
rs111899271
rs76225630
rs2901833
rs17111341
rs73814898
rs142712866
rs1332595
rs72812685
rs11818051
rs9899828
rs111534725
rs1995763
rs241207
rs12778618
rs140079267
All − MR Egger
All − Inverse variance weighted
-5
0
5
MR effect size for
' || id:ebi−a−GCST90001671' on 'siNENs'

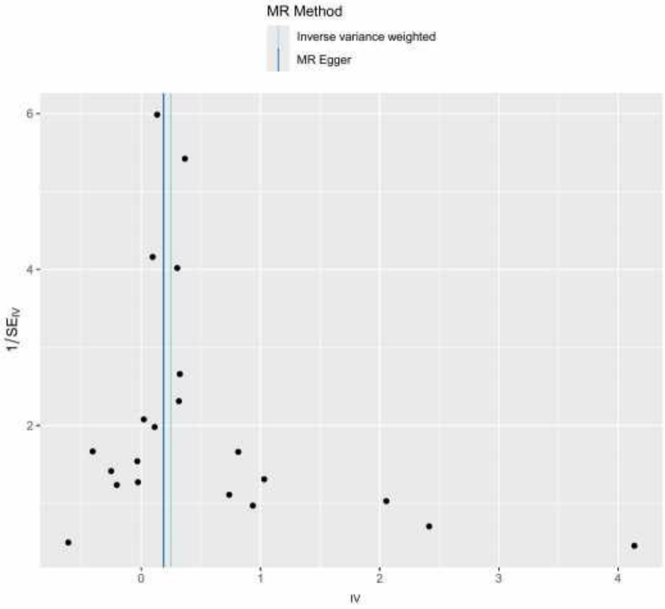
MR Method
Inverse variance weighted
MR Egger
1/SE$_{IV}$
6
4
2
0
1
2
3
4
IV

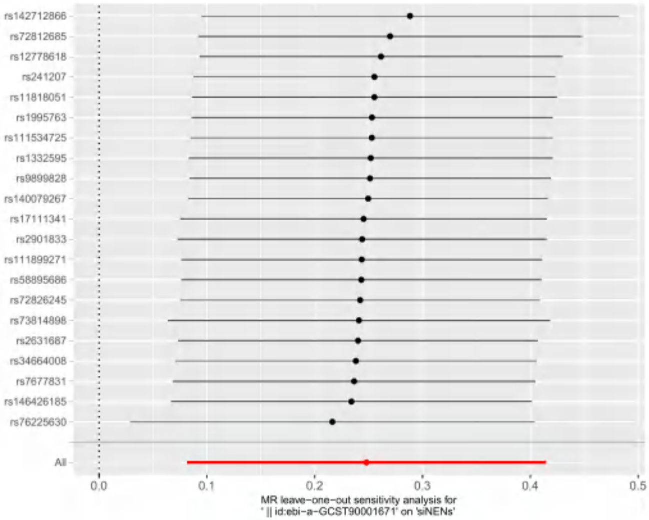
rs142712866
rs72812685
rs12778618
rs241207
rs11818051
rs1995763
rs111534725
rs1332595
rs9899828
rs140079267
rs17111341
rs2901833
rs111899271
rs58895686
rs72826245
rs73814898
rs2631687
rs34664008
rs7677831
rs146426185
rs76225630
All
0.0
0.1
0.2
0.3
0.4
0.5
MR leave−one−out sensitivity analysis for
' || id:ebi−a−GCST90001671' on 'siNENs'

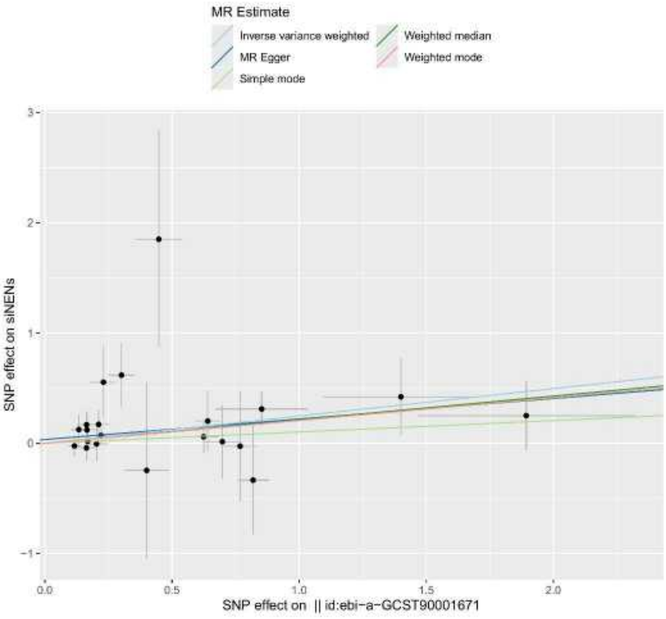
MR Estimate
Inverse variance weighted
MR Egger
Simple mode
Weighted median
Weighted mode
SNP effect on siNENs
3
2
1
0
-1
0.0
0.5
1.0
1.5
2.0
SNP effect on || id:ebi−a−GCST90001671

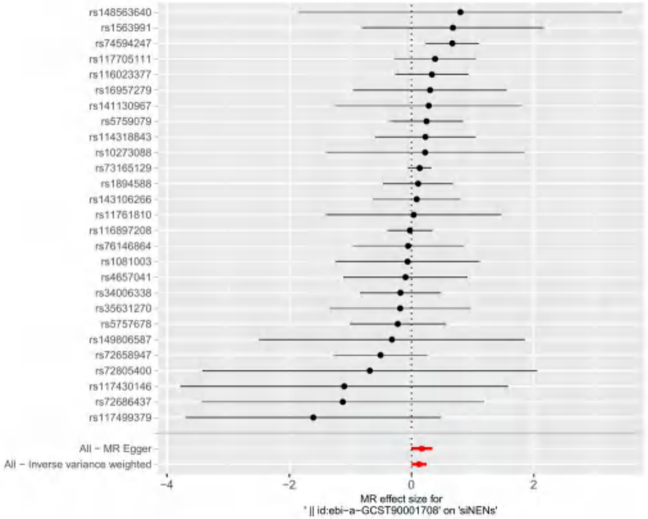
rs148563640
rs1563991
rs74594247
rs117705111
rs116023377
rs16957279
rs141130967
rs5759079
rs114318843
rs10273088
rs73165129
rs1894588
rs143106266
rs11761810
rs116897208
rs76146864
rs1081003
rs4657041
rs34006338
rs35631270
rs5757678
rs149806587
rs72658947
rs72805400
rs117430146
rs72686437
rs117499379
All − MR Egger
All − Inverse variance weighted
-4
-2
0
2
MR effect size for
' || id:ebi−a−GCST90001708' on 'siNENs'

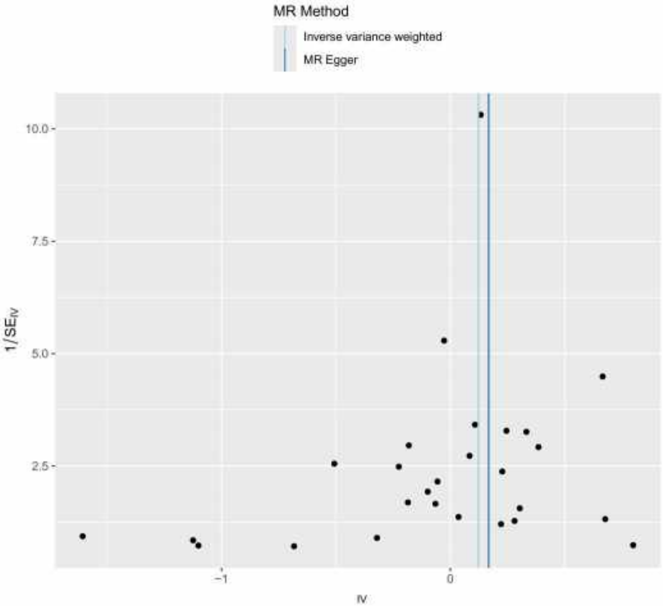
MR Method
Inverse variance weighted
MR Egger
10.0
7.5
5.0
2.5
$1/SE_{IV}$
−1
0
IV

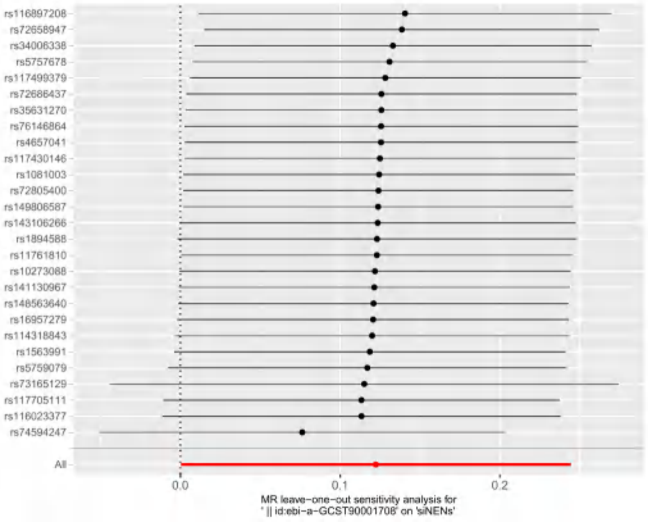
rs116897208
rs72658947
rs34006338
rs5757678
rs117499379
rs72686437
rs35631270
rs76146864
rs4657041
rs117430146
rs1081003
rs72805400
rs149806587
rs143106266
rs1894588
rs11761810
rs10273088
rs141130967
rs148563640
rs16957279
rs114318843
rs1563991
rs5759079
rs73165129
rs117705111
rs116023377
rs74594247
All
0.0
0.1
0.2
MR leave−one−out sensitivity analysis for
' || id:ebi−a−GCST90001708' on 'siNENs'

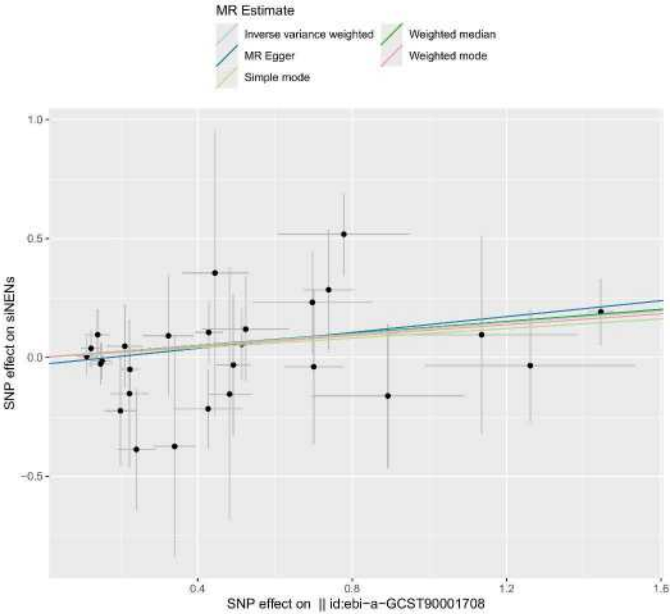
MR Estimate
Inverse variance weighted
MR Egger
Simple mode
Weighted median
Weighted mode
1.0
0.5
0.0
−0.5
SNP effect on siNENs
0.4
0.8
1.2
1.6
SNP effect on || id:ebi−a−GCST90001708

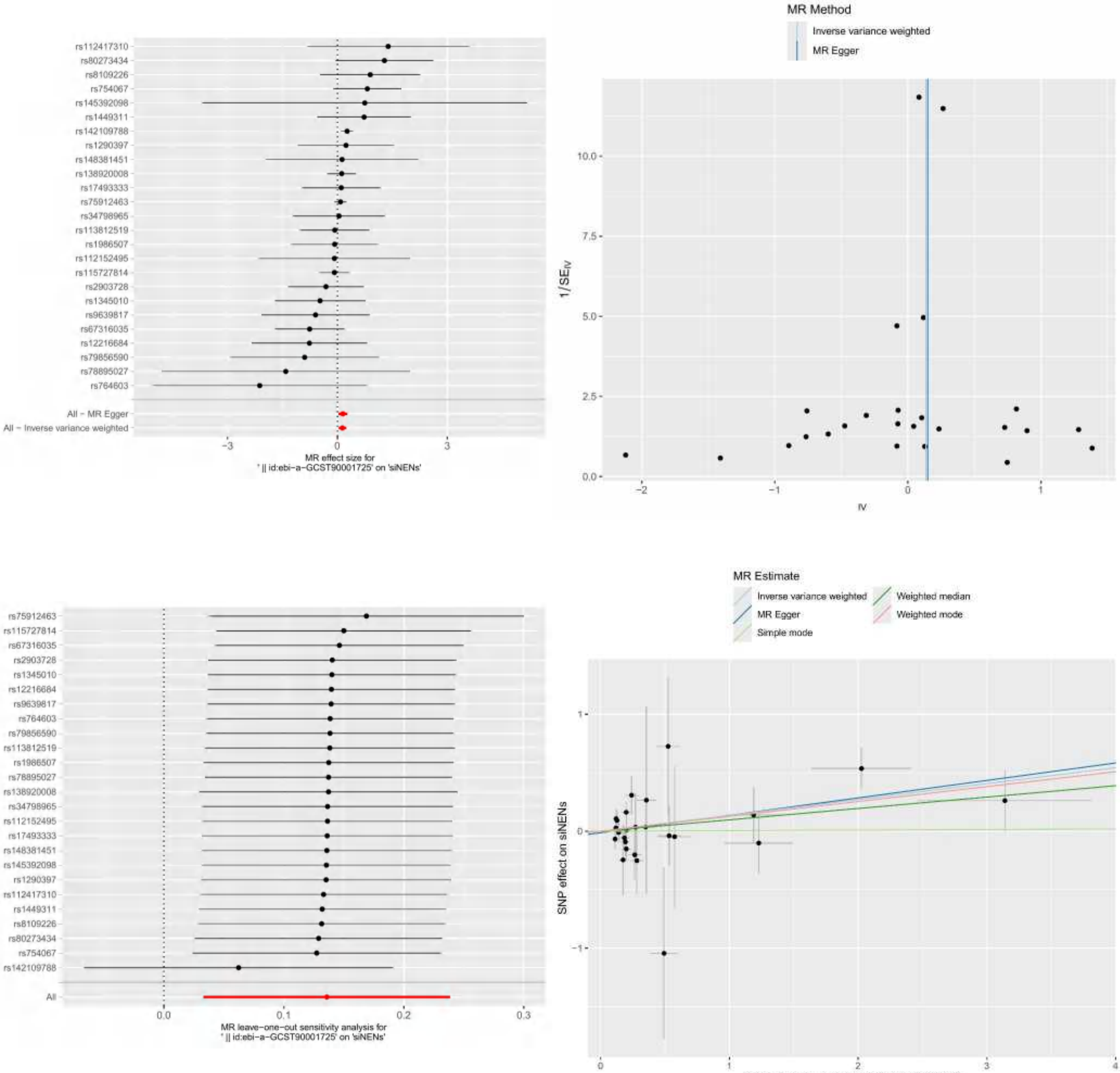

rs112417310
rs80273434
rs8109226
rs754067
rs145392098
rs1449311
rs142109788
rs1290397
rs148381451
rs138920008
rs17493333
rs75912463
rs34798965
rs113812519
rs1986507
rs112152495
rs115727814
rs2903728
rs1345010
rs9639817
rs67316035
rs12216684
rs79856590
rs78895027
rs764603
All - MR Egger
All - Inverse variance weighted
MR effect size for
' || id:ebi-a-GCST90001725' on 'siNENs'
MR Method
Inverse variance weighted
MR Egger
1/SE_IV
IV
MR leave-one-out sensitivity analysis for
' || id:ebi-a-GCST90001725' on 'siNENs'
All
MR Estimate
Inverse variance weighted
MR Egger
Simple mode
Weighted median
Weighted mode
SNP effect on siNENs
SNP effect on || id:ebi-a-GCST90001725

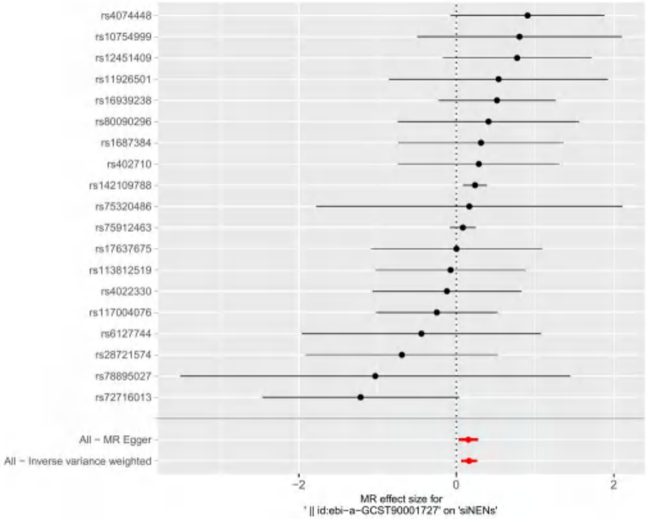
rs4074448
rs10754999
rs12451409
rs11926501
rs16939238
rs80090296
rs1687384
rs402710
rs142109788
rs75320486
rs75912463
rs17637675
rs113812519
rs4022330
rs117004076
rs6127744
rs28721574
rs78895027
rs72716013
All – MR Egger
All – Inverse variance weighted
−2
0
2
MR effect size for
' || id:ebi−a−GCST90001727' on 'siNENs'

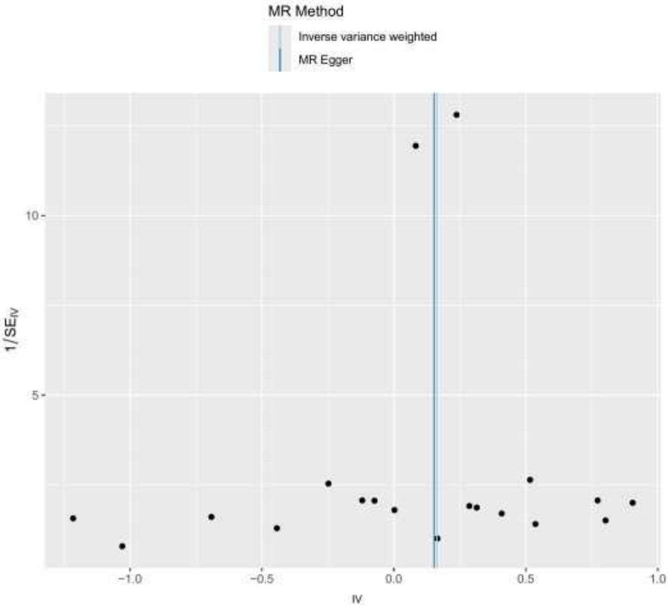
MR Method
Inverse variance weighted
MR Egger
1/SE_IV
10
5
−1.0
−0.5
0.0
0.5
1.0
IV

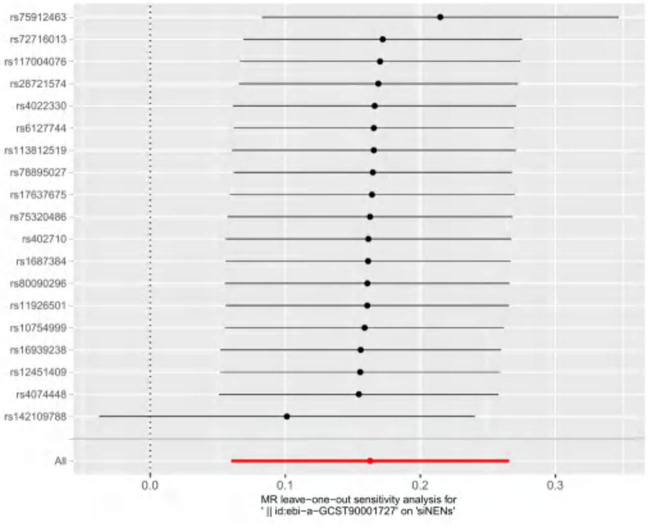
rs75912463
rs72716013
rs117004076
rs28721574
rs4022330
rs6127744
rs113812519
rs78895027
rs17637675
rs75320486
rs402710
rs1687384
rs80090296
rs11926501
rs10754999
rs16939238
rs12451409
rs4074448
rs142109788
All
0.0
0.1
0.2
0.3
MR leave−one−out sensitivity analysis for
' || id:ebi−a−GCST90001727' on 'siNENs'

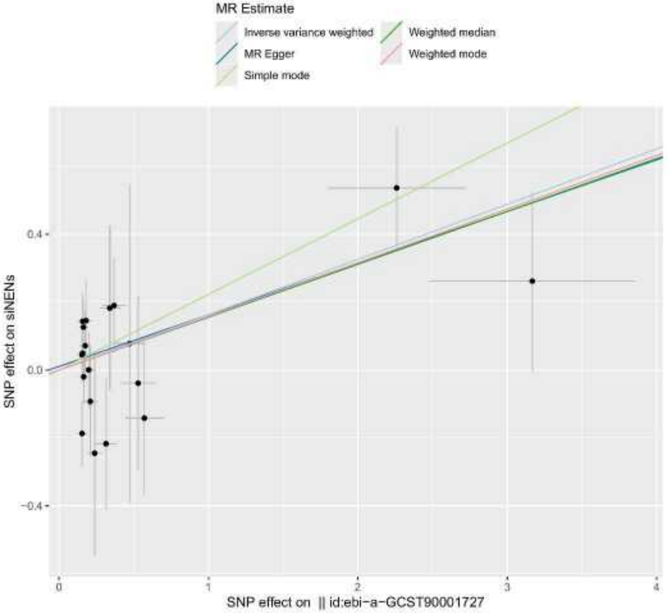
MR Estimate
Inverse variance weighted
MR Egger
Simple mode
Weighted median
Weighted mode
SNP effect on siNENs
0.4
0.0
−0.4
0
1
2
3
4
SNP effect on || id:ebi−a−GCST90001727

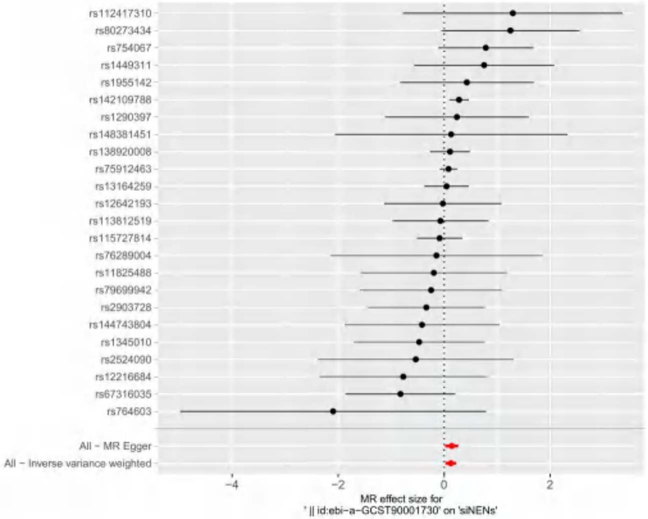
rs112417310
rs80273434
rs754067
rs1449311
rs1955142
rs142109788
rs1290397
rs148381451
rs138920008
rs75912463
rs13164259
rs12642193
rs113812519
rs115727814
rs76289004
rs11825488
rs79699942
rs2903728
rs144743804
rs1345010
rs2524090
rs12216684
rs67316035
rs764603
All - MR Egger
All - Inverse variance weighted
-4
-2
0
2
MR effect size for
' || id:ebi-a-GCST90001730' on 'siNENs'

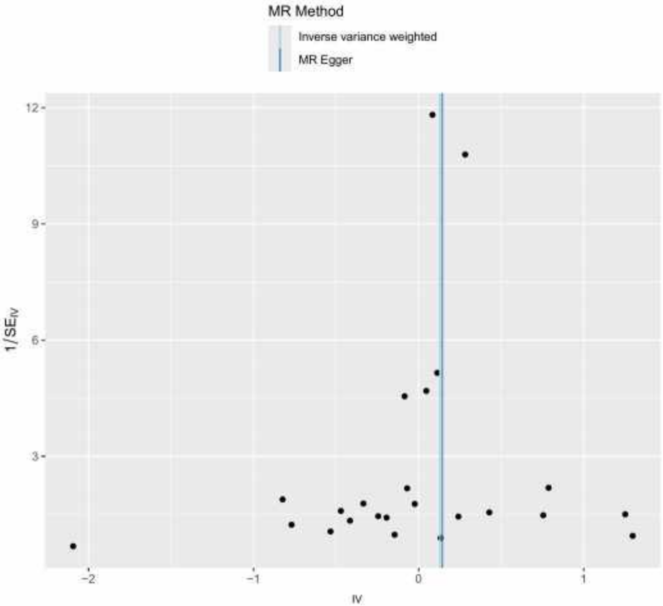
MR Method
Inverse variance weighted
MR Egger
12
9
6
3
$1/SE_{IV}$
−2
−1
0
1
IV

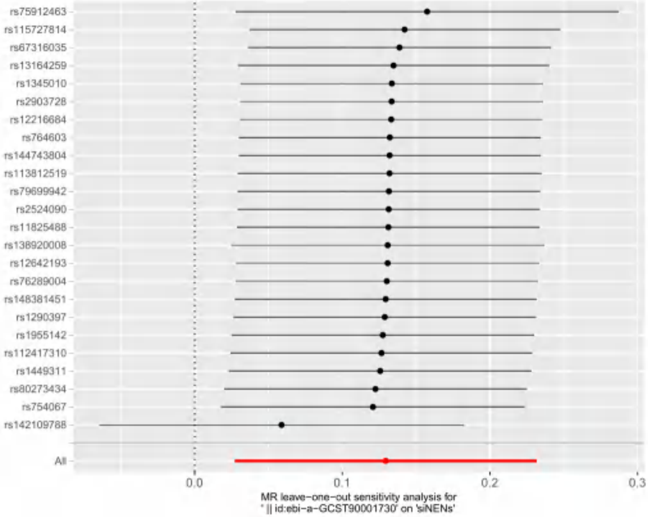
rs75912463
rs115727814
rs67316035
rs13164259
rs1345010
rs2903728
rs12216684
rs764603
rs144743804
rs113812519
rs79699942
rs2524090
rs11825488
rs138920008
rs12642193
rs76289004
rs148381451
rs1290397
rs1955142
rs112417310
rs1449311
rs80273434
rs754067
rs142109788
All
0.0
0.1
0.2
0.3
MR leave−one−out sensitivity analysis for
' || id:ebi−a−GCST90001730' on 'siNENs'

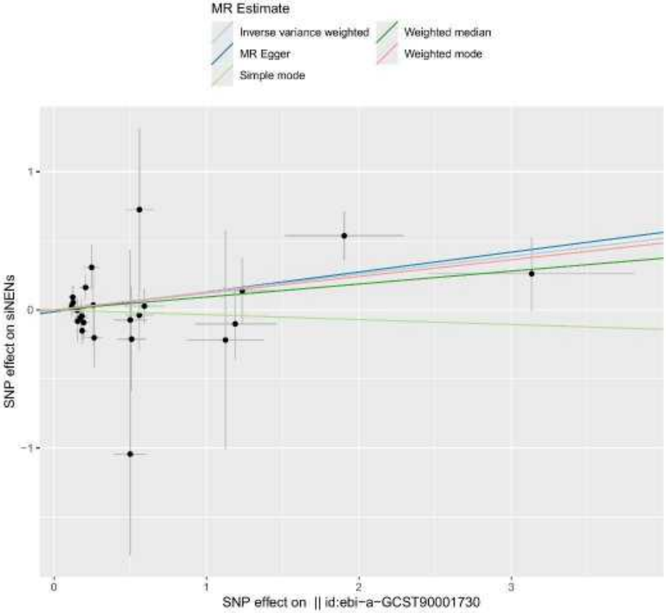
MR Estimate
Inverse variance weighted
MR Egger
Simple mode
Weighted median
Weighted mode
1
0
−1
SNP effect on siNENs
0
1
2
3
SNP effect on || id:ebi−a−GCST90001730

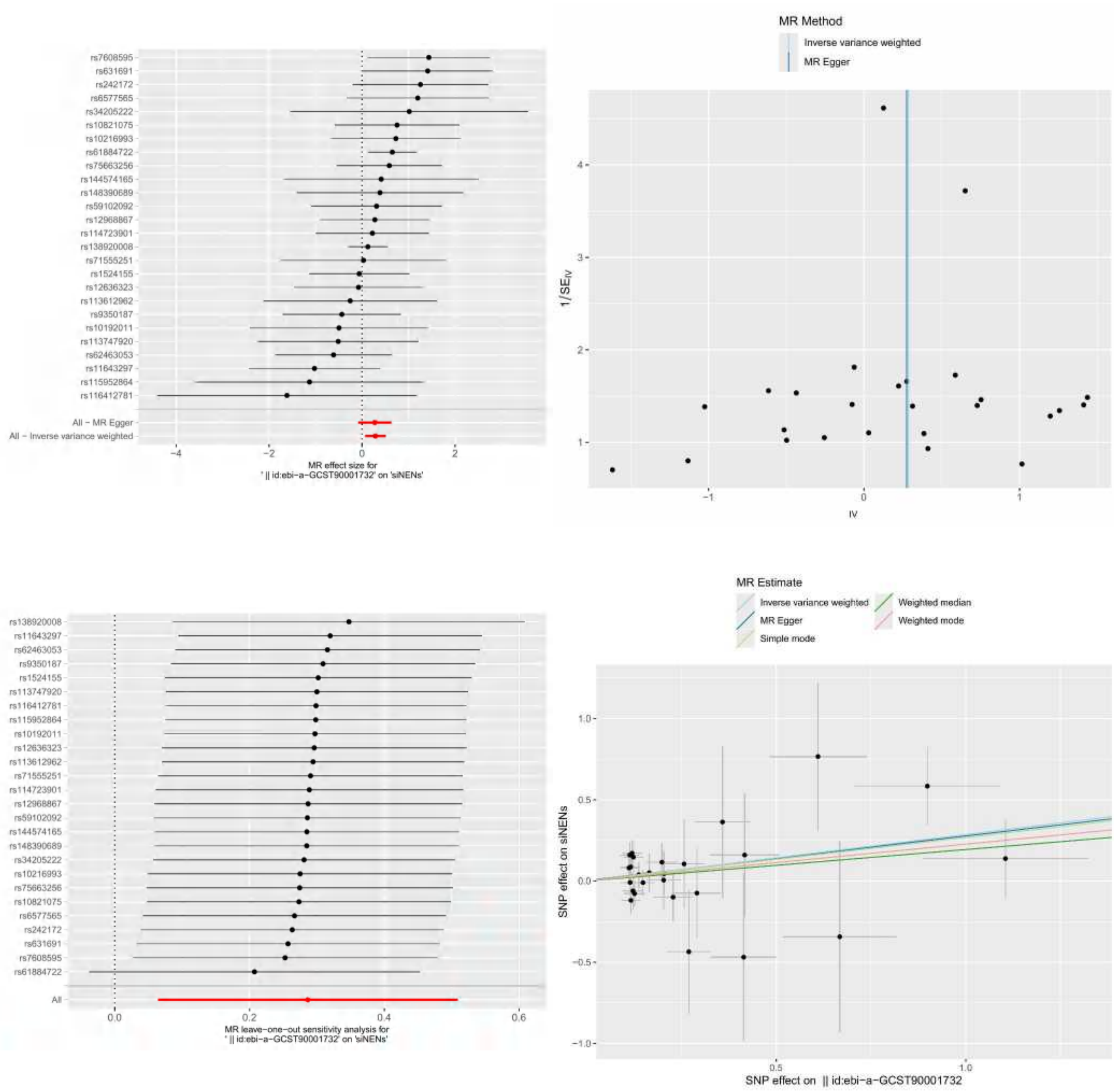
rs7608595
rs631691
rs242172
rs6577565
rs34205222
rs10821075
rs10216993
rs61884722
rs75663256
rs144574165
rs148390689
rs59102092
rs12968867
rs114723901
rs138920008
rs71555251
rs1524155
rs12636323
rs113612962
rs9350187
rs10192011
rs113747920
rs62463053
rs11643297
rs115952864
rs116412781
All − MR Egger
All − Inverse variance weighted
−4
−2
0
2
MR effect size for
' || id:ebi−a−GCST90001732' on 'siNENs'
MR Method
Inverse variance weighted
MR Egger
1/SE_IV
IV
MR leave−one−out sensitivity analysis for
' || id:ebi−a−GCST90001732' on 'siNENs'
All
MR Estimate
Inverse variance weighted
MR Egger
Simple mode
Weighted median
Weighted mode
SNP effect on siNENs
SNP effect on || id:ebi−a−GCST90001732

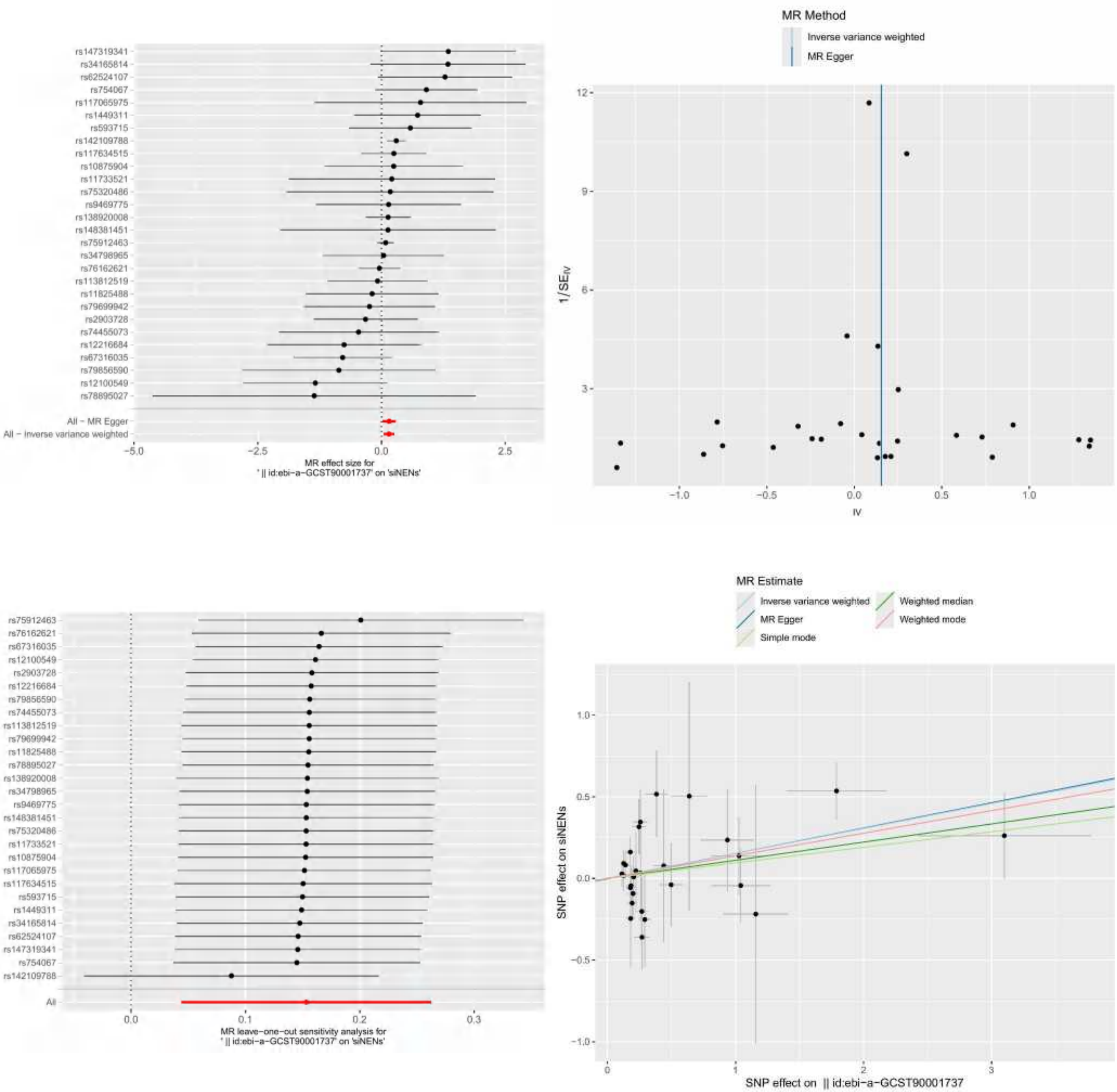

rs147319341
rs34165814
rs62524107
rs754067
rs117065975
rs1449311
rs593715
rs142109788
rs117634515
rs10875904
rs11733521
rs75320486
rs9469775
rs138920008
rs148381451
rs75912463
rs34798965
rs76162621
rs113812519
rs11825488
rs79699942
rs2903728
rs74455073
rs12216684
rs67316035
rs79856590
rs12100549
rs78895027
All − MR Egger
All − Inverse variance weighted
−5.0
−2.5
0.0
2.5
MR effect size for
'|| id:ebi−a−GCST90001737' on 'siNENs'
MR Method
Inverse variance weighted
MR Egger
1/SE$_{IV}$
IV
rs75912463
rs76162621
rs67316035
rs12100549
rs2903728
rs12216684
rs79856590
rs74455073
rs113812519
rs79699942
rs11825488
rs78895027
rs138920008
rs34798965
rs9469775
rs148381451
rs75320486
rs11733521
rs10875904
rs117065975
rs117634515
rs593715
rs1449311
rs34165814
rs62524107
rs147319341
rs754067
rs142109788
All
0.0
0.1
0.2
0.3
MR leave−one−out sensitivity analysis for
'|| id:ebi−a−GCST90001737' on 'siNENs'
MR Estimate
Inverse variance weighted
MR Egger
Simple mode
Weighted median
Weighted mode
SNP effect on siNENs
SNP effect on || id:ebi−a−GCST90001737

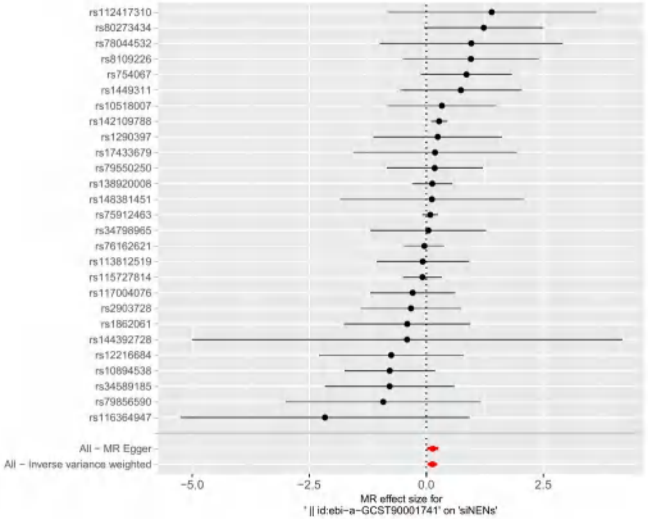
rs112417310
rs80273434
rs78044532
rs8109226
rs754067
rs1449311
rs10518007
rs142109788
rs1290397
rs17433679
rs79550250
rs138920008
rs148381451
rs75912463
rs34798965
rs76162621
rs113812519
rs115727814
rs117004076
rs2903728
rs1862061
rs144392728
rs12216684
rs10894538
rs34589185
rs79856590
rs116364947
All − MR Egger
All − Inverse variance weighted
−5.0
−2.5
0.0
2.5
MR effect size for
' || id:ebi−a−GCST90001741' on 'siNENs'

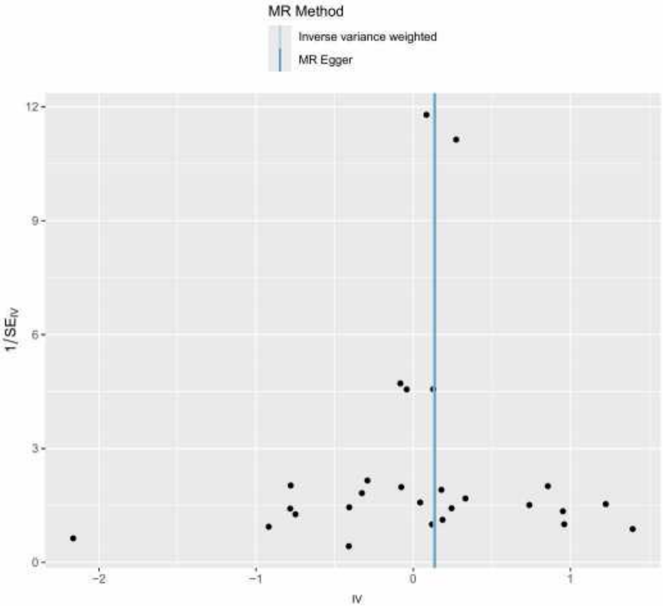
MR Method
Inverse variance weighted
MR Egger
1/SE$_{IV}$
12
9
6
3
0
−2
−1
0
1
IV

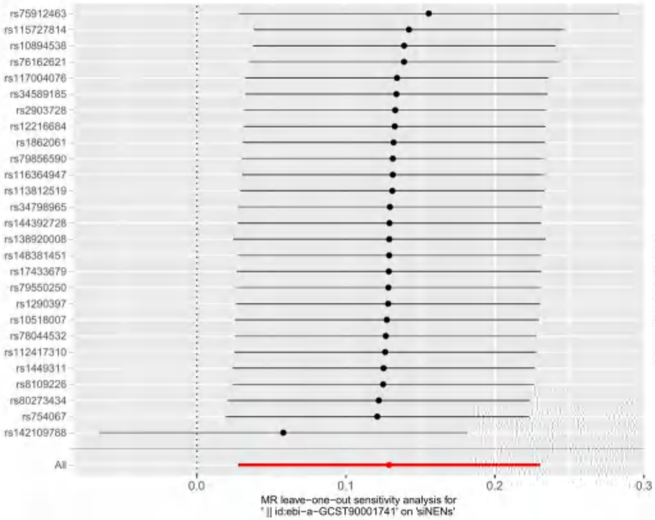
rs75912463
rs115727814
rs10894538
rs76162621
rs117004076
rs34589185
rs2903728
rs12216684
rs1862061
rs79856590
rs116364947
rs113812519
rs34798965
rs144392728
rs138920008
rs148381451
rs17433679
rs79550250
rs1290397
rs10518007
rs78044532
rs112417310
rs1449311
rs8109226
rs80273434
rs754067
rs142109788
All
0.0
0.1
0.2
0.3
MR leave−one−out sensitivity analysis for
' || id:ebi−a−GCST90001741' on 'siNENs'

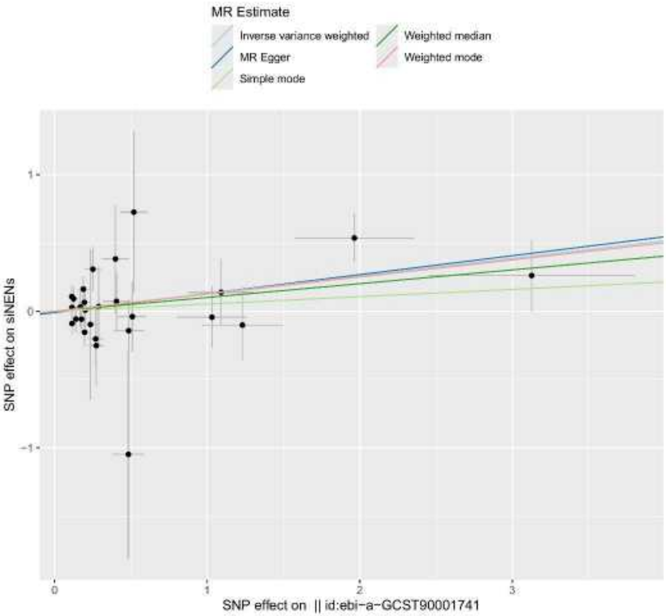
MR Estimate
Inverse variance weighted
MR Egger
Simple mode
Weighted median
Weighted mode
SNP effect on siNENs
1
0
−1
0
1
2
3
SNP effect on || id:ebi−a−GCST90001741

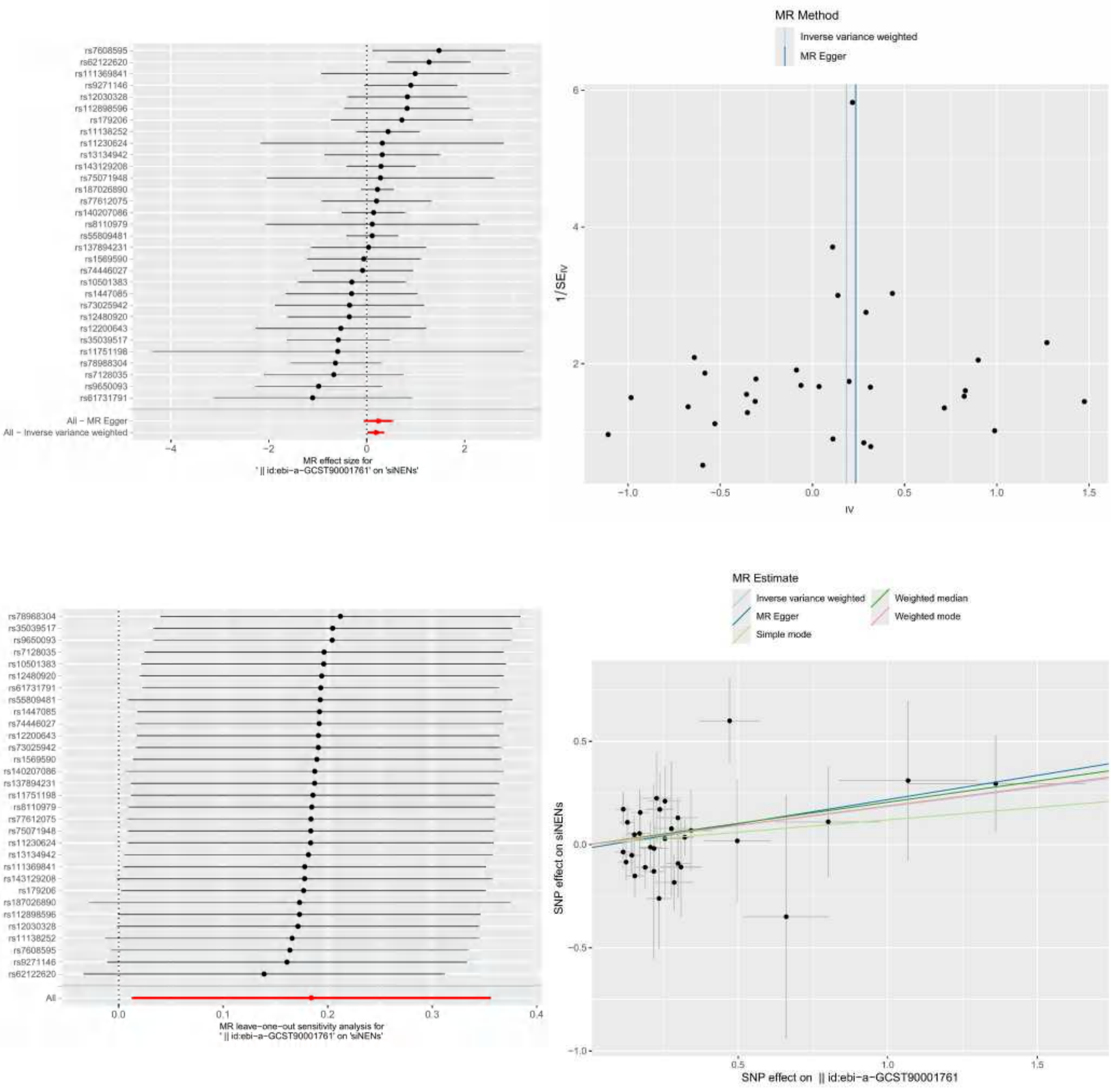

rs7608595
rs62122620
rs111369841
rs9271146
rs12030328
rs112898596
rs179206
rs11138252
rs11230624
rs13134942
rs143129208
rs75071948
rs187026890
rs77612075
rs140207086
rs8110979
rs55809481
rs137894231
rs1569590
rs74446027
rs10501383
rs1447085
rs73025942
rs12480920
rs12200643
rs35039517
rs11751198
rs78988304
rs7128035
rs9650093
rs61731791
All − MR Egger
All − Inverse variance weighted
MR effect size for
' || id:ebi−a−GCST90001761' on 'siNENs'
MR Method
Inverse variance weighted
MR Egger
1/SE$_{IV}$
IV
MR leave−one−out sensitivity analysis for
' || id:ebi−a−GCST90001761' on 'siNENs'
All
MR Estimate
Inverse variance weighted
MR Egger
Simple mode
Weighted median
Weighted mode
SNP effect on siNENs
SNP effect on || id:ebi−a−GCST90001761

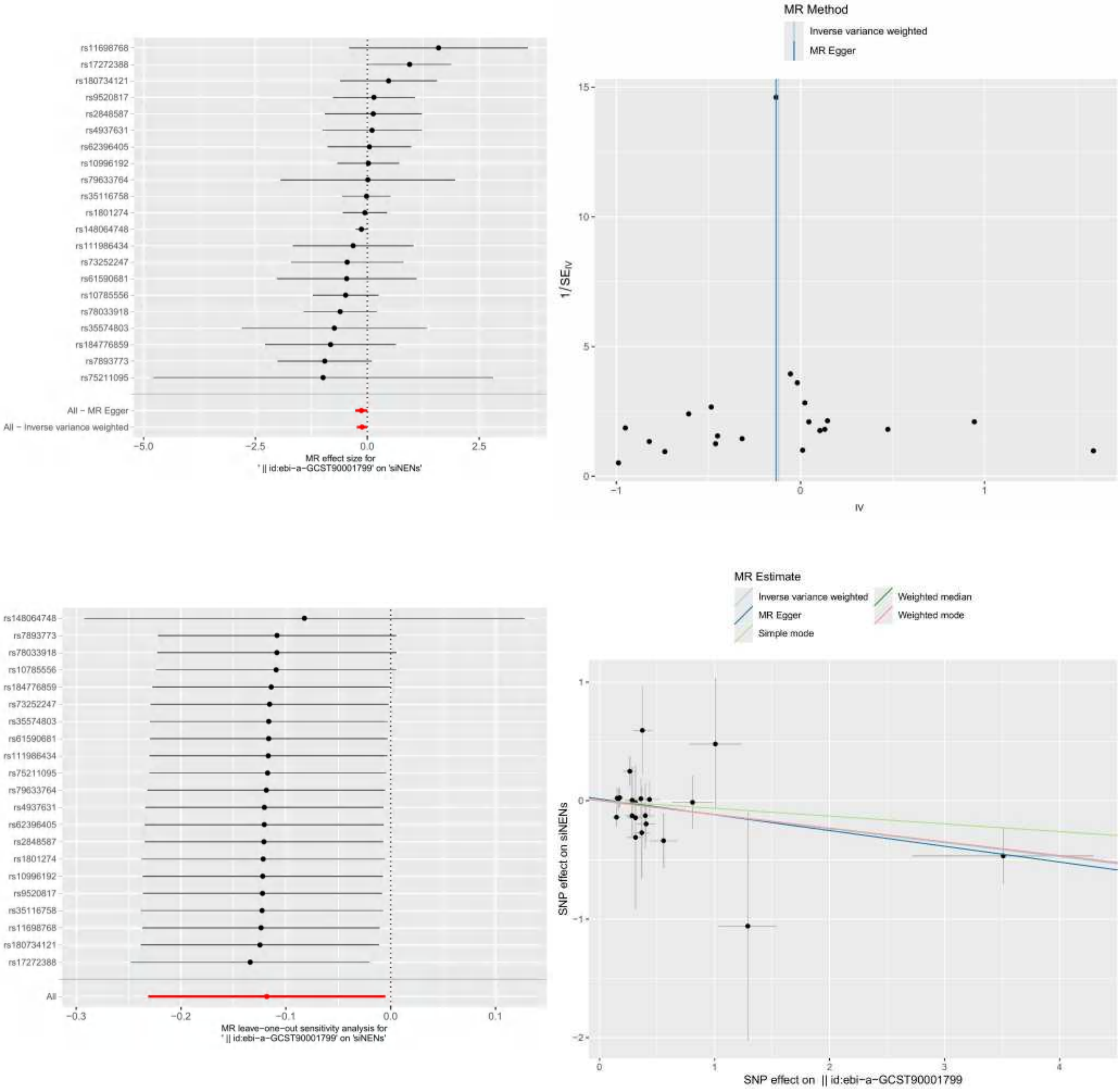
rs11698768
rs17272388
rs180734121
rs9520817
rs2848587
rs4937631
rs62396405
rs10996192
rs79633764
rs35116758
rs1801274
rs148064748
rs111986434
rs73252247
rs61590681
rs10785556
rs78033918
rs35574803
rs184776859
rs7893773
rs75211095
All - MR Egger
All - Inverse variance weighted
-5.0
-2.5
0.0
2.5
MR effect size for
' || id:ebi-a-GCST90001799' on 'siNENs'
MR Method
Inverse variance weighted
MR Egger
15
10
5
0
1/SE_IV
-1
0
1
IV
rs148064748
rs7893773
rs78033918
rs10785556
rs184776859
rs73252247
rs35574803
rs61590681
rs111986434
rs75211095
rs79633764
rs4937631
rs62396405
rs2848587
rs1801274
rs10996192
rs9520817
rs35116758
rs11698768
rs180734121
rs17272388
All
-0.3
-0.2
-0.1
0.0
0.1
MR leave-one-out sensitivity analysis for
' || id:ebi-a-GCST90001799' on 'siNENs'
MR Estimate
Inverse variance weighted
MR Egger
Simple mode
Weighted median
Weighted mode
1
0
-1
-2
SNP effect on siNENs
0
1
2
3
4
SNP effect on || id:ebi-a-GCST90001799

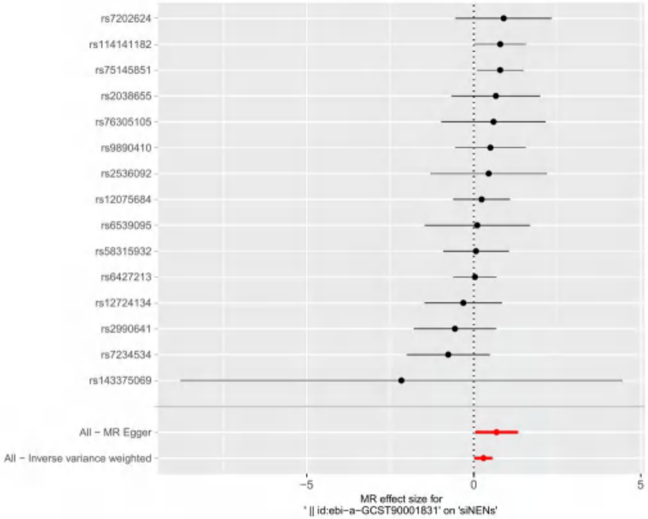
rs7202624
rs114141182
rs75145851
rs2038655
rs76305105
rs9890410
rs2536092
rs12075684
rs6539095
rs58315932
rs6427213
rs12724134
rs2990641
rs7234534
rs143375069
All − MR Egger
All − Inverse variance weighted
−5
0
5
MR effect size for
' || id:ebi−a−GCST90001831' on 'siNENs'

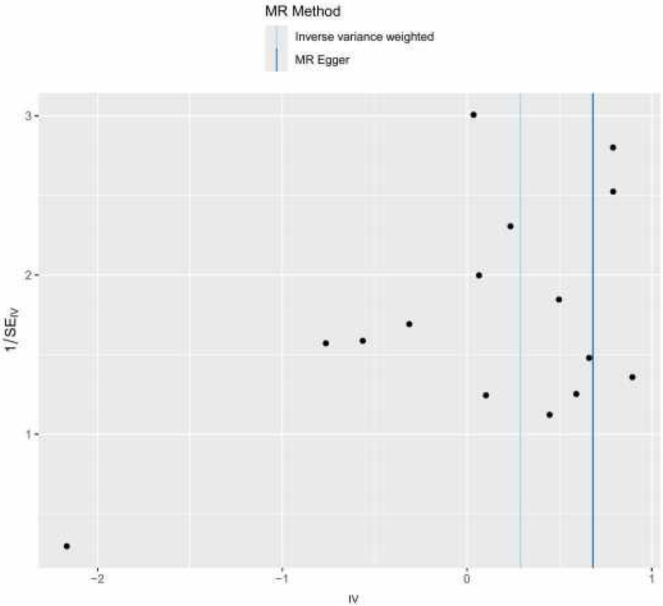
MR Method
Inverse variance weighted
MR Egger
3
2
1
1/SE$_{IV}$
−2
−1
0
1
IV

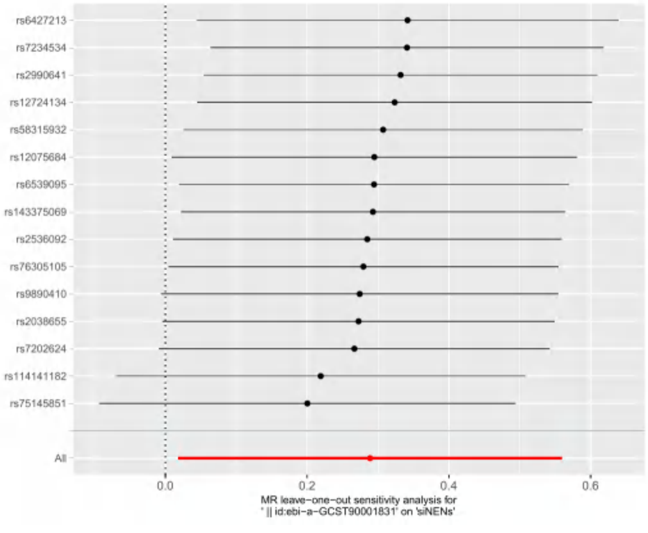
rs6427213
rs7234534
rs2990641
rs12724134
rs58315932
rs12075684
rs6539095
rs143375069
rs2536092
rs76305105
rs9890410
rs2038655
rs7202624
rs114141182
rs75145851
All
0.0
0.2
0.4
0.6
MR leave−one−out sensitivity analysis for
' || id:ebi−a−GCST90001831' on 'siNENs'

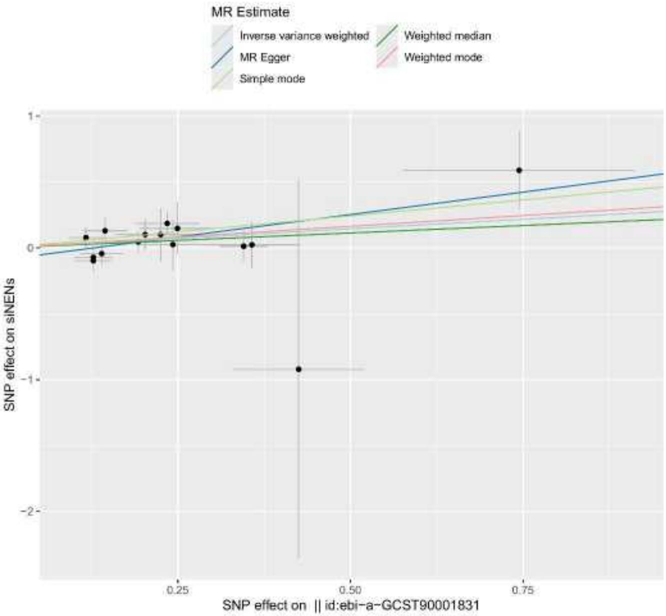
MR Estimate
Inverse variance weighted
MR Egger
Simple mode
Weighted median
Weighted mode
1
0
−1
−2
SNP effect on siNENs
0.25
0.50
0.75
SNP effect on || id:ebi−a−GCST90001831

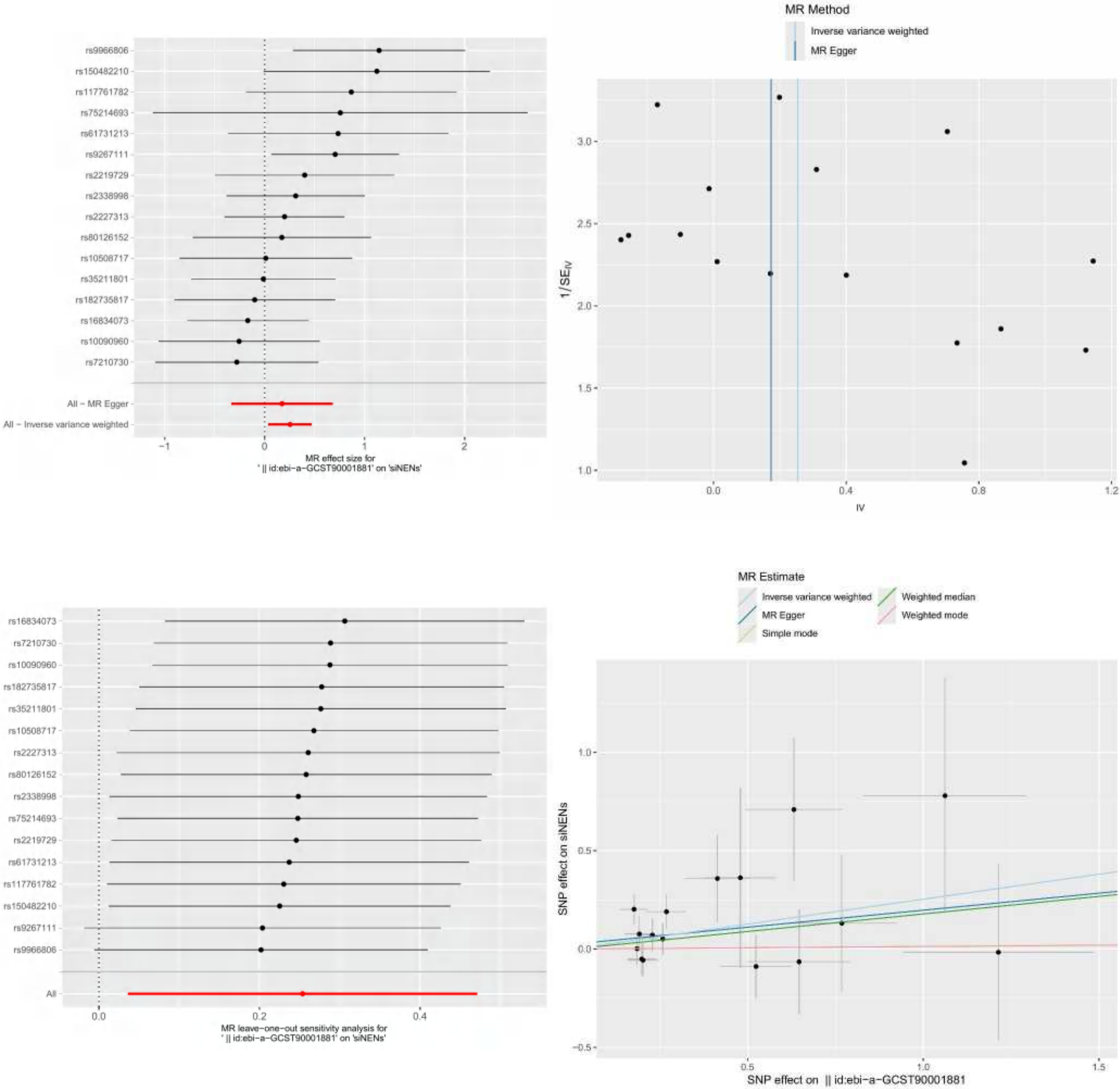

rs9966806
rs150482210
rs117761782
rs75214693
rs61731213
rs9267111
rs2219729
rs2338998
rs2227313
rs80126152
rs10508717
rs35211801
rs182735817
rs16834073
rs10090960
rs7210730
All − MR Egger
All − Inverse variance weighted
MR effect size for
' || id:ebi−a−GCST90001881' on 'siNENs'
MR Method
Inverse variance weighted
MR Egger
1/SE_IV
IV
rs16834073
rs7210730
rs10090960
rs182735817
rs35211801
rs10508717
rs2227313
rs80126152
rs2338998
rs75214693
rs2219729
rs61731213
rs117761782
rs150482210
rs9267111
rs9966806
All
MR leave−one−out sensitivity analysis for
' || id:ebi−a−GCST90001881' on 'siNENs'
MR Estimate
Inverse variance weighted
MR Egger
Simple mode
Weighted median
Weighted mode
SNP effect on siNENs
SNP effect on || id:ebi−a−GCST90001881

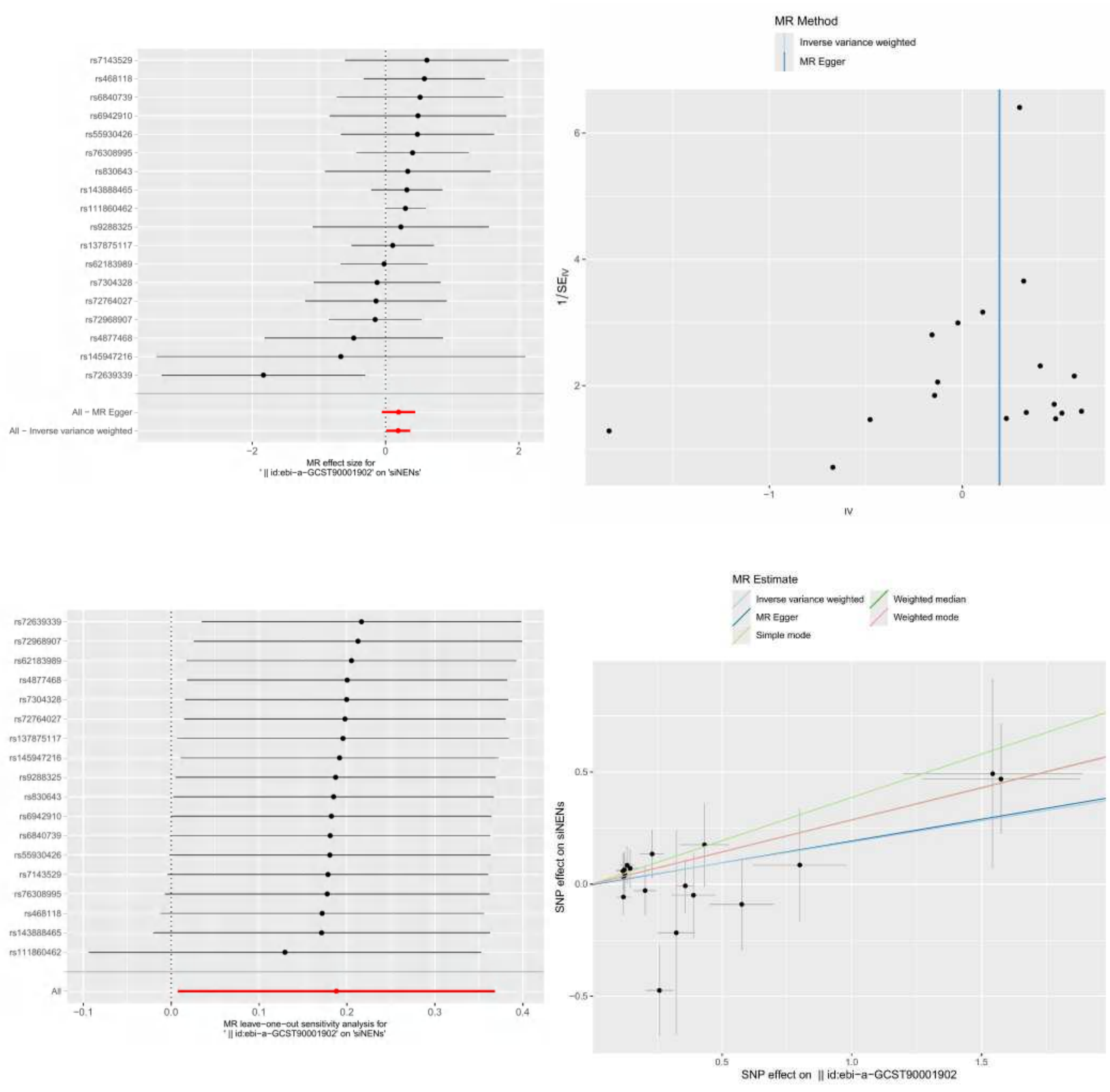

rs7143529
rs468118
rs6840739
rs6942910
rs55930426
rs76308995
rs830643
rs143888465
rs111860462
rs9288325
rs137875117
rs62183989
rs7304328
rs72764027
rs72968907
rs4877468
rs145947216
rs72639339
All − MR Egger
All − Inverse variance weighted
−2
0
2
MR effect size for
' || id:ebi−a−GCST90001902' on 'siNENs'
MR Method
Inverse variance weighted
MR Egger
1/SE_IV
6
4
2
−1
0
IV
rs72639339
rs72968907
rs62183989
rs4877468
rs7304328
rs72764027
rs137875117
rs145947216
rs9288325
rs830643
rs6942910
rs6840739
rs55930426
rs7143529
rs76308995
rs468118
rs143888465
rs111860462
All
−0.1
0.0
0.1
0.2
0.3
0.4
MR leave−one−out sensitivity analysis for
' || id:ebi−a−GCST90001902' on 'siNENs'
MR Estimate
Inverse variance weighted
MR Egger
Simple mode
Weighted median
Weighted mode
SNP effect on siNENs
0.5
0.0
−0.5
1.0
1.5
SNP effect on || id:ebi−a−GCST90001902

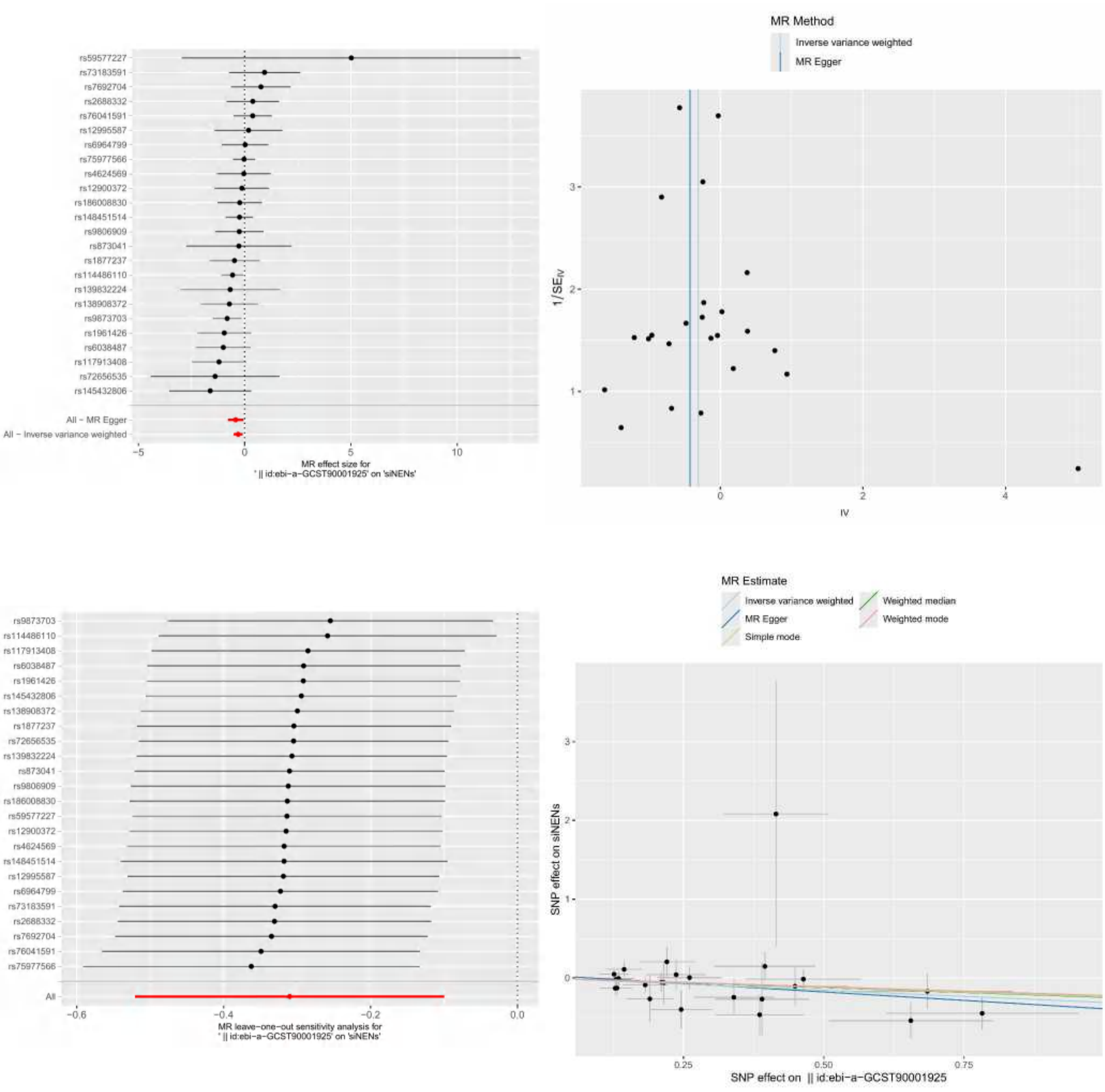

rs59577227
rs73183591
rs7692704
rs2688332
rs76041591
rs12995587
rs6964799
rs75977566
rs4624569
rs12900372
rs186008830
rs148451514
rs9806909
rs873041
rs1877237
rs114486110
rs139832224
rs138908372
rs9873703
rs1961426
rs6038487
rs117913408
rs72656535
rs145432806
All - MR Egger
All - Inverse variance weighted
-5
0
5
10
MR effect size for
' || id:ebi-a-GCST90001925' on 'siNENs'
MR Method
Inverse variance weighted
MR Egger
1/SE_IV
3
2
1
0
2
4
IV
rs9873703
rs114486110
rs117913408
rs6038487
rs1961426
rs145432806
rs138908372
rs1877237
rs72656535
rs139832224
rs873041
rs9806909
rs186008830
rs59577227
rs12900372
rs4624569
rs148451514
rs12995587
rs6964799
rs73183591
rs2688332
rs7692704
rs76041591
rs75977566
All
-0.6
-0.4
-0.2
0.0
MR leave-one-out sensitivity analysis for
' || id:ebi-a-GCST90001925' on 'siNENs'
MR Estimate
Inverse variance weighted
MR Egger
Simple mode
Weighted median
Weighted mode
SNP effect on siNENs
3
2
1
0
0.25
0.50
0.75
SNP effect on || id:ebi-a-GCST90001925

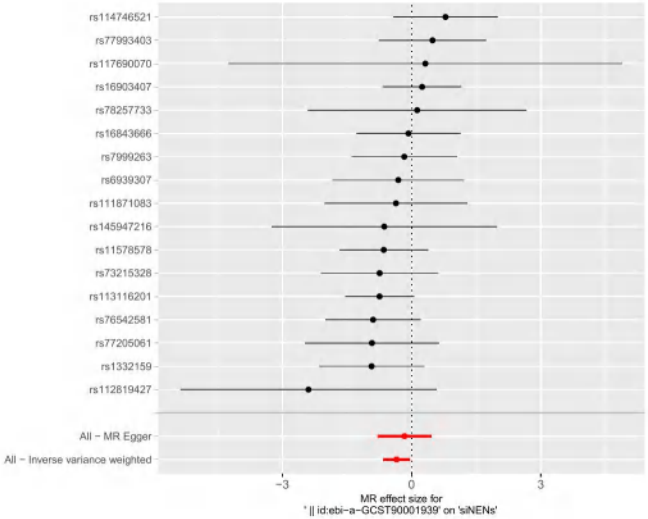
rs114746521
rs77993403
rs117690070
rs16903407
rs78257733
rs16843666
rs7999263
rs6939307
rs111871083
rs145947216
rs11578578
rs73215328
rs113116201
rs76542581
rs77205061
rs1332159
rs112819427
All − MR Egger
All − Inverse variance weighted
-3
0
3
MR effect size for
' || id:ebi−a−GCST90001939' on 'siNENs'

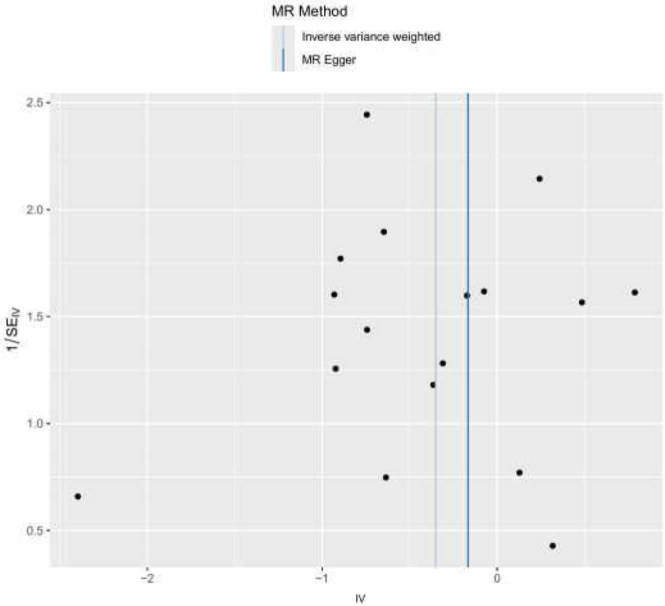
MR Method
Inverse variance weighted
MR Egger
2.5
2.0
1.5
1.0
0.5
1/SE_IV
-2
-1
0
IV

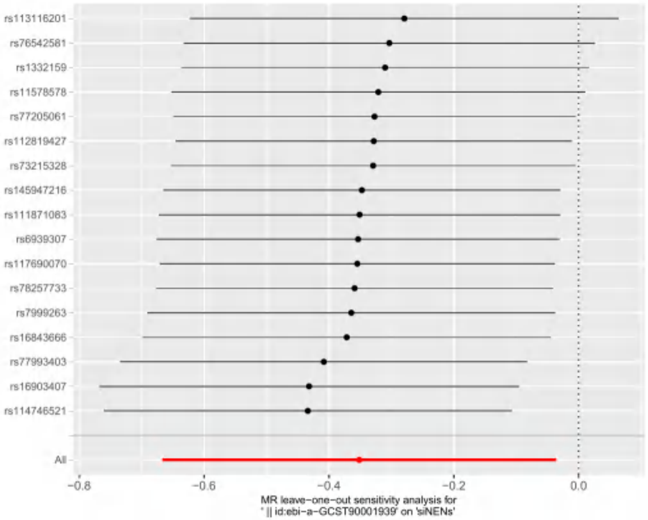
rs113116201
rs76542581
rs1332159
rs11578578
rs77205061
rs112819427
rs73215328
rs145947216
rs111871083
rs6939307
rs117690070
rs78257733
rs7999263
rs16843666
rs77993403
rs16903407
rs114746521
All
-0.8
-0.6
-0.4
-0.2
0.0
MR leave−one−out sensitivity analysis for
' || id:ebi−a−GCST90001939' on 'siNENs'

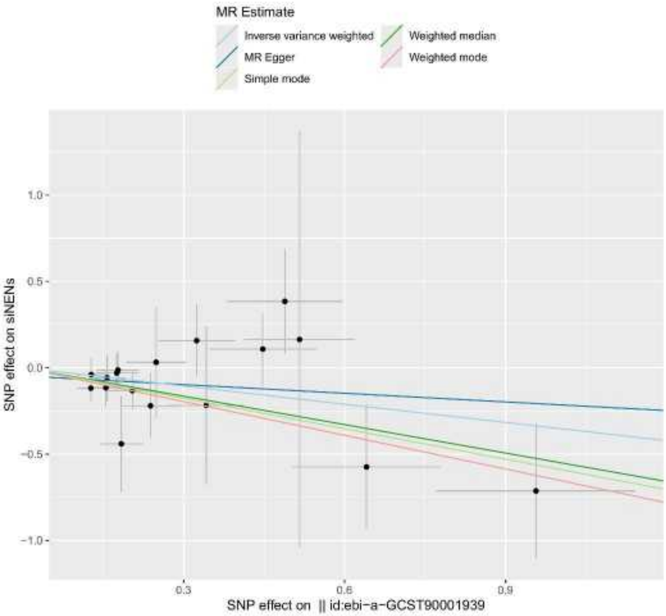
MR Estimate
Inverse variance weighted
MR Egger
Simple mode
Weighted median
Weighted mode
1.0
0.5
0.0
-0.5
-1.0
SNP effect on siNENs
0.3
0.6
0.9
SNP effect on || id:ebi−a−GCST90001939

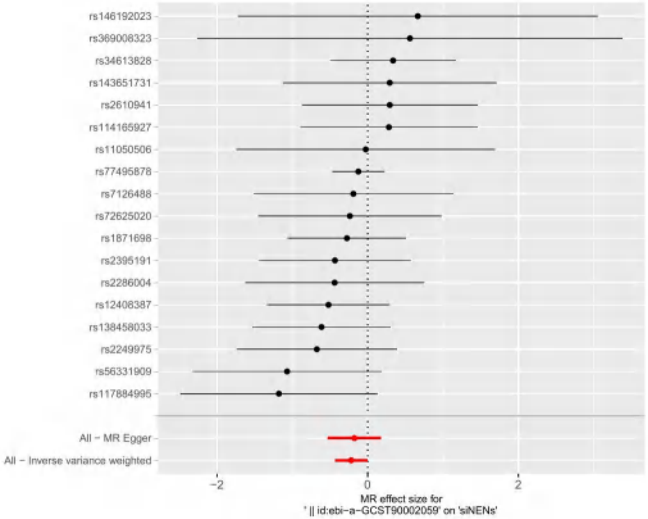
rs146192023
rs369008323
rs34613828
rs143651731
rs2610941
rs114165927
rs11050506
rs77495878
rs7126488
rs72625020
rs1871698
rs2395191
rs2286004
rs12408387
rs138458033
rs2249975
rs56331909
rs117884995
All − MR Egger
All − Inverse variance weighted
−2
0
2
MR effect size for
' || id:ebi−a−GCST90002059' on 'siNENs'

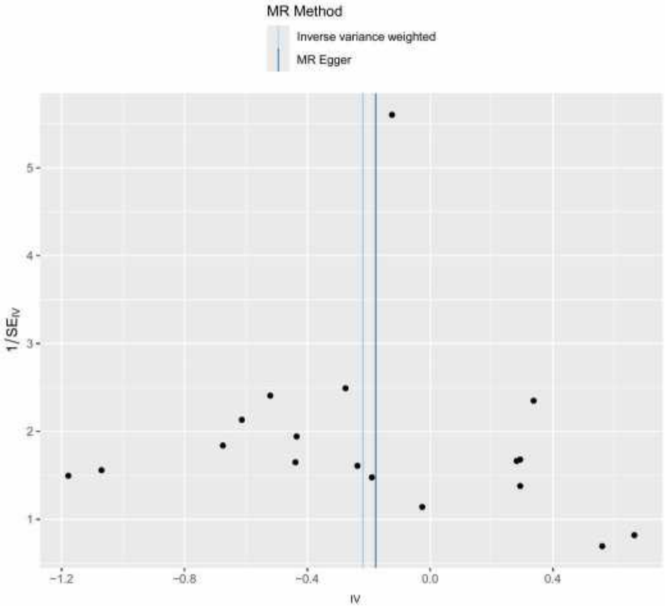
MR Method
Inverse variance weighted
MR Egger
1/SE$_{IV}$
5
4
3
2
1
−1.2
−0.8
−0.4
0.0
0.4
IV

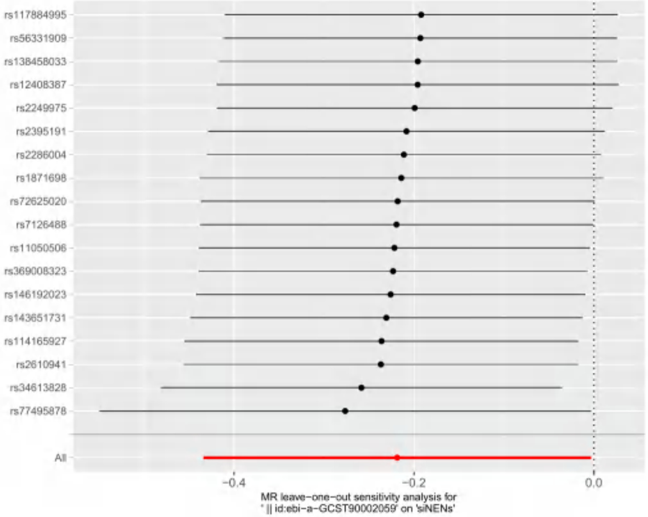
rs117884995
rs56331909
rs138458033
rs12408387
rs2249975
rs2395191
rs2286004
rs1871698
rs72625020
rs7126488
rs11050506
rs369008323
rs146192023
rs143651731
rs114165927
rs2610941
rs34613828
rs77495878
All
−0.4
−0.2
0.0
MR leave−one−out sensitivity analysis for
' || id:ebi−a−GCST90002059' on 'siNENs'

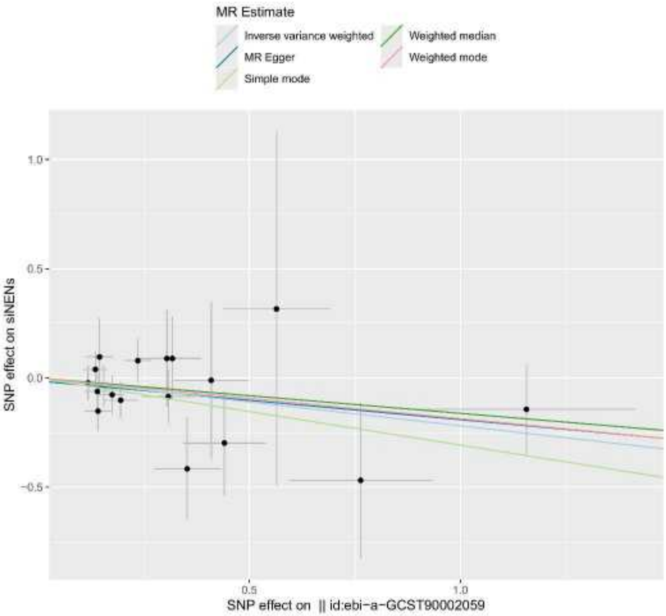
MR Estimate
Inverse variance weighted
MR Egger
Simple mode
Weighted median
Weighted mode
SNP effect on siNENs
1.0
0.5
0.0
−0.5
0.5
1.0
SNP effect on || id:ebi−a−GCST90002059

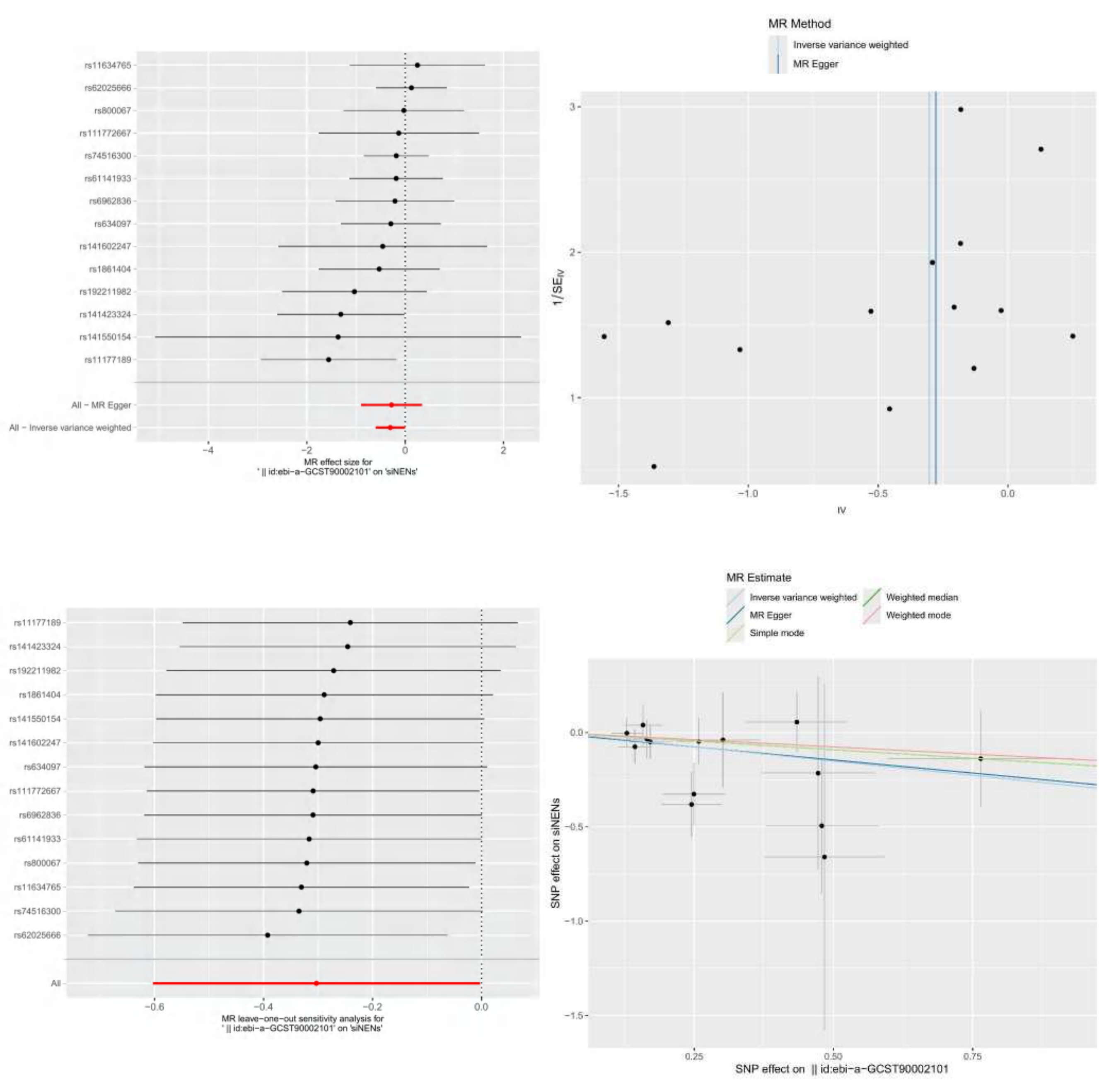
rs11634765
rs62025666
rs800067
rs111772667
rs74516300
rs61141933
rs6962836
rs634097
rs141602247
rs1861404
rs192211982
rs141423324
rs141550154
rs11177189
All − MR Egger
All − Inverse variance weighted
−4
−2
0
2
MR effect size for
' || id:ebi−a−GCST90002101' on 'siNENs'
MR Method
Inverse variance weighted
MR Egger
1/SE_IV
3
2
1
−1.5
−1.0
−0.5
0.0
IV
rs11177189
rs141423324
rs192211982
rs1861404
rs141550154
rs141602247
rs634097
rs111772667
rs6962836
rs61141933
rs800067
rs11634765
rs74516300
rs62025666
All
−0.6
−0.4
−0.2
0.0
MR leave−one−out sensitivity analysis for
' || id:ebi−a−GCST90002101' on 'siNENs'
MR Estimate
Inverse variance weighted
MR Egger
Simple mode
Weighted median
Weighted mode
SNP effect on siNENs
0.0
−0.5
−1.0
−1.5
0.25
0.50
0.75
SNP effect on || id:ebi−a−GCST90002101

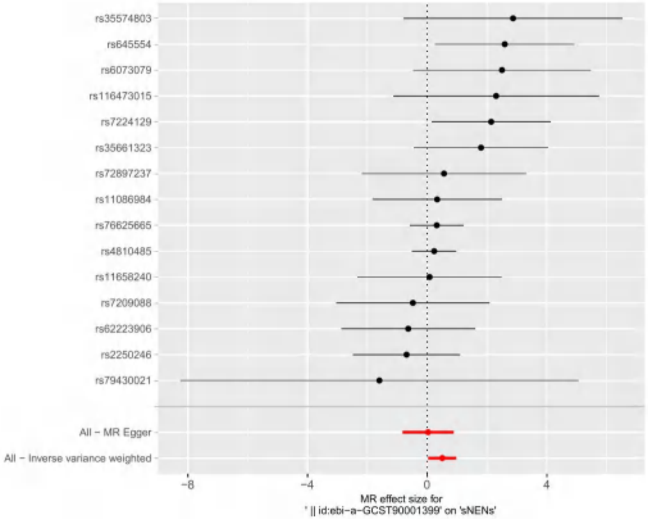
rs35574803
rs645554
rs6073079
rs116473015
rs7224129
rs35661323
rs72897237
rs11086984
rs76625665
rs4810485
rs11658240
rs7209088
rs62223906
rs2250246
rs79430021
All − MR Egger
All − Inverse variance weighted
−8
−4
0
4
MR effect size for
' || id:ebi−a−GCST90001399' on 'sNENs'

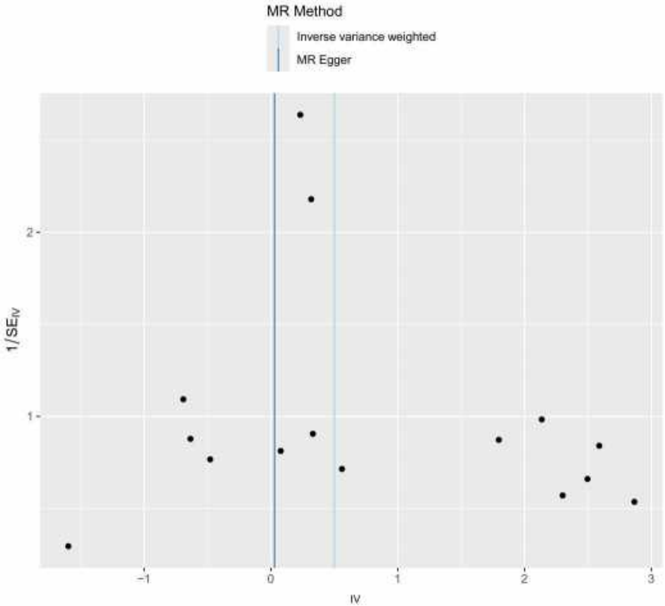
MR Method
Inverse variance weighted
MR Egger
1/SE$_{IV}$
2
1
−1
0
1
2
3
IV

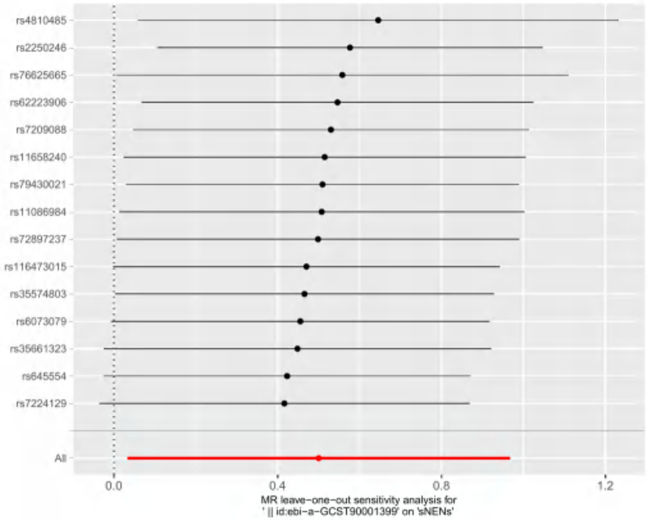
rs4810485
rs2250246
rs76625665
rs62223906
rs7209088
rs11658240
rs79430021
rs11086984
rs72897237
rs116473015
rs35574803
rs6073079
rs35661323
rs645554
rs7224129
All
0.0
0.4
0.8
1.2
MR leave−one−out sensitivity analysis for
' || id:ebi−a−GCST90001399' on 'sNENs'

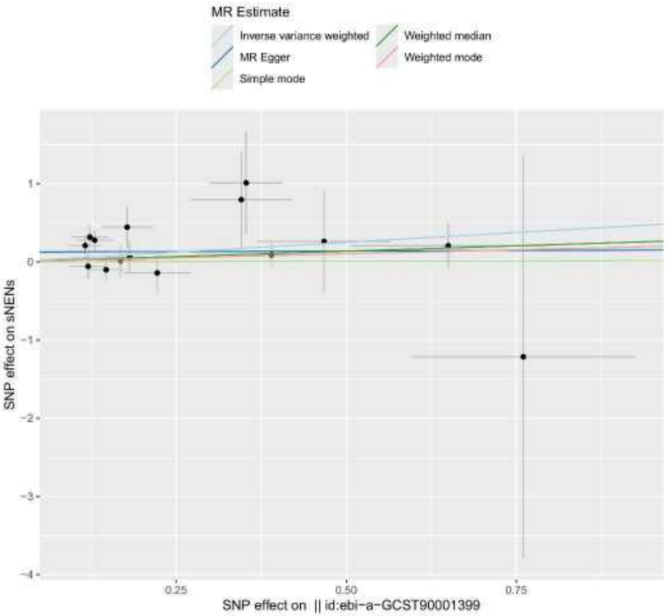
MR Estimate
Inverse variance weighted
MR Egger
Simple mode
Weighted median
Weighted mode
SNP effect on sNENs
1
0
−1
−2
−3
−4
0.25
0.50
0.75
SNP effect on || id:ebi−a−GCST90001399

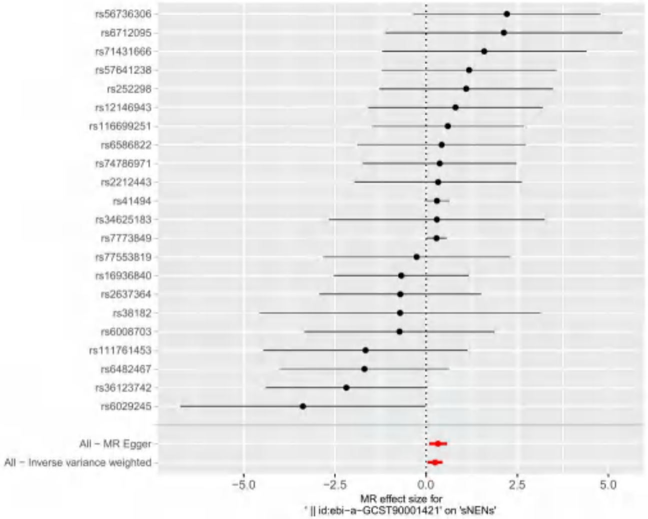
rs56736306
rs6712095
rs71431666
rs57641238
rs252298
rs12146943
rs116699251
rs6586822
rs74786971
rs2212443
rs41494
rs34625183
rs7773849
rs77553819
rs16936840
rs2637364
rs38182
rs6008703
rs111761453
rs6482467
rs36123742
rs6029245
All – MR Egger
All – Inverse variance weighted
-5.0
-2.5
0.0
2.5
5.0
MR effect size for
' || id:ebi-a-GCST90001421' on 'sNENs'

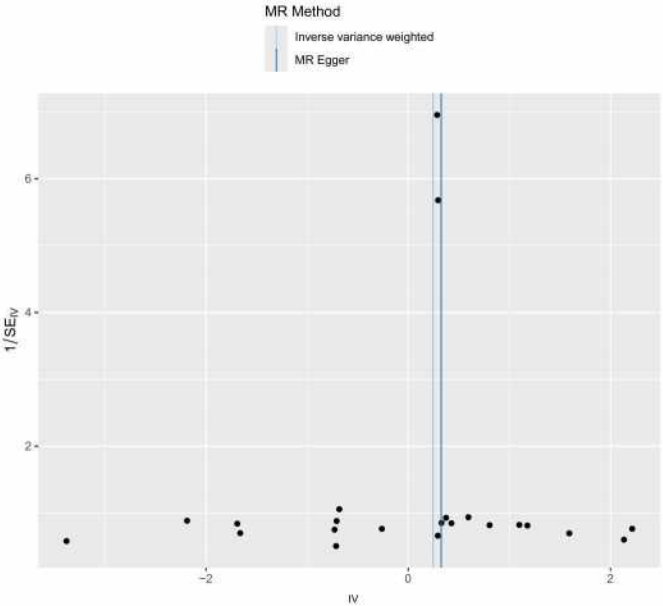
MR Method
Inverse variance weighted
MR Egger
$1/SE_{IV}$
6
4
2
-2
0
2
IV

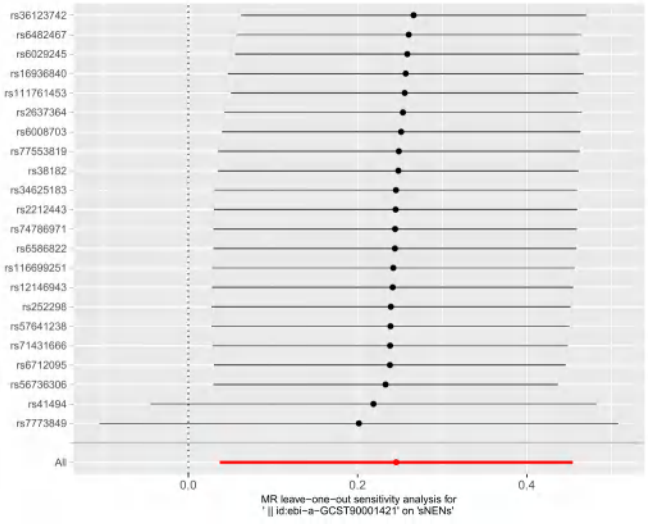
rs36123742
rs6482467
rs6029245
rs16936840
rs111761453
rs2637364
rs6008703
rs77553819
rs38182
rs34625183
rs2212443
rs74786971
rs6586822
rs116699251
rs12146943
rs252298
rs57641238
rs71431666
rs6712095
rs56736306
rs41494
rs7773849
All
0.0
0.2
0.4
MR leave-one-out sensitivity analysis for
' || id:ebi-a-GCST90001421' on 'sNENs'

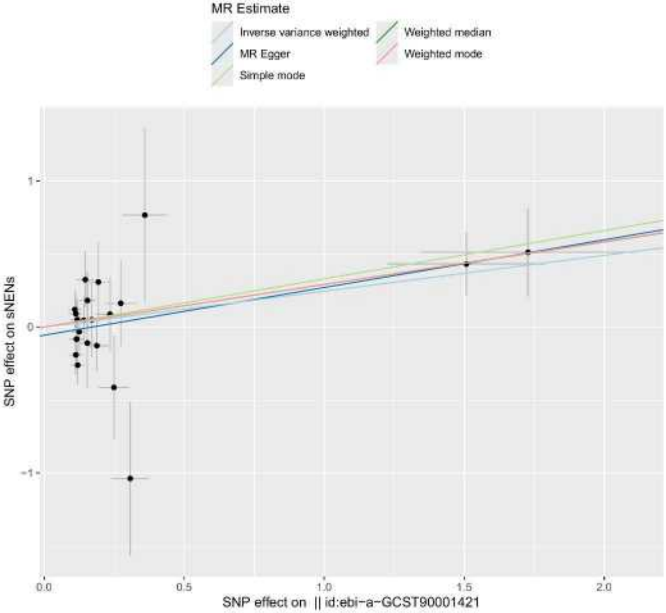
MR Estimate
Inverse variance weighted
MR Egger
Simple mode
Weighted median
Weighted mode
SNP effect on sNENs
1
0
-1
0.0
0.5
1.0
1.5
2.0
SNP effect on || id:ebi-a-GCST90001421

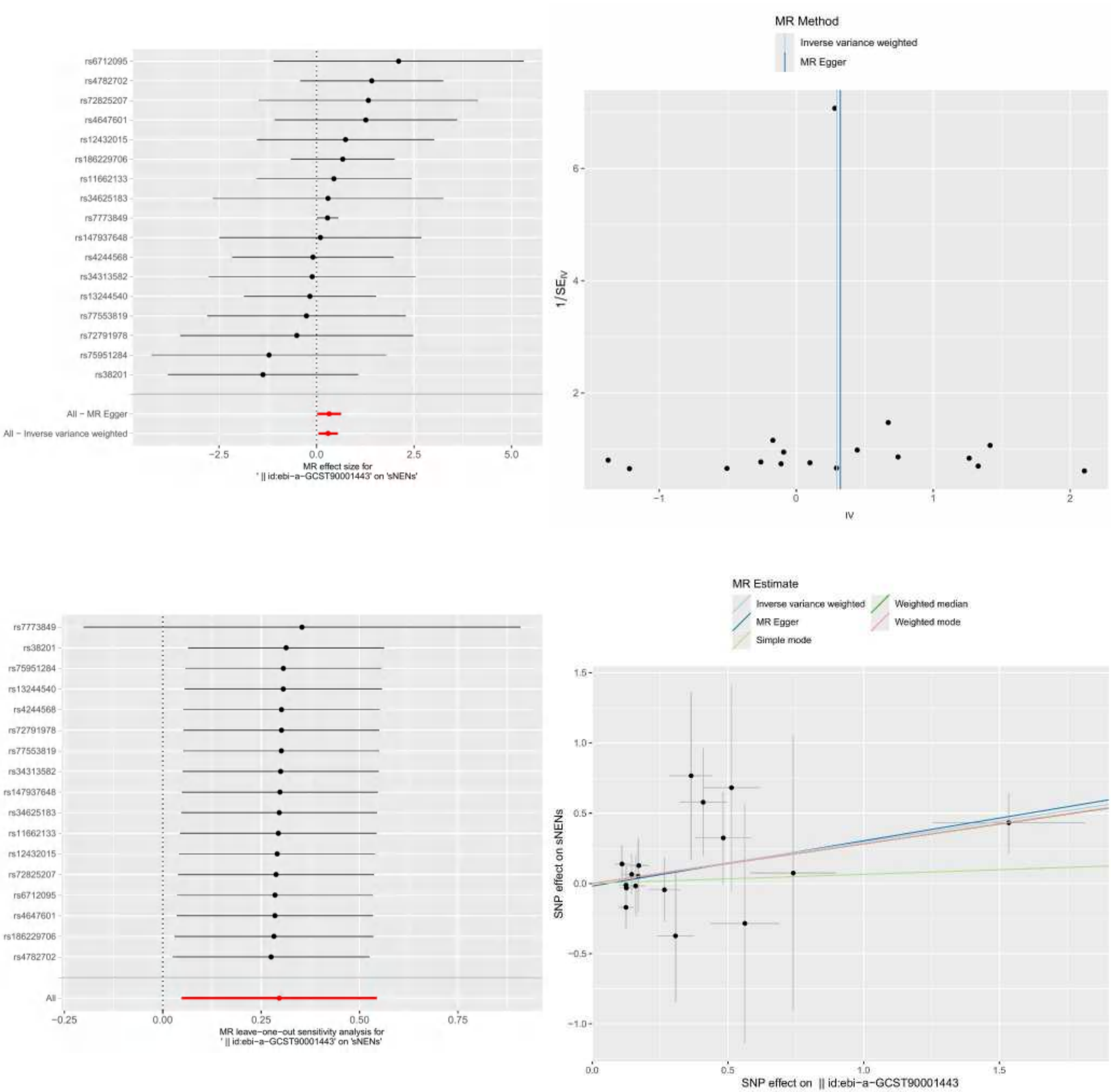

rs6712095
rs4782702
rs72825207
rs4647601
rs12432015
rs186229706
rs11662133
rs34625183
rs7773849
rs147937648
rs4244568
rs34313582
rs13244540
rs77553819
rs72791978
rs75951284
rs38201
All − MR Egger
All − Inverse variance weighted
−2.5
0.0
2.5
5.0
MR effect size for
' || id:ebi−a−GCST90001443' on 'sNENs'
MR Method
Inverse variance weighted
MR Egger
1/SE$_{IV}$
IV
MR leave−one−out sensitivity analysis for
' || id:ebi−a−GCST90001443' on 'sNENs'
MR Estimate
Inverse variance weighted
MR Egger
Simple mode
Weighted median
Weighted mode
SNP effect on sNENs
SNP effect on || id:ebi−a−GCST90001443

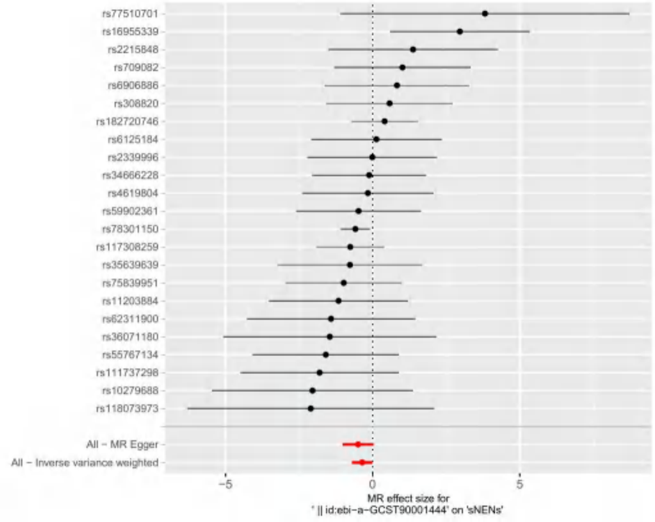
rs77510701
rs16955339
rs2215848
rs709082
rs6906886
rs308820
rs182720746
rs6125184
rs2339996
rs34666228
rs4619804
rs59902361
rs78301150
rs117308259
rs35639639
rs75839951
rs11203884
rs62311900
rs36071180
rs55767134
rs111737298
rs10279688
rs118073973
All − MR Egger
All − Inverse variance weighted
−5
0
5
MR effect size for
' || id:ebi−a−GCST90001444' on 'sNENs'

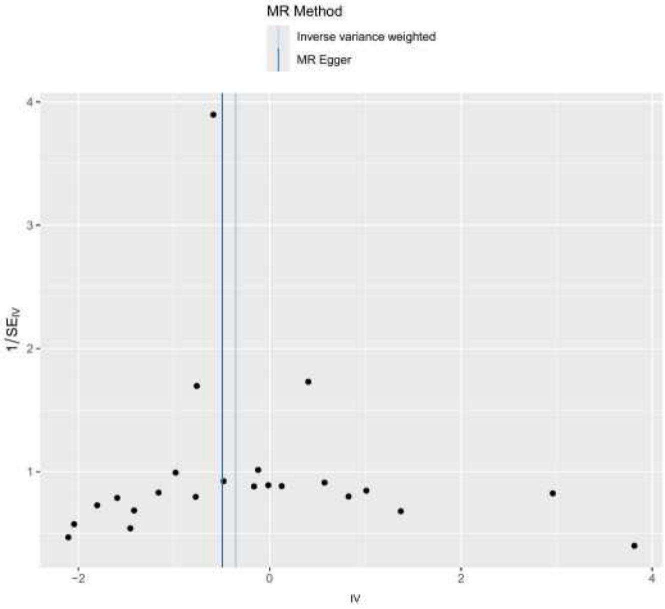
MR Method
Inverse variance weighted
MR Egger
4
3
2
1
1/SE$_{IV}$
−2
0
2
4
IV

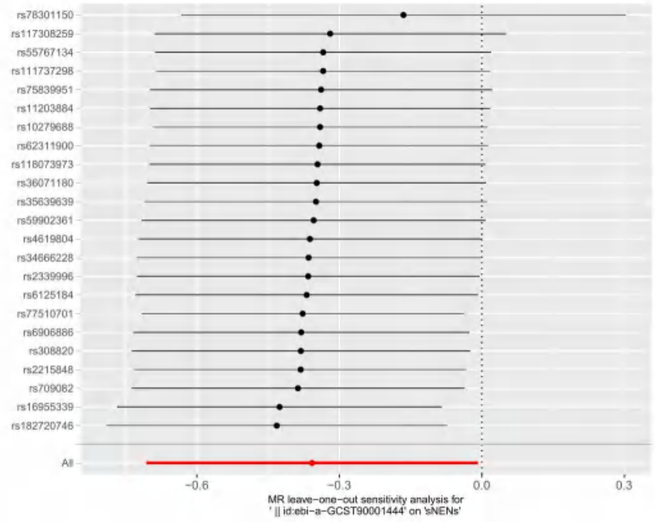
rs78301150
rs117308259
rs55767134
rs111737298
rs75839951
rs11203884
rs10279688
rs62311900
rs118073973
rs36071180
rs35639639
rs59902361
rs4619804
rs34666228
rs2339996
rs6125184
rs77510701
rs6906886
rs308820
rs2215848
rs709082
rs16955339
rs182720746
All
−0.6
−0.3
0.0
0.3
MR leave−one−out sensitivity analysis for
' || id:ebi−a−GCST90001444' on 'sNENs'

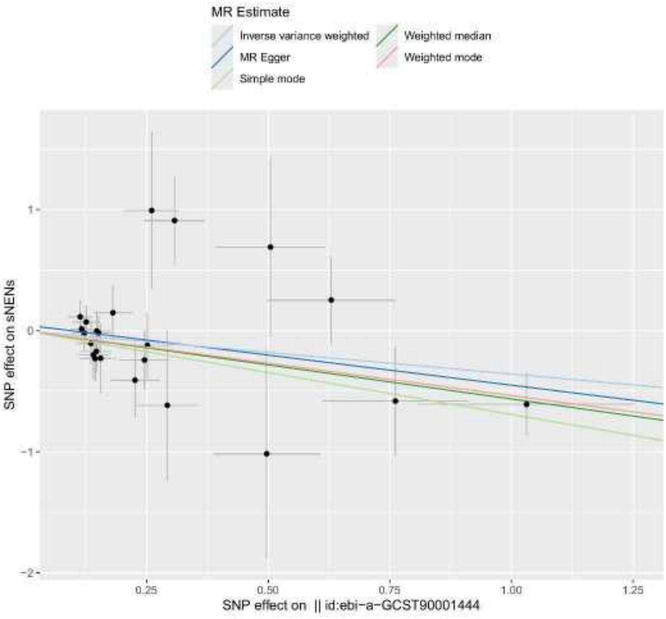
MR Estimate
Inverse variance weighted
MR Egger
Simple mode
Weighted median
Weighted mode
1
0
−1
−2
SNP effect on sNENs
0.25
0.50
0.75
1.00
1.25
SNP effect on || id:ebi−a−GCST90001444

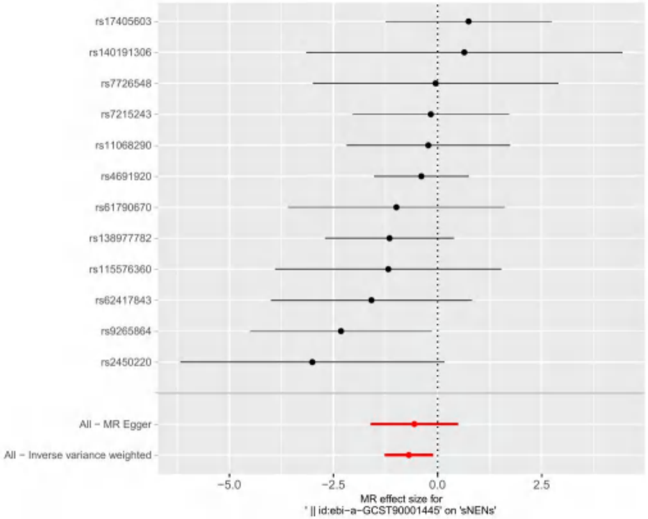
rs17405603
rs140191306
rs7726548
rs7215243
rs11068290
rs4691920
rs61790670
rs138977782
rs115576360
rs62417843
rs9265864
rs2450220
All − MR Egger
All − Inverse variance weighted
−5.0
−2.5
0.0
2.5
MR effect size for
' || id:ebi−a−GCST90001445' on 'sNENs'

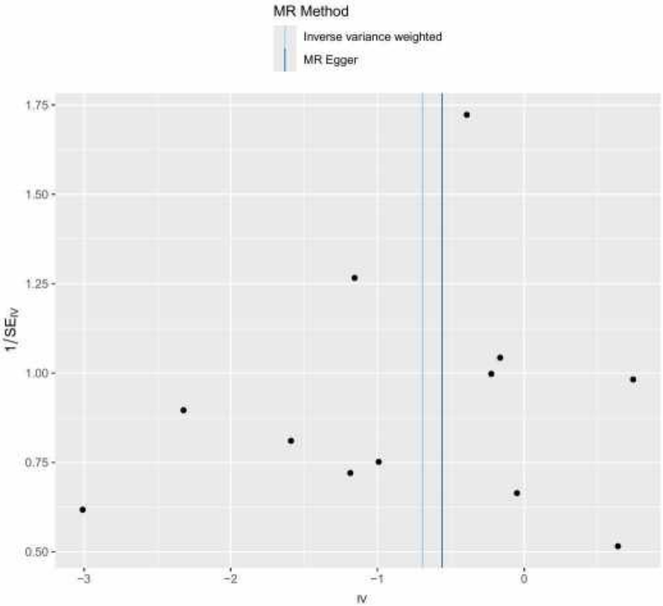
MR Method
Inverse variance weighted
MR Egger
1.75
1.50
1.25
1.00
0.75
0.50
$1/SE_{IV}$
−3
−2
−1
0
IV

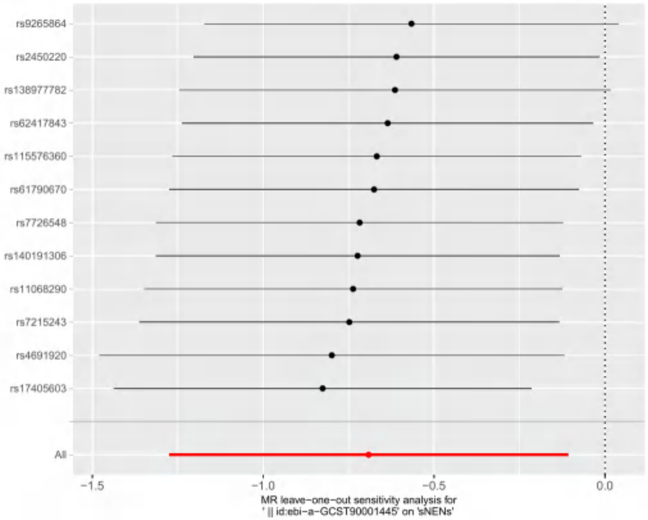
rs9265864
rs2450220
rs138977782
rs62417843
rs115576360
rs61790670
rs7726548
rs140191306
rs11068290
rs7215243
rs4691920
rs17405603
All
−1.5
−1.0
−0.5
0.0
MR leave−one−out sensitivity analysis for
' || id:ebi−a−GCST90001445' on 'sNENs'

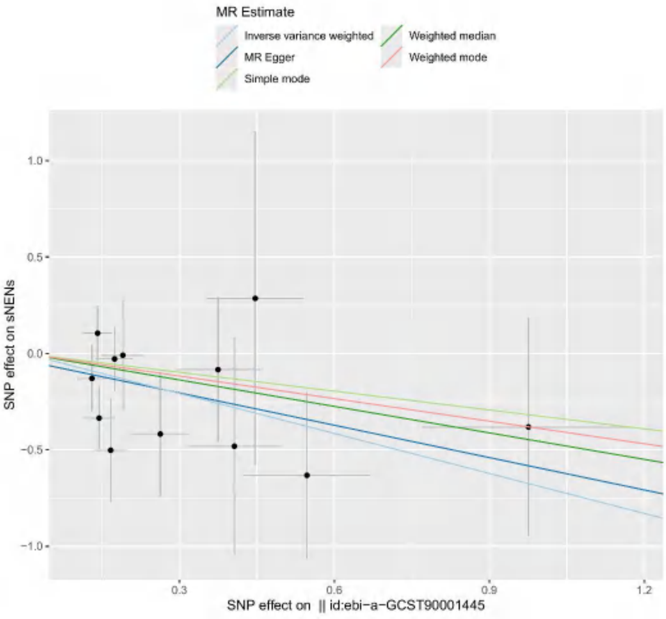
MR Estimate
Inverse variance weighted
MR Egger
Simple mode
Weighted median
Weighted mode
1.0
0.5
0.0
−0.5
−1.0
SNP effect on sNENs
0.3
0.6
0.9
1.2
SNP effect on || id:ebi−a−GCST90001445

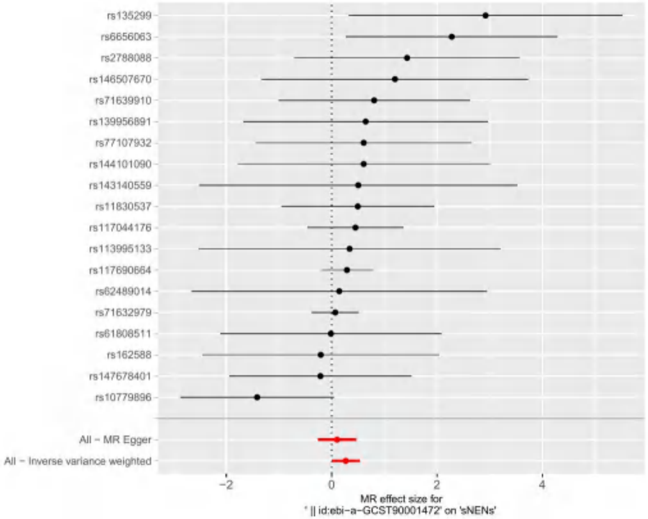
rs135299
rs6656063
rs2788088
rs146507670
rs71639910
rs139956891
rs77107932
rs144101090
rs143140559
rs11830537
rs117044176
rs113995133
rs117690664
rs62489014
rs71632979
rs61808511
rs162588
rs147678401
rs10779896
All − MR Egger
All − Inverse variance weighted
−2
0
2
4
MR effect size for
' || id:ebi−a−GCST90001472' on 'sNENs'

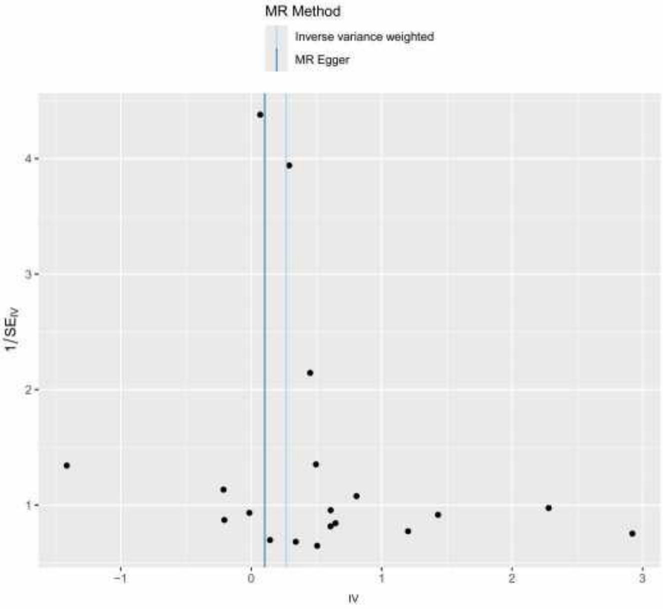
MR Method
Inverse variance weighted
MR Egger
1/SE_IV
1
2
3
4
−1
0
1
2
3
IV

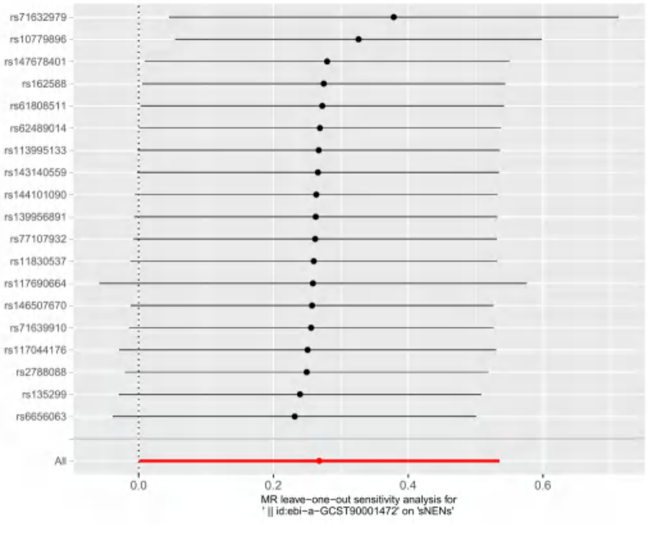
rs71632979
rs10779896
rs147678401
rs162588
rs61808511
rs62489014
rs113995133
rs143140559
rs144101090
rs139956891
rs77107932
rs11830537
rs117690664
rs146507670
rs71639910
rs117044176
rs2788088
rs135299
rs6656063
All
0.0
0.2
0.4
0.6
MR leave−one−out sensitivity analysis for
' || id:ebi−a−GCST90001472' on 'sNENs'

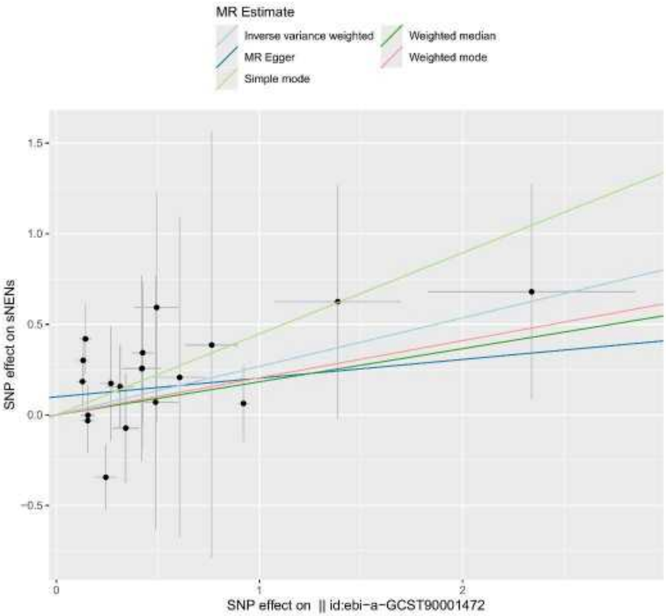
MR Estimate
Inverse variance weighted
MR Egger
Simple mode
Weighted median
Weighted mode
SNP effect on sNENs
1.5
1.0
0.5
0.0
−0.5
0
1
2
SNP effect on || id:ebi−a−GCST90001472

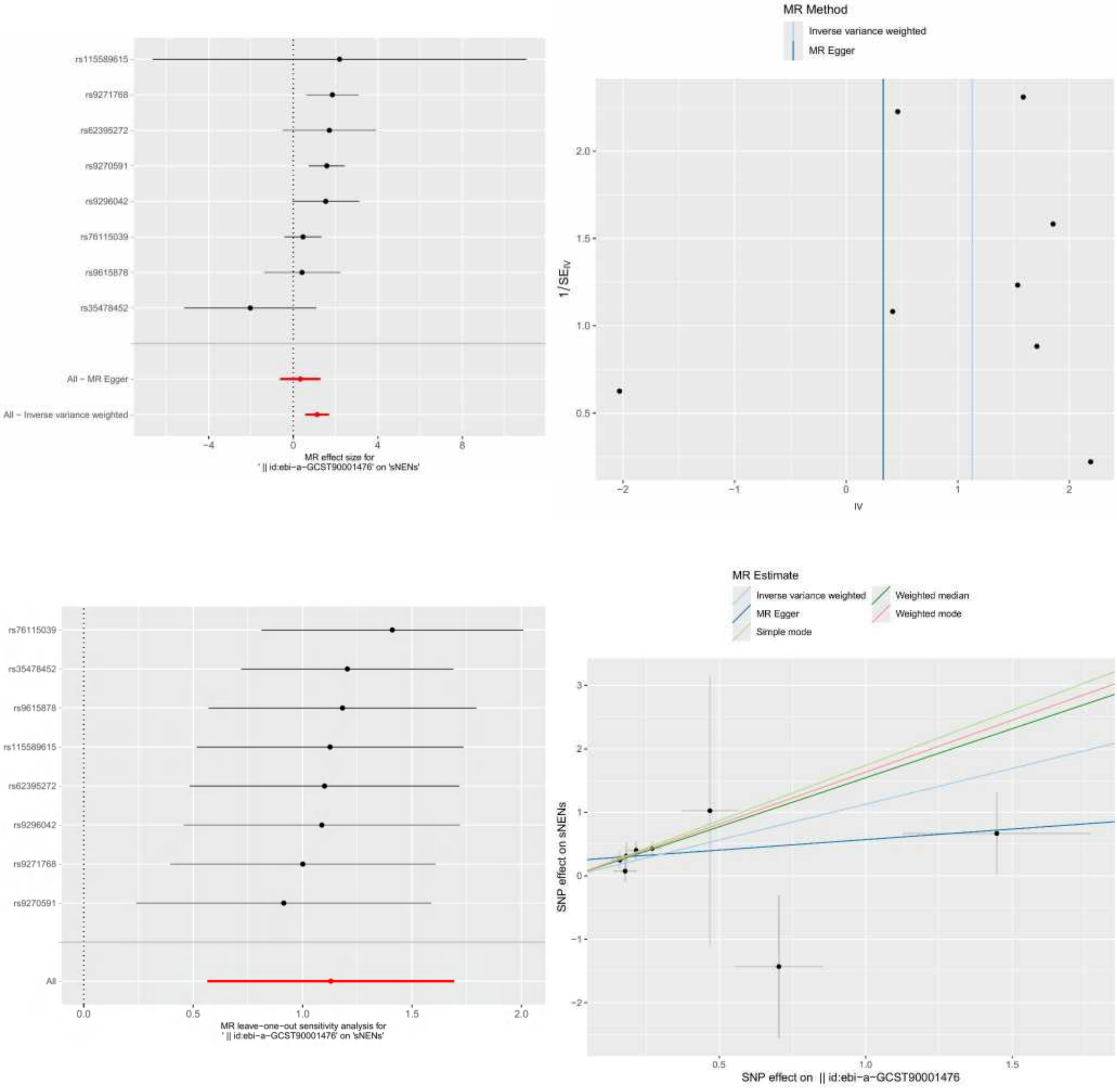
rs115589615
rs9271768
rs62395272
rs9270591
rs9296042
rs76115039
rs9615878
rs35478452
All - MR Egger
All - Inverse variance weighted
-4
0
4
8
MR effect size for
' || id:ebi-a-GCST90001476' on 'sNENs'
MR Method
Inverse variance weighted
MR Egger
1/SE$_{IV}$
2.0
1.5
1.0
0.5
-2
-1
0
1
2
IV
rs76115039
rs35478452
rs9615878
rs115589615
rs62395272
rs9296042
rs9271768
rs9270591
All
0.0
0.5
1.0
1.5
2.0
MR leave-one-out sensitivity analysis for
' || id:ebi-a-GCST90001476' on 'sNENs'
MR Estimate
Inverse variance weighted
MR Egger
Simple mode
Weighted median
Weighted mode
SNP effect on sNENs
3
2
1
0
-1
-2
0.5
1.0
1.5
SNP effect on || id:ebi-a-GCST90001476

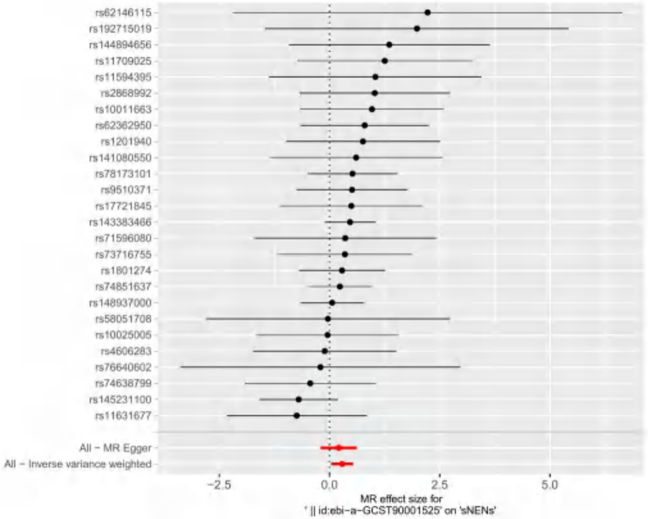
rs62146115
rs192715019
rs144894656
rs11709025
rs11594395
rs2868992
rs10011663
rs62362950
rs1201940
rs141080550
rs78173101
rs9510371
rs17721845
rs143383466
rs71596080
rs73716755
rs1801274
rs74851637
rs148937000
rs58051708
rs10025005
rs4606283
rs76640602
rs74638799
rs145231100
rs11631677
All − MR Egger
All − Inverse variance weighted
−2.5
0.0
2.5
5.0
MR effect size for
' || id:ebi−a−GCST90001525' on 'sNENs'

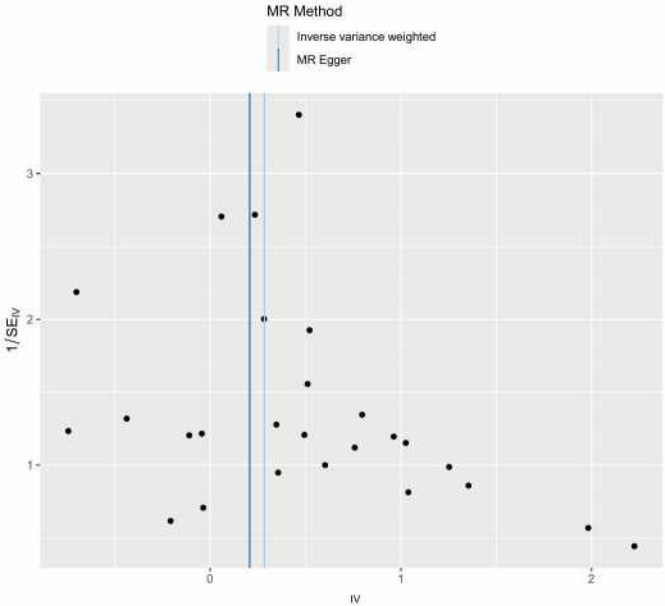
MR Method
Inverse variance weighted
MR Egger
$1/SE_{IV}$
IV

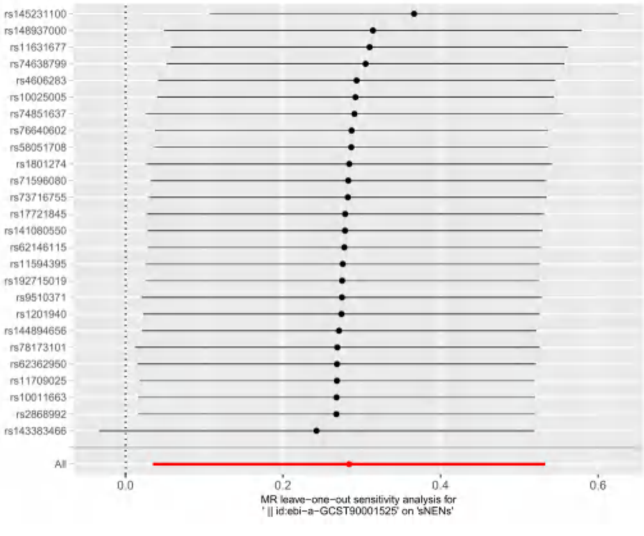
rs145231100
rs148937000
rs11631677
rs74638799
rs4606283
rs10025005
rs74851637
rs76640602
rs58051708
rs1801274
rs71596080
rs73716755
rs17721845
rs141080550
rs62146115
rs11594395
rs192715019
rs9510371
rs1201940
rs144894656
rs78173101
rs62362950
rs11709025
rs10011663
rs2868992
rs143383466
All
0.0
0.2
0.4
0.6
MR leave−one−out sensitivity analysis for
' || id:ebi−a−GCST90001525' on 'sNENs'

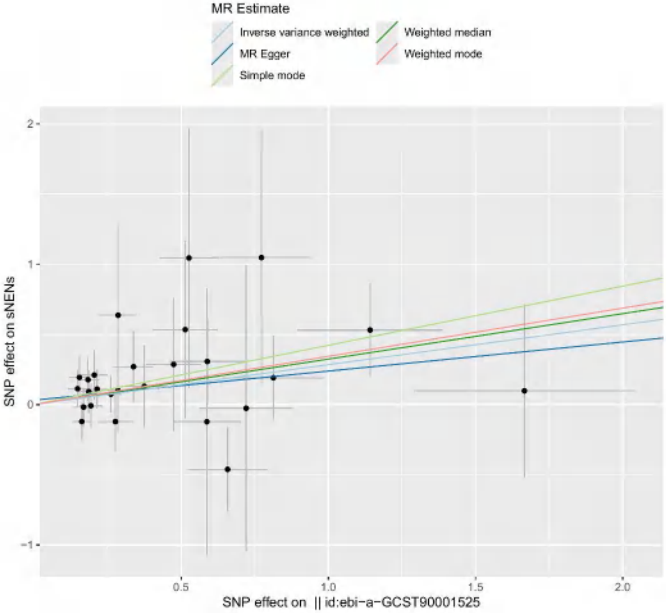
MR Estimate
Inverse variance weighted
MR Egger
Simple mode
Weighted median
Weighted mode
SNP effect on sNENs
SNP effect on || id:ebi−a−GCST90001525

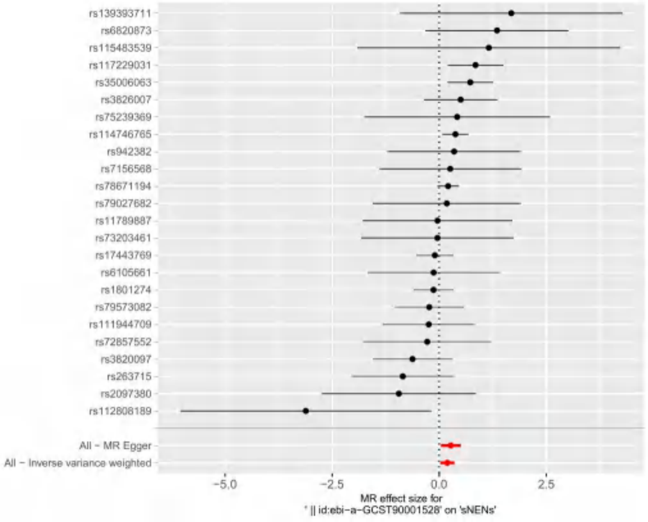
rs139393711
rs6820873
rs115483539
rs117229031
rs35006063
rs3826007
rs75239369
rs114746765
rs942382
rs7156568
rs78671194
rs79027682
rs11789887
rs73203461
rs17443769
rs6105661
rs1801274
rs79573082
rs111944709
rs72857552
rs3820097
rs263715
rs2097380
rs112808189
All − MR Egger
All − Inverse variance weighted
−5.0
−2.5
0.0
2.5
MR effect size for
' || id:ebi−a−GCST90001528' on 'sNENs'

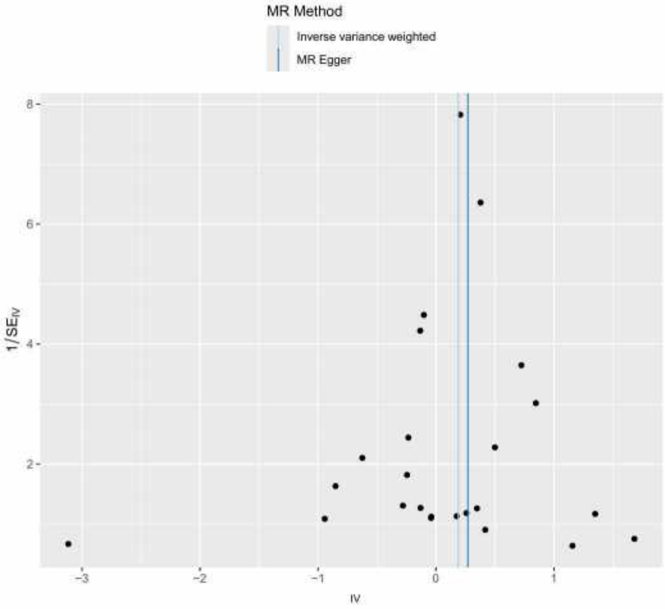
MR Method
Inverse variance weighted
MR Egger
1/SE$_{IV}$
8
6
4
2
−3
−2
−1
0
1
IV

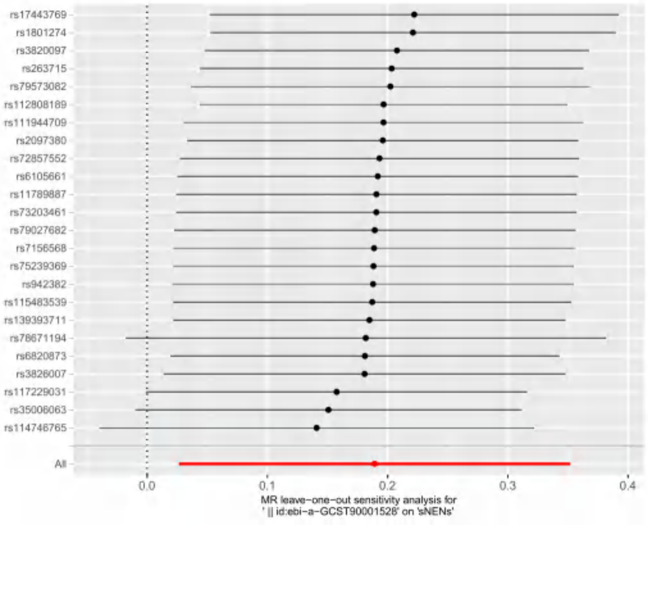
rs17443769
rs1801274
rs3820097
rs263715
rs79573082
rs112808189
rs111944709
rs2097380
rs72857552
rs6105661
rs11789887
rs73203461
rs79027682
rs7156568
rs75239369
rs942382
rs115483539
rs139393711
rs78671194
rs6820873
rs3826007
rs117229031
rs35006063
rs114746765
All
0.0
0.1
0.2
0.3
0.4
MR leave−one−out sensitivity analysis for
' || id:ebi−a−GCST90001528' on 'sNENs'

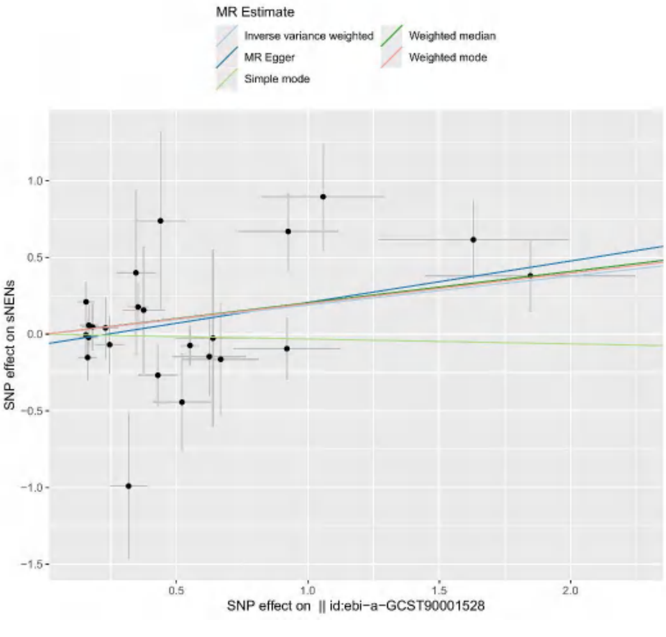
MR Estimate
Inverse variance weighted
MR Egger
Simple mode
Weighted median
Weighted mode
SNP effect on sNENs
1.0
0.5
0.0
−0.5
−1.0
−1.5
0.5
1.0
1.5
2.0
SNP effect on || id:ebi−a−GCST90001528

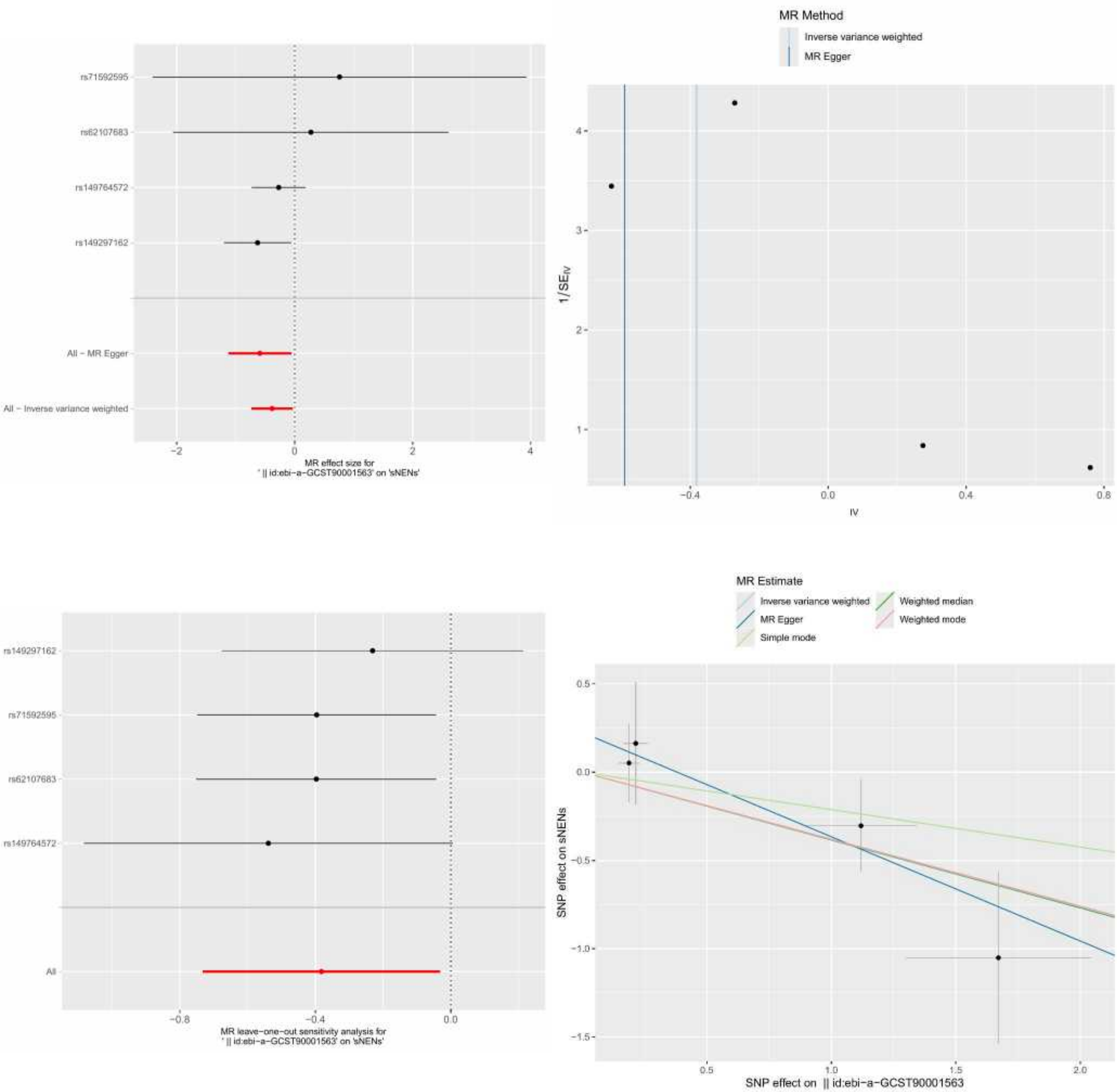

rs71592595
rs62107683
rs149764572
rs149297162
All − MR Egger
All − Inverse variance weighted
−2
0
2
4
MR effect size for
' || id:ebi−a−GCST90001563' on 'sNENs'
MR Method
Inverse variance weighted
MR Egger
1/SE$_{IV}$
IV
−0.4
0.0
0.4
0.8
rs149297162
rs71592595
rs62107683
rs149764572
All
−0.8
−0.4
0.0
MR leave−one−out sensitivity analysis for
' || id:ebi−a−GCST90001563' on 'sNENs'
MR Estimate
Inverse variance weighted
MR Egger
Simple mode
Weighted median
Weighted mode
SNP effect on sNENs
SNP effect on || id:ebi−a−GCST90001563
0.5
0.0
−0.5
−1.0
−1.5
1.0
1.5
2.0

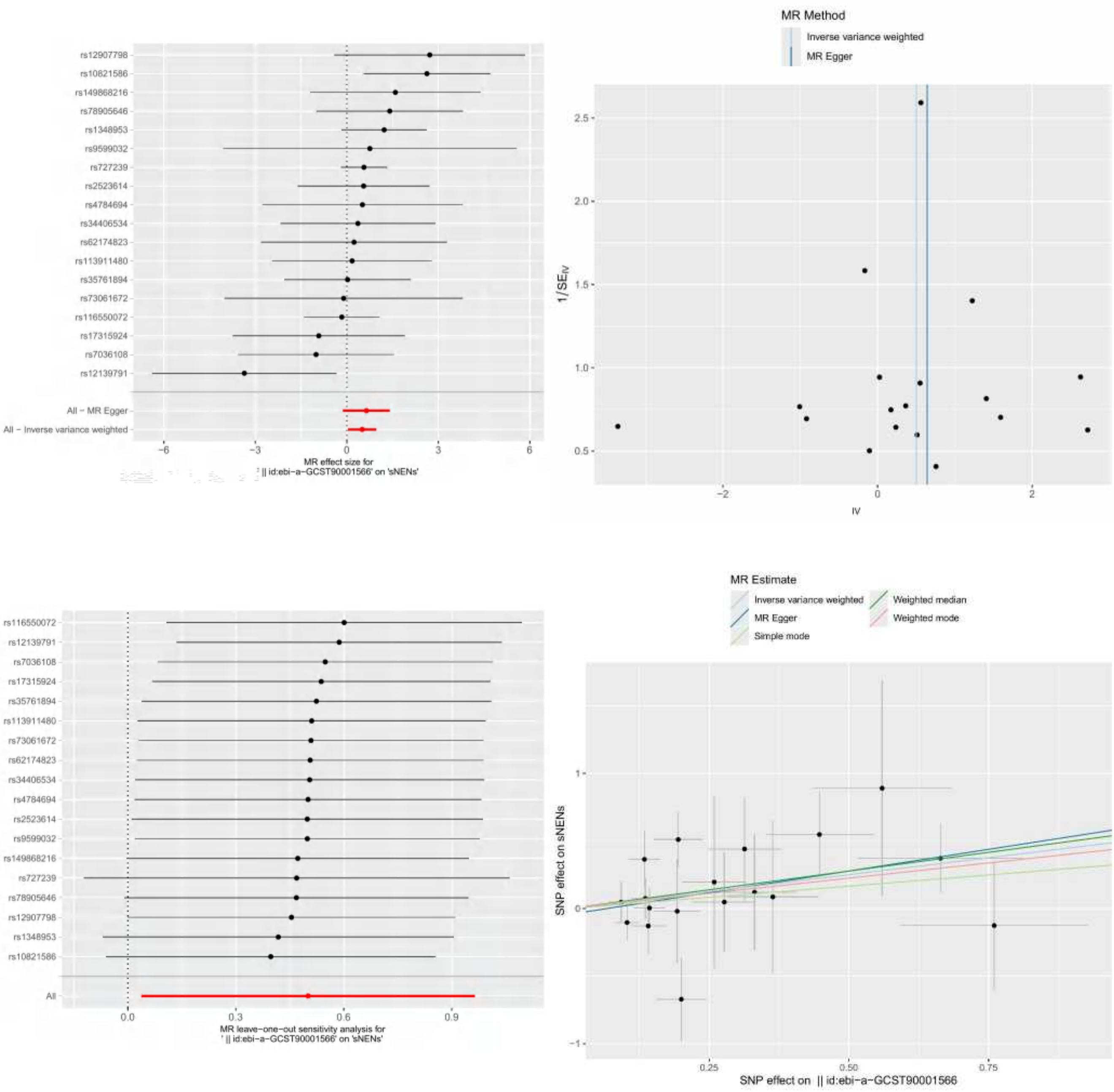

rs12907798
rs10821586
rs149868216
rs78905646
rs1348953
rs9599032
rs727239
rs2523614
rs4784694
rs34406534
rs62174823
rs113911480
rs35761894
rs73061672
rs116550072
rs17315924
rs7036108
rs12139791
All − MR Egger
All − Inverse variance weighted
−6
−3
0
3
6
MR effect size for
' || id:ebi-a-GCST90001566' on 'sNENs'
MR Method
Inverse variance weighted
MR Egger
1/SE_IV
2.5
2.0
1.5
1.0
0.5
−2
0
2
IV
rs116550072
rs12139791
rs7036108
rs17315924
rs35761894
rs113911480
rs73061672
rs62174823
rs34406534
rs4784694
rs2523614
rs9599032
rs149868216
rs727239
rs78905646
rs12907798
rs1348953
rs10821586
All
0.0
0.3
0.6
0.9
MR leave−one−out sensitivity analysis for
' || id:ebi-a-GCST90001566' on 'sNENs'
MR Estimate
Inverse variance weighted
MR Egger
Simple mode
Weighted median
Weighted mode
SNP effect on sNENs
1
0
−1
0.25
0.50
0.75
SNP effect on || id:ebi-a-GCST90001566

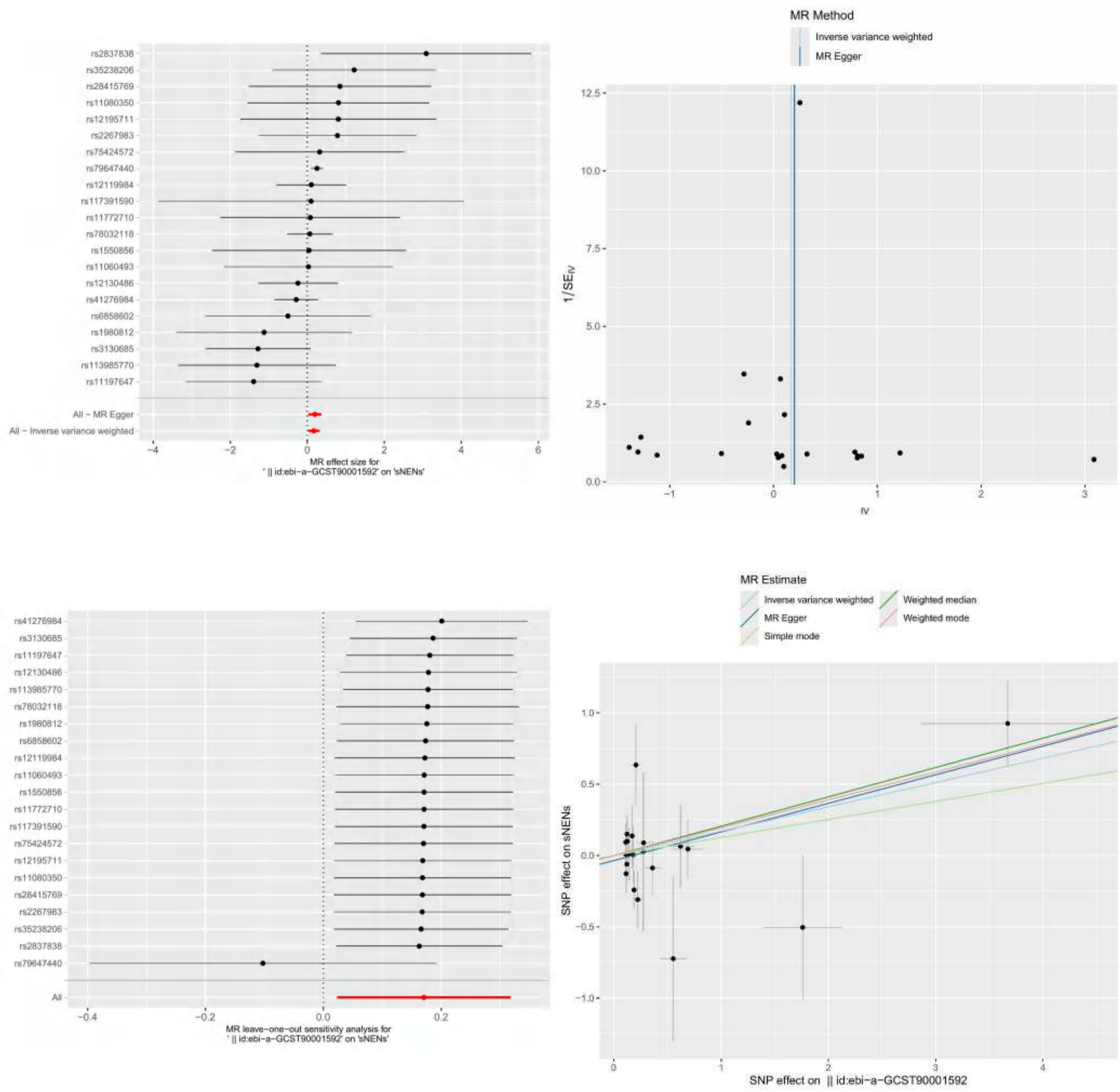

rs2837838
rs35238206
rs28415769
rs11080350
rs12195711
rs2267983
rs75424572
rs79647440
rs12119984
rs117391590
rs11772710
rs78032118
rs1550856
rs11060493
rs12130486
rs41276984
rs6858602
rs1980812
rs3130685
rs113985770
rs11197647
All − MR Egger
All − Inverse variance weighted
−4
−2
0
2
4
6
MR effect size for
' || id:ebi−a−GCST90001592' on 'sNENs'
MR Method
Inverse variance weighted
MR Egger
12.5
10.0
7.5
5.0
2.5
0.0
1/SE_IV
−1
0
1
2
3
IV
rs41276984
rs3130685
rs11197647
rs12130486
rs113985770
rs78032118
rs1980812
rs6858602
rs12119984
rs11060493
rs1550856
rs11772710
rs117391590
rs75424572
rs12195711
rs11080350
rs28415769
rs2267983
rs35238206
rs2837838
rs79647440
All
−0.4
−0.2
0.0
0.2
MR leave−one−out sensitivity analysis for
' || id:ebi−a−GCST90001592' on 'sNENs'
MR Estimate
Inverse variance weighted
MR Egger
Simple mode
Weighted median
Weighted mode
1.0
0.5
0.0
−0.5
−1.0
SNP effect on sNENs
0
1
2
3
4
SNP effect on || id:ebi−a−GCST90001592

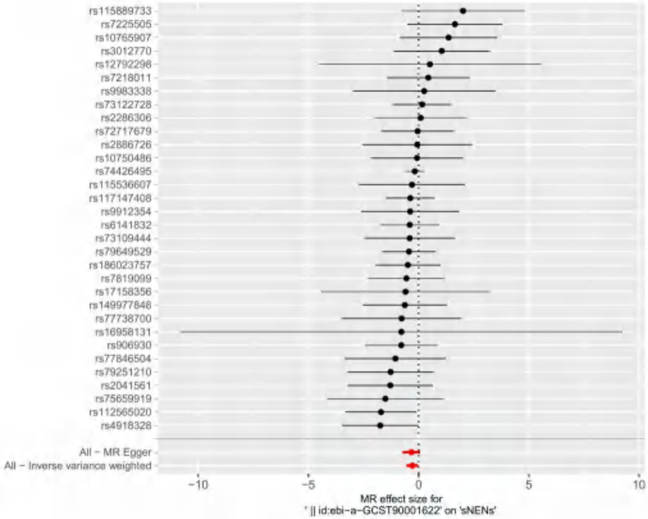

rs115889733
rs7225505
rs10765907
rs3012770
rs12792298
rs7218011
rs9983338
rs73122728
rs2286306
rs72717679
rs2886726
rs10750486
rs74426495
rs115536607
rs117147408
rs9912354
rs6141832
rs73109444
rs79649529
rs186023757
rs7819099
rs17158356
rs149977848
rs77738700
rs16958131
rs906930
rs77846504
rs79251210
rs2041561
rs75659919
rs112565020
rs4918328
All - MR Egger
All - Inverse variance weighted
-10
-5
0
5
10
MR effect size for
' || id:ebi-a-GCST90001622' on 'sNENs'

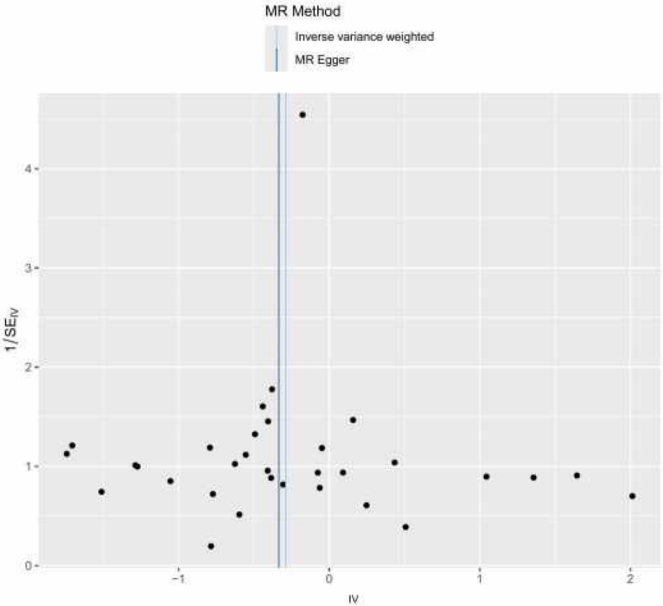

MR Method
Inverse variance weighted
MR Egger
1/SE$_{IV}$
IV

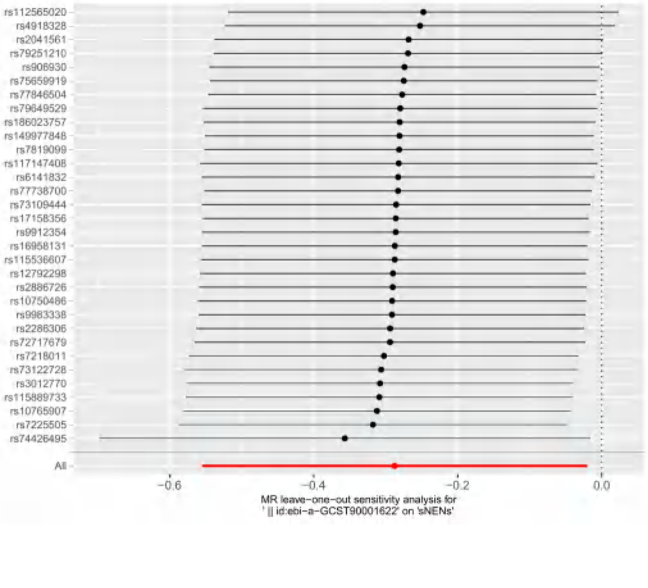

rs112565020
rs4918328
rs2041561
rs79251210
rs906930
rs75659919
rs77846504
rs79649529
rs186023757
rs149977848
rs7819099
rs117147408
rs6141832
rs77738700
rs73109444
rs17158356
rs9912354
rs16958131
rs115536607
rs12792298
rs2886726
rs10750486
rs9983338
rs2286306
rs72717679
rs7218011
rs73122728
rs3012770
rs115889733
rs10765907
rs7225505
rs74426495
All
-0.6
-0.4
-0.2
0.0
MR leave-one-out sensitivity analysis for
' || id:ebi-a-GCST90001622' on 'sNENs'

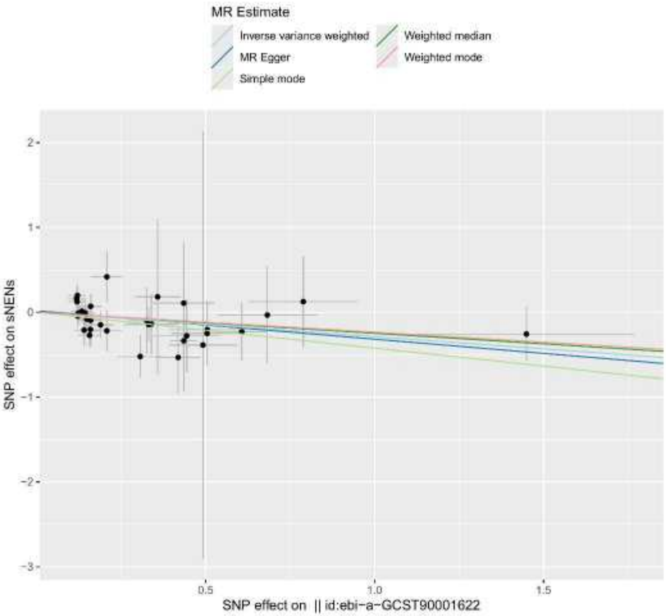

MR Estimate
Inverse variance weighted
MR Egger
Simple mode
Weighted median
Weighted mode
SNP effect on sNENs
SNP effect on || id:ebi-a-GCST90001622

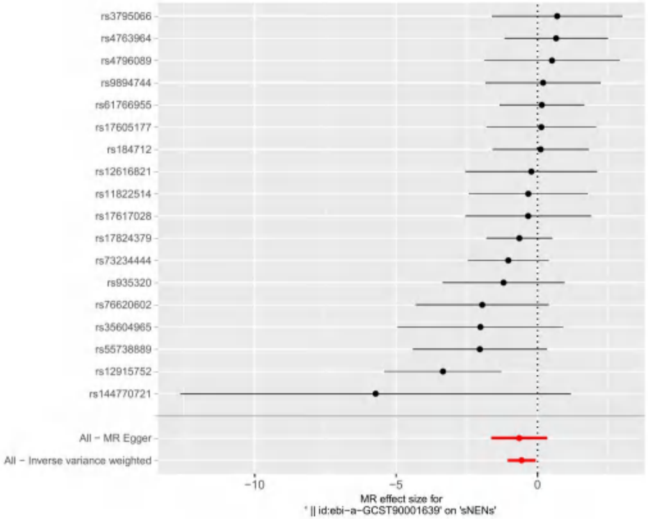
rs3795066
rs4763964
rs4796089
rs9894744
rs61766955
rs17605177
rs184712
rs12616821
rs11822514
rs17617028
rs17824379
rs73234444
rs935320
rs76620602
rs35604965
rs55738889
rs12915752
rs144770721
All − MR Egger
All − Inverse variance weighted
−10
−5
0
MR effect size for
' || id:ebi−a−GCST90001639' on 'sNENs'

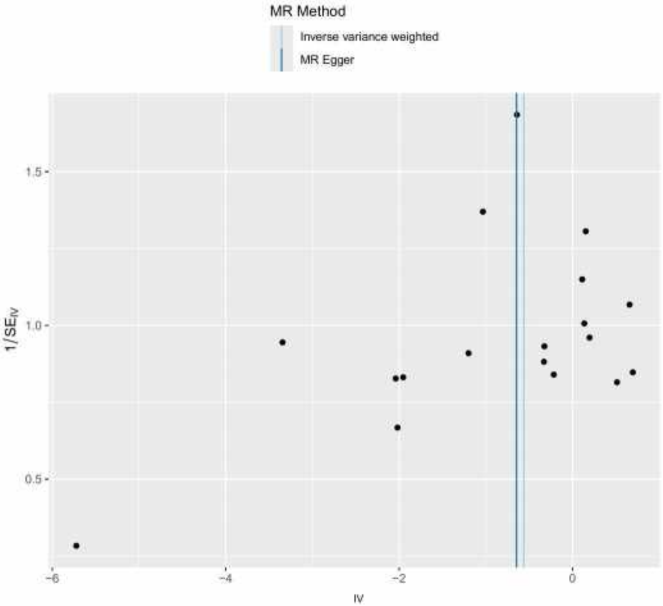
MR Method
Inverse variance weighted
MR Egger
1/SE$_{IV}$
1.5
1.0
0.5
−6
−4
−2
0
IV

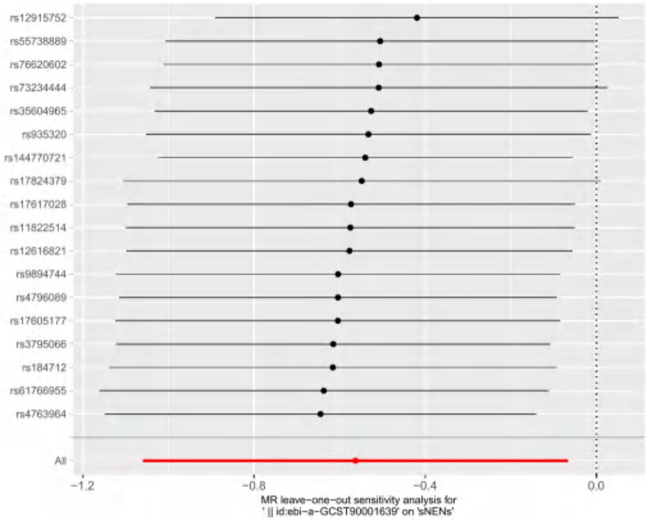
rs12915752
rs55738889
rs76620602
rs73234444
rs35604965
rs935320
rs144770721
rs17824379
rs17617028
rs11822514
rs12616821
rs9894744
rs4796089
rs17605177
rs3795066
rs184712
rs61766955
rs4763964
All
−1.2
−0.8
−0.4
0.0
MR leave−one−out sensitivity analysis for
' || id:ebi−a−GCST90001639' on 'sNENs'

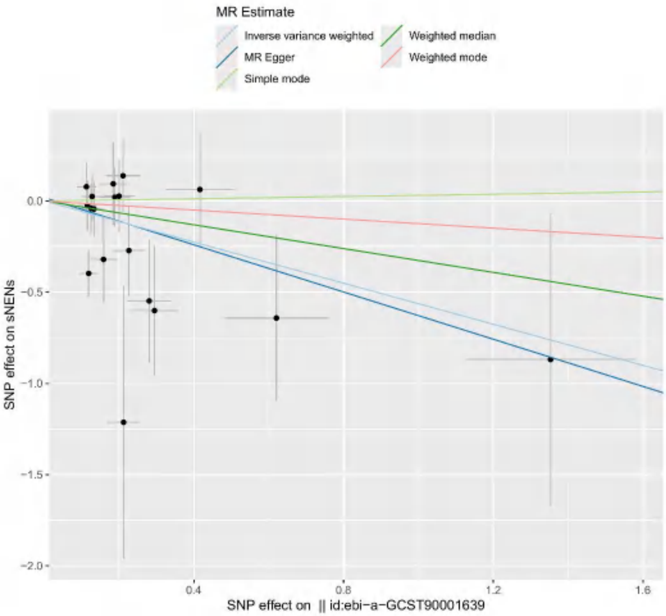
MR Estimate
Inverse variance weighted
MR Egger
Simple mode
Weighted median
Weighted mode
SNP effect on sNENs
0.0
−0.5
−1.0
−1.5
−2.0
0.4
0.8
1.2
1.6
SNP effect on || id:ebi−a−GCST90001639

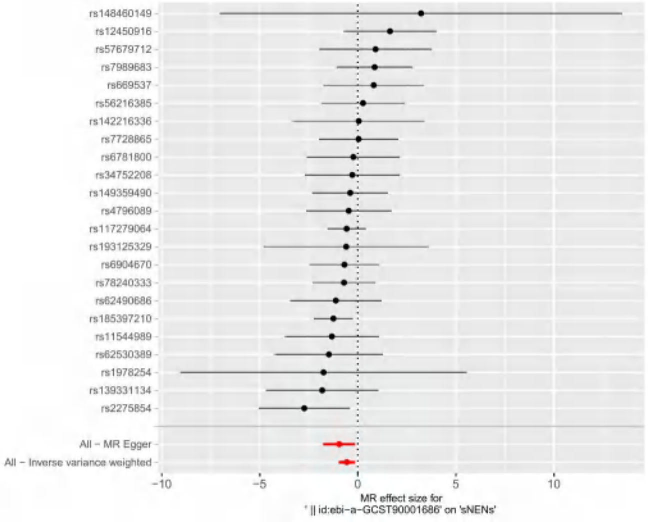
rs148460149
rs12450916
rs57679712
rs7989683
rs669537
rs56216385
rs142216336
rs7728865
rs6781800
rs34752208
rs149359490
rs4796089
rs117279064
rs193125329
rs6904670
rs78240333
rs62490686
rs185397210
rs11544989
rs62530389
rs1978254
rs139331134
rs2275854
All − MR Egger
All − Inverse variance weighted
-10
-5
0
5
10
MR effect size for
' || id:ebi-a-GCST90001686' on 'sNENs'

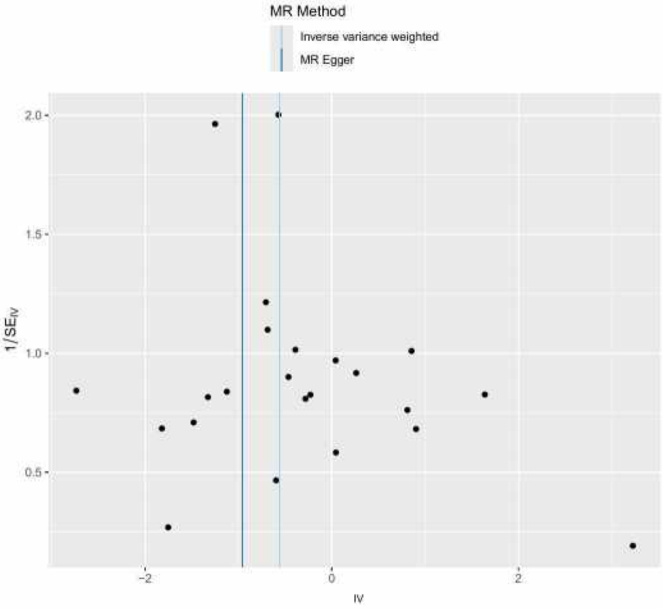
MR Method
Inverse variance weighted
MR Egger
2.0
1.5
1.0
0.5
1/SE$_{IV}$
-2
0
2
IV

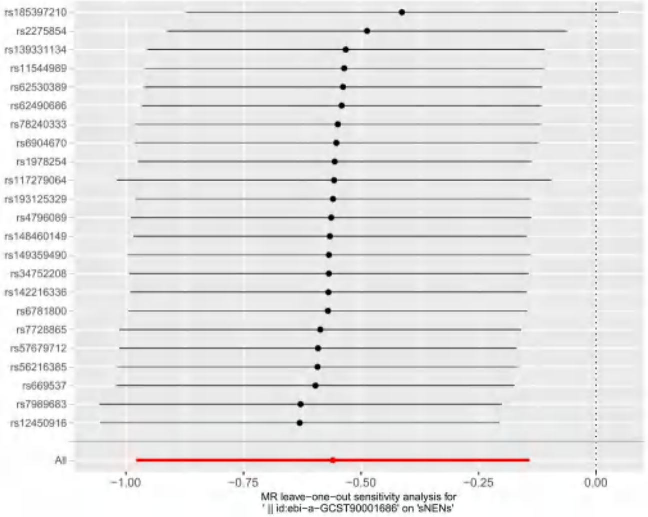
rs185397210
rs2275854
rs139331134
rs11544989
rs62530389
rs62490686
rs78240333
rs6904670
rs1978254
rs117279064
rs193125329
rs4796089
rs148460149
rs149359490
rs34752208
rs142216336
rs6781800
rs7728865
rs57679712
rs56216385
rs669537
rs7989683
rs12450916
All
-1.00
-0.75
-0.50
-0.25
0.00
MR leave-one-out sensitivity analysis for
' || id:ebi-a-GCST90001686' on 'sNENs'

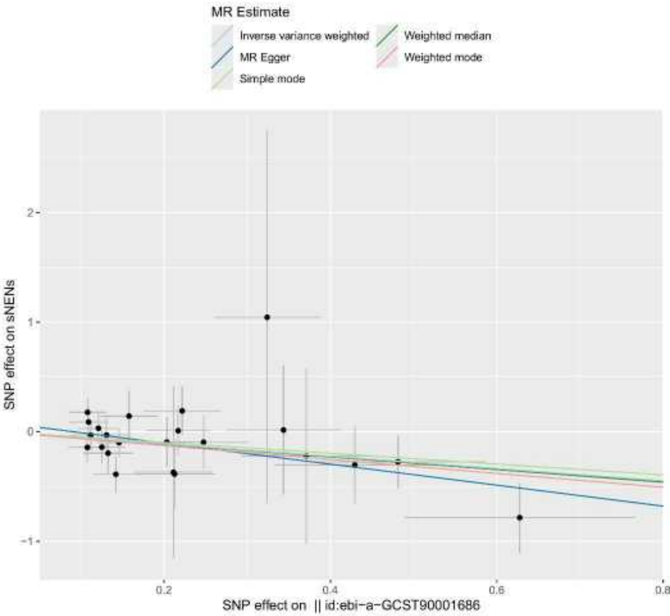
MR Estimate
Inverse variance weighted
MR Egger
Simple mode
Weighted median
Weighted mode
2
1
0
-1
SNP effect on sNENs
0.2
0.4
0.6
0.8
SNP effect on || id:ebi-a-GCST90001686

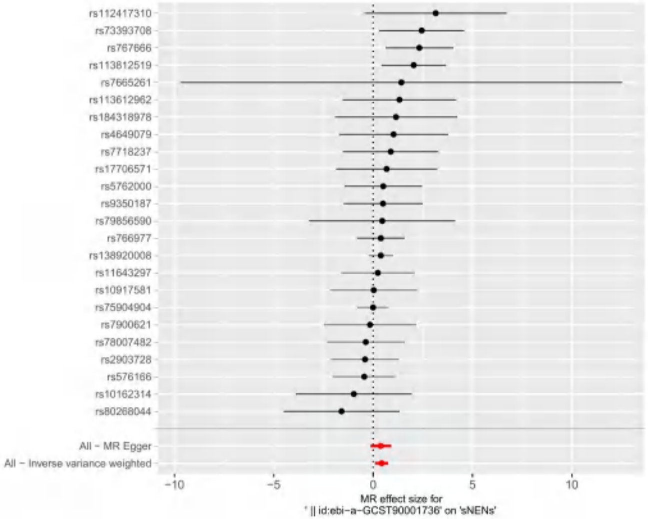

rs112417310
rs73393708
rs767666
rs113812519
rs7665261
rs113612962
rs184318978
rs4649079
rs7718237
rs17706571
rs5762000
rs9350187
rs79856590
rs766977
rs138920008
rs11643297
rs10917581
rs75904904
rs7900621
rs78007482
rs2903728
rs576166
rs10162314
rs80268044
All − MR Egger
All − Inverse variance weighted
−10
−5
0
5
10
MR effect size for
' || id:ebi−a−GCST90001736' on 'sNENs'

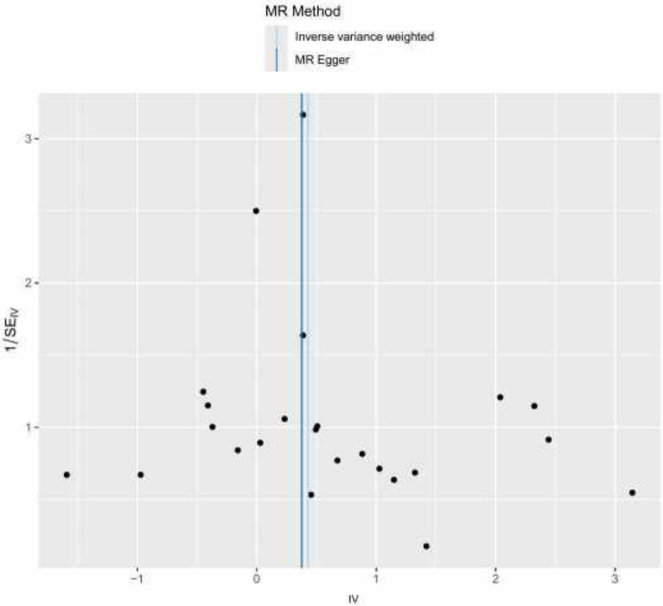

MR Method
Inverse variance weighted
MR Egger
1/SE$_{IV}$
3
2
1
IV
−1
0
1
2
3

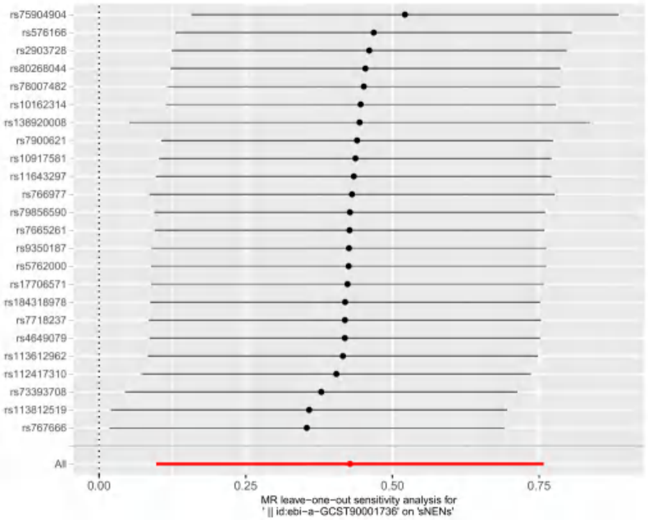

rs75904904
rs576166
rs2903728
rs80268044
rs78007482
rs10162314
rs138920008
rs7900621
rs10917581
rs11643297
rs766977
rs79856590
rs7665261
rs9350187
rs5762000
rs17706571
rs184318978
rs7718237
rs4649079
rs113612962
rs112417310
rs73393708
rs113812519
rs767666
All
0.00
0.25
0.50
0.75
MR leave−one−out sensitivity analysis for
' || id:ebi−a−GCST90001736' on 'sNENs'

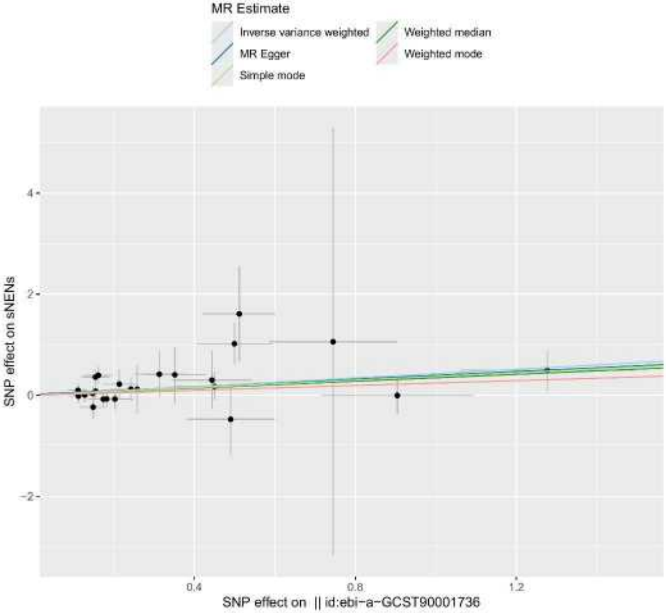

MR Estimate
Inverse variance weighted
MR Egger
Simple mode
Weighted median
Weighted mode
SNP effect on sNENs
4
2
0
−2
0.4
0.8
1.2
SNP effect on || id:ebi−a−GCST90001736

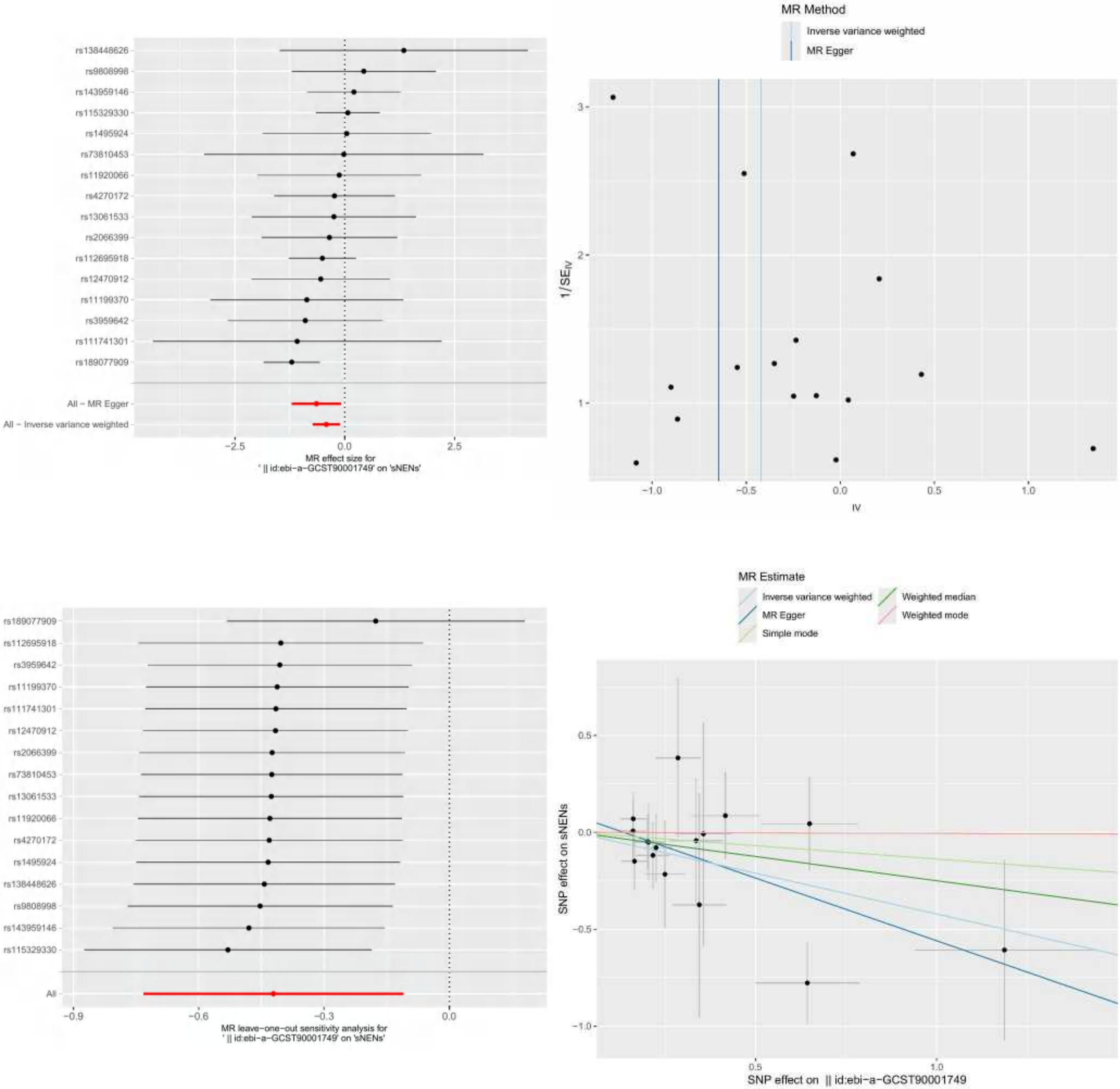

rs138448626
rs9808998
rs143959146
rs115329330
rs1495924
rs73810453
rs11920066
rs4270172
rs13061533
rs2066399
rs112695918
rs12470912
rs11199370
rs3959642
rs111741301
rs189077909
All - MR Egger
All - Inverse variance weighted
-2.5
0.0
2.5
MR effect size for
' || id:ebi-a-GCST90001749' on 'sNENs'
MR Method
Inverse variance weighted
MR Egger
1/SE_IV
3
2
1
-1.0
-0.5
0.0
0.5
1.0
IV
rs189077909
rs112695918
rs3959642
rs11199370
rs111741301
rs12470912
rs2066399
rs73810453
rs13061533
rs11920066
rs4270172
rs1495924
rs138448626
rs9808998
rs143959146
rs115329330
All
-0.9
-0.6
-0.3
0.0
MR leave-one-out sensitivity analysis for
' || id:ebi-a-GCST90001749' on 'sNENs'
MR Estimate
Inverse variance weighted
MR Egger
Simple mode
Weighted median
Weighted mode
SNP effect on sNENs
0.5
0.0
-0.5
-1.0
0.5
1.0
SNP effect on || id:ebi-a-GCST90001749

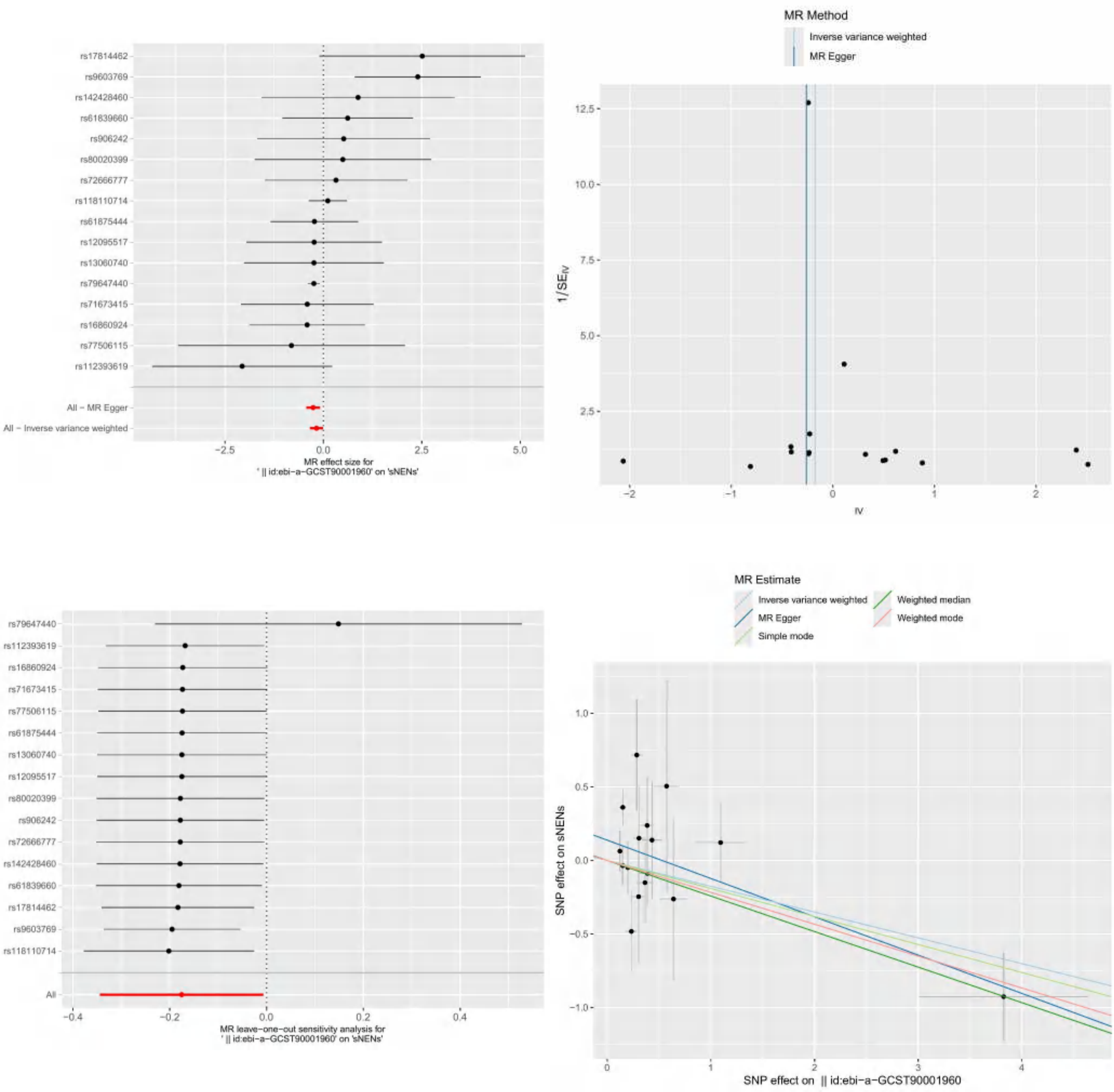

rs17814462
rs9603769
rs142428460
rs61839660
rs906242
rs80020399
rs72666777
rs118110714
rs61875444
rs12095517
rs13060740
rs79647440
rs71673415
rs16860924
rs77506115
rs112393619
All – MR Egger
All – Inverse variance weighted
−2.5
0.0
2.5
5.0
MR effect size for
' || id:ebi−a−GCST90001960' on 'sNENs'
MR Method
Inverse variance weighted
MR Egger
12.5
10.0
7.5
5.0
2.5
$1/SE_{IV}$
−2
−1
0
1
2
IV
rs79647440
rs112393619
rs16860924
rs71673415
rs77506115
rs61875444
rs13060740
rs12095517
rs80020399
rs906242
rs72666777
rs142428460
rs61839660
rs17814462
rs9603769
rs118110714
All
−0.4
−0.2
0.0
0.2
0.4
MR leave−one−out sensitivity analysis for
' || id:ebi−a−GCST90001960' on 'sNENs'
MR Estimate
Inverse variance weighted
MR Egger
Simple mode
Weighted median
Weighted mode
1.0
0.5
0.0
−0.5
−1.0
SNP effect on sNENs
0
1
2
3
4
SNP effect on || id:ebi−a−GCST90001960

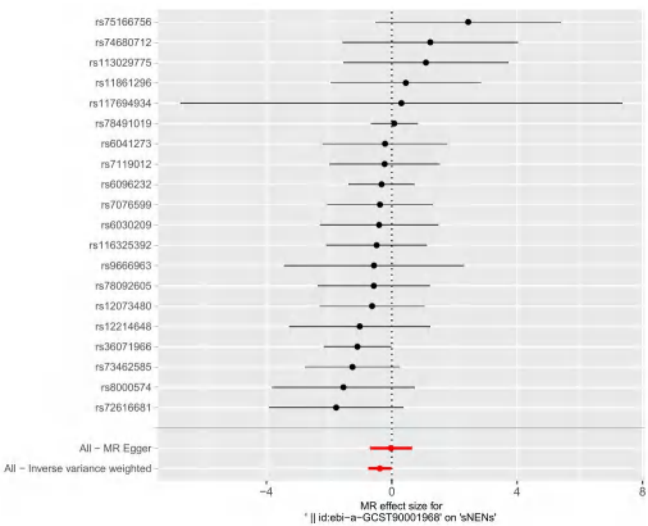
rs75166756
rs74680712
rs113029775
rs11861296
rs117694934
rs78491019
rs6041273
rs7119012
rs6096232
rs7076599
rs6030209
rs116325392
rs9666963
rs78092605
rs12073480
rs12214648
rs36071966
rs73462585
rs8000574
rs72616681
All − MR Egger
All − Inverse variance weighted
−4
0
4
8
MR effect size for
' || id:ebi−a−GCST90001968' on 'sNENs'

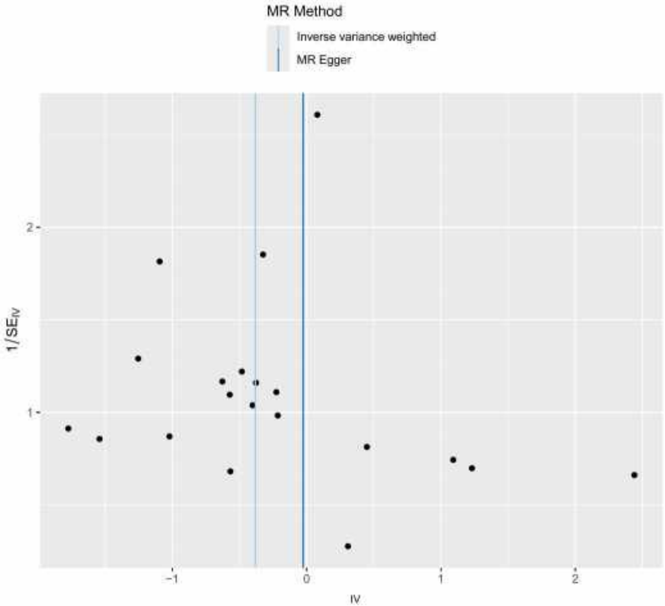
MR Method
Inverse variance weighted
MR Egger
1/SE_IV
2
1
−1
0
1
2
IV

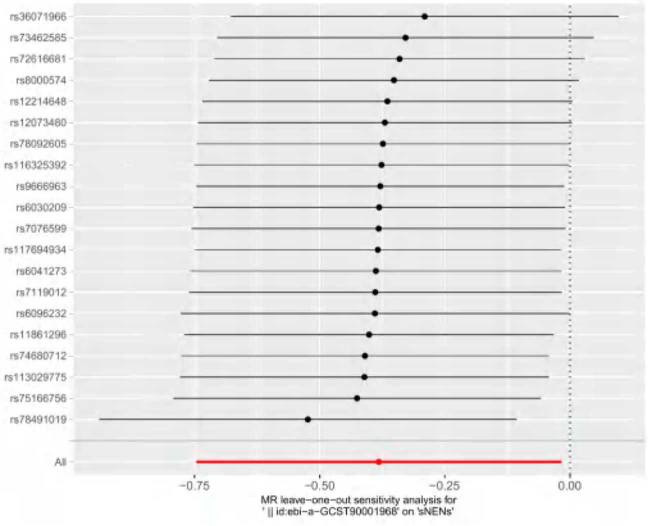
rs36071966
rs73462585
rs72616681
rs8000574
rs12214648
rs12073480
rs78092605
rs116325392
rs9666963
rs6030209
rs7076599
rs117694934
rs6041273
rs7119012
rs6096232
rs11861296
rs74680712
rs113029775
rs75166756
rs78491019
All
−0.75
−0.50
−0.25
0.00
MR leave−one−out sensitivity analysis for
' || id:ebi−a−GCST90001968' on 'sNENs'

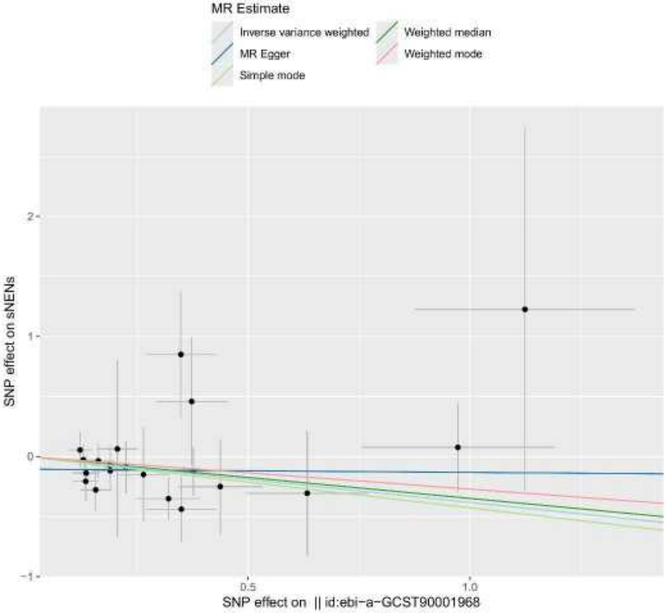
MR Estimate
Inverse variance weighted
MR Egger
Simple mode
Weighted median
Weighted mode
SNP effect on sNENs
2
1
0
−1
0.5
1.0
SNP effect on || id:ebi−a−GCST90001968

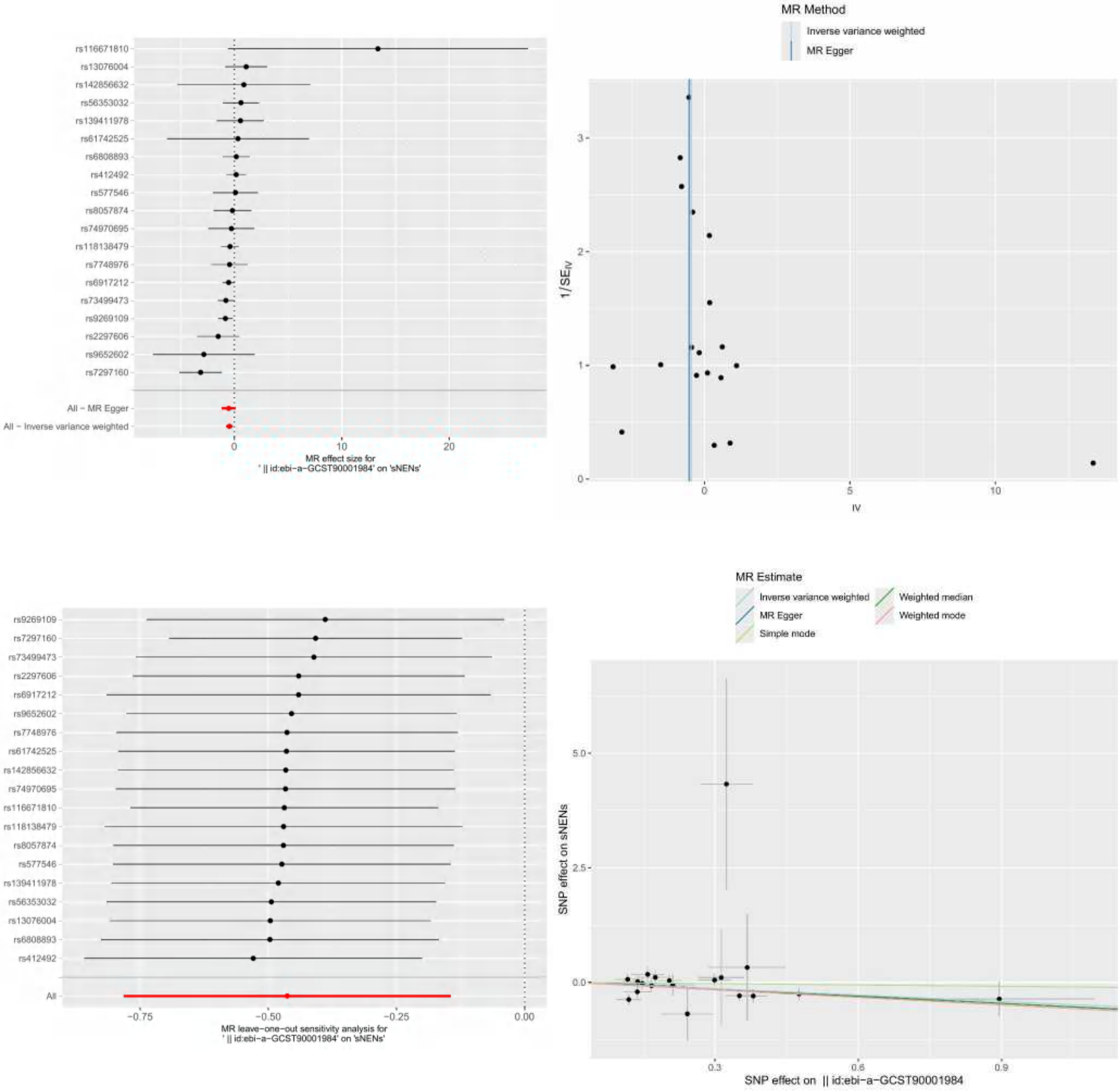

rs116671810
rs13076004
rs142856632
rs56353032
rs139411978
rs61742525
rs6808893
rs412492
rs577546
rs8057874
rs74970695
rs118138479
rs7748976
rs6917212
rs73499473
rs9269109
rs2297606
rs9652602
rs7297160
All − MR Egger
All − Inverse variance weighted
0
10
20
MR effect size for
' || id:ebi−a−GCST90001984' on 'sNENs'
MR Method
Inverse variance weighted
MR Egger
1/SE_IV
0
1
2
3
0
5
10
IV
rs9269109
rs7297160
rs73499473
rs2297606
rs6917212
rs9652602
rs7748976
rs61742525
rs142856632
rs74970695
rs116671810
rs118138479
rs8057874
rs577546
rs139411978
rs56353032
rs13076004
rs6808893
rs412492
All
−0.75
−0.50
−0.25
0.00
MR leave−one−out sensitivity analysis for
' || id:ebi−a−GCST90001984' on 'sNENs'
MR Estimate
Inverse variance weighted
MR Egger
Simple mode
Weighted median
Weighted mode
SNP effect on sNENs
0.0
2.5
5.0
0.3
0.6
0.9
SNP effect on || id:ebi−a−GCST90001984

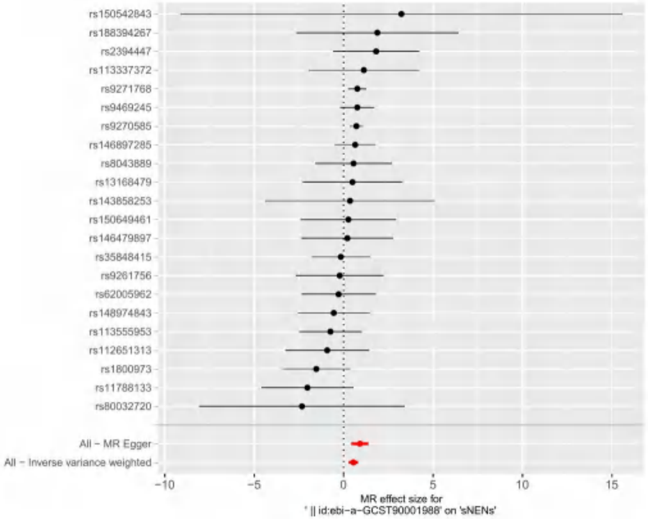
rs150542843
rs188394267
rs2394447
rs113337372
rs9271768
rs9469245
rs9270585
rs146897285
rs8043889
rs13168479
rs143858253
rs150649461
rs146479897
rs35848415
rs9261756
rs62005962
rs148974843
rs113555953
rs112651313
rs1800973
rs11788133
rs80032720
All − MR Egger
All − Inverse variance weighted
-10
-5
0
5
10
15
MR effect size for
' || id:ebi−a−GCST90001988' on 'sNENs'

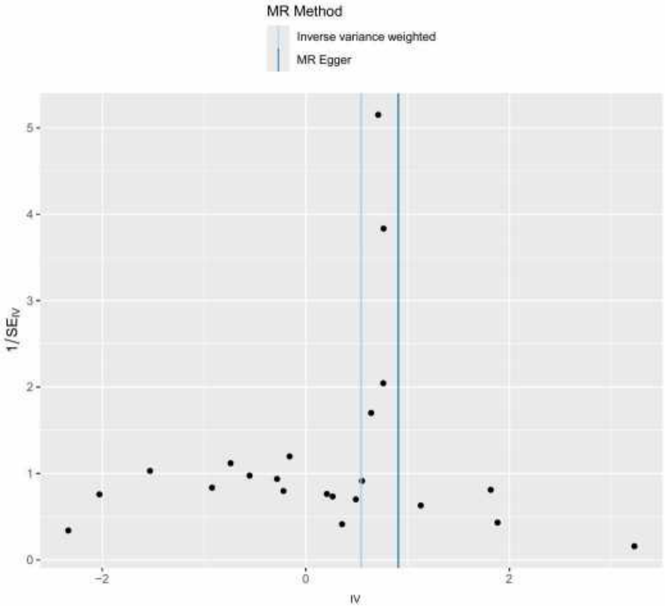
MR Method
Inverse variance weighted
MR Egger
1/SE_IV
0
1
2
3
4
5
−2
0
2
IV

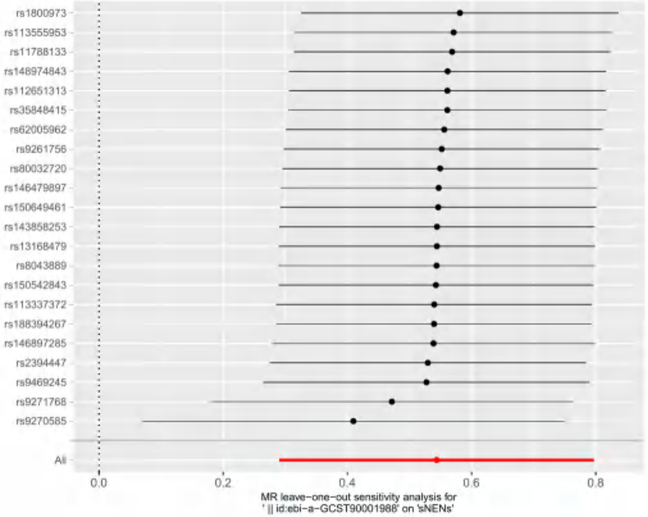
rs1800973
rs113555953
rs11788133
rs148974843
rs112651313
rs35848415
rs62005962
rs9261756
rs80032720
rs146479897
rs150649461
rs143858253
rs13168479
rs8043889
rs150542843
rs113337372
rs188394267
rs146897285
rs2394447
rs9469245
rs9271768
rs9270585
All
0.0
0.2
0.4
0.6
0.8
MR leave−one−out sensitivity analysis for
' || id:ebi−a−GCST90001988' on 'sNENs'

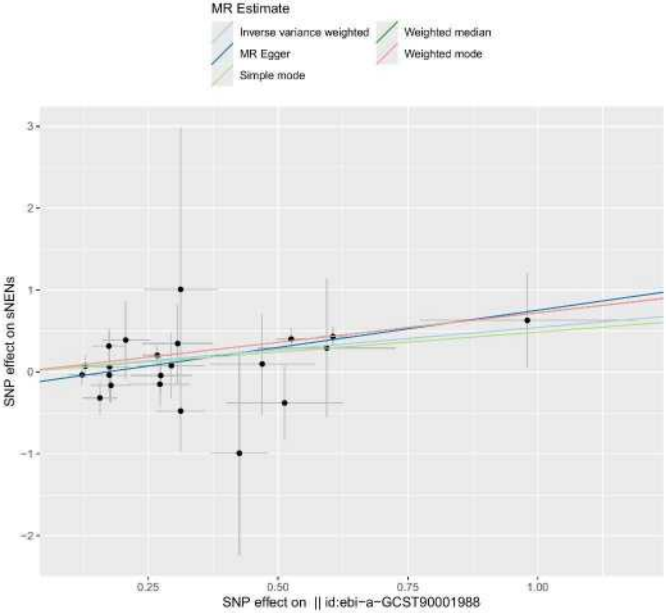
MR Estimate
Inverse variance weighted
MR Egger
Simple mode
Weighted median
Weighted mode
SNP effect on sNENs
3
2
1
0
−1
−2
0.25
0.50
0.75
1.00
SNP effect on || id:ebi−a−GCST90001988

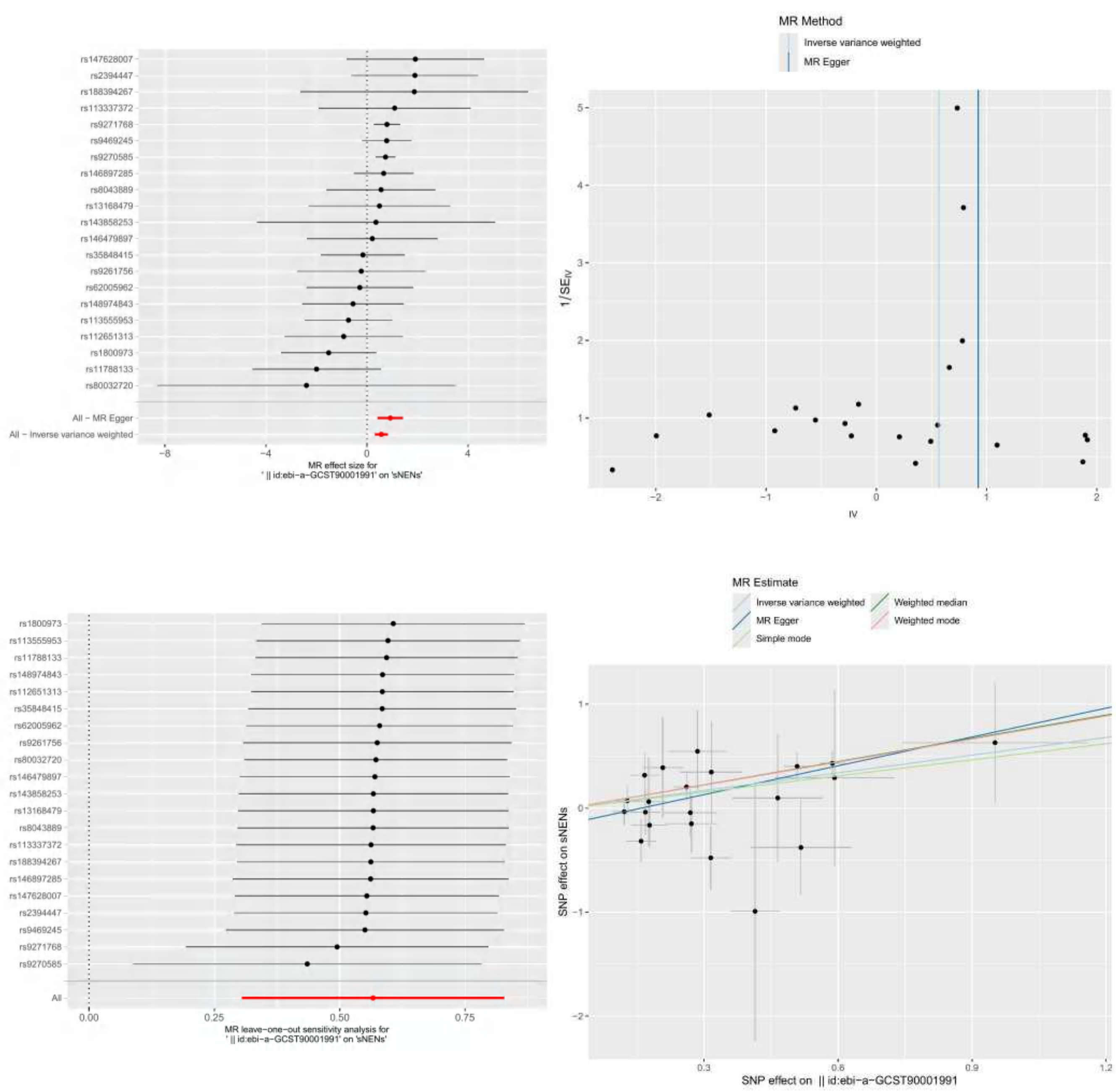

rs147628007
rs2394447
rs188394267
rs113337372
rs9271768
rs9469245
rs9270585
rs146897285
rs8043889
rs13168479
rs143858253
rs146479897
rs35848415
rs9261756
rs62005962
rs148974843
rs113555953
rs112651313
rs1800973
rs11788133
rs80032720
All − MR Egger
All − Inverse variance weighted
−8
−4
0
4
MR effect size for
' || id:ebi−a−GCST90001991' on 'sNENs'
MR Method
Inverse variance weighted
MR Egger
1/SE$_{IV}$
IV
rs1800973
rs113555953
rs11788133
rs148974843
rs112651313
rs35848415
rs62005962
rs9261756
rs80032720
rs146479897
rs143858253
rs13168479
rs8043889
rs113337372
rs188394267
rs146897285
rs147628007
rs2394447
rs9469245
rs9271768
rs9270585
All
0.00
0.25
0.50
0.75
MR leave−one−out sensitivity analysis for
' || id:ebi−a−GCST90001991' on 'sNENs'
MR Estimate
Inverse variance weighted
MR Egger
Simple mode
Weighted median
Weighted mode
SNP effect on sNENs
SNP effect on || id:ebi−a−GCST90001991

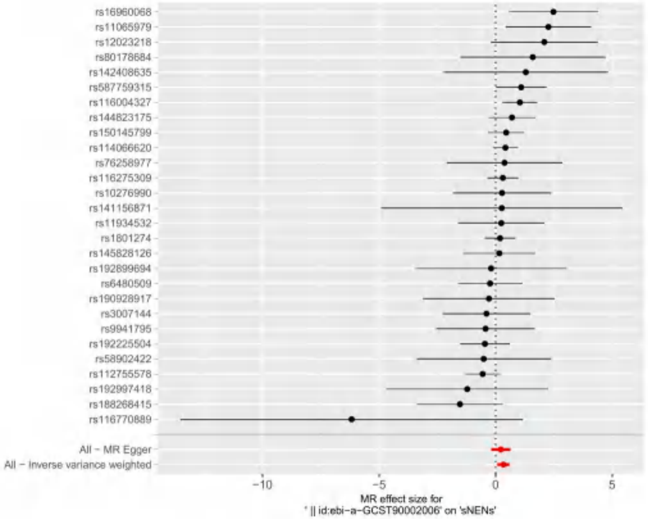
rs16960068
rs11065979
rs12023218
rs80178684
rs142408635
rs587759315
rs116004327
rs144823175
rs150145799
rs114066620
rs76258977
rs116275309
rs10276990
rs141156871
rs11934532
rs1801274
rs145828126
rs192899694
rs6480509
rs190928917
rs3007144
rs9941795
rs192225504
rs58902422
rs112755578
rs192997418
rs188268415
rs116770889
All − MR Egger
All − Inverse variance weighted
−10
−5
0
5
MR effect size for
' || id:ebi−a−GCST90002006' on 'sNENs'

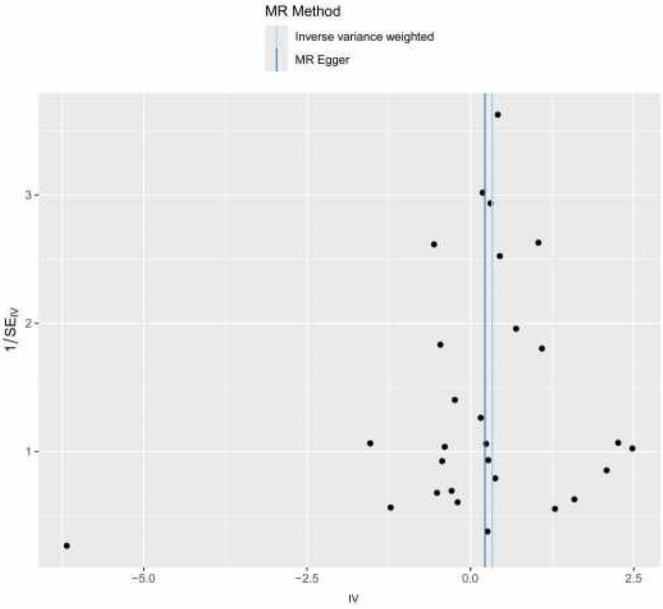
MR Method
Inverse variance weighted
MR Egger
1/SE$_{IV}$
3
2
1
−5.0
−2.5
0.0
2.5
IV

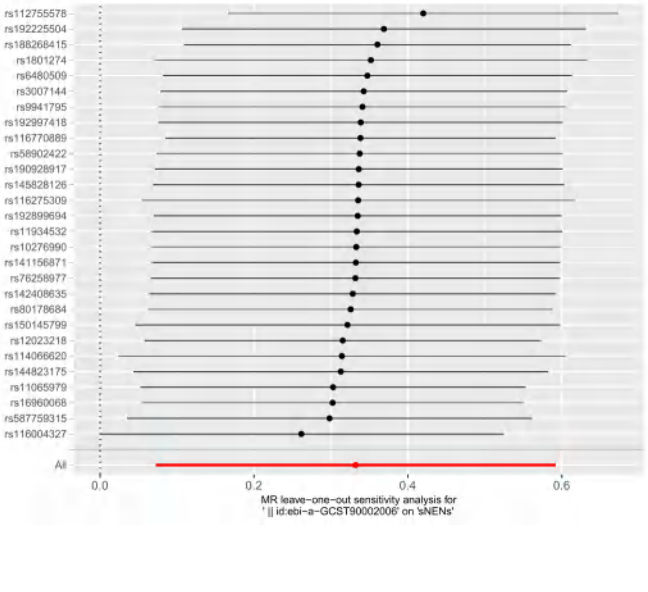
rs112755578
rs192225504
rs188268415
rs1801274
rs6480509
rs3007144
rs9941795
rs192997418
rs116770889
rs58902422
rs190928917
rs145828126
rs116275309
rs192899694
rs11934532
rs10276990
rs141156871
rs76258977
rs142408635
rs80178684
rs150145799
rs12023218
rs114066620
rs144823175
rs11065979
rs16960068
rs587759315
rs116004327
All
0.0
0.2
0.4
0.6
MR leave−one−out sensitivity analysis for
' || id:ebi−a−GCST90002006' on 'sNENs'

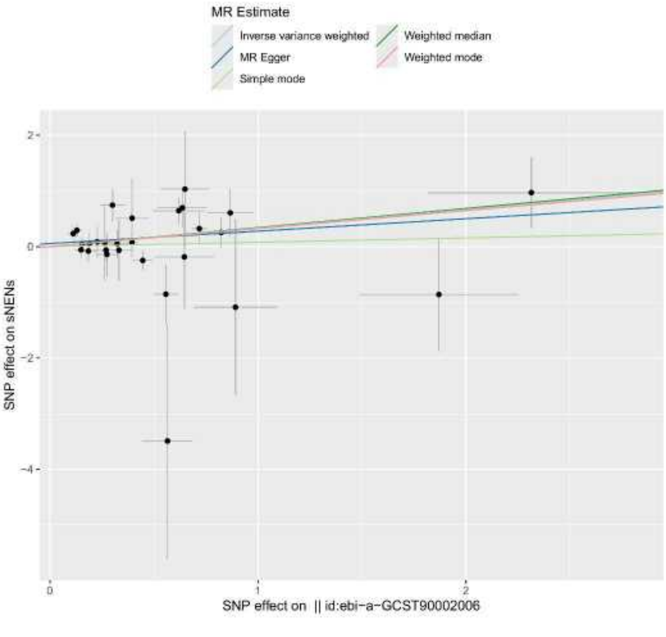
MR Estimate
Inverse variance weighted
MR Egger
Simple mode
Weighted median
Weighted mode
SNP effect on sNENs
2
0
−2
−4
0
1
2
SNP effect on || id:ebi−a−GCST90002006

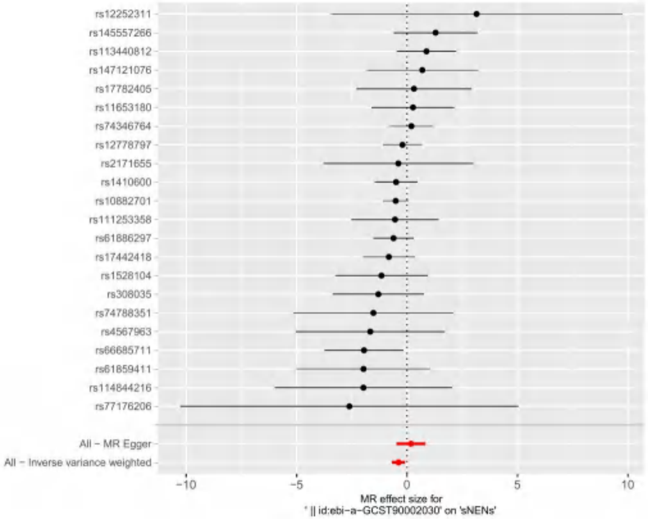
rs12252311
rs145557266
rs113440812
rs147121076
rs17782405
rs11653180
rs74346764
rs12778797
rs2171655
rs1410600
rs10882701
rs111253358
rs61886297
rs17442418
rs1528104
rs308035
rs74788351
rs4567963
rs66685711
rs61859411
rs114844216
rs77176206
All − MR Egger
All − Inverse variance weighted
−10
−5
0
5
10
MR effect size for
' || id:ebi−a−GCST90002030' on 'sNENs'

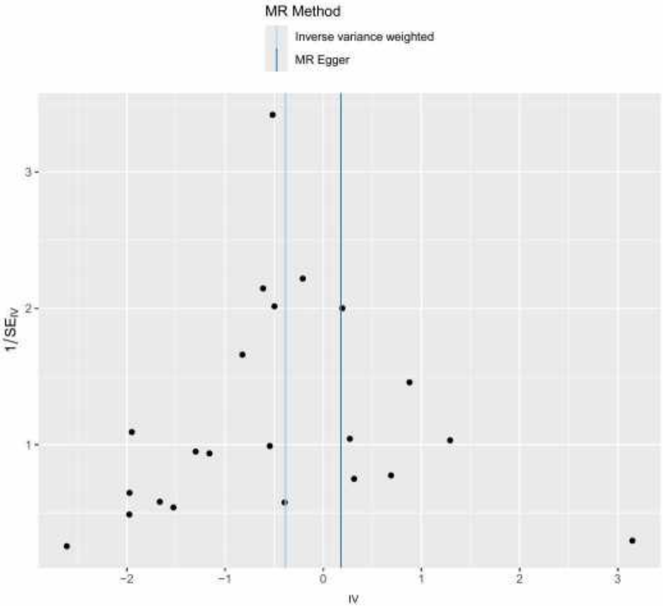
MR Method
Inverse variance weighted
MR Egger
1/SE$_{IV}$
IV

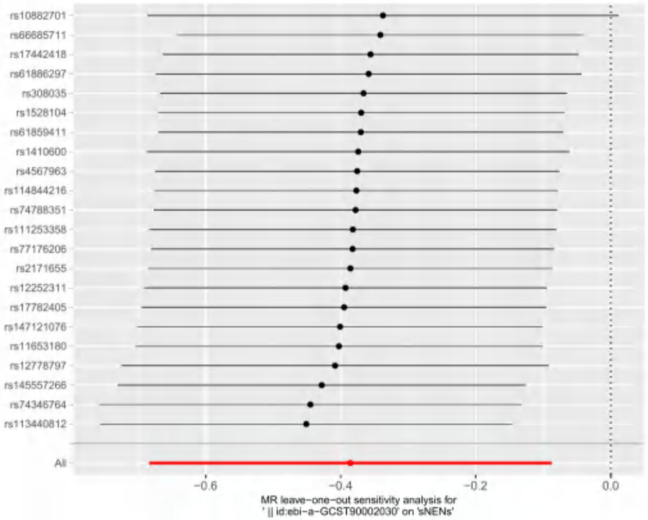
rs10882701
rs66685711
rs17442418
rs61886297
rs308035
rs1528104
rs61859411
rs1410600
rs4567963
rs114844216
rs74788351
rs111253358
rs77176206
rs2171655
rs12252311
rs17782405
rs147121076
rs11653180
rs12778797
rs145557266
rs74346764
rs113440812
All
−0.6
−0.4
−0.2
0.0
MR leave−one−out sensitivity analysis for
' || id:ebi−a−GCST90002030' on 'sNENs'

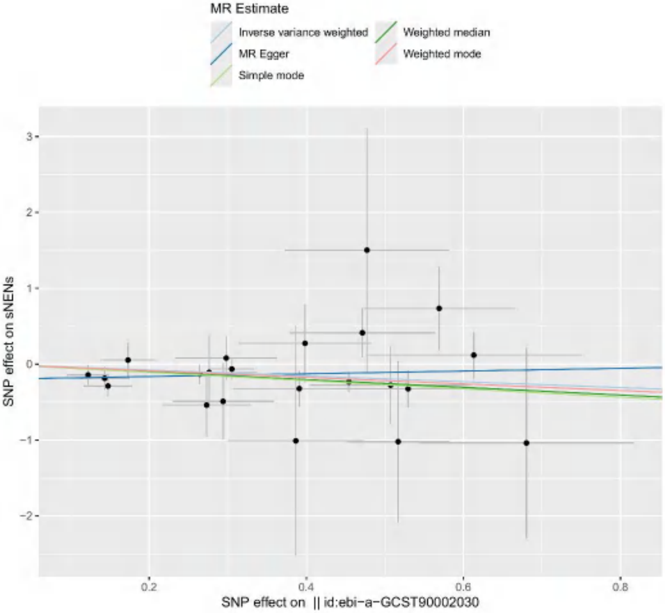
MR Estimate
Inverse variance weighted
MR Egger
Simple mode
Weighted median
Weighted mode
SNP effect on sNENs
SNP effect on || id:ebi−a−GCST90002030

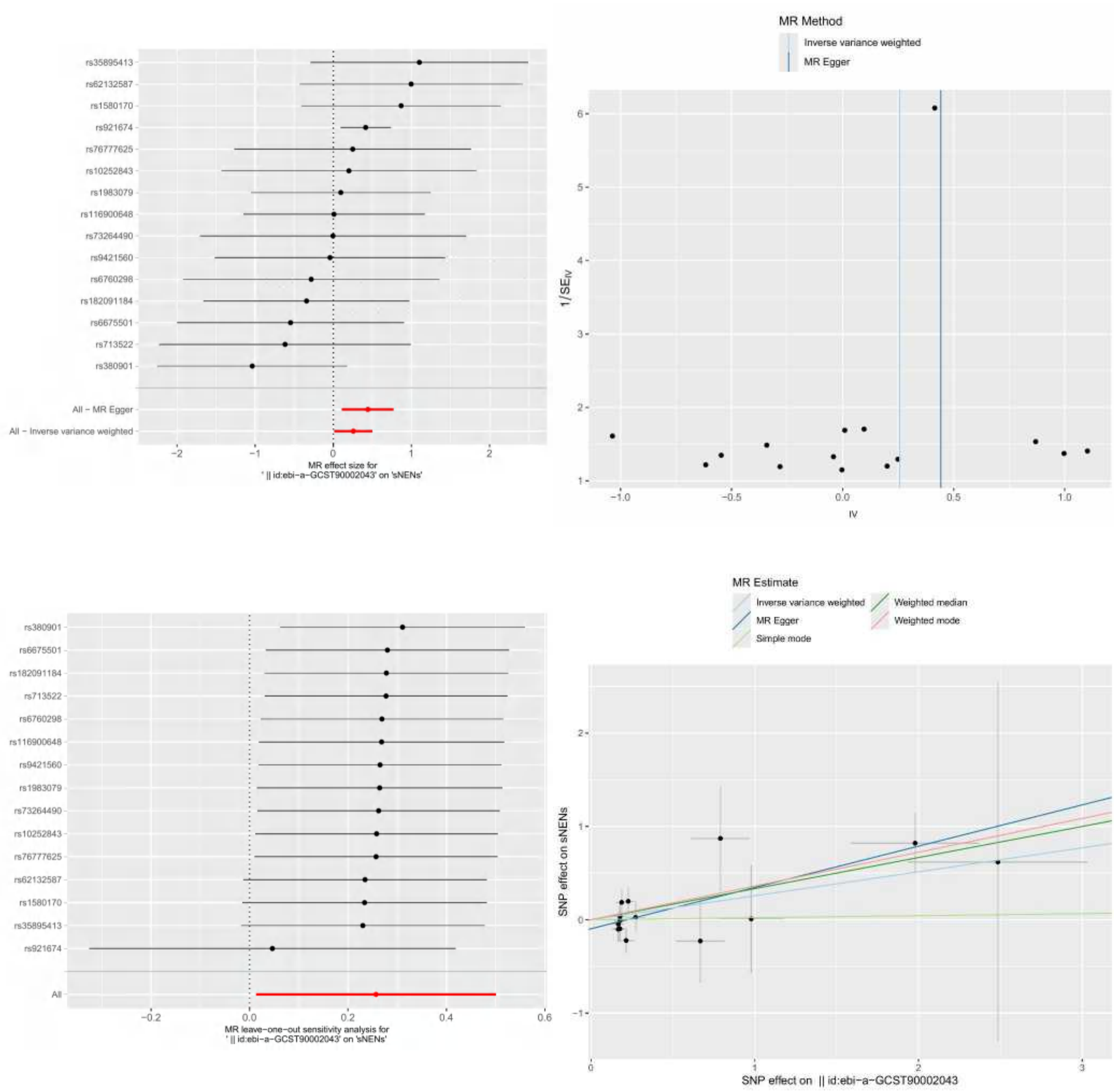

rs35895413
rs62132587
rs1580170
rs921674
rs76777625
rs10252843
rs1983079
rs116900648
rs73264490
rs9421560
rs6760298
rs182091184
rs6675501
rs713522
rs380901
All - MR Egger
All - Inverse variance weighted
MR effect size for
' || id:ebi-a-GCST90002043' on 'sNENs'
MR Method
Inverse variance weighted
MR Egger
1/SE_IV
IV
MR leave-one-out sensitivity analysis for
' || id:ebi-a-GCST90002043' on 'sNENs'
All
MR Estimate
Inverse variance weighted
MR Egger
Simple mode
Weighted median
Weighted mode
SNP effect on sNENs
SNP effect on || id:ebi-a-GCST90002043

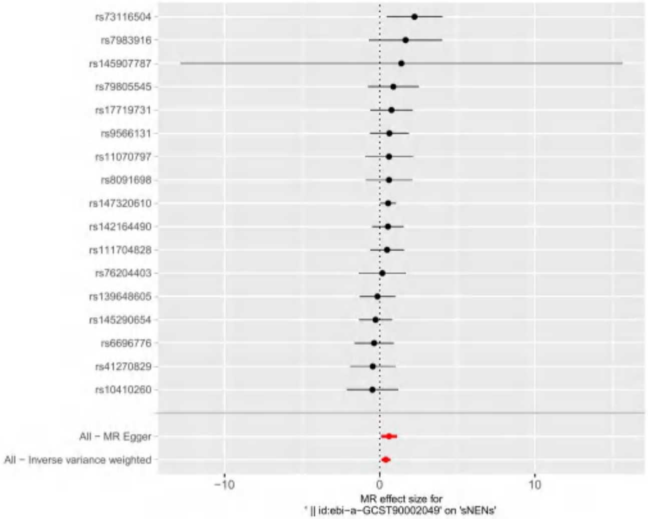
rs73116504
rs7983916
rs145907787
rs79805545
rs17719731
rs9566131
rs11070797
rs8091698
rs147320610
rs142164490
rs111704828
rs76204403
rs139648605
rs145290654
rs6696776
rs41270829
rs10410260
All − MR Egger
All − Inverse variance weighted
−10
0
10
MR effect size for
' || id:ebi−a−GCST90002049' on 'sNENs'

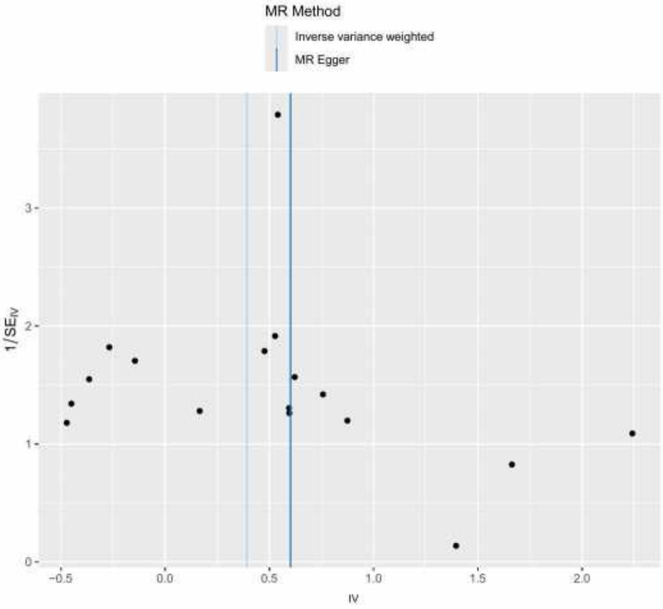
MR Method
Inverse variance weighted
MR Egger
1/SE$_{IV}$
0
1
2
3
−0.5
0.0
0.5
1.0
1.5
2.0
IV

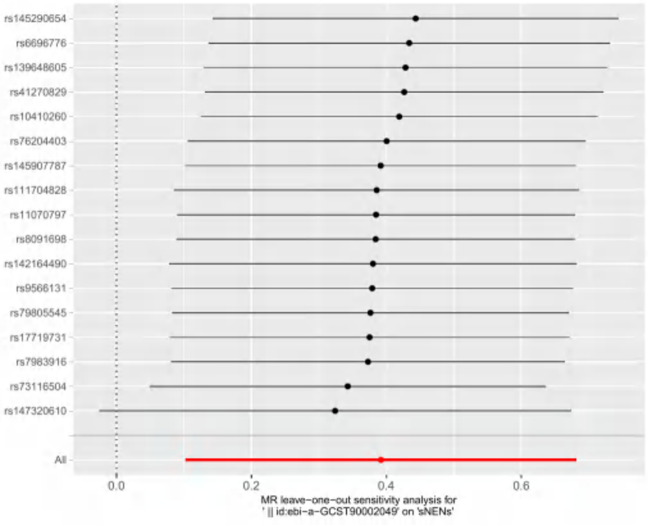
rs145290654
rs6696776
rs139648605
rs41270829
rs10410260
rs76204403
rs145907787
rs111704828
rs11070797
rs8091698
rs142164490
rs9566131
rs79805545
rs17719731
rs7983916
rs73116504
rs147320610
All
0.0
0.2
0.4
0.6
MR leave−one−out sensitivity analysis for
' || id:ebi−a−GCST90002049' on 'sNENs'

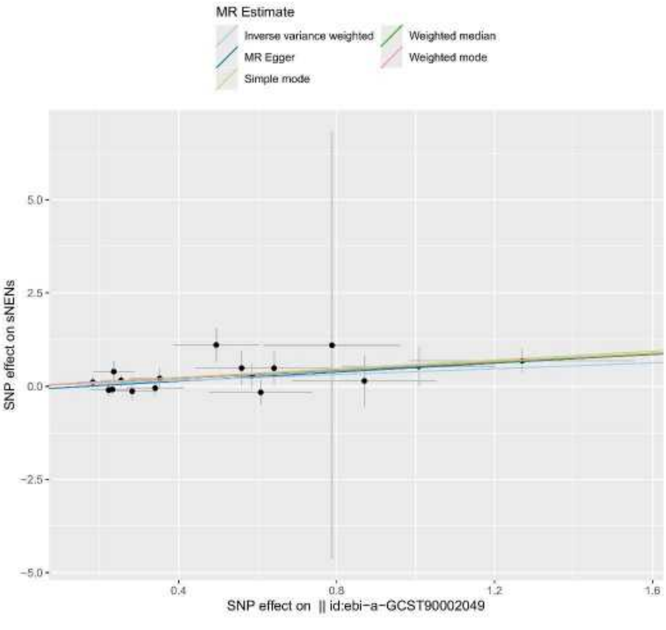
MR Estimate
Inverse variance weighted
MR Egger
Simple mode
Weighted median
Weighted mode
SNP effect on sNENs
5.0
2.5
0.0
−2.5
−5.0
0.4
0.8
1.2
1.6
SNP effect on || id:ebi−a−GCST90002049

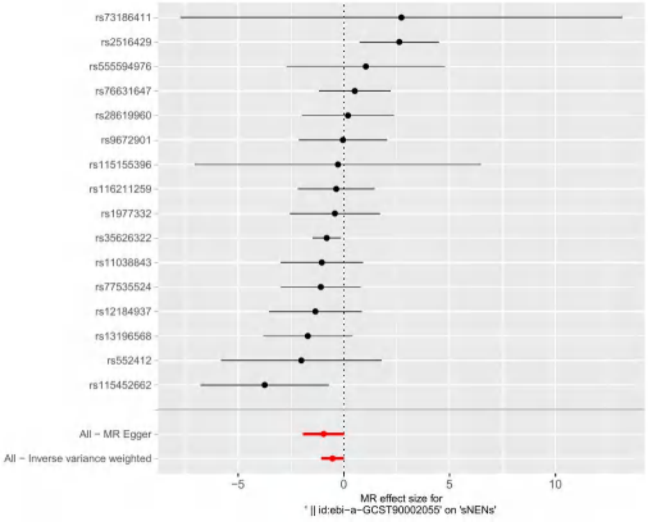
rs73186411
rs2516429
rs555594976
rs76631647
rs28619960
rs9672901
rs115155396
rs116211259
rs1977332
rs35626322
rs11038843
rs77535524
rs12184937
rs13196568
rs552412
rs115452662
All − MR Egger
All − Inverse variance weighted
−5
0
5
10
MR effect size for
' || id:ebi−a−GCST90002055' on 'sNENs'

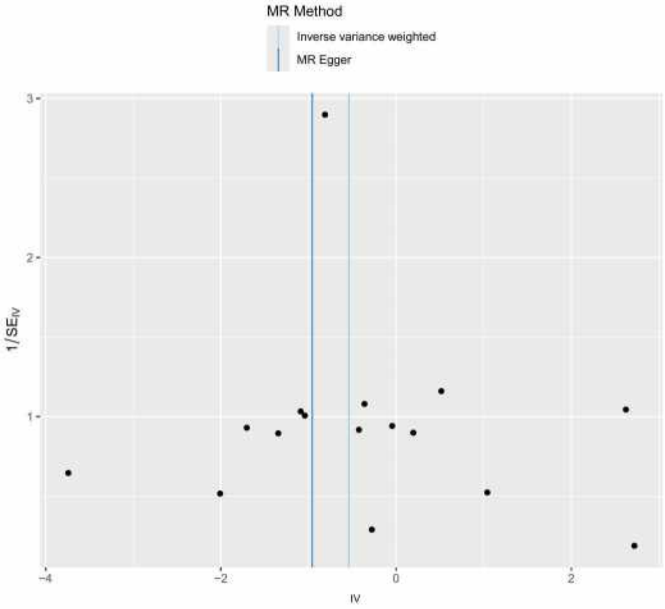
MR Method
Inverse variance weighted
MR Egger
1/SE$_{IV}$
3
2
1
−4
−2
0
2
IV

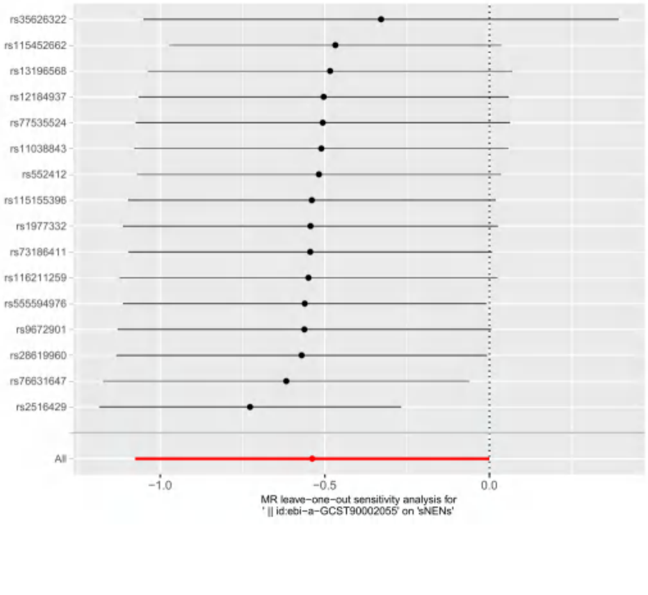
rs35626322
rs115452662
rs13196568
rs12184937
rs77535524
rs11038843
rs552412
rs115155396
rs1977332
rs73186411
rs116211259
rs555594976
rs9672901
rs28619960
rs76631647
rs2516429
All
−1.0
−0.5
0.0
MR leave−one−out sensitivity analysis for
' || id:ebi−a−GCST90002055' on 'sNENs'

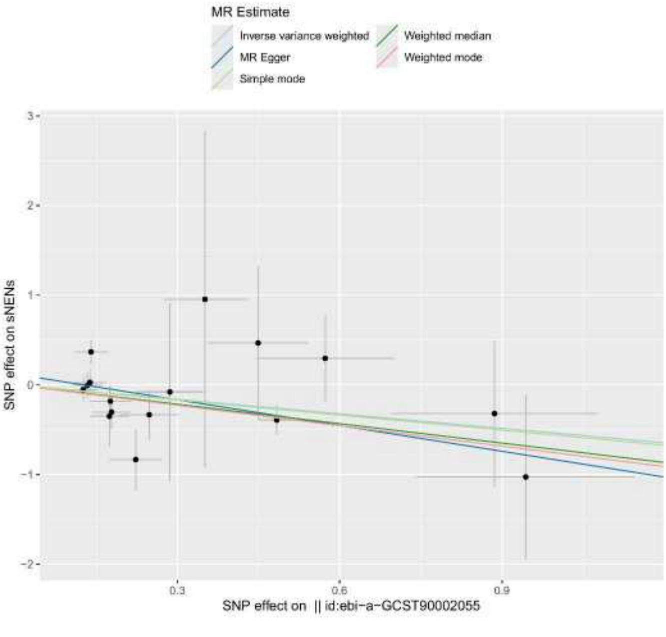
MR Estimate
Inverse variance weighted
MR Egger
Simple mode
Weighted median
Weighted mode
SNP effect on sNENs
3
2
1
0
−1
−2
0.3
0.6
0.9
SNP effect on || id:ebi−a−GCST90002055

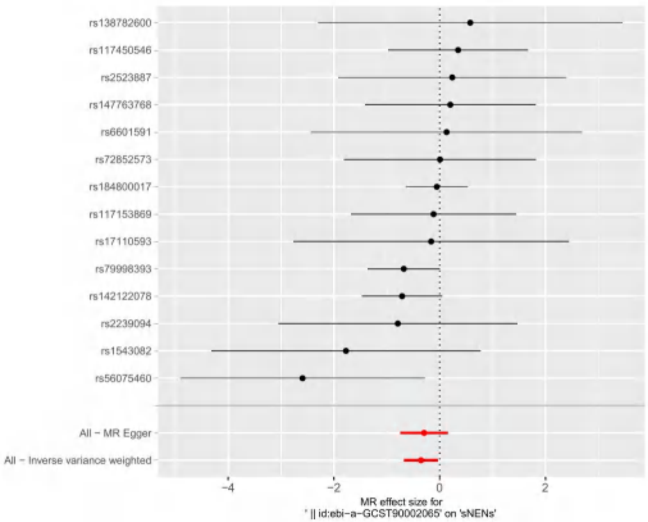
rs138782600
rs117450546
rs2523887
rs147763768
rs6601591
rs72852573
rs184800017
rs117153869
rs17110593
rs79998393
rs142122078
rs2239094
rs1543082
rs56075460
All − MR Egger
All − Inverse variance weighted
−4
−2
0
2
MR effect size for
' || id:ebi−a−GCST90002065' on 'sNENs'

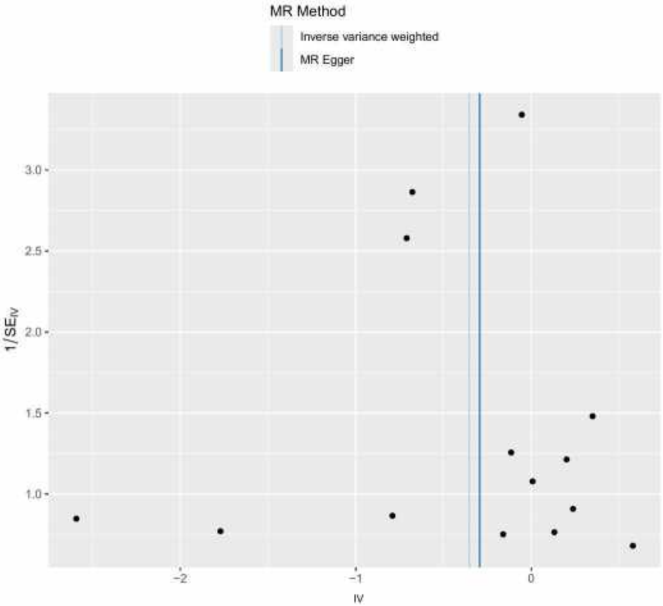
MR Method
Inverse variance weighted
MR Egger
1/SE$_{IV}$
3.0
2.5
2.0
1.5
1.0
−2
−1
0
IV

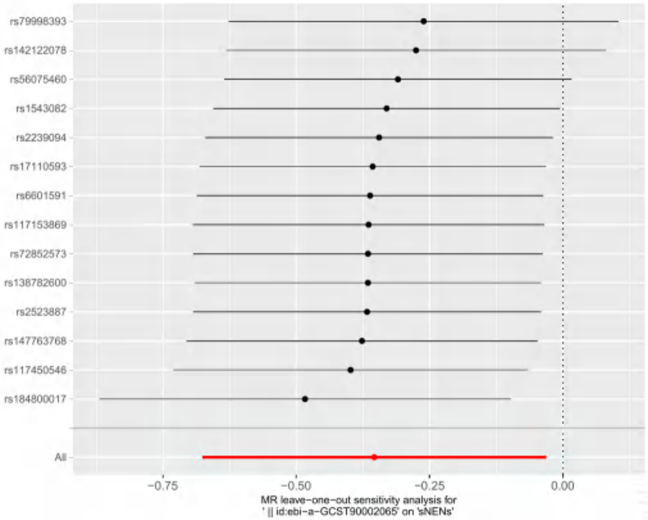
rs79998393
rs142122078
rs56075460
rs1543082
rs2239094
rs17110593
rs6601591
rs117153869
rs72852573
rs138782600
rs2523887
rs147763768
rs117450546
rs184800017
All
−0.75
−0.50
−0.25
0.00
MR leave−one−out sensitivity analysis for
' || id:ebi−a−GCST90002065' on 'sNENs'

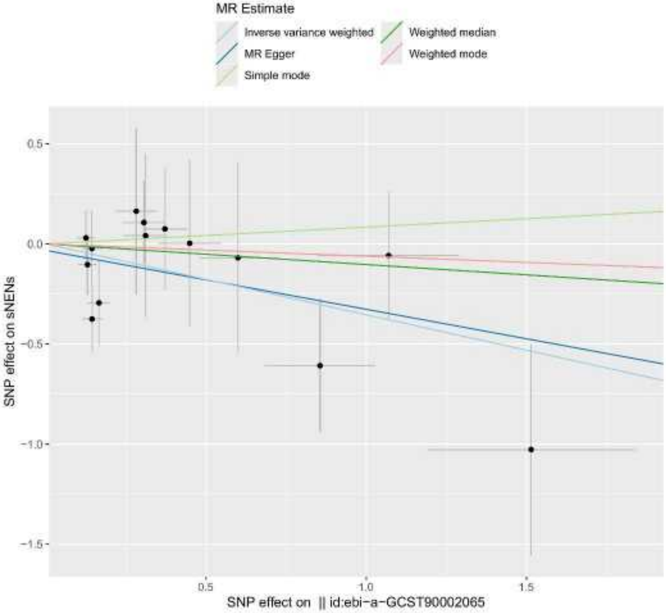
MR Estimate
Inverse variance weighted
MR Egger
Simple mode
Weighted median
Weighted mode
SNP effect on sNENs
0.5
0.0
−0.5
−1.0
−1.5
0.5
1.0
1.5
SNP effect on || id:ebi−a−GCST90002065

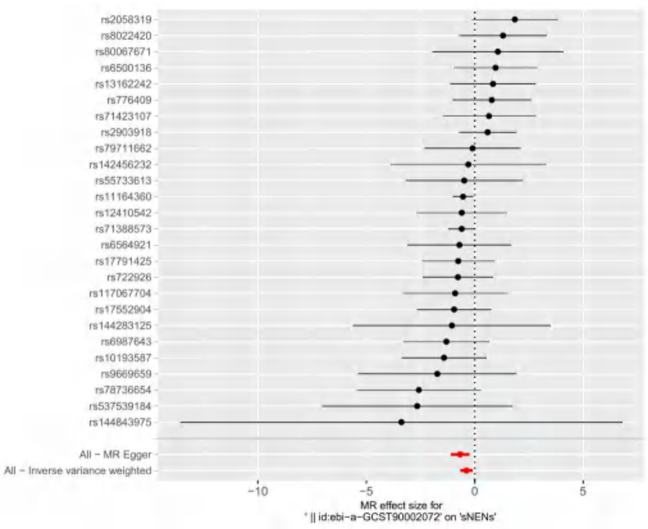
rs2058319
rs8022420
rs80067671
rs6500136
rs13162242
rs776409
rs71423107
rs2903918
rs79711662
rs142456232
rs55733613
rs11164360
rs12410542
rs71388573
rs6564921
rs17791425
rs722926
rs117067704
rs17552904
rs144283125
rs6987643
rs10193587
rs9669659
rs78736654
rs537539184
rs144843975
All − MR Egger
All − Inverse variance weighted
−10
−5
0
5
MR effect size for
' || id:ebi−a−GCST90002072' on 'sNENs'

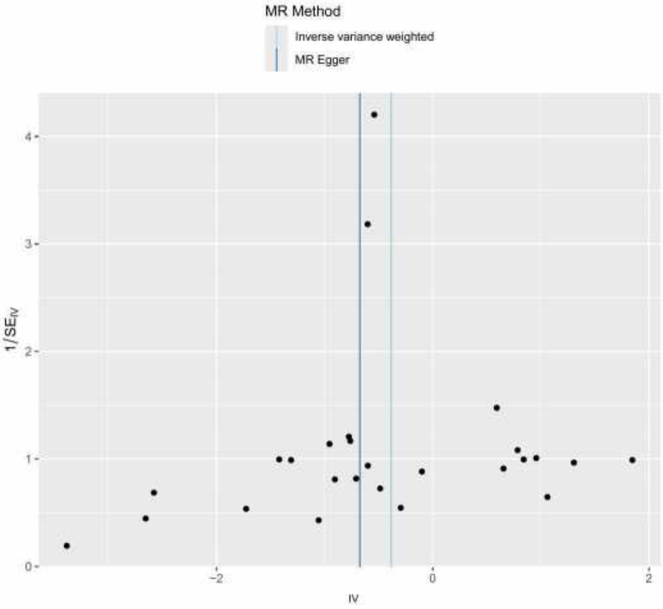
MR Method
Inverse variance weighted
MR Egger
1/SE_{IV}
0
1
2
3
4
−2
0
2
IV

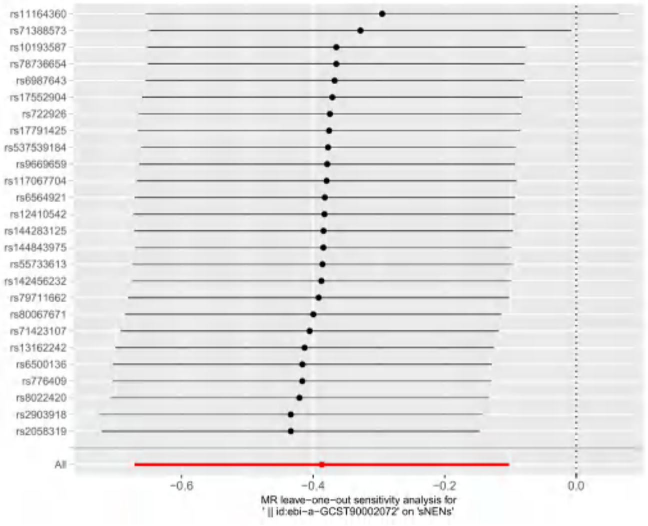
rs11164360
rs71388573
rs10193587
rs78736654
rs6987643
rs17552904
rs722926
rs17791425
rs537539184
rs9669659
rs117067704
rs6564921
rs12410542
rs144283125
rs144843975
rs55733613
rs142456232
rs79711662
rs80067671
rs71423107
rs13162242
rs6500136
rs776409
rs8022420
rs2903918
rs2058319
All
−0.6
−0.4
−0.2
0.0
MR leave−one−out sensitivity analysis for
' || id:ebi−a−GCST90002072' on 'sNENs'

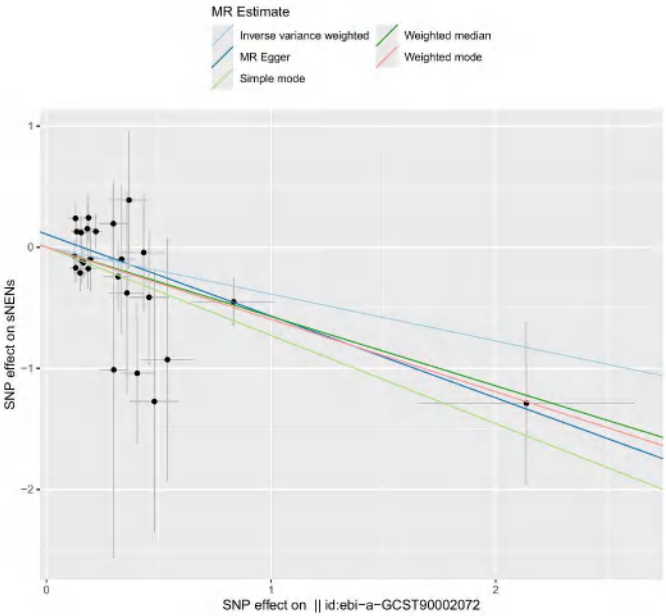
MR Estimate
Inverse variance weighted
MR Egger
Simple mode
Weighted median
Weighted mode
SNP effect on sNENs
1
0
−1
−2
0
1
2
SNP effect on || id:ebi−a−GCST90002072

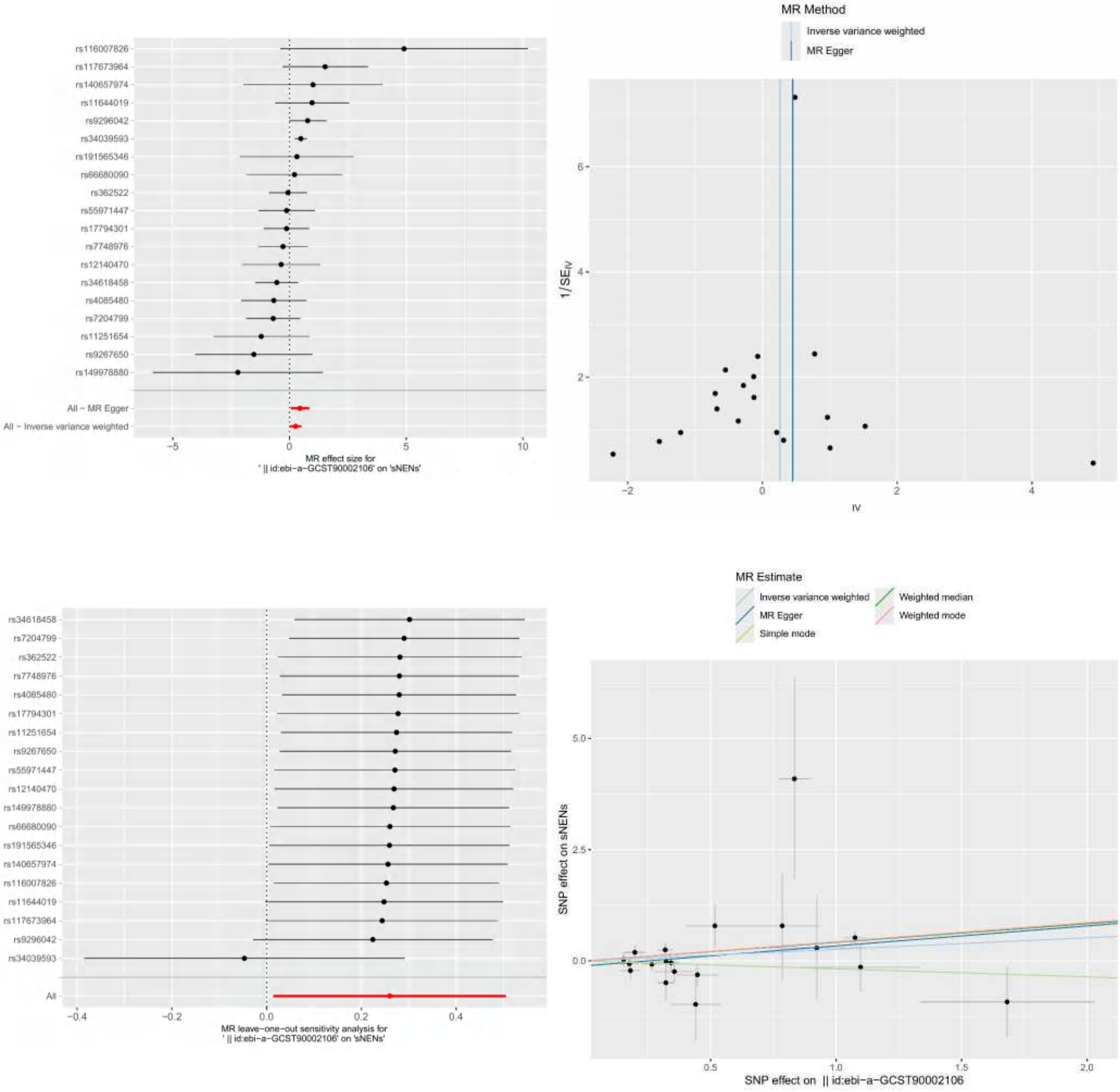
rs116007826
rs117673964
rs140657974
rs11644019
rs9296042
rs34039593
rs191565346
rs66680090
rs362522
rs55971447
rs17794301
rs7748976
rs12140470
rs34618458
rs4085480
rs7204799
rs11251654
rs9267650
rs149978880
All − MR Egger
All − Inverse variance weighted
−5
0
5
10
MR effect size for
' || id:ebi−a−GCST90002106' on 'sNENs'
MR Method
Inverse variance weighted
MR Egger
1/SE_IV
IV
−2
0
2
4
6
rs34618458
rs7204799
rs362522
rs7748976
rs4085480
rs17794301
rs11251654
rs9267650
rs55971447
rs12140470
rs149978880
rs66680090
rs191565346
rs140657974
rs116007826
rs11644019
rs117673964
rs9296042
rs34039593
All
−0.4
−0.2
0.0
0.2
0.4
MR leave−one−out sensitivity analysis for
' || id:ebi−a−GCST90002106' on 'sNENs'
MR Estimate
Inverse variance weighted
MR Egger
Simple mode
Weighted median
Weighted mode
SNP effect on sNENs
5.0
2.5
0.0
0.5
1.0
1.5
2.0
SNP effect on || id:ebi−a−GCST90002106

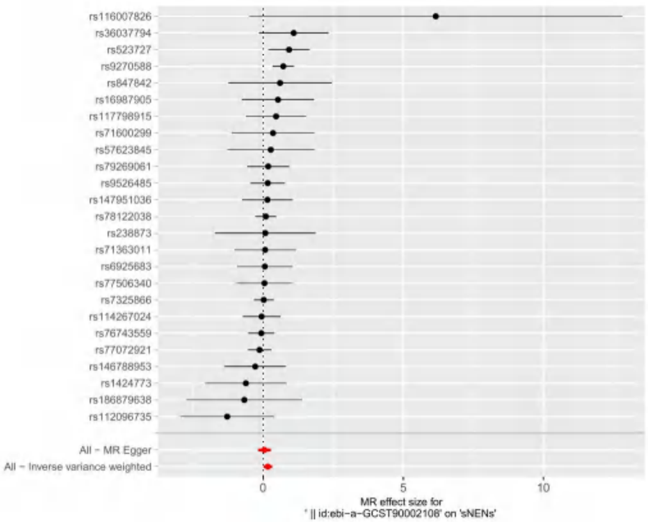

rs116007826
rs36037794
rs523727
rs9270588
rs847842
rs16987905
rs117798915
rs71600299
rs57623845
rs79269061
rs9526485
rs147951036
rs78122038
rs238873
rs71363011
rs6925683
rs77506340
rs7325866
rs114267024
rs76743559
rs77072921
rs146788953
rs1424773
rs186879638
rs112096735
All - MR Egger
All - Inverse variance weighted
0
5
10
MR effect size for
' || id:ebi-a-GCST90002108' on 'sNENs'

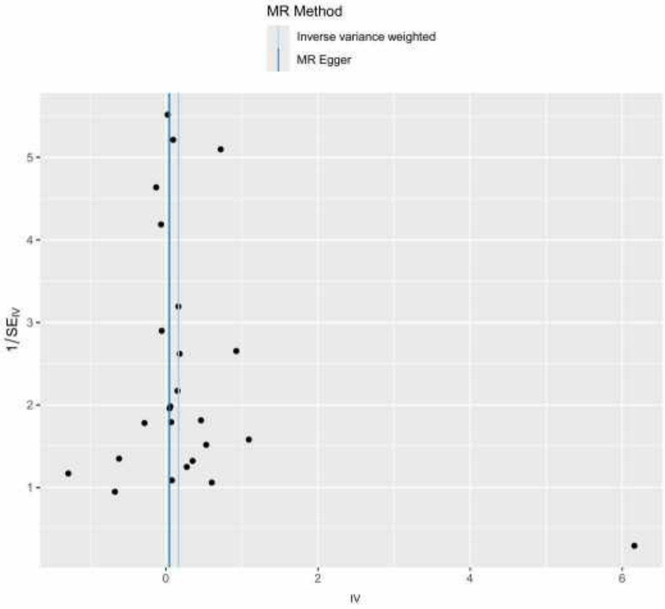

MR Method
Inverse variance weighted
MR Egger
1/SE$_{IV}$
1
2
3
4
5
0
2
4
6
IV

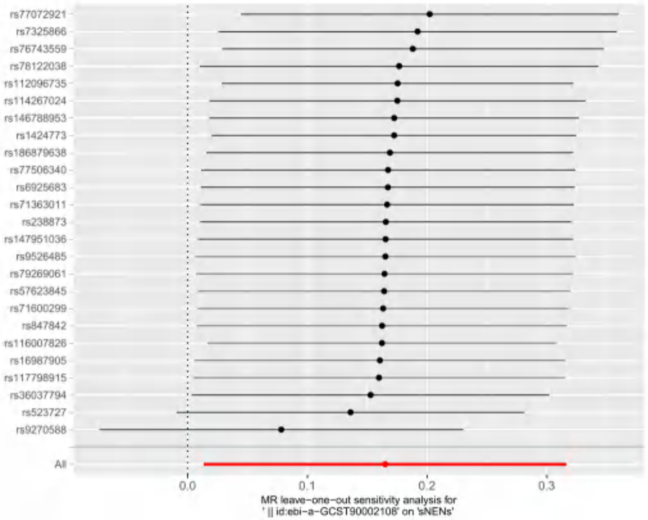

rs77072921
rs7325866
rs76743559
rs78122038
rs112096735
rs114267024
rs146788953
rs1424773
rs186879638
rs77506340
rs6925683
rs71363011
rs238873
rs147951036
rs9526485
rs79269061
rs57623845
rs71600299
rs847842
rs116007826
rs16987905
rs117798915
rs36037794
rs523727
rs9270588
All
0.0
0.1
0.2
0.3
MR leave-one-out sensitivity analysis for
' || id:ebi-a-GCST90002108' on 'sNENs'

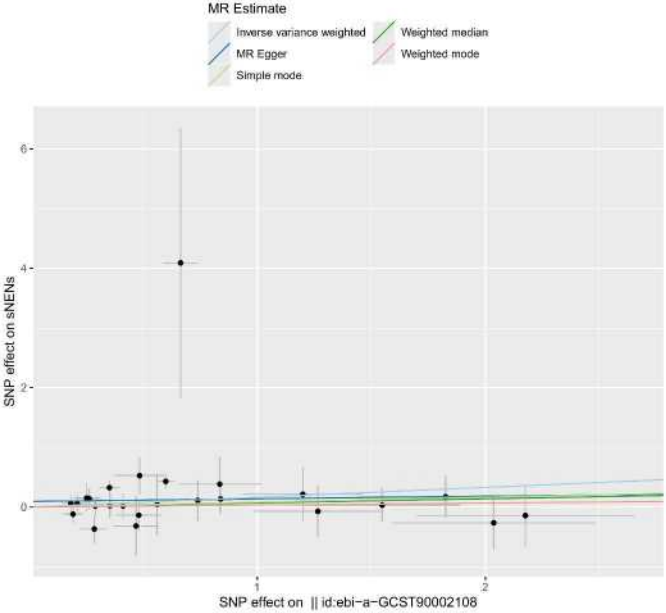

MR Estimate
Inverse variance weighted
MR Egger
Simple mode
Weighted median
Weighted mode
SNP effect on sNENs
0
2
4
6
1
2
SNP effect on || id:ebi-a-GCST90002108

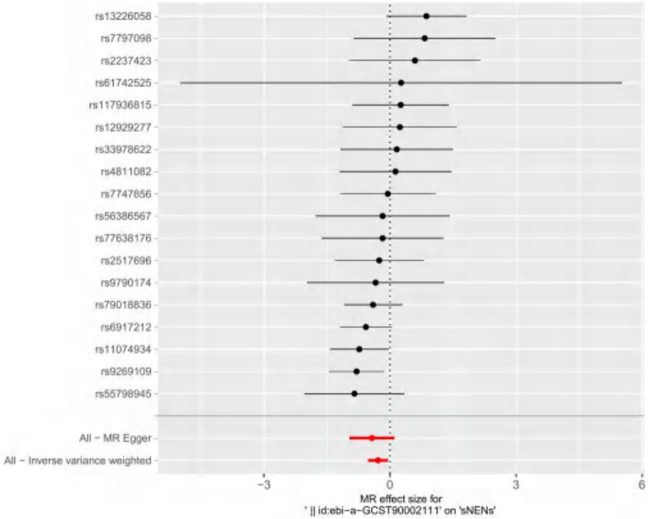
rs13226058
rs7797098
rs2237423
rs61742525
rs117936815
rs12929277
rs33978622
rs4811082
rs7747856
rs56386567
rs77638176
rs2517696
rs9790174
rs79018836
rs6917212
rs11074934
rs9269109
rs55798945
All − MR Egger
All − Inverse variance weighted
−3
0
3
6
MR effect size for
' || id:ebi−a−GCST90002111' on 'sNENs'

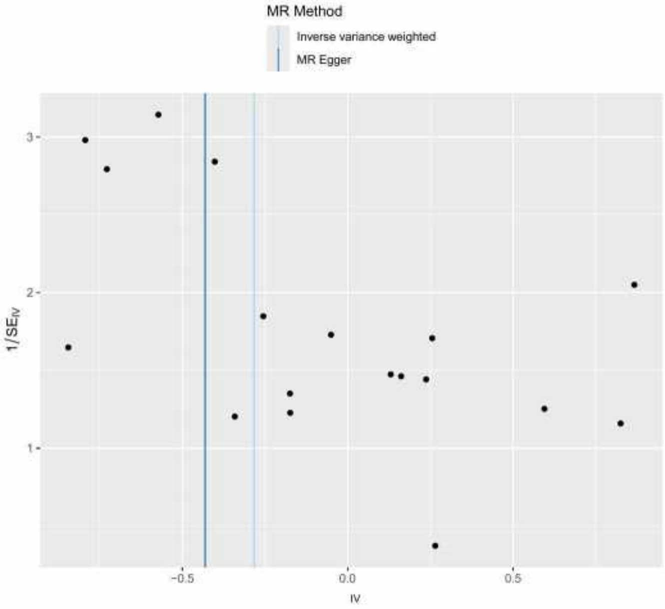
MR Method
Inverse variance weighted
MR Egger
1/SE$_{IV}$
3
2
1
−0.5
0.0
0.5
IV

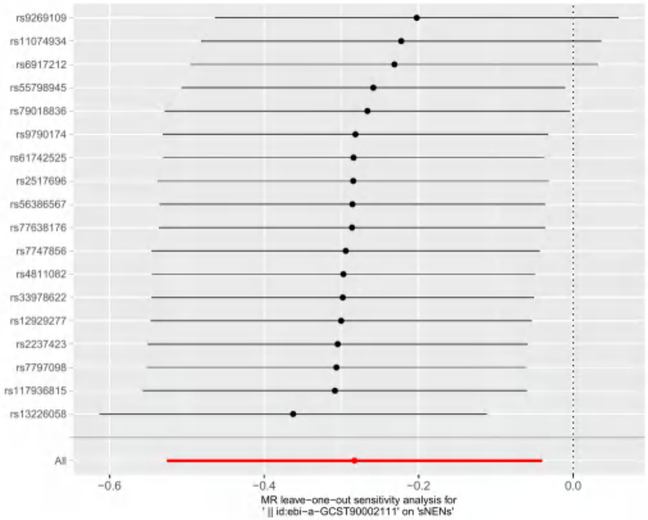
rs9269109
rs11074934
rs6917212
rs55798945
rs79018836
rs9790174
rs61742525
rs2517696
rs56386567
rs77638176
rs7747856
rs4811082
rs33978622
rs12929277
rs2237423
rs7797098
rs117936815
rs13226058
All
−0.6
−0.4
−0.2
0.0
MR leave−one−out sensitivity analysis for
' || id:ebi−a−GCST90002111' on 'sNENs'

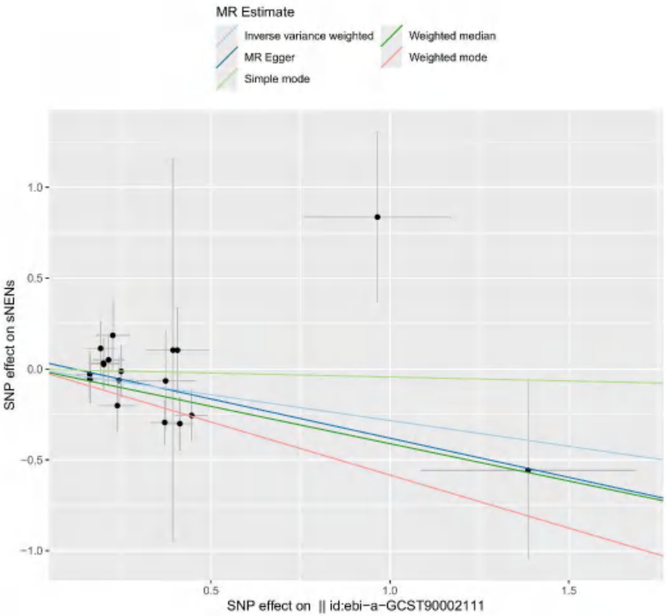
MR Estimate
Inverse variance weighted
MR Egger
Simple mode
Weighted median
Weighted mode
SNP effect on sNENs
1.0
0.5
0.0
−0.5
−1.0
0.5
1.0
1.5
SNP effect on || id:ebi−a−GCST90002111

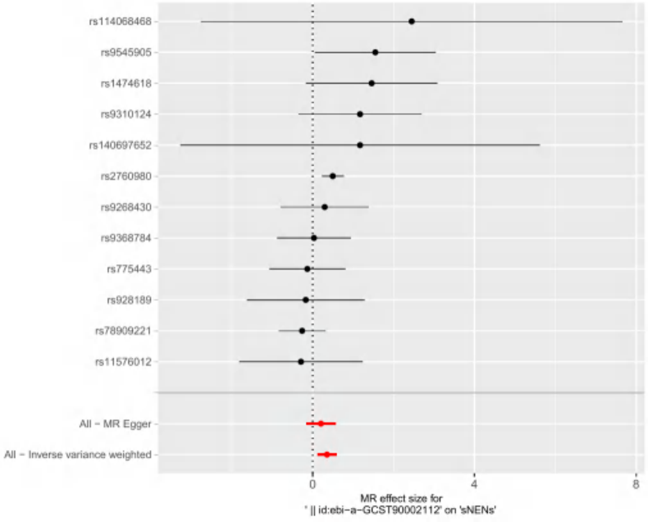
rs114068468
rs9545905
rs1474618
rs9310124
rs140697652
rs2760980
rs9268430
rs9368784
rs775443
rs928189
rs78909221
rs11576012
All - MR Egger
All - Inverse variance weighted
0
4
8
MR effect size for
' || id:ebi-a-GCST90002112' on 'sNENs'

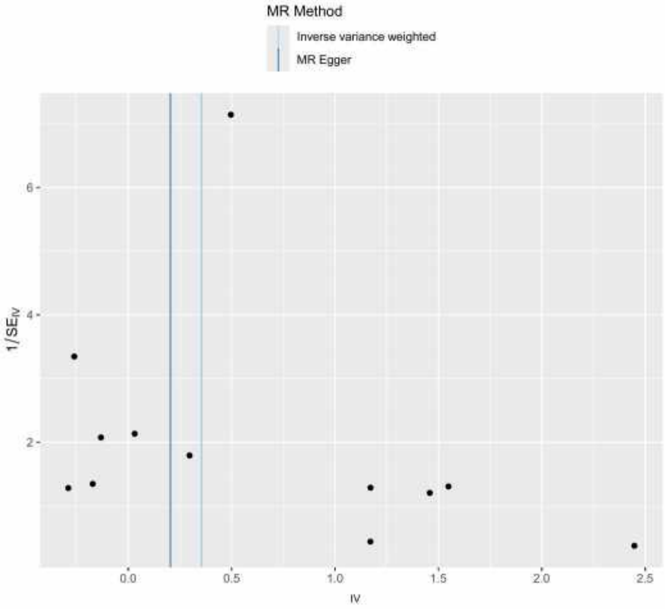
MR Method
Inverse variance weighted
MR Egger
$1/SE_{IV}$
2
4
6
0.0
0.5
1.0
1.5
2.0
2.5
IV

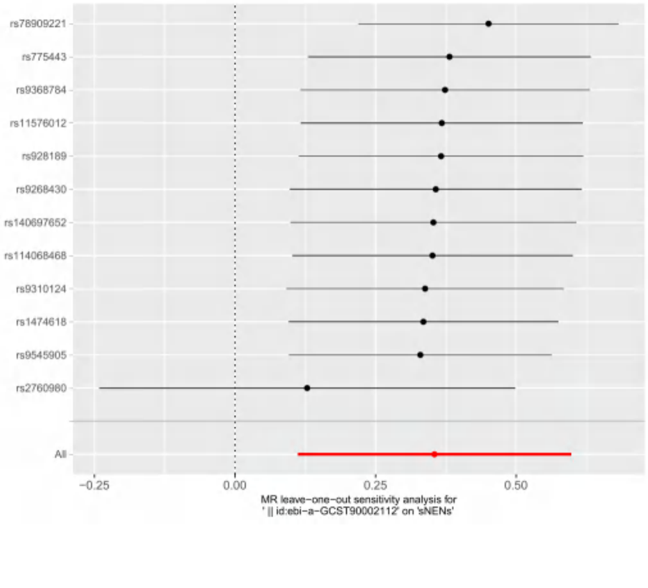
rs78909221
rs775443
rs9368784
rs11576012
rs928189
rs9268430
rs140697652
rs114068468
rs9310124
rs1474618
rs9545905
rs2760980
All
−0.25
0.00
0.25
0.50
MR leave−one−out sensitivity analysis for
' || id:ebi-a-GCST90002112' on 'sNENs'

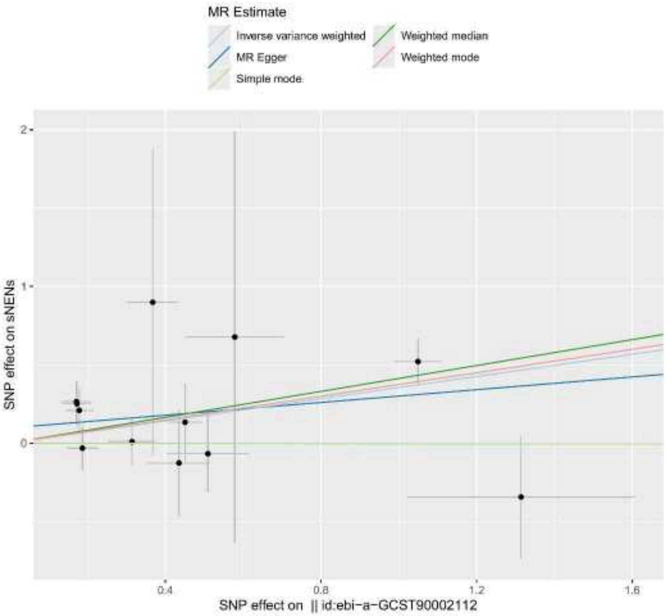
MR Estimate
Inverse variance weighted
MR Egger
Simple mode
Weighted median
Weighted mode
SNP effect on sNENs
0
1
2
0.4
0.8
1.2
1.6
SNP effect on || id:ebi-a-GCST90002112
